# Path finding methods accounting for stoichiometry in metabolic networks

**DOI:** 10.1186/gb-2011-12-5-r49

**Published:** 2011-05-27

**Authors:** Jon Pey, Joaquín Prada, John E Beasley, Francisco J Planes

**Affiliations:** 1 CEIT and TECNUN, University of Navarra, Manuel de Lardizabal 15, 20018 San Sebastian, Spain; 2 Mathematical Sciences Department, Brunel University, Kingston Lane, UB8 3PH, Uxbridge, UK

## Abstract

Graph-based methods have been widely used for the analysis of biological networks. Their application to metabolic networks has been much discussed, in particular noting that an important weakness in such methods is that reaction stoichiometry is neglected. In this study, we show that reaction stoichiometry can be incorporated into path-finding approaches via mixed-integer linear programming. This major advance at the modeling level results in improved prediction of topological and functional properties in metabolic networks.

## Background

The use of graph theory in the analysis of biological networks has been extensive in the past decade [1]. Particularly, in metabolic networks different relevant topics have been examined using the rich variety of graph-theoretic concepts, ranging from topological properties [2-5], evolutionary analysis [6-8], pathway analysis [9-13], transcriptional regulation [14-16], functional interpretation of 'omics' data [17-20] and prediction of novel drug targets [21-23].

Graph-based methods start by converting the metabolic network into an appropriate graph. Different representations are possible here: i) metabolite graphs, where nodes are metabolites and arcs represent reactions linking an input and output metabolite; ii) reaction graphs, in which nodes are reactions and arcs represent intermediate metabolites shared by reactions; iii) bipartite graphs, where nodes are reactions and metabolites, while arcs link metabolites to reactions (for substrates) and reactions to metabolites (for products). Note here that each type of graph can be either directed or undirected. A deeper introduction to such graphs can be found in Deville *et al. *[24].

Importantly, graph-based methods rely on the definition of connectivity based on paths, that is, two nodes in the graph are connected (or not) depending upon whether (or not) we have a path linking them. This definition of connectivity is debatable, however, particularly when it is claimed that such a path is a competent metabolic pathway, as recently discussed [25-27]. In this context, the major criticism raised as to path-finding methods is that they neglect reaction stoichiometry and there is, therefore, no guarantee that any path found can operate in sustained steady-state.

The steady-state condition requires the definition of the boundary of the metabolic network under study. Metabolites inside the boundary of the network, typically called internal metabolites [28], must be in stoichiometric balance. Balancing does not apply to metabolites outside the boundaries of the system (external metabolites), which are typically input/output metabolites and (sometimes) cofactors. In other words, for internal metabolites, their production and consumption (if possible) must be captured with the reactions in the network under study.

The steady-state condition and its underlying boundary definition are critical for the performance of any method for analyzing a metabolic network and ignoring it may provide misleading insights. A nice illustration of this is the one presented in the work of de Figueiredo *et al. *[25], which (unsuccessfully) tested the ability of path-finding methods to answer the question as to whether (or not) fatty acids can be converted into sugars. Klamt *et al. *[29] also recently emphasized this issue for different biological networks.

Note here that elementary flux modes (and extreme pathways) represent a more general and elegant concept for metabolic pathways than paths [28,30]. Their computation is, however, much more expensive in large metabolic networks than paths and, though different efforts have been made in this area [31-33], much research is still needed to make elementary flux modes a practical tool for the analysis of large metabolic networks.

Given the limitations discussed above, a novel theoretical concept termed flux paths is introduced here. A flux path is a simple path (in the graph-theoretical sense, so no nodes revisited) from a source metabolite to a target metabolite able to operate in sustained steady-state. In essence, flux paths incorporate reaction stoichiometry into traditional path-finding methods [4,7,34,35]. By means of this concept we show that the path structure of metabolic networks is substantially altered when stoichiometry is considered. In addition, we illustrate (with several examples) that flux paths offer new perspectives for the analysis of metabolic networks at the topological and functional levels. The determination of flux paths requires going beyond graph theory via mixed-integer linear programming. We present below details as to our mathematical optimization model for determining K-shortest flux paths between source and target metabolites.

## Results and discussion

### Mathematical model

Assume we have a metabolic network that comprises R reactions and C metabolites. Note here that reversible reactions contribute two different reactions to the metabolic network. For this reason we can regard all fluxes as taking positive values. Let S_cr _be the stoichiometric coefficient associated with metabolite c (c = 1,...,C) in reaction r (r = 1,...,R). As usual in the literature [28], input metabolites have a negative stoichiometric coefficient, whilst output metabolites have a positive stoichiometric coefficient.

**Figure 1:**
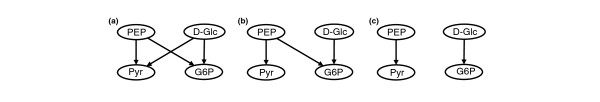
**Metabolite graph representation of the PEP: Pyr uptake system of glucose**. **(a) **Metabolite graph; **(b) **metabolite graph restricted to atomic exchanges; **(c) **metabolite graph restricted to carbon exchanges. D-Glc, glucose; G6P, glucose 6-phosphate; PEP, phosphoenolpyruvate; Pyr, pyruvate.

We here used a metabolite (directed) graph representation of the network where nodes are metabolites and arcs link the input and output metabolites of each reaction. Figure 1a shows an example of the metabolite graph representation of the phosphoenolpyruvate (PEP): pyruvate (Pyr) phosphotransferase system for the uptake of glucose.

Suppose that we are concerned with finding a flux path from a source metabolite α to a target metabolite β. As mentioned above, a flux path is a simple path from the source metabolite α to the target metabolite β able to operate in steady-state. We present below our mathematical optimization model for flux paths.

#### Path finding constraints

We need to decide the arcs involved in the flux path from the source metabolite α to the target metabolite β. This fact is represented with a zero-one (binary) variable u_ij_, where u_ij _= 1 if the arc i→j linking metabolite i (i = 1,...,C) to metabolite j (j = 1,...,C) is active in the flux path, 0 otherwise.

Deletion of arcs from the metabolic graph is standard practice in path-finding methods [4,7,34,35]. We removed arcs not involving an effective carbon exchange. Carbon exchange is indeed essential for metabolic purposes. For this reason, we henceforth use the term carbon flux paths (CFPs).

Note here that a similar criterion has been used in [35]. In this work, however, input and output metabolites can have any type of atom or atom groups in common. This criterion is illustrated in Figure 1b, where PEP donates a phosphate group to glucose (D-Glc). The focus on carbon atoms makes our approach more restrictive, as observed in Figure 1c, which shows that there is only effective carbon exchange between D-Glc and glucose 6-phosphate (G6P), and PEP and Pyr.

Let d_ijr _be a binary (0/1) coefficient establishing whether (or not) there exists an effective carbon exchange between input metabolite i (S_ir _< 0) and output metabolite j (S_jr _> 0) in reaction r. If , so there is no reaction involving metabolites i to j in carbon exchange, then u_ij _is also fixed to zero.

In the following lines we present constraints needed to obtain an appropriate directed path from the source metabolite (α) to the target metabolite (β). Equation 1 ensures that one arc leaves α and one arc enters β; equation 2 that no arc enters α and no arc leaves β:(1)(2)

Equation 3 ensures that the number of arcs entering a metabolite k is equal to the number leaving; Equation 4 ensures that a metabolite cannot be revisited in the path:(3)(4)

#### Stoichiometric constraints

Equations 1 to 4 define a simple path that preserves carbon exchange in each of its intermediate steps. We need to guarantee that this path can work in sustained steady-state. As will be shown below, to do this, it is required to find a steady-state flux distribution able to involve the path. We here introduce variables and constraints needed to define the steady-state flux space.

Any steady-state flux distribution satisfies Equation 5 for the set of internal metabolites (I). We denote v_r _the non-negative (continuous) flux associated with each reaction, r = 1,...,R:(5)

External metabolites (E) are not subject to balancing constraints. If a specific growth medium (E_m_) is introduced, however, metabolites not involved in such a medium can be produced, but cannot be consumed, as observed in Equation 6:(6)

For convenience, we introduced a zero-one (binary) variable z_r _(r = 1,...,R), which defines the reactions involved in a steady-flux distribution, namely z_r _= 1 if reaction r has a non-zero flux, 0 otherwise (r = 1,...,R). We need constraints relating the reaction variables z_r _and the flux variables v_r_. Equation 7 ensures that no flux traverses a reaction r if z_r _= 0:(7)

In addition, it guarantees that v_r _is non-zero if z_r _= 1. Here we have scaled fluxes so that the maximum flux is M and the minimum (non-zero) flux is 1. This does not constitute an issue if we consider M sufficiently large.

As we split reversible reactions into two irreversible steps, we need to prevent a reaction and its reverse from appearing together in any steady-state flux distribution, as observed in Equation 8, where the set B = {(λ,μ)| reaction λ and reaction μ are the reverse of each other}:(8)

Current path-finding approaches deal with this situation indirectly, namely by removing computed paths involving a reaction and its reverse.

Equations 5 to 8 define the steady-state flux space for a particular metabolic network.

#### Linking path finding and stoichiometric constraints

As noted above, it is required that the path defined by constraints 1 to 4 can operate in a steady-state flux distribution. For this purpose, we need to guarantee that if we use an arc i→j in a path, then some reaction r with d_ijr _= 1, that is, involving effective carbon exchange between i and j, is contained in the steady-state flux distribution. This is a critical point in our formulation, which makes it different from previous path-finding methods. With this condition we naturally link the topological and (steady-state) flux planes. This linking constraint is reflected in Equation 9:(9)

Equation 9 ensures that if an arc i→j is active in the CFP (so u_ij _= 1), then at least one reaction r containing this arc in carbon exchange (so d_ijr _= 1) is forced to be active. By forcing z_r _to be 1 there will be a non-zero flux associated with the reaction due to Equation 7. An important point to note from Equation 9 is that it allows reactions to be active even if they are not involved in the CFP. In other words reactions can be active with non-zero flux (to satisfy the requirements of steady-state, Equation 5) but without any of their input/output metabolites being involved in the CFP.

**Figure 2:**
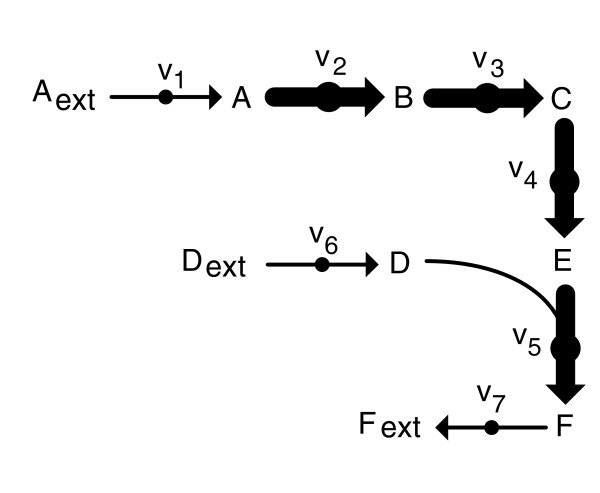
**Example flux path in a toy metabolic network**.

To illustrate constraint 9, consider the example metabolic network in Figure 2, which involves seven reactions and nine metabolites. The set of internal metabolites is I = {A,B,C,D,E,F}. Assume now that we are concerned with finding a CFP between metabolites A and F. We have only one possible path, namely A→B→C→E→F (u_AB _= u_BC _= u_CE _= u_EF _= 1). Due to Equation 9, reactions 2, 3, 4 and 5 are active, that is, z_2 _= z_3 _= z_4 _= z_5 _= 1 and, therefore, via Equation 7, their flux will be non-zero. To balance such a path and satisfy the steady-state condition, Equation 5, we require three additional reactions off-path: reaction 1 for the production of A, reaction 7 to consume F and reaction 6 to produce D. If these off-path reactions are active, the path from A to F is able to work in sustained steady-state and, therefore, it is a flux path, as denoted in the Background section. We are obviously considering that A_ext _and D_ext _are in the growth medium. If we remove one of these metabolites from the medium, though we still have a path from A to F at the graph-theoretical level, no flux path will exist, since the path cannot work in sustained steady-state.

#### Objective function

Equations 1 to 9 define the set of constraints for the determination of any CFP between metabolite α and metabolite β. However, our purpose here is to find the shortest CFP, as observed in Equation 10:(10)

#### Enumerating constraint

As in other path-finding approaches, we may be interested in computing not only the shortest CFP, but the k-shortest CFPs (k = 1,..,K). Since we have an objective relating to finding the shortest CFP, we need to add constraints eliminating previously found CFPs, as shown in Equation 11. In that constraint  is the binary solution value for the u_ij _variable in the k-shortest CFP:(11)

### Validation

This section is organized as follows. By means of several well-documented examples, we first illustrate the biochemical relevance of particular constraints in our CFP approach. We then carry out a side-by-side comparison of our CFP approach with current path-finding approaches.

#### Path-finding comparison

As shown in the 'Mathematical model' section, the path-finding strategy used in our CFP approach is based on using arcs involving effective carbon exchange and imposing the reversibility constraint, Equation 8. In this sub-section we illustrate the importance of these factors and show that a path-finding approach incorporating them outperforms existing methods in the literature. For this analysis, the effect of stoichiometry is not considered, as is common in existing approaches. Its effect will be separately considered in detail in the next sub-section ('Effect of stoichiometry'). Therefore, for this analysis, Equations 5 and 6 were ignored.

##### Effective carbon exchange

**Figure 3:**
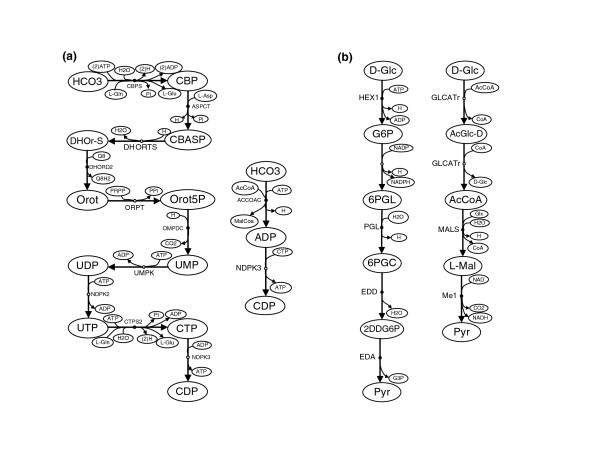
**Effect of carbon exchange and reversibility constraints**. **(a) ***De novo *biosynthesis of pyrimidine ribonucleotides in *E. coli *discussed in Faust *et al. *[13]. **(b) **Shortest pathway from glucose to pyruvate in *E. coli*. 2DDG6P, 2-Dehydro-3-deoxy-D-gluconate 6-phosphate; 6PGC, 6-Phospho-D-gluconate; 6PGL, 6-phospho-D-glucono-1,5-lactone; AcCoA, Acetyl-CoA; ACCOAC, acetyl-CoA carboxylase; AcGlc-D, 6-Acetyl-D-glucose; ASPCT, aspartate carbamoyltransferase; CBASP, N-Carbamoyl-L-aspartate; CBP, Carbamoyl phosphate; CBPS, carbamoyl-phosphate synthase; CDP, cytidine-diphosphate; CTPS2, CTP synthase; D-Glc, D-Glucose; DHOr-S, (S)-Dihydroorotate; DHORTS, dihydroorotase; DHORD2, dihydoorotic acid dehydrogenase; EDA, 2-dehydro-3-deoxy-phosphogluconate aldolase; EDD, 6-phosphogluconate dehydratase; G6PDH2r, glucose 6-phosphate dehydrogenase; GLCATr, D-glucose O-acetyltransferase; G3P, Glyceraldehyde 3-phosphate; G6P, D-Glucose 6-phosphate; GLX, Glyoxylate; HCO3, Bicarbonate; HEX1, hexokinase; L-Asp, L-Aspartate; L-Glu, L-Glutamate; L-Gln, L-Glutamine; L-Mal, L-Malate; OMPDC, orotidine-5'-phosphate decarboxylase; ORPT, orotate phosphoribosyltransferase; MalCoa, Malonyl-CoA; MALS, malate synthase; ME1, malic enzyme; NDPK2, nucleoside-diphosphate kinase; NDPK3, nucleoside-diphosphate kinase; Orot, Orotate; Orot5P, Orotidine 5'-phosphate; PGL 6-phosphogluconolactonase; PRPP, 5-Phospho-alpha-D-ribose 1-diphosphate; Pyr, Pyruvate; UMPK, UMP kinase.

Figure 3a shows two paths from bicarbonate (HCO3) to cytidine-diphosphate (CDP) in *Escherichia coli*. The long path is a well-known (canonical) metabolic pathway for *de novo *pyrimidine biosynthesis. The short path is a shortcut via ADP, which has no biological relevance. The removal of arcs not involving carbon exchange, as done in our CFP approach, considerably reduces the appearance of such non-meaningful paths. Indeed, when we applied our approach to find a CFP from HCO3 to CDP to the genome-scale metabolic network of *E. coli *[36], the long pathway was directly recovered. Note here that we manually removed arcs not involving carbon exchange in the network of Feist *et al. *[36]. The resulting list of arcs can be found in Additional file 1. This same biochemical example was recently discussed in Faust *et al. *[13], under different strategies. In the best case scenario, they require additional information as to the intermediate metabolites to recover this pathway. The fact that our approach can recover the pathway without intermediate metabolite information shows how effective the carbon exchange constraint is.

##### Reversibility

Path-finding methods typically split reversible reactions into two irreversible steps. In contrast to current approaches [13], in our CFP approach we prevent two such irreversible steps from being active in the same path, as observed in Equation 8. To illustrate the importance of this constraint, we analyzed the shortest path from D-Glc to Pyr in *E. coli*, which is the Entner-Doudoroff pathway, as shown in the left-hand side of Figure 3b. When we applied our CFP approach from D-Glc to Pyr without including Equation 8, we obtained the path in the right hand-side of Figure 3b (D-Glc→AcGlc-D→AcCoA→L-Mal→Pyr). This solution has no biochemical meaning, since the first and second step in that path is a cycle involving the forward and backward step of the reversible reaction catalyzed by D-glucose O-acetyltransferase (GLCATr: D-Glc + AcCoA ↔ AcGlc-D + CoA). By adding Equation 8 this path is removed from the solution space and our CFP approach directly obtains the Entner-Doudoroff pathway.

##### Side-by-side comparison

In order to analyze the performance of any path-finding method, it is usual in the literature to evaluate its ability in recovering well-known metabolic pathways. For this purpose, we used a database of 40 reference *E. coli *(metabolic) pathways previously discussed in Planes and Beasley [37] (these 40 pathways are listed in Additional file 2).

The input metabolic graph was built from the genome-scale metabolic network of *E. coli *[36]. We computed the 100 shortest CFPs between the source and target metabolites of each of the 40 reference pathways. As mentioned above, stoichiometric constraints are not considered in this sub-section since the aim is to establish the effectiveness of carbon exchange when combined with reversibility in path finding. To compare the 100 shortest CFPs and the reference pathway, we used the recovery rate. Recovery is a 0/1 parameter, being 1 if a CFP fully matches with the reference pathway, 0 otherwise.

A similar analysis was conducted for existing path-finding methods [4,7,34,35]. These methods make use of different strategies to provide biochemical meaning to the computed paths. For comparison, we classified these strategies into different groups: the first strategy (denoted 'topology') involves the use of an unadjusted metabolic graph; the second strategy (denoted 'hubs') adjusts the metabolic graph by removing any arc involving a highly connected metabolite (hubs) [7,34] (we took the list of hubs from Planes and Beasley [37]); the third strategy (denoted 'connectivity') assigns weights to metabolites according to their connectivity in an unadjusted metabolic graph, where connectivity is defined to be the number of reactions involving a metabolite [9]. Finding K-shortest paths is substituted here by finding K- lightest paths, that is, the sum of weights of arcs involved in the path is minimized.

Note here that there are path-finding strategies that use structural atomic mapping information. These approaches can be classified into two different groups. In the first group atomic mapping is used to build the metabolic graph, that is, an input metabolite is linked to an output metabolite in a given reaction if they share an atom mapping. In other words, an arc between a given pair of input/output metabolites exists if they have atoms in common in at least one reaction. The work of Faust *et al. *[35], based on the RPAIR database [38], is a reference example for these approaches. The effective carbon exchange strategy used in our CFP approach also falls into this group. However, it is slightly more restrictive than the approach presented in Faust *et al. *[35], since we exclusively focus on carbon atoms, that is, an arc between a given pair of input/output metabolites exists if they have carbon atoms in common in at least one reaction.

In the second group atomic mapping is used to guarantee that the pathway target metabolite involves at least one atom from the source metabolite. This concept was first introduced by Arita *et al. *[39], and recently revisited in Blum and Kohlbacher [40], and Heath *et al. *[41]. We are aware that this type of approach is, in theory, more restrictive than the effective carbon exchange strategy used in our CFP approach, since we guarantee effective carbon exchange between intermediates in the path, but not between the source and target metabolites. Tracing an atom from source to target metabolite, however, requires detailed knowledge of carbon atom mappings for each reaction. Though active research is being undertaken into this topic, more effort is still needed to release a fully curated and complete database for atomic mappings in genome-scale metabolic networks, especially for those from the Biochemical Genetic and Genomic (BiGG) database [42], which we are using here. For completeness, we will include results for the most recent approach [41], denoted as atom mapping-based strategy. Results were extracted from the web service (named AtomMetaNetWeb) available from Kavraki's lab [43].

**Figure 4:**
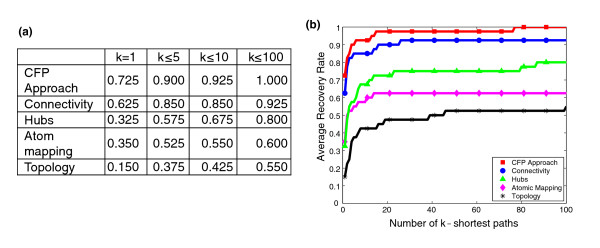
**Pathway recovery analysis**. **(a) **Average recovery rate among the k-shortest paths for k = 1, 5, 10, 100. **(b) **Average recovery rate among the k-shortest paths for k = 1,...,100 for different path-finding approaches.

Figure 4 shows results obtained for each of the strategies discussed above. It can be observed that the hubs-based strategy increases the average recovery rate with respect to the unadjusted metabolic graph (topology) by around 20% on average. The atom mapping-based strategy is clearly less accurate than the hubs-based strategy, which reflects the point discussed above that current databases for atomic mappings require further development. In addition, the connectivity-based strategy substantially outperforms the hubs-based strategy - for example, for k = 1, 62.5% and 32.5% of reference pathways are recovered, respectively. Finally, our CFP approach outperforms the connectivity-based strategy. This analysis shows, therefore, that our CFP approach (even without considering stoichiometry) outperforms existing path-finding methods.

Finally, note that other works [9,13] typically used the accuracy rate, instead of the recovery rate, for comparing the computed paths and reference pathways. We repeated the same analysis using this parameter. As observed in Additional file 2, a similar result to Figure 4 is obtained, which again shows that our CFP approach outperforms current methods.

#### Effect of stoichiometry

To illustrate the effect of stoichiometry, we first analyze a previously considered example from the literature, which emphasizes the fact that some paths (at the graph-theoretical level) cannot perform in steady-state and therefore are not biologically meaningful. We then repeat the side-by-side comparison presented in Figure 4 when stoichiometry is considered. To emphasize its importance, we examine how the connectivity structure of several metabolites is altered when stoichiometry is considered.

##### Stoichiometry and infeasible paths

**Figure 5:**
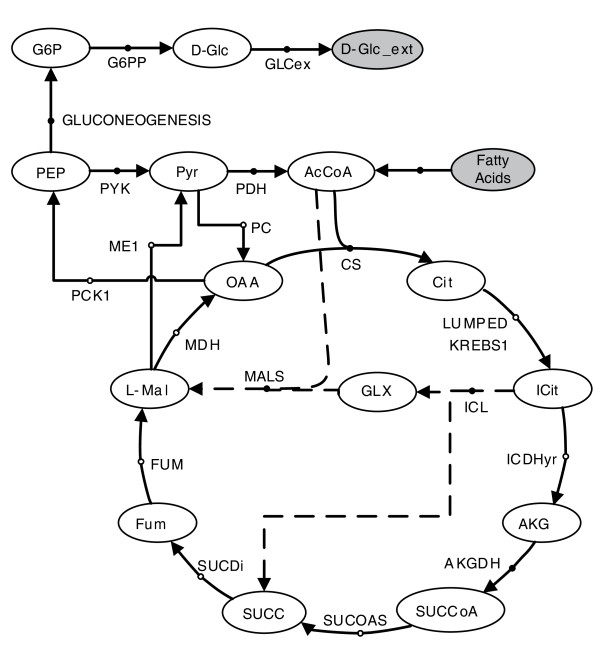
**Simplified network from that presented in de Figueiredo *et al. ***[25]**considering the question of conversion of fatty acids to sugars**. AcCoA, acetyl-CoA; AKG, 2-oxoglutarate; AKGDH, 2-oxogluterate dehydrogenase; Cit, citrate; CS, citrate synthase; D-Glc, glucose; D-Glc-ext, external glucose; Fum, fumarate; FUM, fumarase; G6P, glucose 6-phosphate; G6PP, glucose-6-phosphate phosphatase; GLCex, Glucose Exchange; Glx, Glyoxylate; ICDHyr, isocitrate dehydrogenase; ICit, isocitrate; ICL, Isocitrate lyase; L-Mal, Malate; MALS, malate synthase; MDH, malate dehydrogenase; ME1, malic enzyme (NAD); OAA, oxaloacetate; PC, Pyruvate carboxylase; PCK1, phosphoenolpyruvate carboxykinase; PDH, pyruvate dehydrogenase; PEP, phosphoenolpyruvate; PYK, pyruvate kinase; Pyr, pyruvate; SUCC, succinyl; SUCCoA, succinyl-coenzyme A; SUCDi, succinate dehydrogenase; SUCOAS, succinyl-CoA synthetase.

Figure 5 shows a simplified network from that presented in de Figueiredo *et al. *[25], which considered the question as to whether (or not) fatty acids can be converted into sugars. This question is answered by finding pathways from acetyl-CoA (AcCoA) to G6P. In that work, two scenarios were analyzed, namely pathway structure from AcCoA to G6P in the presence and absence of the enzymes of the glyoxylate shunt (indicated by dashed lines in Figure 5). In the metabolic network in Figure 5, when the glyoxylate shunt is absent, no possible pathway can exist in a stoichiometric balance from AcCoA to G6P. As observed in de Figueiredo *et al. *[25], this fact is not properly captured by path-finding methods, since stoichiometry is not taken into account. In contrast, our CFP approach correctly answers this question, by finding no paths between AcCoA and G6P when the glyoxylate shunt is not active. This is due to the addition of constraint 9, which forces paths to be able to work in sustained steady-state.

##### Side-by-side comparison with stoichiometry

We repeated the side-by-side comparison previously presented in Figure 4 for path-finding methods when stoichiometry is considered. Similarly, we used the 40 *E. coli *metabolic pathways discussed in Planes and Beasely [37], and the *E. coli *metabolic network in Feist *et al. *[36].

As we previously showed above (Figure 4) that our CFP approach (without considering stoichiometry, Equations 5 and 6) outperforms existing path-finding methods, we here compare the performance of our CFP approach with and without Equations 5 and 6 so as to evaluate the effect of stoichiometry. For this purpose, we analyzed our CFP approach in two different scenarios, namely when we used a minimal medium based on glucose as a sole carbon source under oxic and anoxic conditions, respectively. See Additional file 3 for details.

It is important to note that the use of a specific minimal medium (as we do here) prevents some known metabolic pathways from functioning in *E. coli *due to stoichiometric constraints. For example, the tricarboxylic acid (TCA) cycle cannot work in anoxic conditions in *E. coli*. The ability to detect these false positives cannot be accomplished without the use of stoichiometry. In light of this, the definition of recovery (as used in Figure 4) is slightly modified here. Recovery rate is 1 if (under a given growth medium) the model recovers a feasible pathway or the model excludes from the solution space an infeasible pathway, 0 otherwise. For illustration, if our CFP approach (incorrectly) detects the TCA cycle in anoxic conditions, recovery would be zero. However, if our CFP approach correctly excludes the TCA cycle from the solution space, then recovery would be 1.

**Figure 6:**
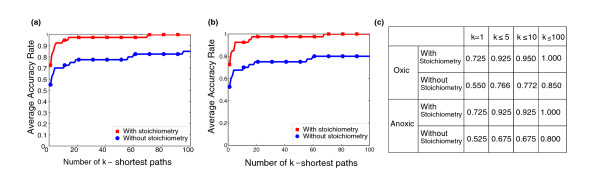
**Effect of stoichiometry in pathway recovery analysis**. Average recovery rate among the k-shortest paths for k = 1,...,100 for CFP approach with and without considering stoichometry in **(a) **oxic conditions; and **(b) **anoxic conditions; **(c) **Average recovery rate among the k-shortest paths for k = 1, 5, 10, 100 for the CFP approach in oxic and anoxic conditions.

Figure 6a shows how recovery rate evolves over k-shortest CFPs (k = 1,...,100) with/without stoichiometry in oxic conditions. We found that in these conditions, 6 out of 40 metabolic pathways cannot work in steady-state (Additional file 2). For example, the pathway for the degradation of 2,5-diketo-D-gluconate is not functionally feasible under these conditions since it cannot be synthesized from glucose in *E. coli *[44]. This logically cannot be captured without considering stoichiometry. This is reflected in Figure 6a, where average recovery rate among 100 shortest CFPs decreases to 0.85 without stoichiometry. The same analysis was repeated in anoxic conditions (Figure 6b), finding two additional pathways (TCA cycle and Allantoin degradation) not able to work in steady-state (given our growth medium). Figure 6c summarizes Figure 6a and Figure 6b for some particular values (k = 1, 5, 10 and 100). See Additional file 2 for further details, including results when average accuracy rate was used instead of recovery rate. This analysis shows the importance of stoichiometry and its underlying boundary definition at the functional level.

##### Connectivity analysis and stoichiometry

To emphasize the effect of stoichiometry, we examined the connectivity structure of oxaloacetate (OAA) in *E. coli*. OAA plays an important role in the regulation of carbon flux in most organisms. Again, for this study, we used the metabolic network presented in Feist *et al. *[36] and a minimal medium based on glucose as a sole carbon source and oxic conditions.

We determined CFPs from OAA to all reachable metabolites (obviously some metabolites may not be reachable via a CFP from OAA). In order to organize and compare the obtained results, we plotted a connectivity curve that shows the total number of connected metabolites when we move a specified number of reaction steps away from the source metabolite. To show the effect of stoichiometry, we plot the connectivity curves when stoichiometry is included (so including Equations 5 and 6) and when it is not included (so excluding Equations 5 and 6).

**Figure 7:**
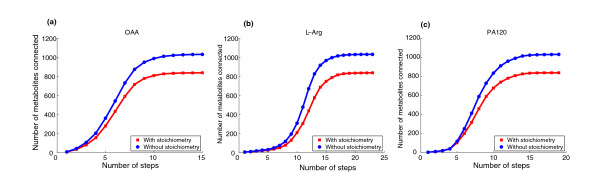
**Effect of stoichiometry in connectivity analysis**. **(a) **Connectivity curve for oxaloacetate (OAA). **(b) **Connectivity curve for arginine (L-Arg). **(c) **Connectivity curve for phosphatidic acid (PA120).

Figure 7a shows the connectivity curves for OAA. For example, in five reaction steps, OAA reaches 300 metabolites when stoichiometry is included and 400 metabolites otherwise. It can also be observed that, in any number of reaction steps, the number of metabolites reachable from OAA when stoichiometry is taken into account is 834, but 1,028 metabolites when it is not considered. These results clearly show the effect of considering stoichiometry. We repeated the same analysis in two structurally different metabolites, namely arginine (L-Arg), an amino acid, and phosphatidic acid (PA120), an important lipid. We found a very similar behavior, as observed in Figure 7b,c. This analysis shows the importance of considering stoichiometry for the topological analysis of metabolic networks from a path-based perspective.

### Application

It is usual to find K paths between a pair of key metabolites/reactions in, for example, the interpretation of 'omics' data [13,20]. Current path-finding methods do not take into account stoichiometric constraints for this analysis. In the analysis presented below we show that the resulting K functional paths are strongly dependent on stoichiometric constraints. This fact is illustrated in this sub-section with the pathway analysis of Pyr-OAA metabolism.

PEP, Pyr and OAA are important metabolites whose underlying inter-conversions control the carbon flux distribution in bacteria [45]. The performance of the PEP-Pyr-OAA node changes in different organisms and growth conditions. We focus here on the structure of CFPs from Pyr to OAA in *E. coli *in two different scenarios, namely in oxic and anoxic conditions. Pyr and OAA are linked by two fundamental metabolic processes. Firstly, Pyr (via PEP) can be carboxylated to OAA for the replenishment of TCA cycle intermediates or for anabolic purposes (for example, amino acid biosynthesis). This process is typically referred to as anaplerosis. In addition, Pyr and OAA are strongly related via the TCA cycle, which oxidizes carbon of Pyr to CO_2 _and requires OAA to operate.

We calculated the 100 shortest CFPs in both scenarios using the metabolic network presented in Feist *et al. *[36]. Again, we used the list of arcs presented in Additional file 1. In addition, we used a minimal medium based on glucose. See Additional file 3 for details as to the medium used.

**Figure 8:**
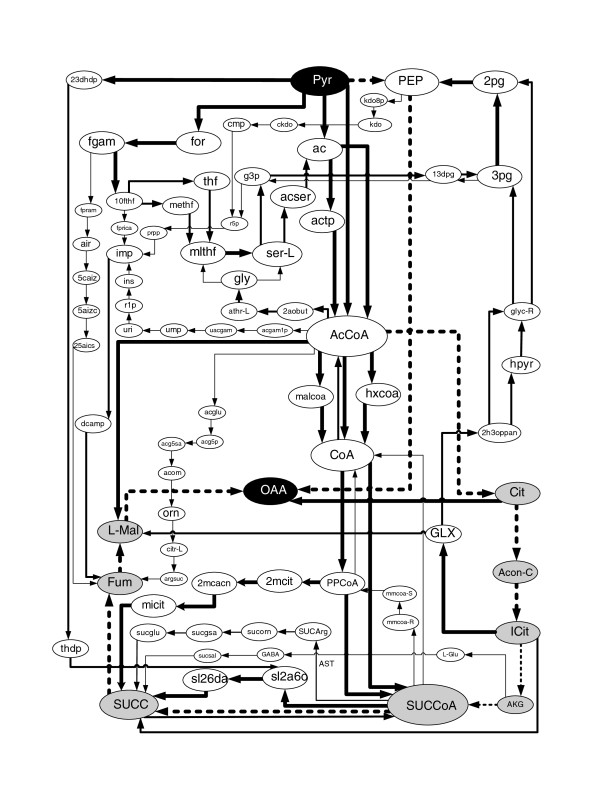
**100 shortest CFPs in *E. coli *from Pyr to OAA in oxic conditions**. Both the thickness of arcs and the size of metabolite nodes correspond to their frequency of appearance in the 100 shortest CFPs. Metabolites in grey are intermediates involved in the TCA cycle. See Additional file 3 for details.

Figure 8 shows the 100 shortest CFPs from Pyr to OAA in oxic conditions. Both fundamental metabolic processes described above between Pyr and OAA (anaplerotic route via PEP and the TCA cycle) are recovered (see dashed lines). In addition, different alternative routes to these processes are found. In particular, several bypasses to the TCA cycle can be observed in Figure 8. The glyoxylate (GLX) shunt was recovered, as well as the γ-aminobutyrate (GABA) shunt, whose role as an integral part of the TCA cycle was recently hypothesized [46]. We also determined a (theoretical, non-experimentally determined) bypass via propionyl-CoA (PPCoA), which was reported in a previous paper [31]. Interestingly, we also predicted a bypass to the TCA cycle via L-Arg catabolism. Though not shown in Figure 8, L-Arg is consumed in a reaction catalyzed by arginine succinyltransferase (AST; SUCCoA + L-Arg → SUCArg). Several links to the TCA cycle with arginine-L metabolism has been previously reported [47], although more research is needed to examine whether this detour is a functionally feasible alternative route to succinyl-CoA synthetase (SUCOAS) (ATP + CoA + SUCC ↔ ADP + Pi + SUCCoA).

Though the number of non-meaningful paths has been substantially reduced, it can be observed in Figure 8 that they still exist - for example, different routes via CoA. These false positives do not arise from the lack of stoichiometric balancing, but due to carbon exchange constraints. Indeed, these routes exchange carbon atoms in each of their intermediate steps but do not exchange carbon atoms between Pyr and OAA. When the current limitations described above (in the discussion of atom mapping-based approaches) are addressed, such strategies may be an effective constraint to remove these false positives.

**Figure 9:**
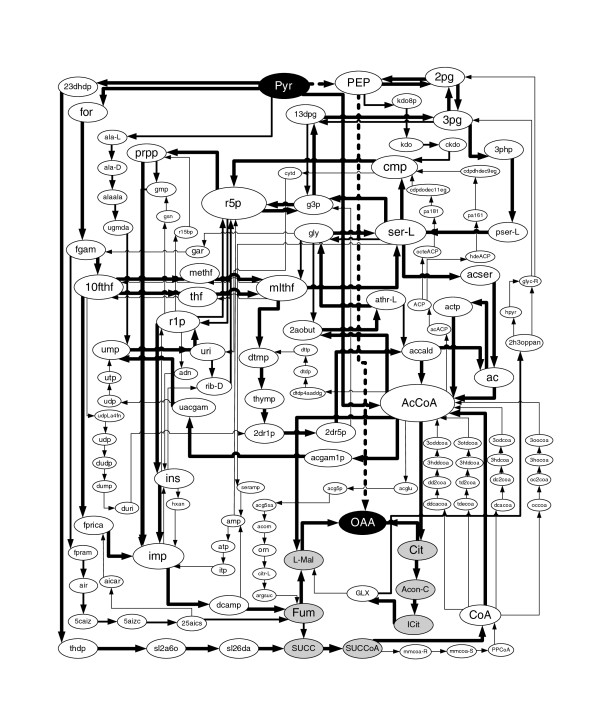
**100 shortest CFPs in *E. coli *from Pyr to OAA in anoxic conditions**. Both the thickness of arcs and the size of metabolite nodes correspond to their frequency of appearance in the 100 shortest CFPs. Metabolites in grey are intermediates involved in the TCA cycle. See Additional file 3 for details.

We repeated the same analysis in anoxic conditions (Figure 9). In this situation, the main variability in the 100 shortest CFPs is found in anaplerotic routes, since the TCA cycle is not active. This is due to the fact that the balancing of coenzyme Q (CoQ) and ubiquinol is not possible without oxygen and therefore enzyme succinate dehydrogenase (CoQ + SUCC → Fum + CoQH2) cannot work in sustained steady-state. This meant that several other reactions involved in the TCA cycle do not appear in the 100 shortest CFPs, namely isocitrate dehydrogenase (ICit + NADP ↔ AKG + CO_2 _+ NADPH), 2-oxogluterate dehydrogenase (AKG + CoA + NAD → CO_2 _+ NADH + SUCCoA) and SUCOAS (ATP + CoA + SUCC ↔ ADP + Pi + SUCCoA) are not in Figure 9. This is also the case for metabolite AKG, which is now not involved in the 100 shortest CFPs from Pyr to OAA, while in oxic conditions it appeared in five solutions. In addition, most of the bypasses previously mentioned in oxic conditions are not involved in Figure 9; indeed just the glyoxylate shunt is kept in the solution.

Finally, as observed in Figures 8 and 9, our CFP approach properly captures the metabolic changes induced when oxygen is removed from the medium. These changes cannot be captured if stoichiometric constraints are not considered, showing again the strength of our CFP approach.

## Conclusions

Graph-based methods have been widely used for the analysis of metabolic networks, but suffer from the important weakness that reaction stoichiometry is neglected. In this paper we show that, using the novel concept of CFPs, reaction stoichiometry can be incorporated into path-finding approaches, which constitute a clear progress over the state of the art at the methodological level.

Our results show that, when stoichiometry is incorporated into path-finding methods, the resulting set of functional pathways is substantially altered, as observed in the analysis of the 40 reference pathways. This idea is also reflected in the analysis of aerobic and anaerobic Pyr-OAA metabolism, which emphasizes the importance of the steady-state condition and its underlying boundary definition for the analysis of metabolic networks. In addition, connectivity analysis revealed important differences when stoichiometry was considered, as we illustrated with regard to a number of metabolites. In summary, CFPs open new avenues for analyzing metabolic networks at the topological and functional levels and constitute a major advance.

Though the incorporation of stoichiometry into a path-finding method is the main feature of our work, our CFP approach focuses on paths involving effective carbon exchange in each of their intermediate steps. The results we have presented confirm the relevance of this strategy when analyzing metabolic networks using a path-finding approach. Our public release of the manually curated *E. coli *database incorporating effective carbon exchange information (based on BiGG [42] and the work of Feist *et al. *[36]) represents a valuable dataset available for the scientific community, which can be used for further analysis.

It is important to mention that our CFP approach is formulated as a mixed-integer linear program, which cannot be solved using classical algorithms from graph theory and requires a branch and bound approach. Computational experience shows that the determination of CFPs is not expensive, namely in the order of milliseconds. This fact makes our approach an effective tool for addressing other relevant questions previously addressed by path-finding approaches.

Our analysis of CFPs in aerobic Pyr-OAA metabolism allowed us to detect several bypasses to the TCA cycle. Some of these bypasses have been recently reported using a different pathway analysis technique, namely elementary flux patterns for the bypass via the GABA shunt [48] and generating flux modes for the bypass via PPCoA [31]. In addition, we found an alternative bypass to the TCA cycle via L-Arg. This novel pathway is currently theoretical (it should be treated with caution) and requires experimental validation; however, it shows the capability of our CFP approach to generate new hypothesis.

Finally, despite much debate in the field comparing the performance of path-finding methods and stoichiometric methods [25,27,49], this article shows that both approaches can work in a synergic fashion so as to explore the huge complexity in cellular metabolism.

## Materials and methods

Equations 1 to 11 presented in the 'Mathematical model' sub-section define a mixed-integer linear problem and, algorithmically, such problems are solved by linear programming-based tree search. Modern software packages to perform this task, such as ILOG CPLEX, which we used, are well developed and highly sophisticated. ILOG CPLEX was run in a Matlab environment version 7.5 (R2007b).

The computation of the shortest CFP and the 100 shortest CFPs took us (on average) 300 ms and 2.5 minutes, respectively, on a 64-bit, 2.00 GHz PC with 12 Gb RAM. Analysis using regression indicated that, over the range of K values examined (up to K = 250), the total time for computing the K shortest CFPs was (approximately) proportional to K^1.4^. This implies that the computation time of CFPs grows only as a low power of the number of paths (K) sought.

## Abbreviations

AcCoA: acetyl-CoA; AcGlc-D: 6-acetyl-D-glucose; AKG: 2-oxoglutarate; AST: Arginine succinyltransferase; BiGG: Biochemical Genetic and Genomic; CDP: cytidine-diphosphate; CFP: carbon flux path; CoA: coenzyme A; CoQ: coenzyme Q; D-Glc: glucose; Fum: fumarate; G6P: glucose 6-phosphate; GABA: gamma-aminobutyric acid; GLCATr: D-glucose O-acetyltransferase; GLX: Glyoxylate; PA120: phosphatidic acid; HCO3: bicarbonate; ICit: isocitrate; L-Arg: arginine; L-Mal: acetyl-maltose; OAA: oxaloacetate; PEP: phosphoenolpyruvate; PPCoA: propionyl-CoA; Pyr: pyruvate; SUCArg: Succinyl-L-arginine; SUCC: Succinate; SUCCoA: succinyl-coenzyme A; SUCOAS: succinyl-CoA synthetase; TCA: tricarboxylic acid.

## Authors' contributions

JPe developed and implemented the method, wrote the manuscript and performed analyses; JPr developed the method and carbon exchange database; JEB developed the method and wrote the manuscript; FJP conceived the study, developed the method and wrote the manuscript. All authors discussed the results, and read, commented and approved the final manuscript.

## Supplementary Material

Additional file 1 **Database of carbon exchange arcs**. PDF document containing a list of arcs involving effective carbon flux in the metabolic network of Feist *et al. *[36]. Click here for file

Additional file 2 **Supporting data for side-by-side comparison**. Word document containing a list of 40 reference pathways used in the side-by-side comparison, a side-by-side comparison using accuracy rate, and a discussion on infeasible pathways in Figure 6 [7,9,12,13,35-37,41,44,50-56]. Click here for file

Additional file 3 **Supporting data for Figures 8 and 9**. Details of the 100 shortest CFPs in oxic and anoxic conditions from Pyr to OAA. Click here for file
