# Supplementary material for: Path finding methods accounting for stoichiometry in metabolic networks
Source: Genome Biol. 2011 May 27;12(5):R49. doi: 10.1186/gb-2011-12-5-r49 (PMC3219972; doi:10.1186/gb-2011-12-5-r49)
Supplement: Additional file 1 — Database of carbon exchange arcs. PDF document containing a list of arcs involving effective carbon flux in the metabolic network of Feist et al. [36]. [file gb-2011-12-5-r49-S1.PDF]

Supplementary Material II: List of carbon exchange arcs in the metabolic network of Feist et al, 2007

Input Metabolite		Output Metabolite		Reaction		
Index	Name	Index	Name	Index	Name	Carbon Exchange
1	12dgr120[p]	377	12dgr120	1	12DGR120tipp	1
2	12dgr140[p]	378	12dgr140	2	12DGR140tipp	1
3	12dgr141[p]	379	12dgr141	3	12DGR141tipp	1
4	12dgr160[p]	380	12dgr160	4	12DGR160tipp	1
5	12dgr161[p]	381	12dgr161	5	12DGR161tipp	1
6	12dgr180[p]	382	12dgr180	6	12DGR180tipp	1
7	12dgr181[p]	383	12dgr181	7	12DGR181tipp	1
8	12ppd-R[e]	9	12ppd-R[p]	8	12PPDRtex	1
9	12ppd-R[p]	1069	12ppd-R	9	12PPDRtpp	1
10	12ppd-S[e]	11	12ppd-S[p]	10	12PPDStex	1
11	12ppd-S[p]	1070	12ppd-S	11	12PPDStpp	1
12	14glucan[p]	98	h	12	14GLUCANabcpp	0
12	14glucan[p]	121	14glucan	12	14GLUCANabcpp	1
12	14glucan[p]	281	adp	12	14GLUCANabcpp	0
12	14glucan[p]	962	pi	12	14GLUCANabcpp	0
109	h2o	98	h	12	14GLUCANabcpp	0
109	h2o	121	14glucan	12	14GLUCANabcpp	0
109	h2o	281	adp	12	14GLUCANabcpp	0
109	h2o	962	pi	12	14GLUCANabcpp	0
135	atp	98	h	12	14GLUCANabcpp	0
135	atp	121	14glucan	12	14GLUCANabcpp	0
135	atp	281	adp	12	14GLUCANabcpp	1
135	atp	962	pi	12	14GLUCANabcpp	0
13	14glucan[e]	12	14glucan[p]	13	14GLUCANtexi	1
14	23camp[e]	22	23camp[p]	14	23CAMPtex	1
15	23ccmp[e]	21	23ccmp[p]	15	23CCMPtex	1
16	23cgmp[e]	23	23cgmp[p]	16	23CGMPtex	1
17	23cump[e]	20	23cump[p]	17	23CUMPtex	1
18	23dappa[p]	98	h	18	23DAPPAt2pp	0
18	23dappa[p]	388	23dappa	18	23DAPPAt2pp	1
437	h[p]	98	h	18	23DAPPAt2pp	1
437	h[p]	388	23dappa	18	23DAPPAt2pp	0
19	23dappa[e]	18	23dappa[p]	19	23DAPPAtex	1
20	23cump[p]	82	3ump[p]	20	23PDE2pp	1
20	23cump[p]	437	h[p]	20	23PDE2pp	0
684	h2o[p]	82	3ump[p]	20	23PDE2pp	0
684	h2o[p]	437	h[p]	20	23PDE2pp	0
21	23ccmp[p]	83	3cmp[p]	21	23PDE4pp	1
21	23ccmp[p]	437	h[p]	21	23PDE4pp	0
684	h2o[p]	83	3cmp[p]	21	23PDE4pp	0
684	h2o[p]	437	h[p]	21	23PDE4pp	0
22	23camp[p]	84	3amp[p]	22	23PDE7pp	1
22	23camp[p]	437	h[p]	22	23PDE7pp	0
684	h2o[p]	84	3amp[p]	22	23PDE7pp	0
684	h2o[p]	437	h[p]	22	23PDE7pp	0
23	23cgmp[p]	85	3gmp[p]	23	23PDE9pp	1
23	23cgmp[p]	437	h[p]	23	23PDE9pp	0
684	h2o[p]	85	3gmp[p]	23	23PDE9pp	0
684	h2o[p]	437	h[p]	23	23PDE9pp	0

24	26dap-M[e]	387	26dap-M[p]	24	26DAHtex	1
25	2ddecg3p[p]	778	2ddecg3p	25	2AGPA120tipp	1
26	2tdecg3p[p]	779	2tdecg3p	26	2AGPA140tipp	1
27	2tdec7eg3p[p]	780	2tdec7eg3p	27	2AGPA141tipp	1
28	2hdecg3p[p]	781	2hdecg3p	28	2AGPA160tipp	1
29	2hdec9eg3p[p]	782	2hdec9eg3p	29	2AGPA161tipp	1
30	2odecg3p[p]	783	2odecg3p	30	2AGPA180tipp	1
31	2odec11eg3p[p]	784	2odec11eg3p	31	2AGPA181tipp	1
32	2agpe120[p]	39	2agpe120	32	2AGPE120tipp	1
33	2agpe140[p]	40	2agpe140	33	2AGPE140tipp	1
34	2agpe141[p]	41	2agpe141	34	2AGPE141tipp	1
35	2agpe160[p]	42	2agpe160	35	2AGPE160tipp	1
36	2agpe161[p]	43	2agpe161	36	2AGPE161tipp	1
37	2agpe180[p]	44	2agpe180	37	2AGPE180tipp	1
38	2agpe181[p]	45	2agpe181	38	2AGPE181tipp	1
39	2agpe120	177	amp	39	2AGPEAT120	0
39	2agpe120	1192	ppi	39	2AGPEAT120	0
39	2agpe120	1565	pe120	39	2AGPEAT120	1
135	atp	177	amp	39	2AGPEAT120	1
135	atp	1192	ppi	39	2AGPEAT120	0
135	atp	1565	pe120	39	2AGPEAT120	0
1303	ddca	177	amp	39	2AGPEAT120	0
1303	ddca	1192	ppi	39	2AGPEAT120	0
1303	ddca	1565	pe120	39	2AGPEAT120	1
40	2agpe140	177	amp	40	2AGPEAT140	0
40	2agpe140	1192	ppi	40	2AGPEAT140	0
40	2agpe140	1566	pe140	40	2AGPEAT140	1
135	atp	177	amp	40	2AGPEAT140	1
135	atp	1192	ppi	40	2AGPEAT140	0
135	atp	1566	pe140	40	2AGPEAT140	0
1616	ttdca	177	amp	40	2AGPEAT140	0
1616	ttdca	1192	ppi	40	2AGPEAT140	0
1616	ttdca	1566	pe140	40	2AGPEAT140	1
41	2agpe141	177	amp	41	2AGPEAT141	0
41	2agpe141	1192	ppi	41	2AGPEAT141	0
41	2agpe141	1567	pe141	41	2AGPEAT141	1
135	atp	177	amp	41	2AGPEAT141	1
135	atp	1192	ppi	41	2AGPEAT141	0
135	atp	1567	pe141	41	2AGPEAT141	0
1617	ttdcea	177	amp	41	2AGPEAT141	0
1617	ttdcea	1192	ppi	41	2AGPEAT141	0
1617	ttdcea	1567	pe141	41	2AGPEAT141	1
42	2agpe160	177	amp	42	2AGPEAT160	0
42	2agpe160	1192	ppi	42	2AGPEAT160	0
42	2agpe160	1568	pe160	42	2AGPEAT160	1
135	atp	177	amp	42	2AGPEAT160	1
135	atp	1192	ppi	42	2AGPEAT160	0
135	atp	1568	pe160	42	2AGPEAT160	0
1618	hdca	177	amp	42	2AGPEAT160	0
1618	hdca	1192	ppi	42	2AGPEAT160	0
1618	hdca	1568	pe160	42	2AGPEAT160	1
43	2agpe161	177	amp	43	2AGPEAT161	0
43	2agpe161	1192	ppi	43	2AGPEAT161	0
43	2agpe161	1387	pe161	43	2AGPEAT161	1
135	atp	177	amp	43	2AGPEAT161	1
135	atp	1192	ppi	43	2AGPEAT161	0

135	atp	1387	pe161	43	2AGPEAT161	0
1619	hdcea	177	amp	43	2AGPEAT161	0
1619	hdcea	1192	ppi	43	2AGPEAT161	0
1619	hdcea	1387	pe161	43	2AGPEAT161	1
44	2agpe180	177	amp	44	2AGPEAT180	0
44	2agpe180	1192	ppi	44	2AGPEAT180	0
44	2agpe180	1569	pe180	44	2AGPEAT180	1
135	atp	177	amp	44	2AGPEAT180	1
135	atp	1192	ppi	44	2AGPEAT180	0
135	atp	1569	pe180	44	2AGPEAT180	0
1620	ocdca	177	amp	44	2AGPEAT180	0
1620	ocdca	1192	ppi	44	2AGPEAT180	0
1620	ocdca	1569	pe180	44	2AGPEAT180	1
45	2agpe181	177	amp	45	2AGPEAT181	0
45	2agpe181	1192	ppi	45	2AGPEAT181	0
45	2agpe181	1388	pe181	45	2AGPEAT181	1
135	atp	177	amp	45	2AGPEAT181	1
135	atp	1192	ppi	45	2AGPEAT181	0
135	atp	1388	pe181	45	2AGPEAT181	0
1621	ocdcea	177	amp	45	2AGPEAT181	0
1621	ocdcea	1192	ppi	45	2AGPEAT181	0
1621	ocdcea	1388	pe181	45	2AGPEAT181	1
46	2agpg120[p]	53	2agpg120	46	2AGPG120tipp	1
47	2agpg140[p]	54	2agpg140	47	2AGPG140tipp	1
48	2agpg141[p]	55	2agpg141	48	2AGPG141tipp	1
49	2agpg160[p]	56	2agpg160	49	2AGPG160tipp	1
50	2agpg161[p]	57	2agpg161	50	2AGPG161tipp	1
51	2agpg180[p]	58	2agpg180	51	2AGPG180tipp	1
52	2agpg181[p]	59	2agpg181	52	2AGPG181tipp	1
53	2agpg120	177	amp	53	2AGPGAT120	0
53	2agpg120	1192	ppi	53	2AGPGAT120	0
53	2agpg120	1336	pg120	53	2AGPGAT120	1
135	atp	177	amp	53	2AGPGAT120	1
135	atp	1192	ppi	53	2AGPGAT120	0
135	atp	1336	pg120	53	2AGPGAT120	0
1303	ddca	177	amp	53	2AGPGAT120	0
1303	ddca	1192	ppi	53	2AGPGAT120	0
1303	ddca	1336	pg120	53	2AGPGAT120	1
54	2agpg140	177	amp	54	2AGPGAT140	0
54	2agpg140	1192	ppi	54	2AGPGAT140	0
54	2agpg140	1337	pg140	54	2AGPGAT140	1
135	atp	177	amp	54	2AGPGAT140	1
135	atp	1192	ppi	54	2AGPGAT140	0
135	atp	1337	pg140	54	2AGPGAT140	0
1616	ttdca	177	amp	54	2AGPGAT140	0
1616	ttdca	1192	ppi	54	2AGPGAT140	0
1616	ttdca	1337	pg140	54	2AGPGAT140	1
55	2agpg141	177	amp	55	2AGPGAT141	0
55	2agpg141	1192	ppi	55	2AGPGAT141	0
55	2agpg141	1338	pg141	55	2AGPGAT141	1
135	atp	177	amp	55	2AGPGAT141	1
135	atp	1192	ppi	55	2AGPGAT141	0
135	atp	1338	pg141	55	2AGPGAT141	0
1617	ttdcea	177	amp	55	2AGPGAT141	0
1617	ttdcea	1192	ppi	55	2AGPGAT141	0
1617	ttdcea	1338	pg141	55	2AGPGAT141	1

56	2agpg160	177	amp	56	2AGPGAT160	0
56	2agpg160	1192	ppi	56	2AGPGAT160	0
56	2agpg160	1339	pg160	56	2AGPGAT160	1
135	atp	177	amp	56	2AGPGAT160	1
135	atp	1192	ppi	56	2AGPGAT160	0
135	atp	1339	pg160	56	2AGPGAT160	0
1618	hdca	177	amp	56	2AGPGAT160	0
1618	hdca	1192	ppi	56	2AGPGAT160	0
1618	hdca	1339	pg160	56	2AGPGAT160	1
57	2agpg161	177	amp	57	2AGPGAT161	0
57	2agpg161	1192	ppi	57	2AGPGAT161	0
57	2agpg161	1340	pg161	57	2AGPGAT161	1
135	atp	177	amp	57	2AGPGAT161	1
135	atp	1192	ppi	57	2AGPGAT161	0
135	atp	1340	pg161	57	2AGPGAT161	0
1619	hdcea	177	amp	57	2AGPGAT161	0
1619	hdcea	1192	ppi	57	2AGPGAT161	0
1619	hdcea	1340	pg161	57	2AGPGAT161	1
58	2agpg180	177	amp	58	2AGPGAT180	0
58	2agpg180	1192	ppi	58	2AGPGAT180	0
58	2agpg180	1341	pg180	58	2AGPGAT180	1
135	atp	177	amp	58	2AGPGAT180	1
135	atp	1192	ppi	58	2AGPGAT180	0
135	atp	1341	pg180	58	2AGPGAT180	0
1620	ocdca	177	amp	58	2AGPGAT180	0
1620	ocdca	1192	ppi	58	2AGPGAT180	0
1620	ocdca	1341	pg180	58	2AGPGAT180	1
59	2agpg181	177	amp	59	2AGPGAT181	0
59	2agpg181	1192	ppi	59	2AGPGAT181	0
59	2agpg181	1342	pg181	59	2AGPGAT181	1
135	atp	177	amp	59	2AGPGAT181	1
135	atp	1192	ppi	59	2AGPGAT181	0
135	atp	1342	pg181	59	2AGPGAT181	0
1621	ocdcea	177	amp	59	2AGPGAT181	0
1621	ocdcea	1192	ppi	59	2AGPGAT181	0
1621	ocdcea	1342	pg181	59	2AGPGAT181	1
60	2dhglcn	856	nad	60	2DGLCNRx	0
60	2dhglcn	1073	glcn	60	2DGLCNRx	1
98	h	856	nad	60	2DGLCNRx	0
98	h	1073	glcn	60	2DGLCNRx	0
870	nadh	856	nad	60	2DGLCNRx	1
870	nadh	1073	glcn	60	2DGLCNRx	0
60	2dhglcn	459	nadp	61	2DGLCNRy	0
60	2dhglcn	1073	glcn	61	2DGLCNRy	1
98	h	459	nadp	61	2DGLCNRy	0
98	h	1073	glcn	61	2DGLCNRy	0
871	nadph	459	nadp	61	2DGLCNRy	1
871	nadph	1073	glcn	61	2DGLCNRy	0
61	2dhguln	856	nad	62	2DGULRx	0
61	2dhguln	1129	idon-L	62	2DGULRx	1
98	h	856	nad	62	2DGULRx	0
98	h	1129	idon-L	62	2DGULRx	0
870	nadh	856	nad	62	2DGULRx	1
870	nadh	1129	idon-L	62	2DGULRx	0
61	2dhguln	459	nadp	63	2DGULRy	0
61	2dhguln	1129	idon-L	63	2DGULRy	1

98	h	459	nadp	63	2DGULRy	0
98	h	1129	idon-L	63	2DGULRy	0
871	nadph	459	nadp	63	2DGULRy	1
871	nadph	1129	idon-L	63	2DGULRy	0
62	2mahmp	98	h	64	2MAHMP	0
62	2mahmp	940	4ampm	64	2MAHMP	1
62	2mahmp	962	pi	64	2MAHMP	0
109	h2o	98	h	64	2MAHMP	0
109	h2o	940	4ampm	64	2MAHMP	0
109	h2o	962	pi	64	2MAHMP	0
63	34dhpac[e]	1071	34dhpac[p]	65	34dhpactex	1
64	3amp[e]	84	3amp[p]	66	3AMPtex	1
65	3cmp[e]	83	3cmp[p]	67	3CMPtex	1
66	3gmp[e]	85	3gmp[p]	68	3GMPtex	1
67	3hdecACP	109	h2o	69	3HAD100	0
67	3hdecACP	1006	tdec2eACP	69	3HAD100	1
68	3hddecACP	109	h2o	70	3HAD120	0
68	3hddecACP	1570	tddec2eACP	70	3HAD120	1
69	3hcddec5eACP	109	h2o	71	3HAD121	0
69	3hcddec5eACP	1571	t3c5ddeceACP	71	3HAD121	1
70	3hmrsACP	109	h2o	72	3HAD140	0
70	3hmrsACP	1572	tmrs2eACP	72	3HAD140	1
71	3hcmrs7eACP	109	h2o	73	3HAD141	0
71	3hcmrs7eACP	1573	t3c7mrseACP	73	3HAD141	1
72	3hpalmACP	109	h2o	74	3HAD160	0
72	3hpalmACP	1574	tpalm2eACP	74	3HAD160	1
73	3hcpalm9eACP	109	h2o	75	3HAD161	0
73	3hcpalm9eACP	1575	t3c9palmeACP	75	3HAD161	1
74	3hoctaACP	109	h2o	76	3HAD180	0
74	3hoctaACP	1576	toctd2eACP	76	3HAD180	1
75	3hcvac11eACP	109	h2o	77	3HAD181	0
75	3hcvac11eACP	1577	t3c11vaceACP	77	3HAD181	1
76	3haACP	109	h2o	78	3HAD40	0
76	3haACP	469	but2eACP	78	3HAD40	1
77	3hhexACP	109	h2o	79	3HAD60	0
77	3hhexACP	1578	thex2eACP	79	3HAD60	1
78	3hoctACP	109	h2o	80	3HAD80	0
78	3hoctACP	1579	toct2eACP	80	3HAD80	1
79	3hcinnm	109	h2o	81	3HCINNMH	0
79	3hcinnm	419	dhcinnm	81	3HCINNMH	1
79	3hcinnm	856	nad	81	3HCINNMH	0
98	h	109	h2o	81	3HCINNMH	0
98	h	419	dhcinnm	81	3HCINNMH	0
98	h	856	nad	81	3HCINNMH	0
870	nadh	109	h2o	81	3HCINNMH	0
870	nadh	419	dhcinnm	81	3HCINNMH	0
870	nadh	856	nad	81	3HCINNMH	1
928	o2	109	h2o	81	3HCINNMH	0
928	o2	419	dhcinnm	81	3HCINNMH	0
928	o2	856	nad	81	3HCINNMH	0
80	3hpppn	109	h2o	82	3HPPPNH	0
80	3hpppn	706	dhpppn	82	3HPPPNH	1
80	3hpppn	856	nad	82	3HPPPNH	0
98	h	109	h2o	82	3HPPPNH	0
98	h	706	dhpppn	82	3HPPPNH	0
98	h	856	nad	82	3HPPPNH	0

870	nadh	109	h2o	82	3HPPPNH	0
870	nadh	706	dhpppn	82	3HPPPNH	0
870	nadh	856	nad	82	3HPPPNH	1
928	o2	109	h2o	82	3HPPPNH	0
928	o2	706	dhpppn	82	3HPPPNH	0
928	o2	856	nad	82	3HPPPNH	0
81	3dhguln	98	h	83	3KGK	0
81	3dhguln	281	adp	83	3KGK	0
81	3dhguln	743	3dhgulnp	83	3KGK	1
135	atp	98	h	83	3KGK	0
135	atp	281	adp	83	3KGK	1
135	atp	743	3dhgulnp	83	3KGK	0
82	3ump[p]	1173	pi[p]	84	3NTD2pp	0
82	3ump[p]	1229	uri[p]	84	3NTD2pp	1
684	h2o[p]	1173	pi[p]	84	3NTD2pp	0
684	h2o[p]	1229	uri[p]	84	3NTD2pp	0
83	3cmp[p]	372	cytd[p]	85	3NTD4pp	1
83	3cmp[p]	1173	pi[p]	85	3NTD4pp	0
684	h2o[p]	372	cytd[p]	85	3NTD4pp	0
684	h2o[p]	1173	pi[p]	85	3NTD4pp	0
84	3amp[p]	179	adn[p]	86	3NTD7pp	1
84	3amp[p]	1173	pi[p]	86	3NTD7pp	0
684	h2o[p]	179	adn[p]	86	3NTD7pp	0
684	h2o[p]	1173	pi[p]	86	3NTD7pp	0
85	3gmp[p]	666	gsn[p]	87	3NTD9pp	1
85	3gmp[p]	1173	pi[p]	87	3NTD9pp	0
684	h2o[p]	666	gsn[p]	87	3NTD9pp	0
684	h2o[p]	1173	pi[p]	87	3NTD9pp	0
86	3odecACP	67	3hdecACP	88	3OAR100	1
86	3odecACP	459	nadp	88	3OAR100	0
98	h	67	3hdecACP	88	3OAR100	0
98	h	459	nadp	88	3OAR100	0
871	nadph	67	3hdecACP	88	3OAR100	0
871	nadph	459	nadp	88	3OAR100	1
87	3oddecACP	68	3hddecACP	89	3OAR120	1
87	3oddecACP	459	nadp	89	3OAR120	0
98	h	68	3hddecACP	89	3OAR120	0
98	h	459	nadp	89	3OAR120	0
871	nadph	68	3hddecACP	89	3OAR120	0
871	nadph	459	nadp	89	3OAR120	1
88	3ocddec5eACP	69	3hcddec5eACP	90	3OAR121	1
88	3ocddec5eACP	459	nadp	90	3OAR121	0
98	h	69	3hcddec5eACP	90	3OAR121	0
98	h	459	nadp	90	3OAR121	0
871	nadph	69	3hcddec5eACP	90	3OAR121	0
871	nadph	459	nadp	90	3OAR121	1
89	3omrsACP	70	3hmrsACP	91	3OAR140	1
89	3omrsACP	459	nadp	91	3OAR140	0
98	h	70	3hmrsACP	91	3OAR140	0
98	h	459	nadp	91	3OAR140	0
871	nadph	70	3hmrsACP	91	3OAR140	0
871	nadph	459	nadp	91	3OAR140	1
90	3ocmrs7eACP	71	3hcmrs7eACP	92	3OAR141	1
90	3ocmrs7eACP	459	nadp	92	3OAR141	0
98	h	71	3hcmrs7eACP	92	3OAR141	0
98	h	459	nadp	92	3OAR141	0

871	nadph	71	3hcmrs7eACP	92	3OAR141	0
871	nadph	459	nadp	92	3OAR141	1
91	3opalmACP	72	3hpalmACP	93	3OAR160	1
91	3opalmACP	459	nadp	93	3OAR160	0
98	h	72	3hpalmACP	93	3OAR160	0
98	h	459	nadp	93	3OAR160	0
871	nadph	72	3hpalmACP	93	3OAR160	0
871	nadph	459	nadp	93	3OAR160	1
92	3ocpalm9eACP	73	3hcpalm9eACP	94	3OAR161	1
92	3ocpalm9eACP	459	nadp	94	3OAR161	0
98	h	73	3hcpalm9eACP	94	3OAR161	0
98	h	459	nadp	94	3OAR161	0
871	nadph	73	3hcpalm9eACP	94	3OAR161	0
871	nadph	459	nadp	94	3OAR161	1
93	3ooctdACP	74	3hoctaACP	95	3OAR180	1
93	3ooctdACP	459	nadp	95	3OAR180	0
98	h	74	3hoctaACP	95	3OAR180	0
98	h	459	nadp	95	3OAR180	0
871	nadph	74	3hoctaACP	95	3OAR180	0
871	nadph	459	nadp	95	3OAR180	1
94	3ocvac11eACP	75	3hcvac11eACP	96	3OAR181	1
94	3ocvac11eACP	459	nadp	96	3OAR181	0
98	h	75	3hcvac11eACP	96	3OAR181	0
98	h	459	nadp	96	3OAR181	0
871	nadph	75	3hcvac11eACP	96	3OAR181	0
871	nadph	459	nadp	96	3OAR181	1
95	actACP	76	3haACP	97	3OAR40	1
95	actACP	459	nadp	97	3OAR40	0
98	h	76	3haACP	97	3OAR40	0
98	h	459	nadp	97	3OAR40	0
871	nadph	76	3haACP	97	3OAR40	0
871	nadph	459	nadp	97	3OAR40	1
96	3ohexACP	77	3hhexACP	98	3OAR60	1
96	3ohexACP	459	nadp	98	3OAR60	0
98	h	77	3hhexACP	98	3OAR60	0
98	h	459	nadp	98	3OAR60	0
871	nadph	77	3hhexACP	98	3OAR60	0
871	nadph	459	nadp	98	3OAR60	1
97	3ooctACP	78	3hoctACP	99	3OAR80	1
97	3ooctACP	459	nadp	99	3OAR80	0
98	h	78	3hoctACP	99	3OAR80	0
98	h	459	nadp	99	3OAR80	0
871	nadph	78	3hoctACP	99	3OAR80	0
871	nadph	459	nadp	99	3OAR80	1
98	h	86	3odecACP	100	3OAS100	0
98	h	118	ACP	100	3OAS100	0
98	h	692	co2	100	3OAS100	0
1363	malACP	86	3odecACP	100	3OAS100	1
1363	malACP	118	ACP	100	3OAS100	1
1363	malACP	692	co2	100	3OAS100	0
1395	ocACP	86	3odecACP	100	3OAS100	1
1395	ocACP	118	ACP	100	3OAS100	1
1395	ocACP	692	co2	100	3OAS100	0
98	h	87	3oddecACP	101	3OAS120	0
98	h	118	ACP	101	3OAS120	0
98	h	692	co2	101	3OAS120	0

99	dcaACP	87	3oddecACP	101	3OAS120	1
99	dcaACP	118	ACP	101	3OAS120	1
99	dcaACP	692	co2	101	3OAS120	0
1363	malACP	87	3oddecACP	101	3OAS120	1
1363	malACP	118	ACP	101	3OAS120	1
1363	malACP	692	co2	101	3OAS120	0
98	h	88	3ocddec5eACP	102	3OAS121	0
98	h	118	ACP	102	3OAS121	0
98	h	692	co2	102	3OAS121	0
100	cdec3eACP	88	3ocddec5eACP	102	3OAS121	1
100	cdec3eACP	118	ACP	102	3OAS121	1
100	cdec3eACP	692	co2	102	3OAS121	0
1363	malACP	88	3ocddec5eACP	102	3OAS121	1
1363	malACP	118	ACP	102	3OAS121	1
1363	malACP	692	co2	102	3OAS121	0
98	h	89	3omrsACP	103	3OAS140	0
98	h	118	ACP	103	3OAS140	0
98	h	692	co2	103	3OAS140	0
101	ddcaACP	89	3omrsACP	103	3OAS140	1
101	ddcaACP	118	ACP	103	3OAS140	1
101	ddcaACP	692	co2	103	3OAS140	0
1363	malACP	89	3omrsACP	103	3OAS140	1
1363	malACP	118	ACP	103	3OAS140	1
1363	malACP	692	co2	103	3OAS140	0
98	h	90	3ocmrs7eACP	104	3OAS141	0
98	h	118	ACP	104	3OAS141	0
98	h	692	co2	104	3OAS141	0
102	cddec5eACP	90	3ocmrs7eACP	104	3OAS141	1
102	cddec5eACP	118	ACP	104	3OAS141	1
102	cddec5eACP	692	co2	104	3OAS141	0
1363	malACP	90	3ocmrs7eACP	104	3OAS141	1
1363	malACP	118	ACP	104	3OAS141	1
1363	malACP	692	co2	104	3OAS141	0
98	h	91	3opalmACP	105	3OAS160	0
98	h	118	ACP	105	3OAS160	0
98	h	692	co2	105	3OAS160	0
1267	myrsACP	91	3opalmACP	105	3OAS160	1
1267	myrsACP	118	ACP	105	3OAS160	1
1267	myrsACP	692	co2	105	3OAS160	0
1363	malACP	91	3opalmACP	105	3OAS160	1
1363	malACP	118	ACP	105	3OAS160	1
1363	malACP	692	co2	105	3OAS160	0
98	h	92	3ocpalm9eACP	106	3OAS161	0
98	h	118	ACP	106	3OAS161	0
98	h	692	co2	106	3OAS161	0
1363	malACP	92	3ocpalm9eACP	106	3OAS161	1
1363	malACP	118	ACP	106	3OAS161	1
1363	malACP	692	co2	106	3OAS161	0
1373	tdeACP	92	3ocpalm9eACP	106	3OAS161	1
1373	tdeACP	118	ACP	106	3OAS161	1
1373	tdeACP	692	co2	106	3OAS161	0
98	h	93	3ooctdACP	107	3OAS180	0
98	h	118	ACP	107	3OAS180	0
98	h	692	co2	107	3OAS180	0
1363	malACP	93	3ooctdACP	107	3OAS180	1
1363	malACP	118	ACP	107	3OAS180	1

1363	malACP	692	co2	107	3OAS180	0
1374	palmACP	93	3ooctdACP	107	3OAS180	1
1374	palmACP	118	ACP	107	3OAS180	1
1374	palmACP	692	co2	107	3OAS180	0
98	h	94	3ocvac11eACP	108	3OAS181	0
98	h	118	ACP	108	3OAS181	0
98	h	692	co2	108	3OAS181	0
486	hdeACP	94	3ocvac11eACP	108	3OAS181	1
486	hdeACP	118	ACP	108	3OAS181	1
486	hdeACP	692	co2	108	3OAS181	0
1363	malACP	94	3ocvac11eACP	108	3OAS181	1
1363	malACP	118	ACP	108	3OAS181	1
1363	malACP	692	co2	108	3OAS181	0
98	h	96	3ohexACP	109	3OAS60	0
98	h	118	ACP	109	3OAS60	0
98	h	692	co2	109	3OAS60	0
103	butACP	96	3ohexACP	109	3OAS60	1
103	butACP	118	ACP	109	3OAS60	1
103	butACP	692	co2	109	3OAS60	0
1363	malACP	96	3ohexACP	109	3OAS60	1
1363	malACP	118	ACP	109	3OAS60	1
1363	malACP	692	co2	109	3OAS60	0
98	h	97	3ooctACP	110	3OAS80	0
98	h	118	ACP	110	3OAS80	0
98	h	692	co2	110	3OAS80	0
1268	hexACP	97	3ooctACP	110	3OAS80	1
1268	hexACP	118	ACP	110	3OAS80	1
1268	hexACP	692	co2	110	3OAS80	0
1363	malACP	97	3ooctACP	110	3OAS80	1
1363	malACP	118	ACP	110	3OAS80	1
1363	malACP	692	co2	110	3OAS80	0
104	.alaDgluMdap[p	98	h	111	3PEPTabcpp	0
104	.alaDgluMdap[p	281	adp	111	3PEPTabcpp	0
104	.alaDgluMdap[p	745	LalaDgluMdap	111	3PEPTabcpp	1
104	.alaDgluMdap[p	962	pi	111	3PEPTabcpp	0
109	h2o	98	h	111	3PEPTabcpp	0
109	h2o	281	adp	111	3PEPTabcpp	0
109	h2o	745	LalaDgluMdap	111	3PEPTabcpp	0
109	h2o	962	pi	111	3PEPTabcpp	0
135	atp	98	h	111	3PEPTabcpp	0
135	atp	281	adp	111	3PEPTabcpp	1
135	atp	745	LalaDgluMdap	111	3PEPTabcpp	0
135	atp	962	pi	111	3PEPTabcpp	0
105	.alaDgluMdap[e	104	LalaDgluMdap[p]	112	3PEPTtex	1
106	3ump[e]	82	3ump[p]	113	3UMPtex	1
107	dopa[p]	859	nh4[p]	114	42A12BOOXpp	0
107	dopa[p]	1071	34dhpac[p]	114	42A12BOOXpp	1
107	dopa[p]	1122	h2o2[p]	114	42A12BOOXpp	0
684	h2o[p]	859	nh4[p]	114	42A12BOOXpp	0
684	h2o[p]	1071	34dhpac[p]	114	42A12BOOXpp	0
684	h2o[p]	1122	h2o2[p]	114	42A12BOOXpp	0
903	o2[p]	859	nh4[p]	114	42A12BOOXpp	0
903	o2[p]	1071	34dhpac[p]	114	42A12BOOXpp	0
903	o2[p]	1122	h2o2[p]	114	42A12BOOXpp	0
108	4hoxpacd[e]	1072	4hoxpacd[p]	115	4HOXPACDtex	1
109	h2o	962	pi	116	4HTHRS	0

109	h2o	1238	4hthr	116	4HTHRS	0
1364	phthr	962	pi	116	4HTHRS	0
1364	phthr	1238	4hthr	116	4HTHRS	1
109	h2o	219	ala-D	117	4PCP	0
109	h2o	745	LalaDgluMdap	117	4PCP	0
110	ılaDgluMdapDa	219	ala-D	117	4PCP	1
110	ılaDgluMdapDa	745	LalaDgluMdap	117	4PCP	1
111	aDgluMdapDala	104	LalaDgluMdap[p]	118	4PCPpp	1
111	aDgluMdapDala	384	ala-D[p]	118	4PCPpp	1
684	h2o[p]	104	LalaDgluMdap[p]	118	4PCPpp	0
684	h2o[p]	384	ala-D[p]	118	4PCPpp	0
109	h2o	98	h	119	4PEPTabcpp	0
109	h2o	110	LalaDgluMdapDala	119	4PEPTabcpp	0
109	h2o	281	adp	119	4PEPTabcpp	0
109	h2o	962	pi	119	4PEPTabcpp	0
111	aDgluMdapDala	98	h	119	4PEPTabcpp	0
111	aDgluMdapDala	110	LalaDgluMdapDala	119	4PEPTabcpp	1
111	aDgluMdapDala	281	adp	119	4PEPTabcpp	0
111	aDgluMdapDala	962	pi	119	4PEPTabcpp	0
135	atp	98	h	119	4PEPTabcpp	0
135	atp	110	LalaDgluMdapDala	119	4PEPTabcpp	0
135	atp	281	adp	119	4PEPTabcpp	1
135	atp	962	pi	119	4PEPTabcpp	0
112	aDgluMdapDala	111	LalaDgluMdapDala[p]	120	4PEPTtex	1
98	h	459	nadp	121	5DGLCNR	0
98	h	1073	glcn	121	5DGLCNR	0
113	5dglcn	459	nadp	121	5DGLCNR	0
113	5dglcn	1073	glcn	121	5DGLCNR	1
871	nadph	459	nadp	121	5DGLCNR	1
871	nadph	1073	glcn	121	5DGLCNR	0
114	5dglcn[p]	98	h	122	5DGLCNt2rpp	0
114	5dglcn[p]	113	5dglcn	122	5DGLCNt2rpp	1
437	h[p]	98	h	122	5DGLCNt2rpp	0
437	h[p]	113	5dglcn	122	5DGLCNt2rpp	0
115	5dglcn[e]	114	5dglcn[p]	123	5DGLCNtex	1
109	h2o	174	ade	124	5DOAN	0
109	h2o	440	5drib	124	5DOAN	0
116	dad-5	174	ade	124	5DOAN	1
116	dad-5	440	5drib	124	5DOAN	1
117	ru5p-D	742	ara5p	125	A5PISO	1
118	ACP	177	amp	126	AACPS1	0
118	ACP	1192	ppi	126	AACPS1	0
118	ACP	1267	myrsACP	126	AACPS1	1
135	atp	177	amp	126	AACPS1	1
135	atp	1192	ppi	126	AACPS1	0
135	atp	1267	myrsACP	126	AACPS1	0
1616	ttdca	177	amp	126	AACPS1	0
1616	ttdca	1192	ppi	126	AACPS1	0
1616	ttdca	1267	myrsACP	126	AACPS1	1
118	ACP	177	amp	127	AACPS2	0
118	ACP	1192	ppi	127	AACPS2	0
118	ACP	1373	tdeACP	127	AACPS2	1
135	atp	177	amp	127	AACPS2	1
135	atp	1192	ppi	127	AACPS2	0
135	atp	1373	tdeACP	127	AACPS2	0
1617	ttdcea	177	amp	127	AACPS2	0

1617	ttdcea	1192	ppi	127	AACPS2	0
1617	ttdcea	1373	tdeACP	127	AACPS2	1
118	ACP	177	amp	128	AACPS3	0
118	ACP	1192	ppi	128	AACPS3	0
118	ACP	1374	palmACP	128	AACPS3	1
135	atp	177	amp	128	AACPS3	1
135	atp	1192	ppi	128	AACPS3	0
135	atp	1374	palmACP	128	AACPS3	0
1618	hdca	177	amp	128	AACPS3	0
1618	hdca	1192	ppi	128	AACPS3	0
1618	hdca	1374	palmACP	128	AACPS3	1
118	ACP	177	amp	129	AACPS4	0
118	ACP	486	hdeACP	129	AACPS4	1
118	ACP	1192	ppi	129	AACPS4	0
135	atp	177	amp	129	AACPS4	1
135	atp	486	hdeACP	129	AACPS4	0
135	atp	1192	ppi	129	AACPS4	0
1619	hdcea	177	amp	129	AACPS4	0
1619	hdcea	486	hdeACP	129	AACPS4	1
1619	hdcea	1192	ppi	129	AACPS4	0
118	ACP	177	amp	130	AACPS5	0
118	ACP	1192	ppi	130	AACPS5	0
118	ACP	1376	octeACP	130	AACPS5	1
135	atp	177	amp	130	AACPS5	1
135	atp	1192	ppi	130	AACPS5	0
135	atp	1376	octeACP	130	AACPS5	0
1621	ocdcea	177	amp	130	AACPS5	0
1621	ocdcea	1192	ppi	130	AACPS5	0
1621	ocdcea	1376	octeACP	130	AACPS5	1
118	ACP	177	amp	131	AACPS6	0
118	ACP	1192	ppi	131	AACPS6	0
118	ACP	1375	ocdcaACP	131	AACPS6	1
135	atp	177	amp	131	AACPS6	1
135	atp	1192	ppi	131	AACPS6	0
135	atp	1375	ocdcaACP	131	AACPS6	0
1620	ocdca	177	amp	131	AACPS6	0
1620	ocdca	1192	ppi	131	AACPS6	0
1620	ocdca	1375	ocdcaACP	131	AACPS6	1
118	ACP	101	ddcaACP	132	AACPS7	1
118	ACP	177	amp	132	AACPS7	0
118	ACP	1192	ppi	132	AACPS7	0
135	atp	101	ddcaACP	132	AACPS7	0
135	atp	177	amp	132	AACPS7	1
135	atp	1192	ppi	132	AACPS7	0
1303	ddca	101	ddcaACP	132	AACPS7	1
1303	ddca	177	amp	132	AACPS7	0
1303	ddca	1192	ppi	132	AACPS7	0
118	ACP	99	dcaACP	133	AACPS8	1
118	ACP	177	amp	133	AACPS8	0
118	ACP	1192	ppi	133	AACPS8	0
135	atp	99	dcaACP	133	AACPS8	0
135	atp	177	amp	133	AACPS8	1
135	atp	1192	ppi	133	AACPS8	0
1596	dca	99	dcaACP	133	AACPS8	1
1596	dca	177	amp	133	AACPS8	0
1596	dca	1192	ppi	133	AACPS8	0

118	ACP	177	amp	134	AACPS9	0
118	ACP	1192	ppi	134	AACPS9	0
118	ACP	1395	ocACP	134	AACPS9	1
135	atp	177	amp	134	AACPS9	1
135	atp	1192	ppi	134	AACPS9	0
135	atp	1395	ocACP	134	AACPS9	0
1622	octa	177	amp	134	AACPS9	0
1622	octa	1192	ppi	134	AACPS9	0
1622	octa	1395	ocACP	134	AACPS9	1
109	h2o	292	h2o2	135	AACTOOR	0
109	h2o	1160	nh4	135	AACTOOR	0
109	h2o	1321	mthgxl	135	AACTOOR	0
119	aact	292	h2o2	135	AACTOOR	0
119	aact	1160	nh4	135	AACTOOR	0
119	aact	1321	mthgxl	135	AACTOOR	1
928	o2	292	h2o2	135	AACTOOR	0
928	o2	1160	nh4	135	AACTOOR	0
928	o2	1321	mthgxl	135	AACTOOR	0
120	dtdp4aaddg	98	h	136	AADDGT	0
120	dtdp4aaddg	474	unagamuf	136	AADDGT	0
120	dtdp4aaddg	1239	dtdp	136	AADDGT	1
1365	unagamu	98	h	136	AADDGT	0
1365	unagamu	474	unagamuf	136	AADDGT	1
1365	unagamu	1239	dtdp	136	AADDGT	0
121	14glucan	834	malthx	137	AAMYL	1
12	14glucan[p]	1240	malthx[p]	138	AAMYLpp	1
109	h2o	568	g6p	139	AB6PGH	0
109	h2o	1580	hqn	139	AB6PGH	0
122	arbt6p	568	g6p	139	AB6PGH	1
122	arbt6p	1580	hqn	139	AB6PGH	1
123	4abut	624	glu-L	140	ABTA	0
123	4abut	1581	sucsal	140	ABTA	1
213	akg	624	glu-L	140	ABTA	1
213	akg	1581	sucsal	140	ABTA	0
109	h2o	98	h	141	ABUTD	0
109	h2o	123	4abut	141	ABUTD	0
109	h2o	870	nadh	141	ABUTD	0
124	4abutn	98	h	141	ABUTD	0
124	4abutn	123	4abut	141	ABUTD	1
124	4abutn	870	nadh	141	ABUTD	0
856	nad	98	h	141	ABUTD	0
856	nad	123	4abut	141	ABUTD	0
856	nad	870	nadh	141	ABUTD	1
125	4abut[p]	98	h	142	ABUTt2pp	0
125	4abut[p]	123	4abut	142	ABUTt2pp	1
437	h[p]	98	h	142	ABUTt2pp	0
437	h[p]	123	4abut	142	ABUTt2pp	0
126	4abut[e]	125	4abut[p]	143	ABUTtex	1
127	acac	147	ac	144	ACACCT	1
127	acac	734	aacoa	144	ACACCT	1
128	accoa	147	ac	144	ACACCT	0
128	accoa	734	aacoa	144	ACACCT	1
128	accoa	734	aacoa	145	ACACT1r	1
128	accoa	927	coa	145	ACACT1r	1
129	acac[p]	98	h	146	ACACt2pp	0
129	acac[p]	127	acac	146	ACACt2pp	1

437	h[p]	98	h	146	ACACt2pp	0
437	h[p]	127	acac	146	ACACt2pp	0
130	acac[e]	129	acac[p]	147	ACACtex	1
131	acald	98	h	148	ACALD	0
131	acald	128	accoa	148	ACALD	1
131	acald	870	nadh	148	ACALD	0
856	nad	98	h	148	ACALD	0
856	nad	128	accoa	148	ACALD	0
856	nad	870	nadh	148	ACALD	1
927	coa	98	h	148	ACALD	0
927	coa	128	accoa	148	ACALD	1
927	coa	870	nadh	148	ACALD	0
132	acald[e]	133	acald[p]	149	ACALDtex	1
133	acald[p]	131	acald	150	ACALDtpp	1
128	accoa	927	coa	151	ACANTHAT	1
128	accoa	1241	acanth	151	ACANTHAT	1
250	anth	927	coa	151	ACANTHAT	0
250	anth	1241	acanth	151	ACANTHAT	1
98	h	183	agdpcbi	152	ACBIPGT	0
98	h	1192	ppi	152	ACBIPGT	0
134	adocbip	183	agdpcbi	152	ACBIPGT	1
134	adocbip	1192	ppi	152	ACBIPGT	0
673	gtp	183	agdpcbi	152	ACBIPGT	1
673	gtp	1192	ppi	152	ACBIPGT	0
128	accoa	98	h	153	ACCOAC	0
128	accoa	281	adp	153	ACCOAC	0
128	accoa	962	pi	153	ACCOAC	0
128	accoa	1426	malcoa	153	ACCOAC	1
135	atp	98	h	153	ACCOAC	0
135	atp	281	adp	153	ACCOAC	1
135	atp	962	pi	153	ACCOAC	0
135	atp	1426	malcoa	153	ACCOAC	0
1560	hco3	98	h	153	ACCOAC	0
1560	hco3	281	adp	153	ACCOAC	0
1560	hco3	962	pi	153	ACCOAC	0
1560	hco3	1426	malcoa	153	ACCOAC	0
135	atp	281	adp	154	ACCOAL	1
135	atp	948	ppcoa	154	ACCOAL	0
135	atp	962	pi	154	ACCOAL	0
927	coa	281	adp	154	ACCOAL	0
927	coa	948	ppcoa	154	ACCOAL	1
927	coa	962	pi	154	ACCOAL	0
1345	ppa	281	adp	154	ACCOAL	0
1345	ppa	948	ppcoa	154	ACCOAL	1
1345	ppa	962	pi	154	ACCOAL	0
136	acgal1p[p]	1074	acgal[p]	155	ACGAL1PPpp	1
136	acgal1p[p]	1173	pi[p]	155	ACGAL1PPpp	0
684	h2o[p]	1074	acgal[p]	155	ACGAL1PPpp	0
684	h2o[p]	1173	pi[p]	155	ACGAL1PPpp	0
137	acgal1p[e]	136	acgal1p[p]	156	ACGAL1Ptex	1
138	acgal[e]	1074	acgal[p]	157	ACGALtex	1
139	acgam1p[p]	143	acgam[p]	158	ACGAM1PPpp	1
139	acgam1p[p]	1173	pi[p]	158	ACGAM1PPpp	0
684	h2o[p]	143	acgam[p]	158	ACGAM1PPpp	0
684	h2o[p]	1173	pi[p]	158	ACGAM1PPpp	0
140	acgam1p[e]	139	acgam1p[p]	159	ACGAM1Ptex	1

135	atp	98	h	160	ACGAMK	0
135	atp	190	acgam6p	160	ACGAMK	0
135	atp	281	adp	160	ACGAMK	1
141	acgam	98	h	160	ACGAMK	0
141	acgam	190	acgam6p	160	ACGAMK	1
141	acgam	281	adp	160	ACGAMK	0
142	uacgam	1242	ump	161	ACGAMT	1
142	uacgam	1366	unaga	161	ACGAMT	1
923	udcpp	1242	ump	161	ACGAMT	0
923	udcpp	1366	unaga	161	ACGAMT	1
143	acgam[p]	190	acgam6p	162	ACGAptspp	1
143	acgam[p]	1148	pyr	162	ACGAptspp	0
959	pep	190	acgam6p	162	ACGAptspp	0
959	pep	1148	pyr	162	ACGAptspp	1
144	acgam[e]	143	acgam[p]	163	ACGAtex	1
135	atp	281	adp	164	ACGK	1
135	atp	1082	acg5p	164	ACGK	0
145	acglu	281	adp	164	ACGK	0
145	acglu	1082	acg5p	164	ACGK	1
128	accoa	98	h	165	ACGS	0
128	accoa	145	acglu	165	ACGS	1
128	accoa	927	coa	165	ACGS	1
624	glu-L	98	h	165	ACGS	0
624	glu-L	145	acglu	165	ACGS	1
624	glu-L	927	coa	165	ACGS	0
98	h	692	co2	166	ACHBS	0
98	h	732	2ahbut	166	ACHBS	0
146	2obut	692	co2	166	ACHBS	0
146	2obut	732	2ahbut	166	ACHBS	1
1148	pyr	692	co2	166	ACHBS	0
1148	pyr	732	2ahbut	166	ACHBS	1
135	atp	281	adp	167	ACKr	1
135	atp	1075	actp	167	ACKr	0
147	ac	281	adp	167	ACKr	0
147	ac	1075	actp	167	ACKr	1
98	h	692	co2	168	ACLS	0
98	h	1138	alac-S	168	ACLS	0
1148	pyr	692	co2	168	ACLS	0
1148	pyr	1138	alac-S	168	ACLS	1
109	h2o	190	acgam6p	169	ACM6PH	0
109	h2o	748	lac-D	169	ACM6PH	0
148	acmum6p	190	acgam6p	169	ACM6PH	1
148	acmum6p	748	lac-D	169	ACM6PH	1
149	uacmamu	98	h	170	ACMAMUT	0
149	uacmamu	1365	unagamu	170	ACMAMUT	1
149	uacmamu	1440	udp	170	ACMAMUT	1
1366	unaga	98	h	170	ACMAMUT	0
1366	unaga	1365	unagamu	170	ACMAMUT	1
1366	unaga	1440	udp	170	ACMAMUT	0
150	acmana[p]	242	acmanap	171	ACMANAptspp	1
150	acmana[p]	1148	pyr	171	ACMANAptspp	0
959	pep	242	acmanap	171	ACMANAptspp	0
959	pep	1148	pyr	171	ACMANAptspp	1
151	acmana[e]	150	acmana[p]	172	ACMANAtex	1
152	acmum[p]	148	acmum6p	173	ACMUMptspp	1
152	acmum[p]	1148	pyr	173	ACMUMptspp	0

959	pep	148	acmum6p	173	ACMUMptspp	0
959	pep	1148	pyr	173	ACMUMptspp	1
153	acmum[e]	152	acmum[p]	174	ACMUMtex	1
154	acnam[p]	98	h	175	ACNAMt2pp	0
154	acnam[p]	156	acnam	175	ACNAMt2pp	1
437	h[p]	98	h	175	ACNAMt2pp	0
437	h[p]	156	acnam	175	ACNAMt2pp	0
155	acnam[e]	154	acnam[p]	176	ACNAMtex	1
156	acnam	243	acmana	177	ACNML	1
156	acnam	1148	pyr	177	ACNML	1
157	btcoa	520	fadh2	178	ACOAD1f	0
157	btcoa	1076	b2coa	178	ACOAD1f	1
158	fad	520	fadh2	178	ACOAD1f	1
158	fad	1076	b2coa	178	ACOAD1f	0
158	fad	520	fadh2	179	ACOAD2f	1
158	fad	1556	hx2coa	179	ACOAD2f	0
1367	hxcoa	520	fadh2	179	ACOAD2f	0
1367	hxcoa	1556	hx2coa	179	ACOAD2f	1
158	fad	520	fadh2	180	ACOAD3f	1
158	fad	1557	oc2coa	180	ACOAD3f	0
1368	occoa	520	fadh2	180	ACOAD3f	0
1368	occoa	1557	oc2coa	180	ACOAD3f	1
158	fad	520	fadh2	181	ACOAD4f	1
158	fad	1077	dc2coa	181	ACOAD4f	0
159	dcacoa	520	fadh2	181	ACOAD4f	0
159	dcacoa	1077	dc2coa	181	ACOAD4f	1
158	fad	520	fadh2	182	ACOAD5f	1
158	fad	1078	dd2coa	182	ACOAD5f	0
160	ddcacoa	520	fadh2	182	ACOAD5f	0
160	ddcacoa	1078	dd2coa	182	ACOAD5f	1
158	fad	520	fadh2	183	ACOAD6f	1
158	fad	1354	td2coa	183	ACOAD6f	0
1369	tdcoa	520	fadh2	183	ACOAD6f	0
1369	tdcoa	1354	td2coa	183	ACOAD6f	1
158	fad	520	fadh2	184	ACOAD7f	1
158	fad	1291	hdd2coa	184	ACOAD7f	0
1370	pmtcoa	520	fadh2	184	ACOAD7f	0
1370	pmtcoa	1291	hdd2coa	184	ACOAD7f	1
158	fad	520	fadh2	185	ACOAD8f	1
158	fad	1334	od2coa	185	ACOAD8f	0
1371	stcoa	520	fadh2	185	ACOAD8f	0
1371	stcoa	1334	od2coa	185	ACOAD8f	1
118	ACP	733	acACP	186	ACOATA	1
118	ACP	927	coa	186	ACOATA	0
128	accoa	733	acACP	186	ACOATA	1
128	accoa	927	coa	186	ACOATA	1
109	h2o	147	ac	187	ACODA	0
109	h2o	963	orn	187	ACODA	0
161	acorn	147	ac	187	ACODA	1
161	acorn	963	orn	187	ACODA	1
162	acolipa[p]	1243	acolipa[e]	188	ACOLIPAtex	1
163	acon-T	165	acon-C	189	ACONIs	1
163	acon-T	209	ahcys	190	ACONMT	0
163	acon-T	1244	aconm	190	ACONMT	1
178	amet	209	ahcys	190	ACONMT	1
178	amet	1244	aconm	190	ACONMT	1

164	cit	109	h2o	191	ACONTa	0
164	cit	165	acon-C	191	ACONTa	1
109	h2o	716	icit	192	ACONTb	0
165	acon-C	716	icit	192	ACONTb	1
161	acorn	207	acg5sa	193	ACOTA	1
161	acorn	624	glu-L	193	ACOTA	0
213	akg	207	acg5sa	193	ACOTA	0
213	akg	624	glu-L	193	ACOTA	1
166	apoACP	98	h	194	ACPS1	0
166	apoACP	118	ACP	194	ACPS1	1
166	apoACP	1383	pap	194	ACPS1	1
927	coa	98	h	194	ACPS1	0
927	coa	118	ACP	194	ACPS1	0
927	coa	1383	pap	194	ACPS1	1
135	atp	128	accoa	195	ACS	0
135	atp	177	amp	195	ACS	1
135	atp	1192	ppi	195	ACS	0
147	ac	128	accoa	195	ACS	1
147	ac	177	amp	195	ACS	0
147	ac	1192	ppi	195	ACS	0
927	coa	128	accoa	195	ACS	1
927	coa	177	amp	195	ACS	0
927	coa	1192	ppi	195	ACS	0
167	acser[e]	1079	acser[p]	196	ACSERtex	1
168	acser	1079	acser[p]	197	ACSERtpp	1
169	ac[p]	98	h	198	ACt2rpp	0
169	ac[p]	147	ac	198	ACt2rpp	1
437	h[p]	98	h	198	ACt2rpp	0
437	h[p]	147	ac	198	ACt2rpp	0
169	ac[p]	147	ac	199	ACt4pp	1
169	ac[p]	1344	na1	199	ACt4pp	0
941	na1[p]	147	ac	199	ACt4pp	0
941	na1[p]	1344	na1	199	ACt4pp	0
170	ac[e]	169	ac[p]	200	ACtex	1
98	h	967	ins	201	ADA	0
98	h	1160	nh4	201	ADA	0
109	h2o	967	ins	201	ADA	0
109	h2o	1160	nh4	201	ADA	0
171	adn	967	ins	201	ADA	1
171	adn	1160	nh4	201	ADA	0
172	4adcho	98	h	202	ADCL	0
172	4adcho	427	4abz	202	ADCL	1
172	4adcho	1148	pyr	202	ADCL	1
173	chor	172	4adcho	203	ADCS	1
173	chor	624	glu-L	203	ADCS	0
625	gln-L	172	4adcho	203	ADCS	0
625	gln-L	624	glu-L	203	ADCS	1
98	h	713	hxan	204	ADD	0
98	h	1160	nh4	204	ADD	0
109	h2o	713	hxan	204	ADD	0
109	h2o	1160	nh4	204	ADD	0
174	ade	713	hxan	204	ADD	1
174	ade	1160	nh4	204	ADD	0
175	ade[p]	98	h	205	ADEt2rpp	0
175	ade[p]	174	ade	205	ADEt2rpp	1
437	h[p]	98	h	205	ADEt2rpp	0

437	h[p]	174	ade	205	ADEt2rpp	0
176	ade[e]	175	ade[p]	206	ADEtex	1
135	atp	281	adp	207	ADK1	1
177	amp	281	adp	207	ADK1	1
177	amp	281	adp	208	ADK3	1
177	amp	798	gdp	208	ADK3	0
673	gtp	281	adp	208	ADK3	0
673	gtp	798	gdp	208	ADK3	1
177	amp	281	adp	209	ADK4	1
177	amp	1558	idp	209	ADK4	0
1249	itp	281	adp	209	ADK4	0
1249	itp	1558	idp	209	ADK4	1
98	h	692	co2	210	ADMDC	0
98	h	1000	ametam	210	ADMDC	0
178	amet	692	co2	210	ADMDC	0
178	amet	1000	ametam	210	ADMDC	1
135	atp	926	camp	211	ADNCYC	1
135	atp	1192	ppi	211	ADNCYC	0
135	atp	98	h	212	ADNK1	0
135	atp	177	amp	212	ADNK1	1
135	atp	281	adp	212	ADNK1	1
171	adn	98	h	212	ADNK1	0
171	adn	177	amp	212	ADNK1	1
171	adn	281	adp	212	ADNK1	1
179	adn[p]	98	h	213	ADNt2pp	0
179	adn[p]	171	adn	213	ADNt2pp	1
437	h[p]	98	h	213	ADNt2pp	0
437	h[p]	171	adn	213	ADNt2pp	0
179	adn[p]	98	h	214	ADNt2rpp	0
179	adn[p]	171	adn	214	ADNt2rpp	1
437	h[p]	98	h	214	ADNt2rpp	0
437	h[p]	171	adn	214	ADNt2rpp	0
180	adn[e]	179	adn[p]	215	ADNtex	1
109	h2o	174	ade	216	ADNUC	0
109	h2o	1361	rib-D	216	ADNUC	0
171	adn	174	ade	216	ADNUC	1
171	adn	1361	rib-D	216	ADNUC	1
135	atp	98	h	217	ADOCBIK	0
135	atp	134	adocbip	217	ADOCBIK	0
135	atp	281	adp	217	ADOCBIK	1
181	adocbi	98	h	217	ADOCBIK	0
181	adocbi	134	adocbip	217	ADOCBIK	1
181	adocbi	281	adp	217	ADOCBIK	0
109	h2o	98	h	218	ADOCBLabcpp	0
109	h2o	281	adp	218	ADOCBLabcpp	0
109	h2o	962	pi	218	ADOCBLabcpp	0
109	h2o	1092	adocbl	218	ADOCBLabcpp	0
135	atp	98	h	218	ADOCBLabcpp	0
135	atp	281	adp	218	ADOCBLabcpp	1
135	atp	962	pi	218	ADOCBLabcpp	0
135	atp	1092	adocbl	218	ADOCBLabcpp	0
182	adocbl[p]	98	h	218	ADOCBLabcpp	0
182	adocbl[p]	281	adp	218	ADOCBLabcpp	0
182	adocbl[p]	962	pi	218	ADOCBLabcpp	0
182	adocbl[p]	1092	adocbl	218	ADOCBLabcpp	1
183	agdpcbi	98	h	219	ADOCBLS	0

183	agdpcbi	650	gmp	219	ADOCBLS	0
183	agdpcbi	1092	adocbl	219	ADOCBLS	1
1372	rdmbzi	98	h	219	ADOCBLS	0
1372	rdmbzi	650	gmp	219	ADOCBLS	1
1372	rdmbzi	1092	adocbl	219	ADOCBLS	1
184	adocbl[e]	98	h	220	ADOCBLtonex	0
184	adocbl[e]	182	adocbl[p]	220	ADOCBLtonex	1
437	h[p]	98	h	220	ADOCBLtonex	0
437	h[p]	182	adocbl[p]	220	ADOCBLtonex	0
109	h2o	98	h	221	ADPRDP	0
109	h2o	177	amp	221	ADPRDP	0
109	h2o	983	r5p	221	ADPRDP	0
185	adprib	98	h	221	ADPRDP	0
185	adprib	177	amp	221	ADPRDP	1
185	adprib	983	r5p	221	ADPRDP	1
174	ade	177	amp	222	ADPT	1
174	ade	1192	ppi	222	ADPT	0
1052	prpp	177	amp	222	ADPT	1
1052	prpp	1192	ppi	222	ADPT	0
135	atp	98	h	223	ADSK	0
135	atp	281	adp	223	ADSK	1
135	atp	924	paps	223	ADSK	0
186	aps	98	h	223	ADSK	0
186	aps	281	adp	223	ADSK	0
186	aps	924	paps	223	ADSK	1
187	dcamp	177	amp	224	ADSL1r	1
187	dcamp	540	fum	224	ADSL1r	1
188	25aics	540	fum	225	ADSL2r	1
188	25aics	1080	aicar	225	ADSL2r	1
189	asp-L	98	h	226	ADSS	0
189	asp-L	187	dcamp	226	ADSS	1
189	asp-L	798	gdp	226	ADSS	0
189	asp-L	962	pi	226	ADSS	0
673	gtp	98	h	226	ADSS	0
673	gtp	187	dcamp	226	ADSS	0
673	gtp	798	gdp	226	ADSS	1
673	gtp	962	pi	226	ADSS	0
1287	imp	98	h	226	ADSS	0
1287	imp	187	dcamp	226	ADSS	1
1287	imp	798	gdp	226	ADSS	0
1287	imp	962	pi	226	ADSS	0
109	h2o	147	ac	227	AGDC	0
109	h2o	567	gam6p	227	AGDC	0
190	acgam6p	147	ac	227	AGDC	1
190	acgam6p	567	gam6p	227	AGDC	1
109	h2o	195	anhgm	228	AGM3PA	0
109	h2o	745	LalaDgluMdap	228	AGM3PA	0
191	anhgm3p	195	anhgm	228	AGM3PA	1
191	anhgm3p	745	LalaDgluMdap	228	AGM3PA	1
192	anhgm3p[p]	104	LalaDgluMdap[p]	229	AGM3PApp	1
192	anhgm3p[p]	198	anhgm[p]	229	AGM3PApp	1
684	h2o[p]	104	LalaDgluMdap[p]	229	AGM3PApp	0
684	h2o[p]	198	anhgm[p]	229	AGM3PApp	0
109	h2o	141	acgam	230	AGM3PH	0
109	h2o	239	anhm3p	230	AGM3PH	0
191	anhgm3p	141	acgam	230	AGM3PH	1

191	anhgm3p	239	anhm3p	230	AGM3PH	1
192	anhgm3p[p]	98	h	231	AGM3Pt2pp	0
192	anhgm3p[p]	191	anhgm3p	231	AGM3Pt2pp	1
437	h[p]	98	h	231	AGM3Pt2pp	0
437	h[p]	191	anhgm3p	231	AGM3Pt2pp	0
109	h2o	110	LalaDgluMdapDala	232	AGM4PA	0
109	h2o	195	anhgm	232	AGM4PA	0
193	anhgm4p	110	LalaDgluMdapDala	232	AGM4PA	1
193	anhgm4p	195	anhgm	232	AGM4PA	1
194	anhgm4p[p]	111	LalaDgluMdapDala[p]	233	AGM4PApp	1
194	anhgm4p[p]	198	anhgm[p]	233	AGM4PApp	1
684	h2o[p]	111	LalaDgluMdapDala[p]	233	AGM4PApp	0
684	h2o[p]	198	anhgm[p]	233	AGM4PApp	0
109	h2o	191	anhgm3p	234	AGM4PCP	0
109	h2o	219	ala-D	234	AGM4PCP	0
193	anhgm4p	191	anhgm3p	234	AGM4PCP	1
193	anhgm4p	219	ala-D	234	AGM4PCP	1
194	anhgm4p[p]	192	anhgm3p[p]	235	AGM4PCPpp	1
194	anhgm4p[p]	384	ala-D[p]	235	AGM4PCPpp	1
684	h2o[p]	192	anhgm3p[p]	235	AGM4PCPpp	0
684	h2o[p]	384	ala-D[p]	235	AGM4PCPpp	0
109	h2o	141	acgam	236	AGM4PH	0
109	h2o	240	anhm4p	236	AGM4PH	0
193	anhgm4p	141	acgam	236	AGM4PH	1
193	anhgm4p	240	anhm4p	236	AGM4PH	1
194	anhgm4p[p]	98	h	237	AGM4Pt2pp	0
194	anhgm4p[p]	193	anhgm4p	237	AGM4Pt2pp	1
437	h[p]	98	h	237	AGM4Pt2pp	0
437	h[p]	193	anhgm4p	237	AGM4Pt2pp	0
109	h2o	141	acgam	238	AGMH	0
109	h2o	249	anhm	238	AGMH	0
195	anhgm	141	acgam	238	AGMH	1
195	anhgm	249	anhm	238	AGMH	1
196	adphep-DD	698	adphep-LD	239	AGMHE	1
109	h2o	1228	urea	240	AGMT	0
109	h2o	1245	ptrc	240	AGMT	0
197	agm	1228	urea	240	AGMT	1
197	agm	1245	ptrc	240	AGMT	1
198	anhgm[p]	98	h	241	AGMt2pp	0
198	anhgm[p]	195	anhgm	241	AGMt2pp	1
437	h[p]	98	h	241	AGMt2pp	0
437	h[p]	195	anhgm	241	AGMt2pp	0
199	agm[e]	1081	agm[p]	242	AGMtex	1
101	ddcaACP	118	ACP	243	AGPAT120	1
101	ddcaACP	1452	pa120	243	AGPAT120	1
200	1ddecg3p	118	ACP	243	AGPAT120	0
200	1ddecg3p	1452	pa120	243	AGPAT120	1
201	1tdecg3p	118	ACP	244	AGPAT140	0
201	1tdecg3p	1454	pa140	244	AGPAT140	1
1267	myrsACP	118	ACP	244	AGPAT140	1
1267	myrsACP	1454	pa140	244	AGPAT140	1
202	1tdec7eg3p	118	ACP	245	AGPAT141	0
202	1tdec7eg3p	1456	pa141	245	AGPAT141	1
1373	tdeACP	118	ACP	245	AGPAT141	1
1373	tdeACP	1456	pa141	245	AGPAT141	1
203	1hdecg3p	118	ACP	246	AGPAT160	0

203	1hdecg3p	1458	pa160	246	AGPAT160	1
1374	palmACP	118	ACP	246	AGPAT160	1
1374	palmACP	1458	pa160	246	AGPAT160	1
204	1hdec9eg3p	118	ACP	247	AGPAT161	0
204	1hdec9eg3p	1460	pa161	247	AGPAT161	1
486	hdeACP	118	ACP	247	AGPAT161	1
486	hdeACP	1460	pa161	247	AGPAT161	1
205	1odecg3p	118	ACP	248	AGPAT180	0
205	1odecg3p	1462	pa180	248	AGPAT180	1
1375	ocdcaACP	118	ACP	248	AGPAT180	1
1375	ocdcaACP	1462	pa180	248	AGPAT180	1
206	1odec11eg3p	118	ACP	249	AGPAT181	0
206	1odec11eg3p	1464	pa181	249	AGPAT181	1
1376	octeACP	118	ACP	249	AGPAT181	1
1376	octeACP	1464	pa181	249	AGPAT181	1
207	acg5sa	98	h	250	AGPR	0
207	acg5sa	871	nadph	250	AGPR	0
207	acg5sa	1082	acg5p	250	AGPR	1
459	nadp	98	h	250	AGPR	0
459	nadp	871	nadph	250	AGPR	1
459	nadp	1082	acg5p	250	AGPR	0
962	pi	98	h	250	AGPR	0
962	pi	871	nadph	250	AGPR	0
962	pi	1082	acg5p	250	AGPR	0
208	ag	98	h	251	AGt3	0
208	ag	1246	ag[e]	251	AGt3	0
711	h[e]	98	h	251	AGt3	0
711	h[e]	1246	ag[e]	251	AGt3	0
109	h2o	174	ade	252	AHCYSNS	0
109	h2o	975	rhcys	252	AHCYSNS	0
209	ahcys	174	ade	252	AHCYSNS	1
209	ahcys	975	rhcys	252	AHCYSNS	1
210	10fthf	1083	fprica	253	AICART	1
210	10fthf	1403	thf	253	AICART	1
1080	aicar	1083	fprica	253	AICART	1
1080	aicar	1403	thf	253	AICART	0
135	atp	98	h	254	AIRC2	0
135	atp	281	adp	254	AIRC2	1
135	atp	962	pi	254	AIRC2	0
135	atp	1084	5caiz	254	AIRC2	0
211	air	98	h	254	AIRC2	0
211	air	281	adp	254	AIRC2	0
211	air	962	pi	254	AIRC2	0
211	air	1084	5caiz	254	AIRC2	1
1560	hco3	98	h	254	AIRC2	0
1560	hco3	281	adp	254	AIRC2	0
1560	hco3	962	pi	254	AIRC2	0
1560	hco3	1084	5caiz	254	AIRC2	0
212	5aizc	1084	5caiz	255	AIRC3	1
213	akg	692	co2	256	AKGDH	0
213	akg	838	succoa	256	AKGDH	1
213	akg	870	nadh	256	AKGDH	0
856	nad	692	co2	256	AKGDH	0
856	nad	838	succoa	256	AKGDH	0
856	nad	870	nadh	256	AKGDH	1
927	coa	692	co2	256	AKGDH	0

927	coa	838	succoa	256	AKGDH	1
927	coa	870	nadh	256	AKGDH	0
214	akg[p]	98	h	257	AKGt2rpp	0
214	akg[p]	213	akg	257	AKGt2rpp	1
437	h[p]	98	h	257	AKGt2rpp	0
437	h[p]	213	akg	257	AKGt2rpp	0
215	akg[e]	214	akg[p]	258	AKGtex	1
109	h2o	98	h	259	ALAabcpp	0
109	h2o	222	ala-L	259	ALAabcpp	0
109	h2o	281	adp	259	ALAabcpp	0
109	h2o	962	pi	259	ALAabcpp	0
135	atp	98	h	259	ALAabcpp	0
135	atp	222	ala-L	259	ALAabcpp	0
135	atp	281	adp	259	ALAabcpp	1
135	atp	962	pi	259	ALAabcpp	0
216	ala-L[p]	98	h	259	ALAabcpp	0
216	ala-L[p]	222	ala-L	259	ALAabcpp	1
216	ala-L[p]	281	adp	259	ALAabcpp	0
216	ala-L[p]	962	pi	259	ALAabcpp	0
109	h2o	98	h	260	ALAALAabcpp	0
109	h2o	218	alaala	260	ALAALAabcpp	0
109	h2o	281	adp	260	ALAALAabcpp	0
109	h2o	962	pi	260	ALAALAabcpp	0
135	atp	98	h	260	ALAALAabcpp	0
135	atp	218	alaala	260	ALAALAabcpp	0
135	atp	281	adp	260	ALAALAabcpp	1
135	atp	962	pi	260	ALAALAabcpp	0
217	alaala[p]	98	h	260	ALAALAabcpp	0
217	alaala[p]	218	alaala	260	ALAALAabcpp	1
217	alaala[p]	281	adp	260	ALAALAabcpp	0
217	alaala[p]	962	pi	260	ALAALAabcpp	0
109	h2o	219	ala-D	261	ALAALAD	0
218	alaala	219	ala-D	261	ALAALAD	1
135	atp	98	h	262	ALAALAr	0
135	atp	218	alaala	262	ALAALAr	0
135	atp	281	adp	262	ALAALAr	1
135	atp	962	pi	262	ALAALAr	0
219	ala-D	98	h	262	ALAALAr	0
219	ala-D	218	alaala	262	ALAALAr	1
219	ala-D	281	adp	262	ALAALAr	0
219	ala-D	962	pi	262	ALAALAr	0
220	alaala[e]	217	alaala[p]	263	ALAALAtex	1
221	LalaDglu	746	LalaLglu	264	ALAGLUE	1
222	ala-L	219	ala-D	265	ALAR	1
216	ala-L[p]	98	h	266	ALAt2pp	0
216	ala-L[p]	222	ala-L	266	ALAt2pp	1
437	h[p]	98	h	266	ALAt2pp	0
437	h[p]	222	ala-L	266	ALAt2pp	0
216	ala-L[p]	222	ala-L	267	ALAt4pp	1
216	ala-L[p]	1344	na1	267	ALAt4pp	0
941	na1[p]	222	ala-L	267	ALAt4pp	0
941	na1[p]	1344	na1	267	ALAt4pp	0
219	ala-D	1148	pyr	268	ALATA_D2	1
219	ala-D	1247	pyam5p	268	ALATA_D2	0
1377	pydx5p	1148	pyr	268	ALATA_D2	0
1377	pydx5p	1247	pyam5p	268	ALATA_D2	1

213	akg	624	glu-L	269	ALATA_L	1
213	akg	1148	pyr	269	ALATA_L	0
222	ala-L	624	glu-L	269	ALATA_L	0
222	ala-L	1148	pyr	269	ALATA_L	1
222	ala-L	1148	pyr	270	ALATA_L2	1
222	ala-L	1247	pyam5p	270	ALATA_L2	0
1377	pydx5p	1148	pyr	270	ALATA_L2	0
1377	pydx5p	1247	pyam5p	270	ALATA_L2	1
223	ala-L[e]	216	ala-L[p]	271	ALAtex	1
135	atp	177	amp	272	ALATRS	1
135	atp	1192	ppi	272	ALATRS	0
135	atp	1248	alatrna	272	ALATRS	0
222	ala-L	177	amp	272	ALATRS	0
222	ala-L	1192	ppi	272	ALATRS	0
222	ala-L	1248	alatrna	272	ALATRS	1
1623	trnaala	177	amp	272	ALATRS	0
1623	trnaala	1192	ppi	272	ALATRS	0
1623	trnaala	1248	alatrna	272	ALATRS	1
98	h	641	glyc	273	ALCD19	0
98	h	856	nad	273	ALCD19	0
224	glyald	641	glyc	273	ALCD19	1
224	glyald	856	nad	273	ALCD19	0
870	nadh	641	glyc	273	ALCD19	0
870	nadh	856	nad	273	ALCD19	1
225	etoh	98	h	274	ALCD2x	0
225	etoh	131	acald	274	ALCD2x	1
225	etoh	870	nadh	274	ALCD2x	0
856	nad	98	h	274	ALCD2x	0
856	nad	131	acald	274	ALCD2x	0
856	nad	870	nadh	274	ALCD2x	1
109	h2o	98	h	275	ALDD19x	0
109	h2o	870	nadh	275	ALDD19x	0
109	h2o	1640	pac	275	ALDD19x	0
856	nad	98	h	275	ALDD19x	0
856	nad	870	nadh	275	ALDD19x	1
856	nad	1640	pac	275	ALDD19x	0
1561	pacald	98	h	275	ALDD19x	0
1561	pacald	870	nadh	275	ALDD19x	0
1561	pacald	1640	pac	275	ALDD19x	1
109	h2o	98	h	276	ALDD2x	0
109	h2o	147	ac	276	ALDD2x	0
109	h2o	870	nadh	276	ALDD2x	0
131	acald	98	h	276	ALDD2x	0
131	acald	147	ac	276	ALDD2x	1
131	acald	870	nadh	276	ALDD2x	0
856	nad	98	h	276	ALDD2x	0
856	nad	147	ac	276	ALDD2x	0
856	nad	870	nadh	276	ALDD2x	1
109	h2o	98	h	277	ALDD2y	0
109	h2o	147	ac	277	ALDD2y	0
109	h2o	871	nadph	277	ALDD2y	0
131	acald	98	h	277	ALDD2y	0
131	acald	147	ac	277	ALDD2y	1
131	acald	871	nadph	277	ALDD2y	0
459	nadp	98	h	277	ALDD2y	0
459	nadp	147	ac	277	ALDD2y	0

459	nadp	871	nadph	277	ALDD2y	1
109	h2o	98	h	278	ALDD3y	0
109	h2o	871	nadph	278	ALDD3y	0
109	h2o	1345	ppa	278	ALDD3y	0
459	nadp	98	h	278	ALDD3y	0
459	nadp	871	nadph	278	ALDD3y	1
459	nadp	1345	ppa	278	ALDD3y	0
944	ppal	98	h	278	ALDD3y	0
944	ppal	871	nadph	278	ALDD3y	0
944	ppal	1345	ppa	278	ALDD3y	1
109	h2o	98	h	279	ALDD4	0
109	h2o	870	nadh	279	ALDD4	0
109	h2o	1090	but	279	ALDD4	0
226	btal	98	h	279	ALDD4	0
226	btal	870	nadh	279	ALDD4	0
226	btal	1090	but	279	ALDD4	1
856	nad	98	h	279	ALDD4	0
856	nad	870	nadh	279	ALDD4	1
856	nad	1090	but	279	ALDD4	0
109	h2o	98	h	280	ALLabcpp	0
109	h2o	228	all-D	280	ALLabcpp	0
109	h2o	281	adp	280	ALLabcpp	0
109	h2o	962	pi	280	ALLabcpp	0
135	atp	98	h	280	ALLabcpp	0
135	atp	228	all-D	280	ALLabcpp	0
135	atp	281	adp	280	ALLabcpp	1
135	atp	962	pi	280	ALLabcpp	0
227	all-D[p]	98	h	280	ALLabcpp	0
227	all-D[p]	228	all-D	280	ALLabcpp	1
227	all-D[p]	281	adp	280	ALLabcpp	0
227	all-D[p]	962	pi	280	ALLabcpp	0
135	atp	98	h	281	ALLK	0
135	atp	229	all6p	281	ALLK	0
135	atp	281	adp	281	ALLK	1
228	all-D	98	h	281	ALLK	0
228	all-D	229	all6p	281	ALLK	1
228	all-D	281	adp	281	ALLK	0
229	all6p	235	allul6p	282	ALLPI	1
98	h	692	co2	283	ALLTAMH	0
98	h	1160	nh4	283	ALLTAMH	0
98	h	1553	urdglyc	283	ALLTAMH	0
109	h2o	692	co2	283	ALLTAMH	0
109	h2o	1160	nh4	283	ALLTAMH	0
109	h2o	1553	urdglyc	283	ALLTAMH	0
230	alltt	692	co2	283	ALLTAMH	0
230	alltt	1160	nh4	283	ALLTAMH	0
230	alltt	1553	urdglyc	283	ALLTAMH	1
231	all-D[e]	227	all-D[p]	284	ALLtex	1
109	h2o	98	h	285	ALLTN	0
109	h2o	230	alltt	285	ALLTN	0
232	alltn	98	h	285	ALLTN	0
232	alltn	230	alltt	285	ALLTN	1
233	alltn[p]	98	h	286	ALLTNt2rpp	0
233	alltn[p]	232	alltn	286	ALLTNt2rpp	1
437	h[p]	98	h	286	ALLTNt2rpp	0
437	h[p]	232	alltn	286	ALLTNt2rpp	0

234	alltn[e]	233	alltn[p]	287	ALLTNtex	1
235	allul6p	499	f6p	288	ALLULPE	1
236	alpp[p]	35	2agpe160[p]	289	ALPATE160pp	0
236	alpp[p]	1582	lpp[p]	289	ALPATE160pp	1
1378	pe160[p]	35	2agpe160[p]	289	ALPATE160pp	1
1378	pe160[p]	1582	lpp[p]	289	ALPATE160pp	1
236	alpp[p]	49	2agpg160[p]	290	ALPATG160pp	0
236	alpp[p]	1582	lpp[p]	290	ALPATG160pp	1
324	pg160[p]	49	2agpg160[p]	290	ALPATG160pp	1
324	pg160[p]	1582	lpp[p]	290	ALPATG160pp	1
98	h	237	acetol	291	ALR2	0
98	h	459	nadp	291	ALR2	0
871	nadph	237	acetol	291	ALR2	0
871	nadph	459	nadp	291	ALR2	1
1321	mthgxl	237	acetol	291	ALR2	1
1321	mthgxl	459	nadp	291	ALR2	0
98	h	856	nad	292	ALR4x	0
98	h	1069	12ppd-R	292	ALR4x	0
237	acetol	856	nad	292	ALR4x	0
237	acetol	1069	12ppd-R	292	ALR4x	1
870	nadh	856	nad	292	ALR4x	1
870	nadh	1069	12ppd-R	292	ALR4x	0
238	altrn	109	h2o	293	ALTRH	0
238	altrn	404	2ddglcn	293	ALTRH	1
109	h2o	249	anhm	294	AM3PA	0
109	h2o	745	LalaDgluMdap	294	AM3PA	0
239	anhm3p	249	anhm	294	AM3PA	1
239	anhm3p	745	LalaDgluMdap	294	AM3PA	1
109	h2o	110	LalaDgluMdapDala	295	AM4PA	0
109	h2o	249	anhm	295	AM4PA	0
240	anhm4p	110	LalaDgluMdapDala	295	AM4PA	1
240	anhm4p	249	anhm	295	AM4PA	1
109	h2o	219	ala-D	296	AM4PCP	0
109	h2o	239	anhm3p	296	AM4PCP	0
240	anhm4p	219	ala-D	296	AM4PCP	1
240	anhm4p	239	anhm3p	296	AM4PCP	1
241	malt	1064	glc-D	297	AMALT1	1
241	malt	1380	maltttr	297	AMALT1	1
1379	malttr	1064	glc-D	297	AMALT1	0
1379	malttr	1380	maltttr	297	AMALT1	1
241	malt	833	maltpt	298	AMALT2	1
241	malt	1064	glc-D	298	AMALT2	1
1380	maltttr	833	maltpt	298	AMALT2	1
1380	maltttr	1064	glc-D	298	AMALT2	0
241	malt	834	malthx	299	AMALT3	1
241	malt	1064	glc-D	299	AMALT3	1
833	maltpt	834	malthx	299	AMALT3	1
833	maltpt	1064	glc-D	299	AMALT3	0
241	malt	835	malthp	300	AMALT4	1
241	malt	1064	glc-D	300	AMALT4	1
834	malthx	835	malthp	300	AMALT4	1
834	malthx	1064	glc-D	300	AMALT4	0
242	acmanap	190	acgam6p	301	AMANAPEr	1
135	atp	98	h	302	AMANK	0
135	atp	242	acmanap	302	AMANK	0
135	atp	281	adp	302	AMANK	1

243	acmana	98	h	302	AMANK	0
243	acmana	242	acmanap	302	AMANK	1
243	acmana	281	adp	302	AMANK	0
178	amet	1085	amob	303	AMAOTr	1
178	amet	1559	dann	303	AMAOTr	0
244	8aonn	1085	amob	303	AMAOTr	0
244	8aonn	1559	dann	303	AMAOTr	1
178	amet	98	h	304	AMMQLT8	0
178	amet	209	ahcys	304	AMMQLT8	1
178	amet	867	mql8	304	AMMQLT8	1
245	2dmmql8	98	h	304	AMMQLT8	0
245	2dmmql8	209	ahcys	304	AMMQLT8	0
245	2dmmql8	867	mql8	304	AMMQLT8	1
109	h2o	98	h	305	AMPMS2	0
109	h2o	536	for	305	AMPMS2	0
109	h2o	870	nadh	305	AMPMS2	0
109	h2o	940	4ampm	305	AMPMS2	0
211	air	98	h	305	AMPMS2	0
211	air	536	for	305	AMPMS2	1
211	air	870	nadh	305	AMPMS2	0
211	air	940	4ampm	305	AMPMS2	1
856	nad	98	h	305	AMPMS2	0
856	nad	536	for	305	AMPMS2	0
856	nad	870	nadh	305	AMPMS2	1
856	nad	940	4ampm	305	AMPMS2	0
109	h2o	174	ade	306	AMPN	0
109	h2o	983	r5p	306	AMPN	0
177	amp	174	ade	306	AMPN	1
177	amp	983	r5p	306	AMPN	1
109	h2o	366	cys-L	307	AMPTASECG	0
109	h2o	642	gly	307	AMPTASECG	0
246	cgly	366	cys-L	307	AMPTASECG	1
246	cgly	642	gly	307	AMPTASECG	1
109	h2o	642	gly	308	AMPTASEPG	0
109	h2o	1498	pro-L	308	AMPTASEPG	0
1381	progly	642	gly	308	AMPTASEPG	1
1381	progly	1498	pro-L	308	AMPTASEPG	1
247	amp[e]	886	amp[p]	309	AMPtex	1
248	anhgm[e]	198	anhgm[p]	310	ANHGMtex	1
109	h2o	98	h	311	ANHMK	0
109	h2o	148	acmum6p	311	ANHMK	0
109	h2o	281	adp	311	ANHMK	0
135	atp	98	h	311	ANHMK	0
135	atp	148	acmum6p	311	ANHMK	0
135	atp	281	adp	311	ANHMK	1
249	anhm	98	h	311	ANHMK	0
249	anhm	148	acmum6p	311	ANHMK	1
249	anhm	281	adp	311	ANHMK	0
250	anth	954	pran	312	ANPRT	1
250	anth	1192	ppi	312	ANPRT	0
1052	prpp	954	pran	312	ANPRT	1
1052	prpp	1192	ppi	312	ANPRT	0
173	chor	98	h	313	ANS	0
173	chor	250	anth	313	ANS	1
173	chor	624	glu-L	313	ANS	0
173	chor	1148	pyr	313	ANS	1

625	gln-L	98	h	313	ANS	0
625	gln-L	250	anth	313	ANS	0
625	gln-L	624	glu-L	313	ANS	1
625	gln-L	1148	pyr	313	ANS	0
98	h	119	aact	314	AOBUTDs	0
98	h	692	co2	314	AOBUTDs	0
251	2aobut	119	aact	314	AOBUTDs	1
251	2aobut	692	co2	314	AOBUTDs	0
98	h	244	8aonn	315	AOXSr	0
98	h	692	co2	315	AOXSr	0
98	h	927	coa	315	AOXSr	0
222	ala-L	244	8aonn	315	AOXSr	1
222	ala-L	692	co2	315	AOXSr	0
222	ala-L	927	coa	315	AOXSr	0
1624	pmcoa	244	8aonn	315	AOXSr	1
1624	pmcoa	692	co2	315	AOXSr	0
1624	pmcoa	927	coa	315	AOXSr	1
109	h2o	98	h	316	AP4AH	0
109	h2o	281	adp	316	AP4AH	0
252	ap4a	98	h	316	AP4AH	0
252	ap4a	281	adp	316	AP4AH	1
109	h2o	98	h	317	AP5AH	0
109	h2o	135	atp	317	AP5AH	0
109	h2o	281	adp	317	AP5AH	0
253	ap5a	98	h	317	AP5AH	0
253	ap5a	135	atp	317	AP5AH	1
253	ap5a	281	adp	317	AP5AH	1
98	h	459	nadp	318	APRAUR	0
98	h	939	5aprbu	318	APRAUR	0
254	5apru	459	nadp	318	APRAUR	0
254	5apru	939	5aprbu	318	APRAUR	1
871	nadph	459	nadp	318	APRAUR	1
871	nadph	939	5aprbu	318	APRAUR	0
255	arab-L	1086	rbl-L	319	ARAI	1
109	h2o	98	h	320	ARBabcpp	0
109	h2o	255	arab-L	320	ARBabcpp	0
109	h2o	281	adp	320	ARBabcpp	0
109	h2o	962	pi	320	ARBabcpp	0
135	atp	98	h	320	ARBabcpp	0
135	atp	255	arab-L	320	ARBabcpp	0
135	atp	281	adp	320	ARBabcpp	1
135	atp	962	pi	320	ARBabcpp	0
256	arab-L[p]	98	h	320	ARBabcpp	0
256	arab-L[p]	255	arab-L	320	ARBabcpp	1
256	arab-L[p]	281	adp	320	ARBabcpp	0
256	arab-L[p]	962	pi	320	ARBabcpp	0
256	arab-L[p]	98	h	321	ARBt2rpp	0
256	arab-L[p]	255	arab-L	321	ARBt2rpp	1
437	h[p]	98	h	321	ARBt2rpp	0
437	h[p]	255	arab-L	321	ARBt2rpp	0
255	arab-L	98	h	322	ARBt3ipp	0
255	arab-L	256	arab-L[p]	322	ARBt3ipp	1
437	h[p]	98	h	322	ARBt3ipp	0
437	h[p]	256	arab-L[p]	322	ARBt3ipp	0
257	arab-L[e]	256	arab-L[p]	323	ARBtex	1
109	h2o	98	h	324	ARBTNabcpp	0

109	h2o	260	arbtn-fe3	324	ARBTNabcpp	0
109	h2o	281	adp	324	ARBTNabcpp	0
109	h2o	962	pi	324	ARBTNabcpp	0
135	atp	98	h	324	ARBTNabcpp	0
135	atp	260	arbtn-fe3	324	ARBTNabcpp	0
135	atp	281	adp	324	ARBTNabcpp	1
135	atp	962	pi	324	ARBTNabcpp	0
258	arbtn-fe3[p]	98	h	324	ARBTNabcpp	0
258	arbtn-fe3[p]	260	arbtn-fe3	324	ARBTNabcpp	1
258	arbtn-fe3[p]	281	adp	324	ARBTNabcpp	0
258	arbtn-fe3[p]	962	pi	324	ARBTNabcpp	0
259	arbtn[e]	262	arbtn-fe3[e]	325	ARBTNexs	1
519	fe3[e]	262	arbtn-fe3[e]	325	ARBTNexs	0
260	arbtn-fe3	98	h	326	ARBTNR1	0
260	arbtn-fe3	158	fad	326	ARBTNR1	0
260	arbtn-fe3	263	arbtn	326	ARBTNR1	1
260	arbtn-fe3	509	fe2	326	ARBTNR1	0
520	fadh2	98	h	326	ARBTNR1	0
520	fadh2	158	fad	326	ARBTNR1	1
520	fadh2	263	arbtn	326	ARBTNR1	0
520	fadh2	509	fe2	326	ARBTNR1	0
260	arbtn-fe3	98	h	327	ARBTNR2	0
260	arbtn-fe3	263	arbtn	327	ARBTNR2	1
260	arbtn-fe3	509	fe2	327	ARBTNR2	0
260	arbtn-fe3	538	fmn	327	ARBTNR2	0
511	fmnh2	98	h	327	ARBTNR2	0
511	fmnh2	263	arbtn	327	ARBTNR2	0
511	fmnh2	509	fe2	327	ARBTNR2	0
511	fmnh2	538	fmn	327	ARBTNR2	1
260	arbtn-fe3	98	h	328	ARBTNR3	0
260	arbtn-fe3	263	arbtn	328	ARBTNR3	1
260	arbtn-fe3	509	fe2	328	ARBTNR3	0
260	arbtn-fe3	1515	ribflv	328	ARBTNR3	0
1382	rbflvrd	98	h	328	ARBTNR3	0
1382	rbflvrd	263	arbtn	328	ARBTNR3	0
1382	rbflvrd	509	fe2	328	ARBTNR3	0
1382	rbflvrd	1515	ribflv	328	ARBTNR3	1
261	arbtn[p]	98	h	329	ARBTNtex	0
261	arbtn[p]	259	arbtn[e]	329	ARBTNtex	1
437	h[p]	98	h	329	ARBTNtex	0
437	h[p]	259	arbtn[e]	329	ARBTNtex	0
262	arbtn-fe3[e]	98	h	330	ARBTNtonex	0
262	arbtn-fe3[e]	258	arbtn-fe3[p]	330	ARBTNtonex	1
437	h[p]	98	h	330	ARBTNtonex	0
437	h[p]	258	arbtn-fe3[p]	330	ARBTNtonex	0
263	arbtn	98	h	331	ARBTNtpp	0
263	arbtn	261	arbtn[p]	331	ARBTNtpp	1
437	h[p]	98	h	331	ARBTNtpp	0
437	h[p]	261	arbtn[p]	331	ARBTNtpp	0
109	h2o	98	h	332	ARGabcpp	0
109	h2o	265	arg-L	332	ARGabcpp	0
109	h2o	281	adp	332	ARGabcpp	0
109	h2o	962	pi	332	ARGabcpp	0
135	atp	98	h	332	ARGabcpp	0
135	atp	265	arg-L	332	ARGabcpp	0
135	atp	281	adp	332	ARGabcpp	1

135	atp	962	pi	332	ARGabcpp	0
264	arg-L[p]	98	h	332	ARGabcpp	0
264	arg-L[p]	265	arg-L	332	ARGabcpp	1
264	arg-L[p]	281	adp	332	ARGabcpp	0
264	arg-L[p]	962	pi	332	ARGabcpp	0
197	agm	265	arg-L	333	ARGAGMt7pp	0
197	agm	1081	agm[p]	333	ARGAGMt7pp	1
264	arg-L[p]	265	arg-L	333	ARGAGMt7pp	1
264	arg-L[p]	1081	agm[p]	333	ARGAGMt7pp	0
98	h	197	agm	334	ARGDC	0
98	h	692	co2	334	ARGDC	0
265	arg-L	197	agm	334	ARGDC	1
265	arg-L	692	co2	334	ARGDC	0
264	arg-L[p]	333	co2[p]	335	ARGDCpp	0
264	arg-L[p]	1081	agm[p]	335	ARGDCpp	1
437	h[p]	333	co2[p]	335	ARGDCpp	0
437	h[p]	1081	agm[p]	335	ARGDCpp	0
264	arg-L[p]	265	arg-L	336	ARGORNt7pp	1
264	arg-L[p]	1167	orn[p]	336	ARGORNt7pp	0
963	orn	265	arg-L	336	ARGORNt7pp	0
963	orn	1167	orn[p]	336	ARGORNt7pp	1
266	argsuc	265	arg-L	337	ARGSL	1
266	argsuc	540	fum	337	ARGSL	1
135	atp	98	h	338	ARGSS	0
135	atp	177	amp	338	ARGSS	1
135	atp	266	argsuc	338	ARGSS	0
135	atp	1192	ppi	338	ARGSS	0
189	asp-L	98	h	338	ARGSS	0
189	asp-L	177	amp	338	ARGSS	0
189	asp-L	266	argsuc	338	ARGSS	1
189	asp-L	1192	ppi	338	ARGSS	0
1165	citr-L	98	h	338	ARGSS	0
1165	citr-L	177	amp	338	ARGSS	0
1165	citr-L	266	argsuc	338	ARGSS	1
1165	citr-L	1192	ppi	338	ARGSS	0
265	arg-L	98	h	339	ARGt3pp	0
265	arg-L	264	arg-L[p]	339	ARGt3pp	1
437	h[p]	98	h	339	ARGt3pp	0
437	h[p]	264	arg-L[p]	339	ARGt3pp	0
267	arg-L[e]	264	arg-L[p]	340	ARGtex	1
135	atp	177	amp	341	ARGTRS	1
135	atp	1192	ppi	341	ARGTRS	0
135	atp	1583	argtrna	341	ARGTRS	0
265	arg-L	177	amp	341	ARGTRS	0
265	arg-L	1192	ppi	341	ARGTRS	0
265	arg-L	1583	argtrna	341	ARGTRS	1
1625	trnaarg	177	amp	341	ARGTRS	0
1625	trnaarg	1192	ppi	341	ARGTRS	0
1625	trnaarg	1583	argtrna	341	ARGTRS	1
268	aspsa	98	h	342	ASAD	0
268	aspsa	871	nadph	342	ASAD	0
268	aspsa	1087	4pasp	342	ASAD	1
459	nadp	98	h	342	ASAD	0
459	nadp	871	nadph	342	ASAD	1
459	nadp	1087	4pasp	342	ASAD	0
962	pi	98	h	342	ASAD	0

962	pi	871	nadph	342	ASAD	0
962	pi	1087	4pasp	342	ASAD	0
109	h2o	98	h	343	ASCBPL	0
109	h2o	743	3dhgulnp	343	ASCBPL	0
269	ascb6p	98	h	343	ASCBPL	0
269	ascb6p	743	3dhgulnp	343	ASCBPL	1
270	ascb-L[p]	269	ascb6p	344	ASCBptspp	1
270	ascb-L[p]	1148	pyr	344	ASCBptspp	0
959	pep	269	ascb6p	344	ASCBptspp	0
959	pep	1148	pyr	344	ASCBptspp	1
271	ascb-L[e]	270	ascb-L[p]	345	ASCBtex	1
109	h2o	98	h	346	ASNabcpp	0
109	h2o	273	asn-L	346	ASNabcpp	0
109	h2o	281	adp	346	ASNabcpp	0
109	h2o	962	pi	346	ASNabcpp	0
135	atp	98	h	346	ASNabcpp	0
135	atp	273	asn-L	346	ASNabcpp	0
135	atp	281	adp	346	ASNabcpp	1
135	atp	962	pi	346	ASNabcpp	0
272	asn-L[p]	98	h	346	ASNabcpp	0
272	asn-L[p]	273	asn-L	346	ASNabcpp	1
272	asn-L[p]	281	adp	346	ASNabcpp	0
272	asn-L[p]	962	pi	346	ASNabcpp	0
109	h2o	189	asp-L	347	ASNN	0
109	h2o	1160	nh4	347	ASNN	0
273	asn-L	189	asp-L	347	ASNN	1
273	asn-L	1160	nh4	347	ASNN	0
272	asn-L[p]	277	asp-L[p]	348	ASNNpp	1
272	asn-L[p]	859	nh4[p]	348	ASNNpp	0
684	h2o[p]	277	asp-L[p]	348	ASNNpp	0
684	h2o[p]	859	nh4[p]	348	ASNNpp	0
109	h2o	98	h	349	ASNS1	0
109	h2o	177	amp	349	ASNS1	0
109	h2o	273	asn-L	349	ASNS1	0
109	h2o	624	glu-L	349	ASNS1	0
109	h2o	1192	ppi	349	ASNS1	0
135	atp	98	h	349	ASNS1	0
135	atp	177	amp	349	ASNS1	1
135	atp	273	asn-L	349	ASNS1	0
135	atp	624	glu-L	349	ASNS1	0
135	atp	1192	ppi	349	ASNS1	0
189	asp-L	98	h	349	ASNS1	0
189	asp-L	177	amp	349	ASNS1	0
189	asp-L	273	asn-L	349	ASNS1	1
189	asp-L	624	glu-L	349	ASNS1	0
189	asp-L	1192	ppi	349	ASNS1	0
625	gln-L	98	h	349	ASNS1	0
625	gln-L	177	amp	349	ASNS1	0
625	gln-L	273	asn-L	349	ASNS1	0
625	gln-L	624	glu-L	349	ASNS1	1
625	gln-L	1192	ppi	349	ASNS1	0
135	atp	98	h	350	ASNS2	0
135	atp	177	amp	350	ASNS2	1
135	atp	273	asn-L	350	ASNS2	0
135	atp	1192	ppi	350	ASNS2	0
189	asp-L	98	h	350	ASNS2	0

189	asp-L	177	amp	350	ASNS2	0
189	asp-L	273	asn-L	350	ASNS2	1
189	asp-L	1192	ppi	350	ASNS2	0
1160	nh4	98	h	350	ASNS2	0
1160	nh4	177	amp	350	ASNS2	0
1160	nh4	273	asn-L	350	ASNS2	0
1160	nh4	1192	ppi	350	ASNS2	0
272	asn-L[p]	98	h	351	ASNt2rpp	0
272	asn-L[p]	273	asn-L	351	ASNt2rpp	1
437	h[p]	98	h	351	ASNt2rpp	0
437	h[p]	273	asn-L	351	ASNt2rpp	0
274	asn-L[e]	272	asn-L[p]	352	ASNtex	1
135	atp	177	amp	353	ASNTRS	1
135	atp	1192	ppi	353	ASNTRS	0
135	atp	1584	asntrna	353	ASNTRS	0
273	asn-L	177	amp	353	ASNTRS	0
273	asn-L	1192	ppi	353	ASNTRS	0
273	asn-L	1584	asntrna	353	ASNTRS	1
1626	trnaasn	177	amp	353	ASNTRS	0
1626	trnaasn	1192	ppi	353	ASNTRS	0
1626	trnaasn	1584	asntrna	353	ASNTRS	1
109	h2o	98	h	354	ASO3t8pp	0
109	h2o	281	adp	354	ASO3t8pp	0
109	h2o	962	pi	354	ASO3t8pp	0
109	h2o	1088	aso3[p]	354	ASO3t8pp	0
135	atp	98	h	354	ASO3t8pp	0
135	atp	281	adp	354	ASO3t8pp	1
135	atp	962	pi	354	ASO3t8pp	0
135	atp	1088	aso3[p]	354	ASO3t8pp	0
275	aso3	98	h	354	ASO3t8pp	0
275	aso3	281	adp	354	ASO3t8pp	0
275	aso3	962	pi	354	ASO3t8pp	0
275	aso3	1088	aso3[p]	354	ASO3t8pp	1
276	aso3[e]	1088	aso3[p]	355	ASO3tex	1
98	h	692	co2	356	ASP1DC	0
98	h	922	ala-B	356	ASP1DC	0
189	asp-L	692	co2	356	ASP1DC	0
189	asp-L	922	ala-B	356	ASP1DC	1
109	h2o	98	h	357	ASPabcpp	0
109	h2o	189	asp-L	357	ASPabcpp	0
109	h2o	281	adp	357	ASPabcpp	0
109	h2o	962	pi	357	ASPabcpp	0
135	atp	98	h	357	ASPabcpp	0
135	atp	189	asp-L	357	ASPabcpp	0
135	atp	281	adp	357	ASPabcpp	1
135	atp	962	pi	357	ASPabcpp	0
277	asp-L[p]	98	h	357	ASPabcpp	0
277	asp-L[p]	189	asp-L	357	ASPabcpp	1
277	asp-L[p]	281	adp	357	ASPabcpp	0
277	asp-L[p]	962	pi	357	ASPabcpp	0
189	asp-L	98	h	358	ASPCT	0
189	asp-L	962	pi	358	ASPCT	0
189	asp-L	1102	cbasp	358	ASPCT	1
904	cbp	98	h	358	ASPCT	0
904	cbp	962	pi	358	ASPCT	0
904	cbp	1102	cbasp	358	ASPCT	1

135	atp	281	adp	359	ASPK	1
135	atp	1087	4pasp	359	ASPK	0
189	asp-L	281	adp	359	ASPK	0
189	asp-L	1087	4pasp	359	ASPK	1
189	asp-L	98	h	360	ASPO3	0
189	asp-L	1443	q8h2	360	ASPO3	0
189	asp-L	1513	iasp	360	ASPO3	1
1003	q8	98	h	360	ASPO3	0
1003	q8	1443	q8h2	360	ASPO3	1
1003	q8	1513	iasp	360	ASPO3	0
189	asp-L	98	h	361	ASPO4	0
189	asp-L	867	mql8	361	ASPO4	0
189	asp-L	1513	iasp	361	ASPO4	1
1282	mqn8	98	h	361	ASPO4	0
1282	mqn8	867	mql8	361	ASPO4	1
1282	mqn8	1513	iasp	361	ASPO4	0
189	asp-L	98	h	362	ASPO5	0
189	asp-L	1008	succ	362	ASPO5	0
189	asp-L	1513	iasp	362	ASPO5	1
540	fum	98	h	362	ASPO5	0
540	fum	1008	succ	362	ASPO5	1
540	fum	1513	iasp	362	ASPO5	0
189	asp-L	98	h	363	ASPO6	0
189	asp-L	292	h2o2	363	ASPO6	0
189	asp-L	1513	iasp	363	ASPO6	1
928	o2	98	h	363	ASPO6	0
928	o2	292	h2o2	363	ASPO6	0
928	o2	1513	iasp	363	ASPO6	0
189	asp-L	540	fum	364	ASPT	1
189	asp-L	1160	nh4	364	ASPT	0
277	asp-L[p]	98	h	365	ASPt2_2pp	0
277	asp-L[p]	189	asp-L	365	ASPt2_2pp	1
437	h[p]	98	h	365	ASPt2_2pp	0
437	h[p]	189	asp-L	365	ASPt2_2pp	0
277	asp-L[p]	98	h	366	ASPt2_3pp	0
277	asp-L[p]	189	asp-L	366	ASPt2_3pp	1
437	h[p]	98	h	366	ASPt2_3pp	0
437	h[p]	189	asp-L	366	ASPt2_3pp	0
277	asp-L[p]	98	h	367	ASPt2pp	0
277	asp-L[p]	189	asp-L	367	ASPt2pp	1
437	h[p]	98	h	367	ASPt2pp	0
437	h[p]	189	asp-L	367	ASPt2pp	0
189	asp-L	624	glu-L	368	ASPTA	0
189	asp-L	1318	oaa	368	ASPTA	1
213	akg	624	glu-L	368	ASPTA	1
213	akg	1318	oaa	368	ASPTA	0
278	asp-L[e]	277	asp-L[p]	369	ASPtex	1
135	atp	177	amp	370	ASPTRS	1
135	atp	1192	ppi	370	ASPTRS	0
135	atp	1585	asptrna	370	ASPTRS	0
189	asp-L	177	amp	370	ASPTRS	0
189	asp-L	1192	ppi	370	ASPTRS	0
189	asp-L	1585	asptrna	370	ASPTRS	1
1627	trnaasp	177	amp	370	ASPTRS	0
1627	trnaasp	1192	ppi	370	ASPTRS	0
1627	trnaasp	1585	asptrna	370	ASPTRS	1

279	aso4	109	h2o	371	ASR	0
279	aso4	275	aso3	371	ASR	1
279	aso4	669	gthox	371	ASR	0
751	gthrd	109	h2o	371	ASR	0
751	gthrd	275	aso3	371	ASR	0
751	gthrd	669	gthox	371	ASR	1
265	arg-L	98	h	372	AST	0
265	arg-L	927	coa	372	AST	0
265	arg-L	1644	sucarg	372	AST	1
838	succoa	98	h	372	AST	0
838	succoa	927	coa	372	AST	1
838	succoa	1644	sucarg	372	AST	1
280	athr-L	98	h	373	ATHRDHr	0
280	athr-L	251	2aobut	373	ATHRDHr	1
280	athr-L	871	nadph	373	ATHRDHr	0
459	nadp	98	h	373	ATHRDHr	0
459	nadp	251	2aobut	373	ATHRDHr	0
459	nadp	871	nadph	373	ATHRDHr	1
98	h	1160	nh4	374	ATPHs	0
98	h	1249	itp	374	ATPHs	0
109	h2o	1160	nh4	374	ATPHs	0
109	h2o	1249	itp	374	ATPHs	0
135	atp	1160	nh4	374	ATPHs	0
135	atp	1249	itp	374	ATPHs	1
109	h2o	98	h	375	ATPM	0
109	h2o	281	adp	375	ATPM	0
109	h2o	962	pi	375	ATPM	0
135	atp	98	h	375	ATPM	0
135	atp	281	adp	375	ATPM	1
135	atp	962	pi	375	ATPM	0
135	atp	1192	ppi	376	ATPPRT	0
135	atp	1497	prbatp	376	ATPPRT	1
1052	prpp	1192	ppi	376	ATPPRT	0
1052	prpp	1497	prbatp	376	ATPPRT	1
281	adp	98	h	377	ATPS4rpp	0
281	adp	109	h2o	377	ATPS4rpp	0
281	adp	135	atp	377	ATPS4rpp	1
437	h[p]	98	h	377	ATPS4rpp	0
437	h[p]	109	h2o	377	ATPS4rpp	0
437	h[p]	135	atp	377	ATPS4rpp	0
962	pi	98	h	377	ATPS4rpp	0
962	pi	109	h2o	377	ATPS4rpp	0
962	pi	135	atp	377	ATPS4rpp	0
282	ala-B[p]	98	h	378	BALAt2pp	0
282	ala-B[p]	922	ala-B	378	BALAt2pp	1
437	h[p]	98	h	378	BALAt2pp	0
437	h[p]	922	ala-B	378	BALAt2pp	0
283	ala-B[e]	282	ala-B[p]	379	BALAtex	1
109	h2o	98	h	380	BETALDHx	0
109	h2o	870	nadh	380	BETALDHx	0
109	h2o	1250	glyb	380	BETALDHx	0
284	betald	98	h	380	BETALDHx	0
284	betald	870	nadh	380	BETALDHx	0
284	betald	1250	glyb	380	BETALDHx	1
856	nad	98	h	380	BETALDHx	0
856	nad	870	nadh	380	BETALDHx	1

856	nad	1250	glyb	380	BETALDHx	0
109	h2o	98	h	381	BETALDHy	0
109	h2o	871	nadph	381	BETALDHy	0
109	h2o	1250	glyb	381	BETALDHy	0
284	betald	98	h	381	BETALDHy	0
284	betald	871	nadph	381	BETALDHy	0
284	betald	1250	glyb	381	BETALDHy	1
459	nadp	98	h	381	BETALDHy	0
459	nadp	871	nadph	381	BETALDHy	1
459	nadp	1250	glyb	381	BETALDHy	0
109	h2o	177	amp	382	BPNT	0
109	h2o	962	pi	382	BPNT	0
1383	pap	177	amp	382	BPNT	1
1383	pap	962	pi	382	BPNT	0
98	h	109	h2o	383	BSORx	0
98	h	856	nad	383	BSORx	0
98	h	1251	btn	383	BSORx	0
285	btnso	109	h2o	383	BSORx	0
285	btnso	856	nad	383	BSORx	0
285	btnso	1251	btn	383	BSORx	1
870	nadh	109	h2o	383	BSORx	0
870	nadh	856	nad	383	BSORx	1
870	nadh	1251	btn	383	BSORx	0
98	h	109	h2o	384	BSORy	0
98	h	459	nadp	384	BSORy	0
98	h	1251	btn	384	BSORy	0
285	btnso	109	h2o	384	BSORy	0
285	btnso	459	nadp	384	BSORy	0
285	btnso	1251	btn	384	BSORy	1
871	nadph	109	h2o	384	BSORy	0
871	nadph	459	nadp	384	BSORy	1
871	nadph	1251	btn	384	BSORy	0
178	amet	98	h	385	BTS4	0
178	amet	116	dad-5	385	BTS4	1
178	amet	1251	btn	385	BTS4	0
178	amet	1433	met-L	385	BTS4	1
1384	dtbt	98	h	385	BTS4	0
1384	dtbt	116	dad-5	385	BTS4	0
1384	dtbt	1251	btn	385	BTS4	1
1384	dtbt	1433	met-L	385	BTS4	0
1628	s	98	h	385	BTS4	0
1628	s	116	dad-5	385	BTS4	0
1628	s	1251	btn	385	BTS4	0
1628	s	1433	met-L	385	BTS4	0
128	accoa	147	ac	386	BUTCT	1
128	accoa	157	btcoa	386	BUTCT	1
1090	but	147	ac	386	BUTCT	0
1090	but	157	btcoa	386	BUTCT	1
109	h2o	98	h	387	BUTSO3abcpp	0
109	h2o	281	adp	387	BUTSO3abcpp	0
109	h2o	513	butso3	387	BUTSO3abcpp	0
109	h2o	962	pi	387	BUTSO3abcpp	0
135	atp	98	h	387	BUTSO3abcpp	0
135	atp	281	adp	387	BUTSO3abcpp	1
135	atp	513	butso3	387	BUTSO3abcpp	0
135	atp	962	pi	387	BUTSO3abcpp	0

1089	butso3[p]	98	h	387	BUTSO3abcpp	0
1089	butso3[p]	281	adp	387	BUTSO3abcpp	0
1089	butso3[p]	513	butso3	387	BUTSO3abcpp	1
1089	butso3[p]	962	pi	387	BUTSO3abcpp	0
286	butso3[e]	1089	butso3[p]	388	BUTSO3tex	1
287	but[p]	98	h	389	BUTt2rpp	0
287	but[p]	1090	but	389	BUTt2rpp	1
437	h[p]	98	h	389	BUTt2rpp	0
437	h[p]	1090	but	389	BUTt2rpp	0
288	but[e]	287	but[p]	390	BUTtex	1
289	ca2	98	h	391	CA2t3pp	0
289	ca2	1091	ca2[p]	391	CA2t3pp	0
437	h[p]	98	h	391	CA2t3pp	0
437	h[p]	1091	ca2[p]	391	CA2t3pp	0
290	ca2[e]	1091	ca2[p]	392	CA2tex	0
291	15dap	98	h	393	CADVtpp	0
291	15dap	1100	15dap[p]	393	CADVtpp	1
291	15dap	1425	lys-L	393	CADVtpp	0
437	h[p]	98	h	393	CADVtpp	0
437	h[p]	1100	15dap[p]	393	CADVtpp	0
437	h[p]	1425	lys-L	393	CADVtpp	0
1143	lys-L[p]	98	h	393	CADVtpp	0
1143	lys-L[p]	1100	15dap[p]	393	CADVtpp	0
1143	lys-L[p]	1425	lys-L	393	CADVtpp	1
292	h2o2	109	h2o	394	CAT	0
292	h2o2	928	o2	394	CAT	0
289	ca2	1091	ca2[p]	395	CAt6pp	0
289	ca2	1344	na1	395	CAt6pp	0
941	na1[p]	1091	ca2[p]	395	CAt6pp	0
941	na1[p]	1344	na1	395	CAt6pp	0
98	h	181	adocbi	396	CBIAT	0
98	h	1491	pppi	396	CBIAT	0
135	atp	181	adocbi	396	CBIAT	1
135	atp	1491	pppi	396	CBIAT	0
1385	cbi	181	adocbi	396	CBIAT	1
1385	cbi	1491	pppi	396	CBIAT	0
293	cbi[e]	98	h	397	CBItonex	0
293	cbi[e]	1252	cbi[p]	397	CBItonex	1
437	h[p]	98	h	397	CBItonex	0
437	h[p]	1252	cbi[p]	397	CBItonex	0
109	h2o	98	h	398	CBIuabcpp	0
109	h2o	281	adp	398	CBIuabcpp	0
109	h2o	962	pi	398	CBIuabcpp	0
109	h2o	1385	cbi	398	CBIuabcpp	0
135	atp	98	h	398	CBIuabcpp	0
135	atp	281	adp	398	CBIuabcpp	1
135	atp	962	pi	398	CBIuabcpp	0
135	atp	1385	cbi	398	CBIuabcpp	0
1252	cbi[p]	98	h	398	CBIuabcpp	0
1252	cbi[p]	281	adp	398	CBIuabcpp	0
1252	cbi[p]	962	pi	398	CBIuabcpp	0
1252	cbi[p]	1385	cbi	398	CBIuabcpp	1
109	h2o	98	h	399	CBL1abcpp	0
109	h2o	281	adp	399	CBL1abcpp	0
109	h2o	962	pi	399	CBL1abcpp	0
109	h2o	1386	cbl1	399	CBL1abcpp	0

135	atp	98	h	399	CBL1abcpp	0
135	atp	281	adp	399	CBL1abcpp	1
135	atp	962	pi	399	CBL1abcpp	0
135	atp	1386	cbl1	399	CBL1abcpp	0
1253	cbl1[p]	98	h	399	CBL1abcpp	0
1253	cbl1[p]	281	adp	399	CBL1abcpp	0
1253	cbl1[p]	962	pi	399	CBL1abcpp	0
1253	cbl1[p]	1386	cbl1	399	CBL1abcpp	1
294	cbl1[e]	98	h	400	CBL1tonex	0
294	cbl1[e]	1253	cbl1[p]	400	CBL1tonex	1
437	h[p]	98	h	400	CBL1tonex	0
437	h[p]	1253	cbl1[p]	400	CBL1tonex	0
98	h	1092	adocbl	401	CBLAT	0
98	h	1491	pppi	401	CBLAT	0
135	atp	1092	adocbl	401	CBLAT	1
135	atp	1491	pppi	401	CBLAT	0
1386	cbl1	1092	adocbl	401	CBLAT	1
1386	cbl1	1491	pppi	401	CBLAT	0
135	atp	98	h	402	CBMKr	0
135	atp	281	adp	402	CBMKr	1
135	atp	904	cbp	402	CBMKr	0
692	co2	98	h	402	CBMKr	0
692	co2	281	adp	402	CBMKr	0
692	co2	904	cbp	402	CBMKr	1
1160	nh4	98	h	402	CBMKr	0
1160	nh4	281	adp	402	CBMKr	0
1160	nh4	904	cbp	402	CBMKr	0
109	h2o	98	h	403	CBPS	0
109	h2o	281	adp	403	CBPS	0
109	h2o	624	glu-L	403	CBPS	0
109	h2o	904	cbp	403	CBPS	0
109	h2o	962	pi	403	CBPS	0
135	atp	98	h	403	CBPS	0
135	atp	281	adp	403	CBPS	1
135	atp	624	glu-L	403	CBPS	0
135	atp	904	cbp	403	CBPS	0
135	atp	962	pi	403	CBPS	0
625	gln-L	98	h	403	CBPS	0
625	gln-L	281	adp	403	CBPS	0
625	gln-L	624	glu-L	403	CBPS	1
625	gln-L	904	cbp	403	CBPS	0
625	gln-L	962	pi	403	CBPS	0
1560	hco3	98	h	403	CBPS	0
1560	hco3	281	adp	403	CBPS	0
1560	hco3	624	glu-L	403	CBPS	0
1560	hco3	904	cbp	403	CBPS	1
1560	hco3	962	pi	403	CBPS	0
109	h2o	98	h	404	CD2abcpp	0
109	h2o	281	adp	404	CD2abcpp	0
109	h2o	297	cd2[p]	404	CD2abcpp	0
109	h2o	962	pi	404	CD2abcpp	0
135	atp	98	h	404	CD2abcpp	0
135	atp	281	adp	404	CD2abcpp	1
135	atp	297	cd2[p]	404	CD2abcpp	0
135	atp	962	pi	404	CD2abcpp	0
295	cd2	98	h	404	CD2abcpp	0

295	cd2	281	adp	404	CD2abcpp	0
295	cd2	297	cd2[p]	404	CD2abcpp	1
295	cd2	962	pi	404	CD2abcpp	0
295	cd2	98	h	405	CD2t3pp	0
295	cd2	297	cd2[p]	405	CD2t3pp	1
437	h[p]	98	h	405	CD2t3pp	0
437	h[p]	297	cd2[p]	405	CD2t3pp	0
296	cd2[e]	297	cd2[p]	406	CD2tex	1
297	cd2[p]	295	cd2	407	CD2tpp	1
109	h2o	98	h	408	CDAPPA120	0
109	h2o	330	cmp	408	CDAPPA120	0
109	h2o	1452	pa120	408	CDAPPA120	0
298	cdpdddecg	98	h	408	CDAPPA120	0
298	cdpdddecg	330	cmp	408	CDAPPA120	1
298	cdpdddecg	1452	pa120	408	CDAPPA120	1
109	h2o	98	h	409	CDAPPA140	0
109	h2o	330	cmp	409	CDAPPA140	0
109	h2o	1454	pa140	409	CDAPPA140	0
299	cdpdtdecg	98	h	409	CDAPPA140	0
299	cdpdtdecg	330	cmp	409	CDAPPA140	1
299	cdpdtdecg	1454	pa140	409	CDAPPA140	1
109	h2o	98	h	410	CDAPPA141	0
109	h2o	330	cmp	410	CDAPPA141	0
109	h2o	1456	pa141	410	CDAPPA141	0
300	cdpdtdec7eg	98	h	410	CDAPPA141	0
300	cdpdtdec7eg	330	cmp	410	CDAPPA141	1
300	cdpdtdec7eg	1456	pa141	410	CDAPPA141	1
109	h2o	98	h	411	CDAPPA160	0
109	h2o	330	cmp	411	CDAPPA160	0
109	h2o	1458	pa160	411	CDAPPA160	0
301	cdpdhdecg	98	h	411	CDAPPA160	0
301	cdpdhdecg	330	cmp	411	CDAPPA160	1
301	cdpdhdecg	1458	pa160	411	CDAPPA160	1
109	h2o	98	h	412	CDAPPA161	0
109	h2o	330	cmp	412	CDAPPA161	0
109	h2o	1460	pa161	412	CDAPPA161	0
302	cdpdhdec9eg	98	h	412	CDAPPA161	0
302	cdpdhdec9eg	330	cmp	412	CDAPPA161	1
302	cdpdhdec9eg	1460	pa161	412	CDAPPA161	1
109	h2o	98	h	413	CDAPPA180	0
109	h2o	330	cmp	413	CDAPPA180	0
109	h2o	1462	pa180	413	CDAPPA180	0
303	cdpdodecg	98	h	413	CDAPPA180	0
303	cdpdodecg	330	cmp	413	CDAPPA180	1
303	cdpdodecg	1462	pa180	413	CDAPPA180	1
109	h2o	98	h	414	CDAPPA181	0
109	h2o	330	cmp	414	CDAPPA181	0
109	h2o	1464	pa181	414	CDAPPA181	0
304	cdpdodec11eg	98	h	414	CDAPPA181	0
304	cdpdodec11eg	330	cmp	414	CDAPPA181	1
304	cdpdodec11eg	1464	pa181	414	CDAPPA181	1
135	atp	98	h	415	CDPMEK	0
135	atp	281	adp	415	CDPMEK	1
135	atp	814	2p4c2me	415	CDPMEK	0
305	4c2me	98	h	415	CDPMEK	0
305	4c2me	281	adp	415	CDPMEK	0

305	4c2me	814	2p4c2me	415	CDPMEK	1
178	amet	98	h	416	CFAS160E	0
178	amet	209	ahcys	416	CFAS160E	1
178	amet	1586	cpe160	416	CFAS160E	1
1387	pe161	98	h	416	CFAS160E	0
1387	pe161	209	ahcys	416	CFAS160E	0
1387	pe161	1586	cpe160	416	CFAS160E	1
178	amet	98	h	417	CFAS160G	0
178	amet	209	ahcys	417	CFAS160G	1
178	amet	1587	cpg160	417	CFAS160G	1
1340	pg161	98	h	417	CFAS160G	0
1340	pg161	209	ahcys	417	CFAS160G	0
1340	pg161	1587	cpg160	417	CFAS160G	1
178	amet	98	h	418	CFAS180E	0
178	amet	209	ahcys	418	CFAS180E	1
178	amet	1588	cpe180	418	CFAS180E	1
1388	pe181	98	h	418	CFAS180E	0
1388	pe181	209	ahcys	418	CFAS180E	0
1388	pe181	1588	cpe180	418	CFAS180E	1
178	amet	98	h	419	CFAS180G	0
178	amet	209	ahcys	419	CFAS180G	1
178	amet	1589	cpg180	419	CFAS180G	1
1342	pg181	98	h	419	CFAS180G	0
1342	pg181	209	ahcys	419	CFAS180G	0
1342	pg181	1589	cpg180	419	CFAS180G	1
109	h2o	98	h	420	CGLYabcpp	0
109	h2o	246	cgly	420	CGLYabcpp	0
109	h2o	281	adp	420	CGLYabcpp	0
109	h2o	962	pi	420	CGLYabcpp	0
135	atp	98	h	420	CGLYabcpp	0
135	atp	246	cgly	420	CGLYabcpp	0
135	atp	281	adp	420	CGLYabcpp	1
135	atp	962	pi	420	CGLYabcpp	0
1093	cgly[p]	98	h	420	CGLYabcpp	0
1093	cgly[p]	246	cgly	420	CGLYabcpp	1
1093	cgly[p]	281	adp	420	CGLYabcpp	0
1093	cgly[p]	962	pi	420	CGLYabcpp	0
306	cgly[e]	1093	cgly[p]	421	CGLYtex	1
109	h2o	98	h	422	CHLabcpp	0
109	h2o	281	adp	422	CHLabcpp	0
109	h2o	962	pi	422	CHLabcpp	0
109	h2o	1254	chol	422	CHLabcpp	0
135	atp	98	h	422	CHLabcpp	0
135	atp	281	adp	422	CHLabcpp	1
135	atp	962	pi	422	CHLabcpp	0
135	atp	1254	chol	422	CHLabcpp	0
307	chol[p]	98	h	422	CHLabcpp	0
307	chol[p]	281	adp	422	CHLabcpp	0
307	chol[p]	962	pi	422	CHLabcpp	0
307	chol[p]	1254	chol	422	CHLabcpp	1
307	chol[p]	98	h	423	CHLt2pp	0
307	chol[p]	1254	chol	423	CHLt2pp	1
437	h[p]	98	h	423	CHLt2pp	0
437	h[p]	1254	chol	423	CHLt2pp	0
308	chol[e]	307	chol[p]	424	CHLtex	1
173	chor	1255	pphn	425	CHORM	1

309	3psme	173	chor	426	CHORS	1
309	3psme	962	pi	426	CHORS	0
173	chor	689	4hbz	427	CHRPL	1
173	chor	1148	pyr	427	CHRPL	1
98	h	418	cenchddd	428	CINNDO	0
98	h	856	nad	428	CINNDO	0
310	cinnm	418	cenchddd	428	CINNDO	1
310	cinnm	856	nad	428	CINNDO	0
870	nadh	418	cenchddd	428	CINNDO	0
870	nadh	856	nad	428	CINNDO	1
928	o2	418	cenchddd	428	CINNDO	0
928	o2	856	nad	428	CINNDO	0
164	cit	147	ac	429	CITL	1
164	cit	1318	oaa	429	CITL	1
311	cit[p]	164	cit	430	CITt7pp	1
311	cit[p]	1199	succ[p]	430	CITt7pp	0
1008	succ	164	cit	430	CITt7pp	0
1008	succ	1199	succ[p]	430	CITt7pp	1
312	cit[e]	311	cit[p]	431	CITtex	1
313	lipa_cold[p]	1256	lipa_cold[e]	432	CLIPAtex	1
314	clpn120[p]	321	pg120[p]	433	CLPNH120pp	1
314	clpn120[p]	437	h[p]	433	CLPNH120pp	0
314	clpn120[p]	1453	pa120[p]	433	CLPNH120pp	1
684	h2o[p]	321	pg120[p]	433	CLPNH120pp	0
684	h2o[p]	437	h[p]	433	CLPNH120pp	0
684	h2o[p]	1453	pa120[p]	433	CLPNH120pp	0
315	clpn140[p]	322	pg140[p]	434	CLPNH140pp	1
315	clpn140[p]	437	h[p]	434	CLPNH140pp	0
315	clpn140[p]	1455	pa140[p]	434	CLPNH140pp	1
684	h2o[p]	322	pg140[p]	434	CLPNH140pp	0
684	h2o[p]	437	h[p]	434	CLPNH140pp	0
684	h2o[p]	1455	pa140[p]	434	CLPNH140pp	0
316	clpn141[p]	323	pg141[p]	435	CLPNH141pp	1
316	clpn141[p]	437	h[p]	435	CLPNH141pp	0
316	clpn141[p]	1457	pa141[p]	435	CLPNH141pp	1
684	h2o[p]	323	pg141[p]	435	CLPNH141pp	0
684	h2o[p]	437	h[p]	435	CLPNH141pp	0
684	h2o[p]	1457	pa141[p]	435	CLPNH141pp	0
317	clpn160[p]	324	pg160[p]	436	CLPNH160pp	1
317	clpn160[p]	437	h[p]	436	CLPNH160pp	0
317	clpn160[p]	1459	pa160[p]	436	CLPNH160pp	1
684	h2o[p]	324	pg160[p]	436	CLPNH160pp	0
684	h2o[p]	437	h[p]	436	CLPNH160pp	0
684	h2o[p]	1459	pa160[p]	436	CLPNH160pp	0
318	clpn161[p]	325	pg161[p]	437	CLPNH161pp	1
318	clpn161[p]	437	h[p]	437	CLPNH161pp	0
318	clpn161[p]	1461	pa161[p]	437	CLPNH161pp	1
684	h2o[p]	325	pg161[p]	437	CLPNH161pp	0
684	h2o[p]	437	h[p]	437	CLPNH161pp	0
684	h2o[p]	1461	pa161[p]	437	CLPNH161pp	0
319	clpn180[p]	326	pg180[p]	438	CLPNH180pp	1
319	clpn180[p]	437	h[p]	438	CLPNH180pp	0
319	clpn180[p]	1463	pa180[p]	438	CLPNH180pp	1
684	h2o[p]	326	pg180[p]	438	CLPNH180pp	0
684	h2o[p]	437	h[p]	438	CLPNH180pp	0
684	h2o[p]	1463	pa180[p]	438	CLPNH180pp	0

320	clpn181[p]	327	pg181[p]	439	CLPNH181pp	1
320	clpn181[p]	437	h[p]	439	CLPNH181pp	0
320	clpn181[p]	1465	pa181[p]	439	CLPNH181pp	1
684	h2o[p]	327	pg181[p]	439	CLPNH181pp	0
684	h2o[p]	437	h[p]	439	CLPNH181pp	0
684	h2o[p]	1465	pa181[p]	439	CLPNH181pp	0
321	pg120[p]	314	clpn120[p]	440	CLPNS120pp	1
321	pg120[p]	1119	glyc[p]	440	CLPNS120pp	1
322	pg140[p]	315	clpn140[p]	441	CLPNS140pp	1
322	pg140[p]	1119	glyc[p]	441	CLPNS140pp	1
323	pg141[p]	316	clpn141[p]	442	CLPNS141pp	1
323	pg141[p]	1119	glyc[p]	442	CLPNS141pp	1
324	pg160[p]	317	clpn160[p]	443	CLPNS160pp	1
324	pg160[p]	1119	glyc[p]	443	CLPNS160pp	1
325	pg161[p]	318	clpn161[p]	444	CLPNS161pp	1
325	pg161[p]	1119	glyc[p]	444	CLPNS161pp	1
326	pg180[p]	319	clpn180[p]	445	CLPNS180pp	1
326	pg180[p]	1119	glyc[p]	445	CLPNS180pp	1
327	pg181[p]	320	clpn181[p]	446	CLPNS181pp	1
327	pg181[p]	1119	glyc[p]	446	CLPNS181pp	1
98	h	437	h[p]	447	CLt3_2pp	0
98	h	1257	cl	447	CLt3_2pp	0
328	cl[p]	437	h[p]	447	CLt3_2pp	0
328	cl[p]	1257	cl	447	CLt3_2pp	0
329	cl[e]	328	cl[p]	448	CLtex	0
109	h2o	350	csn	449	CMPN	0
109	h2o	983	r5p	449	CMPN	0
330	cmp	350	csn	449	CMPN	1
330	cmp	983	r5p	449	CMPN	1
331	cmp[e]	881	cmp[p]	450	CMPtex	1
332	co2[e]	333	co2[p]	451	CO2tex	1
333	co2[p]	692	co2	452	CO2tpp	1
109	h2o	98	h	453	COBALT2abcpp	0
109	h2o	281	adp	453	COBALT2abcpp	0
109	h2o	336	cobalt2[p]	453	COBALT2abcpp	0
109	h2o	962	pi	453	COBALT2abcpp	0
135	atp	98	h	453	COBALT2abcpp	0
135	atp	281	adp	453	COBALT2abcpp	1
135	atp	336	cobalt2[p]	453	COBALT2abcpp	0
135	atp	962	pi	453	COBALT2abcpp	0
334	cobalt2	98	h	453	COBALT2abcpp	0
334	cobalt2	281	adp	453	COBALT2abcpp	0
334	cobalt2	336	cobalt2[p]	453	COBALT2abcpp	0
334	cobalt2	962	pi	453	COBALT2abcpp	0
334	cobalt2	98	h	454	COBALT2t3pp	0
334	cobalt2	336	cobalt2[p]	454	COBALT2t3pp	0
437	h[p]	98	h	454	COBALT2t3pp	0
437	h[p]	336	cobalt2[p]	454	COBALT2t3pp	0
335	cobalt2[e]	336	cobalt2[p]	455	COBALT2tex	0
336	cobalt2[p]	334	cobalt2	456	COBALT2tpp	0
109	h2o	98	h	457	COLIPAabcpp	0
109	h2o	281	adp	457	COLIPAabcpp	0
109	h2o	337	colipa[p]	457	COLIPAabcpp	0
109	h2o	962	pi	457	COLIPAabcpp	0
135	atp	98	h	457	COLIPAabcpp	0
135	atp	281	adp	457	COLIPAabcpp	1

135	atp	337	colipa[p]	457	COLIPAabcpp	0
135	atp	962	pi	457	COLIPAabcpp	0
1389	colipa	98	h	457	COLIPAabcpp	0
1389	colipa	281	adp	457	COLIPAabcpp	0
1389	colipa	337	colipa[p]	457	COLIPAabcpp	1
1389	colipa	962	pi	457	COLIPAabcpp	0
337	colipa[p]	752	colipa[e]	458	COLIPAtex	1
109	h2o	98	h	459	CPGNabcpp	0
109	h2o	281	adp	459	CPGNabcpp	0
109	h2o	339	cpgn	459	CPGNabcpp	0
109	h2o	962	pi	459	CPGNabcpp	0
135	atp	98	h	459	CPGNabcpp	0
135	atp	281	adp	459	CPGNabcpp	1
135	atp	339	cpgn	459	CPGNabcpp	0
135	atp	962	pi	459	CPGNabcpp	0
1258	cpgn[p]	98	h	459	CPGNabcpp	0
1258	cpgn[p]	281	adp	459	CPGNabcpp	0
1258	cpgn[p]	339	cpgn	459	CPGNabcpp	1
1258	cpgn[p]	962	pi	459	CPGNabcpp	0
338	cpgn-un[e]	340	cpgn[e]	460	CPGNexs	1
519	fe3[e]	340	cpgn[e]	460	CPGNexs	0
339	cpgn	98	h	461	CPGNR1	0
339	cpgn	158	fad	461	CPGNR1	0
339	cpgn	342	cpgn-un	461	CPGNR1	1
339	cpgn	509	fe2	461	CPGNR1	0
520	fadh2	98	h	461	CPGNR1	0
520	fadh2	158	fad	461	CPGNR1	1
520	fadh2	342	cpgn-un	461	CPGNR1	0
520	fadh2	509	fe2	461	CPGNR1	0
339	cpgn	98	h	462	CPGNR2	0
339	cpgn	342	cpgn-un	462	CPGNR2	1
339	cpgn	509	fe2	462	CPGNR2	0
339	cpgn	538	fmn	462	CPGNR2	0
511	fmnh2	98	h	462	CPGNR2	0
511	fmnh2	342	cpgn-un	462	CPGNR2	0
511	fmnh2	509	fe2	462	CPGNR2	0
511	fmnh2	538	fmn	462	CPGNR2	1
339	cpgn	98	h	463	CPGNR3	0
339	cpgn	342	cpgn-un	463	CPGNR3	1
339	cpgn	509	fe2	463	CPGNR3	0
339	cpgn	1515	ribflv	463	CPGNR3	0
1382	rbflvrd	98	h	463	CPGNR3	0
1382	rbflvrd	342	cpgn-un	463	CPGNR3	0
1382	rbflvrd	509	fe2	463	CPGNR3	0
1382	rbflvrd	1515	ribflv	463	CPGNR3	1
340	cpgn[e]	98	h	464	CPGNtonex	0
340	cpgn[e]	1258	cpgn[p]	464	CPGNtonex	1
437	h[p]	98	h	464	CPGNtonex	0
437	h[p]	1258	cpgn[p]	464	CPGNtonex	0
341	cpgn-un[p]	98	h	465	CPGNUtex	0
341	cpgn-un[p]	338	cpgn-un[e]	465	CPGNUtex	1
437	h[p]	98	h	465	CPGNUtex	0
437	h[p]	338	cpgn-un[e]	465	CPGNUtex	0
342	cpgn-un	98	h	466	CPGNUtpp	0
342	cpgn-un	341	cpgn-un[p]	466	CPGNUtpp	1
437	h[p]	98	h	466	CPGNUtpp	0

437	h[p]	341	cpgn-un[p]	466	CPGNUtpp	0
98	h	109	h2o	467	CPPPGO	0
98	h	692	co2	467	CPPPGO	0
98	h	1494	pppg9	467	CPPPGO	0
343	cpppg3	109	h2o	467	CPPPGO	0
343	cpppg3	692	co2	467	CPPPGO	1
343	cpppg3	1494	pppg9	467	CPPPGO	0
928	o2	109	h2o	467	CPPPGO	0
928	o2	692	co2	467	CPPPGO	0
928	o2	1494	pppg9	467	CPPPGO	0
178	amet	116	dad-5	468	CPPPGO2	1
178	amet	692	co2	468	CPPPGO2	0
178	amet	1433	met-L	468	CPPPGO2	1
178	amet	1494	pppg9	468	CPPPGO2	0
343	cpppg3	116	dad-5	468	CPPPGO2	0
343	cpppg3	692	co2	468	CPPPGO2	1
343	cpppg3	1433	met-L	468	CPPPGO2	0
343	cpppg3	1494	pppg9	468	CPPPGO2	0
109	h2o	98	h	469	CRNabcpp	0
109	h2o	281	adp	469	CRNabcpp	0
109	h2o	346	crn	469	CRNabcpp	0
109	h2o	962	pi	469	CRNabcpp	0
135	atp	98	h	469	CRNabcpp	0
135	atp	281	adp	469	CRNabcpp	1
135	atp	346	crn	469	CRNabcpp	0
135	atp	962	pi	469	CRNabcpp	0
348	crn[p]	98	h	469	CRNabcpp	0
348	crn[p]	281	adp	469	CRNabcpp	0
348	crn[p]	346	crn	469	CRNabcpp	1
348	crn[p]	962	pi	469	CRNabcpp	0
344	bbtcoa	345	crncoa	470	CRNBTCT	1
344	bbtcoa	1390	gbbtn	470	CRNBTCT	1
346	crn	345	crncoa	470	CRNBTCT	1
346	crn	1390	gbbtn	470	CRNBTCT	0
135	atp	281	adp	471	CRNCAL2	1
135	atp	345	crncoa	471	CRNCAL2	0
135	atp	962	pi	471	CRNCAL2	0
346	crn	281	adp	471	CRNCAL2	0
346	crn	345	crncoa	471	CRNCAL2	1
346	crn	962	pi	471	CRNCAL2	0
927	coa	281	adp	471	CRNCAL2	0
927	coa	345	crncoa	471	CRNCAL2	1
927	coa	962	pi	471	CRNCAL2	0
345	crncoa	1094	crnDcoa	472	CRNCAR	1
346	crn	345	crncoa	473	CRNCBCT	1
346	crn	1097	ctbt	473	CRNCBCT	0
1095	ctbtcoa	345	crncoa	473	CRNCBCT	1
1095	ctbtcoa	1097	ctbt	473	CRNCBCT	1
345	crncoa	109	h2o	474	CRNCDH	0
345	crncoa	1095	ctbtcoa	474	CRNCDH	1
109	h2o	98	h	475	CRNDabcpp	0
109	h2o	281	adp	475	CRNDabcpp	0
109	h2o	962	pi	475	CRNDabcpp	0
109	h2o	1096	crn-D	475	CRNDabcpp	0
135	atp	98	h	475	CRNDabcpp	0
135	atp	281	adp	475	CRNDabcpp	1

135	atp	962	pi	475	CRNDabcpp	0
135	atp	1096	crn-D	475	CRNDabcpp	0
347	crn-D[p]	98	h	475	CRNDabcpp	0
347	crn-D[p]	281	adp	475	CRNDabcpp	0
347	crn-D[p]	962	pi	475	CRNDabcpp	0
347	crn-D[p]	1096	crn-D	475	CRNDabcpp	1
135	atp	281	adp	476	CRNDCAL2	1
135	atp	962	pi	476	CRNDCAL2	0
135	atp	1094	crnDcoa	476	CRNDCAL2	0
927	coa	281	adp	476	CRNDCAL2	0
927	coa	962	pi	476	CRNDCAL2	0
927	coa	1094	crnDcoa	476	CRNDCAL2	1
1096	crn-D	281	adp	476	CRNDCAL2	0
1096	crn-D	962	pi	476	CRNDCAL2	0
1096	crn-D	1094	crnDcoa	476	CRNDCAL2	1
347	crn-D[p]	98	h	477	CRNDt2rpp	0
347	crn-D[p]	1096	crn-D	477	CRNDt2rpp	1
437	h[p]	98	h	477	CRNDt2rpp	0
437	h[p]	1096	crn-D	477	CRNDt2rpp	0
348	crn[p]	98	h	478	CRNt2rpp	0
348	crn[p]	346	crn	478	CRNt2rpp	1
437	h[p]	98	h	478	CRNt2rpp	0
437	h[p]	346	crn	478	CRNt2rpp	0
348	crn[p]	346	crn	479	CRNt7pp	1
348	crn[p]	1115	gbbtn[p]	479	CRNt7pp	0
1390	gbbtn	346	crn	479	CRNt7pp	0
1390	gbbtn	1115	gbbtn[p]	479	CRNt7pp	1
348	crn[p]	346	crn	480	CRNt8pp	1
348	crn[p]	347	crn-D[p]	480	CRNt8pp	0
1096	crn-D	346	crn	480	CRNt8pp	0
1096	crn-D	347	crn-D[p]	480	CRNt8pp	1
349	crn[e]	348	crn[p]	481	CRNtex	1
109	h2o	98	h	482	CS	0
109	h2o	164	cit	482	CS	0
109	h2o	927	coa	482	CS	0
128	accoa	98	h	482	CS	0
128	accoa	164	cit	482	CS	1
128	accoa	927	coa	482	CS	1
1318	oaa	98	h	482	CS	0
1318	oaa	164	cit	482	CS	1
1318	oaa	927	coa	482	CS	0
98	h	1160	nh4	483	CSND	0
98	h	1552	ura	483	CSND	0
109	h2o	1160	nh4	483	CSND	0
109	h2o	1552	ura	483	CSND	0
350	csn	1160	nh4	483	CSND	0
350	csn	1552	ura	483	CSND	1
351	csn[p]	98	h	484	CSNt2pp	0
351	csn[p]	350	csn	484	CSNt2pp	1
437	h[p]	98	h	484	CSNt2pp	0
437	h[p]	350	csn	484	CSNt2pp	0
352	csn[e]	351	csn[p]	485	CSNtex	1
109	h2o	98	h	486	CTBTabcpp	0
109	h2o	281	adp	486	CTBTabcpp	0
109	h2o	962	pi	486	CTBTabcpp	0
109	h2o	1097	ctbt	486	CTBTabcpp	0

135	atp	98	h	486	CTBTabcpp	0
135	atp	281	adp	486	CTBTabcpp	1
135	atp	962	pi	486	CTBTabcpp	0
135	atp	1097	ctbt	486	CTBTabcpp	0
353	ctbt[p]	98	h	486	CTBTabcpp	0
353	ctbt[p]	281	adp	486	CTBTabcpp	0
353	ctbt[p]	962	pi	486	CTBTabcpp	0
353	ctbt[p]	1097	ctbt	486	CTBTabcpp	1
135	atp	281	adp	487	CTBTCAL2	1
135	atp	962	pi	487	CTBTCAL2	0
135	atp	1095	ctbtcoa	487	CTBTCAL2	0
927	coa	281	adp	487	CTBTCAL2	0
927	coa	962	pi	487	CTBTCAL2	0
927	coa	1095	ctbtcoa	487	CTBTCAL2	1
1097	ctbt	281	adp	487	CTBTCAL2	0
1097	ctbt	962	pi	487	CTBTCAL2	0
1097	ctbt	1095	ctbtcoa	487	CTBTCAL2	1
353	ctbt[p]	98	h	488	CTBTt2rpp	0
353	ctbt[p]	1097	ctbt	488	CTBTt2rpp	1
437	h[p]	98	h	488	CTBTt2rpp	0
437	h[p]	1097	ctbt	488	CTBTt2rpp	0
109	h2o	98	h	489	CTPS2	0
109	h2o	281	adp	489	CTPS2	0
109	h2o	392	ctp	489	CTPS2	0
109	h2o	624	glu-L	489	CTPS2	0
109	h2o	962	pi	489	CTPS2	0
135	atp	98	h	489	CTPS2	0
135	atp	281	adp	489	CTPS2	1
135	atp	392	ctp	489	CTPS2	0
135	atp	624	glu-L	489	CTPS2	0
135	atp	962	pi	489	CTPS2	0
625	gln-L	98	h	489	CTPS2	0
625	gln-L	281	adp	489	CTPS2	0
625	gln-L	392	ctp	489	CTPS2	0
625	gln-L	624	glu-L	489	CTPS2	1
625	gln-L	962	pi	489	CTPS2	0
1447	utp	98	h	489	CTPS2	0
1447	utp	281	adp	489	CTPS2	0
1447	utp	392	ctp	489	CTPS2	1
1447	utp	624	glu-L	489	CTPS2	0
1447	utp	962	pi	489	CTPS2	0
109	h2o	98	h	490	CU1abcpp	0
109	h2o	281	adp	490	CU1abcpp	0
109	h2o	354	cu[p]	490	CU1abcpp	0
109	h2o	962	pi	490	CU1abcpp	0
135	atp	98	h	490	CU1abcpp	0
135	atp	281	adp	490	CU1abcpp	1
135	atp	354	cu[p]	490	CU1abcpp	0
135	atp	962	pi	490	CU1abcpp	0
357	cu	98	h	490	CU1abcpp	0
357	cu	281	adp	490	CU1abcpp	0
357	cu	354	cu[p]	490	CU1abcpp	0
357	cu	962	pi	490	CU1abcpp	0
354	cu[p]	356	cu2[p]	491	CU1Opp	0
354	cu[p]	684	h2o[p]	491	CU1Opp	0
437	h[p]	356	cu2[p]	491	CU1Opp	0

437	h[p]	684	h2o[p]	491	CU1Opp	0
903	o2[p]	356	cu2[p]	491	CU1Opp	0
903	o2[p]	684	h2o[p]	491	CU1Opp	0
109	h2o	98	h	492	CU2abcpp	0
109	h2o	281	adp	492	CU2abcpp	0
109	h2o	356	cu2[p]	492	CU2abcpp	0
109	h2o	962	pi	492	CU2abcpp	0
135	atp	98	h	492	CU2abcpp	0
135	atp	281	adp	492	CU2abcpp	1
135	atp	356	cu2[p]	492	CU2abcpp	0
135	atp	962	pi	492	CU2abcpp	0
1259	cu2	98	h	492	CU2abcpp	0
1259	cu2	281	adp	492	CU2abcpp	0
1259	cu2	356	cu2[p]	492	CU2abcpp	0
1259	cu2	962	pi	492	CU2abcpp	0
355	cu2[e]	356	cu2[p]	493	CU2tex	0
356	cu2[p]	1259	cu2	494	CU2tpp	0
357	cu	98	h	495	CUt3	0
357	cu	358	cu[e]	495	CUt3	0
711	h[e]	98	h	495	CUt3	0
711	h[e]	358	cu[e]	495	CUt3	0
358	cu[e]	354	cu[p]	496	CUtex	0
359	cyan	98	h	497	CYANST	0
359	cyan	1590	so3	497	CYANST	0
359	cyan	1655	tcynt	497	CYANST	1
1391	tsul	98	h	497	CYANST	0
1391	tsul	1590	so3	497	CYANST	0
1391	tsul	1655	tcynt	497	CYANST	0
360	cyan[p]	437	h[p]	498	CYANSTpp	0
360	cyan[p]	1196	so3[p]	498	CYANSTpp	0
360	cyan[p]	1204	tcynt[p]	498	CYANSTpp	1
1214	tsul[p]	437	h[p]	498	CYANSTpp	0
1214	tsul[p]	1196	so3[p]	498	CYANSTpp	0
1214	tsul[p]	1204	tcynt[p]	498	CYANSTpp	0
361	cyan[e]	360	cyan[p]	499	CYANtex	1
98	h	692	co2	500	CYNTAH	0
98	h	1160	nh4	500	CYNTAH	0
362	cynt	692	co2	500	CYNTAH	0
362	cynt	1160	nh4	500	CYNTAH	0
1560	hco3	692	co2	500	CYNTAH	1
1560	hco3	1160	nh4	500	CYNTAH	0
363	cynt[p]	98	h	501	CYNTt2pp	0
363	cynt[p]	362	cynt	501	CYNTt2pp	1
437	h[p]	98	h	501	CYNTt2pp	0
437	h[p]	362	cynt	501	CYNTt2pp	0
364	cynt[e]	363	cynt[p]	502	CYNTtex	1
109	h2o	98	h	503	CYSabc2pp	0
109	h2o	281	adp	503	CYSabc2pp	0
109	h2o	962	pi	503	CYSabc2pp	0
109	h2o	1099	cys-L[p]	503	CYSabc2pp	0
135	atp	98	h	503	CYSabc2pp	0
135	atp	281	adp	503	CYSabc2pp	1
135	atp	962	pi	503	CYSabc2pp	0
135	atp	1099	cys-L[p]	503	CYSabc2pp	0
366	cys-L	98	h	503	CYSabc2pp	0
366	cys-L	281	adp	503	CYSabc2pp	0

366	cys-L	962	pi	503	CYSabc2pp	0
366	cys-L	1099	cys-L[p]	503	CYSabc2pp	1
109	h2o	98	h	504	CYSabcpp	0
109	h2o	281	adp	504	CYSabcpp	0
109	h2o	366	cys-L	504	CYSabcpp	0
109	h2o	962	pi	504	CYSabcpp	0
135	atp	98	h	504	CYSabcpp	0
135	atp	281	adp	504	CYSabcpp	1
135	atp	366	cys-L	504	CYSabcpp	0
135	atp	962	pi	504	CYSabcpp	0
1099	cys-L[p]	98	h	504	CYSabcpp	0
1099	cys-L[p]	281	adp	504	CYSabcpp	0
1099	cys-L[p]	366	cys-L	504	CYSabcpp	1
1099	cys-L[p]	962	pi	504	CYSabcpp	0
109	h2o	98	h	505	CYSDabcpp	0
109	h2o	281	adp	505	CYSDabcpp	0
109	h2o	365	cys-D	505	CYSDabcpp	0
109	h2o	962	pi	505	CYSDabcpp	0
135	atp	98	h	505	CYSDabcpp	0
135	atp	281	adp	505	CYSDabcpp	1
135	atp	365	cys-D	505	CYSDabcpp	0
135	atp	962	pi	505	CYSDabcpp	0
1098	cys-D[p]	98	h	505	CYSDabcpp	0
1098	cys-D[p]	281	adp	505	CYSDabcpp	0
1098	cys-D[p]	365	cys-D	505	CYSDabcpp	1
1098	cys-D[p]	962	pi	505	CYSDabcpp	0
109	h2o	685	h2s	506	CYSDDS	0
109	h2o	1148	pyr	506	CYSDDS	0
109	h2o	1160	nh4	506	CYSDDS	0
365	cys-D	685	h2s	506	CYSDDS	0
365	cys-D	1148	pyr	506	CYSDDS	1
365	cys-D	1160	nh4	506	CYSDDS	0
109	h2o	685	h2s	507	CYSDS	0
109	h2o	1148	pyr	507	CYSDS	0
109	h2o	1160	nh4	507	CYSDS	0
366	cys-L	685	h2s	507	CYSDS	0
366	cys-L	1148	pyr	507	CYSDS	1
366	cys-L	1160	nh4	507	CYSDS	0
367	cys-D[e]	1098	cys-D[p]	508	CYSDtex	1
168	acser	98	h	509	CYSS	0
168	acser	147	ac	509	CYSS	1
168	acser	366	cys-L	509	CYSS	1
685	h2s	98	h	509	CYSS	0
685	h2s	147	ac	509	CYSS	0
685	h2s	366	cys-L	509	CYSS	0
98	h	222	ala-L	510	CYSSADS	0
98	h	1195	so2	510	CYSSADS	0
368	3sala	222	ala-L	510	CYSSADS	1
368	3sala	1195	so2	510	CYSSADS	0
369	cys-L[e]	1099	cys-L[p]	511	CYStex	1
109	h2o	693	hcys-L	512	CYSTL	0
109	h2o	1148	pyr	512	CYSTL	0
109	h2o	1160	nh4	512	CYSTL	0
370	cyst-L	693	hcys-L	512	CYSTL	1
370	cyst-L	1148	pyr	512	CYSTL	1
370	cyst-L	1160	nh4	512	CYSTL	0

366	cys-L	1099	cys-L[p]	513	CYStpp	1
135	atp	177	amp	514	CYSTRS	1
135	atp	1192	ppi	514	CYSTRS	0
135	atp	1591	cystrna	514	CYSTRS	0
366	cys-L	177	amp	514	CYSTRS	0
366	cys-L	1192	ppi	514	CYSTRS	0
366	cys-L	1591	cystrna	514	CYSTRS	1
1629	trnacys	177	amp	514	CYSTRS	0
1629	trnacys	1192	ppi	514	CYSTRS	0
1629	trnacys	1591	cystrna	514	CYSTRS	1
98	h	109	h2o	515	CYTBD2pp	0
98	h	437	h[p]	515	CYTBD2pp	0
98	h	1282	mqn8	515	CYTBD2pp	0
867	mql8	109	h2o	515	CYTBD2pp	0
867	mql8	437	h[p]	515	CYTBD2pp	0
867	mql8	1282	mqn8	515	CYTBD2pp	1
928	o2	109	h2o	515	CYTBD2pp	0
928	o2	437	h[p]	515	CYTBD2pp	0
928	o2	1282	mqn8	515	CYTBD2pp	0
98	h	109	h2o	516	CYTBDpp	0
98	h	437	h[p]	516	CYTBDpp	0
98	h	1003	q8	516	CYTBDpp	0
928	o2	109	h2o	516	CYTBDpp	0
928	o2	437	h[p]	516	CYTBDpp	0
928	o2	1003	q8	516	CYTBDpp	0
1443	q8h2	109	h2o	516	CYTBDpp	0
1443	q8h2	437	h[p]	516	CYTBDpp	0
1443	q8h2	1003	q8	516	CYTBDpp	1
98	h	109	h2o	517	CYTBO3_4pp	0
98	h	437	h[p]	517	CYTBO3_4pp	0
98	h	1003	q8	517	CYTBO3_4pp	0
928	o2	109	h2o	517	CYTBO3_4pp	0
928	o2	437	h[p]	517	CYTBO3_4pp	0
928	o2	1003	q8	517	CYTBO3_4pp	0
1443	q8h2	109	h2o	517	CYTBO3_4pp	0
1443	q8h2	437	h[p]	517	CYTBO3_4pp	0
1443	q8h2	1003	q8	517	CYTBO3_4pp	1
98	h	1160	nh4	518	CYTD	0
98	h	1512	uri	518	CYTD	0
109	h2o	1160	nh4	518	CYTD	0
109	h2o	1512	uri	518	CYTD	0
371	cytd	1160	nh4	518	CYTD	0
371	cytd	1512	uri	518	CYTD	1
109	h2o	350	csn	519	CYTDH	0
109	h2o	1361	rib-D	519	CYTDH	0
371	cytd	350	csn	519	CYTDH	1
371	cytd	1361	rib-D	519	CYTDH	1
371	cytd	98	h	520	CYTDK2	0
371	cytd	330	cmp	520	CYTDK2	1
371	cytd	798	gdp	520	CYTDK2	0
673	gtp	98	h	520	CYTDK2	0
673	gtp	330	cmp	520	CYTDK2	0
673	gtp	798	gdp	520	CYTDK2	1
372	cytd[p]	98	h	521	CYTDt2pp	0
372	cytd[p]	371	cytd	521	CYTDt2pp	1
437	h[p]	98	h	521	CYTDt2pp	0

437	h[p]	371	cytd	521	CYTDt2pp	0
372	cytd[p]	98	h	522	CYTDt2rpp	0
372	cytd[p]	371	cytd	522	CYTDt2rpp	1
437	h[p]	98	h	522	CYTDt2rpp	0
437	h[p]	371	cytd	522	CYTDt2rpp	0
373	cytd[e]	372	cytd[p]	523	CYTDtex	1
135	atp	281	adp	524	CYTK1	1
135	atp	980	cdp	524	CYTK1	0
330	cmp	281	adp	524	CYTK1	0
330	cmp	980	cdp	524	CYTK1	1
135	atp	281	adp	525	CYTK2	1
135	atp	1350	dcdp	525	CYTK2	0
879	dcmp	281	adp	525	CYTK2	0
879	dcmp	1350	dcdp	525	CYTK2	1
109	h2o	520	fadh2	526	DAAD	0
109	h2o	1148	pyr	526	DAAD	0
109	h2o	1160	nh4	526	DAAD	0
158	fad	520	fadh2	526	DAAD	1
158	fad	1148	pyr	526	DAAD	0
158	fad	1160	nh4	526	DAAD	0
219	ala-D	520	fadh2	526	DAAD	0
219	ala-D	1148	pyr	526	DAAD	1
219	ala-D	1160	nh4	526	DAAD	0
98	h	968	din	527	DADA	0
98	h	1160	nh4	527	DADA	0
109	h2o	968	din	527	DADA	0
109	h2o	1160	nh4	527	DADA	0
374	dad-2	968	din	527	DADA	1
374	dad-2	1160	nh4	527	DADA	0
135	atp	281	adp	528	DADK	1
135	atp	1348	dadp	528	DADK	0
884	damp	281	adp	528	DADK	0
884	damp	1348	dadp	528	DADK	1
375	dad-2[p]	98	h	529	DADNt2pp	0
375	dad-2[p]	374	dad-2	529	DADNt2pp	1
437	h[p]	98	h	529	DADNt2pp	0
437	h[p]	374	dad-2	529	DADNt2pp	0
376	dad-2[e]	375	dad-2[p]	530	DADNtex	1
135	atp	98	h	531	DAGK120	0
135	atp	281	adp	531	DAGK120	1
135	atp	1452	pa120	531	DAGK120	0
377	12dgr120	98	h	531	DAGK120	0
377	12dgr120	281	adp	531	DAGK120	0
377	12dgr120	1452	pa120	531	DAGK120	1
135	atp	98	h	532	DAGK140	0
135	atp	281	adp	532	DAGK140	1
135	atp	1454	pa140	532	DAGK140	0
378	12dgr140	98	h	532	DAGK140	0
378	12dgr140	281	adp	532	DAGK140	0
378	12dgr140	1454	pa140	532	DAGK140	1
135	atp	98	h	533	DAGK141	0
135	atp	281	adp	533	DAGK141	1
135	atp	1456	pa141	533	DAGK141	0
379	12dgr141	98	h	533	DAGK141	0
379	12dgr141	281	adp	533	DAGK141	0
379	12dgr141	1456	pa141	533	DAGK141	1

135	atp	98	h	534	DAGK160	0
135	atp	281	adp	534	DAGK160	1
135	atp	1458	pa160	534	DAGK160	0
380	12dgr160	98	h	534	DAGK160	0
380	12dgr160	281	adp	534	DAGK160	0
380	12dgr160	1458	pa160	534	DAGK160	1
135	atp	98	h	535	DAGK161	0
135	atp	281	adp	535	DAGK161	1
135	atp	1460	pa161	535	DAGK161	0
381	12dgr161	98	h	535	DAGK161	0
381	12dgr161	281	adp	535	DAGK161	0
381	12dgr161	1460	pa161	535	DAGK161	1
135	atp	98	h	536	DAGK180	0
135	atp	281	adp	536	DAGK180	1
135	atp	1462	pa180	536	DAGK180	0
382	12dgr180	98	h	536	DAGK180	0
382	12dgr180	281	adp	536	DAGK180	0
382	12dgr180	1462	pa180	536	DAGK180	1
135	atp	98	h	537	DAGK181	0
135	atp	281	adp	537	DAGK181	1
135	atp	1464	pa181	537	DAGK181	0
383	12dgr181	98	h	537	DAGK181	0
383	12dgr181	281	adp	537	DAGK181	0
383	12dgr181	1464	pa181	537	DAGK181	1
384	ala-D[p]	98	h	538	DALAt2pp	0
384	ala-D[p]	219	ala-D	538	DALAt2pp	1
437	h[p]	98	h	538	DALAt2pp	0
437	h[p]	219	ala-D	538	DALAt2pp	0
385	ala-D[e]	384	ala-D[p]	539	DALAtex	1
386	damp[e]	885	damp[p]	540	DAMPtex	1
109	h2o	98	h	541	DAPabcpp	0
109	h2o	281	adp	541	DAPabcpp	0
109	h2o	389	26dap-M	541	DAPabcpp	0
109	h2o	962	pi	541	DAPabcpp	0
135	atp	98	h	541	DAPabcpp	0
135	atp	281	adp	541	DAPabcpp	1
135	atp	389	26dap-M	541	DAPabcpp	0
135	atp	962	pi	541	DAPabcpp	0
387	26dap-M[p]	98	h	541	DAPabcpp	0
387	26dap-M[p]	281	adp	541	DAPabcpp	0
387	26dap-M[p]	389	26dap-M	541	DAPabcpp	1
387	26dap-M[p]	962	pi	541	DAPabcpp	0
109	h2o	1148	pyr	542	DAPAL	0
109	h2o	1160	nh4	542	DAPAL	0
388	23dappa	1148	pyr	542	DAPAL	1
388	23dappa	1160	nh4	542	DAPAL	0
98	h	692	co2	543	DAPDC	0
98	h	1425	lys-L	543	DAPDC	0
389	26dap-M	692	co2	543	DAPDC	0
389	26dap-M	1425	lys-L	543	DAPDC	1
390	26dap-LL	389	26dap-M	544	DAPE	1
391	15dap[e]	1100	15dap[p]	545	DAPtex	1
98	h	298	cdpdddecg	546	DASYN120	0
98	h	1192	ppi	546	DASYN120	0
392	ctp	298	cdpdddecg	546	DASYN120	1
392	ctp	1192	ppi	546	DASYN120	0

1452	pa120	298	cdpdddecg	546	DASYN120	1
1452	pa120	1192	ppi	546	DASYN120	0
98	h	299	cdpdtdecg	547	DASYN140	0
98	h	1192	ppi	547	DASYN140	0
392	ctp	299	cdpdtdecg	547	DASYN140	1
392	ctp	1192	ppi	547	DASYN140	0
1454	pa140	299	cdpdtdecg	547	DASYN140	1
1454	pa140	1192	ppi	547	DASYN140	0
98	h	300	cdpdtdec7eg	548	DASYN141	0
98	h	1192	ppi	548	DASYN141	0
392	ctp	300	cdpdtdec7eg	548	DASYN141	1
392	ctp	1192	ppi	548	DASYN141	0
1456	pa141	300	cdpdtdec7eg	548	DASYN141	1
1456	pa141	1192	ppi	548	DASYN141	0
98	h	301	cdpdhdecg	549	DASYN160	0
98	h	1192	ppi	549	DASYN160	0
392	ctp	301	cdpdhdecg	549	DASYN160	1
392	ctp	1192	ppi	549	DASYN160	0
1458	pa160	301	cdpdhdecg	549	DASYN160	1
1458	pa160	1192	ppi	549	DASYN160	0
98	h	302	cdpdhdec9eg	550	DASYN161	0
98	h	1192	ppi	550	DASYN161	0
392	ctp	302	cdpdhdec9eg	550	DASYN161	1
392	ctp	1192	ppi	550	DASYN161	0
1460	pa161	302	cdpdhdec9eg	550	DASYN161	1
1460	pa161	1192	ppi	550	DASYN161	0
98	h	303	cdpdodecg	551	DASYN180	0
98	h	1192	ppi	551	DASYN180	0
392	ctp	303	cdpdodecg	551	DASYN180	1
392	ctp	1192	ppi	551	DASYN180	0
1462	pa180	303	cdpdodecg	551	DASYN180	1
1462	pa180	1192	ppi	551	DASYN180	0
98	h	304	cdpdodec11eg	552	DASYN181	0
98	h	1192	ppi	552	DASYN181	0
392	ctp	304	cdpdodec11eg	552	DASYN181	1
392	ctp	1192	ppi	552	DASYN181	0
1464	pa181	304	cdpdodec11eg	552	DASYN181	1
1464	pa181	1192	ppi	552	DASYN181	0
98	h	890	ditp	553	DATPHs	0
98	h	1160	nh4	553	DATPHs	0
109	h2o	890	ditp	553	DATPHs	0
109	h2o	1160	nh4	553	DATPHs	0
393	datp	890	ditp	553	DATPHs	1
393	datp	1160	nh4	553	DATPHs	0
117	ru5p-D	98	h	554	DB4PS	0
117	ru5p-D	536	for	554	DB4PS	1
117	ru5p-D	1260	db4p	554	DB4PS	1
135	atp	98	h	555	DBTS	0
135	atp	281	adp	555	DBTS	1
135	atp	962	pi	555	DBTS	0
135	atp	1384	dtbt	555	DBTS	0
692	co2	98	h	555	DBTS	0
692	co2	281	adp	555	DBTS	0
692	co2	962	pi	555	DBTS	0
692	co2	1384	dtbt	555	DBTS	1
1559	dann	98	h	555	DBTS	0

1559	dann	281	adp	555	DBTS	0
1559	dann	962	pi	555	DBTS	0
1559	dann	1384	dtbt	555	DBTS	1
394	dca[e]	1101	dca[p]	556	DCAtex	1
395	dcmp[e]	880	dcmp[p]	557	DCMPtex	1
98	h	466	dutp	558	DCTPD	0
98	h	1160	nh4	558	DCTPD	0
109	h2o	466	dutp	558	DCTPD	0
109	h2o	1160	nh4	558	DCTPD	0
396	dctp	466	dutp	558	DCTPD	1
396	dctp	1160	nh4	558	DCTPD	0
98	h	463	duri	559	DCYTD	0
98	h	1160	nh4	559	DCYTD	0
109	h2o	463	duri	559	DCYTD	0
109	h2o	1160	nh4	559	DCYTD	0
397	dcyt	463	duri	559	DCYTD	1
397	dcyt	1160	nh4	559	DCYTD	0
398	dcyt[p]	98	h	560	DCYTt2pp	0
398	dcyt[p]	397	dcyt	560	DCYTt2pp	1
437	h[p]	98	h	560	DCYTt2pp	0
437	h[p]	397	dcyt	560	DCYTt2pp	0
399	dcyt[e]	398	dcyt[p]	561	DCYTtex	1
400	ddca[e]	1261	ddca[p]	562	DDCAtexi	1
135	atp	98	h	563	DDGALK	0
135	atp	281	adp	563	DDGALK	1
135	atp	406	2dh3dgal6p	563	DDGALK	0
401	2dh3dgal	98	h	563	DDGALK	0
401	2dh3dgal	281	adp	563	DDGALK	0
401	2dh3dgal	406	2dh3dgal6p	563	DDGALK	1
402	2ddglcn[p]	98	h	564	DDGLCNt2rpp	0
402	2ddglcn[p]	404	2ddglcn	564	DDGLCNt2rpp	1
437	h[p]	98	h	564	DDGLCNt2rpp	0
437	h[p]	404	2ddglcn	564	DDGLCNt2rpp	0
403	2ddglcn[e]	402	2ddglcn[p]	565	DDGLCNtex	1
135	atp	98	h	566	DDGLK	0
135	atp	281	adp	566	DDGLK	1
135	atp	483	2ddg6p	566	DDGLK	0
404	2ddglcn	98	h	566	DDGLK	0
404	2ddglcn	281	adp	566	DDGLK	0
404	2ddglcn	483	2ddg6p	566	DDGLK	1
109	h2o	430	2dda7p	567	DDPA	0
109	h2o	962	pi	567	DDPA	0
405	e4p	430	2dda7p	567	DDPA	1
405	e4p	962	pi	567	DDPA	0
959	pep	430	2dda7p	567	DDPA	1
959	pep	962	pi	567	DDPA	0
406	2dh3dgal6p	468	g3p	568	DDPGALA	1
406	2dh3dgal6p	1148	pyr	568	DDPGALA	1
135	atp	281	adp	569	DGK1	1
135	atp	1349	dgdp	569	DGK1	0
887	dgmp	281	adp	569	DGK1	0
887	dgmp	1349	dgdp	569	DGK1	1
407	dgmp[e]	888	dgmp[p]	570	DGMPtex	1
408	dgsn[p]	98	h	571	DGSNt2pp	0
408	dgsn[p]	966	dgsn	571	DGSNt2pp	1
437	h[p]	98	h	571	DGSNt2pp	0

437	h[p]	966	dgsn	571	DGSNt2pp	0
409	dgsn[e]	408	dgsn[p]	572	DGSNtex	1
410	23dhmb	109	h2o	573	DHAD1	0
410	23dhmb	729	3mob	573	DHAD1	1
411	23dhmp	109	h2o	574	DHAD2	0
411	23dhmp	1131	3mop	574	DHAD2	1
412	dha	826	dhap	575	DHAPT	1
412	dha	1148	pyr	575	DHAPT	0
959	pep	826	dhap	575	DHAPT	0
959	pep	1148	pyr	575	DHAPT	1
413	dha[e]	414	dha[p]	576	DHAtex	1
414	dha[p]	412	dha	577	DHAtpp	1
415	23ddhb	98	h	578	DHBD	0
415	23ddhb	416	23dhb	578	DHBD	1
415	23ddhb	870	nadh	578	DHBD	0
856	nad	98	h	578	DHBD	0
856	nad	416	23dhb	578	DHBD	0
856	nad	870	nadh	578	DHBD	1
98	h	490	23dhba	579	DHBS	0
98	h	1192	ppi	579	DHBS	0
135	atp	490	23dhba	579	DHBS	1
135	atp	1192	ppi	579	DHBS	0
416	23dhb	490	23dhba	579	DHBS	1
416	23dhb	1192	ppi	579	DHBS	0
109	h2o	416	23dhb	580	DHBSH	0
109	h2o	604	ser-L	580	DHBSH	0
417	23dhbzs	416	23dhb	580	DHBSH	1
417	23dhbzs	604	ser-L	580	DHBSH	1
418	cenchddd	98	h	581	DHCIND	0
418	cenchddd	419	dhcinnm	581	DHCIND	1
418	cenchddd	870	nadh	581	DHCIND	0
856	nad	98	h	581	DHCIND	0
856	nad	419	dhcinnm	581	DHCIND	0
856	nad	870	nadh	581	DHCIND	1
419	dhcinnm	98	h	582	DHCINDO	0
419	dhcinnm	1415	hkntd	582	DHCINDO	1
928	o2	98	h	582	DHCINDO	0
928	o2	1415	hkntd	582	DHCINDO	0
98	h	459	nadp	583	DHDPRy	0
98	h	1592	thdp	583	DHDPRy	0
420	23dhdp	459	nadp	583	DHDPRy	0
420	23dhdp	1592	thdp	583	DHDPRy	1
871	nadph	459	nadp	583	DHDPRy	1
871	nadph	1592	thdp	583	DHDPRy	0
268	aspsa	98	h	584	DHDPS	0
268	aspsa	109	h2o	584	DHDPS	0
268	aspsa	420	23dhdp	584	DHDPS	1
1148	pyr	98	h	584	DHDPS	0
1148	pyr	109	h2o	584	DHDPS	0
1148	pyr	420	23dhdp	584	DHDPS	1
98	h	459	nadp	585	DHFR	0
98	h	1403	thf	585	DHFR	0
421	dhf	459	nadp	585	DHFR	0
421	dhf	1403	thf	585	DHFR	1
871	nadph	459	nadp	585	DHFR	1
871	nadph	1403	thf	585	DHFR	0

135	atp	98	h	586	DHFS	0
135	atp	281	adp	586	DHFS	1
135	atp	421	dhf	586	DHFS	0
135	atp	962	pi	586	DHFS	0
624	glu-L	98	h	586	DHFS	0
624	glu-L	281	adp	586	DHFS	0
624	glu-L	421	dhf	586	DHFS	1
624	glu-L	962	pi	586	DHFS	0
1262	dhpt	98	h	586	DHFS	0
1262	dhpt	281	adp	586	DHFS	0
1262	dhpt	421	dhf	586	DHFS	1
1262	dhpt	962	pi	586	DHFS	0
98	h	245	2dmmql8	587	DHNAOT4	0
98	h	692	co2	587	DHNAOT4	0
98	h	1192	ppi	587	DHNAOT4	0
422	dhna	245	2dmmql8	587	DHNAOT4	1
422	dhna	692	co2	587	DHNAOT4	0
422	dhna	1192	ppi	587	DHNAOT4	0
1333	octdp	245	2dmmql8	587	DHNAOT4	1
1333	octdp	692	co2	587	DHNAOT4	0
1333	octdp	1192	ppi	587	DHNAOT4	0
423	dhnpt	597	gcald	588	DHNPA2	1
423	dhnpt	705	6hmhpt	588	DHNPA2	1
424	dhor-S	1169	orot	589	DHORD2	1
424	dhor-S	1443	q8h2	589	DHORD2	0
1003	q8	1169	orot	589	DHORD2	0
1003	q8	1443	q8h2	589	DHORD2	1
424	dhor-S	867	mql8	590	DHORD5	0
424	dhor-S	1169	orot	590	DHORD5	1
1282	mqn8	867	mql8	590	DHORD5	1
1282	mqn8	1169	orot	590	DHORD5	0
109	h2o	98	h	591	DHORTS	0
109	h2o	1102	cbasp	591	DHORTS	0
424	dhor-S	98	h	591	DHORTS	0
424	dhor-S	1102	cbasp	591	DHORTS	1
425	cechddd	98	h	592	DHPPD	0
425	cechddd	706	dhpppn	592	DHPPD	1
425	cechddd	870	nadh	592	DHPPD	0
856	nad	98	h	592	DHPPD	0
856	nad	706	dhpppn	592	DHPPD	0
856	nad	870	nadh	592	DHPPD	1
98	h	254	5apru	593	DHPPDA2	0
98	h	1160	nh4	593	DHPPDA2	0
109	h2o	254	5apru	593	DHPPDA2	0
109	h2o	1160	nh4	593	DHPPDA2	0
426	25drapp	254	5apru	593	DHPPDA2	1
426	25drapp	1160	nh4	593	DHPPDA2	0
427	4abz	1192	ppi	594	DHPS2	0
427	4abz	1262	dhpt	594	DHPS2	1
1295	6hmhptpp	1192	ppi	594	DHPS2	0
1295	6hmhptpp	1262	dhpt	594	DHPS2	1
428	dhptd	109	h2o	595	DHPTDCs	0
428	dhptd	442	hmfurn	595	DHPTDCs	1
429	ahdt	1103	dhmptp	596	DHPTPE	1
430	2dda7p	431	3dhq	597	DHQS	1
430	2dda7p	962	pi	597	DHQS	0

431	3dhq	109	h2o	598	DHQTi	0
431	3dhq	993	3dhsk	598	DHQTi	1
432	dimp[e]	877	dimp[p]	599	DIMPtex	1
433	din[p]	98	h	600	DINSt2pp	0
433	din[p]	968	din	600	DINSt2pp	1
437	h[p]	98	h	600	DINSt2pp	0
437	h[p]	968	din	600	DINSt2pp	0
434	din[e]	433	din[p]	601	DINStex	1
98	h	61	2dhguln	602	DKGLCNR1	0
98	h	459	nadp	602	DKGLCNR1	0
435	25dkglcn	61	2dhguln	602	DKGLCNR1	1
435	25dkglcn	459	nadp	602	DKGLCNR1	0
871	nadph	61	2dhguln	602	DKGLCNR1	0
871	nadph	459	nadp	602	DKGLCNR1	1
98	h	113	5dglcn	603	DKGLCNR2x	0
98	h	856	nad	603	DKGLCNR2x	0
435	25dkglcn	113	5dglcn	603	DKGLCNR2x	1
435	25dkglcn	856	nad	603	DKGLCNR2x	0
870	nadh	113	5dglcn	603	DKGLCNR2x	0
870	nadh	856	nad	603	DKGLCNR2x	1
98	h	113	5dglcn	604	DKGLCNR2y	0
98	h	459	nadp	604	DKGLCNR2y	0
435	25dkglcn	113	5dglcn	604	DKGLCNR2y	1
435	25dkglcn	459	nadp	604	DKGLCNR2y	0
871	nadph	113	5dglcn	604	DKGLCNR2y	0
871	nadph	459	nadp	604	DKGLCNR2y	1
109	h2o	98	h	605	DKMPPD	0
109	h2o	536	for	605	DKMPPD	0
109	h2o	962	pi	605	DKMPPD	0
109	h2o	1050	2kmb	605	DKMPPD	0
436	dkmpp	98	h	605	DKMPPD	0
436	dkmpp	536	for	605	DKMPPD	1
436	dkmpp	962	pi	605	DKMPPD	0
436	dkmpp	1050	2kmb	605	DKMPPD	1
928	o2	98	h	605	DKMPPD	0
928	o2	536	for	605	DKMPPD	0
928	o2	962	pi	605	DKMPPD	0
928	o2	1050	2kmb	605	DKMPPD	0
109	h2o	98	h	606	DKMPPD3	0
109	h2o	536	for	606	DKMPPD3	0
109	h2o	870	nadh	606	DKMPPD3	0
109	h2o	962	pi	606	DKMPPD3	0
109	h2o	1050	2kmb	606	DKMPPD3	0
436	dkmpp	98	h	606	DKMPPD3	0
436	dkmpp	536	for	606	DKMPPD3	1
436	dkmpp	870	nadh	606	DKMPPD3	0
436	dkmpp	962	pi	606	DKMPPD3	0
436	dkmpp	1050	2kmb	606	DKMPPD3	1
856	nad	98	h	606	DKMPPD3	0
856	nad	536	for	606	DKMPPD3	0
856	nad	870	nadh	606	DKMPPD3	1
856	nad	962	pi	606	DKMPPD3	0
856	nad	1050	2kmb	606	DKMPPD3	0
437	h[p]	98	h	607	D-LACt2pp	0
437	h[p]	748	lac-D	607	D-LACt2pp	0
1104	lac-D[p]	98	h	607	D-LACt2pp	0

1104	lac-D[p]	748	lac-D	607	D-LACt2pp	1
438	lac-D[e]	1104	lac-D[p]	608	D-LACtex	1
444	dmpp	664	grdp	614	DMATT	1
444	dmpp	1192	ppi	614	DMATT	0
726	ipdp	664	grdp	614	DMATT	1
726	ipdp	1192	ppi	614	DMATT	0
98	h	109	h2o	615	DMPPS	0
98	h	444	dmpp	615	DMPPS	0
98	h	856	nad	615	DMPPS	0
870	nadh	109	h2o	615	DMPPS	0
870	nadh	444	dmpp	615	DMPPS	0
870	nadh	856	nad	615	DMPPS	1
1319	h2mb4p	109	h2o	615	DMPPS	0
1319	h2mb4p	444	dmpp	615	DMPPS	1
1319	h2mb4p	856	nad	615	DMPPS	0
178	amet	98	h	616	DMQMT	0
178	amet	209	ahcys	616	DMQMT	1
178	amet	1443	q8h2	616	DMQMT	1
445	2omhmbl	98	h	616	DMQMT	0
445	2omhmbl	209	ahcys	616	DMQMT	0
445	2omhmbl	1443	q8h2	616	DMQMT	1
446	dmso	109	h2o	617	DMSOR1	0
446	dmso	1263	dms	617	DMSOR1	1
446	dmso	1282	mqn8	617	DMSOR1	0
867	mql8	109	h2o	617	DMSOR1	0
867	mql8	1263	dms	617	DMSOR1	0
867	mql8	1282	mqn8	617	DMSOR1	1
447	dmso[p]	684	h2o[p]	618	DMSOR1pp	0
447	dmso[p]	1105	dms[p]	618	DMSOR1pp	1
447	dmso[p]	1282	mqn8	618	DMSOR1pp	0
867	mql8	684	h2o[p]	618	DMSOR1pp	0
867	mql8	1105	dms[p]	618	DMSOR1pp	0
867	mql8	1282	mqn8	618	DMSOR1pp	1
245	2dmmql8	109	h2o	619	DMSOR2	0
245	2dmmql8	560	2dmmq8	619	DMSOR2	1
245	2dmmql8	1263	dms	619	DMSOR2	0
446	dmso	109	h2o	619	DMSOR2	0
446	dmso	560	2dmmq8	619	DMSOR2	0
446	dmso	1263	dms	619	DMSOR2	1
245	2dmmql8	560	2dmmq8	620	DMSOR2pp	1
245	2dmmql8	684	h2o[p]	620	DMSOR2pp	0
245	2dmmql8	1105	dms[p]	620	DMSOR2pp	0
447	dmso[p]	560	2dmmq8	620	DMSOR2pp	0
447	dmso[p]	684	h2o[p]	620	DMSOR2pp	0
447	dmso[p]	1105	dms[p]	620	DMSOR2pp	1
448	dmso[e]	447	dmso[p]	621	DMSOtex	1
449	dms[e]	1105	dms[p]	622	DMStex	1
109	h2o	423	dhnpt	623	DNMPPA	0
109	h2o	962	pi	623	DNMPPA	0
450	dhpmp	423	dhnpt	623	DNMPPA	1
450	dhpmp	962	pi	623	DNMPPA	0
109	h2o	98	h	624	DNTPPA	0
109	h2o	450	dhpmp	624	DNTPPA	0
109	h2o	1192	ppi	624	DNTPPA	0
429	ahdt	98	h	624	DNTPPA	0
429	ahdt	450	dhpmp	624	DNTPPA	1

429	ahdt	1192	ppi	624	DNTPPA	0
98	h	81	3dhguln	625	DOGULNR	0
98	h	856	nad	625	DOGULNR	0
451	23doguln	81	3dhguln	625	DOGULNR	1
451	23doguln	856	nad	625	DOGULNR	0
870	nadh	81	3dhguln	625	DOGULNR	0
870	nadh	856	nad	625	DOGULNR	1
452	dopa[e]	107	dopa[p]	626	DOPAtex	1
135	atp	98	h	627	DPCOAK	0
135	atp	281	adp	627	DPCOAK	1
135	atp	927	coa	627	DPCOAK	0
1347	dpcoa	98	h	627	DPCOAK	0
1347	dpcoa	281	adp	627	DPCOAK	0
1347	dpcoa	927	coa	627	DPCOAK	1
98	h	459	nadp	628	DPR	0
98	h	1593	pant-R	628	DPR	0
453	2dhp	459	nadp	628	DPR	0
453	2dhp	1593	pant-R	628	DPR	1
871	nadph	459	nadp	628	DPR	1
871	nadph	1593	pant-R	628	DPR	0
454	2dr5p	131	acald	629	DRPA	1
454	2dr5p	468	g3p	629	DRPA	1
455	dsbard[p]	1264	dsbaox[p]	630	DSBAO1	1
455	dsbard[p]	1443	q8h2	630	DSBAO1	0
1003	q8	1264	dsbaox[p]	630	DSBAO1	0
1003	q8	1443	q8h2	630	DSBAO1	1
455	dsbard[p]	867	mql8	631	DSBAO2	0
455	dsbard[p]	1264	dsbaox[p]	631	DSBAO2	1
1282	mqn8	867	mql8	631	DSBAO2	1
1282	mqn8	1264	dsbaox[p]	631	DSBAO2	0
456	dsbcox[p]	1120	gthox[p]	632	DSBCGT	0
456	dsbcox[p]	1265	dsbcrd[p]	632	DSBCGT	1
671	gthrd[p]	1120	gthox[p]	632	DSBCGT	1
671	gthrd[p]	1265	dsbcrd[p]	632	DSBCGT	0
457	dsbdox	1016	dsbdrd	633	DSBDR	1
457	dsbdox	1594	trdox	633	DSBDR	0
981	trdrd	1016	dsbdrd	633	DSBDR	0
981	trdrd	1594	trdox	633	DSBDR	1
458	dsbgox[p]	1120	gthox[p]	634	DSBGGT	0
458	dsbgox[p]	1266	dsbgrd[p]	634	DSBGGT	1
671	gthrd[p]	1120	gthox[p]	634	DSBGGT	1
671	gthrd[p]	1266	dsbgrd[p]	634	DSBGGT	0
459	nadp	98	h	635	DSERDHr	0
459	nadp	871	nadph	635	DSERDHr	1
459	nadp	1106	2amsa	635	DSERDHr	0
988	ser-D	98	h	635	DSERDHr	0
988	ser-D	871	nadph	635	DSERDHr	0
988	ser-D	1106	2amsa	635	DSERDHr	1
437	h[p]	98	h	636	DSERt2pp	0
437	h[p]	988	ser-D	636	DSERt2pp	0
1107	ser-D[p]	98	h	636	DSERt2pp	0
1107	ser-D[p]	988	ser-D	636	DSERt2pp	1
460	ser-D[e]	1107	ser-D[p]	637	DSERtex	1
135	atp	281	adp	638	DTMPK	1
135	atp	1239	dtdp	638	DTMPK	0
882	dtmp	281	adp	638	DTMPK	0

882	dtmp	1239	dtdp	638	DTMPK	1
461	dtmp[e]	883	dtmp[p]	639	DTMPtex	1
462	dump[e]	878	dump[p]	640	DUMPtex	1
135	atp	98	h	641	DURIK1	0
135	atp	281	adp	641	DURIK1	1
135	atp	875	dump	641	DURIK1	0
463	duri	98	h	641	DURIK1	0
463	duri	281	adp	641	DURIK1	0
463	duri	875	dump	641	DURIK1	1
463	duri	950	2dr1p	642	DURIPP	1
463	duri	1552	ura	642	DURIPP	1
962	pi	950	2dr1p	642	DURIPP	0
962	pi	1552	ura	642	DURIPP	0
437	h[p]	98	h	643	DURIt2pp	0
437	h[p]	463	duri	643	DURIt2pp	0
464	duri[p]	98	h	643	DURIt2pp	0
464	duri[p]	463	duri	643	DURIt2pp	1
465	duri[e]	464	duri[p]	644	DURItex	1
109	h2o	98	h	645	DUTPDP	0
109	h2o	875	dump	645	DUTPDP	0
109	h2o	1192	ppi	645	DUTPDP	0
466	dutp	98	h	645	DUTPDP	0
466	dutp	875	dump	645	DUTPDP	1
466	dutp	1192	ppi	645	DUTPDP	0
98	h	459	nadp	646	DXPRIi	0
98	h	816	2me4p	646	DXPRIi	0
467	dxyl5p	459	nadp	646	DXPRIi	0
467	dxyl5p	816	2me4p	646	DXPRIi	1
871	nadph	459	nadp	646	DXPRIi	1
871	nadph	816	2me4p	646	DXPRIi	0
98	h	467	dxyl5p	647	DXPS	0
98	h	692	co2	647	DXPS	0
468	g3p	467	dxyl5p	647	DXPS	1
468	g3p	692	co2	647	DXPS	0
1148	pyr	467	dxyl5p	647	DXPS	1
1148	pyr	692	co2	647	DXPS	0
135	atp	98	h	648	DXYLK	0
135	atp	281	adp	648	DXYLK	1
135	atp	467	dxyl5p	648	DXYLK	0
1392	dxyl	98	h	648	DXYLK	0
1392	dxyl	281	adp	648	DXYLK	0
1392	dxyl	467	dxyl5p	648	DXYLK	1
109	h2o	98	h	649	E4PD	0
109	h2o	870	nadh	649	E4PD	0
109	h2o	930	4per	649	E4PD	0
405	e4p	98	h	649	E4PD	0
405	e4p	870	nadh	649	E4PD	0
405	e4p	930	4per	649	E4PD	1
856	nad	98	h	649	E4PD	0
856	nad	870	nadh	649	E4PD	1
856	nad	930	4per	649	E4PD	0
98	h	99	dcaACP	650	EAR100x	0
98	h	856	nad	650	EAR100x	0
870	nadh	99	dcaACP	650	EAR100x	0
870	nadh	856	nad	650	EAR100x	1
1006	tdec2eACP	99	dcaACP	650	EAR100x	1

1006	tdec2eACP	856	nad	650	EAR100x	0
98	h	99	dcaACP	651	EAR100y	0
98	h	459	nadp	651	EAR100y	0
871	nadph	99	dcaACP	651	EAR100y	0
871	nadph	459	nadp	651	EAR100y	1
1006	tdec2eACP	99	dcaACP	651	EAR100y	1
1006	tdec2eACP	459	nadp	651	EAR100y	0
98	h	101	ddcaACP	652	EAR120x	0
98	h	856	nad	652	EAR120x	0
870	nadh	101	ddcaACP	652	EAR120x	0
870	nadh	856	nad	652	EAR120x	1
1570	tddec2eACP	101	ddcaACP	652	EAR120x	1
1570	tddec2eACP	856	nad	652	EAR120x	0
98	h	101	ddcaACP	653	EAR120y	0
98	h	459	nadp	653	EAR120y	0
871	nadph	101	ddcaACP	653	EAR120y	0
871	nadph	459	nadp	653	EAR120y	1
1570	tddec2eACP	101	ddcaACP	653	EAR120y	1
1570	tddec2eACP	459	nadp	653	EAR120y	0
98	h	102	cddec5eACP	654	EAR121x	0
98	h	856	nad	654	EAR121x	0
870	nadh	102	cddec5eACP	654	EAR121x	0
870	nadh	856	nad	654	EAR121x	1
1571	t3c5ddeceACP	102	cddec5eACP	654	EAR121x	1
1571	t3c5ddeceACP	856	nad	654	EAR121x	0
98	h	102	cddec5eACP	655	EAR121y	0
98	h	459	nadp	655	EAR121y	0
871	nadph	102	cddec5eACP	655	EAR121y	0
871	nadph	459	nadp	655	EAR121y	1
1571	t3c5ddeceACP	102	cddec5eACP	655	EAR121y	1
1571	t3c5ddeceACP	459	nadp	655	EAR121y	0
98	h	856	nad	656	EAR140x	0
98	h	1267	myrsACP	656	EAR140x	0
870	nadh	856	nad	656	EAR140x	1
870	nadh	1267	myrsACP	656	EAR140x	0
1572	tmrs2eACP	856	nad	656	EAR140x	0
1572	tmrs2eACP	1267	myrsACP	656	EAR140x	1
98	h	459	nadp	657	EAR140y	0
98	h	1267	myrsACP	657	EAR140y	0
871	nadph	459	nadp	657	EAR140y	1
871	nadph	1267	myrsACP	657	EAR140y	0
1572	tmrs2eACP	459	nadp	657	EAR140y	0
1572	tmrs2eACP	1267	myrsACP	657	EAR140y	1
98	h	856	nad	658	EAR141x	0
98	h	1373	tdeACP	658	EAR141x	0
870	nadh	856	nad	658	EAR141x	1
870	nadh	1373	tdeACP	658	EAR141x	0
1573	t3c7mrseACP	856	nad	658	EAR141x	0
1573	t3c7mrseACP	1373	tdeACP	658	EAR141x	1
98	h	459	nadp	659	EAR141y	0
98	h	1373	tdeACP	659	EAR141y	0
871	nadph	459	nadp	659	EAR141y	1
871	nadph	1373	tdeACP	659	EAR141y	0
1573	t3c7mrseACP	459	nadp	659	EAR141y	0
1573	t3c7mrseACP	1373	tdeACP	659	EAR141y	1
98	h	856	nad	660	EAR160x	0

98	h	1374	palmACP	660	EAR160x	0
870	nadh	856	nad	660	EAR160x	1
870	nadh	1374	palmACP	660	EAR160x	0
1574	tpalm2eACP	856	nad	660	EAR160x	0
1574	tpalm2eACP	1374	palmACP	660	EAR160x	1
98	h	459	nadp	661	EAR160y	0
98	h	1374	palmACP	661	EAR160y	0
871	nadph	459	nadp	661	EAR160y	1
871	nadph	1374	palmACP	661	EAR160y	0
1574	tpalm2eACP	459	nadp	661	EAR160y	0
1574	tpalm2eACP	1374	palmACP	661	EAR160y	1
98	h	486	hdeACP	662	EAR161x	0
98	h	856	nad	662	EAR161x	0
870	nadh	486	hdeACP	662	EAR161x	0
870	nadh	856	nad	662	EAR161x	1
1575	t3c9palmeACP	486	hdeACP	662	EAR161x	1
1575	t3c9palmeACP	856	nad	662	EAR161x	0
98	h	459	nadp	663	EAR161y	0
98	h	486	hdeACP	663	EAR161y	0
871	nadph	459	nadp	663	EAR161y	1
871	nadph	486	hdeACP	663	EAR161y	0
1575	t3c9palmeACP	459	nadp	663	EAR161y	0
1575	t3c9palmeACP	486	hdeACP	663	EAR161y	1
98	h	856	nad	664	EAR180x	0
98	h	1375	ocdcaACP	664	EAR180x	0
870	nadh	856	nad	664	EAR180x	1
870	nadh	1375	ocdcaACP	664	EAR180x	0
1576	toctd2eACP	856	nad	664	EAR180x	0
1576	toctd2eACP	1375	ocdcaACP	664	EAR180x	1
98	h	459	nadp	665	EAR180y	0
98	h	1375	ocdcaACP	665	EAR180y	0
871	nadph	459	nadp	665	EAR180y	1
871	nadph	1375	ocdcaACP	665	EAR180y	0
1576	toctd2eACP	459	nadp	665	EAR180y	0
1576	toctd2eACP	1375	ocdcaACP	665	EAR180y	1
98	h	856	nad	666	EAR181x	0
98	h	1376	octeACP	666	EAR181x	0
870	nadh	856	nad	666	EAR181x	1
870	nadh	1376	octeACP	666	EAR181x	0
1577	t3c11vaceACP	856	nad	666	EAR181x	0
1577	t3c11vaceACP	1376	octeACP	666	EAR181x	1
98	h	459	nadp	667	EAR181y	0
98	h	1376	octeACP	667	EAR181y	0
871	nadph	459	nadp	667	EAR181y	1
871	nadph	1376	octeACP	667	EAR181y	0
1577	t3c11vaceACP	459	nadp	667	EAR181y	0
1577	t3c11vaceACP	1376	octeACP	667	EAR181y	1
98	h	103	butACP	668	EAR40x	0
98	h	856	nad	668	EAR40x	0
469	but2eACP	103	butACP	668	EAR40x	1
469	but2eACP	856	nad	668	EAR40x	0
870	nadh	103	butACP	668	EAR40x	0
870	nadh	856	nad	668	EAR40x	1
98	h	103	butACP	669	EAR40y	0
98	h	459	nadp	669	EAR40y	0
469	but2eACP	103	butACP	669	EAR40y	1

469	but2eACP	459	nadp	669	EAR40y	0
871	nadph	103	butACP	669	EAR40y	0
871	nadph	459	nadp	669	EAR40y	1
98	h	856	nad	670	EAR60x	0
98	h	1268	hexACP	670	EAR60x	0
870	nadh	856	nad	670	EAR60x	1
870	nadh	1268	hexACP	670	EAR60x	0
1578	thex2eACP	856	nad	670	EAR60x	0
1578	thex2eACP	1268	hexACP	670	EAR60x	1
98	h	459	nadp	671	EAR60y	0
98	h	1268	hexACP	671	EAR60y	0
871	nadph	459	nadp	671	EAR60y	1
871	nadph	1268	hexACP	671	EAR60y	0
1578	thex2eACP	459	nadp	671	EAR60y	0
1578	thex2eACP	1268	hexACP	671	EAR60y	1
98	h	856	nad	672	EAR80x	0
98	h	1395	ocACP	672	EAR80x	0
870	nadh	856	nad	672	EAR80x	1
870	nadh	1395	ocACP	672	EAR80x	0
1579	toct2eACP	856	nad	672	EAR80x	0
1579	toct2eACP	1395	ocACP	672	EAR80x	1
98	h	459	nadp	673	EAR80y	0
98	h	1395	ocACP	673	EAR80y	0
871	nadph	459	nadp	673	EAR80y	1
871	nadph	1395	ocACP	673	EAR80y	0
1579	toct2eACP	459	nadp	673	EAR80y	0
1579	toct2eACP	1395	ocACP	673	EAR80y	1
470	eca4colipa[p]	1269	eca4colipa[e]	674	ECA4COLIPAtex	1
337	colipa[p]	437	h[p]	675	ECA4OALpp	0
337	colipa[p]	470	eca4colipa[p]	675	ECA4OALpp	1
337	colipa[p]	1544	udcpdp[p]	675	ECA4OALpp	0
1270	eca4und[p]	437	h[p]	675	ECA4OALpp	0
1270	eca4und[p]	470	eca4colipa[p]	675	ECA4OALpp	1
1270	eca4und[p]	1544	udcpdp[p]	675	ECA4OALpp	1
471	unagamuf[p]	437	h[p]	676	ECAP1pp	0
471	unagamuf[p]	472	eca2und[p]	676	ECAP1pp	1
471	unagamuf[p]	1544	udcpdp[p]	676	ECAP1pp	1
471	unagamuf[p]	437	h[p]	677	ECAP2pp	0
471	unagamuf[p]	473	eca3und[p]	677	ECAP2pp	0
471	unagamuf[p]	1544	udcpdp[p]	677	ECAP2pp	1
472	eca2und[p]	437	h[p]	677	ECAP2pp	0
472	eca2und[p]	473	eca3und[p]	677	ECAP2pp	1
472	eca2und[p]	1544	udcpdp[p]	677	ECAP2pp	0
471	unagamuf[p]	437	h[p]	678	ECAP3pp	0
471	unagamuf[p]	1270	eca4und[p]	678	ECAP3pp	0
471	unagamuf[p]	1544	udcpdp[p]	678	ECAP3pp	1
473	eca3und[p]	437	h[p]	678	ECAP3pp	0
473	eca3und[p]	1270	eca4und[p]	678	ECAP3pp	1
473	eca3und[p]	1544	udcpdp[p]	678	ECAP3pp	0
474	unagamuf	471	unagamuf[p]	679	ECAtpp	1
475	3hbcoa	109	h2o	680	ECOAH1	0
475	3hbcoa	1076	b2coa	680	ECOAH1	1
476	3hhcoa	109	h2o	681	ECOAH2	0
476	3hhcoa	1556	hx2coa	681	ECOAH2	1
477	3hocoa	109	h2o	682	ECOAH3	0
477	3hocoa	1557	oc2coa	682	ECOAH3	1

478	3hdcoa	109	h2o	683	ECOAH4	0
478	3hdcoa	1077	dc2coa	683	ECOAH4	1
479	3hddcoa	109	h2o	684	ECOAH5	0
479	3hddcoa	1078	dd2coa	684	ECOAH5	1
480	3htdcoa	109	h2o	685	ECOAH6	0
480	3htdcoa	1354	td2coa	685	ECOAH6	1
481	3hhdcoa	109	h2o	686	ECOAH7	0
481	3hhdcoa	1291	hdd2coa	686	ECOAH7	1
482	3hodcoa	109	h2o	687	ECOAH8	0
482	3hodcoa	1334	od2coa	687	ECOAH8	1
483	2ddg6p	468	g3p	688	EDA	1
483	2ddg6p	1148	pyr	688	EDA	1
484	6pgc	109	h2o	689	EDD	0
484	6pgc	483	2ddg6p	689	EDD	1
101	ddcaACP	118	ACP	690	EDTXS1	1
101	ddcaACP	485	kdo2lipid4L	690	EDTXS1	1
1393	kdo2lipid4	118	ACP	690	EDTXS1	0
1393	kdo2lipid4	485	kdo2lipid4L	690	EDTXS1	1
485	kdo2lipid4L	118	ACP	691	EDTXS2	0
485	kdo2lipid4L	1408	lipa	691	EDTXS2	1
1267	myrsACP	118	ACP	691	EDTXS2	1
1267	myrsACP	1408	lipa	691	EDTXS2	1
486	hdeACP	118	ACP	692	EDTXS3	1
486	hdeACP	487	kdo2lipid4p	692	EDTXS3	1
1393	kdo2lipid4	118	ACP	692	EDTXS3	0
1393	kdo2lipid4	487	kdo2lipid4p	692	EDTXS3	1
487	kdo2lipid4p	118	ACP	693	EDTXS4	0
487	kdo2lipid4p	1595	lipa_cold	693	EDTXS4	1
1267	myrsACP	118	ACP	693	EDTXS4	1
1267	myrsACP	1595	lipa_cold	693	EDTXS4	1
488	enlipa[p]	1271	enlipa[e]	694	ENLIPAtex	1
489	2pg	109	h2o	695	ENO	0
489	2pg	959	pep	695	ENO	1
490	23dhba	98	h	696	ENTCS	0
490	23dhba	177	amp	696	ENTCS	0
490	23dhba	491	enter	696	ENTCS	1
1394	seramp	98	h	696	ENTCS	0
1394	seramp	177	amp	696	ENTCS	1
1394	seramp	491	enter	696	ENTCS	1
109	h2o	98	h	697	ENTERES	0
109	h2o	417	23dhbzs	697	ENTERES	0
491	enter	98	h	697	ENTERES	0
491	enter	417	23dhbzs	697	ENTERES	1
109	h2o	98	h	698	ENTERES2	0
109	h2o	417	23dhbzs	698	ENTERES2	0
109	h2o	1398	fe3	698	ENTERES2	0
492	feenter	98	h	698	ENTERES2	0
492	feenter	417	23dhbzs	698	ENTERES2	1
492	feenter	1398	fe3	698	ENTERES2	0
493	etha	131	acald	699	ETHAAL	1
493	etha	1160	nh4	699	ETHAAL	0
437	h[p]	98	h	700	ETHAt2pp	0
437	h[p]	493	etha	700	ETHAt2pp	0
494	etha[p]	98	h	700	ETHAt2pp	0
494	etha[p]	493	etha	700	ETHAt2pp	1
495	etha[e]	494	etha[p]	701	ETHAtex	1

109	h2o	98	h	702	ETHSO3abcpp	0
109	h2o	281	adp	702	ETHSO3abcpp	0
109	h2o	512	ethso3	702	ETHSO3abcpp	0
109	h2o	962	pi	702	ETHSO3abcpp	0
135	atp	98	h	702	ETHSO3abcpp	0
135	atp	281	adp	702	ETHSO3abcpp	1
135	atp	512	ethso3	702	ETHSO3abcpp	0
135	atp	962	pi	702	ETHSO3abcpp	0
1108	ethso3[p]	98	h	702	ETHSO3abcpp	0
1108	ethso3[p]	281	adp	702	ETHSO3abcpp	0
1108	ethso3[p]	512	ethso3	702	ETHSO3abcpp	1
1108	ethso3[p]	962	pi	702	ETHSO3abcpp	0
496	ethso3[e]	1108	ethso3[p]	703	ETHSO3tex	1
437	h[p]	98	h	704	ETOHt2rpp	0
437	h[p]	225	etoh	704	ETOHt2rpp	0
497	etoh[p]	98	h	704	ETOHt2rpp	0
497	etoh[p]	225	etoh	704	ETOHt2rpp	1
498	etoh[e]	497	etoh[p]	705	ETOHtex	1
499	f6p	412	dha	706	F6PA	1
499	f6p	468	g3p	706	F6PA	1
109	h2o	962	pi	707	F6PP	0
109	h2o	1235	fru	707	F6PP	0
499	f6p	962	pi	707	F6PP	0
499	f6p	1235	fru	707	F6PP	1
500	f6p[p]	499	f6p	708	F6Pt6_2pp	1
500	f6p[p]	1173	pi[p]	708	F6Pt6_2pp	0
962	pi	499	f6p	708	F6Pt6_2pp	0
962	pi	1173	pi[p]	708	F6Pt6_2pp	0
501	f6p[e]	500	f6p[p]	709	F6Ptex	1
99	dcaACP	98	h	710	FA100ACPHi	0
99	dcaACP	118	ACP	710	FA100ACPHi	1
99	dcaACP	1596	dca	710	FA100ACPHi	1
109	h2o	98	h	710	FA100ACPHi	0
109	h2o	118	ACP	710	FA100ACPHi	0
109	h2o	1596	dca	710	FA100ACPHi	0
101	ddcaACP	98	h	711	FA120ACPHi	0
101	ddcaACP	118	ACP	711	FA120ACPHi	1
101	ddcaACP	1303	ddca	711	FA120ACPHi	1
109	h2o	98	h	711	FA120ACPHi	0
109	h2o	118	ACP	711	FA120ACPHi	0
109	h2o	1303	ddca	711	FA120ACPHi	0
109	h2o	98	h	712	FA140ACPHi	0
109	h2o	118	ACP	712	FA140ACPHi	0
109	h2o	1616	ttdca	712	FA140ACPHi	0
1267	myrsACP	98	h	712	FA140ACPHi	0
1267	myrsACP	118	ACP	712	FA140ACPHi	1
1267	myrsACP	1616	ttdca	712	FA140ACPHi	1
109	h2o	98	h	713	FA141ACPHi	0
109	h2o	118	ACP	713	FA141ACPHi	0
109	h2o	1617	ttdcea	713	FA141ACPHi	0
1373	tdeACP	98	h	713	FA141ACPHi	0
1373	tdeACP	118	ACP	713	FA141ACPHi	1
1373	tdeACP	1617	ttdcea	713	FA141ACPHi	1
109	h2o	98	h	714	FA160ACPHi	0
109	h2o	118	ACP	714	FA160ACPHi	0
109	h2o	1618	hdca	714	FA160ACPHi	0

1374	palmACP	98	h	714	FA160ACPHi	0
1374	palmACP	118	ACP	714	FA160ACPHi	1
1374	palmACP	1618	hdca	714	FA160ACPHi	1
109	h2o	98	h	715	FA161ACPHi	0
109	h2o	118	ACP	715	FA161ACPHi	0
109	h2o	1619	hdcea	715	FA161ACPHi	0
486	hdeACP	98	h	715	FA161ACPHi	0
486	hdeACP	118	ACP	715	FA161ACPHi	1
486	hdeACP	1619	hdcea	715	FA161ACPHi	1
109	h2o	98	h	716	FA80ACPHi	0
109	h2o	118	ACP	716	FA80ACPHi	0
109	h2o	1622	octa	716	FA80ACPHi	0
1395	ocACP	98	h	716	FA80ACPHi	0
1395	ocACP	118	ACP	716	FA80ACPHi	1
1395	ocACP	1622	octa	716	FA80ACPHi	1
109	h2o	98	h	717	FACOAE100	0
109	h2o	927	coa	717	FACOAE100	0
109	h2o	1596	dca	717	FACOAE100	0
159	dcacoa	98	h	717	FACOAE100	0
159	dcacoa	927	coa	717	FACOAE100	1
159	dcacoa	1596	dca	717	FACOAE100	1
109	h2o	98	h	718	FACOAE120	0
109	h2o	927	coa	718	FACOAE120	0
109	h2o	1303	ddca	718	FACOAE120	0
160	ddcacoa	98	h	718	FACOAE120	0
160	ddcacoa	927	coa	718	FACOAE120	1
160	ddcacoa	1303	ddca	718	FACOAE120	1
109	h2o	98	h	719	FACOAE140	0
109	h2o	927	coa	719	FACOAE140	0
109	h2o	1616	ttdca	719	FACOAE140	0
1369	tdcoa	98	h	719	FACOAE140	0
1369	tdcoa	927	coa	719	FACOAE140	1
1369	tdcoa	1616	ttdca	719	FACOAE140	1
109	h2o	98	h	720	FACOAE141	0
109	h2o	927	coa	720	FACOAE141	0
109	h2o	1617	ttdcea	720	FACOAE141	0
1012	tdecoa	98	h	720	FACOAE141	0
1012	tdecoa	927	coa	720	FACOAE141	1
1012	tdecoa	1617	ttdcea	720	FACOAE141	1
109	h2o	98	h	721	FACOAE160	0
109	h2o	927	coa	721	FACOAE160	0
109	h2o	1618	hdca	721	FACOAE160	0
1370	pmtcoa	98	h	721	FACOAE160	0
1370	pmtcoa	927	coa	721	FACOAE160	1
1370	pmtcoa	1618	hdca	721	FACOAE160	1
109	h2o	98	h	722	FACOAE161	0
109	h2o	927	coa	722	FACOAE161	0
109	h2o	1619	hdcea	722	FACOAE161	0
696	hdcoa	98	h	722	FACOAE161	0
696	hdcoa	927	coa	722	FACOAE161	1
696	hdcoa	1619	hdcea	722	FACOAE161	1
109	h2o	98	h	723	FACOAE180	0
109	h2o	927	coa	723	FACOAE180	0
109	h2o	1620	ocdca	723	FACOAE180	0
1371	stcoa	98	h	723	FACOAE180	0
1371	stcoa	927	coa	723	FACOAE180	1

1371	stcoa	1620	ocdca	723	FACOAE180	1
109	h2o	98	h	724	FACOAE181	0
109	h2o	927	coa	724	FACOAE181	0
109	h2o	1621	ocdcea	724	FACOAE181	0
908	odecoa	98	h	724	FACOAE181	0
908	odecoa	927	coa	724	FACOAE181	1
908	odecoa	1621	ocdcea	724	FACOAE181	1
109	h2o	98	h	725	FACOAE60	0
109	h2o	927	coa	725	FACOAE60	0
109	h2o	1418	hxa	725	FACOAE60	0
1367	hxcoa	98	h	725	FACOAE60	0
1367	hxcoa	927	coa	725	FACOAE60	1
1367	hxcoa	1418	hxa	725	FACOAE60	1
109	h2o	98	h	726	FACOAE80	0
109	h2o	927	coa	726	FACOAE80	0
109	h2o	1622	octa	726	FACOAE80	0
1368	occoa	98	h	726	FACOAE80	0
1368	occoa	927	coa	726	FACOAE80	1
1368	occoa	1622	octa	726	FACOAE80	1
135	atp	98	h	727	FACOAL100t2pp	0
135	atp	159	dcacoa	727	FACOAL100t2pp	0
135	atp	177	amp	727	FACOAL100t2pp	1
135	atp	1192	ppi	727	FACOAL100t2pp	0
437	h[p]	98	h	727	FACOAL100t2pp	0
437	h[p]	159	dcacoa	727	FACOAL100t2pp	0
437	h[p]	177	amp	727	FACOAL100t2pp	0
437	h[p]	1192	ppi	727	FACOAL100t2pp	0
927	coa	98	h	727	FACOAL100t2pp	0
927	coa	159	dcacoa	727	FACOAL100t2pp	1
927	coa	177	amp	727	FACOAL100t2pp	0
927	coa	1192	ppi	727	FACOAL100t2pp	0
1101	dca[p]	98	h	727	FACOAL100t2pp	0
1101	dca[p]	159	dcacoa	727	FACOAL100t2pp	1
1101	dca[p]	177	amp	727	FACOAL100t2pp	0
1101	dca[p]	1192	ppi	727	FACOAL100t2pp	0
135	atp	98	h	728	FACOAL120t2pp	0
135	atp	160	ddcacoa	728	FACOAL120t2pp	0
135	atp	177	amp	728	FACOAL120t2pp	1
135	atp	1192	ppi	728	FACOAL120t2pp	0
437	h[p]	98	h	728	FACOAL120t2pp	0
437	h[p]	160	ddcacoa	728	FACOAL120t2pp	0
437	h[p]	177	amp	728	FACOAL120t2pp	0
437	h[p]	1192	ppi	728	FACOAL120t2pp	0
927	coa	98	h	728	FACOAL120t2pp	0
927	coa	160	ddcacoa	728	FACOAL120t2pp	1
927	coa	177	amp	728	FACOAL120t2pp	0
927	coa	1192	ppi	728	FACOAL120t2pp	0
1261	ddca[p]	98	h	728	FACOAL120t2pp	0
1261	ddca[p]	160	ddcacoa	728	FACOAL120t2pp	1
1261	ddca[p]	177	amp	728	FACOAL120t2pp	0
1261	ddca[p]	1192	ppi	728	FACOAL120t2pp	0
135	atp	98	h	729	FACOAL140t2pp	0
135	atp	177	amp	729	FACOAL140t2pp	1
135	atp	1192	ppi	729	FACOAL140t2pp	0
135	atp	1369	tdcoa	729	FACOAL140t2pp	0
437	h[p]	98	h	729	FACOAL140t2pp	0

437	h[p]	177	amp	729	FACOAL140t2pp	0
437	h[p]	1192	ppi	729	FACOAL140t2pp	0
437	h[p]	1369	tdcoa	729	FACOAL140t2pp	0
927	coa	98	h	729	FACOAL140t2pp	0
927	coa	177	amp	729	FACOAL140t2pp	0
927	coa	1192	ppi	729	FACOAL140t2pp	0
927	coa	1369	tdcoa	729	FACOAL140t2pp	1
1356	ttdca[p]	98	h	729	FACOAL140t2pp	0
1356	ttdca[p]	177	amp	729	FACOAL140t2pp	0
1356	ttdca[p]	1192	ppi	729	FACOAL140t2pp	0
1356	ttdca[p]	1369	tdcoa	729	FACOAL140t2pp	1
135	atp	98	h	730	FACOAL141t2pp	0
135	atp	177	amp	730	FACOAL141t2pp	1
135	atp	1012	tdecoa	730	FACOAL141t2pp	0
135	atp	1192	ppi	730	FACOAL141t2pp	0
437	h[p]	98	h	730	FACOAL141t2pp	0
437	h[p]	177	amp	730	FACOAL141t2pp	0
437	h[p]	1012	tdecoa	730	FACOAL141t2pp	0
437	h[p]	1192	ppi	730	FACOAL141t2pp	0
927	coa	98	h	730	FACOAL141t2pp	0
927	coa	177	amp	730	FACOAL141t2pp	0
927	coa	1012	tdecoa	730	FACOAL141t2pp	1
927	coa	1192	ppi	730	FACOAL141t2pp	0
1357	ttdcea[p]	98	h	730	FACOAL141t2pp	0
1357	ttdcea[p]	177	amp	730	FACOAL141t2pp	0
1357	ttdcea[p]	1012	tdecoa	730	FACOAL141t2pp	1
1357	ttdcea[p]	1192	ppi	730	FACOAL141t2pp	0
135	atp	98	h	731	FACOAL160t2pp	0
135	atp	177	amp	731	FACOAL160t2pp	1
135	atp	1192	ppi	731	FACOAL160t2pp	0
135	atp	1370	pmtcoa	731	FACOAL160t2pp	0
437	h[p]	98	h	731	FACOAL160t2pp	0
437	h[p]	177	amp	731	FACOAL160t2pp	0
437	h[p]	1192	ppi	731	FACOAL160t2pp	0
437	h[p]	1370	pmtcoa	731	FACOAL160t2pp	0
927	coa	98	h	731	FACOAL160t2pp	0
927	coa	177	amp	731	FACOAL160t2pp	0
927	coa	1192	ppi	731	FACOAL160t2pp	0
927	coa	1370	pmtcoa	731	FACOAL160t2pp	1
1289	hdca[p]	98	h	731	FACOAL160t2pp	0
1289	hdca[p]	177	amp	731	FACOAL160t2pp	0
1289	hdca[p]	1192	ppi	731	FACOAL160t2pp	0
1289	hdca[p]	1370	pmtcoa	731	FACOAL160t2pp	1
135	atp	98	h	732	FACOAL161t2pp	0
135	atp	177	amp	732	FACOAL161t2pp	1
135	atp	696	hdcoa	732	FACOAL161t2pp	0
135	atp	1192	ppi	732	FACOAL161t2pp	0
437	h[p]	98	h	732	FACOAL161t2pp	0
437	h[p]	177	amp	732	FACOAL161t2pp	0
437	h[p]	696	hdcoa	732	FACOAL161t2pp	0
437	h[p]	1192	ppi	732	FACOAL161t2pp	0
927	coa	98	h	732	FACOAL161t2pp	0
927	coa	177	amp	732	FACOAL161t2pp	0
927	coa	696	hdcoa	732	FACOAL161t2pp	1
927	coa	1192	ppi	732	FACOAL161t2pp	0
1290	hdcea[p]	98	h	732	FACOAL161t2pp	0

1290	hdcea[p]	177	amp	732	FACOAL161t2pp	0
1290	hdcea[p]	696	hdcoa	732	FACOAL161t2pp	1
1290	hdcea[p]	1192	ppi	732	FACOAL161t2pp	0
135	atp	98	h	733	FACOAL180t2pp	0
135	atp	177	amp	733	FACOAL180t2pp	1
135	atp	1192	ppi	733	FACOAL180t2pp	0
135	atp	1371	stcoa	733	FACOAL180t2pp	0
437	h[p]	98	h	733	FACOAL180t2pp	0
437	h[p]	177	amp	733	FACOAL180t2pp	0
437	h[p]	1192	ppi	733	FACOAL180t2pp	0
437	h[p]	1371	stcoa	733	FACOAL180t2pp	0
927	coa	98	h	733	FACOAL180t2pp	0
927	coa	177	amp	733	FACOAL180t2pp	0
927	coa	1192	ppi	733	FACOAL180t2pp	0
927	coa	1371	stcoa	733	FACOAL180t2pp	1
1331	ocdca[p]	98	h	733	FACOAL180t2pp	0
1331	ocdca[p]	177	amp	733	FACOAL180t2pp	0
1331	ocdca[p]	1192	ppi	733	FACOAL180t2pp	0
1331	ocdca[p]	1371	stcoa	733	FACOAL180t2pp	1
135	atp	98	h	734	FACOAL181t2pp	0
135	atp	177	amp	734	FACOAL181t2pp	1
135	atp	908	odecoa	734	FACOAL181t2pp	0
135	atp	1192	ppi	734	FACOAL181t2pp	0
437	h[p]	98	h	734	FACOAL181t2pp	0
437	h[p]	177	amp	734	FACOAL181t2pp	0
437	h[p]	908	odecoa	734	FACOAL181t2pp	0
437	h[p]	1192	ppi	734	FACOAL181t2pp	0
927	coa	98	h	734	FACOAL181t2pp	0
927	coa	177	amp	734	FACOAL181t2pp	0
927	coa	908	odecoa	734	FACOAL181t2pp	1
927	coa	1192	ppi	734	FACOAL181t2pp	0
1332	ocdcea[p]	98	h	734	FACOAL181t2pp	0
1332	ocdcea[p]	177	amp	734	FACOAL181t2pp	0
1332	ocdcea[p]	908	odecoa	734	FACOAL181t2pp	1
1332	ocdcea[p]	1192	ppi	734	FACOAL181t2pp	0
135	atp	98	h	735	FACOAL60t2pp	0
135	atp	177	amp	735	FACOAL60t2pp	1
135	atp	1192	ppi	735	FACOAL60t2pp	0
135	atp	1367	hxcoa	735	FACOAL60t2pp	0
437	h[p]	98	h	735	FACOAL60t2pp	0
437	h[p]	177	amp	735	FACOAL60t2pp	0
437	h[p]	1192	ppi	735	FACOAL60t2pp	0
437	h[p]	1367	hxcoa	735	FACOAL60t2pp	0
927	coa	98	h	735	FACOAL60t2pp	0
927	coa	177	amp	735	FACOAL60t2pp	0
927	coa	1192	ppi	735	FACOAL60t2pp	0
927	coa	1367	hxcoa	735	FACOAL60t2pp	1
1128	hxa[p]	98	h	735	FACOAL60t2pp	0
1128	hxa[p]	177	amp	735	FACOAL60t2pp	0
1128	hxa[p]	1192	ppi	735	FACOAL60t2pp	0
1128	hxa[p]	1367	hxcoa	735	FACOAL60t2pp	1
135	atp	98	h	736	FACOAL80t2pp	0
135	atp	177	amp	736	FACOAL80t2pp	1
135	atp	1192	ppi	736	FACOAL80t2pp	0
135	atp	1368	occoa	736	FACOAL80t2pp	0
437	h[p]	98	h	736	FACOAL80t2pp	0

437	h[p]	177	amp	736	FACOAL80t2pp	0
437	h[p]	1192	ppi	736	FACOAL80t2pp	0
437	h[p]	1368	occoa	736	FACOAL80t2pp	0
927	coa	98	h	736	FACOAL80t2pp	0
927	coa	177	amp	736	FACOAL80t2pp	0
927	coa	1192	ppi	736	FACOAL80t2pp	0
927	coa	1368	occoa	736	FACOAL80t2pp	1
1166	octa[p]	98	h	736	FACOAL80t2pp	0
1166	octa[p]	177	amp	736	FACOAL80t2pp	0
1166	octa[p]	1192	ppi	736	FACOAL80t2pp	0
1166	octa[p]	1368	occoa	736	FACOAL80t2pp	1
98	h	520	fadh2	737	FADRx	0
98	h	856	nad	737	FADRx	0
158	fad	520	fadh2	737	FADRx	1
158	fad	856	nad	737	FADRx	0
870	nadh	520	fadh2	737	FADRx	0
870	nadh	856	nad	737	FADRx	1
98	h	459	nadp	738	FADRx2	0
98	h	520	fadh2	738	FADRx2	0
158	fad	459	nadp	738	FADRx2	0
158	fad	520	fadh2	738	FADRx2	1
871	nadph	459	nadp	738	FADRx2	1
871	nadph	520	fadh2	738	FADRx2	0
502	hmgth	98	h	739	FALDH2	0
502	hmgth	870	nadh	739	FALDH2	0
502	hmgth	990	Sfglutth	739	FALDH2	1
856	nad	98	h	739	FALDH2	0
856	nad	870	nadh	739	FALDH2	1
856	nad	990	Sfglutth	739	FALDH2	0
503	fald[e]	504	fald[p]	740	FALDtex	1
504	fald[p]	505	fald	741	FALDtpp	1
505	fald	502	hmgth	742	FALGTHLs	1
751	gthrd	502	hmgth	742	FALGTHLs	1
506	fdp	468	g3p	743	FBA	1
506	fdp	826	dhap	743	FBA	1
109	h2o	499	f6p	744	FBP	0
109	h2o	962	pi	744	FBP	0
506	fdp	499	f6p	744	FBP	1
506	fdp	962	pi	744	FBP	0
507	fuc-L	1109	fcl-L	745	FCI	1
135	atp	98	h	746	FCLK	0
135	atp	281	adp	746	FCLK	1
135	atp	508	fc1p	746	FCLK	0
1109	fcl-L	98	h	746	FCLK	0
1109	fcl-L	281	adp	746	FCLK	0
1109	fcl-L	508	fc1p	746	FCLK	1
508	fc1p	826	dhap	747	FCLPA	1
508	fc1p	1422	lald-L	747	FCLPA	1
509	fe2	98	h	748	FCLT	0
509	fe2	1597	pheme	748	FCLT	0
1346	ppp9	98	h	748	FCLT	0
1346	ppp9	1597	pheme	748	FCLT	0
98	h	437	h[p]	749	FDH4pp	0
98	h	692	co2	749	FDH4pp	0
98	h	1443	q8h2	749	FDH4pp	0
510	for[p]	437	h[p]	749	FDH4pp	0

510	for[p]	692	co2	749	FDH4pp	1
510	for[p]	1443	q8h2	749	FDH4pp	0
1003	q8	437	h[p]	749	FDH4pp	0
1003	q8	692	co2	749	FDH4pp	0
1003	q8	1443	q8h2	749	FDH4pp	1
98	h	437	h[p]	750	FDH5pp	0
98	h	692	co2	750	FDH5pp	0
98	h	867	mql8	750	FDH5pp	0
510	for[p]	437	h[p]	750	FDH5pp	0
510	for[p]	692	co2	750	FDH5pp	1
510	for[p]	867	mql8	750	FDH5pp	0
1282	mqn8	437	h[p]	750	FDH5pp	0
1282	mqn8	692	co2	750	FDH5pp	0
1282	mqn8	867	mql8	750	FDH5pp	1
511	fmnh2	98	h	751	FDMO	0
511	fmnh2	109	h2o	751	FDMO	0
511	fmnh2	538	fmn	751	FDMO	1
511	fmnh2	597	gcald	751	FDMO	0
511	fmnh2	1590	so3	751	FDMO	0
928	o2	98	h	751	FDMO	0
928	o2	109	h2o	751	FDMO	0
928	o2	538	fmn	751	FDMO	0
928	o2	597	gcald	751	FDMO	0
928	o2	1590	so3	751	FDMO	0
1396	isetac	98	h	751	FDMO	0
1396	isetac	109	h2o	751	FDMO	0
1396	isetac	538	fmn	751	FDMO	0
1396	isetac	597	gcald	751	FDMO	1
1396	isetac	1590	so3	751	FDMO	0
511	fmnh2	98	h	752	FDMO2	0
511	fmnh2	109	h2o	752	FDMO2	0
511	fmnh2	505	fald	752	FDMO2	0
511	fmnh2	538	fmn	752	FDMO2	1
511	fmnh2	1590	so3	752	FDMO2	0
928	o2	98	h	752	FDMO2	0
928	o2	109	h2o	752	FDMO2	0
928	o2	505	fald	752	FDMO2	0
928	o2	538	fmn	752	FDMO2	0
928	o2	1590	so3	752	FDMO2	0
1397	mso3	98	h	752	FDMO2	0
1397	mso3	109	h2o	752	FDMO2	0
1397	mso3	505	fald	752	FDMO2	1
1397	mso3	538	fmn	752	FDMO2	0
1397	mso3	1590	so3	752	FDMO2	0
511	fmnh2	98	h	753	FDMO3	0
511	fmnh2	109	h2o	753	FDMO3	0
511	fmnh2	131	acald	753	FDMO3	0
511	fmnh2	538	fmn	753	FDMO3	1
511	fmnh2	1590	so3	753	FDMO3	0
512	ethso3	98	h	753	FDMO3	0
512	ethso3	109	h2o	753	FDMO3	0
512	ethso3	131	acald	753	FDMO3	1
512	ethso3	538	fmn	753	FDMO3	0
512	ethso3	1590	so3	753	FDMO3	0
928	o2	98	h	753	FDMO3	0
928	o2	109	h2o	753	FDMO3	0

928	o2	131	acald	753	FDMO3	0
928	o2	538	fmn	753	FDMO3	0
928	o2	1590	so3	753	FDMO3	0
511	fmnh2	98	h	754	FDMO4	0
511	fmnh2	109	h2o	754	FDMO4	0
511	fmnh2	226	btal	754	FDMO4	0
511	fmnh2	538	fmn	754	FDMO4	1
511	fmnh2	1590	so3	754	FDMO4	0
513	butso3	98	h	754	FDMO4	0
513	butso3	109	h2o	754	FDMO4	0
513	butso3	226	btal	754	FDMO4	1
513	butso3	538	fmn	754	FDMO4	0
513	butso3	1590	so3	754	FDMO4	0
928	o2	98	h	754	FDMO4	0
928	o2	109	h2o	754	FDMO4	0
928	o2	226	btal	754	FDMO4	0
928	o2	538	fmn	754	FDMO4	0
928	o2	1590	so3	754	FDMO4	0
511	fmnh2	98	h	755	FDMO6	0
511	fmnh2	109	h2o	755	FDMO6	0
511	fmnh2	538	fmn	755	FDMO6	1
511	fmnh2	631	glx	755	FDMO6	0
511	fmnh2	1590	so3	755	FDMO6	0
928	o2	98	h	755	FDMO6	0
928	o2	109	h2o	755	FDMO6	0
928	o2	538	fmn	755	FDMO6	0
928	o2	631	glx	755	FDMO6	0
928	o2	1590	so3	755	FDMO6	0
1630	sulfac	98	h	755	FDMO6	0
1630	sulfac	109	h2o	755	FDMO6	0
1630	sulfac	538	fmn	755	FDMO6	0
1630	sulfac	631	glx	755	FDMO6	1
1630	sulfac	1590	so3	755	FDMO6	0
109	h2o	98	h	756	FE2abcpp	0
109	h2o	281	adp	756	FE2abcpp	0
109	h2o	509	fe2	756	FE2abcpp	0
109	h2o	962	pi	756	FE2abcpp	0
135	atp	98	h	756	FE2abcpp	0
135	atp	281	adp	756	FE2abcpp	1
135	atp	509	fe2	756	FE2abcpp	0
135	atp	962	pi	756	FE2abcpp	0
514	fe2[p]	98	h	756	FE2abcpp	0
514	fe2[p]	281	adp	756	FE2abcpp	0
514	fe2[p]	509	fe2	756	FE2abcpp	0
514	fe2[p]	962	pi	756	FE2abcpp	0
437	h[p]	98	h	757	FE2t2pp	0
437	h[p]	509	fe2	757	FE2t2pp	0
514	fe2[p]	98	h	757	FE2t2pp	0
514	fe2[p]	509	fe2	757	FE2t2pp	0
437	h[p]	98	h	758	FE2t3pp	0
437	h[p]	514	fe2[p]	758	FE2t3pp	0
509	fe2	98	h	758	FE2t3pp	0
509	fe2	514	fe2[p]	758	FE2t3pp	0
515	fe2[e]	514	fe2[p]	759	FE2tex	0
514	fe2[p]	509	fe2	760	FE2tpp	0
109	h2o	98	h	761	FE3abcpp	0

109	h2o	281	adp	761	FE3abcpp	0
109	h2o	962	pi	761	FE3abcpp	0
109	h2o	1398	fe3	761	FE3abcpp	0
135	atp	98	h	761	FE3abcpp	0
135	atp	281	adp	761	FE3abcpp	1
135	atp	962	pi	761	FE3abcpp	0
135	atp	1398	fe3	761	FE3abcpp	0
1110	fe3[p]	98	h	761	FE3abcpp	0
1110	fe3[p]	281	adp	761	FE3abcpp	0
1110	fe3[p]	962	pi	761	FE3abcpp	0
1110	fe3[p]	1398	fe3	761	FE3abcpp	0
109	h2o	98	h	762	FE3DCITabcpp	0
109	h2o	164	cit	762	FE3DCITabcpp	0
109	h2o	281	adp	762	FE3DCITabcpp	0
109	h2o	962	pi	762	FE3DCITabcpp	0
109	h2o	1398	fe3	762	FE3DCITabcpp	0
135	atp	98	h	762	FE3DCITabcpp	0
135	atp	164	cit	762	FE3DCITabcpp	0
135	atp	281	adp	762	FE3DCITabcpp	1
135	atp	962	pi	762	FE3DCITabcpp	0
135	atp	1398	fe3	762	FE3DCITabcpp	0
1272	fe3dcit[p]	98	h	762	FE3DCITabcpp	0
1272	fe3dcit[p]	164	cit	762	FE3DCITabcpp	1
1272	fe3dcit[p]	281	adp	762	FE3DCITabcpp	0
1272	fe3dcit[p]	962	pi	762	FE3DCITabcpp	0
1272	fe3dcit[p]	1398	fe3	762	FE3DCITabcpp	0
437	h[p]	98	h	763	FE3DCITtonex	0
437	h[p]	1272	fe3dcit[p]	763	FE3DCITtonex	0
516	fe3dcit[e]	98	h	763	FE3DCITtonex	0
516	fe3dcit[e]	1272	fe3dcit[p]	763	FE3DCITtonex	1
517	fe3dhbzs	417	23dhbzs	764	FE3DHBZR	1
517	fe3dhbzs	1398	fe3	764	FE3DHBZR	0
109	h2o	98	h	765	FE3DHBZSabcpp	0
109	h2o	281	adp	765	FE3DHBZSabcpp	0
109	h2o	517	fe3dhbzs	765	FE3DHBZSabcpp	0
109	h2o	962	pi	765	FE3DHBZSabcpp	0
135	atp	98	h	765	FE3DHBZSabcpp	0
135	atp	281	adp	765	FE3DHBZSabcpp	1
135	atp	517	fe3dhbzs	765	FE3DHBZSabcpp	0
135	atp	962	pi	765	FE3DHBZSabcpp	0
1273	fe3dhbzs[p]	98	h	765	FE3DHBZSabcpp	0
1273	fe3dhbzs[p]	281	adp	765	FE3DHBZSabcpp	0
1273	fe3dhbzs[p]	517	fe3dhbzs	765	FE3DHBZSabcpp	1
1273	fe3dhbzs[p]	962	pi	765	FE3DHBZSabcpp	0
437	h[p]	98	h	766	FE3DHBZStonex	0
437	h[p]	1273	fe3dhbzs[p]	766	FE3DHBZStonex	0
518	fe3dhbzs[e]	98	h	766	FE3DHBZStonex	0
518	fe3dhbzs[e]	1273	fe3dhbzs[p]	766	FE3DHBZStonex	1
109	h2o	98	h	767	FE3HOXabcpp	0
109	h2o	281	adp	767	FE3HOXabcpp	0
109	h2o	521	fe3hox	767	FE3HOXabcpp	0
109	h2o	962	pi	767	FE3HOXabcpp	0
135	atp	98	h	767	FE3HOXabcpp	0
135	atp	281	adp	767	FE3HOXabcpp	1
135	atp	521	fe3hox	767	FE3HOXabcpp	0
135	atp	962	pi	767	FE3HOXabcpp	0

1274	fe3hox[p]	98	h	767	FE3HOXabcpp	0
1274	fe3hox[p]	281	adp	767	FE3HOXabcpp	0
1274	fe3hox[p]	521	fe3hox	767	FE3HOXabcpp	1
1274	fe3hox[p]	962	pi	767	FE3HOXabcpp	0
519	fe3[e]	522	fe3hox[e]	768	FE3HOXexs	0
1275	fe3hox-un[e]	522	fe3hox[e]	768	FE3HOXexs	1
520	fadh2	98	h	769	FE3HOXR1	0
520	fadh2	158	fad	769	FE3HOXR1	1
520	fadh2	509	fe2	769	FE3HOXR1	0
520	fadh2	523	fe3hox-un	769	FE3HOXR1	0
521	fe3hox	98	h	769	FE3HOXR1	0
521	fe3hox	158	fad	769	FE3HOXR1	0
521	fe3hox	509	fe2	769	FE3HOXR1	0
521	fe3hox	523	fe3hox-un	769	FE3HOXR1	1
511	fmnh2	98	h	770	FE3HOXR2	0
511	fmnh2	509	fe2	770	FE3HOXR2	0
511	fmnh2	523	fe3hox-un	770	FE3HOXR2	0
511	fmnh2	538	fmn	770	FE3HOXR2	1
521	fe3hox	98	h	770	FE3HOXR2	0
521	fe3hox	509	fe2	770	FE3HOXR2	0
521	fe3hox	523	fe3hox-un	770	FE3HOXR2	1
521	fe3hox	538	fmn	770	FE3HOXR2	0
521	fe3hox	98	h	771	FE3HOXR3	0
521	fe3hox	509	fe2	771	FE3HOXR3	0
521	fe3hox	523	fe3hox-un	771	FE3HOXR3	1
521	fe3hox	1515	ribflv	771	FE3HOXR3	0
1382	rbflvrd	98	h	771	FE3HOXR3	0
1382	rbflvrd	509	fe2	771	FE3HOXR3	0
1382	rbflvrd	523	fe3hox-un	771	FE3HOXR3	0
1382	rbflvrd	1515	ribflv	771	FE3HOXR3	1
437	h[p]	98	h	772	FE3HOXtonex	0
437	h[p]	1274	fe3hox[p]	772	FE3HOXtonex	0
522	fe3hox[e]	98	h	772	FE3HOXtonex	0
522	fe3hox[e]	1274	fe3hox[p]	772	FE3HOXtonex	1
437	h[p]	98	h	773	FE3HOXUtex	0
437	h[p]	524	fe3hox-un[p]	773	FE3HOXUtex	0
523	fe3hox-un	98	h	773	FE3HOXUtex	0
523	fe3hox-un	524	fe3hox-un[p]	773	FE3HOXUtex	1
437	h[p]	98	h	774	FE3HOXUtpp	0
437	h[p]	1275	fe3hox-un[e]	774	FE3HOXUtpp	0
524	fe3hox-un[p]	98	h	774	FE3HOXUtpp	0
524	fe3hox-un[p]	1275	fe3hox-un[e]	774	FE3HOXUtpp	1
520	fadh2	98	h	775	FE3Ri	0
520	fadh2	158	fad	775	FE3Ri	1
520	fadh2	509	fe2	775	FE3Ri	0
1398	fe3	98	h	775	FE3Ri	0
1398	fe3	158	fad	775	FE3Ri	0
1398	fe3	509	fe2	775	FE3Ri	0
519	fe3[e]	1110	fe3[p]	776	FE3tex	0
109	h2o	98	h	777	FECRMabcpp	0
109	h2o	281	adp	777	FECRMabcpp	0
109	h2o	525	fecrm	777	FECRMabcpp	0
109	h2o	962	pi	777	FECRMabcpp	0
135	atp	98	h	777	FECRMabcpp	0
135	atp	281	adp	777	FECRMabcpp	1
135	atp	525	fecrm	777	FECRMabcpp	0

135	atp	962	pi	777	FECRMabcpp	0
1276	fecrm[p]	98	h	777	FECRMabcpp	0
1276	fecrm[p]	281	adp	777	FECRMabcpp	0
1276	fecrm[p]	525	fecrm	777	FECRMabcpp	1
1276	fecrm[p]	962	pi	777	FECRMabcpp	0
519	fe3[e]	526	fecrm[e]	778	FECRMexs	0
1277	fecrm-un[e]	526	fecrm[e]	778	FECRMexs	1
520	fadh2	98	h	779	FECRMR1	0
520	fadh2	158	fad	779	FECRMR1	1
520	fadh2	509	fe2	779	FECRMR1	0
520	fadh2	528	fecrm-un	779	FECRMR1	0
525	fecrm	98	h	779	FECRMR1	0
525	fecrm	158	fad	779	FECRMR1	0
525	fecrm	509	fe2	779	FECRMR1	0
525	fecrm	528	fecrm-un	779	FECRMR1	1
511	fmnh2	98	h	780	FECRMR2	0
511	fmnh2	509	fe2	780	FECRMR2	0
511	fmnh2	528	fecrm-un	780	FECRMR2	0
511	fmnh2	538	fmn	780	FECRMR2	1
525	fecrm	98	h	780	FECRMR2	0
525	fecrm	509	fe2	780	FECRMR2	0
525	fecrm	528	fecrm-un	780	FECRMR2	1
525	fecrm	538	fmn	780	FECRMR2	0
525	fecrm	98	h	781	FECRMR3	0
525	fecrm	509	fe2	781	FECRMR3	0
525	fecrm	528	fecrm-un	781	FECRMR3	1
525	fecrm	1515	ribflv	781	FECRMR3	0
1382	rbflvrd	98	h	781	FECRMR3	0
1382	rbflvrd	509	fe2	781	FECRMR3	0
1382	rbflvrd	528	fecrm-un	781	FECRMR3	0
1382	rbflvrd	1515	ribflv	781	FECRMR3	1
437	h[p]	98	h	782	FECRMtonex	0
437	h[p]	1276	fecrm[p]	782	FECRMtonex	0
526	fecrm[e]	98	h	782	FECRMtonex	0
526	fecrm[e]	1276	fecrm[p]	782	FECRMtonex	1
437	h[p]	98	h	783	FECRMUtex	0
437	h[p]	1277	fecrm-un[e]	783	FECRMUtex	0
527	fecrm-un[p]	98	h	783	FECRMUtex	0
527	fecrm-un[p]	1277	fecrm-un[e]	783	FECRMUtex	1
437	h[p]	98	h	784	FECRMUtpp	0
437	h[p]	527	fecrm-un[p]	784	FECRMUtpp	0
528	fecrm-un	98	h	784	FECRMUtpp	0
528	fecrm-un	527	fecrm-un[p]	784	FECRMUtpp	1
109	h2o	98	h	785	FEENTERabcpp	0
109	h2o	281	adp	785	FEENTERabcpp	0
109	h2o	492	feenter	785	FEENTERabcpp	0
109	h2o	962	pi	785	FEENTERabcpp	0
135	atp	98	h	785	FEENTERabcpp	0
135	atp	281	adp	785	FEENTERabcpp	1
135	atp	492	feenter	785	FEENTERabcpp	0
135	atp	962	pi	785	FEENTERabcpp	0
1278	feenter[p]	98	h	785	FEENTERabcpp	0
1278	feenter[p]	281	adp	785	FEENTERabcpp	0
1278	feenter[p]	492	feenter	785	FEENTERabcpp	1
1278	feenter[p]	962	pi	785	FEENTERabcpp	0
519	fe3[e]	531	feenter[e]	786	FEENTERexs	0

529	enter[e]	531	feenter[e]	786	FEENTERexs	1
492	feenter	98	h	787	FEENTERR1	0
492	feenter	158	fad	787	FEENTERR1	0
492	feenter	491	enter	787	FEENTERR1	1
492	feenter	509	fe2	787	FEENTERR1	0
520	fadh2	98	h	787	FEENTERR1	0
520	fadh2	158	fad	787	FEENTERR1	1
520	fadh2	491	enter	787	FEENTERR1	0
520	fadh2	509	fe2	787	FEENTERR1	0
492	feenter	98	h	788	FEENTERR2	0
492	feenter	491	enter	788	FEENTERR2	1
492	feenter	509	fe2	788	FEENTERR2	0
492	feenter	538	fmn	788	FEENTERR2	0
511	fmnh2	98	h	788	FEENTERR2	0
511	fmnh2	491	enter	788	FEENTERR2	0
511	fmnh2	509	fe2	788	FEENTERR2	0
511	fmnh2	538	fmn	788	FEENTERR2	1
492	feenter	98	h	789	FEENTERR3	0
492	feenter	491	enter	789	FEENTERR3	1
492	feenter	509	fe2	789	FEENTERR3	0
492	feenter	1515	ribflv	789	FEENTERR3	0
1382	rbflvrd	98	h	789	FEENTERR3	0
1382	rbflvrd	491	enter	789	FEENTERR3	0
1382	rbflvrd	509	fe2	789	FEENTERR3	0
1382	rbflvrd	1515	ribflv	789	FEENTERR3	1
437	h[p]	98	h	790	FEENTERtex	0
437	h[p]	529	enter[e]	790	FEENTERtex	0
530	enter[p]	98	h	790	FEENTERtex	0
530	enter[p]	529	enter[e]	790	FEENTERtex	1
437	h[p]	98	h	791	FEENTERtonex	0
437	h[p]	1278	feenter[p]	791	FEENTERtonex	0
531	feenter[e]	98	h	791	FEENTERtonex	0
531	feenter[e]	1278	feenter[p]	791	FEENTERtonex	1
437	h[p]	98	h	792	FEENTERtpp	0
437	h[p]	530	enter[p]	792	FEENTERtpp	0
491	enter	98	h	792	FEENTERtpp	0
491	enter	530	enter[p]	792	FEENTERtpp	1
109	h2o	98	h	793	FEOXAMabcpp	0
109	h2o	281	adp	793	FEOXAMabcpp	0
109	h2o	532	feoxam	793	FEOXAMabcpp	0
109	h2o	962	pi	793	FEOXAMabcpp	0
135	atp	98	h	793	FEOXAMabcpp	0
135	atp	281	adp	793	FEOXAMabcpp	1
135	atp	532	feoxam	793	FEOXAMabcpp	0
135	atp	962	pi	793	FEOXAMabcpp	0
1279	feoxam[p]	98	h	793	FEOXAMabcpp	0
1279	feoxam[p]	281	adp	793	FEOXAMabcpp	0
1279	feoxam[p]	532	feoxam	793	FEOXAMabcpp	1
1279	feoxam[p]	962	pi	793	FEOXAMabcpp	0
519	fe3[e]	533	feoxam[e]	794	FEOXAMexs	0
1280	feoxam-un[e]	533	feoxam[e]	794	FEOXAMexs	1
520	fadh2	98	h	795	FEOXAMR1	0
520	fadh2	158	fad	795	FEOXAMR1	1
520	fadh2	509	fe2	795	FEOXAMR1	0
520	fadh2	535	feoxam-un	795	FEOXAMR1	0
532	feoxam	98	h	795	FEOXAMR1	0

532	feoxam	158	fad	795	FEOXAMR1	0
532	feoxam	509	fe2	795	FEOXAMR1	0
532	feoxam	535	feoxam-un	795	FEOXAMR1	1
511	fmnh2	98	h	796	FEOXAMR2	0
511	fmnh2	509	fe2	796	FEOXAMR2	0
511	fmnh2	535	feoxam-un	796	FEOXAMR2	0
511	fmnh2	538	fmn	796	FEOXAMR2	1
532	feoxam	98	h	796	FEOXAMR2	0
532	feoxam	509	fe2	796	FEOXAMR2	0
532	feoxam	535	feoxam-un	796	FEOXAMR2	1
532	feoxam	538	fmn	796	FEOXAMR2	0
532	feoxam	98	h	797	FEOXAMR3	0
532	feoxam	509	fe2	797	FEOXAMR3	0
532	feoxam	535	feoxam-un	797	FEOXAMR3	1
532	feoxam	1515	ribflv	797	FEOXAMR3	0
1382	rbflvrd	98	h	797	FEOXAMR3	0
1382	rbflvrd	509	fe2	797	FEOXAMR3	0
1382	rbflvrd	535	feoxam-un	797	FEOXAMR3	0
1382	rbflvrd	1515	ribflv	797	FEOXAMR3	1
437	h[p]	98	h	798	FEOXAMtonex	0
437	h[p]	1279	feoxam[p]	798	FEOXAMtonex	0
533	feoxam[e]	98	h	798	FEOXAMtonex	0
533	feoxam[e]	1279	feoxam[p]	798	FEOXAMtonex	1
437	h[p]	98	h	799	FEOXAMUtex	0
437	h[p]	1280	feoxam-un[e]	799	FEOXAMUtex	0
534	feoxam-un[p]	98	h	799	FEOXAMUtex	0
534	feoxam-un[p]	1280	feoxam-un[e]	799	FEOXAMUtex	1
437	h[p]	98	h	800	FEOXAMUtpp	0
437	h[p]	534	feoxam-un[p]	800	FEOXAMUtpp	0
535	feoxam-un	98	h	800	FEOXAMUtpp	0
535	feoxam-un	534	feoxam-un[p]	800	FEOXAMUtpp	1
437	h[p]	684	h2o[p]	801	FEROpp	0
437	h[p]	1110	fe3[p]	801	FEROpp	0
514	fe2[p]	684	h2o[p]	801	FEROpp	0
514	fe2[p]	1110	fe3[p]	801	FEROpp	0
903	o2[p]	684	h2o[p]	801	FEROpp	0
903	o2[p]	1110	fe3[p]	801	FEROpp	0
109	h2o	568	g6p	802	FFSD	0
109	h2o	1235	fru	802	FFSD	0
1399	suc6p	568	g6p	802	FFSD	1
1399	suc6p	1235	fru	802	FFSD	1
98	h	692	co2	803	FHL	0
98	h	1124	h2	803	FHL	0
536	for	692	co2	803	FHL	1
536	for	1124	h2	803	FHL	0
98	h	459	nadp	804	FLDR	0
98	h	982	fldrd	804	FLDR	0
537	fldox	459	nadp	804	FLDR	0
537	fldox	982	fldrd	804	FLDR	1
871	nadph	459	nadp	804	FLDR	1
871	nadph	982	fldrd	804	FLDR	0
98	h	459	nadp	805	FLVR	0
98	h	1382	rbflvrd	805	FLVR	0
871	nadph	459	nadp	805	FLVR	1
871	nadph	1382	rbflvrd	805	FLVR	0
1515	ribflv	459	nadp	805	FLVR	0

1515	ribflv	1382	rbflvrd	805	FLVR	1
98	h	856	nad	806	FLVRx	0
98	h	1382	rbflvrd	806	FLVRx	0
870	nadh	856	nad	806	FLVRx	1
870	nadh	1382	rbflvrd	806	FLVRx	0
1515	ribflv	856	nad	806	FLVRx	0
1515	ribflv	1382	rbflvrd	806	FLVRx	1
210	10fthf	98	h	807	FMETTRS	0
210	10fthf	1281	fmettrna	807	FMETTRS	1
210	10fthf	1403	thf	807	FMETTRS	1
1400	mettrna	98	h	807	FMETTRS	0
1400	mettrna	1281	fmettrna	807	FMETTRS	1
1400	mettrna	1403	thf	807	FMETTRS	0
98	h	158	fad	808	FMNAT	0
98	h	1192	ppi	808	FMNAT	0
135	atp	158	fad	808	FMNAT	1
135	atp	1192	ppi	808	FMNAT	0
538	fmn	158	fad	808	FMNAT	1
538	fmn	1192	ppi	808	FMNAT	0
98	h	511	fmnh2	809	FMNRx	0
98	h	856	nad	809	FMNRx	0
538	fmn	511	fmnh2	809	FMNRx	1
538	fmn	856	nad	809	FMNRx	0
870	nadh	511	fmnh2	809	FMNRx	0
870	nadh	856	nad	809	FMNRx	1
98	h	459	nadp	810	FMNRx2	0
98	h	511	fmnh2	810	FMNRx2	0
538	fmn	459	nadp	810	FMNRx2	0
538	fmn	511	fmnh2	810	FMNRx2	1
871	nadph	459	nadp	810	FMNRx2	1
871	nadph	511	fmnh2	810	FMNRx2	0
437	h[p]	98	h	811	FORt2pp	0
437	h[p]	536	for	811	FORt2pp	0
510	for[p]	98	h	811	FORt2pp	0
510	for[p]	536	for	811	FORt2pp	1
539	for[e]	510	for[p]	812	FORtex	1
536	for	510	for[p]	813	FORtppi	1
540	fum	1008	succ	814	FRD2	1
540	fum	1282	mqn8	814	FRD2	0
867	mql8	1008	succ	814	FRD2	0
867	mql8	1282	mqn8	814	FRD2	1
245	2dmmql8	560	2dmmq8	815	FRD3	1
245	2dmmql8	1008	succ	815	FRD3	0
540	fum	560	2dmmq8	815	FRD3	0
540	fum	1008	succ	815	FRD3	1
135	atp	98	h	816	FRUK	0
135	atp	281	adp	816	FRUK	1
135	atp	506	fdp	816	FRUK	0
1283	f1p	98	h	816	FRUK	0
1283	f1p	281	adp	816	FRUK	0
1283	f1p	506	fdp	816	FRUK	1
109	h2o	568	g6p	817	FRULYSDG	0
109	h2o	1425	lys-L	817	FRULYSDG	0
541	frulysp	568	g6p	817	FRULYSDG	1
541	frulysp	1425	lys-L	817	FRULYSDG	1
542	psclys	1111	frulys	818	FRULYSE	1

135	atp	98	h	819	FRULYSK	0
135	atp	281	adp	819	FRULYSK	1
135	atp	541	frulysp	819	FRULYSK	0
1111	frulys	98	h	819	FRULYSK	0
1111	frulys	281	adp	819	FRULYSK	0
1111	frulys	541	frulysp	819	FRULYSK	1
437	h[p]	98	h	820	FRULYSt2pp	0
437	h[p]	1111	frulys	820	FRULYSt2pp	0
543	frulys[p]	98	h	820	FRULYSt2pp	0
543	frulys[p]	1111	frulys	820	FRULYSt2pp	1
544	frulys[e]	543	frulys[p]	821	FRULYStex	1
545	fru[p]	499	f6p	822	FRUpts2pp	1
545	fru[p]	1148	pyr	822	FRUpts2pp	0
959	pep	499	f6p	822	FRUpts2pp	0
959	pep	1148	pyr	822	FRUpts2pp	1
545	fru[p]	1148	pyr	823	FRUptspp	0
545	fru[p]	1283	f1p	823	FRUptspp	1
959	pep	1148	pyr	823	FRUptspp	1
959	pep	1283	f1p	823	FRUptspp	0
546	fru[e]	545	fru[p]	824	FRUtex	1
437	h[p]	98	h	825	FRUURt2rpp	0
437	h[p]	1112	fruur	825	FRUURt2rpp	0
547	fruur[p]	98	h	825	FRUURt2rpp	0
547	fruur[p]	1112	fruur	825	FRUURt2rpp	1
548	fruur[e]	547	fruur[p]	826	FRUURtex	1
109	h2o	98	h	827	FTHFD	0
109	h2o	536	for	827	FTHFD	0
109	h2o	1403	thf	827	FTHFD	0
210	10fthf	98	h	827	FTHFD	0
210	10fthf	536	for	827	FTHFD	1
210	10fthf	1403	thf	827	FTHFD	1
549	fuc-L[e]	550	fuc-L[p]	828	FUCtex	1
437	h[p]	98	h	829	FUCtpp	0
437	h[p]	507	fuc-L	829	FUCtpp	0
550	fuc-L[p]	98	h	829	FUCtpp	0
550	fuc-L[p]	507	fuc-L	829	FUCtpp	1
109	h2o	811	mal-L	830	FUM	0
540	fum	811	mal-L	830	FUM	1
437	h[p]	98	h	831	FUMt2_2pp	0
437	h[p]	540	fum	831	FUMt2_2pp	0
551	fum[p]	98	h	831	FUMt2_2pp	0
551	fum[p]	540	fum	831	FUMt2_2pp	1
437	h[p]	98	h	832	FUMt2_3pp	0
437	h[p]	540	fum	832	FUMt2_3pp	0
551	fum[p]	98	h	832	FUMt2_3pp	0
551	fum[p]	540	fum	832	FUMt2_3pp	1
552	fum[e]	551	fum[p]	833	FUMtex	1
128	accoa	98	h	834	G1PACT	0
128	accoa	927	coa	834	G1PACT	1
128	accoa	1039	acgam1p	834	G1PACT	1
931	gam1p	98	h	834	G1PACT	0
931	gam1p	927	coa	834	G1PACT	0
931	gam1p	1039	acgam1p	834	G1PACT	1
553	g1p[p]	606	glc-D[p]	835	G1PPpp	1
553	g1p[p]	1173	pi[p]	835	G1PPpp	0
684	h2o[p]	606	glc-D[p]	835	G1PPpp	0

684	h2o[p]	1173	pi[p]	835	G1PPpp	0
554	g1p[e]	553	g1p[p]	836	G1Ptex	1
98	h	1015	dtdpglu	837	G1PTT	0
98	h	1192	ppi	837	G1PTT	0
555	dttp	1015	dtdpglu	837	G1PTT	1
555	dttp	1192	ppi	837	G1PTT	0
589	g1p	1015	dtdpglu	837	G1PTT	1
589	g1p	1192	ppi	837	G1PTT	0
556	glu1sa	946	5aop	838	G1SAT	1
557	glyc2p[p]	1119	glyc[p]	839	G2PPpp	1
557	glyc2p[p]	1173	pi[p]	839	G2PPpp	0
684	h2o[p]	1119	glyc[p]	839	G2PPpp	0
684	h2o[p]	1173	pi[p]	839	G2PPpp	0
101	ddcaACP	118	ACP	840	G3PAT120	1
101	ddcaACP	200	1ddecg3p	840	G3PAT120	1
558	glyc3p	118	ACP	840	G3PAT120	0
558	glyc3p	200	1ddecg3p	840	G3PAT120	1
558	glyc3p	118	ACP	841	G3PAT140	0
558	glyc3p	201	1tdecg3p	841	G3PAT140	1
1267	myrsACP	118	ACP	841	G3PAT140	1
1267	myrsACP	201	1tdecg3p	841	G3PAT140	1
558	glyc3p	118	ACP	842	G3PAT141	0
558	glyc3p	202	1tdec7eg3p	842	G3PAT141	1
1373	tdeACP	118	ACP	842	G3PAT141	1
1373	tdeACP	202	1tdec7eg3p	842	G3PAT141	1
558	glyc3p	118	ACP	843	G3PAT160	0
558	glyc3p	203	1hdecg3p	843	G3PAT160	1
1374	palmACP	118	ACP	843	G3PAT160	1
1374	palmACP	203	1hdecg3p	843	G3PAT160	1
486	hdeACP	118	ACP	844	G3PAT161	1
486	hdeACP	204	1hdec9eg3p	844	G3PAT161	1
558	glyc3p	118	ACP	844	G3PAT161	0
558	glyc3p	204	1hdec9eg3p	844	G3PAT161	1
558	glyc3p	118	ACP	845	G3PAT180	0
558	glyc3p	205	1odecg3p	845	G3PAT180	1
1375	ocdcaACP	118	ACP	845	G3PAT180	1
1375	ocdcaACP	205	1odecg3p	845	G3PAT180	1
558	glyc3p	118	ACP	846	G3PAT181	0
558	glyc3p	206	1odec11eg3p	846	G3PAT181	1
1376	octeACP	118	ACP	846	G3PAT181	1
1376	octeACP	206	1odec11eg3p	846	G3PAT181	1
109	h2o	98	h	847	G3PCabcpp	0
109	h2o	281	adp	847	G3PCabcpp	0
109	h2o	654	g3pc	847	G3PCabcpp	0
109	h2o	962	pi	847	G3PCabcpp	0
135	atp	98	h	847	G3PCabcpp	0
135	atp	281	adp	847	G3PCabcpp	1
135	atp	654	g3pc	847	G3PCabcpp	0
135	atp	962	pi	847	G3PCabcpp	0
655	g3pc[p]	98	h	847	G3PCabcpp	0
655	g3pc[p]	281	adp	847	G3PCabcpp	0
655	g3pc[p]	654	g3pc	847	G3PCabcpp	1
655	g3pc[p]	962	pi	847	G3PCabcpp	0
559	g3pc[e]	655	g3pc[p]	848	G3PCtex	1
459	nadp	98	h	849	G3PD2	0
459	nadp	826	dhap	849	G3PD2	0

459	nadp	871	nadph	849	G3PD2	1
558	glyc3p	98	h	849	G3PD2	0
558	glyc3p	826	dhap	849	G3PD2	1
558	glyc3p	871	nadph	849	G3PD2	0
558	glyc3p	826	dhap	850	G3PD5	1
558	glyc3p	1443	q8h2	850	G3PD5	0
1003	q8	826	dhap	850	G3PD5	0
1003	q8	1443	q8h2	850	G3PD5	1
558	glyc3p	826	dhap	851	G3PD6	1
558	glyc3p	867	mql8	851	G3PD6	0
1282	mqn8	826	dhap	851	G3PD6	0
1282	mqn8	867	mql8	851	G3PD6	1
558	glyc3p	245	2dmmql8	852	G3PD7	0
558	glyc3p	826	dhap	852	G3PD7	1
560	2dmmq8	245	2dmmql8	852	G3PD7	1
560	2dmmq8	826	dhap	852	G3PD7	0
109	h2o	98	h	853	G3PEabcpp	0
109	h2o	281	adp	853	G3PEabcpp	0
109	h2o	656	g3pe	853	G3PEabcpp	0
109	h2o	962	pi	853	G3PEabcpp	0
135	atp	98	h	853	G3PEabcpp	0
135	atp	281	adp	853	G3PEabcpp	1
135	atp	656	g3pe	853	G3PEabcpp	0
135	atp	962	pi	853	G3PEabcpp	0
657	g3pe[p]	98	h	853	G3PEabcpp	0
657	g3pe[p]	281	adp	853	G3PEabcpp	0
657	g3pe[p]	656	g3pe	853	G3PEabcpp	1
657	g3pe[p]	962	pi	853	G3PEabcpp	0
561	g3pe[e]	657	g3pe[p]	854	G3PEtex	1
109	h2o	98	h	855	G3PGabcpp	0
109	h2o	281	adp	855	G3PGabcpp	0
109	h2o	660	g3pg	855	G3PGabcpp	0
109	h2o	962	pi	855	G3PGabcpp	0
135	atp	98	h	855	G3PGabcpp	0
135	atp	281	adp	855	G3PGabcpp	1
135	atp	660	g3pg	855	G3PGabcpp	0
135	atp	962	pi	855	G3PGabcpp	0
661	g3pg[p]	98	h	855	G3PGabcpp	0
661	g3pg[p]	281	adp	855	G3PGabcpp	0
661	g3pg[p]	660	g3pg	855	G3PGabcpp	1
661	g3pg[p]	962	pi	855	G3PGabcpp	0
562	g3pg[e]	661	g3pg[p]	856	G3PGtex	1
109	h2o	98	h	857	G3PIabcpp	0
109	h2o	281	adp	857	G3PIabcpp	0
109	h2o	662	g3pi	857	G3PIabcpp	0
109	h2o	962	pi	857	G3PIabcpp	0
135	atp	98	h	857	G3PIabcpp	0
135	atp	281	adp	857	G3PIabcpp	1
135	atp	662	g3pi	857	G3PIabcpp	0
135	atp	962	pi	857	G3PIabcpp	0
663	g3pi[p]	98	h	857	G3PIabcpp	0
663	g3pi[p]	281	adp	857	G3PIabcpp	0
663	g3pi[p]	662	g3pi	857	G3PIabcpp	1
663	g3pi[p]	962	pi	857	G3PIabcpp	0
563	g3pi[e]	663	g3pi[p]	858	G3PItex	1
109	h2o	98	h	859	G3PSabcpp	0

109	h2o	281	adp	859	G3PSabcpp	0
109	h2o	658	g3ps	859	G3PSabcpp	0
109	h2o	962	pi	859	G3PSabcpp	0
135	atp	98	h	859	G3PSabcpp	0
135	atp	281	adp	859	G3PSabcpp	1
135	atp	658	g3ps	859	G3PSabcpp	0
135	atp	962	pi	859	G3PSabcpp	0
659	g3ps[p]	98	h	859	G3PSabcpp	0
659	g3ps[p]	281	adp	859	G3PSabcpp	0
659	g3ps[p]	658	g3ps	859	G3PSabcpp	1
659	g3ps[p]	962	pi	859	G3PSabcpp	0
564	g3ps[e]	659	g3ps[p]	860	G3PStex	1
109	h2o	641	glyc	861	G3PT	0
109	h2o	962	pi	861	G3PT	0
558	glyc3p	641	glyc	861	G3PT	1
558	glyc3p	962	pi	861	G3PT	0
565	glu5sa	98	h	862	G5SADs	0
565	glu5sa	109	h2o	862	G5SADs	0
565	glu5sa	920	1pyr5c	862	G5SADs	1
98	h	459	nadp	863	G5SD	0
98	h	565	glu5sa	863	G5SD	0
98	h	962	pi	863	G5SD	0
566	glu5p	459	nadp	863	G5SD	0
566	glu5p	565	glu5sa	863	G5SD	1
566	glu5p	962	pi	863	G5SD	0
871	nadph	459	nadp	863	G5SD	1
871	nadph	565	glu5sa	863	G5SD	0
871	nadph	962	pi	863	G5SD	0
109	h2o	499	f6p	864	G6PDA	0
109	h2o	1160	nh4	864	G6PDA	0
567	gam6p	499	f6p	864	G6PDA	1
567	gam6p	1160	nh4	864	G6PDA	0
459	nadp	98	h	865	G6PDH2r	0
459	nadp	871	nadph	865	G6PDH2r	1
459	nadp	933	6pgl	865	G6PDH2r	0
568	g6p	98	h	865	G6PDH2r	0
568	g6p	871	nadph	865	G6PDH2r	0
568	g6p	933	6pgl	865	G6PDH2r	1
109	h2o	962	pi	866	G6PP	0
109	h2o	1064	glc-D	866	G6PP	0
568	g6p	962	pi	866	G6PP	0
568	g6p	1064	glc-D	866	G6PP	1
569	g6p[p]	568	g6p	867	G6Pt6_2pp	1
569	g6p[p]	1173	pi[p]	867	G6Pt6_2pp	0
962	pi	568	g6p	867	G6Pt6_2pp	0
962	pi	1173	pi[p]	867	G6Pt6_2pp	0
570	g6p[e]	569	g6p[p]	868	G6Ptex	1
571	gal1p[p]	585	gal[p]	869	GAL1PPpp	1
571	gal1p[p]	1173	pi[p]	869	GAL1PPpp	0
684	h2o[p]	585	gal[p]	869	GAL1PPpp	0
684	h2o[p]	1173	pi[p]	869	GAL1PPpp	0
572	gal1p[e]	571	gal1p[p]	870	GAL1Ptex	1
109	h2o	98	h	871	GALabcpp	0
109	h2o	281	adp	871	GALabcpp	0
109	h2o	962	pi	871	GALabcpp	0
109	h2o	1284	gal	871	GALabcpp	0

135	atp	98	h	871	GALabcpp	0
135	atp	281	adp	871	GALabcpp	1
135	atp	962	pi	871	GALabcpp	0
135	atp	1284	gal	871	GALabcpp	0
585	gal[p]	98	h	871	GALabcpp	0
585	gal[p]	281	adp	871	GALabcpp	0
585	gal[p]	962	pi	871	GALabcpp	0
585	gal[p]	1284	gal	871	GALabcpp	1
573	gal-bD[e]	583	gal-bD[p]	872	GALBDtex	1
574	galct-D	109	h2o	873	GALCTD	0
574	galct-D	610	5dh4dglc	873	GALCTD	1
575	galctn-L	98	h	874	GALCTLO	0
575	galctn-L	870	nadh	874	GALCTLO	0
575	galctn-L	1121	tagur	874	GALCTLO	1
856	nad	98	h	874	GALCTLO	0
856	nad	870	nadh	874	GALCTLO	1
856	nad	1121	tagur	874	GALCTLO	0
576	galctn-D	109	h2o	875	GALCTND	0
576	galctn-D	401	2dh3dgal	875	GALCTND	1
437	h[p]	98	h	876	GALCTNLt2pp	0
437	h[p]	575	galctn-L	876	GALCTNLt2pp	0
577	galctn-L[p]	98	h	876	GALCTNLt2pp	0
577	galctn-L[p]	575	galctn-L	876	GALCTNLt2pp	1
578	galctn-L[e]	577	galctn-L[p]	877	GALCTNLtex	1
437	h[p]	98	h	878	GALCTNt2pp	0
437	h[p]	576	galctn-D	878	GALCTNt2pp	0
579	galctn-D[p]	98	h	878	GALCTNt2pp	0
579	galctn-D[p]	576	galctn-D	878	GALCTNt2pp	1
580	galctn-D[e]	579	galctn-D[p]	879	GALCTNtex	1
437	h[p]	98	h	880	GALCTt2rpp	0
437	h[p]	574	galct-D	880	GALCTt2rpp	0
581	galct-D[p]	98	h	880	GALCTt2rpp	0
581	galct-D[p]	574	galct-D	880	GALCTt2rpp	1
582	galct-D[e]	581	galct-D[p]	881	GALCTtex	1
135	atp	98	h	882	GALKr	0
135	atp	281	adp	882	GALKr	1
135	atp	1047	gal1p	882	GALKr	0
1284	gal	98	h	882	GALKr	0
1284	gal	281	adp	882	GALKr	0
1284	gal	1047	gal1p	882	GALKr	1
583	gal-bD[p]	585	gal[p]	883	GALM2pp	1
109	h2o	1064	glc-D	884	GALS3	0
109	h2o	1284	gal	884	GALS3	0
1401	melib	1064	glc-D	884	GALS3	1
1401	melib	1284	gal	884	GALS3	1
584	gicolipa	98	h	885	GALT1	0
584	gicolipa	617	gagicolipa	885	GALT1	1
584	gicolipa	1440	udp	885	GALT1	0
1042	udpg	98	h	885	GALT1	0
1042	udpg	617	gagicolipa	885	GALT1	0
1042	udpg	1440	udp	885	GALT1	1
437	h[p]	98	h	886	GALt2pp	0
437	h[p]	1284	gal	886	GALt2pp	0
585	gal[p]	98	h	886	GALt2pp	0
585	gal[p]	1284	gal	886	GALt2pp	1
586	gal[e]	585	gal[p]	887	GALtex	1

587	galt[p]	623	galt1p	888	GALTptspp	1
587	galt[p]	1148	pyr	888	GALTptspp	0
959	pep	623	galt1p	888	GALTptspp	0
959	pep	1148	pyr	888	GALTptspp	1
588	galt[e]	587	galt[p]	889	GALTtex	1
98	h	1042	udpg	890	GALUi	0
98	h	1192	ppi	890	GALUi	0
589	g1p	1042	udpg	890	GALUi	1
589	g1p	1192	ppi	890	GALUi	0
1447	utp	1042	udpg	890	GALUi	1
1447	utp	1192	ppi	890	GALUi	0
437	h[p]	98	h	891	GALURt2rpp	0
437	h[p]	680	galur	891	GALURt2rpp	0
590	galur[p]	98	h	891	GALURt2rpp	0
590	galur[p]	680	galur	891	GALURt2rpp	1
591	galur[e]	590	galur[p]	892	GALURtex	1
592	gam6p[p]	567	gam6p	893	GAM6Pt6_2pp	1
592	gam6p[p]	1173	pi[p]	893	GAM6Pt6_2pp	0
962	pi	567	gam6p	893	GAM6Pt6_2pp	0
962	pi	1173	pi[p]	893	GAM6Pt6_2pp	0
593	gam6p[e]	592	gam6p[p]	894	GAMAN6Ptex	1
594	gam[p]	567	gam6p	895	GAMptspp	1
594	gam[p]	1148	pyr	895	GAMptspp	0
959	pep	567	gam6p	895	GAMptspp	0
959	pep	1148	pyr	895	GAMptspp	1
595	gam[e]	594	gam[p]	896	GAMtex	1
468	g3p	98	h	897	GAPD	0
468	g3p	870	nadh	897	GAPD	0
468	g3p	1113	13dpg	897	GAPD	1
856	nad	98	h	897	GAPD	0
856	nad	870	nadh	897	GAPD	1
856	nad	1113	13dpg	897	GAPD	0
962	pi	98	h	897	GAPD	0
962	pi	870	nadh	897	GAPD	0
962	pi	1113	13dpg	897	GAPD	0
210	10fthf	98	h	898	GARFT	0
210	10fthf	1114	fgam	898	GARFT	1
210	10fthf	1403	thf	898	GARFT	1
1402	gar	98	h	898	GARFT	0
1402	gar	1114	fgam	898	GARFT	1
1402	gar	1403	thf	898	GARFT	0
135	atp	98	h	899	GART	0
135	atp	281	adp	899	GART	1
135	atp	962	pi	899	GART	0
135	atp	1114	fgam	899	GART	0
536	for	98	h	899	GART	0
536	for	281	adp	899	GART	0
536	for	962	pi	899	GART	0
536	for	1114	fgam	899	GART	1
1402	gar	98	h	899	GART	0
1402	gar	281	adp	899	GART	0
1402	gar	962	pi	899	GART	0
1402	gar	1114	fgam	899	GART	1
596	gbbtn[e]	1115	gbbtn[p]	900	GBBTNtex	1
109	h2o	98	h	901	GCALDD	0
109	h2o	646	glyclt	901	GCALDD	0

109	h2o	870	nadh	901	GCALDD	0
597	gcald	98	h	901	GCALDD	0
597	gcald	646	glyclt	901	GCALDD	1
597	gcald	870	nadh	901	GCALDD	0
856	nad	98	h	901	GCALDD	0
856	nad	646	glyclt	901	GCALDD	0
856	nad	870	nadh	901	GCALDD	1
598	gdpddman	652	gdpofuc	902	GDMANE	1
135	atp	98	h	903	GDPDPK	0
135	atp	177	amp	903	GDPDPK	1
135	atp	1493	ppgpp	903	GDPDPK	0
798	gdp	98	h	903	GDPDPK	0
798	gdp	177	amp	903	GDPDPK	0
798	gdp	1493	ppgpp	903	GDPDPK	1
109	h2o	98	h	904	GDPMNH	0
109	h2o	798	gdp	904	GDPMNH	0
109	h2o	1325	man	904	GDPMNH	0
599	gdpmann	98	h	904	GDPMNH	0
599	gdpmann	798	gdp	904	GDPMNH	1
599	gdpmann	1325	man	904	GDPMNH	1
109	h2o	98	h	905	GDPMNP	0
109	h2o	650	gmp	905	GDPMNP	0
109	h2o	938	man1p	905	GDPMNP	0
599	gdpmann	98	h	905	GDPMNP	0
599	gdpmann	650	gmp	905	GDPMNP	1
599	gdpmann	938	man1p	905	GDPMNP	1
600	gdp[e]	1116	gdp[p]	906	GDPtex	1
499	f6p	567	gam6p	907	GF6PTA	1
499	f6p	624	glu-L	907	GF6PTA	0
625	gln-L	567	gam6p	907	GF6PTA	0
625	gln-L	624	glu-L	907	GF6PTA	1
109	h2o	98	h	908	GGGABADr	0
109	h2o	602	gg4abut	908	GGGABADr	0
109	h2o	871	nadph	908	GGGABADr	0
459	nadp	98	h	908	GGGABADr	0
459	nadp	602	gg4abut	908	GGGABADr	0
459	nadp	871	nadph	908	GGGABADr	1
601	ggbutal	98	h	908	GGGABADr	0
601	ggbutal	602	gg4abut	908	GGGABADr	1
601	ggbutal	871	nadph	908	GGGABADr	0
109	h2o	123	4abut	909	GGGABAH	0
109	h2o	624	glu-L	909	GGGABAH	0
602	gg4abut	123	4abut	909	GGGABAH	1
602	gg4abut	624	glu-L	909	GGGABAH	1
109	h2o	292	h2o2	910	GGPTRCO	0
109	h2o	601	ggbutal	910	GGPTRCO	0
109	h2o	1160	nh4	910	GGPTRCO	0
603	ggptrc	292	h2o2	910	GGPTRCO	0
603	ggptrc	601	ggbutal	910	GGPTRCO	1
603	ggptrc	1160	nh4	910	GGPTRCO	0
928	o2	292	h2o2	910	GGPTRCO	0
928	o2	601	ggbutal	910	GGPTRCO	0
928	o2	1160	nh4	910	GGPTRCO	0
135	atp	98	h	911	GGPTRCS	0
135	atp	281	adp	911	GGPTRCS	1
135	atp	603	ggptrc	911	GGPTRCS	0

135	atp	962	pi	911	GGPTRCS	0
624	glu-L	98	h	911	GGPTRCS	0
624	glu-L	281	adp	911	GGPTRCS	0
624	glu-L	603	ggptrc	911	GGPTRCS	1
624	glu-L	962	pi	911	GGPTRCS	0
1245	ptrc	98	h	911	GGPTRCS	0
1245	ptrc	281	adp	911	GGPTRCS	0
1245	ptrc	603	ggptrc	911	GGPTRCS	1
1245	ptrc	962	pi	911	GGPTRCS	0
604	ser-L	109	h2o	912	GHMT2r	0
604	ser-L	642	gly	912	GHMT2r	1
604	ser-L	848	mlthf	912	GHMT2r	1
1403	thf	109	h2o	912	GHMT2r	0
1403	thf	642	gly	912	GHMT2r	0
1403	thf	848	mlthf	912	GHMT2r	1
135	atp	281	adp	913	GK1	1
135	atp	798	gdp	913	GK1	0
650	gmp	281	adp	913	GK1	0
650	gmp	798	gdp	913	GK1	1
605	glycogen	609	bglycogen	914	GLBRAN2	1
109	h2o	98	h	915	GLCabcpp	0
109	h2o	281	adp	915	GLCabcpp	0
109	h2o	962	pi	915	GLCabcpp	0
109	h2o	1064	glc-D	915	GLCabcpp	0
135	atp	98	h	915	GLCabcpp	0
135	atp	281	adp	915	GLCabcpp	1
135	atp	962	pi	915	GLCabcpp	0
135	atp	1064	glc-D	915	GLCabcpp	0
606	glc-D[p]	98	h	915	GLCabcpp	0
606	glc-D[p]	281	adp	915	GLCabcpp	0
606	glc-D[p]	962	pi	915	GLCabcpp	0
606	glc-D[p]	1064	glc-D	915	GLCabcpp	1
128	accoa	927	coa	916	GLCATr	1
128	accoa	1117	acglc-D	916	GLCATr	1
1064	glc-D	927	coa	916	GLCATr	0
1064	glc-D	1117	acglc-D	916	GLCATr	1
606	glc-D[p]	437	h[p]	917	GLCDpp	0
606	glc-D[p]	607	glcn[p]	917	GLCDpp	1
606	glc-D[p]	1443	q8h2	917	GLCDpp	0
684	h2o[p]	437	h[p]	917	GLCDpp	0
684	h2o[p]	607	glcn[p]	917	GLCDpp	0
684	h2o[p]	1443	q8h2	917	GLCDpp	0
1003	q8	437	h[p]	917	GLCDpp	0
1003	q8	607	glcn[p]	917	GLCDpp	0
1003	q8	1443	q8h2	917	GLCDpp	1
437	h[p]	98	h	918	GLCNt2rpp	0
437	h[p]	1073	glcn	918	GLCNt2rpp	0
607	glcn[p]	98	h	918	GLCNt2rpp	0
607	glcn[p]	1073	glcn	918	GLCNt2rpp	1
608	glcn[e]	607	glcn[p]	919	GLCNtex	1
605	glycogen	589	g1p	920	GLCP	1
962	pi	589	g1p	920	GLCP	0
609	bglycogen	589	g1p	921	GLCP2	1
962	pi	589	g1p	921	GLCP2	0
606	glc-D[p]	568	g6p	922	GLCptspp	1
606	glc-D[p]	1148	pyr	922	GLCptspp	0

959	pep	568	g6p	922	GLCptspp	0
959	pep	1148	pyr	922	GLCptspp	1
610	5dh4dglc	1030	2h3oppan	923	GLCRAL	1
610	5dh4dglc	1148	pyr	923	GLCRAL	1
611	glcr	109	h2o	924	GLCRD	0
611	glcr	610	5dh4dglc	924	GLCRD	1
437	h[p]	98	h	925	GLCRt2rpp	0
437	h[p]	611	glcr	925	GLCRt2rpp	0
612	glcr[p]	98	h	925	GLCRt2rpp	0
612	glcr[p]	611	glcr	925	GLCRt2rpp	1
613	glcr[e]	612	glcr[p]	926	GLCRtex	1
614	adpglc	98	h	927	GLCS1	0
614	adpglc	281	adp	927	GLCS1	0
614	adpglc	605	glycogen	927	GLCS1	1
437	h[p]	98	h	928	GLCt2pp	0
437	h[p]	1064	glc-D	928	GLCt2pp	0
606	glc-D[p]	98	h	928	GLCt2pp	0
606	glc-D[p]	1064	glc-D	928	GLCt2pp	1
615	glc-D[e]	606	glc-D[p]	929	GLCtex	1
615	glc-D[e]	606	glc-D[p]	930	GLCtexi	1
616	icolipa	98	h	931	GLCTR1	0
616	icolipa	584	gicolipa	931	GLCTR1	1
616	icolipa	1440	udp	931	GLCTR1	0
1042	udpg	98	h	931	GLCTR1	0
1042	udpg	584	gicolipa	931	GLCTR1	1
1042	udpg	1440	udp	931	GLCTR1	1
617	gagicolipa	98	h	932	GLCTR2	0
617	gagicolipa	618	ggagicolipa	932	GLCTR2	1
617	gagicolipa	1440	udp	932	GLCTR2	0
1042	udpg	98	h	932	GLCTR2	0
1042	udpg	618	ggagicolipa	932	GLCTR2	1
1042	udpg	1440	udp	932	GLCTR2	1
618	ggagicolipa	98	h	933	GLCTR3	0
618	ggagicolipa	1285	gggagicolipa	933	GLCTR3	1
618	ggagicolipa	1440	udp	933	GLCTR3	0
1042	udpg	98	h	933	GLCTR3	0
1042	udpg	1285	gggagicolipa	933	GLCTR3	1
1042	udpg	1440	udp	933	GLCTR3	1
619	glcur1p[e]	681	glcur1p[p]	934	GLCUR1Ptex	1
437	h[p]	98	h	935	GLCURt2rpp	0
437	h[p]	679	glcur	935	GLCURt2rpp	0
620	glcur[p]	98	h	935	GLCURt2rpp	0
620	glcur[p]	679	glcur	935	GLCURt2rpp	1
621	glcur[e]	620	glcur[p]	936	GLCURtex	1
609	bglycogen	605	glycogen	937	GLDBRAN2	1
98	h	614	adpglc	938	GLGC	0
98	h	1192	ppi	938	GLGC	0
135	atp	614	adpglc	938	GLGC	1
135	atp	1192	ppi	938	GLGC	0
589	g1p	614	adpglc	938	GLGC	1
589	g1p	1192	ppi	938	GLGC	0
109	h2o	98	h	939	GLNabcpp	0
109	h2o	281	adp	939	GLNabcpp	0
109	h2o	625	gln-L	939	GLNabcpp	0
109	h2o	962	pi	939	GLNabcpp	0
135	atp	98	h	939	GLNabcpp	0

135	atp	281	adp	939	GLNabcpp	1
135	atp	625	gln-L	939	GLNabcpp	0
135	atp	962	pi	939	GLNabcpp	0
626	gln-L[p]	98	h	939	GLNabcpp	0
626	gln-L[p]	281	adp	939	GLNabcpp	0
626	gln-L[p]	625	gln-L	939	GLNabcpp	1
626	gln-L[p]	962	pi	939	GLNabcpp	0
135	atp	98	h	940	GLNS	0
135	atp	281	adp	940	GLNS	1
135	atp	625	gln-L	940	GLNS	0
135	atp	962	pi	940	GLNS	0
624	glu-L	98	h	940	GLNS	0
624	glu-L	281	adp	940	GLNS	0
624	glu-L	625	gln-L	940	GLNS	1
624	glu-L	962	pi	940	GLNS	0
1160	nh4	98	h	940	GLNS	0
1160	nh4	281	adp	940	GLNS	0
1160	nh4	625	gln-L	940	GLNS	0
1160	nh4	962	pi	940	GLNS	0
622	gln-L[e]	626	gln-L[p]	941	GLNtex	1
135	atp	177	amp	942	GLNTRS	1
135	atp	1192	ppi	942	GLNTRS	0
135	atp	1598	glntrna	942	GLNTRS	0
625	gln-L	177	amp	942	GLNTRS	0
625	gln-L	1192	ppi	942	GLNTRS	0
625	gln-L	1598	glntrna	942	GLNTRS	1
1631	trnagln	177	amp	942	GLNTRS	0
1631	trnagln	1192	ppi	942	GLNTRS	0
1631	trnagln	1598	glntrna	942	GLNTRS	1
623	galt1p	98	h	943	GLTPD	0
623	galt1p	870	nadh	943	GLTPD	0
623	galt1p	1470	tag6p-D	943	GLTPD	1
856	nad	98	h	943	GLTPD	0
856	nad	870	nadh	943	GLTPD	1
856	nad	1470	tag6p-D	943	GLTPD	0
135	atp	281	adp	944	GLU5K	1
135	atp	566	glu5p	944	GLU5K	0
624	glu-L	281	adp	944	GLU5K	0
624	glu-L	566	glu5p	944	GLU5K	1
109	h2o	98	h	945	GLUabcpp	0
109	h2o	281	adp	945	GLUabcpp	0
109	h2o	624	glu-L	945	GLUabcpp	0
109	h2o	962	pi	945	GLUabcpp	0
135	atp	98	h	945	GLUabcpp	0
135	atp	281	adp	945	GLUabcpp	1
135	atp	624	glu-L	945	GLUabcpp	0
135	atp	962	pi	945	GLUabcpp	0
628	glu-L[p]	98	h	945	GLUabcpp	0
628	glu-L[p]	281	adp	945	GLUabcpp	0
628	glu-L[p]	624	glu-L	945	GLUabcpp	1
628	glu-L[p]	962	pi	945	GLUabcpp	0
123	4abut	125	4abut[p]	946	GLUABUTt7pp	1
123	4abut	624	glu-L	946	GLUABUTt7pp	0
628	glu-L[p]	125	4abut[p]	946	GLUABUTt7pp	0
628	glu-L[p]	624	glu-L	946	GLUABUTt7pp	1
135	atp	98	h	947	GLUCYS	0

135	atp	281	adp	947	GLUCYS	1
135	atp	962	pi	947	GLUCYS	0
135	atp	1404	glucys	947	GLUCYS	0
366	cys-L	98	h	947	GLUCYS	0
366	cys-L	281	adp	947	GLUCYS	0
366	cys-L	962	pi	947	GLUCYS	0
366	cys-L	1404	glucys	947	GLUCYS	1
624	glu-L	98	h	947	GLUCYS	0
624	glu-L	281	adp	947	GLUCYS	0
624	glu-L	962	pi	947	GLUCYS	0
624	glu-L	1404	glucys	947	GLUCYS	1
98	h	123	4abut	948	GLUDC	0
98	h	692	co2	948	GLUDC	0
624	glu-L	123	4abut	948	GLUDC	1
624	glu-L	692	co2	948	GLUDC	0
109	h2o	98	h	949	GLUDy	0
109	h2o	213	akg	949	GLUDy	0
109	h2o	871	nadph	949	GLUDy	0
109	h2o	1160	nh4	949	GLUDy	0
459	nadp	98	h	949	GLUDy	0
459	nadp	213	akg	949	GLUDy	0
459	nadp	871	nadph	949	GLUDy	1
459	nadp	1160	nh4	949	GLUDy	0
624	glu-L	98	h	949	GLUDy	0
624	glu-L	213	akg	949	GLUDy	1
624	glu-L	871	nadph	949	GLUDy	0
624	glu-L	1160	nh4	949	GLUDy	0
109	h2o	624	glu-L	950	GLUN	0
109	h2o	1160	nh4	950	GLUN	0
625	gln-L	624	glu-L	950	GLUN	1
625	gln-L	1160	nh4	950	GLUN	0
626	gln-L[p]	628	glu-L[p]	951	GLUNpp	1
626	gln-L[p]	859	nh4[p]	951	GLUNpp	0
684	h2o[p]	628	glu-L[p]	951	GLUNpp	0
684	h2o[p]	859	nh4[p]	951	GLUNpp	0
109	h2o	624	glu-L	952	GLUPRT	0
109	h2o	1192	ppi	952	GLUPRT	0
109	h2o	1642	pram	952	GLUPRT	0
625	gln-L	624	glu-L	952	GLUPRT	1
625	gln-L	1192	ppi	952	GLUPRT	0
625	gln-L	1642	pram	952	GLUPRT	0
1052	prpp	624	glu-L	952	GLUPRT	0
1052	prpp	1192	ppi	952	GLUPRT	0
1052	prpp	1642	pram	952	GLUPRT	1
627	glu-D	624	glu-L	953	GLUR	1
98	h	459	nadp	954	GLUSy	0
98	h	624	glu-L	954	GLUSy	0
213	akg	459	nadp	954	GLUSy	0
213	akg	624	glu-L	954	GLUSy	1
625	gln-L	459	nadp	954	GLUSy	0
625	gln-L	624	glu-L	954	GLUSy	1
871	nadph	459	nadp	954	GLUSy	1
871	nadph	624	glu-L	954	GLUSy	0
437	h[p]	98	h	955	GLUt2rpp	0
437	h[p]	624	glu-L	955	GLUt2rpp	0
628	glu-L[p]	98	h	955	GLUt2rpp	0

628	glu-L[p]	624	glu-L	955	GLUt2rpp	1
628	glu-L[p]	624	glu-L	956	GLUt4pp	1
628	glu-L[p]	1344	na1	956	GLUt4pp	0
941	na1[p]	624	glu-L	956	GLUt4pp	0
941	na1[p]	1344	na1	956	GLUt4pp	0
629	glu-L[e]	628	glu-L[p]	957	GLUtex	1
98	h	459	nadp	958	GLUTRR	0
98	h	556	glu1sa	958	GLUTRR	0
98	h	1632	trnaglu	958	GLUTRR	0
630	glutrna	459	nadp	958	GLUTRR	0
630	glutrna	556	glu1sa	958	GLUTRR	1
630	glutrna	1632	trnaglu	958	GLUTRR	1
871	nadph	459	nadp	958	GLUTRR	1
871	nadph	556	glu1sa	958	GLUTRR	0
871	nadph	1632	trnaglu	958	GLUTRR	0
135	atp	177	amp	959	GLUTRS	1
135	atp	630	glutrna	959	GLUTRS	0
135	atp	1192	ppi	959	GLUTRS	0
624	glu-L	177	amp	959	GLUTRS	0
624	glu-L	630	glutrna	959	GLUTRS	1
624	glu-L	1192	ppi	959	GLUTRS	0
1632	trnaglu	177	amp	959	GLUTRS	0
1632	trnaglu	630	glutrna	959	GLUTRS	1
1632	trnaglu	1192	ppi	959	GLUTRS	0
98	h	692	co2	960	GLXCL	0
98	h	1030	2h3oppan	960	GLXCL	0
631	glx	692	co2	960	GLXCL	0
631	glx	1030	2h3oppan	960	GLXCL	1
632	glyald[e]	633	glyald[p]	961	GLYALDtex	1
633	glyald[p]	224	glyald	962	GLYALDtpp	1
128	accoa	251	2aobut	963	GLYAT	1
128	accoa	927	coa	963	GLYAT	1
642	gly	251	2aobut	963	GLYAT	1
642	gly	927	coa	963	GLYAT	0
109	h2o	98	h	964	GLYBabcpp	0
109	h2o	281	adp	964	GLYBabcpp	0
109	h2o	962	pi	964	GLYBabcpp	0
109	h2o	1250	glyb	964	GLYBabcpp	0
135	atp	98	h	964	GLYBabcpp	0
135	atp	281	adp	964	GLYBabcpp	1
135	atp	962	pi	964	GLYBabcpp	0
135	atp	1250	glyb	964	GLYBabcpp	0
634	glyb[p]	98	h	964	GLYBabcpp	0
634	glyb[p]	281	adp	964	GLYBabcpp	0
634	glyb[p]	962	pi	964	GLYBabcpp	0
634	glyb[p]	1250	glyb	964	GLYBabcpp	1
437	h[p]	98	h	965	GLYBt2pp	0
437	h[p]	1250	glyb	965	GLYBt2pp	0
634	glyb[p]	98	h	965	GLYBt2pp	0
634	glyb[p]	1250	glyb	965	GLYBt2pp	1
635	glyb[e]	634	glyb[p]	966	GLYBtex	1
636	glyc2p[e]	557	glyc2p[p]	967	GLYC2Ptex	1
109	h2o	98	h	968	GLYC3Pabcpp	0
109	h2o	281	adp	968	GLYC3Pabcpp	0
109	h2o	558	glyc3p	968	GLYC3Pabcpp	0
109	h2o	962	pi	968	GLYC3Pabcpp	0

135	atp	98	h	968	GLYC3Pabcpp	0
135	atp	281	adp	968	GLYC3Pabcpp	1
135	atp	558	glyc3p	968	GLYC3Pabcpp	0
135	atp	962	pi	968	GLYC3Pabcpp	0
637	glyc3p[p]	98	h	968	GLYC3Pabcpp	0
637	glyc3p[p]	281	adp	968	GLYC3Pabcpp	0
637	glyc3p[p]	558	glyc3p	968	GLYC3Pabcpp	1
637	glyc3p[p]	962	pi	968	GLYC3Pabcpp	0
637	glyc3p[p]	558	glyc3p	969	GLYC3Pt6pp	1
637	glyc3p[p]	1173	pi[p]	969	GLYC3Pt6pp	0
962	pi	558	glyc3p	969	GLYC3Pt6pp	0
962	pi	1173	pi[p]	969	GLYC3Pt6pp	0
638	glyc3p[e]	637	glyc3p[p]	970	GLYC3Ptex	1
437	h[p]	98	h	971	GLYCAt2rpp	0
437	h[p]	1118	glyc-R	971	GLYCAt2rpp	0
639	glyc-R[p]	98	h	971	GLYCAt2rpp	0
639	glyc-R[p]	1118	glyc-R	971	GLYCAt2rpp	1
640	glyc-R[e]	639	glyc-R[p]	972	GLYCAtex	1
641	glyc	98	h	973	GLYCDx	0
641	glyc	412	dha	973	GLYCDx	1
641	glyc	870	nadh	973	GLYCDx	0
856	nad	98	h	973	GLYCDx	0
856	nad	412	dha	973	GLYCDx	0
856	nad	870	nadh	973	GLYCDx	1
135	atp	98	h	974	GLYCK	0
135	atp	281	adp	974	GLYCK	1
135	atp	932	3pg	974	GLYCK	0
1118	glyc-R	98	h	974	GLYCK	0
1118	glyc-R	281	adp	974	GLYCK	0
1118	glyc-R	932	3pg	974	GLYCK	1
135	atp	98	h	975	GLYCK2	0
135	atp	281	adp	975	GLYCK2	1
135	atp	489	2pg	975	GLYCK2	0
1118	glyc-R	98	h	975	GLYCK2	0
1118	glyc-R	281	adp	975	GLYCK2	0
1118	glyc-R	489	2pg	975	GLYCK2	1
642	gly	692	co2	976	GLYCL	1
642	gly	848	mlthf	976	GLYCL	1
642	gly	870	nadh	976	GLYCL	0
642	gly	1160	nh4	976	GLYCL	0
856	nad	692	co2	976	GLYCL	0
856	nad	848	mlthf	976	GLYCL	0
856	nad	870	nadh	976	GLYCL	1
856	nad	1160	nh4	976	GLYCL	0
1403	thf	692	co2	976	GLYCL	0
1403	thf	848	mlthf	976	GLYCL	1
1403	thf	870	nadh	976	GLYCL	0
1403	thf	1160	nh4	976	GLYCL	0
98	h	646	glyclt	977	GLYCLTDx	0
98	h	856	nad	977	GLYCLTDx	0
631	glx	646	glyclt	977	GLYCLTDx	1
631	glx	856	nad	977	GLYCLTDx	0
870	nadh	646	glyclt	977	GLYCLTDx	0
870	nadh	856	nad	977	GLYCLTDx	1
98	h	459	nadp	978	GLYCLTDy	0
98	h	646	glyclt	978	GLYCLTDy	0

631	glx	459	nadp	978	GLYCLTDy	0
631	glx	646	glyclt	978	GLYCLTDy	1
871	nadph	459	nadp	978	GLYCLTDy	1
871	nadph	646	glyclt	978	GLYCLTDy	0
437	h[p]	98	h	979	GLYCLTt2rpp	0
437	h[p]	646	glyclt	979	GLYCLTt2rpp	0
643	glyclt[p]	98	h	979	GLYCLTt2rpp	0
643	glyclt[p]	646	glyclt	979	GLYCLTt2rpp	1
643	glyclt[p]	646	glyclt	980	GLYCLTt4pp	1
643	glyclt[p]	1344	na1	980	GLYCLTt4pp	0
941	na1[p]	646	glyclt	980	GLYCLTt4pp	0
941	na1[p]	1344	na1	980	GLYCLTt4pp	0
644	glyclt[e]	643	glyclt[p]	981	GLYCLTtex	1
645	glyc[e]	1119	glyc[p]	982	GLYCtex	1
646	glyclt	631	glx	983	GLYCTO2	1
646	glyclt	1443	q8h2	983	GLYCTO2	0
1003	q8	631	glx	983	GLYCTO2	0
1003	q8	1443	q8h2	983	GLYCTO2	1
646	glyclt	631	glx	984	GLYCTO3	1
646	glyclt	867	mql8	984	GLYCTO3	0
1282	mqn8	631	glx	984	GLYCTO3	0
1282	mqn8	867	mql8	984	GLYCTO3	1
560	2dmmq8	245	2dmmql8	985	GLYCTO4	1
560	2dmmq8	631	glx	985	GLYCTO4	0
646	glyclt	245	2dmmql8	985	GLYCTO4	1
646	glyclt	631	glx	985	GLYCTO4	1
641	glyc	1119	glyc[p]	986	GLYCtpp	1
135	atp	98	h	987	GLYK	1
135	atp	281	adp	987	GLYK	1
135	atp	558	glyc3p	987	GLYK	0
641	glyc	98	h	987	GLYK	0
641	glyc	281	adp	987	GLYK	0
641	glyc	558	glyc3p	987	GLYK	1
109	h2o	98	h	988	GLYOX	0
109	h2o	748	lac-D	988	GLYOX	0
109	h2o	751	gthrd	988	GLYOX	0
1301	lgt-S	98	h	988	GLYOX	0
1301	lgt-S	748	lac-D	988	GLYOX	1
1301	lgt-S	751	gthrd	988	GLYOX	1
109	h2o	98	h	989	GLYOX3	0
109	h2o	748	lac-D	989	GLYOX3	0
1321	mthgxl	98	h	989	GLYOX3	0
1321	mthgxl	748	lac-D	989	GLYOX3	1
437	h[p]	98	h	990	GLYt2pp	0
437	h[p]	642	gly	990	GLYt2pp	0
647	gly[p]	98	h	990	GLYt2pp	0
647	gly[p]	642	gly	990	GLYt2pp	1
647	gly[p]	642	gly	991	GLYt4pp	1
647	gly[p]	1344	na1	991	GLYt4pp	0
941	na1[p]	642	gly	991	GLYt4pp	0
941	na1[p]	1344	na1	991	GLYt4pp	0
648	gly[e]	647	gly[p]	992	GLYtex	1
135	atp	177	amp	993	GLYTRS	1
135	atp	1192	ppi	993	GLYTRS	0
135	atp	1599	glytrna	993	GLYTRS	0
642	gly	177	amp	993	GLYTRS	0

642	gly	1192	ppi	993	GLYTRS	0
642	gly	1599	glytrna	993	GLYTRS	1
1633	trnagly	177	amp	993	GLYTRS	0
1633	trnagly	1192	ppi	993	GLYTRS	0
1633	trnagly	1599	glytrna	993	GLYTRS	1
599	gdpmann	109	h2o	994	GMAND	0
599	gdpmann	598	gdpddman	994	GMAND	1
98	h	196	adphep-DD	995	GMHEPAT	0
98	h	1192	ppi	995	GMHEPAT	0
135	atp	196	adphep-DD	995	GMHEPAT	1
135	atp	1192	ppi	995	GMHEPAT	0
1286	gmhep1p	196	adphep-DD	995	GMHEPAT	1
1286	gmhep1p	1192	ppi	995	GMHEPAT	0
135	atp	98	h	996	GMHEPK	0
135	atp	281	adp	996	GMHEPK	1
135	atp	649	gmhep17bp	996	GMHEPK	0
1352	gmhep7p	98	h	996	GMHEPK	0
1352	gmhep7p	281	adp	996	GMHEPK	0
1352	gmhep7p	649	gmhep17bp	996	GMHEPK	1
109	h2o	962	pi	997	GMHEPPA	0
109	h2o	1286	gmhep1p	997	GMHEPPA	0
649	gmhep17bp	962	pi	997	GMHEPPA	0
649	gmhep17bp	1286	gmhep1p	997	GMHEPPA	1
98	h	459	nadp	998	GMPR	0
98	h	1160	nh4	998	GMPR	0
98	h	1287	imp	998	GMPR	0
650	gmp	459	nadp	998	GMPR	0
650	gmp	1160	nh4	998	GMPR	0
650	gmp	1287	imp	998	GMPR	1
871	nadph	459	nadp	998	GMPR	1
871	nadph	1160	nh4	998	GMPR	0
871	nadph	1287	imp	998	GMPR	0
109	h2o	98	h	999	GMPS2	0
109	h2o	177	amp	999	GMPS2	0
109	h2o	624	glu-L	999	GMPS2	0
109	h2o	650	gmp	999	GMPS2	0
109	h2o	1192	ppi	999	GMPS2	0
135	atp	98	h	999	GMPS2	0
135	atp	177	amp	999	GMPS2	1
135	atp	624	glu-L	999	GMPS2	0
135	atp	650	gmp	999	GMPS2	0
135	atp	1192	ppi	999	GMPS2	0
625	gln-L	98	h	999	GMPS2	0
625	gln-L	177	amp	999	GMPS2	0
625	gln-L	624	glu-L	999	GMPS2	1
625	gln-L	650	gmp	999	GMPS2	0
625	gln-L	1192	ppi	999	GMPS2	0
1445	xmp	98	h	999	GMPS2	0
1445	xmp	177	amp	999	GMPS2	0
1445	xmp	624	glu-L	999	GMPS2	0
1445	xmp	650	gmp	999	GMPS2	1
1445	xmp	1192	ppi	999	GMPS2	0
651	gmp[e]	889	gmp[p]	1000	GMPtex	1
459	nadp	117	ru5p-D	1001	GND	0
459	nadp	692	co2	1001	GND	0
459	nadp	871	nadph	1001	GND	1

484	6pgc	117	ru5p-D	1001	GND	1
484	6pgc	692	co2	1001	GND	1
484	6pgc	871	nadph	1001	GND	0
135	atp	98	h	1002	GNK	0
135	atp	281	adp	1002	GNK	1
135	atp	484	6pgc	1002	GNK	0
1073	glcn	98	h	1002	GNK	0
1073	glcn	281	adp	1002	GNK	0
1073	glcn	484	6pgc	1002	GNK	1
98	h	459	nadp	1003	GOFUCR	0
98	h	1288	gdpfuc	1003	GOFUCR	0
652	gdpofuc	459	nadp	1003	GOFUCR	0
652	gdpofuc	1288	gdpfuc	1003	GOFUCR	1
871	nadph	459	nadp	1003	GOFUCR	1
871	nadph	1288	gdpfuc	1003	GOFUCR	0
109	h2o	98	h	1004	GP4GH	0
109	h2o	798	gdp	1004	GP4GH	0
653	gp4g	98	h	1004	GP4GH	0
653	gp4g	798	gdp	1004	GP4GH	1
109	h2o	98	h	1005	GPDDA1	0
109	h2o	558	glyc3p	1005	GPDDA1	0
109	h2o	1254	chol	1005	GPDDA1	0
654	g3pc	98	h	1005	GPDDA1	0
654	g3pc	558	glyc3p	1005	GPDDA1	1
654	g3pc	1254	chol	1005	GPDDA1	1
655	g3pc[p]	307	chol[p]	1006	GPDDA1pp	1
655	g3pc[p]	437	h[p]	1006	GPDDA1pp	0
655	g3pc[p]	637	glyc3p[p]	1006	GPDDA1pp	1
684	h2o[p]	307	chol[p]	1006	GPDDA1pp	0
684	h2o[p]	437	h[p]	1006	GPDDA1pp	0
684	h2o[p]	637	glyc3p[p]	1006	GPDDA1pp	0
109	h2o	98	h	1007	GPDDA2	0
109	h2o	493	etha	1007	GPDDA2	0
109	h2o	558	glyc3p	1007	GPDDA2	0
656	g3pe	98	h	1007	GPDDA2	0
656	g3pe	493	etha	1007	GPDDA2	1
656	g3pe	558	glyc3p	1007	GPDDA2	1
657	g3pe[p]	437	h[p]	1008	GPDDA2pp	0
657	g3pe[p]	494	etha[p]	1008	GPDDA2pp	1
657	g3pe[p]	637	glyc3p[p]	1008	GPDDA2pp	1
684	h2o[p]	437	h[p]	1008	GPDDA2pp	0
684	h2o[p]	494	etha[p]	1008	GPDDA2pp	0
684	h2o[p]	637	glyc3p[p]	1008	GPDDA2pp	0
109	h2o	98	h	1009	GPDDA3	0
109	h2o	558	glyc3p	1009	GPDDA3	0
109	h2o	604	ser-L	1009	GPDDA3	0
658	g3ps	98	h	1009	GPDDA3	0
658	g3ps	558	glyc3p	1009	GPDDA3	1
658	g3ps	604	ser-L	1009	GPDDA3	1
659	g3ps[p]	437	h[p]	1010	GPDDA3pp	0
659	g3ps[p]	637	glyc3p[p]	1010	GPDDA3pp	1
659	g3ps[p]	1193	ser-L[p]	1010	GPDDA3pp	1
684	h2o[p]	437	h[p]	1010	GPDDA3pp	0
684	h2o[p]	637	glyc3p[p]	1010	GPDDA3pp	0
684	h2o[p]	1193	ser-L[p]	1010	GPDDA3pp	0
109	h2o	98	h	1011	GPDDA4	0

109	h2o	558	glyc3p	1011	GPDDA4	0
109	h2o	641	glyc	1011	GPDDA4	0
660	g3pg	98	h	1011	GPDDA4	0
660	g3pg	558	glyc3p	1011	GPDDA4	1
660	g3pg	641	glyc	1011	GPDDA4	1
661	g3pg[p]	437	h[p]	1012	GPDDA4pp	0
661	g3pg[p]	637	glyc3p[p]	1012	GPDDA4pp	1
661	g3pg[p]	1119	glyc[p]	1012	GPDDA4pp	1
684	h2o[p]	437	h[p]	1012	GPDDA4pp	0
684	h2o[p]	637	glyc3p[p]	1012	GPDDA4pp	0
684	h2o[p]	1119	glyc[p]	1012	GPDDA4pp	0
109	h2o	98	h	1013	GPDDA5	0
109	h2o	558	glyc3p	1013	GPDDA5	0
109	h2o	1296	inost	1013	GPDDA5	0
662	g3pi	98	h	1013	GPDDA5	0
662	g3pi	558	glyc3p	1013	GPDDA5	1
662	g3pi	1296	inost	1013	GPDDA5	1
663	g3pi[p]	437	h[p]	1014	GPDDA5pp	0
663	g3pi[p]	637	glyc3p[p]	1014	GPDDA5pp	1
663	g3pi[p]	723	inost[p]	1014	GPDDA5pp	1
684	h2o[p]	437	h[p]	1014	GPDDA5pp	0
684	h2o[p]	637	glyc3p[p]	1014	GPDDA5pp	0
684	h2o[p]	723	inost[p]	1014	GPDDA5pp	0
664	grdp	697	frdp	1015	GRTT	1
664	grdp	1192	ppi	1015	GRTT	0
726	ipdp	697	frdp	1015	GRTT	1
726	ipdp	1192	ppi	1015	GRTT	0
665	grxox	669	gthox	1016	GRXR	0
665	grxox	925	grxrd	1016	GRXR	1
751	gthrd	669	gthox	1016	GRXR	1
751	gthrd	925	grxrd	1016	GRXR	0
135	atp	98	h	1017	GSNK	0
135	atp	281	adp	1017	GSNK	1
135	atp	650	gmp	1017	GSNK	0
965	gsn	98	h	1017	GSNK	0
965	gsn	281	adp	1017	GSNK	0
965	gsn	650	gmp	1017	GSNK	1
437	h[p]	98	h	1018	GSNt2pp	0
437	h[p]	965	gsn	1018	GSNt2pp	0
666	gsn[p]	98	h	1018	GSNt2pp	0
666	gsn[p]	965	gsn	1018	GSNt2pp	1
667	gsn[e]	666	gsn[p]	1019	GSNtex	1
109	h2o	751	gthrd	1020	GSPMDA	0
109	h2o	1526	spmd	1020	GSPMDA	0
668	gtspmd	751	gthrd	1020	GSPMDA	1
668	gtspmd	1526	spmd	1020	GSPMDA	1
135	atp	98	h	1021	GSPMDS	0
135	atp	281	adp	1021	GSPMDS	1
135	atp	668	gtspmd	1021	GSPMDS	0
135	atp	962	pi	1021	GSPMDS	0
751	gthrd	98	h	1021	GSPMDS	0
751	gthrd	281	adp	1021	GSPMDS	0
751	gthrd	668	gtspmd	1021	GSPMDS	1
751	gthrd	962	pi	1021	GSPMDS	0
1526	spmd	98	h	1021	GSPMDS	0
1526	spmd	281	adp	1021	GSPMDS	0

1526	spmd	668	gtspmd	1021	GSPMDS	1
1526	spmd	962	pi	1021	GSPMDS	0
98	h	459	nadp	1022	GTHOr	0
98	h	751	gthrd	1022	GTHOr	0
669	gthox	459	nadp	1022	GTHOr	0
669	gthox	751	gthrd	1022	GTHOr	1
871	nadph	459	nadp	1022	GTHOr	1
871	nadph	751	gthrd	1022	GTHOr	0
670	gthox[e]	1120	gthox[p]	1023	GTHOXtex	1
109	h2o	98	h	1024	GTHRDabc2pp	0
109	h2o	281	adp	1024	GTHRDabc2pp	0
109	h2o	671	gthrd[p]	1024	GTHRDabc2pp	0
109	h2o	962	pi	1024	GTHRDabc2pp	0
135	atp	98	h	1024	GTHRDabc2pp	0
135	atp	281	adp	1024	GTHRDabc2pp	1
135	atp	671	gthrd[p]	1024	GTHRDabc2pp	0
135	atp	962	pi	1024	GTHRDabc2pp	0
751	gthrd	98	h	1024	GTHRDabc2pp	0
751	gthrd	281	adp	1024	GTHRDabc2pp	0
751	gthrd	671	gthrd[p]	1024	GTHRDabc2pp	1
751	gthrd	962	pi	1024	GTHRDabc2pp	0
109	h2o	98	h	1025	GTHRDabcpp	0
109	h2o	281	adp	1025	GTHRDabcpp	0
109	h2o	751	gthrd	1025	GTHRDabcpp	0
109	h2o	962	pi	1025	GTHRDabcpp	0
135	atp	98	h	1025	GTHRDabcpp	0
135	atp	281	adp	1025	GTHRDabcpp	1
135	atp	751	gthrd	1025	GTHRDabcpp	0
135	atp	962	pi	1025	GTHRDabcpp	0
671	gthrd[p]	98	h	1025	GTHRDabcpp	0
671	gthrd[p]	281	adp	1025	GTHRDabcpp	0
671	gthrd[p]	751	gthrd	1025	GTHRDabcpp	1
671	gthrd[p]	962	pi	1025	GTHRDabcpp	0
671	gthrd[p]	628	glu-L[p]	1026	GTHRDHpp	1
671	gthrd[p]	1093	cgly[p]	1026	GTHRDHpp	1
684	h2o[p]	628	glu-L[p]	1026	GTHRDHpp	0
684	h2o[p]	1093	cgly[p]	1026	GTHRDHpp	0
672	gthrd[e]	671	gthrd[p]	1027	GTHRDtex	1
135	atp	98	h	1028	GTHS	0
135	atp	281	adp	1028	GTHS	1
135	atp	751	gthrd	1028	GTHS	0
135	atp	962	pi	1028	GTHS	0
642	gly	98	h	1028	GTHS	0
642	gly	281	adp	1028	GTHS	0
642	gly	751	gthrd	1028	GTHS	1
642	gly	962	pi	1028	GTHS	0
1404	glucys	98	h	1028	GTHS	0
1404	glucys	281	adp	1028	GTHS	0
1404	glucys	751	gthrd	1028	GTHS	1
1404	glucys	962	pi	1028	GTHS	0
109	h2o	98	h	1029	GTPCI	0
109	h2o	429	ahdt	1029	GTPCI	0
109	h2o	536	for	1029	GTPCI	0
673	gtp	98	h	1029	GTPCI	0
673	gtp	429	ahdt	1029	GTPCI	1
673	gtp	536	for	1029	GTPCI	1

109	h2o	98	h	1030	GTPCII2	0
109	h2o	426	25drapp	1030	GTPCII2	0
109	h2o	536	for	1030	GTPCII2	0
109	h2o	1192	ppi	1030	GTPCII2	0
673	gtp	98	h	1030	GTPCII2	0
673	gtp	426	25drapp	1030	GTPCII2	1
673	gtp	536	for	1030	GTPCII2	1
673	gtp	1192	ppi	1030	GTPCII2	0
109	h2o	98	h	1031	GTPDPDP	0
109	h2o	962	pi	1031	GTPDPDP	0
109	h2o	1493	ppgpp	1031	GTPDPDP	0
674	gdptp	98	h	1031	GTPDPDP	0
674	gdptp	962	pi	1031	GTPDPDP	0
674	gdptp	1493	ppgpp	1031	GTPDPDP	1
135	atp	98	h	1032	GTPDPK	0
135	atp	177	amp	1032	GTPDPK	1
135	atp	674	gdptp	1032	GTPDPK	0
673	gtp	98	h	1032	GTPDPK	0
673	gtp	177	amp	1032	GTPDPK	0
673	gtp	674	gdptp	1032	GTPDPK	1
98	h	1160	nh4	1033	GTPHs	0
98	h	1446	xtp	1033	GTPHs	0
109	h2o	1160	nh4	1033	GTPHs	0
109	h2o	1446	xtp	1033	GTPHs	0
673	gtp	1160	nh4	1033	GTPHs	0
673	gtp	1446	xtp	1033	GTPHs	1
675	gtp[e]	891	gtp[p]	1034	GTPtex	1
98	h	1160	nh4	1035	GUAD	0
98	h	1231	xan	1035	GUAD	0
109	h2o	1160	nh4	1035	GUAD	0
109	h2o	1231	xan	1035	GUAD	0
676	gua	1160	nh4	1035	GUAD	0
676	gua	1231	xan	1035	GUAD	1
676	gua	650	gmp	1036	GUAPRT	1
676	gua	1192	ppi	1036	GUAPRT	0
1052	prpp	650	gmp	1036	GUAPRT	1
1052	prpp	1192	ppi	1036	GUAPRT	0
437	h[p]	98	h	1037	GUAt2pp	0
437	h[p]	676	gua	1037	GUAt2pp	0
677	gua[p]	98	h	1037	GUAt2pp	0
677	gua[p]	676	gua	1037	GUAt2pp	1
678	gua[e]	677	gua[p]	1038	GUAtex	1
677	gua[p]	676	gua	1039	GUAtpp	1
679	glcur	1112	fruur	1040	GUI1	1
680	galur	1121	tagur	1041	GUI2	1
681	glcur1p[p]	620	glcur[p]	1042	GUR1PPpp	1
681	glcur1p[p]	1173	pi[p]	1042	GUR1PPpp	0
684	h2o[p]	620	glcur[p]	1042	GUR1PPpp	0
684	h2o[p]	1173	pi[p]	1042	GUR1PPpp	0
682	h2o2[e]	1122	h2o2[p]	1043	H2O2tex	0
683	h2o[e]	684	h2o[p]	1044	H2Otex	0
684	h2o[p]	109	h2o	1045	H2Otpp	0
685	h2s	98	h	1046	H2SO	0
685	h2s	1600	so4	1046	H2SO	0
928	o2	98	h	1046	H2SO	0
928	o2	1600	so4	1046	H2SO	0

685	h2s	1123	h2s[p]	1047	H2St1pp	0
686	h2s[e]	1123	h2s[p]	1048	H2Stex	0
687	h2[e]	688	h2[p]	1049	H2tex	0
688	h2[p]	1124	h2	1050	H2tpp	0
475	3hbcoa	98	h	1051	HACD1i	0
475	3hbcoa	734	aacoa	1051	HACD1i	1
475	3hbcoa	870	nadh	1051	HACD1i	0
856	nad	98	h	1051	HACD1i	0
856	nad	734	aacoa	1051	HACD1i	0
856	nad	870	nadh	1051	HACD1i	1
476	3hhcoa	98	h	1052	HACD2i	0
476	3hhcoa	735	3ohcoa	1052	HACD2i	1
476	3hhcoa	870	nadh	1052	HACD2i	0
856	nad	98	h	1052	HACD2i	0
856	nad	735	3ohcoa	1052	HACD2i	0
856	nad	870	nadh	1052	HACD2i	1
477	3hocoa	98	h	1053	HACD3i	0
477	3hocoa	736	3oocoa	1053	HACD3i	1
477	3hocoa	870	nadh	1053	HACD3i	0
856	nad	98	h	1053	HACD3i	0
856	nad	736	3oocoa	1053	HACD3i	0
856	nad	870	nadh	1053	HACD3i	1
478	3hdcoa	98	h	1054	HACD4i	0
478	3hdcoa	737	3odcoa	1054	HACD4i	1
478	3hdcoa	870	nadh	1054	HACD4i	0
856	nad	98	h	1054	HACD4i	0
856	nad	737	3odcoa	1054	HACD4i	0
856	nad	870	nadh	1054	HACD4i	1
479	3hddcoa	98	h	1055	HACD5i	0
479	3hddcoa	738	3oddcoa	1055	HACD5i	1
479	3hddcoa	870	nadh	1055	HACD5i	0
856	nad	98	h	1055	HACD5i	0
856	nad	738	3oddcoa	1055	HACD5i	0
856	nad	870	nadh	1055	HACD5i	1
480	3htdcoa	98	h	1056	HACD6i	0
480	3htdcoa	739	3otdcoa	1056	HACD6i	1
480	3htdcoa	870	nadh	1056	HACD6i	0
856	nad	98	h	1056	HACD6i	0
856	nad	739	3otdcoa	1056	HACD6i	0
856	nad	870	nadh	1056	HACD6i	1
481	3hhdcoa	98	h	1057	HACD7i	0
481	3hhdcoa	740	3ohdcoa	1057	HACD7i	1
481	3hhdcoa	870	nadh	1057	HACD7i	0
856	nad	98	h	1057	HACD7i	0
856	nad	740	3ohdcoa	1057	HACD7i	0
856	nad	870	nadh	1057	HACD7i	1
482	3hodcoa	98	h	1058	HACD8i	0
482	3hodcoa	741	3oodcoa	1058	HACD8i	1
482	3hodcoa	870	nadh	1058	HACD8i	0
856	nad	98	h	1058	HACD8i	0
856	nad	741	3oodcoa	1058	HACD8i	0
856	nad	870	nadh	1058	HACD8i	1
689	4hbz	914	3ophb	1059	HBZOPT	1
689	4hbz	1192	ppi	1059	HBZOPT	0
1333	octdp	914	3ophb	1059	HBZOPT	1
1333	octdp	1192	ppi	1059	HBZOPT	0

437	h[p]	79	3hcinnm	1060	HCINNMt2rpp	0
437	h[p]	98	h	1060	HCINNMt2rpp	0
690	3hcinnm[p]	79	3hcinnm	1060	HCINNMt2rpp	1
690	3hcinnm[p]	98	h	1060	HCINNMt2rpp	0
691	3hcinnm[e]	690	3hcinnm[p]	1061	HCINNMtex	1
109	h2o	98	h	1062	HCO3E	0
109	h2o	1560	hco3	1062	HCO3E	0
692	co2	98	h	1062	HCO3E	0
692	co2	1560	hco3	1062	HCO3E	1
178	amet	98	h	1063	HCYSMT	0
178	amet	209	ahcys	1063	HCYSMT	1
178	amet	1433	met-L	1063	HCYSMT	1
693	hcys-L	98	h	1063	HCYSMT	0
693	hcys-L	209	ahcys	1063	HCYSMT	1
693	hcys-L	1433	met-L	1063	HCYSMT	0
693	hcys-L	98	h	1064	HCYSMT2	0
693	hcys-L	1433	met-L	1064	HCYSMT2	1
1405	mmet	98	h	1064	HCYSMT2	0
1405	mmet	1433	met-L	1064	HCYSMT2	1
694	hdca[e]	1289	hdca[p]	1065	HDCAtexi	1
695	hdcea[e]	1290	hdcea[p]	1066	HDCEAtexi	1
696	hdcoa	1291	hdd2coa	1067	HDCOAI	1
109	h2o	1192	ppi	1068	HEMEOS	0
109	h2o	1292	hemeO	1068	HEMEOS	0
697	frdp	1192	ppi	1068	HEMEOS	0
697	frdp	1292	hemeO	1068	HEMEOS	1
1597	pheme	1192	ppi	1068	HEMEOS	0
1597	pheme	1292	hemeO	1068	HEMEOS	1
135	atp	98	h	1069	HEPK1	0
135	atp	281	adp	1069	HEPK1	1
135	atp	1410	phhlipa	1069	HEPK1	0
1406	hhlipa	98	h	1069	HEPK1	0
1406	hhlipa	281	adp	1069	HEPK1	0
1406	hhlipa	1410	phhlipa	1069	HEPK1	1
135	atp	98	h	1070	HEPK2	0
135	atp	281	adp	1070	HEPK2	1
135	atp	1439	phphhlipa	1070	HEPK2	0
1407	hphhlipa	98	h	1070	HEPK2	0
1407	hphhlipa	281	adp	1070	HEPK2	0
1407	hphhlipa	1439	phphhlipa	1070	HEPK2	1
698	adphep-LD	98	h	1071	HEPT1	0
698	adphep-LD	281	adp	1071	HEPT1	1
698	adphep-LD	1409	hlipa	1071	HEPT1	1
1408	lipa	98	h	1071	HEPT1	0
1408	lipa	281	adp	1071	HEPT1	0
1408	lipa	1409	hlipa	1071	HEPT1	1
698	adphep-LD	98	h	1072	HEPT2	0
698	adphep-LD	281	adp	1072	HEPT2	1
698	adphep-LD	1406	hhlipa	1072	HEPT2	1
1409	hlipa	98	h	1072	HEPT2	0
1409	hlipa	281	adp	1072	HEPT2	0
1409	hlipa	1406	hhlipa	1072	HEPT2	1
698	adphep-LD	98	h	1073	HEPT3	0
698	adphep-LD	281	adp	1073	HEPT3	1
698	adphep-LD	1407	hphhlipa	1073	HEPT3	1
1410	phhlipa	98	h	1073	HEPT3	0

1410	phhlipa	281	adp	1073	HEPT3	0
1410	phhlipa	1407	hphhlipa	1073	HEPT3	1
698	adphep-LD	98	h	1074	HEPT4	0
698	adphep-LD	281	adp	1074	HEPT4	1
698	adphep-LD	1389	colipa	1074	HEPT4	1
1285	gggagicolipa	98	h	1074	HEPT4	0
1285	gggagicolipa	281	adp	1074	HEPT4	0
1285	gggagicolipa	1389	colipa	1074	HEPT4	1
135	atp	98	h	1075	HETZK	0
135	atp	281	adp	1075	HETZK	1
135	atp	1293	4mpetz	1075	HETZK	0
699	4mhetz	98	h	1075	HETZK	0
699	4mhetz	281	adp	1075	HETZK	0
699	4mhetz	1293	4mpetz	1075	HETZK	1
135	atp	98	h	1076	HEX1	0
135	atp	281	adp	1076	HEX1	1
135	atp	568	g6p	1076	HEX1	0
1064	glc-D	98	h	1076	HEX1	0
1064	glc-D	281	adp	1076	HEX1	0
1064	glc-D	568	g6p	1076	HEX1	1
135	atp	98	h	1077	HEX4	0
135	atp	281	adp	1077	HEX4	1
135	atp	799	man6p	1077	HEX4	0
1325	man	98	h	1077	HEX4	0
1325	man	281	adp	1077	HEX4	0
1325	man	799	man6p	1077	HEX4	1
135	atp	98	h	1078	HEX7	0
135	atp	281	adp	1078	HEX7	1
135	atp	499	f6p	1078	HEX7	0
1235	fru	98	h	1078	HEX7	0
1235	fru	281	adp	1078	HEX7	0
1235	fru	499	f6p	1078	HEX7	1
437	h[p]	98	h	1079	HEXt2rpp	0
437	h[p]	1418	hxa	1079	HEXt2rpp	0
1128	hxa[p]	98	h	1079	HEXt2rpp	0
1128	hxa[p]	1418	hxa	1079	HEXt2rpp	1
109	h2o	98	h	1080	HG2abcpp	0
109	h2o	281	adp	1080	HG2abcpp	0
109	h2o	962	pi	1080	HG2abcpp	0
109	h2o	1125	hg2[p]	1080	HG2abcpp	0
135	atp	98	h	1080	HG2abcpp	0
135	atp	281	adp	1080	HG2abcpp	1
135	atp	962	pi	1080	HG2abcpp	0
135	atp	1125	hg2[p]	1080	HG2abcpp	0
1411	hg2	98	h	1080	HG2abcpp	0
1411	hg2	281	adp	1080	HG2abcpp	0
1411	hg2	962	pi	1080	HG2abcpp	0
1411	hg2	1125	hg2[p]	1080	HG2abcpp	0
437	h[p]	98	h	1081	HG2t3pp	0
437	h[p]	1125	hg2[p]	1081	HG2t3pp	0
1411	hg2	98	h	1081	HG2t3pp	0
1411	hg2	1125	hg2[p]	1081	HG2t3pp	0
700	hg2[e]	1125	hg2[p]	1082	HG2tex	0
109	h2o	98	h	1083	HISabcpp	0
109	h2o	281	adp	1083	HISabcpp	0
109	h2o	962	pi	1083	HISabcpp	0

109	h2o	1413	his-L	1083	HISabcpp	0
135	atp	98	h	1083	HISabcpp	0
135	atp	281	adp	1083	HISabcpp	1
135	atp	962	pi	1083	HISabcpp	0
135	atp	1413	his-L	1083	HISabcpp	0
1126	his-L[p]	98	h	1083	HISabcpp	0
1126	his-L[p]	281	adp	1083	HISabcpp	0
1126	his-L[p]	962	pi	1083	HISabcpp	0
1126	his-L[p]	1413	his-L	1083	HISabcpp	1
437	h[p]	98	h	1084	HISt2rpp	0
437	h[p]	1413	his-L	1084	HISt2rpp	0
1126	his-L[p]	98	h	1084	HISt2rpp	0
1126	his-L[p]	1413	his-L	1084	HISt2rpp	1
109	h2o	98	h	1085	HISTD	0
109	h2o	870	nadh	1085	HISTD	0
109	h2o	1413	his-L	1085	HISTD	0
856	nad	98	h	1085	HISTD	0
856	nad	870	nadh	1085	HISTD	1
856	nad	1413	his-L	1085	HISTD	0
1294	histd	98	h	1085	HISTD	0
1294	histd	870	nadh	1085	HISTD	0
1294	histd	1413	his-L	1085	HISTD	1
701	his-L[e]	1126	his-L[p]	1086	HIStex	1
109	h2o	962	pi	1087	HISTP	0
109	h2o	1294	histd	1087	HISTP	0
1412	hisp	962	pi	1087	HISTP	0
1412	hisp	1294	histd	1087	HISTP	1
135	atp	177	amp	1088	HISTRS	1
135	atp	1192	ppi	1088	HISTRS	0
135	atp	1601	histrna	1088	HISTRS	0
1413	his-L	177	amp	1088	HISTRS	0
1413	his-L	1192	ppi	1088	HISTRS	0
1413	his-L	1601	histrna	1088	HISTRS	1
1634	trnahis	177	amp	1088	HISTRS	0
1634	trnahis	1192	ppi	1088	HISTRS	0
1634	trnahis	1601	histrna	1088	HISTRS	1
109	h2o	98	h	1089	HKNDDH	0
109	h2o	1008	succ	1089	HKNDDH	0
109	h2o	1451	op4en	1089	HKNDDH	0
1414	hkndd	98	h	1089	HKNDDH	0
1414	hkndd	1008	succ	1089	HKNDDH	1
1414	hkndd	1451	op4en	1089	HKNDDH	1
109	h2o	98	h	1090	HKNTDH	0
109	h2o	540	fum	1090	HKNTDH	0
109	h2o	1451	op4en	1090	HKNTDH	0
1415	hkntd	98	h	1090	HKNTDH	0
1415	hkntd	540	fum	1090	HKNTDH	1
1415	hkntd	1451	op4en	1090	HKNTDH	1
109	h2o	1051	hmbil	1091	HMBS	0
109	h2o	1160	nh4	1091	HMBS	0
1416	ppbng	1051	hmbil	1091	HMBS	1
1416	ppbng	1160	nh4	1091	HMBS	0
135	atp	98	h	1092	HMPK1	0
135	atp	281	adp	1092	HMPK1	1
135	atp	940	4ampm	1092	HMPK1	0
702	4ahmmp	98	h	1092	HMPK1	0

702	4ahmmp	281	adp	1092	HMPK1	0
702	4ahmmp	940	4ampm	1092	HMPK1	1
437	h[p]	98	h	1093	HOMt2pp	0
437	h[p]	1127	hom-L[p]	1093	HOMt2pp	0
710	hom-L	98	h	1093	HOMt2pp	0
710	hom-L	1127	hom-L[p]	1093	HOMt2pp	1
703	hom-L[e]	1127	hom-L[p]	1094	HOMtex	1
704	4h2opntn	131	acald	1095	HOPNTAL	1
704	4h2opntn	1148	pyr	1095	HOPNTAL	1
135	atp	98	h	1096	HPPK2	0
135	atp	177	amp	1096	HPPK2	1
135	atp	1295	6hmhptpp	1096	HPPK2	0
705	6hmhpt	98	h	1096	HPPK2	0
705	6hmhpt	177	amp	1096	HPPK2	0
705	6hmhpt	1295	6hmhptpp	1096	HPPK2	1
706	dhpppn	98	h	1097	HPPPNDO	0
706	dhpppn	1414	hkndd	1097	HPPPNDO	1
928	o2	98	h	1097	HPPPNDO	0
928	o2	1414	hkndd	1097	HPPPNDO	0
437	h[p]	80	3hpppn	1098	HPPPNt2rpp	0
437	h[p]	98	h	1098	HPPPNt2rpp	0
707	3hpppn[p]	80	3hpppn	1098	HPPPNt2rpp	1
707	3hpppn[p]	98	h	1098	HPPPNt2rpp	0
708	3hpppn[e]	707	3hpppn[p]	1099	HPPPNtex	1
709	hpyr	1030	2h3oppan	1100	HPYRI	1
98	h	856	nad	1101	HPYRRx	0
98	h	1118	glyc-R	1101	HPYRRx	0
709	hpyr	856	nad	1101	HPYRRx	0
709	hpyr	1118	glyc-R	1101	HPYRRx	1
870	nadh	856	nad	1101	HPYRRx	1
870	nadh	1118	glyc-R	1101	HPYRRx	0
98	h	459	nadp	1102	HPYRRy	0
98	h	1118	glyc-R	1102	HPYRRy	0
709	hpyr	459	nadp	1102	HPYRRy	0
709	hpyr	1118	glyc-R	1102	HPYRRy	1
871	nadph	459	nadp	1102	HPYRRy	1
871	nadph	1118	glyc-R	1102	HPYRRy	0
459	nadp	98	h	1103	HSDy	0
459	nadp	268	aspsa	1103	HSDy	0
459	nadp	871	nadph	1103	HSDy	1
710	hom-L	98	h	1103	HSDy	0
710	hom-L	268	aspsa	1103	HSDy	1
710	hom-L	871	nadph	1103	HSDy	0
135	atp	98	h	1104	HSK	0
135	atp	281	adp	1104	HSK	1
135	atp	1531	phom	1104	HSK	0
710	hom-L	98	h	1104	HSK	0
710	hom-L	281	adp	1104	HSK	0
710	hom-L	1531	phom	1104	HSK	1
710	hom-L	927	coa	1105	HSST	0
710	hom-L	1524	suchms	1105	HSST	1
838	succoa	927	coa	1105	HSST	1
838	succoa	1524	suchms	1105	HSST	1
624	glu-L	213	akg	1106	HSTPT	1
624	glu-L	1412	hisp	1106	HSTPT	0
1417	imacp	213	akg	1106	HSTPT	0

1417	imacp	1412	hisp	1106	HSTPT	1
711	h[e]	437	h[p]	1107	Htex	0
109	h2o	98	h	1108	HXAND	0
109	h2o	870	nadh	1108	HXAND	0
109	h2o	1231	xan	1108	HXAND	0
713	hxan	98	h	1108	HXAND	0
713	hxan	870	nadh	1108	HXAND	0
713	hxan	1231	xan	1108	HXAND	1
856	nad	98	h	1108	HXAND	0
856	nad	870	nadh	1108	HXAND	1
856	nad	1231	xan	1108	HXAND	0
712	hxa[e]	1128	hxa[p]	1109	HXAtex	1
128	accoa	147	ac	1110	HXCT	1
128	accoa	1367	hxcoa	1110	HXCT	1
1418	hxa	147	ac	1110	HXCT	0
1418	hxa	1367	hxcoa	1110	HXCT	1
713	hxan	1192	ppi	1111	HXPRT	0
713	hxan	1287	imp	1111	HXPRT	1
1052	prpp	1192	ppi	1111	HXPRT	0
1052	prpp	1287	imp	1111	HXPRT	1
98	h	437	h[p]	1112	HYD1pp	0
98	h	1443	q8h2	1112	HYD1pp	0
1003	q8	437	h[p]	1112	HYD1pp	0
1003	q8	1443	q8h2	1112	HYD1pp	1
1124	h2	437	h[p]	1112	HYD1pp	0
1124	h2	1443	q8h2	1112	HYD1pp	0
98	h	437	h[p]	1113	HYD2pp	0
98	h	867	mql8	1113	HYD2pp	0
1124	h2	437	h[p]	1113	HYD2pp	0
1124	h2	867	mql8	1113	HYD2pp	0
1282	mqn8	437	h[p]	1113	HYD2pp	0
1282	mqn8	867	mql8	1113	HYD2pp	1
98	h	245	2dmmql8	1114	HYD3pp	0
98	h	437	h[p]	1114	HYD3pp	0
560	2dmmq8	245	2dmmql8	1114	HYD3pp	1
560	2dmmq8	437	h[p]	1114	HYD3pp	0
1124	h2	245	2dmmql8	1114	HYD3pp	0
1124	h2	437	h[p]	1114	HYD3pp	0
109	h2o	962	pi	1115	HYPOE	0
109	h2o	1509	pydam	1115	HYPOE	0
1247	pyam5p	962	pi	1115	HYPOE	0
1247	pyam5p	1509	pydam	1115	HYPOE	1
714	hxan[e]	715	hxan[p]	1116	HYXNtex	1
715	hxan[p]	713	hxan	1117	HYXNtpp	1
459	nadp	213	akg	1118	ICDHyr	0
459	nadp	692	co2	1118	ICDHyr	0
459	nadp	871	nadph	1118	ICDHyr	1
716	icit	213	akg	1118	ICDHyr	1
716	icit	692	co2	1118	ICDHyr	0
716	icit	871	nadph	1118	ICDHyr	0
173	chor	991	ichor	1119	ICHORS	1
173	chor	991	ichor	1120	ICHORSi	1
109	h2o	415	23ddhb	1121	ICHORT	0
109	h2o	1148	pyr	1121	ICHORT	0
991	ichor	415	23ddhb	1121	ICHORT	1
991	ichor	1148	pyr	1121	ICHORT	1

716	icit	631	glx	1122	ICL	1
716	icit	1008	succ	1122	ICL	1
98	h	856	nad	1123	IDOND	0
98	h	1129	idon-L	1123	IDOND	0
113	5dglcn	856	nad	1123	IDOND	0
113	5dglcn	1129	idon-L	1123	IDOND	1
870	nadh	856	nad	1123	IDOND	1
870	nadh	1129	idon-L	1123	IDOND	0
98	h	459	nadp	1124	IDOND2	0
98	h	1129	idon-L	1124	IDOND2	0
113	5dglcn	459	nadp	1124	IDOND2	0
113	5dglcn	1129	idon-L	1124	IDOND2	1
871	nadph	459	nadp	1124	IDOND2	1
871	nadph	1129	idon-L	1124	IDOND2	0
437	h[p]	98	h	1125	IDONt2rpp	0
437	h[p]	1129	idon-L	1125	IDONt2rpp	0
1130	idon-L[p]	98	h	1125	IDONt2rpp	0
1130	idon-L[p]	1129	idon-L	1125	IDONt2rpp	1
717	idon-L[e]	1130	idon-L[p]	1126	IDONtex	1
625	gln-L	98	h	1127	IG3PS	0
625	gln-L	624	glu-L	1127	IG3PS	1
625	gln-L	718	eig3p	1127	IG3PS	0
625	gln-L	1080	aicar	1127	IG3PS	0
1179	prlp	98	h	1127	IG3PS	0
1179	prlp	624	glu-L	1127	IG3PS	0
1179	prlp	718	eig3p	1127	IG3PS	1
1179	prlp	1080	aicar	1127	IG3PS	1
718	eig3p	109	h2o	1128	IGPDH	0
718	eig3p	1417	imacp	1128	IGPDH	1
98	h	109	h2o	1129	IGPS	0
98	h	692	co2	1129	IGPS	0
98	h	1027	3ig3p	1129	IGPS	0
719	2cpr5p	109	h2o	1129	IGPS	0
719	2cpr5p	692	co2	1129	IGPS	0
719	2cpr5p	1027	3ig3p	1129	IGPS	1
109	h2o	98	h	1130	ILEabcpp	0
109	h2o	281	adp	1130	ILEabcpp	0
109	h2o	962	pi	1130	ILEabcpp	0
109	h2o	1419	ile-L	1130	ILEabcpp	0
135	atp	98	h	1130	ILEabcpp	0
135	atp	281	adp	1130	ILEabcpp	1
135	atp	962	pi	1130	ILEabcpp	0
135	atp	1419	ile-L	1130	ILEabcpp	0
1132	ile-L[p]	98	h	1130	ILEabcpp	0
1132	ile-L[p]	281	adp	1130	ILEabcpp	0
1132	ile-L[p]	962	pi	1130	ILEabcpp	0
1132	ile-L[p]	1419	ile-L	1130	ILEabcpp	1
437	h[p]	98	h	1131	ILEt2rpp	0
437	h[p]	1419	ile-L	1131	ILEt2rpp	0
1132	ile-L[p]	98	h	1131	ILEt2rpp	0
1132	ile-L[p]	1419	ile-L	1131	ILEt2rpp	1
213	akg	624	glu-L	1132	ILETA	1
213	akg	1131	3mop	1132	ILETA	0
1419	ile-L	624	glu-L	1132	ILETA	0
1419	ile-L	1131	3mop	1132	ILETA	1
720	ile-L[e]	1132	ile-L[p]	1133	ILEtex	1

135	atp	177	amp	1134	ILETRS	1
135	atp	1192	ppi	1134	ILETRS	0
135	atp	1602	iletrna	1134	ILETRS	0
1419	ile-L	177	amp	1134	ILETRS	0
1419	ile-L	1192	ppi	1134	ILETRS	0
1419	ile-L	1602	iletrna	1134	ILETRS	1
1635	trnaile	177	amp	1134	ILETRS	0
1635	trnaile	1192	ppi	1134	ILETRS	0
1635	trnaile	1602	iletrna	1134	ILETRS	1
109	h2o	1083	fprica	1135	IMPC	0
1287	imp	1083	fprica	1135	IMPC	1
109	h2o	98	h	1136	IMPD	0
109	h2o	870	nadh	1136	IMPD	0
109	h2o	1445	xmp	1136	IMPD	0
856	nad	98	h	1136	IMPD	0
856	nad	870	nadh	1136	IMPD	1
856	nad	1445	xmp	1136	IMPD	0
1287	imp	98	h	1136	IMPD	0
1287	imp	870	nadh	1136	IMPD	0
1287	imp	1445	xmp	1136	IMPD	1
721	imp[e]	1133	imp[p]	1137	IMPtex	1
98	h	437	h[p]	1138	INDOLEt2pp	0
98	h	1134	indole[p]	1138	INDOLEt2pp	0
1028	indole	437	h[p]	1138	INDOLEt2pp	0
1028	indole	1134	indole[p]	1138	INDOLEt2pp	1
437	h[p]	98	h	1139	INDOLEt2rpp	0
437	h[p]	1028	indole	1139	INDOLEt2rpp	0
1134	indole[p]	98	h	1139	INDOLEt2rpp	0
1134	indole[p]	1028	indole	1139	INDOLEt2rpp	1
722	indole[e]	1134	indole[p]	1140	INDOLEtex	1
723	inost[p]	1296	inost	1141	INOSTt4pp	1
723	inost[p]	1344	na1	1141	INOSTt4pp	0
941	na1[p]	1296	inost	1141	INOSTt4pp	0
941	na1[p]	1344	na1	1141	INOSTt4pp	0
109	h2o	713	hxan	1142	INSH	0
109	h2o	1361	rib-D	1142	INSH	0
967	ins	713	hxan	1142	INSH	1
967	ins	1361	rib-D	1142	INSH	1
135	atp	98	h	1143	INSK	0
135	atp	281	adp	1143	INSK	1
135	atp	1287	imp	1143	INSK	0
967	ins	98	h	1143	INSK	0
967	ins	281	adp	1143	INSK	0
967	ins	1287	imp	1143	INSK	1
437	h[p]	98	h	1144	INSt2pp	0
437	h[p]	967	ins	1144	INSt2pp	0
1135	ins[p]	98	h	1144	INSt2pp	0
1135	ins[p]	967	ins	1144	INSt2pp	1
437	h[p]	98	h	1145	INSt2rpp	0
437	h[p]	967	ins	1145	INSt2rpp	0
1135	ins[p]	98	h	1145	INSt2rpp	0
1135	ins[p]	967	ins	1145	INSt2rpp	1
724	ins[e]	1135	ins[p]	1146	INStex	1
725	inost[e]	723	inost[p]	1147	INSTtex	1
726	ipdp	444	dmpp	1148	IPDDI	1
98	h	109	h2o	1149	IPDPS	0

98	h	726	ipdp	1149	IPDPS	0
98	h	856	nad	1149	IPDPS	0
870	nadh	109	h2o	1149	IPDPS	0
870	nadh	726	ipdp	1149	IPDPS	0
870	nadh	856	nad	1149	IPDPS	1
1319	h2mb4p	109	h2o	1149	IPDPS	0
1319	h2mb4p	726	ipdp	1149	IPDPS	1
1319	h2mb4p	856	nad	1149	IPDPS	0
727	3c2hmp	98	h	1150	IPMD	0
727	3c2hmp	870	nadh	1150	IPMD	0
727	3c2hmp	911	3c4mop	1150	IPMD	1
856	nad	98	h	1150	IPMD	0
856	nad	870	nadh	1150	IPMD	1
856	nad	911	3c4mop	1150	IPMD	0
727	3c2hmp	109	h2o	1151	IPPMIa	0
727	3c2hmp	728	2ippm	1151	IPPMIa	1
109	h2o	1136	3c3hmp	1152	IPPMIb	0
728	2ippm	1136	3c3hmp	1152	IPPMIb	1
109	h2o	98	h	1153	IPPS	0
109	h2o	927	coa	1153	IPPS	0
109	h2o	1136	3c3hmp	1153	IPPS	0
128	accoa	98	h	1153	IPPS	0
128	accoa	927	coa	1153	IPPS	1
128	accoa	1136	3c3hmp	1153	IPPS	1
729	3mob	98	h	1153	IPPS	0
729	3mob	927	coa	1153	IPPS	0
729	3mob	1136	3c3hmp	1153	IPPS	1
109	h2o	98	h	1154	ISETACabcpp	0
109	h2o	281	adp	1154	ISETACabcpp	0
109	h2o	962	pi	1154	ISETACabcpp	0
109	h2o	1396	isetac	1154	ISETACabcpp	0
135	atp	98	h	1154	ISETACabcpp	0
135	atp	281	adp	1154	ISETACabcpp	1
135	atp	962	pi	1154	ISETACabcpp	0
135	atp	1396	isetac	1154	ISETACabcpp	0
1137	isetac[p]	98	h	1154	ISETACabcpp	0
1137	isetac[p]	281	adp	1154	ISETACabcpp	0
1137	isetac[p]	962	pi	1154	ISETACabcpp	0
1137	isetac[p]	1396	isetac	1154	ISETACabcpp	1
730	isetac[e]	1137	isetac[p]	1155	ISETACtex	1
109	h2o	98	h	1156	K2L4Aabcpp	0
109	h2o	281	adp	1156	K2L4Aabcpp	0
109	h2o	731	kdo2lipid4[p]	1156	K2L4Aabcpp	0
109	h2o	962	pi	1156	K2L4Aabcpp	0
135	atp	98	h	1156	K2L4Aabcpp	0
135	atp	281	adp	1156	K2L4Aabcpp	1
135	atp	731	kdo2lipid4[p]	1156	K2L4Aabcpp	0
135	atp	962	pi	1156	K2L4Aabcpp	0
1393	kdo2lipid4	98	h	1156	K2L4Aabcpp	0
1393	kdo2lipid4	281	adp	1156	K2L4Aabcpp	0
1393	kdo2lipid4	731	kdo2lipid4[p]	1156	K2L4Aabcpp	1
1393	kdo2lipid4	962	pi	1156	K2L4Aabcpp	0
731	kdo2lipid4[p]	1297	kdo2lipid4[e]	1157	K2L4Atex	1
109	h2o	98	h	1158	Kabcpp	0
109	h2o	281	adp	1158	Kabcpp	0
109	h2o	962	pi	1158	Kabcpp	0

109	h2o	1420	k	1158	Kabcpp	0
135	atp	98	h	1158	Kabcpp	0
135	atp	281	adp	1158	Kabcpp	1
135	atp	962	pi	1158	Kabcpp	0
135	atp	1420	k	1158	Kabcpp	0
1139	k[p]	98	h	1158	Kabcpp	0
1139	k[p]	281	adp	1158	Kabcpp	0
1139	k[p]	962	pi	1158	Kabcpp	0
1139	k[p]	1420	k	1158	Kabcpp	0
410	23dhmb	98	h	1159	KARA1	0
410	23dhmb	871	nadph	1159	KARA1	0
410	23dhmb	1138	alac-S	1159	KARA1	1
459	nadp	98	h	1159	KARA1	0
459	nadp	871	nadph	1159	KARA1	1
459	nadp	1138	alac-S	1159	KARA1	0
98	h	411	23dhmp	1160	KARA2	0
98	h	459	nadp	1160	KARA2	0
732	2ahbut	411	23dhmp	1160	KARA2	1
732	2ahbut	459	nadp	1160	KARA2	0
871	nadph	411	23dhmp	1160	KARA2	0
871	nadph	459	nadp	1160	KARA2	1
98	h	95	actACP	1161	KAS14	0
98	h	118	ACP	1161	KAS14	0
98	h	692	co2	1161	KAS14	0
733	acACP	95	actACP	1161	KAS14	1
733	acACP	118	ACP	1161	KAS14	1
733	acACP	692	co2	1161	KAS14	0
1363	malACP	95	actACP	1161	KAS14	1
1363	malACP	118	ACP	1161	KAS14	1
1363	malACP	692	co2	1161	KAS14	0
98	h	95	actACP	1162	KAS15	0
98	h	692	co2	1162	KAS15	0
98	h	927	coa	1162	KAS15	0
128	accoa	95	actACP	1162	KAS15	1
128	accoa	692	co2	1162	KAS15	0
128	accoa	927	coa	1162	KAS15	1
1363	malACP	95	actACP	1162	KAS15	1
1363	malACP	692	co2	1162	KAS15	0
1363	malACP	927	coa	1162	KAS15	0
734	aacoa	128	accoa	1163	KAT1	1
927	coa	128	accoa	1163	KAT1	1
735	3ohcoa	128	accoa	1164	KAT2	1
735	3ohcoa	157	btcoa	1164	KAT2	1
927	coa	128	accoa	1164	KAT2	0
927	coa	157	btcoa	1164	KAT2	1
736	3oocoa	128	accoa	1165	KAT3	1
736	3oocoa	1367	hxcoa	1165	KAT3	1
927	coa	128	accoa	1165	KAT3	0
927	coa	1367	hxcoa	1165	KAT3	1
737	3odcoa	128	accoa	1166	KAT4	1
737	3odcoa	1368	occoa	1166	KAT4	1
927	coa	128	accoa	1166	KAT4	0
927	coa	1368	occoa	1166	KAT4	1
738	3oddcoa	128	accoa	1167	KAT5	1
738	3oddcoa	159	dcacoa	1167	KAT5	1
927	coa	128	accoa	1167	KAT5	0

927	coa	159	dcacoa	1167	KAT5	1
739	3otdcoa	128	accoa	1168	KAT6	1
739	3otdcoa	160	ddcacoa	1168	KAT6	1
927	coa	128	accoa	1168	KAT6	0
927	coa	160	ddcacoa	1168	KAT6	1
740	3ohdcoa	128	accoa	1169	KAT7	1
740	3ohdcoa	1369	tdcoa	1169	KAT7	1
927	coa	128	accoa	1169	KAT7	0
927	coa	1369	tdcoa	1169	KAT7	1
741	3oodcoa	128	accoa	1170	KAT8	1
741	3oodcoa	1370	pmtcoa	1170	KAT8	1
927	coa	128	accoa	1170	KAT8	0
927	coa	1370	pmtcoa	1170	KAT8	1
392	ctp	843	ckdo	1171	KDOCT2	1
392	ctp	1192	ppi	1171	KDOCT2	0
1298	kdo	843	ckdo	1171	KDOCT2	1
1298	kdo	1192	ppi	1171	KDOCT2	0
109	h2o	962	pi	1172	KDOPP	0
109	h2o	1298	kdo	1172	KDOPP	0
1299	kdo8p	962	pi	1172	KDOPP	0
1299	kdo8p	1298	kdo	1172	KDOPP	1
109	h2o	962	pi	1173	KDOPS	0
109	h2o	1299	kdo8p	1173	KDOPS	0
742	ara5p	962	pi	1173	KDOPS	0
742	ara5p	1299	kdo8p	1173	KDOPS	1
959	pep	962	pi	1173	KDOPS	0
959	pep	1299	kdo8p	1173	KDOPS	1
98	h	692	co2	1174	KG6PDC	0
98	h	1058	xu5p-L	1174	KG6PDC	0
743	3dhgulnp	692	co2	1174	KG6PDC	0
743	3dhgulnp	1058	xu5p-L	1174	KG6PDC	1
437	h[p]	98	h	1175	Kt2pp	0
437	h[p]	1420	k	1175	Kt2pp	0
1139	k[p]	98	h	1175	Kt2pp	0
1139	k[p]	1420	k	1175	Kt2pp	0
437	h[p]	98	h	1176	Kt3pp	0
437	h[p]	1139	k[p]	1176	Kt3pp	0
1420	k	98	h	1176	Kt3pp	0
1420	k	1139	k[p]	1176	Kt3pp	0
744	k[e]	1139	k[p]	1177	Ktex	0
337	colipa[p]	162	acolipa[p]	1178	LA4NTpp	1
337	colipa[p]	1040	udcpp[p]	1178	LA4NTpp	0
1359	uLa4n[p]	162	acolipa[p]	1178	LA4NTpp	1
1359	uLa4n[p]	1040	udcpp[p]	1178	LA4NTpp	1
109	h2o	1064	glc-D	1179	LACZ	0
109	h2o	1284	gal	1179	LACZ	0
1421	lcts	1064	glc-D	1179	LACZ	1
1421	lcts	1284	gal	1179	LACZ	1
684	h2o[p]	585	gal[p]	1180	LACZpp	0
684	h2o[p]	606	glc-D[p]	1180	LACZpp	0
1140	lcts[p]	585	gal[p]	1180	LACZpp	1
1140	lcts[p]	606	glc-D[p]	1180	LACZpp	1
109	h2o	221	LalaDglu	1181	LADGMDH	0
109	h2o	389	26dap-M	1181	LADGMDH	0
745	LalaDgluMdap	221	LalaDglu	1181	LADGMDH	1
745	LalaDgluMdap	389	26dap-M	1181	LADGMDH	1

98	h	856	nad	1182	LALDO2x	0
98	h	1300	lald-D	1182	LALDO2x	0
870	nadh	856	nad	1182	LALDO2x	1
870	nadh	1300	lald-D	1182	LALDO2x	0
1321	mthgxl	856	nad	1182	LALDO2x	0
1321	mthgxl	1300	lald-D	1182	LALDO2x	1
109	h2o	222	ala-L	1183	LALGP	0
109	h2o	624	glu-L	1183	LALGP	0
746	LalaLglu	222	ala-L	1183	LALGP	1
746	LalaLglu	624	glu-L	1183	LALGP	1
109	h2o	98	h	1184	LCADi	0
109	h2o	754	lac-L	1184	LCADi	0
109	h2o	870	nadh	1184	LCADi	0
856	nad	98	h	1184	LCADi	0
856	nad	754	lac-L	1184	LCADi	0
856	nad	870	nadh	1184	LCADi	1
1422	lald-L	98	h	1184	LCADi	0
1422	lald-L	754	lac-L	1184	LCADi	1
1422	lald-L	870	nadh	1184	LCADi	0
98	h	856	nad	1185	LCARR	0
98	h	1069	12ppd-R	1185	LCARR	0
870	nadh	856	nad	1185	LCARR	1
870	nadh	1069	12ppd-R	1185	LCARR	0
1300	lald-D	856	nad	1185	LCARR	0
1300	lald-D	1069	12ppd-R	1185	LCARR	1
98	h	856	nad	1186	LCARS	0
98	h	1070	12ppd-S	1186	LCARS	0
870	nadh	856	nad	1186	LCARS	1
870	nadh	1070	12ppd-S	1186	LCARS	0
1422	lald-L	856	nad	1186	LCARS	0
1422	lald-L	1070	12ppd-S	1186	LCARS	1
437	h[p]	98	h	1187	LCTSt3ipp	0
437	h[p]	1140	lcts[p]	1187	LCTSt3ipp	0
1421	lcts	98	h	1187	LCTSt3ipp	0
1421	lcts	1140	lcts[p]	1187	LCTSt3ipp	1
747	lcts[e]	1140	lcts[p]	1188	LCTStex	1
437	h[p]	98	h	1189	LCTStpp	0
437	h[p]	1421	lcts	1189	LCTStpp	0
1140	lcts[p]	98	h	1189	LCTStpp	0
1140	lcts[p]	1421	lcts	1189	LCTStpp	1
748	lac-D	98	h	1190	LDH_D	0
748	lac-D	870	nadh	1190	LDH_D	0
748	lac-D	1148	pyr	1190	LDH_D	1
856	nad	98	h	1190	LDH_D	0
856	nad	870	nadh	1190	LDH_D	1
856	nad	1148	pyr	1190	LDH_D	0
748	lac-D	1148	pyr	1191	LDH_D2	1
748	lac-D	1443	q8h2	1191	LDH_D2	0
1003	q8	1148	pyr	1191	LDH_D2	0
1003	q8	1443	q8h2	1191	LDH_D2	1
109	h2o	98	h	1192	LEUabcpp	0
109	h2o	281	adp	1192	LEUabcpp	0
109	h2o	962	pi	1192	LEUabcpp	0
109	h2o	1423	leu-L	1192	LEUabcpp	0
135	atp	98	h	1192	LEUabcpp	0
135	atp	281	adp	1192	LEUabcpp	1

135	atp	962	pi	1192	LEUabcpp	0
135	atp	1423	leu-L	1192	LEUabcpp	0
1141	leu-L[p]	98	h	1192	LEUabcpp	0
1141	leu-L[p]	281	adp	1192	LEUabcpp	0
1141	leu-L[p]	962	pi	1192	LEUabcpp	0
1141	leu-L[p]	1423	leu-L	1192	LEUabcpp	1
437	h[p]	98	h	1193	LEUt2rpp	0
437	h[p]	1423	leu-L	1193	LEUt2rpp	0
1141	leu-L[p]	98	h	1193	LEUt2rpp	0
1141	leu-L[p]	1423	leu-L	1193	LEUt2rpp	1
624	glu-L	213	akg	1194	LEUTAi	1
624	glu-L	1423	leu-L	1194	LEUTAi	0
749	4mop	213	akg	1194	LEUTAi	0
749	4mop	1423	leu-L	1194	LEUTAi	1
750	leu-L[e]	1141	leu-L[p]	1195	LEUtex	1
135	atp	177	amp	1196	LEUTRS	1
135	atp	1192	ppi	1196	LEUTRS	0
135	atp	1603	leutrna	1196	LEUTRS	0
1423	leu-L	177	amp	1196	LEUTRS	0
1423	leu-L	1192	ppi	1196	LEUTRS	0
1423	leu-L	1603	leutrna	1196	LEUTRS	1
1636	trnaleu	177	amp	1196	LEUTRS	0
1636	trnaleu	1192	ppi	1196	LEUTRS	0
1636	trnaleu	1603	leutrna	1196	LEUTRS	1
751	gthrd	1301	lgt-S	1197	LGTHL	1
1321	mthgxl	1301	lgt-S	1197	LGTHL	1
109	h2o	98	h	1198	LIPAabcpp	0
109	h2o	281	adp	1198	LIPAabcpp	0
109	h2o	753	lipa[p]	1198	LIPAabcpp	0
109	h2o	962	pi	1198	LIPAabcpp	0
135	atp	98	h	1198	LIPAabcpp	0
135	atp	281	adp	1198	LIPAabcpp	1
135	atp	753	lipa[p]	1198	LIPAabcpp	0
135	atp	962	pi	1198	LIPAabcpp	0
1408	lipa	98	h	1198	LIPAabcpp	0
1408	lipa	281	adp	1198	LIPAabcpp	0
1408	lipa	753	lipa[p]	1198	LIPAabcpp	1
1408	lipa	962	pi	1198	LIPAabcpp	0
109	h2o	98	h	1199	LIPACabcpp	0
109	h2o	281	adp	1199	LIPACabcpp	0
109	h2o	313	lipa_cold[p]	1199	LIPACabcpp	0
109	h2o	962	pi	1199	LIPACabcpp	0
135	atp	98	h	1199	LIPACabcpp	0
135	atp	281	adp	1199	LIPACabcpp	1
135	atp	313	lipa_cold[p]	1199	LIPACabcpp	0
135	atp	962	pi	1199	LIPACabcpp	0
1595	lipa_cold	98	h	1199	LIPACabcpp	0
1595	lipa_cold	281	adp	1199	LIPACabcpp	0
1595	lipa_cold	313	lipa_cold[p]	1199	LIPACabcpp	1
1595	lipa_cold	962	pi	1199	LIPACabcpp	0
694	hdca[e]	683	h2o[e]	1200	LIPAHT2ex	0
694	hdca[e]	1604	hacolipa[e]	1200	LIPAHT2ex	1
711	h[e]	683	h2o[e]	1200	LIPAHT2ex	0
711	h[e]	1604	hacolipa[e]	1200	LIPAHT2ex	0
752	colipa[e]	683	h2o[e]	1200	LIPAHT2ex	0
752	colipa[e]	1604	hacolipa[e]	1200	LIPAHT2ex	1

694	hdca[e]	683	h2o[e]	1201	LIPAHTex	0
694	hdca[e]	1605	halipa[e]	1201	LIPAHTex	1
711	h[e]	683	h2o[e]	1201	LIPAHTex	0
711	h[e]	1605	halipa[e]	1201	LIPAHTex	0
1302	lipa[e]	683	h2o[e]	1201	LIPAHTex	0
1302	lipa[e]	1605	halipa[e]	1201	LIPAHTex	1
753	lipa[p]	1302	lipa[e]	1202	LIPAtex	1
754	lac-L	1148	pyr	1203	L-LACD2	1
754	lac-L	1443	q8h2	1203	L-LACD2	0
1003	q8	1148	pyr	1203	L-LACD2	0
1003	q8	1443	q8h2	1203	L-LACD2	1
754	lac-L	867	mql8	1204	L-LACD3	0
754	lac-L	1148	pyr	1204	L-LACD3	1
1282	mqn8	867	mql8	1204	L-LACD3	1
1282	mqn8	1148	pyr	1204	L-LACD3	0
437	h[p]	98	h	1205	L-LACt2rpp	0
437	h[p]	754	lac-L	1205	L-LACt2rpp	0
1142	lac-L[p]	98	h	1205	L-LACt2rpp	0
1142	lac-L[p]	754	lac-L	1205	L-LACt2rpp	1
755	lac-L[e]	1142	lac-L[p]	1206	L-LACtex	1
756	lipidX	98	h	1207	LPADSS	0
756	lipidX	1440	udp	1207	LPADSS	0
756	lipidX	1530	lipidAds	1207	LPADSS	1
1424	u23ga	98	h	1207	LPADSS	0
1424	u23ga	1440	udp	1207	LPADSS	1
1424	u23ga	1530	lipidAds	1207	LPADSS	1
684	h2o[p]	437	h[p]	1208	LPLIPAL1A120pp	0
684	h2o[p]	637	glyc3p[p]	1208	LPLIPAL1A120pp	0
684	h2o[p]	1261	ddca[p]	1208	LPLIPAL1A120pp	0
757	1ddecg3p[p]	437	h[p]	1208	LPLIPAL1A120pp	0
757	1ddecg3p[p]	637	glyc3p[p]	1208	LPLIPAL1A120pp	1
757	1ddecg3p[p]	1261	ddca[p]	1208	LPLIPAL1A120pp	1
684	h2o[p]	437	h[p]	1209	LPLIPAL1A140pp	0
684	h2o[p]	637	glyc3p[p]	1209	LPLIPAL1A140pp	0
684	h2o[p]	1356	ttdca[p]	1209	LPLIPAL1A140pp	0
758	1tdecg3p[p]	437	h[p]	1209	LPLIPAL1A140pp	0
758	1tdecg3p[p]	637	glyc3p[p]	1209	LPLIPAL1A140pp	1
758	1tdecg3p[p]	1356	ttdca[p]	1209	LPLIPAL1A140pp	1
684	h2o[p]	437	h[p]	1210	LPLIPAL1A141pp	0
684	h2o[p]	637	glyc3p[p]	1210	LPLIPAL1A141pp	0
684	h2o[p]	1357	ttdcea[p]	1210	LPLIPAL1A141pp	0
759	1tdec7eg3p[p]	437	h[p]	1210	LPLIPAL1A141pp	0
759	1tdec7eg3p[p]	637	glyc3p[p]	1210	LPLIPAL1A141pp	1
759	1tdec7eg3p[p]	1357	ttdcea[p]	1210	LPLIPAL1A141pp	1
684	h2o[p]	437	h[p]	1211	LPLIPAL1A160pp	0
684	h2o[p]	637	glyc3p[p]	1211	LPLIPAL1A160pp	0
684	h2o[p]	1289	hdca[p]	1211	LPLIPAL1A160pp	0
760	1hdecg3p[p]	437	h[p]	1211	LPLIPAL1A160pp	0
760	1hdecg3p[p]	637	glyc3p[p]	1211	LPLIPAL1A160pp	1
760	1hdecg3p[p]	1289	hdca[p]	1211	LPLIPAL1A160pp	1
684	h2o[p]	437	h[p]	1212	LPLIPAL1A161pp	0
684	h2o[p]	637	glyc3p[p]	1212	LPLIPAL1A161pp	0
684	h2o[p]	1290	hdcea[p]	1212	LPLIPAL1A161pp	0
761	1hdec9eg3p[p]	437	h[p]	1212	LPLIPAL1A161pp	0
761	1hdec9eg3p[p]	637	glyc3p[p]	1212	LPLIPAL1A161pp	1
761	1hdec9eg3p[p]	1290	hdcea[p]	1212	LPLIPAL1A161pp	1

684	h2o[p]	437	h[p]	1213	LPLIPAL1A180pp	0
684	h2o[p]	637	glyc3p[p]	1213	LPLIPAL1A180pp	0
684	h2o[p]	1331	ocdca[p]	1213	LPLIPAL1A180pp	0
762	1odecg3p[p]	437	h[p]	1213	LPLIPAL1A180pp	0
762	1odecg3p[p]	637	glyc3p[p]	1213	LPLIPAL1A180pp	1
762	1odecg3p[p]	1331	ocdca[p]	1213	LPLIPAL1A180pp	1
684	h2o[p]	437	h[p]	1214	LPLIPAL1A181pp	0
684	h2o[p]	637	glyc3p[p]	1214	LPLIPAL1A181pp	0
684	h2o[p]	1332	ocdcea[p]	1214	LPLIPAL1A181pp	0
763	1odec11eg3p[p]	437	h[p]	1214	LPLIPAL1A181pp	0
763	1odec11eg3p[p]	637	glyc3p[p]	1214	LPLIPAL1A181pp	1
763	1odec11eg3p[p]	1332	ocdcea[p]	1214	LPLIPAL1A181pp	1
684	h2o[p]	437	h[p]	1215	LPLIPAL1E120pp	0
684	h2o[p]	657	g3pe[p]	1215	LPLIPAL1E120pp	0
684	h2o[p]	1261	ddca[p]	1215	LPLIPAL1E120pp	0
764	1agpe120[p]	437	h[p]	1215	LPLIPAL1E120pp	0
764	1agpe120[p]	657	g3pe[p]	1215	LPLIPAL1E120pp	1
764	1agpe120[p]	1261	ddca[p]	1215	LPLIPAL1E120pp	1
684	h2o[p]	437	h[p]	1216	LPLIPAL1E140pp	0
684	h2o[p]	657	g3pe[p]	1216	LPLIPAL1E140pp	0
684	h2o[p]	1356	ttdca[p]	1216	LPLIPAL1E140pp	0
765	1agpe140[p]	437	h[p]	1216	LPLIPAL1E140pp	0
765	1agpe140[p]	657	g3pe[p]	1216	LPLIPAL1E140pp	1
765	1agpe140[p]	1356	ttdca[p]	1216	LPLIPAL1E140pp	1
684	h2o[p]	437	h[p]	1217	LPLIPAL1E141pp	0
684	h2o[p]	657	g3pe[p]	1217	LPLIPAL1E141pp	0
684	h2o[p]	1357	ttdcea[p]	1217	LPLIPAL1E141pp	0
766	1agpe141[p]	437	h[p]	1217	LPLIPAL1E141pp	0
766	1agpe141[p]	657	g3pe[p]	1217	LPLIPAL1E141pp	1
766	1agpe141[p]	1357	ttdcea[p]	1217	LPLIPAL1E141pp	1
684	h2o[p]	437	h[p]	1218	LPLIPAL1E160pp	0
684	h2o[p]	657	g3pe[p]	1218	LPLIPAL1E160pp	0
684	h2o[p]	1289	hdca[p]	1218	LPLIPAL1E160pp	0
767	1agpe160[p]	437	h[p]	1218	LPLIPAL1E160pp	0
767	1agpe160[p]	657	g3pe[p]	1218	LPLIPAL1E160pp	1
767	1agpe160[p]	1289	hdca[p]	1218	LPLIPAL1E160pp	1
684	h2o[p]	437	h[p]	1219	LPLIPAL1E161pp	0
684	h2o[p]	657	g3pe[p]	1219	LPLIPAL1E161pp	0
684	h2o[p]	1290	hdcea[p]	1219	LPLIPAL1E161pp	0
768	1agpe161[p]	437	h[p]	1219	LPLIPAL1E161pp	0
768	1agpe161[p]	657	g3pe[p]	1219	LPLIPAL1E161pp	1
768	1agpe161[p]	1290	hdcea[p]	1219	LPLIPAL1E161pp	1
684	h2o[p]	437	h[p]	1220	LPLIPAL1E180pp	0
684	h2o[p]	657	g3pe[p]	1220	LPLIPAL1E180pp	0
684	h2o[p]	1331	ocdca[p]	1220	LPLIPAL1E180pp	0
769	1agpe180[p]	437	h[p]	1220	LPLIPAL1E180pp	0
769	1agpe180[p]	657	g3pe[p]	1220	LPLIPAL1E180pp	1
769	1agpe180[p]	1331	ocdca[p]	1220	LPLIPAL1E180pp	1
684	h2o[p]	437	h[p]	1221	LPLIPAL1E181pp	0
684	h2o[p]	657	g3pe[p]	1221	LPLIPAL1E181pp	0
684	h2o[p]	1332	ocdcea[p]	1221	LPLIPAL1E181pp	0
770	1agpe181[p]	437	h[p]	1221	LPLIPAL1E181pp	0
770	1agpe181[p]	657	g3pe[p]	1221	LPLIPAL1E181pp	1
770	1agpe181[p]	1332	ocdcea[p]	1221	LPLIPAL1E181pp	1
684	h2o[p]	437	h[p]	1222	LPLIPAL1G120pp	0
684	h2o[p]	661	g3pg[p]	1222	LPLIPAL1G120pp	0

684	h2o[p]	1261	ddca[p]	1222	LPLIPAL1G120pp	0
771	1agpg120[p]	437	h[p]	1222	LPLIPAL1G120pp	0
771	1agpg120[p]	661	g3pg[p]	1222	LPLIPAL1G120pp	1
771	1agpg120[p]	1261	ddca[p]	1222	LPLIPAL1G120pp	1
684	h2o[p]	437	h[p]	1223	LPLIPAL1G140pp	0
684	h2o[p]	661	g3pg[p]	1223	LPLIPAL1G140pp	0
684	h2o[p]	1356	ttdca[p]	1223	LPLIPAL1G140pp	0
772	1agpg140[p]	437	h[p]	1223	LPLIPAL1G140pp	0
772	1agpg140[p]	661	g3pg[p]	1223	LPLIPAL1G140pp	1
772	1agpg140[p]	1356	ttdca[p]	1223	LPLIPAL1G140pp	1
684	h2o[p]	437	h[p]	1224	LPLIPAL1G141pp	0
684	h2o[p]	661	g3pg[p]	1224	LPLIPAL1G141pp	0
684	h2o[p]	1357	ttdcea[p]	1224	LPLIPAL1G141pp	0
773	1agpg141[p]	437	h[p]	1224	LPLIPAL1G141pp	0
773	1agpg141[p]	661	g3pg[p]	1224	LPLIPAL1G141pp	1
773	1agpg141[p]	1357	ttdcea[p]	1224	LPLIPAL1G141pp	1
684	h2o[p]	437	h[p]	1225	LPLIPAL1G160pp	0
684	h2o[p]	661	g3pg[p]	1225	LPLIPAL1G160pp	0
684	h2o[p]	1289	hdca[p]	1225	LPLIPAL1G160pp	0
774	1agpg160[p]	437	h[p]	1225	LPLIPAL1G160pp	0
774	1agpg160[p]	661	g3pg[p]	1225	LPLIPAL1G160pp	1
774	1agpg160[p]	1289	hdca[p]	1225	LPLIPAL1G160pp	1
684	h2o[p]	437	h[p]	1226	LPLIPAL1G161pp	0
684	h2o[p]	661	g3pg[p]	1226	LPLIPAL1G161pp	0
684	h2o[p]	1290	hdcea[p]	1226	LPLIPAL1G161pp	0
775	1agpg161[p]	437	h[p]	1226	LPLIPAL1G161pp	0
775	1agpg161[p]	661	g3pg[p]	1226	LPLIPAL1G161pp	1
775	1agpg161[p]	1290	hdcea[p]	1226	LPLIPAL1G161pp	1
684	h2o[p]	437	h[p]	1227	LPLIPAL1G180pp	0
684	h2o[p]	661	g3pg[p]	1227	LPLIPAL1G180pp	0
684	h2o[p]	1331	ocdca[p]	1227	LPLIPAL1G180pp	0
776	1agpg180[p]	437	h[p]	1227	LPLIPAL1G180pp	0
776	1agpg180[p]	661	g3pg[p]	1227	LPLIPAL1G180pp	1
776	1agpg180[p]	1331	ocdca[p]	1227	LPLIPAL1G180pp	1
684	h2o[p]	437	h[p]	1228	LPLIPAL1G181pp	0
684	h2o[p]	661	g3pg[p]	1228	LPLIPAL1G181pp	0
684	h2o[p]	1332	ocdcea[p]	1228	LPLIPAL1G181pp	0
777	1agpg181[p]	437	h[p]	1228	LPLIPAL1G181pp	0
777	1agpg181[p]	661	g3pg[p]	1228	LPLIPAL1G181pp	1
777	1agpg181[p]	1332	ocdcea[p]	1228	LPLIPAL1G181pp	1
109	h2o	98	h	1229	LPLIPAL2A120	0
109	h2o	558	glyc3p	1229	LPLIPAL2A120	0
109	h2o	1303	ddca	1229	LPLIPAL2A120	0
778	2ddecg3p	98	h	1229	LPLIPAL2A120	0
778	2ddecg3p	558	glyc3p	1229	LPLIPAL2A120	1
778	2ddecg3p	1303	ddca	1229	LPLIPAL2A120	1
109	h2o	98	h	1230	LPLIPAL2A140	0
109	h2o	558	glyc3p	1230	LPLIPAL2A140	0
109	h2o	1616	ttdca	1230	LPLIPAL2A140	0
779	2tdecg3p	98	h	1230	LPLIPAL2A140	0
779	2tdecg3p	558	glyc3p	1230	LPLIPAL2A140	1
779	2tdecg3p	1616	ttdca	1230	LPLIPAL2A140	1
109	h2o	98	h	1231	LPLIPAL2A141	0
109	h2o	558	glyc3p	1231	LPLIPAL2A141	0
109	h2o	1617	ttdcea	1231	LPLIPAL2A141	0
780	2tdec7eg3p	98	h	1231	LPLIPAL2A141	0

780	2tdec7eg3p	558	glyc3p	1231	LPLIPAL2A141	1
780	2tdec7eg3p	1617	ttdcea	1231	LPLIPAL2A141	1
109	h2o	98	h	1232	LPLIPAL2A160	0
109	h2o	558	glyc3p	1232	LPLIPAL2A160	0
109	h2o	1618	hdca	1232	LPLIPAL2A160	0
781	2hdecg3p	98	h	1232	LPLIPAL2A160	0
781	2hdecg3p	558	glyc3p	1232	LPLIPAL2A160	1
781	2hdecg3p	1618	hdca	1232	LPLIPAL2A160	1
109	h2o	98	h	1233	LPLIPAL2A161	0
109	h2o	558	glyc3p	1233	LPLIPAL2A161	0
109	h2o	1619	hdcea	1233	LPLIPAL2A161	0
782	2hdec9eg3p	98	h	1233	LPLIPAL2A161	0
782	2hdec9eg3p	558	glyc3p	1233	LPLIPAL2A161	1
782	2hdec9eg3p	1619	hdcea	1233	LPLIPAL2A161	1
109	h2o	98	h	1234	LPLIPAL2A180	0
109	h2o	558	glyc3p	1234	LPLIPAL2A180	0
109	h2o	1620	ocdca	1234	LPLIPAL2A180	0
783	2odecg3p	98	h	1234	LPLIPAL2A180	0
783	2odecg3p	558	glyc3p	1234	LPLIPAL2A180	1
783	2odecg3p	1620	ocdca	1234	LPLIPAL2A180	1
109	h2o	98	h	1235	LPLIPAL2A181	0
109	h2o	558	glyc3p	1235	LPLIPAL2A181	0
109	h2o	1621	ocdcea	1235	LPLIPAL2A181	0
784	2odec11eg3p	98	h	1235	LPLIPAL2A181	0
784	2odec11eg3p	558	glyc3p	1235	LPLIPAL2A181	1
784	2odec11eg3p	1621	ocdcea	1235	LPLIPAL2A181	1
39	2agpe120	656	g3pe	1236	LPLIPAL2ATE120	1
39	2agpe120	1304	apg120	1236	LPLIPAL2ATE120	1
1336	pg120	656	g3pe	1236	LPLIPAL2ATE120	0
1336	pg120	1304	apg120	1236	LPLIPAL2ATE120	1
40	2agpe140	656	g3pe	1237	LPLIPAL2ATE140	1
40	2agpe140	1305	apg140	1237	LPLIPAL2ATE140	1
1337	pg140	656	g3pe	1237	LPLIPAL2ATE140	0
1337	pg140	1305	apg140	1237	LPLIPAL2ATE140	1
41	2agpe141	656	g3pe	1238	LPLIPAL2ATE141	1
41	2agpe141	1306	apg141	1238	LPLIPAL2ATE141	1
1338	pg141	656	g3pe	1238	LPLIPAL2ATE141	0
1338	pg141	1306	apg141	1238	LPLIPAL2ATE141	1
42	2agpe160	656	g3pe	1239	LPLIPAL2ATE160	1
42	2agpe160	1307	apg160	1239	LPLIPAL2ATE160	1
1339	pg160	656	g3pe	1239	LPLIPAL2ATE160	0
1339	pg160	1307	apg160	1239	LPLIPAL2ATE160	1
43	2agpe161	656	g3pe	1240	LPLIPAL2ATE161	1
43	2agpe161	1308	apg161	1240	LPLIPAL2ATE161	1
1340	pg161	656	g3pe	1240	LPLIPAL2ATE161	0
1340	pg161	1308	apg161	1240	LPLIPAL2ATE161	1
44	2agpe180	656	g3pe	1241	LPLIPAL2ATE180	1
44	2agpe180	1309	apg180	1241	LPLIPAL2ATE180	1
1341	pg180	656	g3pe	1241	LPLIPAL2ATE180	0
1341	pg180	1309	apg180	1241	LPLIPAL2ATE180	1
45	2agpe181	656	g3pe	1242	LPLIPAL2ATE181	1
45	2agpe181	1310	apg181	1242	LPLIPAL2ATE181	1
1342	pg181	656	g3pe	1242	LPLIPAL2ATE181	0
1342	pg181	1310	apg181	1242	LPLIPAL2ATE181	1
53	2agpg120	660	g3pg	1243	LPLIPAL2ATG120	1
53	2agpg120	1304	apg120	1243	LPLIPAL2ATG120	1

1336	pg120	660	g3pg	1243	LPLIPAL2ATG120	0
1336	pg120	1304	apg120	1243	LPLIPAL2ATG120	1
54	2agpg140	660	g3pg	1244	LPLIPAL2ATG140	1
54	2agpg140	1305	apg140	1244	LPLIPAL2ATG140	1
1337	pg140	660	g3pg	1244	LPLIPAL2ATG140	0
1337	pg140	1305	apg140	1244	LPLIPAL2ATG140	1
55	2agpg141	660	g3pg	1245	LPLIPAL2ATG141	1
55	2agpg141	1306	apg141	1245	LPLIPAL2ATG141	1
1338	pg141	660	g3pg	1245	LPLIPAL2ATG141	0
1338	pg141	1306	apg141	1245	LPLIPAL2ATG141	1
56	2agpg160	660	g3pg	1246	LPLIPAL2ATG160	1
56	2agpg160	1307	apg160	1246	LPLIPAL2ATG160	1
1339	pg160	660	g3pg	1246	LPLIPAL2ATG160	0
1339	pg160	1307	apg160	1246	LPLIPAL2ATG160	1
57	2agpg161	660	g3pg	1247	LPLIPAL2ATG161	1
57	2agpg161	1308	apg161	1247	LPLIPAL2ATG161	1
1340	pg161	660	g3pg	1247	LPLIPAL2ATG161	0
1340	pg161	1308	apg161	1247	LPLIPAL2ATG161	1
58	2agpg180	660	g3pg	1248	LPLIPAL2ATG180	1
58	2agpg180	1309	apg180	1248	LPLIPAL2ATG180	1
1341	pg180	660	g3pg	1248	LPLIPAL2ATG180	0
1341	pg180	1309	apg180	1248	LPLIPAL2ATG180	1
59	2agpg181	660	g3pg	1249	LPLIPAL2ATG181	1
59	2agpg181	1310	apg181	1249	LPLIPAL2ATG181	1
1342	pg181	660	g3pg	1249	LPLIPAL2ATG181	0
1342	pg181	1310	apg181	1249	LPLIPAL2ATG181	1
39	2agpe120	98	h	1250	LPLIPAL2E120	0
39	2agpe120	656	g3pe	1250	LPLIPAL2E120	1
39	2agpe120	1303	ddca	1250	LPLIPAL2E120	1
109	h2o	98	h	1250	LPLIPAL2E120	0
109	h2o	656	g3pe	1250	LPLIPAL2E120	0
109	h2o	1303	ddca	1250	LPLIPAL2E120	0
40	2agpe140	98	h	1251	LPLIPAL2E140	0
40	2agpe140	656	g3pe	1251	LPLIPAL2E140	1
40	2agpe140	1616	ttdca	1251	LPLIPAL2E140	1
109	h2o	98	h	1251	LPLIPAL2E140	0
109	h2o	656	g3pe	1251	LPLIPAL2E140	0
109	h2o	1616	ttdca	1251	LPLIPAL2E140	0
41	2agpe141	98	h	1252	LPLIPAL2E141	0
41	2agpe141	656	g3pe	1252	LPLIPAL2E141	1
41	2agpe141	1617	ttdcea	1252	LPLIPAL2E141	1
109	h2o	98	h	1252	LPLIPAL2E141	0
109	h2o	656	g3pe	1252	LPLIPAL2E141	0
109	h2o	1617	ttdcea	1252	LPLIPAL2E141	0
42	2agpe160	98	h	1253	LPLIPAL2E160	0
42	2agpe160	656	g3pe	1253	LPLIPAL2E160	1
42	2agpe160	1618	hdca	1253	LPLIPAL2E160	1
109	h2o	98	h	1253	LPLIPAL2E160	0
109	h2o	656	g3pe	1253	LPLIPAL2E160	0
109	h2o	1618	hdca	1253	LPLIPAL2E160	0
43	2agpe161	98	h	1254	LPLIPAL2E161	0
43	2agpe161	656	g3pe	1254	LPLIPAL2E161	1
43	2agpe161	1619	hdcea	1254	LPLIPAL2E161	1
109	h2o	98	h	1254	LPLIPAL2E161	0
109	h2o	656	g3pe	1254	LPLIPAL2E161	0
109	h2o	1619	hdcea	1254	LPLIPAL2E161	0

44	2agpe180	98	h	1255	LPLIPAL2E180	0
44	2agpe180	656	g3pe	1255	LPLIPAL2E180	1
44	2agpe180	1620	ocdca	1255	LPLIPAL2E180	1
109	h2o	98	h	1255	LPLIPAL2E180	0
109	h2o	656	g3pe	1255	LPLIPAL2E180	0
109	h2o	1620	ocdca	1255	LPLIPAL2E180	0
45	2agpe181	98	h	1256	LPLIPAL2E181	0
45	2agpe181	656	g3pe	1256	LPLIPAL2E181	1
45	2agpe181	1621	ocdcea	1256	LPLIPAL2E181	1
109	h2o	98	h	1256	LPLIPAL2E181	0
109	h2o	656	g3pe	1256	LPLIPAL2E181	0
109	h2o	1621	ocdcea	1256	LPLIPAL2E181	0
53	2agpg120	98	h	1257	LPLIPAL2G120	0
53	2agpg120	660	g3pg	1257	LPLIPAL2G120	1
53	2agpg120	1303	ddca	1257	LPLIPAL2G120	1
109	h2o	98	h	1257	LPLIPAL2G120	0
109	h2o	660	g3pg	1257	LPLIPAL2G120	0
109	h2o	1303	ddca	1257	LPLIPAL2G120	0
54	2agpg140	98	h	1258	LPLIPAL2G140	0
54	2agpg140	660	g3pg	1258	LPLIPAL2G140	1
54	2agpg140	1616	ttdca	1258	LPLIPAL2G140	1
109	h2o	98	h	1258	LPLIPAL2G140	0
109	h2o	660	g3pg	1258	LPLIPAL2G140	0
109	h2o	1616	ttdca	1258	LPLIPAL2G140	0
55	2agpg141	98	h	1259	LPLIPAL2G141	0
55	2agpg141	660	g3pg	1259	LPLIPAL2G141	1
55	2agpg141	1617	ttdcea	1259	LPLIPAL2G141	1
109	h2o	98	h	1259	LPLIPAL2G141	0
109	h2o	660	g3pg	1259	LPLIPAL2G141	0
109	h2o	1617	ttdcea	1259	LPLIPAL2G141	0
56	2agpg160	98	h	1260	LPLIPAL2G160	0
56	2agpg160	660	g3pg	1260	LPLIPAL2G160	1
56	2agpg160	1618	hdca	1260	LPLIPAL2G160	1
109	h2o	98	h	1260	LPLIPAL2G160	0
109	h2o	660	g3pg	1260	LPLIPAL2G160	0
109	h2o	1618	hdca	1260	LPLIPAL2G160	0
57	2agpg161	98	h	1261	LPLIPAL2G161	0
57	2agpg161	660	g3pg	1261	LPLIPAL2G161	1
57	2agpg161	1619	hdcea	1261	LPLIPAL2G161	1
109	h2o	98	h	1261	LPLIPAL2G161	0
109	h2o	660	g3pg	1261	LPLIPAL2G161	0
109	h2o	1619	hdcea	1261	LPLIPAL2G161	0
58	2agpg180	98	h	1262	LPLIPAL2G180	0
58	2agpg180	660	g3pg	1262	LPLIPAL2G180	1
58	2agpg180	1620	ocdca	1262	LPLIPAL2G180	1
109	h2o	98	h	1262	LPLIPAL2G180	0
109	h2o	660	g3pg	1262	LPLIPAL2G180	0
109	h2o	1620	ocdca	1262	LPLIPAL2G180	0
59	2agpg181	98	h	1263	LPLIPAL2G181	0
59	2agpg181	660	g3pg	1263	LPLIPAL2G181	1
59	2agpg181	1621	ocdcea	1263	LPLIPAL2G181	1
109	h2o	98	h	1263	LPLIPAL2G181	0
109	h2o	660	g3pg	1263	LPLIPAL2G181	0
109	h2o	1621	ocdcea	1263	LPLIPAL2G181	0
459	nadp	98	h	1264	LSERDHr	0
459	nadp	871	nadph	1264	LSERDHr	1

459	nadp	1106	2amsa	1264	LSERDHr	0
604	ser-L	98	h	1264	LSERDHr	0
604	ser-L	871	nadph	1264	LSERDHr	0
604	ser-L	1106	2amsa	1264	LSERDHr	1
109	h2o	98	h	1265	LYSabcpp	0
109	h2o	281	adp	1265	LYSabcpp	0
109	h2o	962	pi	1265	LYSabcpp	0
109	h2o	1425	lys-L	1265	LYSabcpp	0
135	atp	98	h	1265	LYSabcpp	0
135	atp	281	adp	1265	LYSabcpp	1
135	atp	962	pi	1265	LYSabcpp	0
135	atp	1425	lys-L	1265	LYSabcpp	0
1143	lys-L[p]	98	h	1265	LYSabcpp	0
1143	lys-L[p]	281	adp	1265	LYSabcpp	0
1143	lys-L[p]	962	pi	1265	LYSabcpp	0
1143	lys-L[p]	1425	lys-L	1265	LYSabcpp	1
98	h	291	15dap	1266	LYSDC	0
98	h	692	co2	1266	LYSDC	0
1425	lys-L	291	15dap	1266	LYSDC	1
1425	lys-L	692	co2	1266	LYSDC	0
437	h[p]	98	h	1267	LYSt2pp	0
437	h[p]	1425	lys-L	1267	LYSt2pp	0
1143	lys-L[p]	98	h	1267	LYSt2pp	0
1143	lys-L[p]	1425	lys-L	1267	LYSt2pp	1
437	h[p]	98	h	1268	LYSt3pp	0
437	h[p]	1143	lys-L[p]	1268	LYSt3pp	0
1425	lys-L	98	h	1268	LYSt3pp	0
1425	lys-L	1143	lys-L[p]	1268	LYSt3pp	1
785	lys-L[e]	1143	lys-L[p]	1269	LYStex	1
135	atp	177	amp	1270	LYSTRS	1
135	atp	1192	ppi	1270	LYSTRS	0
135	atp	1606	lystrna	1270	LYSTRS	0
1425	lys-L	177	amp	1270	LYSTRS	0
1425	lys-L	1192	ppi	1270	LYSTRS	0
1425	lys-L	1606	lystrna	1270	LYSTRS	1
1637	trnalys	177	amp	1270	LYSTRS	0
1637	trnalys	1192	ppi	1270	LYSTRS	0
1637	trnalys	1606	lystrna	1270	LYSTRS	1
786	lyx-L	1311	xylu-L	1271	LYXI	1
437	h[p]	98	h	1272	LYXt2pp	0
437	h[p]	786	lyx-L	1272	LYXt2pp	0
1144	lyx-L[p]	98	h	1272	LYXt2pp	0
1144	lyx-L[p]	786	lyx-L	1272	LYXt2pp	1
787	lyx-L[e]	1144	lyx-L[p]	1273	LYXtex	1
788	mnl1p	98	h	1274	M1PD	0
788	mnl1p	499	f6p	1274	M1PD	1
788	mnl1p	870	nadh	1274	M1PD	0
856	nad	98	h	1274	M1PD	0
856	nad	499	f6p	1274	M1PD	0
856	nad	870	nadh	1274	M1PD	1
98	h	692	co2	1275	MACPD	0
98	h	733	acACP	1275	MACPD	0
1363	malACP	692	co2	1275	MACPD	0
1363	malACP	733	acACP	1275	MACPD	1
789	mal-D	692	co2	1276	MALDDH	0
789	mal-D	870	nadh	1276	MALDDH	0

789	mal-D	1148	pyr	1276	MALDDH	1
856	nad	692	co2	1276	MALDDH	0
856	nad	870	nadh	1276	MALDDH	1
856	nad	1148	pyr	1276	MALDDH	0
437	h[p]	98	h	1277	MALDt2_2pp	0
437	h[p]	789	mal-D	1277	MALDt2_2pp	0
1145	mal-D[p]	98	h	1277	MALDt2_2pp	0
1145	mal-D[p]	789	mal-D	1277	MALDt2_2pp	1
790	mal-D[e]	1145	mal-D[p]	1278	MALDtex	1
109	h2o	98	h	1279	MALS	0
109	h2o	811	mal-L	1279	MALS	0
109	h2o	927	coa	1279	MALS	0
128	accoa	98	h	1279	MALS	0
128	accoa	811	mal-L	1279	MALS	1
128	accoa	927	coa	1279	MALS	1
631	glx	98	h	1279	MALS	0
631	glx	811	mal-L	1279	MALS	1
631	glx	927	coa	1279	MALS	0
437	h[p]	98	h	1280	MALt2_2pp	0
437	h[p]	811	mal-L	1280	MALt2_2pp	0
1147	mal-L[p]	98	h	1280	MALt2_2pp	0
1147	mal-L[p]	811	mal-L	1280	MALt2_2pp	1
437	h[p]	98	h	1281	MALt2_3pp	0
437	h[p]	811	mal-L	1281	MALt2_3pp	0
1147	mal-L[p]	98	h	1281	MALt2_3pp	0
1147	mal-L[p]	811	mal-L	1281	MALt2_3pp	1
109	h2o	98	h	1282	MALTabcpp	0
109	h2o	241	malt	1282	MALTabcpp	0
109	h2o	281	adp	1282	MALTabcpp	0
109	h2o	962	pi	1282	MALTabcpp	0
135	atp	98	h	1282	MALTabcpp	0
135	atp	241	malt	1282	MALTabcpp	0
135	atp	281	adp	1282	MALTabcpp	1
135	atp	962	pi	1282	MALTabcpp	0
793	malt[p]	98	h	1282	MALTabcpp	0
793	malt[p]	241	malt	1282	MALTabcpp	1
793	malt[p]	281	adp	1282	MALTabcpp	0
793	malt[p]	962	pi	1282	MALTabcpp	0
128	accoa	927	coa	1283	MALTATr	1
128	accoa	1146	acmalt	1283	MALTATr	1
241	malt	927	coa	1283	MALTATr	0
241	malt	1146	acmalt	1283	MALTATr	1
791	mal-L[e]	1147	mal-L[p]	1284	MALtex	1
109	h2o	98	h	1285	MALTHXabcpp	0
109	h2o	281	adp	1285	MALTHXabcpp	0
109	h2o	834	malthx	1285	MALTHXabcpp	0
109	h2o	962	pi	1285	MALTHXabcpp	0
135	atp	98	h	1285	MALTHXabcpp	0
135	atp	281	adp	1285	MALTHXabcpp	1
135	atp	834	malthx	1285	MALTHXabcpp	0
135	atp	962	pi	1285	MALTHXabcpp	0
1240	malthx[p]	98	h	1285	MALTHXabcpp	0
1240	malthx[p]	281	adp	1285	MALTHXabcpp	0
1240	malthx[p]	834	malthx	1285	MALTHXabcpp	1
1240	malthx[p]	962	pi	1285	MALTHXabcpp	0
792	malthx[e]	1240	malthx[p]	1286	MALTHXtexi	1

109	h2o	98	h	1287	MALTPTabcpp	0
109	h2o	281	adp	1287	MALTPTabcpp	0
109	h2o	833	maltpt	1287	MALTPTabcpp	0
109	h2o	962	pi	1287	MALTPTabcpp	0
135	atp	98	h	1287	MALTPTabcpp	0
135	atp	281	adp	1287	MALTPTabcpp	1
135	atp	833	maltpt	1287	MALTPTabcpp	0
135	atp	962	pi	1287	MALTPTabcpp	0
1313	maltpt[p]	98	h	1287	MALTPTabcpp	0
1313	maltpt[p]	281	adp	1287	MALTPTabcpp	0
1313	maltpt[p]	833	maltpt	1287	MALTPTabcpp	1
1313	maltpt[p]	962	pi	1287	MALTPTabcpp	0
793	malt[p]	1148	pyr	1288	MALTptspp	0
793	malt[p]	1312	malt6p	1288	MALTptspp	1
959	pep	1148	pyr	1288	MALTptspp	1
959	pep	1312	malt6p	1288	MALTptspp	0
794	maltpt[e]	1313	maltpt[p]	1289	MALTPTtexi	1
795	malt[e]	793	malt[p]	1290	MALTtexi	1
109	h2o	98	h	1291	MALTTRabcpp	0
109	h2o	281	adp	1291	MALTTRabcpp	0
109	h2o	962	pi	1291	MALTTRabcpp	0
109	h2o	1379	malttr	1291	MALTTRabcpp	0
135	atp	98	h	1291	MALTTRabcpp	0
135	atp	281	adp	1291	MALTTRabcpp	1
135	atp	962	pi	1291	MALTTRabcpp	0
135	atp	1379	malttr	1291	MALTTRabcpp	0
1314	malttr[p]	98	h	1291	MALTTRabcpp	0
1314	malttr[p]	281	adp	1291	MALTTRabcpp	0
1314	malttr[p]	962	pi	1291	MALTTRabcpp	0
1314	malttr[p]	1379	malttr	1291	MALTTRabcpp	1
796	malttr[e]	1314	malttr[p]	1292	MALTTRtexi	1
109	h2o	98	h	1293	MALTTTRabcpp	0
109	h2o	281	adp	1293	MALTTTRabcpp	0
109	h2o	962	pi	1293	MALTTTRabcpp	0
109	h2o	1380	maltttr	1293	MALTTTRabcpp	0
135	atp	98	h	1293	MALTTTRabcpp	0
135	atp	281	adp	1293	MALTTTRabcpp	1
135	atp	962	pi	1293	MALTTTRabcpp	0
135	atp	1380	maltttr	1293	MALTTTRabcpp	0
1315	maltttr[p]	98	h	1293	MALTTTRabcpp	0
1315	maltttr[p]	281	adp	1293	MALTTTRabcpp	0
1315	maltttr[p]	962	pi	1293	MALTTTRabcpp	0
1315	maltttr[p]	1380	maltttr	1293	MALTTTRabcpp	1
797	maltttr[e]	1315	maltttr[p]	1294	MALTTTRtexi	1
98	h	599	gdpmann	1295	MAN1PT2	0
98	h	962	pi	1295	MAN1PT2	0
798	gdp	599	gdpmann	1295	MAN1PT2	1
798	gdp	962	pi	1295	MAN1PT2	0
938	man1p	599	gdpmann	1295	MAN1PT2	1
938	man1p	962	pi	1295	MAN1PT2	0
799	man6p	499	f6p	1296	MAN6PI	1
800	man6p[p]	799	man6p	1297	MAN6Pt6_2pp	1
800	man6p[p]	1173	pi[p]	1297	MAN6Pt6_2pp	0
962	pi	799	man6p	1297	MAN6Pt6_2pp	0
962	pi	1173	pi[p]	1297	MAN6Pt6_2pp	0
801	man6p[e]	800	man6p[p]	1298	MAN6Ptex	1

802	mana	98	h	1299	MANAO	0
802	mana	870	nadh	1299	MANAO	0
802	mana	1112	fruur	1299	MANAO	1
856	nad	98	h	1299	MANAO	0
856	nad	870	nadh	1299	MANAO	1
856	nad	1112	fruur	1299	MANAO	0
803	manglyc[p]	1148	pyr	1300	MANGLYCptspp	0
803	manglyc[p]	1316	man6pglyc	1300	MANGLYCptspp	1
959	pep	1148	pyr	1300	MANGLYCptspp	1
959	pep	1316	man6pglyc	1300	MANGLYCptspp	0
804	manglyc[e]	803	manglyc[p]	1301	MANGLYCtex	1
109	h2o	799	man6p	1302	MANPGH	0
109	h2o	1118	glyc-R	1302	MANPGH	0
1316	man6pglyc	799	man6p	1302	MANPGH	1
1316	man6pglyc	1118	glyc-R	1302	MANPGH	1
805	man[p]	799	man6p	1303	MANptspp	1
805	man[p]	1148	pyr	1303	MANptspp	0
959	pep	799	man6p	1303	MANptspp	0
959	pep	1148	pyr	1303	MANptspp	1
806	man[e]	805	man[p]	1304	MANtex	1
807	2mcit	109	h2o	1305	MCITD	0
807	2mcit	827	2mcacn	1305	MCITD	1
808	micit	1008	succ	1306	MCITL2	1
808	micit	1148	pyr	1306	MCITL2	1
109	h2o	98	h	1307	MCITS	0
109	h2o	807	2mcit	1307	MCITS	0
109	h2o	927	coa	1307	MCITS	0
948	ppcoa	98	h	1307	MCITS	0
948	ppcoa	807	2mcit	1307	MCITS	1
948	ppcoa	927	coa	1307	MCITS	1
1318	oaa	98	h	1307	MCITS	0
1318	oaa	807	2mcit	1307	MCITS	1
1318	oaa	927	coa	1307	MCITS	0
118	ACP	927	coa	1308	MCOATA	0
118	ACP	1363	malACP	1308	MCOATA	1
1426	malcoa	927	coa	1308	MCOATA	1
1426	malcoa	1363	malACP	1308	MCOATA	1
359	cyan	98	h	1309	MCPST	0
359	cyan	1148	pyr	1309	MCPST	0
359	cyan	1655	tcynt	1309	MCPST	1
1427	mercppyr	98	h	1309	MCPST	0
1427	mercppyr	1148	pyr	1309	MCPST	1
1427	mercppyr	1655	tcynt	1309	MCPST	1
809	murein5p5p[p]	384	ala-D[p]	1310	MCTP1App	1
809	murein5p5p[p]	1428	murein5px4p[p]	1310	MCTP1App	1
809	murein5p5p[p]	217	alaala[p]	1311	MCTP1Bpp	1
809	murein5p5p[p]	1435	murein5px3p[p]	1311	MCTP1Bpp	1
810	nurein5p5p5p[p	384	ala-D[p]	1312	MCTP2App	1
810	nurein5p5p5p[p	1429	murein5px4px4p[p]	1312	MCTP2App	1
684	h2o[p]	384	ala-D[p]	1313	MDDCP1pp	0
684	h2o[p]	1430	murein4px4p[p]	1313	MDDCP1pp	0
1428	murein5px4p[p]	384	ala-D[p]	1313	MDDCP1pp	1
1428	murein5px4p[p]	1430	murein4px4p[p]	1313	MDDCP1pp	1
684	h2o[p]	384	ala-D[p]	1314	MDDCP2pp	0
684	h2o[p]	1432	murein4px4px4p[p]	1314	MDDCP2pp	0
1429	urein5px4px4p[	384	ala-D[p]	1314	MDDCP2pp	1

1429	urein5px4px4p[	1432	murein4px4px4p[p]	1314	MDDCP2pp	1
684	h2o[p]	384	ala-D[p]	1315	MDDCP3pp	0
684	h2o[p]	1317	murein5p4p[p]	1315	MDDCP3pp	0
809	murein5p5p[p]	384	ala-D[p]	1315	MDDCP3pp	1
809	murein5p5p[p]	1317	murein5p4p[p]	1315	MDDCP3pp	1
684	h2o[p]	384	ala-D[p]	1316	MDDCP4pp	0
684	h2o[p]	829	murein4p4p[p]	1316	MDDCP4pp	0
1317	murein5p4p[p]	384	ala-D[p]	1316	MDDCP4pp	1
1317	murein5p4p[p]	829	murein4p4p[p]	1316	MDDCP4pp	1
684	h2o[p]	384	ala-D[p]	1317	MDDCP5pp	0
684	h2o[p]	830	murein4p3p[p]	1317	MDDCP5pp	0
1323	murein5p3p[p]	384	ala-D[p]	1317	MDDCP5pp	1
1323	murein5p3p[p]	830	murein4p3p[p]	1317	MDDCP5pp	1
684	h2o[p]	829	murein4p4p[p]	1318	MDDEP1pp	0
1430	murein4px4p[p	829	murein4p4p[p]	1318	MDDEP1pp	1
684	h2o[p]	830	murein4p3p[p]	1319	MDDEP2pp	0
1431	murein3px4p[p	830	murein4p3p[p]	1319	MDDEP2pp	1
684	h2o[p]	1317	murein5p4p[p]	1320	MDDEP3pp	0
1428	murein5px4p[p	1317	murein5p4p[p]	1320	MDDEP3pp	1
684	h2o[p]	832	murein4px4p4p[p]	1321	MDDEP4pp	0
1432	urein4px4px4p[	832	murein4px4p4p[p]	1321	MDDEP4pp	1
811	mal-L	98	h	1322	MDH	0
811	mal-L	870	nadh	1322	MDH	0
811	mal-L	1318	oaa	1322	MDH	1
856	nad	98	h	1322	MDH	0
856	nad	870	nadh	1322	MDH	1
856	nad	1318	oaa	1322	MDH	0
811	mal-L	1318	oaa	1323	MDH2	1
811	mal-L	1443	q8h2	1323	MDH2	0
1003	q8	1318	oaa	1323	MDH2	0
1003	q8	1443	q8h2	1323	MDH2	1
811	mal-L	867	mql8	1324	MDH3	0
811	mal-L	1318	oaa	1324	MDH3	1
1282	mqn8	867	mql8	1324	MDH3	1
1282	mqn8	1318	oaa	1324	MDH3	0
812	5mdru1p	109	h2o	1325	MDRPD	0
812	5mdru1p	436	dkmpp	1325	MDRPD	1
811	mal-L	692	co2	1326	ME1	0
811	mal-L	870	nadh	1326	ME1	0
811	mal-L	1148	pyr	1326	ME1	1
856	nad	692	co2	1326	ME1	0
856	nad	870	nadh	1326	ME1	1
856	nad	1148	pyr	1326	ME1	0
459	nadp	692	co2	1327	ME2	0
459	nadp	871	nadph	1327	ME2	1
459	nadp	1148	pyr	1327	ME2	0
811	mal-L	692	co2	1327	ME2	0
811	mal-L	871	nadph	1327	ME2	0
811	mal-L	1148	pyr	1327	ME2	1
813	2mecdp	109	h2o	1328	MECDPDH2	0
813	2mecdp	856	nad	1328	MECDPDH2	0
813	2mecdp	1319	h2mb4p	1328	MECDPDH2	1
870	nadh	109	h2o	1328	MECDPDH2	0
870	nadh	856	nad	1328	MECDPDH2	1
870	nadh	1319	h2mb4p	1328	MECDPDH2	0
814	2p4c2me	330	cmp	1329	MECDPS	1

814	2p4c2me	813	2mecdp	1329	MECDPS	1
437	h[p]	98	h	1330	MELIBt2pp	0
437	h[p]	1401	melib	1330	MELIBt2pp	0
1149	melib[p]	98	h	1330	MELIBt2pp	0
1149	melib[p]	1401	melib	1330	MELIBt2pp	1
437	h[p]	98	h	1331	MELIBt3ipp	0
437	h[p]	1149	melib[p]	1331	MELIBt3ipp	0
1401	melib	98	h	1331	MELIBt3ipp	0
1401	melib	1149	melib[p]	1331	MELIBt3ipp	1
815	melib[e]	1149	melib[p]	1332	MELIBtex	1
98	h	305	4c2me	1333	MEPCT	0
98	h	1192	ppi	1333	MEPCT	0
392	ctp	305	4c2me	1333	MEPCT	1
392	ctp	1192	ppi	1333	MEPCT	0
816	2me4p	305	4c2me	1333	MEPCT	1
816	2me4p	1192	ppi	1333	MEPCT	0
109	h2o	98	h	1334	METabcpp	0
109	h2o	281	adp	1334	METabcpp	0
109	h2o	962	pi	1334	METabcpp	0
109	h2o	1433	met-L	1334	METabcpp	0
135	atp	98	h	1334	METabcpp	0
135	atp	281	adp	1334	METabcpp	1
135	atp	962	pi	1334	METabcpp	0
135	atp	1433	met-L	1334	METabcpp	0
1153	met-L[p]	98	h	1334	METabcpp	0
1153	met-L[p]	281	adp	1334	METabcpp	0
1153	met-L[p]	962	pi	1334	METabcpp	0
1153	met-L[p]	1433	met-L	1334	METabcpp	1
109	h2o	178	amet	1335	METAT	0
109	h2o	962	pi	1335	METAT	0
109	h2o	1192	ppi	1335	METAT	0
135	atp	178	amet	1335	METAT	1
135	atp	962	pi	1335	METAT	0
135	atp	1192	ppi	1335	METAT	0
1433	met-L	178	amet	1335	METAT	1
1433	met-L	962	pi	1335	METAT	0
1433	met-L	1192	ppi	1335	METAT	0
109	h2o	98	h	1336	METDabcpp	0
109	h2o	281	adp	1336	METDabcpp	0
109	h2o	962	pi	1336	METDabcpp	0
109	h2o	1656	met-D	1336	METDabcpp	0
135	atp	98	h	1336	METDabcpp	0
135	atp	281	adp	1336	METDabcpp	1
135	atp	962	pi	1336	METDabcpp	0
135	atp	1656	met-D	1336	METDabcpp	0
1150	met-D[p]	98	h	1336	METDabcpp	0
1150	met-D[p]	281	adp	1336	METDabcpp	0
1150	met-D[p]	962	pi	1336	METDabcpp	0
1150	met-D[p]	1656	met-D	1336	METDabcpp	1
817	met-D[e]	1150	met-D[p]	1337	METDtex	1
292	h2o2	109	h2o	1338	METOX1s	0
292	h2o2	821	metsox-S-L	1338	METOX1s	0
1433	met-L	109	h2o	1338	METOX1s	0
1433	met-L	821	metsox-S-L	1338	METOX1s	1
292	h2o2	109	h2o	1339	METOX2s	0
292	h2o2	822	metsox-R-L	1339	METOX2s	0

1433	met-L	109	h2o	1339	METOX2s	0
1433	met-L	822	metsox-R-L	1339	METOX2s	1
693	hcys-L	98	h	1340	METS	0
693	hcys-L	1403	thf	1340	METS	0
693	hcys-L	1433	met-L	1340	METS	1
818	5mthf	98	h	1340	METS	0
818	5mthf	1403	thf	1340	METS	1
818	5mthf	1433	met-L	1340	METS	1
109	h2o	98	h	1341	METSOX1abcpp	0
109	h2o	281	adp	1341	METSOX1abcpp	0
109	h2o	821	metsox-S-L	1341	METSOX1abcpp	0
109	h2o	962	pi	1341	METSOX1abcpp	0
135	atp	98	h	1341	METSOX1abcpp	0
135	atp	281	adp	1341	METSOX1abcpp	1
135	atp	821	metsox-S-L	1341	METSOX1abcpp	0
135	atp	962	pi	1341	METSOX1abcpp	0
1151	metsox-S-L[p]	98	h	1341	METSOX1abcpp	0
1151	metsox-S-L[p]	281	adp	1341	METSOX1abcpp	0
1151	metsox-S-L[p]	821	metsox-S-L	1341	METSOX1abcpp	1
1151	metsox-S-L[p]	962	pi	1341	METSOX1abcpp	0
819	metsox-S-L[e]	1151	metsox-S-L[p]	1342	METSOX1tex	1
109	h2o	98	h	1343	METSOX2abcpp	0
109	h2o	281	adp	1343	METSOX2abcpp	0
109	h2o	822	metsox-R-L	1343	METSOX2abcpp	0
109	h2o	962	pi	1343	METSOX2abcpp	0
135	atp	98	h	1343	METSOX2abcpp	0
135	atp	281	adp	1343	METSOX2abcpp	1
135	atp	822	metsox-R-L	1343	METSOX2abcpp	0
135	atp	962	pi	1343	METSOX2abcpp	0
1152	metsox-R-L[p]	98	h	1343	METSOX2abcpp	0
1152	metsox-R-L[p]	281	adp	1343	METSOX2abcpp	0
1152	metsox-R-L[p]	822	metsox-R-L	1343	METSOX2abcpp	1
1152	metsox-R-L[p]	962	pi	1343	METSOX2abcpp	0
820	metsox-R-L[e]	1152	metsox-R-L[p]	1344	METSOX2tex	1
821	metsox-S-L	109	h2o	1345	METSOXR1	0
821	metsox-S-L	1433	met-L	1345	METSOXR1	1
821	metsox-S-L	1594	trdox	1345	METSOXR1	1
981	trdrd	109	h2o	1345	METSOXR1	0
981	trdrd	1433	met-L	1345	METSOXR1	0
981	trdrd	1594	trdox	1345	METSOXR1	1
822	metsox-R-L	109	h2o	1346	METSOXR2	0
822	metsox-R-L	1433	met-L	1346	METSOXR2	1
822	metsox-R-L	1594	trdox	1346	METSOXR2	1
981	trdrd	109	h2o	1346	METSOXR2	0
981	trdrd	1433	met-L	1346	METSOXR2	0
981	trdrd	1594	trdox	1346	METSOXR2	1
823	met-L[e]	1153	met-L[p]	1347	METtex	1
135	atp	177	amp	1348	METTRS	1
135	atp	1192	ppi	1348	METTRS	0
135	atp	1400	mettrna	1348	METTRS	0
1433	met-L	177	amp	1348	METTRS	0
1433	met-L	1192	ppi	1348	METTRS	0
1433	met-L	1400	mettrna	1348	METTRS	1
1638	trnamet	177	amp	1348	METTRS	0
1638	trnamet	1192	ppi	1348	METTRS	0
1638	trnamet	1400	mettrna	1348	METTRS	1

98	h	437	h[p]	1349	MG2t3_2pp	0
98	h	1320	mg2	1349	MG2t3_2pp	0
825	mg2[p]	437	h[p]	1349	MG2t3_2pp	0
825	mg2[p]	1320	mg2	1349	MG2t3_2pp	0
824	mg2[e]	825	mg2[p]	1350	MG2tex	0
825	mg2[p]	1320	mg2	1351	MG2tpp	0
109	h2o	98	h	1352	MG2uabcpp	0
109	h2o	281	adp	1352	MG2uabcpp	0
109	h2o	962	pi	1352	MG2uabcpp	0
109	h2o	1320	mg2	1352	MG2uabcpp	0
135	atp	98	h	1352	MG2uabcpp	0
135	atp	281	adp	1352	MG2uabcpp	1
135	atp	962	pi	1352	MG2uabcpp	0
135	atp	1320	mg2	1352	MG2uabcpp	0
825	mg2[p]	98	h	1352	MG2uabcpp	0
825	mg2[p]	281	adp	1352	MG2uabcpp	0
825	mg2[p]	962	pi	1352	MG2uabcpp	0
825	mg2[p]	1320	mg2	1352	MG2uabcpp	0
826	dhap	962	pi	1353	MGSA	0
826	dhap	1321	mthgxl	1353	MGSA	1
109	h2o	962	pi	1354	MI1PP	0
109	h2o	1296	inost	1354	MI1PP	0
1434	mi1p-D	962	pi	1354	MI1PP	0
1434	mi1p-D	1296	inost	1354	MI1PP	1
109	h2o	808	micit	1355	MICITD	0
827	2mcacn	808	micit	1355	MICITD	1
828	minohp[e]	1322	minohp[p]	1356	MINOHPtexi	1
684	h2o[p]	217	alaala[p]	1357	MLDCP1App	0
684	h2o[p]	1431	murein3px4p[p]	1357	MLDCP1App	0
1428	murein5px4p[p]	217	alaala[p]	1357	MLDCP1App	1
1428	murein5px4p[p]	1431	murein3px4p[p]	1357	MLDCP1App	1
684	h2o[p]	384	ala-D[p]	1358	MLDCP1Bpp	0
684	h2o[p]	830	murein4p3p[p]	1358	MLDCP1Bpp	0
829	murein4p4p[p]	384	ala-D[p]	1358	MLDCP1Bpp	1
829	murein4p4p[p]	830	murein4p3p[p]	1358	MLDCP1Bpp	1
684	h2o[p]	217	alaala[p]	1359	MLDCP2App	0
684	h2o[p]	1323	murein5p3p[p]	1359	MLDCP2App	0
809	murein5p5p[p]	217	alaala[p]	1359	MLDCP2App	1
809	murein5p5p[p]	1323	murein5p3p[p]	1359	MLDCP2App	1
684	h2o[p]	384	ala-D[p]	1360	MLDCP2Bpp	0
684	h2o[p]	831	murein3p3p[p]	1360	MLDCP2Bpp	0
830	murein4p3p[p]	384	ala-D[p]	1360	MLDCP2Bpp	1
830	murein4p3p[p]	831	murein3p3p[p]	1360	MLDCP2Bpp	1
684	h2o[p]	217	alaala[p]	1361	MLDCP3App	0
684	h2o[p]	1436	murein3px3p[p]	1361	MLDCP3App	0
1435	murein5px3p[p]	217	alaala[p]	1361	MLDCP3App	1
1435	murein5px3p[p]	1436	murein3px3p[p]	1361	MLDCP3App	1
684	h2o[p]	831	murein3p3p[p]	1362	MLDEP1pp	0
1436	murein3px3p[p]	831	murein3p3p[p]	1362	MLDEP1pp	1
684	h2o[p]	1323	murein5p3p[p]	1363	MLDEP2pp	0
1435	murein5px3p[p]	1323	murein5p3p[p]	1363	MLDEP2pp	1
109	h2o	241	malt	1364	MLTG1	0
109	h2o	1064	glc-D	1364	MLTG1	0
1379	malttr	241	malt	1364	MLTG1	1
1379	malttr	1064	glc-D	1364	MLTG1	1
109	h2o	1064	glc-D	1365	MLTG2	0

109	h2o	1379	malttr	1365	MLTG2	0
1380	maltttr	1064	glc-D	1365	MLTG2	1
1380	maltttr	1379	malttr	1365	MLTG2	1
109	h2o	1064	glc-D	1366	MLTG3	0
109	h2o	1380	maltttr	1366	MLTG3	0
833	maltpt	1064	glc-D	1366	MLTG3	1
833	maltpt	1380	maltttr	1366	MLTG3	1
109	h2o	833	maltpt	1367	MLTG4	0
109	h2o	1064	glc-D	1367	MLTG4	0
834	malthx	833	maltpt	1367	MLTG4	1
834	malthx	1064	glc-D	1367	MLTG4	1
109	h2o	834	malthx	1368	MLTG5	0
109	h2o	1064	glc-D	1368	MLTG5	0
835	malthp	834	malthx	1368	MLTG5	1
835	malthp	1064	glc-D	1368	MLTG5	1
829	murein4p4p[p]	194	anhgm4p[p]	1369	MLTGY1pp	1
830	murein4p3p[p]	192	anhgm3p[p]	1370	MLTGY2pp	1
830	murein4p3p[p]	194	anhgm4p[p]	1370	MLTGY2pp	1
831	murein3p3p[p]	192	anhgm3p[p]	1371	MLTGY3pp	1
832	urein4px4p4p[	194	anhgm4p[p]	1372	MLTGY4pp	1
832	urein4px4p4p[	1430	murein4px4p[p]	1372	MLTGY4pp	1
833	maltpt	589	g1p	1373	MLTP1	1
833	maltpt	1380	maltttr	1373	MLTP1	1
962	pi	589	g1p	1373	MLTP1	0
962	pi	1380	maltttr	1373	MLTP1	0
834	malthx	589	g1p	1374	MLTP2	1
834	malthx	833	maltpt	1374	MLTP2	1
962	pi	589	g1p	1374	MLTP2	0
962	pi	833	maltpt	1374	MLTP2	0
835	malthp	589	g1p	1375	MLTP3	1
835	malthp	834	malthx	1375	MLTP3	1
962	pi	589	g1p	1375	MLTP3	0
962	pi	834	malthx	1375	MLTP3	0
98	h	692	co2	1376	MMCD	0
98	h	948	ppcoa	1376	MMCD	0
1154	mmcoa-S	692	co2	1376	MMCD	0
1154	mmcoa-S	948	ppcoa	1376	MMCD	1
836	mmcoa-R	1154	mmcoa-S	1377	MME	1
437	h[p]	98	h	1378	MMETt2pp	0
437	h[p]	1405	mmet	1378	MMETt2pp	0
1155	mmet[p]	98	h	1378	MMETt2pp	0
1155	mmet[p]	1405	mmet	1378	MMETt2pp	1
837	mmet[e]	1155	mmet[p]	1379	MMETtex	1
838	succoa	836	mmcoa-R	1380	MMM2	1
437	h[p]	98	h	1381	MN2t3pp	0
437	h[p]	839	mn2[p]	1381	MN2t3pp	0
1324	mn2	98	h	1381	MN2t3pp	0
1324	mn2	839	mn2[p]	1381	MN2t3pp	0
839	mn2[p]	1324	mn2	1382	MN2tpp	0
109	h2o	962	pi	1383	MN6PP	0
109	h2o	1325	man	1383	MN6PP	0
799	man6p	962	pi	1383	MN6PP	0
799	man6p	1325	man	1383	MN6PP	1
840	mnl[p]	788	mnl1p	1384	MNLptspp	1
840	mnl[p]	1148	pyr	1384	MNLptspp	0
959	pep	788	mnl1p	1384	MNLptspp	0

959	pep	1148	pyr	1384	MNLptspp	1
841	mnl[e]	840	mnl[p]	1385	MNLtex	1
802	mana	109	h2o	1386	MNNH	0
802	mana	404	2ddglcn	1386	MNNH	1
437	h[p]	98	h	1387	MNt2pp	0
437	h[p]	1324	mn2	1387	MNt2pp	0
839	mn2[p]	98	h	1387	MNt2pp	0
839	mn2[p]	1324	mn2	1387	MNt2pp	0
842	mn2[e]	839	mn2[p]	1388	MNtex	0
843	ckdo	98	h	1389	MOAT	0
843	ckdo	330	cmp	1389	MOAT	1
843	ckdo	1438	kdolipid4	1389	MOAT	1
1437	lipidA	98	h	1389	MOAT	0
1437	lipidA	330	cmp	1389	MOAT	0
1437	lipidA	1438	kdolipid4	1389	MOAT	1
843	ckdo	98	h	1390	MOAT2	0
843	ckdo	330	cmp	1390	MOAT2	1
843	ckdo	1393	kdo2lipid4	1390	MOAT2	1
1438	kdolipid4	98	h	1390	MOAT2	0
1438	kdolipid4	330	cmp	1390	MOAT2	0
1438	kdolipid4	1393	kdo2lipid4	1390	MOAT2	1
843	ckdo	98	h	1391	MOAT3C	0
843	ckdo	330	cmp	1391	MOAT3C	1
843	ckdo	1516	kphphhlipa	1391	MOAT3C	1
1439	phphhlipa	98	h	1391	MOAT3C	0
1439	phphhlipa	330	cmp	1391	MOAT3C	0
1439	phphhlipa	1516	kphphhlipa	1391	MOAT3C	1
109	h2o	98	h	1392	MOBDabcpp	0
109	h2o	281	adp	1392	MOBDabcpp	0
109	h2o	962	pi	1392	MOBDabcpp	0
109	h2o	1657	mobd	1392	MOBDabcpp	0
135	atp	98	h	1392	MOBDabcpp	0
135	atp	281	adp	1392	MOBDabcpp	1
135	atp	962	pi	1392	MOBDabcpp	0
135	atp	1657	mobd	1392	MOBDabcpp	0
1156	mobd[p]	98	h	1392	MOBDabcpp	0
1156	mobd[p]	281	adp	1392	MOBDabcpp	0
1156	mobd[p]	962	pi	1392	MOBDabcpp	0
1156	mobd[p]	1657	mobd	1392	MOBDabcpp	1
844	mobd[e]	1156	mobd[p]	1393	MOBDtex	1
109	h2o	453	2dhp	1394	MOHMT	0
109	h2o	1403	thf	1394	MOHMT	0
729	3mob	453	2dhp	1394	MOHMT	1
729	3mob	1403	thf	1394	MOHMT	0
848	mlthf	453	2dhp	1394	MOHMT	1
848	mlthf	1403	thf	1394	MOHMT	1
845	uaagmda	98	h	1395	MPTG	0
845	uaagmda	809	murein5p5p[p]	1395	MPTG	1
845	uaagmda	1543	udcpdp	1395	MPTG	1
809	murein5p5p[p]	98	h	1396	MPTG2	0
809	murein5p5p[p]	810	murein5p5p5p[p]	1396	MPTG2	1
809	murein5p5p[p]	1543	udcpdp	1396	MPTG2	0
845	uaagmda	98	h	1396	MPTG2	0
845	uaagmda	810	murein5p5p5p[p]	1396	MPTG2	1
845	uaagmda	1543	udcpdp	1396	MPTG2	1
109	h2o	98	h	1397	MSO3abcpp	0

109	h2o	281	adp	1397	MSO3abcpp	0
109	h2o	962	pi	1397	MSO3abcpp	0
109	h2o	1397	mso3	1397	MSO3abcpp	0
135	atp	98	h	1397	MSO3abcpp	0
135	atp	281	adp	1397	MSO3abcpp	1
135	atp	962	pi	1397	MSO3abcpp	0
135	atp	1397	mso3	1397	MSO3abcpp	0
1157	mso3[p]	98	h	1397	MSO3abcpp	0
1157	mso3[p]	281	adp	1397	MSO3abcpp	0
1157	mso3[p]	962	pi	1397	MSO3abcpp	0
1157	mso3[p]	1397	mso3	1397	MSO3abcpp	1
846	mso3[e]	1157	mso3[p]	1398	MSO3tex	1
109	h2o	174	ade	1399	MTAN	0
109	h2o	850	5mtr	1399	MTAN	0
847	5mta	174	ade	1399	MTAN	0
847	5mta	850	5mtr	1399	MTAN	1
109	h2o	98	h	1400	MTHFC	0
109	h2o	210	10fthf	1400	MTHFC	0
1158	methf	98	h	1400	MTHFC	0
1158	methf	210	10fthf	1400	MTHFC	1
459	nadp	871	nadph	1401	MTHFD	1
459	nadp	1158	methf	1401	MTHFD	0
848	mlthf	871	nadph	1401	MTHFD	0
848	mlthf	1158	methf	1401	MTHFD	1
98	h	818	5mthf	1402	MTHFR2	0
98	h	856	nad	1402	MTHFR2	0
848	mlthf	818	5mthf	1402	MTHFR2	1
848	mlthf	856	nad	1402	MTHFR2	0
870	nadh	818	5mthf	1402	MTHFR2	0
870	nadh	856	nad	1402	MTHFR2	1
849	5mdr1p	812	5mdru1p	1403	MTRI	1
135	atp	98	h	1404	MTRK	0
135	atp	281	adp	1404	MTRK	1
135	atp	849	5mdr1p	1404	MTRK	0
850	5mtr	98	h	1404	MTRK	0
850	5mtr	281	adp	1404	MTRK	0
850	5mtr	849	5mdr1p	1404	MTRK	1
109	h2o	292	h2o2	1405	MTRPOX	0
109	h2o	505	fald	1405	MTRPOX	0
109	h2o	1538	trp-L	1405	MTRPOX	0
851	Nmtrp	292	h2o2	1405	MTRPOX	0
851	Nmtrp	505	fald	1405	MTRPOX	1
851	Nmtrp	1538	trp-L	1405	MTRPOX	1
928	o2	292	h2o2	1405	MTRPOX	0
928	o2	505	fald	1405	MTRPOX	0
928	o2	1538	trp-L	1405	MTRPOX	0
852	n2o[e]	853	n2o[p]	1406	N2Otex	0
853	n2o[p]	1159	n2o	1407	N2Otpp	0
109	h2o	147	ac	1408	NACODA	0
109	h2o	565	glu5sa	1408	NACODA	0
207	acg5sa	147	ac	1408	NACODA	1
207	acg5sa	565	glu5sa	1408	NACODA	1
854	nac[e]	855	nac[p]	1409	NACtex	1
855	nac[p]	1326	nac	1410	NACtpp	1
109	h2o	98	h	1411	NADDP	0
109	h2o	177	amp	1411	NADDP	0

109	h2o	1328	nmn	1411	NADDP	0
856	nad	98	h	1411	NADDP	0
856	nad	177	amp	1411	NADDP	1
856	nad	1328	nmn	1411	NADDP	1
98	h	856	nad	1412	NADH10	0
98	h	867	mql8	1412	NADH10	0
870	nadh	856	nad	1412	NADH10	1
870	nadh	867	mql8	1412	NADH10	0
1282	mqn8	856	nad	1412	NADH10	0
1282	mqn8	867	mql8	1412	NADH10	1
98	h	437	h[p]	1413	NADH16pp	0
98	h	856	nad	1413	NADH16pp	0
98	h	1443	q8h2	1413	NADH16pp	0
870	nadh	437	h[p]	1413	NADH16pp	0
870	nadh	856	nad	1413	NADH16pp	1
870	nadh	1443	q8h2	1413	NADH16pp	0
1003	q8	437	h[p]	1413	NADH16pp	0
1003	q8	856	nad	1413	NADH16pp	0
1003	q8	1443	q8h2	1413	NADH16pp	1
98	h	437	h[p]	1414	NADH17pp	0
98	h	856	nad	1414	NADH17pp	0
98	h	867	mql8	1414	NADH17pp	0
870	nadh	437	h[p]	1414	NADH17pp	0
870	nadh	856	nad	1414	NADH17pp	1
870	nadh	867	mql8	1414	NADH17pp	0
1282	mqn8	437	h[p]	1414	NADH17pp	0
1282	mqn8	856	nad	1414	NADH17pp	0
1282	mqn8	867	mql8	1414	NADH17pp	1
98	h	245	2dmmql8	1415	NADH18pp	0
98	h	437	h[p]	1415	NADH18pp	0
98	h	856	nad	1415	NADH18pp	0
560	2dmmq8	245	2dmmql8	1415	NADH18pp	1
560	2dmmq8	437	h[p]	1415	NADH18pp	0
560	2dmmq8	856	nad	1415	NADH18pp	0
870	nadh	245	2dmmql8	1415	NADH18pp	0
870	nadh	437	h[p]	1415	NADH18pp	0
870	nadh	856	nad	1415	NADH18pp	1
98	h	856	nad	1416	NADH5	0
98	h	1443	q8h2	1416	NADH5	0
870	nadh	856	nad	1416	NADH5	1
870	nadh	1443	q8h2	1416	NADH5	0
1003	q8	856	nad	1416	NADH5	0
1003	q8	1443	q8h2	1416	NADH5	1
98	h	245	2dmmql8	1417	NADH9	0
98	h	856	nad	1417	NADH9	0
560	2dmmq8	245	2dmmql8	1417	NADH9	1
560	2dmmq8	856	nad	1417	NADH9	0
870	nadh	245	2dmmql8	1417	NADH9	0
870	nadh	856	nad	1417	NADH9	1
135	atp	98	h	1418	NADK	0
135	atp	281	adp	1418	NADK	1
135	atp	459	nadp	1418	NADK	0
856	nad	98	h	1418	NADK	0
856	nad	281	adp	1418	NADK	0
856	nad	459	nadp	1418	NADK	1
109	h2o	98	h	1419	NADN	0

109	h2o	185	adprib	1419	NADN	0
109	h2o	1441	ncam	1419	NADN	0
856	nad	98	h	1419	NADN	0
856	nad	185	adprib	1419	NADN	1
856	nad	1441	ncam	1419	NADN	1
98	h	459	nadp	1420	NADPHQR2	0
98	h	1443	q8h2	1420	NADPHQR2	0
871	nadph	459	nadp	1420	NADPHQR2	1
871	nadph	1443	q8h2	1420	NADPHQR2	0
1003	q8	459	nadp	1420	NADPHQR2	0
1003	q8	1443	q8h2	1420	NADPHQR2	1
98	h	459	nadp	1421	NADPHQR3	0
98	h	867	mql8	1421	NADPHQR3	0
871	nadph	459	nadp	1421	NADPHQR3	1
871	nadph	867	mql8	1421	NADPHQR3	0
1282	mqn8	459	nadp	1421	NADPHQR3	0
1282	mqn8	867	mql8	1421	NADPHQR3	1
98	h	245	2dmmql8	1422	NADPHQR4	0
98	h	459	nadp	1422	NADPHQR4	0
560	2dmmq8	245	2dmmql8	1422	NADPHQR4	1
560	2dmmq8	459	nadp	1422	NADPHQR4	0
871	nadph	245	2dmmql8	1422	NADPHQR4	0
871	nadph	459	nadp	1422	NADPHQR4	1
109	h2o	856	nad	1423	NADPPPS	0
109	h2o	962	pi	1423	NADPPPS	0
459	nadp	856	nad	1423	NADPPPS	1
459	nadp	962	pi	1423	NADPPPS	0
135	atp	98	h	1424	NADS1	0
135	atp	177	amp	1424	NADS1	1
135	atp	856	nad	1424	NADS1	0
135	atp	1192	ppi	1424	NADS1	0
1160	nh4	98	h	1424	NADS1	0
1160	nh4	177	amp	1424	NADS1	0
1160	nh4	856	nad	1424	NADS1	0
1160	nh4	1192	ppi	1424	NADS1	0
1161	dnad	98	h	1424	NADS1	0
1161	dnad	177	amp	1424	NADS1	0
1161	dnad	856	nad	1424	NADS1	1
1161	dnad	1192	ppi	1424	NADS1	0
856	nad	459	nadp	1425	NADTRHD	0
856	nad	870	nadh	1425	NADTRHD	1
871	nadph	459	nadp	1425	NADTRHD	1
871	nadph	870	nadh	1425	NADTRHD	0
109	h2o	281	adp	1426	NAMNPP	0
109	h2o	962	pi	1426	NAMNPP	0
109	h2o	1192	ppi	1426	NAMNPP	0
109	h2o	1442	nicrnt	1426	NAMNPP	0
135	atp	281	adp	1426	NAMNPP	1
135	atp	962	pi	1426	NAMNPP	0
135	atp	1192	ppi	1426	NAMNPP	0
135	atp	1442	nicrnt	1426	NAMNPP	0
1052	prpp	281	adp	1426	NAMNPP	0
1052	prpp	962	pi	1426	NAMNPP	0
1052	prpp	1192	ppi	1426	NAMNPP	0
1052	prpp	1442	nicrnt	1426	NAMNPP	1
1326	nac	281	adp	1426	NAMNPP	0

1326	nac	962	pi	1426	NAMNPP	0
1326	nac	1192	ppi	1426	NAMNPP	0
1326	nac	1442	nicrnt	1426	NAMNPP	1
437	h[p]	98	h	1427	NAt3_1p5pp	0
437	h[p]	941	na1[p]	1427	NAt3_1p5pp	0
1344	na1	98	h	1427	NAt3_1p5pp	0
1344	na1	941	na1[p]	1427	NAt3_1p5pp	0
437	h[p]	98	h	1428	NAt3_2pp	0
437	h[p]	941	na1[p]	1428	NAt3_2pp	0
1344	na1	98	h	1428	NAt3_2pp	0
1344	na1	941	na1[p]	1428	NAt3_2pp	0
437	h[p]	98	h	1429	NAt3pp	0
437	h[p]	941	na1[p]	1429	NAt3pp	0
1344	na1	98	h	1429	NAt3pp	0
1344	na1	941	na1[p]	1429	NAt3pp	0
857	na1[e]	941	na1[p]	1430	NAtex	0
135	atp	281	adp	1431	NDPK1	1
135	atp	673	gtp	1431	NDPK1	0
798	gdp	281	adp	1431	NDPK1	0
798	gdp	673	gtp	1431	NDPK1	1
135	atp	281	adp	1432	NDPK2	1
135	atp	1447	utp	1432	NDPK2	0
1440	udp	281	adp	1432	NDPK2	0
1440	udp	1447	utp	1432	NDPK2	1
135	atp	281	adp	1433	NDPK3	1
135	atp	392	ctp	1433	NDPK3	0
980	cdp	281	adp	1433	NDPK3	0
980	cdp	392	ctp	1433	NDPK3	1
135	atp	281	adp	1434	NDPK4	1
135	atp	555	dttp	1434	NDPK4	0
1239	dtdp	281	adp	1434	NDPK4	0
1239	dtdp	555	dttp	1434	NDPK4	1
135	atp	281	adp	1435	NDPK5	1
135	atp	892	dgtp	1435	NDPK5	0
1349	dgdp	281	adp	1435	NDPK5	0
1349	dgdp	892	dgtp	1435	NDPK5	1
135	atp	281	adp	1436	NDPK6	1
135	atp	466	dutp	1436	NDPK6	0
1351	dudp	281	adp	1436	NDPK6	0
1351	dudp	466	dutp	1436	NDPK6	1
135	atp	281	adp	1437	NDPK7	1
135	atp	396	dctp	1437	NDPK7	0
1350	dcdp	281	adp	1437	NDPK7	0
1350	dcdp	396	dctp	1437	NDPK7	1
135	atp	281	adp	1438	NDPK8	1
135	atp	393	datp	1438	NDPK8	0
1348	dadp	281	adp	1438	NDPK8	0
1348	dadp	393	datp	1438	NDPK8	1
858	nh4[e]	859	nh4[p]	1439	NH4tex	0
859	nh4[p]	1160	nh4	1440	NH4tpp	0
98	h	109	h2o	1441	NHFRBO	0
98	h	856	nad	1441	NHFRBO	0
98	h	1159	n2o	1441	NHFRBO	0
870	nadh	109	h2o	1441	NHFRBO	0
870	nadh	856	nad	1441	NHFRBO	1
870	nadh	1159	n2o	1441	NHFRBO	0

1163	no	109	h2o	1441	NHFRBO	0
1163	no	856	nad	1441	NHFRBO	0
1163	no	1159	n2o	1441	NHFRBO	0
109	h2o	98	h	1442	NI2abcpp	0
109	h2o	281	adp	1442	NI2abcpp	0
109	h2o	861	ni2[p]	1442	NI2abcpp	0
109	h2o	962	pi	1442	NI2abcpp	0
135	atp	98	h	1442	NI2abcpp	0
135	atp	281	adp	1442	NI2abcpp	1
135	atp	861	ni2[p]	1442	NI2abcpp	0
135	atp	962	pi	1442	NI2abcpp	0
1327	ni2	98	h	1442	NI2abcpp	0
1327	ni2	281	adp	1442	NI2abcpp	0
1327	ni2	861	ni2[p]	1442	NI2abcpp	0
1327	ni2	962	pi	1442	NI2abcpp	0
437	h[p]	98	h	1443	NI2t3pp	0
437	h[p]	861	ni2[p]	1443	NI2t3pp	0
1327	ni2	98	h	1443	NI2t3pp	0
1327	ni2	861	ni2[p]	1443	NI2t3pp	0
860	ni2[e]	861	ni2[p]	1444	NI2tex	0
861	ni2[p]	1327	ni2	1445	NI2tpp	0
109	h2o	98	h	1446	NI2uabcpp	0
109	h2o	281	adp	1446	NI2uabcpp	0
109	h2o	962	pi	1446	NI2uabcpp	0
109	h2o	1327	ni2	1446	NI2uabcpp	0
135	atp	98	h	1446	NI2uabcpp	0
135	atp	281	adp	1446	NI2uabcpp	1
135	atp	962	pi	1446	NI2uabcpp	0
135	atp	1327	ni2	1446	NI2uabcpp	0
861	ni2[p]	98	h	1446	NI2uabcpp	0
861	ni2[p]	281	adp	1446	NI2uabcpp	0
861	ni2[p]	962	pi	1446	NI2uabcpp	0
861	ni2[p]	1327	ni2	1446	NI2uabcpp	0
98	h	856	nad	1447	NMNAT	0
98	h	1192	ppi	1447	NMNAT	0
135	atp	856	nad	1447	NMNAT	1
135	atp	1192	ppi	1447	NMNAT	0
1328	nmn	856	nad	1447	NMNAT	1
1328	nmn	1192	ppi	1447	NMNAT	0
109	h2o	1160	nh4	1448	NMNDA	0
109	h2o	1442	nicrnt	1448	NMNDA	0
1328	nmn	1160	nh4	1448	NMNDA	0
1328	nmn	1442	nicrnt	1448	NMNDA	1
109	h2o	98	h	1449	NMNN	0
109	h2o	983	r5p	1449	NMNN	0
109	h2o	1441	ncam	1449	NMNN	0
1328	nmn	98	h	1449	NMNN	0
1328	nmn	983	r5p	1449	NMNN	1
1328	nmn	1441	ncam	1449	NMNN	1
862	nmn[p]	1328	nmn	1450	NMNPtpp	1
109	h2o	98	h	1451	NMNt7pp	0
109	h2o	983	r5p	1451	NMNt7pp	0
109	h2o	1441	ncam	1451	NMNt7pp	0
862	nmn[p]	98	h	1451	NMNt7pp	0
862	nmn[p]	983	r5p	1451	NMNt7pp	1
862	nmn[p]	1441	ncam	1451	NMNt7pp	1

863	nmn[e]	862	nmn[p]	1452	NMNtex	1
109	h2o	1160	nh4	1453	NNAM	0
109	h2o	1326	nac	1453	NNAM	0
1441	ncam	1160	nh4	1453	NNAM	0
1441	ncam	1326	nac	1453	NNAM	1
98	h	1161	dnad	1454	NNATr	0
98	h	1192	ppi	1454	NNATr	0
135	atp	1161	dnad	1454	NNATr	1
135	atp	1192	ppi	1454	NNATr	0
1442	nicrnt	1161	dnad	1454	NNATr	1
1442	nicrnt	1192	ppi	1454	NNATr	0
864	dmbzid	98	h	1455	NNDMBRT	0
864	dmbzid	984	5prdmbz	1455	NNDMBRT	1
864	dmbzid	1326	nac	1455	NNDMBRT	0
1442	nicrnt	98	h	1455	NNDMBRT	0
1442	nicrnt	984	5prdmbz	1455	NNDMBRT	1
1442	nicrnt	1326	nac	1455	NNDMBRT	1
98	h	692	co2	1456	NNDPR	0
98	h	1192	ppi	1456	NNDPR	0
98	h	1442	nicrnt	1456	NNDPR	0
1052	prpp	692	co2	1456	NNDPR	0
1052	prpp	1192	ppi	1456	NNDPR	0
1052	prpp	1442	nicrnt	1456	NNDPR	1
1639	quln	692	co2	1456	NNDPR	0
1639	quln	1192	ppi	1456	NNDPR	0
1639	quln	1442	nicrnt	1456	NNDPR	1
437	h[p]	98	h	1457	NO2t2rpp	0
437	h[p]	868	no2	1457	NO2t2rpp	0
1162	no2[p]	98	h	1457	NO2t2rpp	0
1162	no2[p]	868	no2	1457	NO2t2rpp	0
865	no2[e]	1162	no2[p]	1458	NO2tex	0
866	no3[p]	684	h2o[p]	1459	NO3R1bpp	0
866	no3[p]	1003	q8	1459	NO3R1bpp	0
866	no3[p]	1162	no2[p]	1459	NO3R1bpp	0
1443	q8h2	684	h2o[p]	1459	NO3R1bpp	0
1443	q8h2	1003	q8	1459	NO3R1bpp	1
1443	q8h2	1162	no2[p]	1459	NO3R1bpp	0
98	h	109	h2o	1460	NO3R1pp	0
98	h	437	h[p]	1460	NO3R1pp	0
98	h	868	no2	1460	NO3R1pp	0
98	h	1003	q8	1460	NO3R1pp	0
1443	q8h2	109	h2o	1460	NO3R1pp	0
1443	q8h2	437	h[p]	1460	NO3R1pp	0
1443	q8h2	868	no2	1460	NO3R1pp	0
1443	q8h2	1003	q8	1460	NO3R1pp	1
1444	no3	109	h2o	1460	NO3R1pp	0
1444	no3	437	h[p]	1460	NO3R1pp	0
1444	no3	868	no2	1460	NO3R1pp	0
1444	no3	1003	q8	1460	NO3R1pp	0
866	no3[p]	684	h2o[p]	1461	NO3R2bpp	0
866	no3[p]	1162	no2[p]	1461	NO3R2bpp	0
866	no3[p]	1282	mqn8	1461	NO3R2bpp	0
867	mql8	684	h2o[p]	1461	NO3R2bpp	0
867	mql8	1162	no2[p]	1461	NO3R2bpp	0
867	mql8	1282	mqn8	1461	NO3R2bpp	1
98	h	109	h2o	1462	NO3R2pp	0

98	h	437	h[p]	1462	NO3R2pp	0
98	h	868	no2	1462	NO3R2pp	0
98	h	1282	mqn8	1462	NO3R2pp	0
867	mql8	109	h2o	1462	NO3R2pp	0
867	mql8	437	h[p]	1462	NO3R2pp	0
867	mql8	868	no2	1462	NO3R2pp	0
867	mql8	1282	mqn8	1462	NO3R2pp	1
1444	no3	109	h2o	1462	NO3R2pp	0
1444	no3	437	h[p]	1462	NO3R2pp	0
1444	no3	868	no2	1462	NO3R2pp	0
1444	no3	1282	mqn8	1462	NO3R2pp	0
866	no3[p]	1162	no2[p]	1463	NO3t7pp	0
866	no3[p]	1444	no3	1463	NO3t7pp	0
868	no2	1162	no2[p]	1463	NO3t7pp	0
868	no2	1444	no3	1463	NO3t7pp	0
869	no3[e]	866	no3[p]	1464	NO3tex	0
870	nadh	98	h	1465	NODOx	0
870	nadh	856	nad	1465	NODOx	1
870	nadh	1444	no3	1465	NODOx	0
928	o2	98	h	1465	NODOx	0
928	o2	856	nad	1465	NODOx	0
928	o2	1444	no3	1465	NODOx	0
1163	no	98	h	1465	NODOx	0
1163	no	856	nad	1465	NODOx	0
1163	no	1444	no3	1465	NODOx	0
871	nadph	98	h	1466	NODOy	0
871	nadph	459	nadp	1466	NODOy	1
871	nadph	1444	no3	1466	NODOy	0
928	o2	98	h	1466	NODOy	0
928	o2	459	nadp	1466	NODOy	0
928	o2	1444	no3	1466	NODOy	0
1163	no	98	h	1466	NODOy	0
1163	no	459	nadp	1466	NODOy	0
1163	no	1444	no3	1466	NODOy	0
872	no[e]	873	no[p]	1467	NOtex	0
873	no[p]	1163	no	1468	NOtpp	0
874	sbzcoa	422	dhna	1469	NPHS	1
874	sbzcoa	927	coa	1469	NPHS	1
109	h2o	463	duri	1470	NTD1	0
109	h2o	962	pi	1470	NTD1	0
875	dump	463	duri	1470	NTD1	1
875	dump	962	pi	1470	NTD1	0
109	h2o	962	pi	1471	NTD10	0
109	h2o	1508	xtsn	1471	NTD10	0
1445	xmp	962	pi	1471	NTD10	0
1445	xmp	1508	xtsn	1471	NTD10	1
684	h2o[p]	1173	pi[p]	1472	NTD10pp	0
684	h2o[p]	1233	xtsn[p]	1472	NTD10pp	0
1232	xmp[p]	1173	pi[p]	1472	NTD10pp	0
1232	xmp[p]	1233	xtsn[p]	1472	NTD10pp	1
109	h2o	962	pi	1473	NTD11	0
109	h2o	967	ins	1473	NTD11	0
1287	imp	962	pi	1473	NTD11	0
1287	imp	967	ins	1473	NTD11	1
684	h2o[p]	1135	ins[p]	1474	NTD11pp	0
684	h2o[p]	1173	pi[p]	1474	NTD11pp	0

1133	imp[p]	1135	ins[p]	1474	NTD11pp	1
1133	imp[p]	1173	pi[p]	1474	NTD11pp	0
109	h2o	962	pi	1475	NTD12	0
109	h2o	968	din	1475	NTD12	0
876	dimp	962	pi	1475	NTD12	0
876	dimp	968	din	1475	NTD12	1
684	h2o[p]	433	din[p]	1476	NTD12pp	0
684	h2o[p]	1173	pi[p]	1476	NTD12pp	0
877	dimp[p]	433	din[p]	1476	NTD12pp	1
877	dimp[p]	1173	pi[p]	1476	NTD12pp	0
684	h2o[p]	464	duri[p]	1477	NTD1pp	0
684	h2o[p]	1173	pi[p]	1477	NTD1pp	0
878	dump[p]	464	duri[p]	1477	NTD1pp	1
878	dump[p]	1173	pi[p]	1477	NTD1pp	0
109	h2o	962	pi	1478	NTD2	0
109	h2o	1512	uri	1478	NTD2	0
1242	ump	962	pi	1478	NTD2	0
1242	ump	1512	uri	1478	NTD2	1
684	h2o[p]	1173	pi[p]	1479	NTD2pp	0
684	h2o[p]	1229	uri[p]	1479	NTD2pp	0
1226	ump[p]	1173	pi[p]	1479	NTD2pp	0
1226	ump[p]	1229	uri[p]	1479	NTD2pp	1
109	h2o	397	dcyt	1480	NTD3	0
109	h2o	962	pi	1480	NTD3	0
879	dcmp	397	dcyt	1480	NTD3	1
879	dcmp	962	pi	1480	NTD3	0
684	h2o[p]	398	dcyt[p]	1481	NTD3pp	0
684	h2o[p]	1173	pi[p]	1481	NTD3pp	0
880	dcmp[p]	398	dcyt[p]	1481	NTD3pp	1
880	dcmp[p]	1173	pi[p]	1481	NTD3pp	0
109	h2o	371	cytd	1482	NTD4	0
109	h2o	962	pi	1482	NTD4	0
330	cmp	371	cytd	1482	NTD4	1
330	cmp	962	pi	1482	NTD4	0
684	h2o[p]	372	cytd[p]	1483	NTD4pp	0
684	h2o[p]	1173	pi[p]	1483	NTD4pp	0
881	cmp[p]	372	cytd[p]	1483	NTD4pp	1
881	cmp[p]	1173	pi[p]	1483	NTD4pp	0
109	h2o	962	pi	1484	NTD5	0
109	h2o	1533	thymd	1484	NTD5	0
882	dtmp	962	pi	1484	NTD5	0
882	dtmp	1533	thymd	1484	NTD5	1
684	h2o[p]	1173	pi[p]	1485	NTD5pp	0
684	h2o[p]	1205	thymd[p]	1485	NTD5pp	0
883	dtmp[p]	1173	pi[p]	1485	NTD5pp	0
883	dtmp[p]	1205	thymd[p]	1485	NTD5pp	1
109	h2o	374	dad-2	1486	NTD6	0
109	h2o	962	pi	1486	NTD6	0
884	damp	374	dad-2	1486	NTD6	1
884	damp	962	pi	1486	NTD6	0
684	h2o[p]	375	dad-2[p]	1487	NTD6pp	0
684	h2o[p]	1173	pi[p]	1487	NTD6pp	0
885	damp[p]	375	dad-2[p]	1487	NTD6pp	1
885	damp[p]	1173	pi[p]	1487	NTD6pp	0
109	h2o	171	adn	1488	NTD7	0
109	h2o	962	pi	1488	NTD7	0

177	amp	171	adn	1488	NTD7	1
177	amp	962	pi	1488	NTD7	0
684	h2o[p]	179	adn[p]	1489	NTD7pp	0
684	h2o[p]	1173	pi[p]	1489	NTD7pp	0
886	amp[p]	179	adn[p]	1489	NTD7pp	1
886	amp[p]	1173	pi[p]	1489	NTD7pp	0
109	h2o	962	pi	1490	NTD8	0
109	h2o	966	dgsn	1490	NTD8	0
887	dgmp	962	pi	1490	NTD8	0
887	dgmp	966	dgsn	1490	NTD8	1
684	h2o[p]	408	dgsn[p]	1491	NTD8pp	0
684	h2o[p]	1173	pi[p]	1491	NTD8pp	0
888	dgmp[p]	408	dgsn[p]	1491	NTD8pp	1
888	dgmp[p]	1173	pi[p]	1491	NTD8pp	0
109	h2o	962	pi	1492	NTD9	0
109	h2o	965	gsn	1492	NTD9	0
650	gmp	962	pi	1492	NTD9	0
650	gmp	965	gsn	1492	NTD9	1
684	h2o[p]	666	gsn[p]	1493	NTD9pp	0
684	h2o[p]	1173	pi[p]	1493	NTD9pp	0
889	gmp[p]	666	gsn[p]	1493	NTD9pp	1
889	gmp[p]	1173	pi[p]	1493	NTD9pp	0
109	h2o	98	h	1494	NTP1	0
109	h2o	281	adp	1494	NTP1	0
109	h2o	962	pi	1494	NTP1	0
135	atp	98	h	1494	NTP1	0
135	atp	281	adp	1494	NTP1	1
135	atp	962	pi	1494	NTP1	0
109	h2o	98	h	1495	NTP10	0
109	h2o	962	pi	1495	NTP10	0
109	h2o	1558	idp	1495	NTP10	0
1249	itp	98	h	1495	NTP10	0
1249	itp	962	pi	1495	NTP10	0
1249	itp	1558	idp	1495	NTP10	1
109	h2o	98	h	1496	NTP11	0
109	h2o	962	pi	1496	NTP11	0
109	h2o	1329	didp	1496	NTP11	0
890	ditp	98	h	1496	NTP11	0
890	ditp	962	pi	1496	NTP11	0
890	ditp	1329	didp	1496	NTP11	1
109	h2o	98	h	1497	NTP12	0
109	h2o	962	pi	1497	NTP12	0
109	h2o	1658	xdp	1497	NTP12	0
1446	xtp	98	h	1497	NTP12	0
1446	xtp	962	pi	1497	NTP12	0
1446	xtp	1658	xdp	1497	NTP12	1
109	h2o	98	h	1498	NTP3	0
109	h2o	798	gdp	1498	NTP3	0
109	h2o	962	pi	1498	NTP3	0
673	gtp	98	h	1498	NTP3	0
673	gtp	798	gdp	1498	NTP3	1
673	gtp	962	pi	1498	NTP3	0
684	h2o[p]	437	h[p]	1499	NTP3pp	0
684	h2o[p]	1116	gdp[p]	1499	NTP3pp	0
684	h2o[p]	1173	pi[p]	1499	NTP3pp	0
891	gtp[p]	437	h[p]	1499	NTP3pp	0

891	gtp[p]	1116	gdp[p]	1499	NTP3pp	1
891	gtp[p]	1173	pi[p]	1499	NTP3pp	0
109	h2o	98	h	1500	NTP5	0
109	h2o	962	pi	1500	NTP5	0
109	h2o	980	cdp	1500	NTP5	0
392	ctp	98	h	1500	NTP5	0
392	ctp	962	pi	1500	NTP5	0
392	ctp	980	cdp	1500	NTP5	1
109	h2o	98	h	1501	NTPP1	0
109	h2o	887	dgmp	1501	NTPP1	0
109	h2o	1192	ppi	1501	NTPP1	0
892	dgtp	98	h	1501	NTPP1	0
892	dgtp	887	dgmp	1501	NTPP1	1
892	dgtp	1192	ppi	1501	NTPP1	0
109	h2o	98	h	1502	NTPP10	0
109	h2o	876	dimp	1502	NTPP10	0
109	h2o	1192	ppi	1502	NTPP10	0
890	ditp	98	h	1502	NTPP10	0
890	ditp	876	dimp	1502	NTPP10	1
890	ditp	1192	ppi	1502	NTPP10	0
109	h2o	98	h	1503	NTPP11	0
109	h2o	1192	ppi	1503	NTPP11	0
109	h2o	1445	xmp	1503	NTPP11	0
1446	xtp	98	h	1503	NTPP11	0
1446	xtp	1192	ppi	1503	NTPP11	0
1446	xtp	1445	xmp	1503	NTPP11	1
109	h2o	98	h	1504	NTPP2	0
109	h2o	650	gmp	1504	NTPP2	0
109	h2o	1192	ppi	1504	NTPP2	0
673	gtp	98	h	1504	NTPP2	0
673	gtp	650	gmp	1504	NTPP2	1
673	gtp	1192	ppi	1504	NTPP2	0
109	h2o	98	h	1505	NTPP3	0
109	h2o	879	dcmp	1505	NTPP3	0
109	h2o	1192	ppi	1505	NTPP3	0
396	dctp	98	h	1505	NTPP3	0
396	dctp	879	dcmp	1505	NTPP3	1
396	dctp	1192	ppi	1505	NTPP3	0
109	h2o	98	h	1506	NTPP4	0
109	h2o	330	cmp	1506	NTPP4	0
109	h2o	1192	ppi	1506	NTPP4	0
392	ctp	98	h	1506	NTPP4	0
392	ctp	330	cmp	1506	NTPP4	1
392	ctp	1192	ppi	1506	NTPP4	0
109	h2o	98	h	1507	NTPP5	0
109	h2o	884	damp	1507	NTPP5	0
109	h2o	1192	ppi	1507	NTPP5	0
393	datp	98	h	1507	NTPP5	0
393	datp	884	damp	1507	NTPP5	1
393	datp	1192	ppi	1507	NTPP5	0
109	h2o	98	h	1508	NTPP6	0
109	h2o	177	amp	1508	NTPP6	0
109	h2o	1192	ppi	1508	NTPP6	0
135	atp	98	h	1508	NTPP6	0
135	atp	177	amp	1508	NTPP6	1
135	atp	1192	ppi	1508	NTPP6	0

109	h2o	98	h	1509	NTPP7	0
109	h2o	882	dtmp	1509	NTPP7	0
109	h2o	1192	ppi	1509	NTPP7	0
555	dttp	98	h	1509	NTPP7	0
555	dttp	882	dtmp	1509	NTPP7	1
555	dttp	1192	ppi	1509	NTPP7	0
109	h2o	98	h	1510	NTPP8	0
109	h2o	1192	ppi	1510	NTPP8	0
109	h2o	1242	ump	1510	NTPP8	0
1447	utp	98	h	1510	NTPP8	0
1447	utp	1192	ppi	1510	NTPP8	0
1447	utp	1242	ump	1510	NTPP8	1
109	h2o	98	h	1511	NTPP9	0
109	h2o	1192	ppi	1511	NTPP9	0
109	h2o	1287	imp	1511	NTPP9	0
1249	itp	98	h	1511	NTPP9	0
1249	itp	1192	ppi	1511	NTPP9	0
1249	itp	1287	imp	1511	NTPP9	1
109	h2o	966	dgsn	1512	NTPTP1	0
109	h2o	1491	pppi	1512	NTPTP1	0
892	dgtp	966	dgsn	1512	NTPTP1	1
892	dgtp	1491	pppi	1512	NTPTP1	0
109	h2o	965	gsn	1513	NTPTP2	0
109	h2o	1491	pppi	1513	NTPTP2	0
673	gtp	965	gsn	1513	NTPTP2	1
673	gtp	1491	pppi	1513	NTPTP2	0
98	h	109	h2o	1514	NTRIR2x	0
98	h	856	nad	1514	NTRIR2x	0
98	h	1160	nh4	1514	NTRIR2x	0
868	no2	109	h2o	1514	NTRIR2x	0
868	no2	856	nad	1514	NTRIR2x	0
868	no2	1160	nh4	1514	NTRIR2x	0
870	nadh	109	h2o	1514	NTRIR2x	0
870	nadh	856	nad	1514	NTRIR2x	1
870	nadh	1160	nh4	1514	NTRIR2x	0
437	h[p]	684	h2o[p]	1515	NTRIR3pp	0
437	h[p]	859	nh4[p]	1515	NTRIR3pp	0
437	h[p]	1003	q8	1515	NTRIR3pp	0
1162	no2[p]	684	h2o[p]	1515	NTRIR3pp	0
1162	no2[p]	859	nh4[p]	1515	NTRIR3pp	0
1162	no2[p]	1003	q8	1515	NTRIR3pp	0
1443	q8h2	684	h2o[p]	1515	NTRIR3pp	0
1443	q8h2	859	nh4[p]	1515	NTRIR3pp	0
1443	q8h2	1003	q8	1515	NTRIR3pp	1
437	h[p]	684	h2o[p]	1516	NTRIR4pp	0
437	h[p]	859	nh4[p]	1516	NTRIR4pp	0
437	h[p]	1282	mqn8	1516	NTRIR4pp	0
867	mql8	684	h2o[p]	1516	NTRIR4pp	0
867	mql8	859	nh4[p]	1516	NTRIR4pp	0
867	mql8	1282	mqn8	1516	NTRIR4pp	1
1162	no2[p]	684	h2o[p]	1516	NTRIR4pp	0
1162	no2[p]	859	nh4[p]	1516	NTRIR4pp	0
1162	no2[p]	1282	mqn8	1516	NTRIR4pp	0
893	o16a4colipa[p]	1330	o16a4colipa[e]	1517	O16A4COLIPAtex	1
337	colipa[p]	437	h[p]	1518	O16A4Lpp	0
337	colipa[p]	893	o16a4colipa[p]	1518	O16A4Lpp	1

337	colipa[p]	1544	udcpdp[p]	1518	O16A4Lpp	0
1448	o16a4und[p]	437	h[p]	1518	O16A4Lpp	0
1448	o16a4und[p]	893	o16a4colipa[p]	1518	O16A4Lpp	1
1448	o16a4und[p]	1544	udcpdp[p]	1518	O16A4Lpp	1
894	o16aund[p]	437	h[p]	1519	O16AP1pp	0
894	o16aund[p]	895	o16a2und[p]	1519	O16AP1pp	1
894	o16aund[p]	1544	udcpdp[p]	1519	O16AP1pp	1
894	o16aund[p]	437	h[p]	1520	O16AP2pp	0
894	o16aund[p]	896	o16a3und[p]	1520	O16AP2pp	1
894	o16aund[p]	1544	udcpdp[p]	1520	O16AP2pp	1
895	o16a2und[p]	437	h[p]	1520	O16AP2pp	0
895	o16a2und[p]	896	o16a3und[p]	1520	O16AP2pp	1
895	o16a2und[p]	1544	udcpdp[p]	1520	O16AP2pp	0
894	o16aund[p]	437	h[p]	1521	O16AP3pp	0
894	o16aund[p]	1448	o16a4und[p]	1521	O16AP3pp	1
894	o16aund[p]	1544	udcpdp[p]	1521	O16AP3pp	1
896	o16a3und[p]	437	h[p]	1521	O16AP3pp	0
896	o16a3und[p]	1448	o16a4und[p]	1521	O16AP3pp	1
896	o16a3und[p]	1544	udcpdp[p]	1521	O16AP3pp	0
128	accoa	899	aragund	1522	O16AT	1
128	accoa	927	coa	1522	O16AT	1
1449	ragund	899	aragund	1522	O16AT	1
1449	ragund	927	coa	1522	O16AT	0
897	o16aund	894	o16aund[p]	1523	O16AUNDtpp	1
898	garagund	98	h	1524	O16GALFT	0
898	garagund	900	gfgaragund	1524	O16GALFT	1
898	garagund	1440	udp	1524	O16GALFT	0
1358	udpgalfur	98	h	1524	O16GALFT	0
1358	udpgalfur	900	gfgaragund	1524	O16GALFT	1
1358	udpgalfur	1440	udp	1524	O16GALFT	1
899	aragund	98	h	1525	O16GLCT1	0
899	aragund	898	garagund	1525	O16GLCT1	1
899	aragund	1440	udp	1525	O16GLCT1	0
1042	udpg	98	h	1525	O16GLCT1	0
1042	udpg	898	garagund	1525	O16GLCT1	1
1042	udpg	1440	udp	1525	O16GLCT1	1
900	gfgaragund	98	h	1526	O16GLCT2	0
900	gfgaragund	897	o16aund	1526	O16GLCT2	1
900	gfgaragund	1440	udp	1526	O16GLCT2	0
1042	udpg	98	h	1526	O16GLCT2	0
1042	udpg	897	o16aund	1526	O16GLCT2	1
1042	udpg	1440	udp	1526	O16GLCT2	1
901	o2s[e]	1164	o2s[p]	1527	O2Stex	0
902	o2[e]	903	o2[p]	1528	O2tex	0
903	o2[p]	928	o2	1529	O2tpp	0
98	h	692	co2	1530	OAADC	0
98	h	1148	pyr	1530	OAADC	0
1318	oaa	692	co2	1530	OAADC	0
1318	oaa	1148	pyr	1530	OAADC	1
146	2obut	536	for	1531	OBTFL	1
146	2obut	948	ppcoa	1531	OBTFL	1
927	coa	536	for	1531	OBTFL	0
927	coa	948	ppcoa	1531	OBTFL	1
904	cbp	98	h	1532	OCBT	0
904	cbp	962	pi	1532	OCBT	0
904	cbp	1165	citr-L	1532	OCBT	1

963	orn	98	h	1532	OCBT	0
963	orn	962	pi	1532	OCBT	0
963	orn	1165	citr-L	1532	OCBT	1
905	ocdca[e]	1331	ocdca[p]	1533	OCDCAtexi	1
906	ocdcea[e]	1332	ocdcea[p]	1534	OCDCEAtexi	1
907	octa[e]	1166	octa[p]	1535	OCTAtex	1
697	frdp	1192	ppi	1536	OCTDPS	0
697	frdp	1333	octdp	1536	OCTDPS	1
726	ipdp	1192	ppi	1536	OCTDPS	0
726	ipdp	1333	octdp	1536	OCTDPS	1
908	odecoa	1334	od2coa	1537	ODECOAI	1
624	glu-L	213	akg	1538	OHPBAT	1
624	glu-L	1364	phthr	1538	OHPBAT	0
1450	ohpb	213	akg	1538	OHPBAT	0
1450	ohpb	1364	phthr	1538	OHPBAT	1
178	amet	98	h	1539	OHPHM	0
178	amet	209	ahcys	1539	OHPHM	1
178	amet	913	2omph	1539	OHPHM	1
909	2ohph	98	h	1539	OHPHM	0
909	2ohph	209	ahcys	1539	OHPHM	0
909	2ohph	913	2omph	1539	OHPHM	1
178	amet	98	h	1540	OMBZLM	0
178	amet	209	ahcys	1540	OMBZLM	1
178	amet	912	2ommbl	1540	OMBZLM	1
910	2ombzl	98	h	1540	OMBZLM	0
910	2ombzl	209	ahcys	1540	OMBZLM	0
910	2ombzl	912	2ommbl	1540	OMBZLM	1
98	h	692	co2	1541	OMCDC	0
98	h	749	4mop	1541	OMCDC	0
911	3c4mop	692	co2	1541	OMCDC	0
911	3c4mop	749	4mop	1541	OMCDC	1
912	2ommbl	445	2omhmbl	1542	OMMBLHX	1
928	o2	445	2omhmbl	1542	OMMBLHX	0
109	h2o	98	h	1543	OMMBLHX3	0
109	h2o	281	adp	1543	OMMBLHX3	0
109	h2o	445	2omhmbl	1543	OMMBLHX3	0
109	h2o	870	nadh	1543	OMMBLHX3	0
109	h2o	962	pi	1543	OMMBLHX3	0
135	atp	98	h	1543	OMMBLHX3	0
135	atp	281	adp	1543	OMMBLHX3	1
135	atp	445	2omhmbl	1543	OMMBLHX3	0
135	atp	870	nadh	1543	OMMBLHX3	0
135	atp	962	pi	1543	OMMBLHX3	0
856	nad	98	h	1543	OMMBLHX3	0
856	nad	281	adp	1543	OMMBLHX3	0
856	nad	445	2omhmbl	1543	OMMBLHX3	0
856	nad	870	nadh	1543	OMMBLHX3	1
856	nad	962	pi	1543	OMMBLHX3	0
912	2ommbl	98	h	1543	OMMBLHX3	0
912	2ommbl	281	adp	1543	OMMBLHX3	0
912	2ommbl	445	2omhmbl	1543	OMMBLHX3	1
912	2ommbl	870	nadh	1543	OMMBLHX3	0
912	2ommbl	962	pi	1543	OMMBLHX3	0
98	h	692	co2	1544	OMPDC	0
98	h	1242	ump	1544	OMPDC	0
918	orot5p	692	co2	1544	OMPDC	0

918	orot5p	1242	ump	1544	OMPDC	1
913	2omph	910	2ombzl	1545	OMPHHX	1
928	o2	910	2ombzl	1545	OMPHHX	0
109	h2o	98	h	1546	OMPHHX3	0
109	h2o	281	adp	1546	OMPHHX3	0
109	h2o	870	nadh	1546	OMPHHX3	0
109	h2o	910	2ombzl	1546	OMPHHX3	0
109	h2o	962	pi	1546	OMPHHX3	0
135	atp	98	h	1546	OMPHHX3	0
135	atp	281	adp	1546	OMPHHX3	1
135	atp	870	nadh	1546	OMPHHX3	0
135	atp	910	2ombzl	1546	OMPHHX3	0
135	atp	962	pi	1546	OMPHHX3	0
856	nad	98	h	1546	OMPHHX3	0
856	nad	281	adp	1546	OMPHHX3	0
856	nad	870	nadh	1546	OMPHHX3	1
856	nad	910	2ombzl	1546	OMPHHX3	0
856	nad	962	pi	1546	OMPHHX3	0
913	2omph	98	h	1546	OMPHHX3	0
913	2omph	281	adp	1546	OMPHHX3	0
913	2omph	870	nadh	1546	OMPHHX3	0
913	2omph	910	2ombzl	1546	OMPHHX3	1
913	2omph	962	pi	1546	OMPHHX3	0
109	h2o	704	4h2opntn	1547	OP4ENH	0
1451	op4en	704	4h2opntn	1547	OP4ENH	1
98	h	692	co2	1548	OPHBDC	0
98	h	915	2oph	1548	OPHBDC	0
914	3ophb	692	co2	1548	OPHBDC	0
914	3ophb	915	2oph	1548	OPHBDC	1
915	2oph	909	2ohph	1549	OPHHX	1
928	o2	909	2ohph	1549	OPHHX	0
109	h2o	98	h	1550	OPHHX3	0
109	h2o	281	adp	1550	OPHHX3	0
109	h2o	870	nadh	1550	OPHHX3	0
109	h2o	909	2ohph	1550	OPHHX3	0
109	h2o	962	pi	1550	OPHHX3	0
135	atp	98	h	1550	OPHHX3	0
135	atp	281	adp	1550	OPHHX3	1
135	atp	870	nadh	1550	OPHHX3	0
135	atp	909	2ohph	1550	OPHHX3	0
135	atp	962	pi	1550	OPHHX3	0
856	nad	98	h	1550	OPHHX3	0
856	nad	281	adp	1550	OPHHX3	0
856	nad	870	nadh	1550	OPHHX3	1
856	nad	909	2ohph	1550	OPHHX3	0
856	nad	962	pi	1550	OPHHX3	0
915	2oph	98	h	1550	OPHHX3	0
915	2oph	281	adp	1550	OPHHX3	0
915	2oph	870	nadh	1550	OPHHX3	0
915	2oph	909	2ohph	1550	OPHHX3	1
915	2oph	962	pi	1550	OPHHX3	0
109	h2o	98	h	1551	ORNabcpp	0
109	h2o	281	adp	1551	ORNabcpp	0
109	h2o	962	pi	1551	ORNabcpp	0
109	h2o	963	orn	1551	ORNabcpp	0
135	atp	98	h	1551	ORNabcpp	0

135	atp	281	adp	1551	ORNabcpp	1
135	atp	962	pi	1551	ORNabcpp	0
135	atp	963	orn	1551	ORNabcpp	0
1167	orn[p]	98	h	1551	ORNabcpp	0
1167	orn[p]	281	adp	1551	ORNabcpp	0
1167	orn[p]	962	pi	1551	ORNabcpp	0
1167	orn[p]	963	orn	1551	ORNabcpp	1
98	h	692	co2	1552	ORNDC	0
98	h	1245	ptrc	1552	ORNDC	0
963	orn	692	co2	1552	ORNDC	0
963	orn	1245	ptrc	1552	ORNDC	1
916	orn[e]	1167	orn[p]	1553	ORNtex	1
437	h[p]	98	h	1554	OROTt2_2pp	0
437	h[p]	1169	orot	1554	OROTt2_2pp	0
1168	orot[p]	98	h	1554	OROTt2_2pp	0
1168	orot[p]	1169	orot	1554	OROTt2_2pp	1
917	orot[e]	1168	orot[p]	1555	OROTtex	1
918	orot5p	1052	prpp	1556	ORPT	1
918	orot5p	1169	orot	1556	ORPT	1
1192	ppi	1052	prpp	1556	ORPT	0
1192	ppi	1169	orot	1556	ORPT	0
919	oxur	443	oxam	1557	OXAMTC	1
919	oxur	904	cbp	1557	OXAMTC	1
962	pi	443	oxam	1557	OXAMTC	0
962	pi	904	cbp	1557	OXAMTC	0
98	h	692	co2	1558	OXGDC2	0
98	h	1521	ssaltpp	1558	OXGDC2	0
213	akg	692	co2	1558	OXGDC2	0
213	akg	1521	ssaltpp	1558	OXGDC2	1
1528	thmpp	692	co2	1558	OXGDC2	0
1528	thmpp	1521	ssaltpp	1558	OXGDC2	1
109	h2o	98	h	1559	P5CD	0
109	h2o	624	glu-L	1559	P5CD	0
109	h2o	870	nadh	1559	P5CD	0
856	nad	98	h	1559	P5CD	0
856	nad	624	glu-L	1559	P5CD	0
856	nad	870	nadh	1559	P5CD	1
920	1pyr5c	98	h	1559	P5CD	0
920	1pyr5c	624	glu-L	1559	P5CD	1
920	1pyr5c	870	nadh	1559	P5CD	0
98	h	459	nadp	1560	P5CR	0
98	h	1498	pro-L	1560	P5CR	0
871	nadph	459	nadp	1560	P5CR	1
871	nadph	1498	pro-L	1560	P5CR	0
920	1pyr5c	459	nadp	1560	P5CR	0
920	1pyr5c	1498	pro-L	1560	P5CR	1
109	h2o	98	h	1561	PA120abcpp	0
109	h2o	281	adp	1561	PA120abcpp	0
109	h2o	962	pi	1561	PA120abcpp	0
109	h2o	1453	pa120[p]	1561	PA120abcpp	0
135	atp	98	h	1561	PA120abcpp	0
135	atp	281	adp	1561	PA120abcpp	1
135	atp	962	pi	1561	PA120abcpp	0
135	atp	1453	pa120[p]	1561	PA120abcpp	0
1452	pa120	98	h	1561	PA120abcpp	0
1452	pa120	281	adp	1561	PA120abcpp	0

1452	pa120	962	pi	1561	PA120abcpp	0
1452	pa120	1453	pa120[p]	1561	PA120abcpp	1
109	h2o	98	h	1562	PA140abcpp	0
109	h2o	281	adp	1562	PA140abcpp	0
109	h2o	962	pi	1562	PA140abcpp	0
109	h2o	1455	pa140[p]	1562	PA140abcpp	0
135	atp	98	h	1562	PA140abcpp	0
135	atp	281	adp	1562	PA140abcpp	1
135	atp	962	pi	1562	PA140abcpp	0
135	atp	1455	pa140[p]	1562	PA140abcpp	0
1454	pa140	98	h	1562	PA140abcpp	0
1454	pa140	281	adp	1562	PA140abcpp	0
1454	pa140	962	pi	1562	PA140abcpp	0
1454	pa140	1455	pa140[p]	1562	PA140abcpp	1
109	h2o	98	h	1563	PA141abcpp	0
109	h2o	281	adp	1563	PA141abcpp	0
109	h2o	962	pi	1563	PA141abcpp	0
109	h2o	1457	pa141[p]	1563	PA141abcpp	0
135	atp	98	h	1563	PA141abcpp	0
135	atp	281	adp	1563	PA141abcpp	1
135	atp	962	pi	1563	PA141abcpp	0
135	atp	1457	pa141[p]	1563	PA141abcpp	0
1456	pa141	98	h	1563	PA141abcpp	0
1456	pa141	281	adp	1563	PA141abcpp	0
1456	pa141	962	pi	1563	PA141abcpp	0
1456	pa141	1457	pa141[p]	1563	PA141abcpp	1
109	h2o	98	h	1564	PA160abcpp	0
109	h2o	281	adp	1564	PA160abcpp	0
109	h2o	962	pi	1564	PA160abcpp	0
109	h2o	1459	pa160[p]	1564	PA160abcpp	0
135	atp	98	h	1564	PA160abcpp	0
135	atp	281	adp	1564	PA160abcpp	1
135	atp	962	pi	1564	PA160abcpp	0
135	atp	1459	pa160[p]	1564	PA160abcpp	0
1458	pa160	98	h	1564	PA160abcpp	0
1458	pa160	281	adp	1564	PA160abcpp	0
1458	pa160	962	pi	1564	PA160abcpp	0
1458	pa160	1459	pa160[p]	1564	PA160abcpp	1
109	h2o	98	h	1565	PA161abcpp	0
109	h2o	281	adp	1565	PA161abcpp	0
109	h2o	962	pi	1565	PA161abcpp	0
109	h2o	1461	pa161[p]	1565	PA161abcpp	0
135	atp	98	h	1565	PA161abcpp	0
135	atp	281	adp	1565	PA161abcpp	1
135	atp	962	pi	1565	PA161abcpp	0
135	atp	1461	pa161[p]	1565	PA161abcpp	0
1460	pa161	98	h	1565	PA161abcpp	0
1460	pa161	281	adp	1565	PA161abcpp	0
1460	pa161	962	pi	1565	PA161abcpp	0
1460	pa161	1461	pa161[p]	1565	PA161abcpp	1
109	h2o	98	h	1566	PA180abcpp	0
109	h2o	281	adp	1566	PA180abcpp	0
109	h2o	962	pi	1566	PA180abcpp	0
109	h2o	1463	pa180[p]	1566	PA180abcpp	0
135	atp	98	h	1566	PA180abcpp	0
135	atp	281	adp	1566	PA180abcpp	1

135	atp	962	pi	1566	PA180abcpp	0
135	atp	1463	pa180[p]	1566	PA180abcpp	0
1462	pa180	98	h	1566	PA180abcpp	0
1462	pa180	281	adp	1566	PA180abcpp	0
1462	pa180	962	pi	1566	PA180abcpp	0
1462	pa180	1463	pa180[p]	1566	PA180abcpp	1
109	h2o	98	h	1567	PA181abcpp	0
109	h2o	281	adp	1567	PA181abcpp	0
109	h2o	962	pi	1567	PA181abcpp	0
109	h2o	1465	pa181[p]	1567	PA181abcpp	0
135	atp	98	h	1567	PA181abcpp	0
135	atp	281	adp	1567	PA181abcpp	1
135	atp	962	pi	1567	PA181abcpp	0
135	atp	1465	pa181[p]	1567	PA181abcpp	0
1464	pa181	98	h	1567	PA181abcpp	0
1464	pa181	281	adp	1567	PA181abcpp	0
1464	pa181	962	pi	1567	PA181abcpp	0
1464	pa181	1465	pa181[p]	1567	PA181abcpp	1
437	h[p]	98	h	1568	PACALDt2rpp	0
437	h[p]	1561	pacald	1568	PACALDt2rpp	0
1170	pacald[p]	98	h	1568	PACALDt2rpp	0
1170	pacald[p]	1561	pacald	1568	PACALDt2rpp	1
921	pacald[e]	1170	pacald[p]	1569	PACALDtex	1
135	atp	177	amp	1570	PACCOAL	1
135	atp	1192	ppi	1570	PACCOAL	0
135	atp	1607	phaccoa	1570	PACCOAL	0
927	coa	177	amp	1570	PACCOAL	0
927	coa	1192	ppi	1570	PACCOAL	0
927	coa	1607	phaccoa	1570	PACCOAL	1
1640	pac	177	amp	1570	PACCOAL	0
1640	pac	1192	ppi	1570	PACCOAL	0
1640	pac	1607	phaccoa	1570	PACCOAL	1
135	atp	98	h	1571	PANTS	0
135	atp	177	amp	1571	PANTS	1
135	atp	1192	ppi	1571	PANTS	0
135	atp	1490	pnto-R	1571	PANTS	0
922	ala-B	98	h	1571	PANTS	0
922	ala-B	177	amp	1571	PANTS	0
922	ala-B	1192	ppi	1571	PANTS	0
922	ala-B	1490	pnto-R	1571	PANTS	1
1593	pant-R	98	h	1571	PANTS	0
1593	pant-R	177	amp	1571	PANTS	0
1593	pant-R	1192	ppi	1571	PANTS	0
1593	pant-R	1490	pnto-R	1571	PANTS	1
109	h2o	377	12dgr120	1572	PAPA120	0
109	h2o	962	pi	1572	PAPA120	0
1452	pa120	377	12dgr120	1572	PAPA120	1
1452	pa120	962	pi	1572	PAPA120	0
684	h2o[p]	1	12dgr120[p]	1573	PAPA120pp	0
684	h2o[p]	1173	pi[p]	1573	PAPA120pp	0
1453	pa120[p]	1	12dgr120[p]	1573	PAPA120pp	1
1453	pa120[p]	1173	pi[p]	1573	PAPA120pp	0
109	h2o	378	12dgr140	1574	PAPA140	0
109	h2o	962	pi	1574	PAPA140	0
1454	pa140	378	12dgr140	1574	PAPA140	1
1454	pa140	962	pi	1574	PAPA140	0

684	h2o[p]	2	12dgr140[p]	1575	PAPA140pp	0
684	h2o[p]	1173	pi[p]	1575	PAPA140pp	0
1455	pa140[p]	2	12dgr140[p]	1575	PAPA140pp	1
1455	pa140[p]	1173	pi[p]	1575	PAPA140pp	0
109	h2o	379	12dgr141	1576	PAPA141	0
109	h2o	962	pi	1576	PAPA141	0
1456	pa141	379	12dgr141	1576	PAPA141	1
1456	pa141	962	pi	1576	PAPA141	0
684	h2o[p]	3	12dgr141[p]	1577	PAPA141pp	0
684	h2o[p]	1173	pi[p]	1577	PAPA141pp	0
1457	pa141[p]	3	12dgr141[p]	1577	PAPA141pp	1
1457	pa141[p]	1173	pi[p]	1577	PAPA141pp	0
109	h2o	380	12dgr160	1578	PAPA160	0
109	h2o	962	pi	1578	PAPA160	0
1458	pa160	380	12dgr160	1578	PAPA160	1
1458	pa160	962	pi	1578	PAPA160	0
684	h2o[p]	4	12dgr160[p]	1579	PAPA160pp	0
684	h2o[p]	1173	pi[p]	1579	PAPA160pp	0
1459	pa160[p]	4	12dgr160[p]	1579	PAPA160pp	1
1459	pa160[p]	1173	pi[p]	1579	PAPA160pp	0
109	h2o	381	12dgr161	1580	PAPA161	0
109	h2o	962	pi	1580	PAPA161	0
1460	pa161	381	12dgr161	1580	PAPA161	1
1460	pa161	962	pi	1580	PAPA161	0
684	h2o[p]	5	12dgr161[p]	1581	PAPA161pp	0
684	h2o[p]	1173	pi[p]	1581	PAPA161pp	0
1461	pa161[p]	5	12dgr161[p]	1581	PAPA161pp	1
1461	pa161[p]	1173	pi[p]	1581	PAPA161pp	0
109	h2o	382	12dgr180	1582	PAPA180	0
109	h2o	962	pi	1582	PAPA180	0
1462	pa180	382	12dgr180	1582	PAPA180	1
1462	pa180	962	pi	1582	PAPA180	0
684	h2o[p]	6	12dgr180[p]	1583	PAPA180pp	0
684	h2o[p]	1173	pi[p]	1583	PAPA180pp	0
1463	pa180[p]	6	12dgr180[p]	1583	PAPA180pp	1
1463	pa180[p]	1173	pi[p]	1583	PAPA180pp	0
109	h2o	383	12dgr181	1584	PAPA181	0
109	h2o	962	pi	1584	PAPA181	0
1464	pa181	383	12dgr181	1584	PAPA181	1
1464	pa181	962	pi	1584	PAPA181	0
684	h2o[p]	7	12dgr181[p]	1585	PAPA181pp	0
684	h2o[p]	1173	pi[p]	1585	PAPA181pp	0
1465	pa181[p]	7	12dgr181[p]	1585	PAPA181pp	1
1465	pa181[p]	1173	pi[p]	1585	PAPA181pp	0
923	udcpp	1242	ump	1586	PAPPT3	0
923	udcpp	1335	uagmda	1586	PAPPT3	1
1466	ugmda	1242	ump	1586	PAPPT3	1
1466	ugmda	1335	uagmda	1586	PAPPT3	1
924	paps	98	h	1587	PAPSR	0
924	paps	1383	pap	1587	PAPSR	1
924	paps	1590	so3	1587	PAPSR	0
924	paps	1594	trdox	1587	PAPSR	0
981	trdrd	98	h	1587	PAPSR	0
981	trdrd	1383	pap	1587	PAPSR	0
981	trdrd	1590	so3	1587	PAPSR	0
981	trdrd	1594	trdox	1587	PAPSR	1

924	paps	98	h	1588	PAPSR2	0
924	paps	665	grxox	1588	PAPSR2	0
924	paps	1383	pap	1588	PAPSR2	1
924	paps	1590	so3	1588	PAPSR2	0
925	grxrd	98	h	1588	PAPSR2	0
925	grxrd	665	grxox	1588	PAPSR2	1
925	grxrd	1383	pap	1588	PAPSR2	0
925	grxrd	1590	so3	1588	PAPSR2	0
109	h2o	98	h	1589	PDE1	0
109	h2o	177	amp	1589	PDE1	0
926	camp	98	h	1589	PDE1	0
926	camp	177	amp	1589	PDE1	1
856	nad	128	accoa	1590	PDH	0
856	nad	692	co2	1590	PDH	0
856	nad	870	nadh	1590	PDH	1
927	coa	128	accoa	1590	PDH	1
927	coa	692	co2	1590	PDH	0
927	coa	870	nadh	1590	PDH	0
1148	pyr	128	accoa	1590	PDH	1
1148	pyr	692	co2	1590	PDH	0
1148	pyr	870	nadh	1590	PDH	0
856	nad	98	h	1591	PDX5PO2	0
856	nad	870	nadh	1591	PDX5PO2	1
856	nad	1377	pydx5p	1591	PDX5PO2	0
1467	pdx5p	98	h	1591	PDX5PO2	0
1467	pdx5p	870	nadh	1591	PDX5PO2	0
1467	pdx5p	1377	pydx5p	1591	PDX5PO2	1
928	o2	292	h2o2	1592	PDX5POi	0
928	o2	1377	pydx5p	1592	PDX5POi	0
1467	pdx5p	292	h2o2	1592	PDX5POi	0
1467	pdx5p	1377	pydx5p	1592	PDX5POi	1
467	dxyl5p	98	h	1593	PDX5PS	0
467	dxyl5p	109	h2o	1593	PDX5PS	0
467	dxyl5p	692	co2	1593	PDX5PS	0
467	dxyl5p	870	nadh	1593	PDX5PS	0
467	dxyl5p	1467	pdx5p	1593	PDX5PS	1
856	nad	98	h	1593	PDX5PS	0
856	nad	109	h2o	1593	PDX5PS	0
856	nad	692	co2	1593	PDX5PS	0
856	nad	870	nadh	1593	PDX5PS	1
856	nad	1467	pdx5p	1593	PDX5PS	0
1364	phthr	98	h	1593	PDX5PS	0
1364	phthr	109	h2o	1593	PDX5PS	0
1364	phthr	692	co2	1593	PDX5PS	0
1364	phthr	870	nadh	1593	PDX5PS	0
1364	phthr	1467	pdx5p	1593	PDX5PS	1
109	h2o	962	pi	1594	PDXPP	0
109	h2o	1511	pydxn	1594	PDXPP	0
1467	pdx5p	962	pi	1594	PDXPP	0
1467	pdx5p	1511	pydxn	1594	PDXPP	1
109	h2o	98	h	1595	PE120abcpp	0
109	h2o	281	adp	1595	PE120abcpp	0
109	h2o	962	pi	1595	PE120abcpp	0
109	h2o	1486	pe120[p]	1595	PE120abcpp	0
135	atp	98	h	1595	PE120abcpp	0
135	atp	281	adp	1595	PE120abcpp	1

135	atp	962	pi	1595	PE120abcpp	0
135	atp	1486	pe120[p]	1595	PE120abcpp	0
1565	pe120	98	h	1595	PE120abcpp	0
1565	pe120	281	adp	1595	PE120abcpp	0
1565	pe120	962	pi	1595	PE120abcpp	0
1565	pe120	1486	pe120[p]	1595	PE120abcpp	1
109	h2o	98	h	1596	PE140abcpp	0
109	h2o	281	adp	1596	PE140abcpp	0
109	h2o	962	pi	1596	PE140abcpp	0
109	h2o	1487	pe140[p]	1596	PE140abcpp	0
135	atp	98	h	1596	PE140abcpp	0
135	atp	281	adp	1596	PE140abcpp	1
135	atp	962	pi	1596	PE140abcpp	0
135	atp	1487	pe140[p]	1596	PE140abcpp	0
1566	pe140	98	h	1596	PE140abcpp	0
1566	pe140	281	adp	1596	PE140abcpp	0
1566	pe140	962	pi	1596	PE140abcpp	0
1566	pe140	1487	pe140[p]	1596	PE140abcpp	1
109	h2o	98	h	1597	PE141abcpp	0
109	h2o	281	adp	1597	PE141abcpp	0
109	h2o	962	pi	1597	PE141abcpp	0
109	h2o	1488	pe141[p]	1597	PE141abcpp	0
135	atp	98	h	1597	PE141abcpp	0
135	atp	281	adp	1597	PE141abcpp	1
135	atp	962	pi	1597	PE141abcpp	0
135	atp	1488	pe141[p]	1597	PE141abcpp	0
1567	pe141	98	h	1597	PE141abcpp	0
1567	pe141	281	adp	1597	PE141abcpp	0
1567	pe141	962	pi	1597	PE141abcpp	0
1567	pe141	1488	pe141[p]	1597	PE141abcpp	1
109	h2o	98	h	1598	PE160abcpp	0
109	h2o	281	adp	1598	PE160abcpp	0
109	h2o	962	pi	1598	PE160abcpp	0
109	h2o	1378	pe160[p]	1598	PE160abcpp	0
135	atp	98	h	1598	PE160abcpp	0
135	atp	281	adp	1598	PE160abcpp	1
135	atp	962	pi	1598	PE160abcpp	0
135	atp	1378	pe160[p]	1598	PE160abcpp	0
1568	pe160	98	h	1598	PE160abcpp	0
1568	pe160	281	adp	1598	PE160abcpp	0
1568	pe160	962	pi	1598	PE160abcpp	0
1568	pe160	1378	pe160[p]	1598	PE160abcpp	1
109	h2o	98	h	1599	PE161abcpp	0
109	h2o	281	adp	1599	PE161abcpp	0
109	h2o	962	pi	1599	PE161abcpp	0
109	h2o	1468	pe161[p]	1599	PE161abcpp	0
135	atp	98	h	1599	PE161abcpp	0
135	atp	281	adp	1599	PE161abcpp	1
135	atp	962	pi	1599	PE161abcpp	0
135	atp	1468	pe161[p]	1599	PE161abcpp	0
1387	pe161	98	h	1599	PE161abcpp	0
1387	pe161	281	adp	1599	PE161abcpp	0
1387	pe161	962	pi	1599	PE161abcpp	0
1387	pe161	1468	pe161[p]	1599	PE161abcpp	1
109	h2o	98	h	1600	PE180abcpp	0
109	h2o	281	adp	1600	PE180abcpp	0

109	h2o	962	pi	1600	PE180abcpp	0
109	h2o	1489	pe180[p]	1600	PE180abcpp	0
135	atp	98	h	1600	PE180abcpp	0
135	atp	281	adp	1600	PE180abcpp	1
135	atp	962	pi	1600	PE180abcpp	0
135	atp	1489	pe180[p]	1600	PE180abcpp	0
1569	pe180	98	h	1600	PE180abcpp	0
1569	pe180	281	adp	1600	PE180abcpp	0
1569	pe180	962	pi	1600	PE180abcpp	0
1569	pe180	1489	pe180[p]	1600	PE180abcpp	1
109	h2o	98	h	1601	PE181abcpp	0
109	h2o	281	adp	1601	PE181abcpp	0
109	h2o	962	pi	1601	PE181abcpp	0
109	h2o	1469	pe181[p]	1601	PE181abcpp	0
135	atp	98	h	1601	PE181abcpp	0
135	atp	281	adp	1601	PE181abcpp	1
135	atp	962	pi	1601	PE181abcpp	0
135	atp	1469	pe181[p]	1601	PE181abcpp	0
1388	pe181	98	h	1601	PE181abcpp	0
1388	pe181	281	adp	1601	PE181abcpp	0
1388	pe181	962	pi	1601	PE181abcpp	0
1388	pe181	1469	pe181[p]	1601	PE181abcpp	1
684	h2o[p]	859	nh4[p]	1602	PEAMNOpp	0
684	h2o[p]	1122	h2o2[p]	1602	PEAMNOpp	0
684	h2o[p]	1170	pacald[p]	1602	PEAMNOpp	0
903	o2[p]	859	nh4[p]	1602	PEAMNOpp	0
903	o2[p]	1122	h2o2[p]	1602	PEAMNOpp	0
903	o2[p]	1170	pacald[p]	1602	PEAMNOpp	0
1171	peamn[p]	859	nh4[p]	1602	PEAMNOpp	0
1171	peamn[p]	1122	h2o2[p]	1602	PEAMNOpp	0
1171	peamn[p]	1170	pacald[p]	1602	PEAMNOpp	1
929	peamn[e]	1171	peamn[p]	1603	PEAMNtex	1
856	nad	98	h	1604	PERD	0
856	nad	870	nadh	1604	PERD	1
856	nad	1450	ohpb	1604	PERD	0
930	4per	98	h	1604	PERD	0
930	4per	870	nadh	1604	PERD	0
930	4per	1450	ohpb	1604	PERD	1
753	lipa[p]	5	12dgr161[p]	1605	PETNT161pp	0
753	lipa[p]	488	enlipa[p]	1605	PETNT161pp	1
1468	pe161[p]	5	12dgr161[p]	1605	PETNT161pp	1
1468	pe161[p]	488	enlipa[p]	1605	PETNT161pp	1
753	lipa[p]	7	12dgr181[p]	1606	PETNT181pp	0
753	lipa[p]	488	enlipa[p]	1606	PETNT181pp	1
1469	pe181[p]	7	12dgr181[p]	1606	PETNT181pp	1
1469	pe181[p]	488	enlipa[p]	1606	PETNT181pp	1
135	atp	98	h	1607	PFK	0
135	atp	281	adp	1607	PFK	1
135	atp	506	fdp	1607	PFK	0
499	f6p	98	h	1607	PFK	0
499	f6p	281	adp	1607	PFK	0
499	f6p	506	fdp	1607	PFK	1
135	atp	98	h	1608	PFK_2	0
135	atp	281	adp	1608	PFK_2	1
135	atp	1017	tagdp-D	1608	PFK_2	0
1470	tag6p-D	98	h	1608	PFK_2	0

1470	tag6p-D	281	adp	1608	PFK_2	0
1470	tag6p-D	1017	tagdp-D	1608	PFK_2	1
927	coa	128	accoa	1609	PFL	1
927	coa	536	for	1609	PFL	0
1148	pyr	128	accoa	1609	PFL	1
1148	pyr	536	for	1609	PFL	1
109	h2o	98	h	1610	PG120abcpp	0
109	h2o	281	adp	1610	PG120abcpp	0
109	h2o	321	pg120[p]	1610	PG120abcpp	0
109	h2o	962	pi	1610	PG120abcpp	0
135	atp	98	h	1610	PG120abcpp	0
135	atp	281	adp	1610	PG120abcpp	1
135	atp	321	pg120[p]	1610	PG120abcpp	0
135	atp	962	pi	1610	PG120abcpp	0
1336	pg120	98	h	1610	PG120abcpp	0
1336	pg120	281	adp	1610	PG120abcpp	0
1336	pg120	321	pg120[p]	1610	PG120abcpp	1
1336	pg120	962	pi	1610	PG120abcpp	0
109	h2o	98	h	1611	PG140abcpp	0
109	h2o	281	adp	1611	PG140abcpp	0
109	h2o	322	pg140[p]	1611	PG140abcpp	0
109	h2o	962	pi	1611	PG140abcpp	0
135	atp	98	h	1611	PG140abcpp	0
135	atp	281	adp	1611	PG140abcpp	1
135	atp	322	pg140[p]	1611	PG140abcpp	0
135	atp	962	pi	1611	PG140abcpp	0
1337	pg140	98	h	1611	PG140abcpp	0
1337	pg140	281	adp	1611	PG140abcpp	0
1337	pg140	322	pg140[p]	1611	PG140abcpp	1
1337	pg140	962	pi	1611	PG140abcpp	0
109	h2o	98	h	1612	PG141abcpp	0
109	h2o	281	adp	1612	PG141abcpp	0
109	h2o	323	pg141[p]	1612	PG141abcpp	0
109	h2o	962	pi	1612	PG141abcpp	0
135	atp	98	h	1612	PG141abcpp	0
135	atp	281	adp	1612	PG141abcpp	1
135	atp	323	pg141[p]	1612	PG141abcpp	0
135	atp	962	pi	1612	PG141abcpp	0
1338	pg141	98	h	1612	PG141abcpp	0
1338	pg141	281	adp	1612	PG141abcpp	0
1338	pg141	323	pg141[p]	1612	PG141abcpp	1
1338	pg141	962	pi	1612	PG141abcpp	0
109	h2o	98	h	1613	PG160abcpp	0
109	h2o	281	adp	1613	PG160abcpp	0
109	h2o	324	pg160[p]	1613	PG160abcpp	0
109	h2o	962	pi	1613	PG160abcpp	0
135	atp	98	h	1613	PG160abcpp	0
135	atp	281	adp	1613	PG160abcpp	1
135	atp	324	pg160[p]	1613	PG160abcpp	0
135	atp	962	pi	1613	PG160abcpp	0
1339	pg160	98	h	1613	PG160abcpp	0
1339	pg160	281	adp	1613	PG160abcpp	0
1339	pg160	324	pg160[p]	1613	PG160abcpp	1
1339	pg160	962	pi	1613	PG160abcpp	0
109	h2o	98	h	1614	PG161abcpp	0
109	h2o	281	adp	1614	PG161abcpp	0

109	h2o	325	pg161[p]	1614	PG161abcpp	0
109	h2o	962	pi	1614	PG161abcpp	0
135	atp	98	h	1614	PG161abcpp	0
135	atp	281	adp	1614	PG161abcpp	1
135	atp	325	pg161[p]	1614	PG161abcpp	0
135	atp	962	pi	1614	PG161abcpp	0
1340	pg161	98	h	1614	PG161abcpp	0
1340	pg161	281	adp	1614	PG161abcpp	0
1340	pg161	325	pg161[p]	1614	PG161abcpp	1
1340	pg161	962	pi	1614	PG161abcpp	0
109	h2o	98	h	1615	PG180abcpp	0
109	h2o	281	adp	1615	PG180abcpp	0
109	h2o	326	pg180[p]	1615	PG180abcpp	0
109	h2o	962	pi	1615	PG180abcpp	0
135	atp	98	h	1615	PG180abcpp	0
135	atp	281	adp	1615	PG180abcpp	1
135	atp	326	pg180[p]	1615	PG180abcpp	0
135	atp	962	pi	1615	PG180abcpp	0
1341	pg180	98	h	1615	PG180abcpp	0
1341	pg180	281	adp	1615	PG180abcpp	0
1341	pg180	326	pg180[p]	1615	PG180abcpp	1
1341	pg180	962	pi	1615	PG180abcpp	0
109	h2o	98	h	1616	PG181abcpp	0
109	h2o	281	adp	1616	PG181abcpp	0
109	h2o	327	pg181[p]	1616	PG181abcpp	0
109	h2o	962	pi	1616	PG181abcpp	0
135	atp	98	h	1616	PG181abcpp	0
135	atp	281	adp	1616	PG181abcpp	1
135	atp	327	pg181[p]	1616	PG181abcpp	0
135	atp	962	pi	1616	PG181abcpp	0
1342	pg181	98	h	1616	PG181abcpp	0
1342	pg181	281	adp	1616	PG181abcpp	0
1342	pg181	327	pg181[p]	1616	PG181abcpp	1
1342	pg181	962	pi	1616	PG181abcpp	0
931	gam1p	567	gam6p	1617	PGAMT	1
856	nad	98	h	1618	PGCD	0
856	nad	870	nadh	1618	PGCD	1
856	nad	960	3php	1618	PGCD	0
932	3pg	98	h	1618	PGCD	0
932	3pg	870	nadh	1618	PGCD	0
932	3pg	960	3php	1618	PGCD	1
568	g6p	499	f6p	1619	PGI	1
135	atp	281	adp	1620	PGK	1
135	atp	1113	13dpg	1620	PGK	0
932	3pg	281	adp	1620	PGK	0
932	3pg	1113	13dpg	1620	PGK	1
109	h2o	98	h	1621	PGL	0
109	h2o	484	6pgc	1621	PGL	0
933	6pgl	98	h	1621	PGL	0
933	6pgl	484	6pgc	1621	PGL	1
109	h2o	646	glyclt	1622	PGLYCP	0
109	h2o	962	pi	1622	PGLYCP	0
934	2pglyc	646	glyclt	1622	PGLYCP	1
934	2pglyc	962	pi	1622	PGLYCP	0
489	2pg	932	3pg	1623	PGM	1
589	g1p	568	g6p	1624	PGMT	1

109	h2o	98	h	1625	PGP120abcpp	0
109	h2o	281	adp	1625	PGP120abcpp	0
109	h2o	962	pi	1625	PGP120abcpp	0
109	h2o	1472	pgp120[p]	1625	PGP120abcpp	0
135	atp	98	h	1625	PGP120abcpp	0
135	atp	281	adp	1625	PGP120abcpp	1
135	atp	962	pi	1625	PGP120abcpp	0
135	atp	1472	pgp120[p]	1625	PGP120abcpp	0
1471	pgp120	98	h	1625	PGP120abcpp	0
1471	pgp120	281	adp	1625	PGP120abcpp	0
1471	pgp120	962	pi	1625	PGP120abcpp	0
1471	pgp120	1472	pgp120[p]	1625	PGP120abcpp	1
109	h2o	98	h	1626	PGP140abcpp	0
109	h2o	281	adp	1626	PGP140abcpp	0
109	h2o	962	pi	1626	PGP140abcpp	0
109	h2o	1474	pgp140[p]	1626	PGP140abcpp	0
135	atp	98	h	1626	PGP140abcpp	0
135	atp	281	adp	1626	PGP140abcpp	1
135	atp	962	pi	1626	PGP140abcpp	0
135	atp	1474	pgp140[p]	1626	PGP140abcpp	0
1473	pgp140	98	h	1626	PGP140abcpp	0
1473	pgp140	281	adp	1626	PGP140abcpp	0
1473	pgp140	962	pi	1626	PGP140abcpp	0
1473	pgp140	1474	pgp140[p]	1626	PGP140abcpp	1
109	h2o	98	h	1627	PGP141abcpp	0
109	h2o	281	adp	1627	PGP141abcpp	0
109	h2o	962	pi	1627	PGP141abcpp	0
109	h2o	1476	pgp141[p]	1627	PGP141abcpp	0
135	atp	98	h	1627	PGP141abcpp	0
135	atp	281	adp	1627	PGP141abcpp	1
135	atp	962	pi	1627	PGP141abcpp	0
135	atp	1476	pgp141[p]	1627	PGP141abcpp	0
1475	pgp141	98	h	1627	PGP141abcpp	0
1475	pgp141	281	adp	1627	PGP141abcpp	0
1475	pgp141	962	pi	1627	PGP141abcpp	0
1475	pgp141	1476	pgp141[p]	1627	PGP141abcpp	1
109	h2o	98	h	1628	PGP160abcpp	0
109	h2o	281	adp	1628	PGP160abcpp	0
109	h2o	962	pi	1628	PGP160abcpp	0
109	h2o	1478	pgp160[p]	1628	PGP160abcpp	0
135	atp	98	h	1628	PGP160abcpp	0
135	atp	281	adp	1628	PGP160abcpp	1
135	atp	962	pi	1628	PGP160abcpp	0
135	atp	1478	pgp160[p]	1628	PGP160abcpp	0
1477	pgp160	98	h	1628	PGP160abcpp	0
1477	pgp160	281	adp	1628	PGP160abcpp	0
1477	pgp160	962	pi	1628	PGP160abcpp	0
1477	pgp160	1478	pgp160[p]	1628	PGP160abcpp	1
109	h2o	98	h	1629	PGP161abcpp	0
109	h2o	281	adp	1629	PGP161abcpp	0
109	h2o	962	pi	1629	PGP161abcpp	0
109	h2o	1480	pgp161[p]	1629	PGP161abcpp	0
135	atp	98	h	1629	PGP161abcpp	0
135	atp	281	adp	1629	PGP161abcpp	1
135	atp	962	pi	1629	PGP161abcpp	0
135	atp	1480	pgp161[p]	1629	PGP161abcpp	0

1479	pgp161	98	h	1629	PGP161abcpp	0
1479	pgp161	281	adp	1629	PGP161abcpp	0
1479	pgp161	962	pi	1629	PGP161abcpp	0
1479	pgp161	1480	pgp161[p]	1629	PGP161abcpp	1
109	h2o	98	h	1630	PGP180abcpp	0
109	h2o	281	adp	1630	PGP180abcpp	0
109	h2o	962	pi	1630	PGP180abcpp	0
109	h2o	1482	pgp180[p]	1630	PGP180abcpp	0
135	atp	98	h	1630	PGP180abcpp	0
135	atp	281	adp	1630	PGP180abcpp	1
135	atp	962	pi	1630	PGP180abcpp	0
135	atp	1482	pgp180[p]	1630	PGP180abcpp	0
1481	pgp180	98	h	1630	PGP180abcpp	0
1481	pgp180	281	adp	1630	PGP180abcpp	0
1481	pgp180	962	pi	1630	PGP180abcpp	0
1481	pgp180	1482	pgp180[p]	1630	PGP180abcpp	1
109	h2o	98	h	1631	PGP181abcpp	0
109	h2o	281	adp	1631	PGP181abcpp	0
109	h2o	962	pi	1631	PGP181abcpp	0
109	h2o	1484	pgp181[p]	1631	PGP181abcpp	0
135	atp	98	h	1631	PGP181abcpp	0
135	atp	281	adp	1631	PGP181abcpp	1
135	atp	962	pi	1631	PGP181abcpp	0
135	atp	1484	pgp181[p]	1631	PGP181abcpp	0
1483	pgp181	98	h	1631	PGP181abcpp	0
1483	pgp181	281	adp	1631	PGP181abcpp	0
1483	pgp181	962	pi	1631	PGP181abcpp	0
1483	pgp181	1484	pgp181[p]	1631	PGP181abcpp	1
109	h2o	962	pi	1632	PGPP120	0
109	h2o	1336	pg120	1632	PGPP120	0
1471	pgp120	962	pi	1632	PGPP120	0
1471	pgp120	1336	pg120	1632	PGPP120	1
684	h2o[p]	321	pg120[p]	1633	PGPP120pp	0
684	h2o[p]	1173	pi[p]	1633	PGPP120pp	0
1472	pgp120[p]	321	pg120[p]	1633	PGPP120pp	1
1472	pgp120[p]	1173	pi[p]	1633	PGPP120pp	0
109	h2o	962	pi	1634	PGPP140	0
109	h2o	1337	pg140	1634	PGPP140	0
1473	pgp140	962	pi	1634	PGPP140	0
1473	pgp140	1337	pg140	1634	PGPP140	1
684	h2o[p]	322	pg140[p]	1635	PGPP140pp	0
684	h2o[p]	1173	pi[p]	1635	PGPP140pp	0
1474	pgp140[p]	322	pg140[p]	1635	PGPP140pp	1
1474	pgp140[p]	1173	pi[p]	1635	PGPP140pp	0
109	h2o	962	pi	1636	PGPP141	0
109	h2o	1338	pg141	1636	PGPP141	0
1475	pgp141	962	pi	1636	PGPP141	0
1475	pgp141	1338	pg141	1636	PGPP141	1
684	h2o[p]	323	pg141[p]	1637	PGPP141pp	0
684	h2o[p]	1173	pi[p]	1637	PGPP141pp	0
1476	pgp141[p]	323	pg141[p]	1637	PGPP141pp	1
1476	pgp141[p]	1173	pi[p]	1637	PGPP141pp	0
109	h2o	962	pi	1638	PGPP160	0
109	h2o	1339	pg160	1638	PGPP160	0
1477	pgp160	962	pi	1638	PGPP160	0
1477	pgp160	1339	pg160	1638	PGPP160	1

684	h2o[p]	324	pg160[p]	1639	PGPP160pp	0
684	h2o[p]	1173	pi[p]	1639	PGPP160pp	0
1478	pgp160[p]	324	pg160[p]	1639	PGPP160pp	1
1478	pgp160[p]	1173	pi[p]	1639	PGPP160pp	0
109	h2o	962	pi	1640	PGPP161	0
109	h2o	1340	pg161	1640	PGPP161	0
1479	pgp161	962	pi	1640	PGPP161	0
1479	pgp161	1340	pg161	1640	PGPP161	1
684	h2o[p]	325	pg161[p]	1641	PGPP161pp	0
684	h2o[p]	1173	pi[p]	1641	PGPP161pp	0
1480	pgp161[p]	325	pg161[p]	1641	PGPP161pp	1
1480	pgp161[p]	1173	pi[p]	1641	PGPP161pp	0
109	h2o	962	pi	1642	PGPP180	0
109	h2o	1341	pg180	1642	PGPP180	0
1481	pgp180	962	pi	1642	PGPP180	0
1481	pgp180	1341	pg180	1642	PGPP180	1
684	h2o[p]	326	pg180[p]	1643	PGPP180pp	0
684	h2o[p]	1173	pi[p]	1643	PGPP180pp	0
1482	pgp180[p]	326	pg180[p]	1643	PGPP180pp	1
1482	pgp180[p]	1173	pi[p]	1643	PGPP180pp	0
109	h2o	962	pi	1644	PGPP181	0
109	h2o	1342	pg181	1644	PGPP181	0
1483	pgp181	962	pi	1644	PGPP181	0
1483	pgp181	1342	pg181	1644	PGPP181	1
684	h2o[p]	327	pg181[p]	1645	PGPP181pp	0
684	h2o[p]	1173	pi[p]	1645	PGPP181pp	0
1484	pgp181[p]	327	pg181[p]	1645	PGPP181pp	1
1484	pgp181[p]	1173	pi[p]	1645	PGPP181pp	0
298	cdpdddecg	98	h	1646	PGSA120	0
298	cdpdddecg	330	cmp	1646	PGSA120	1
298	cdpdddecg	1471	pgp120	1646	PGSA120	1
558	glyc3p	98	h	1646	PGSA120	0
558	glyc3p	330	cmp	1646	PGSA120	1
558	glyc3p	1471	pgp120	1646	PGSA120	0
299	cdpdtdecg	98	h	1647	PGSA140	0
299	cdpdtdecg	330	cmp	1647	PGSA140	1
299	cdpdtdecg	1473	pgp140	1647	PGSA140	1
558	glyc3p	98	h	1647	PGSA140	0
558	glyc3p	330	cmp	1647	PGSA140	1
558	glyc3p	1473	pgp140	1647	PGSA140	0
300	cdpdtdec7eg	98	h	1648	PGSA141	0
300	cdpdtdec7eg	330	cmp	1648	PGSA141	1
300	cdpdtdec7eg	1475	pgp141	1648	PGSA141	1
558	glyc3p	98	h	1648	PGSA141	0
558	glyc3p	330	cmp	1648	PGSA141	1
558	glyc3p	1475	pgp141	1648	PGSA141	0
301	cdpdhdecg	98	h	1649	PGSA160	0
301	cdpdhdecg	330	cmp	1649	PGSA160	1
301	cdpdhdecg	1477	pgp160	1649	PGSA160	1
558	glyc3p	98	h	1649	PGSA160	0
558	glyc3p	330	cmp	1649	PGSA160	1
558	glyc3p	1477	pgp160	1649	PGSA160	0
302	cdpdhdec9eg	98	h	1650	PGSA161	0
302	cdpdhdec9eg	330	cmp	1650	PGSA161	1
302	cdpdhdec9eg	1479	pgp161	1650	PGSA161	1
558	glyc3p	98	h	1650	PGSA161	0

558	glyc3p	330	cmp	1650	PGSA161	1
558	glyc3p	1479	pgp161	1650	PGSA161	0
303	cdpdodecg	98	h	1651	PGSA180	0
303	cdpdodecg	330	cmp	1651	PGSA180	1
303	cdpdodecg	1481	pgp180	1651	PGSA180	1
558	glyc3p	98	h	1651	PGSA180	0
558	glyc3p	330	cmp	1651	PGSA180	1
558	glyc3p	1481	pgp180	1651	PGSA180	0
304	cdpdodec11eg	98	h	1652	PGSA181	0
304	cdpdodec11eg	330	cmp	1652	PGSA181	1
304	cdpdodec11eg	1483	pgp181	1652	PGSA181	1
558	glyc3p	98	h	1652	PGSA181	0
558	glyc3p	330	cmp	1652	PGSA181	1
558	glyc3p	1483	pgp181	1652	PGSA181	0
109	h2o	98	h	1653	PHEMEabcpp	0
109	h2o	281	adp	1653	PHEMEabcpp	0
109	h2o	935	pheme[p]	1653	PHEMEabcpp	0
109	h2o	962	pi	1653	PHEMEabcpp	0
135	atp	98	h	1653	PHEMEabcpp	0
135	atp	281	adp	1653	PHEMEabcpp	1
135	atp	935	pheme[p]	1653	PHEMEabcpp	0
135	atp	962	pi	1653	PHEMEabcpp	0
1597	pheme	98	h	1653	PHEMEabcpp	0
1597	pheme	281	adp	1653	PHEMEabcpp	0
1597	pheme	935	pheme[p]	1653	PHEMEabcpp	1
1597	pheme	962	pi	1653	PHEMEabcpp	0
935	pheme[p]	1343	pheme[e]	1654	PHEMEtiex	1
437	h[p]	98	h	1655	PHEt2rpp	0
437	h[p]	1485	phe-L	1655	PHEt2rpp	0
1172	phe-L[p]	98	h	1655	PHEt2rpp	0
1172	phe-L[p]	1485	phe-L	1655	PHEt2rpp	1
213	akg	624	glu-L	1656	PHETA1	1
213	akg	1562	phpyr	1656	PHETA1	0
1485	phe-L	624	glu-L	1656	PHETA1	0
1485	phe-L	1562	phpyr	1656	PHETA1	1
936	phe-L[e]	1172	phe-L[p]	1657	PHEtex	1
135	atp	177	amp	1658	PHETRS	1
135	atp	1192	ppi	1658	PHETRS	0
135	atp	1608	phetrna	1658	PHETRS	0
1485	phe-L	177	amp	1658	PHETRS	0
1485	phe-L	1192	ppi	1658	PHETRS	0
1485	phe-L	1608	phetrna	1658	PHETRS	1
1641	trnaphe	177	amp	1658	PHETRS	0
1641	trnaphe	1192	ppi	1658	PHETRS	0
1641	trnaphe	1608	phetrna	1658	PHETRS	1
684	h2o[p]	723	inost[p]	1659	PHYTSpp	0
684	h2o[p]	1173	pi[p]	1659	PHYTSpp	0
1322	minohp[p]	723	inost[p]	1659	PHYTSpp	1
1322	minohp[p]	1173	pi[p]	1659	PHYTSpp	0
437	h[p]	98	h	1660	PIt2rpp	0
437	h[p]	962	pi	1660	PIt2rpp	0
1173	pi[p]	98	h	1660	PIt2rpp	0
1173	pi[p]	962	pi	1660	PIt2rpp	0
937	pi[e]	1173	pi[p]	1661	PItex	0
109	h2o	98	h	1662	PIuabcpp	0
109	h2o	281	adp	1662	PIuabcpp	0

109	h2o	962	pi	1662	PIuabcpp	0
135	atp	98	h	1662	PIuabcpp	0
135	atp	281	adp	1662	PIuabcpp	1
135	atp	962	pi	1662	PIuabcpp	0
1173	pi[p]	98	h	1662	PIuabcpp	0
1173	pi[p]	281	adp	1662	PIuabcpp	0
1173	pi[p]	962	pi	1662	PIuabcpp	0
684	h2o[p]	25	2ddecg3p[p]	1663	PLIPA1A120pp	0
684	h2o[p]	1261	ddca[p]	1663	PLIPA1A120pp	0
1453	pa120[p]	25	2ddecg3p[p]	1663	PLIPA1A120pp	1
1453	pa120[p]	1261	ddca[p]	1663	PLIPA1A120pp	1
684	h2o[p]	26	2tdecg3p[p]	1664	PLIPA1A140pp	0
684	h2o[p]	1356	ttdca[p]	1664	PLIPA1A140pp	0
1455	pa140[p]	26	2tdecg3p[p]	1664	PLIPA1A140pp	1
1455	pa140[p]	1356	ttdca[p]	1664	PLIPA1A140pp	1
684	h2o[p]	27	2tdec7eg3p[p]	1665	PLIPA1A141pp	0
684	h2o[p]	1357	ttdcea[p]	1665	PLIPA1A141pp	0
1457	pa141[p]	27	2tdec7eg3p[p]	1665	PLIPA1A141pp	1
1457	pa141[p]	1357	ttdcea[p]	1665	PLIPA1A141pp	1
684	h2o[p]	28	2hdecg3p[p]	1666	PLIPA1A160pp	0
684	h2o[p]	1289	hdca[p]	1666	PLIPA1A160pp	0
1459	pa160[p]	28	2hdecg3p[p]	1666	PLIPA1A160pp	1
1459	pa160[p]	1289	hdca[p]	1666	PLIPA1A160pp	1
684	h2o[p]	29	2hdec9eg3p[p]	1667	PLIPA1A161pp	0
684	h2o[p]	1290	hdcea[p]	1667	PLIPA1A161pp	0
1461	pa161[p]	29	2hdec9eg3p[p]	1667	PLIPA1A161pp	1
1461	pa161[p]	1290	hdcea[p]	1667	PLIPA1A161pp	1
684	h2o[p]	30	2odecg3p[p]	1668	PLIPA1A180pp	0
684	h2o[p]	1331	ocdca[p]	1668	PLIPA1A180pp	0
1463	pa180[p]	30	2odecg3p[p]	1668	PLIPA1A180pp	1
1463	pa180[p]	1331	ocdca[p]	1668	PLIPA1A180pp	1
684	h2o[p]	31	2odec11eg3p[p]	1669	PLIPA1A181pp	0
684	h2o[p]	1332	ocdcea[p]	1669	PLIPA1A181pp	0
1465	pa181[p]	31	2odec11eg3p[p]	1669	PLIPA1A181pp	1
1465	pa181[p]	1332	ocdcea[p]	1669	PLIPA1A181pp	1
684	h2o[p]	32	2agpe120[p]	1670	PLIPA1E120pp	0
684	h2o[p]	437	h[p]	1670	PLIPA1E120pp	0
684	h2o[p]	1261	ddca[p]	1670	PLIPA1E120pp	0
1486	pe120[p]	32	2agpe120[p]	1670	PLIPA1E120pp	1
1486	pe120[p]	437	h[p]	1670	PLIPA1E120pp	0
1486	pe120[p]	1261	ddca[p]	1670	PLIPA1E120pp	1
684	h2o[p]	33	2agpe140[p]	1671	PLIPA1E140pp	0
684	h2o[p]	437	h[p]	1671	PLIPA1E140pp	0
684	h2o[p]	1356	ttdca[p]	1671	PLIPA1E140pp	0
1487	pe140[p]	33	2agpe140[p]	1671	PLIPA1E140pp	1
1487	pe140[p]	437	h[p]	1671	PLIPA1E140pp	0
1487	pe140[p]	1356	ttdca[p]	1671	PLIPA1E140pp	1
684	h2o[p]	34	2agpe141[p]	1672	PLIPA1E141pp	0
684	h2o[p]	437	h[p]	1672	PLIPA1E141pp	0
684	h2o[p]	1357	ttdcea[p]	1672	PLIPA1E141pp	0
1488	pe141[p]	34	2agpe141[p]	1672	PLIPA1E141pp	1
1488	pe141[p]	437	h[p]	1672	PLIPA1E141pp	0
1488	pe141[p]	1357	ttdcea[p]	1672	PLIPA1E141pp	1
684	h2o[p]	35	2agpe160[p]	1673	PLIPA1E160pp	0
684	h2o[p]	437	h[p]	1673	PLIPA1E160pp	0
684	h2o[p]	1289	hdca[p]	1673	PLIPA1E160pp	0

1378	pe160[p]	35	2agpe160[p]	1673	PLIPA1E160pp	1
1378	pe160[p]	437	h[p]	1673	PLIPA1E160pp	0
1378	pe160[p]	1289	hdca[p]	1673	PLIPA1E160pp	1
684	h2o[p]	36	2agpe161[p]	1674	PLIPA1E161pp	0
684	h2o[p]	437	h[p]	1674	PLIPA1E161pp	0
684	h2o[p]	1290	hdcea[p]	1674	PLIPA1E161pp	0
1468	pe161[p]	36	2agpe161[p]	1674	PLIPA1E161pp	1
1468	pe161[p]	437	h[p]	1674	PLIPA1E161pp	0
1468	pe161[p]	1290	hdcea[p]	1674	PLIPA1E161pp	1
684	h2o[p]	37	2agpe180[p]	1675	PLIPA1E180pp	0
684	h2o[p]	437	h[p]	1675	PLIPA1E180pp	0
684	h2o[p]	1331	ocdca[p]	1675	PLIPA1E180pp	0
1489	pe180[p]	37	2agpe180[p]	1675	PLIPA1E180pp	1
1489	pe180[p]	437	h[p]	1675	PLIPA1E180pp	0
1489	pe180[p]	1331	ocdca[p]	1675	PLIPA1E180pp	1
684	h2o[p]	38	2agpe181[p]	1676	PLIPA1E181pp	0
684	h2o[p]	437	h[p]	1676	PLIPA1E181pp	0
684	h2o[p]	1332	ocdcea[p]	1676	PLIPA1E181pp	0
1469	pe181[p]	38	2agpe181[p]	1676	PLIPA1E181pp	1
1469	pe181[p]	437	h[p]	1676	PLIPA1E181pp	0
1469	pe181[p]	1332	ocdcea[p]	1676	PLIPA1E181pp	1
321	pg120[p]	46	2agpg120[p]	1677	PLIPA1G120pp	1
321	pg120[p]	437	h[p]	1677	PLIPA1G120pp	0
321	pg120[p]	1261	ddca[p]	1677	PLIPA1G120pp	1
684	h2o[p]	46	2agpg120[p]	1677	PLIPA1G120pp	0
684	h2o[p]	437	h[p]	1677	PLIPA1G120pp	0
684	h2o[p]	1261	ddca[p]	1677	PLIPA1G120pp	0
322	pg140[p]	47	2agpg140[p]	1678	PLIPA1G140pp	1
322	pg140[p]	437	h[p]	1678	PLIPA1G140pp	0
322	pg140[p]	1356	ttdca[p]	1678	PLIPA1G140pp	1
684	h2o[p]	47	2agpg140[p]	1678	PLIPA1G140pp	0
684	h2o[p]	437	h[p]	1678	PLIPA1G140pp	0
684	h2o[p]	1356	ttdca[p]	1678	PLIPA1G140pp	0
323	pg141[p]	48	2agpg141[p]	1679	PLIPA1G141pp	1
323	pg141[p]	437	h[p]	1679	PLIPA1G141pp	0
323	pg141[p]	1357	ttdcea[p]	1679	PLIPA1G141pp	1
684	h2o[p]	48	2agpg141[p]	1679	PLIPA1G141pp	0
684	h2o[p]	437	h[p]	1679	PLIPA1G141pp	0
684	h2o[p]	1357	ttdcea[p]	1679	PLIPA1G141pp	0
324	pg160[p]	49	2agpg160[p]	1680	PLIPA1G160pp	1
324	pg160[p]	437	h[p]	1680	PLIPA1G160pp	0
324	pg160[p]	1289	hdca[p]	1680	PLIPA1G160pp	1
684	h2o[p]	49	2agpg160[p]	1680	PLIPA1G160pp	0
684	h2o[p]	437	h[p]	1680	PLIPA1G160pp	0
684	h2o[p]	1289	hdca[p]	1680	PLIPA1G160pp	0
325	pg161[p]	50	2agpg161[p]	1681	PLIPA1G161pp	1
325	pg161[p]	437	h[p]	1681	PLIPA1G161pp	0
325	pg161[p]	1290	hdcea[p]	1681	PLIPA1G161pp	1
684	h2o[p]	50	2agpg161[p]	1681	PLIPA1G161pp	0
684	h2o[p]	437	h[p]	1681	PLIPA1G161pp	0
684	h2o[p]	1290	hdcea[p]	1681	PLIPA1G161pp	0
326	pg180[p]	51	2agpg180[p]	1682	PLIPA1G180pp	1
326	pg180[p]	437	h[p]	1682	PLIPA1G180pp	0
326	pg180[p]	1331	ocdca[p]	1682	PLIPA1G180pp	1
684	h2o[p]	51	2agpg180[p]	1682	PLIPA1G180pp	0
684	h2o[p]	437	h[p]	1682	PLIPA1G180pp	0

684	h2o[p]	1331	ocdca[p]	1682	PLIPA1G180pp	0
327	pg181[p]	52	2agpg181[p]	1683	PLIPA1G181pp	1
327	pg181[p]	437	h[p]	1683	PLIPA1G181pp	0
327	pg181[p]	1332	ocdcea[p]	1683	PLIPA1G181pp	1
684	h2o[p]	52	2agpg181[p]	1683	PLIPA1G181pp	0
684	h2o[p]	437	h[p]	1683	PLIPA1G181pp	0
684	h2o[p]	1332	ocdcea[p]	1683	PLIPA1G181pp	0
684	h2o[p]	437	h[p]	1684	PLIPA2A120pp	0
684	h2o[p]	757	1ddecg3p[p]	1684	PLIPA2A120pp	0
684	h2o[p]	1261	ddca[p]	1684	PLIPA2A120pp	0
1453	pa120[p]	437	h[p]	1684	PLIPA2A120pp	0
1453	pa120[p]	757	1ddecg3p[p]	1684	PLIPA2A120pp	1
1453	pa120[p]	1261	ddca[p]	1684	PLIPA2A120pp	1
684	h2o[p]	437	h[p]	1685	PLIPA2A140pp	0
684	h2o[p]	758	1tdecg3p[p]	1685	PLIPA2A140pp	0
684	h2o[p]	1356	ttdca[p]	1685	PLIPA2A140pp	0
1455	pa140[p]	437	h[p]	1685	PLIPA2A140pp	0
1455	pa140[p]	758	1tdecg3p[p]	1685	PLIPA2A140pp	1
1455	pa140[p]	1356	ttdca[p]	1685	PLIPA2A140pp	1
684	h2o[p]	437	h[p]	1686	PLIPA2A141pp	0
684	h2o[p]	759	1tdec7eg3p[p]	1686	PLIPA2A141pp	0
684	h2o[p]	1357	ttdcea[p]	1686	PLIPA2A141pp	0
1457	pa141[p]	437	h[p]	1686	PLIPA2A141pp	0
1457	pa141[p]	759	1tdec7eg3p[p]	1686	PLIPA2A141pp	1
1457	pa141[p]	1357	ttdcea[p]	1686	PLIPA2A141pp	1
684	h2o[p]	437	h[p]	1687	PLIPA2A160pp	0
684	h2o[p]	760	1hdecg3p[p]	1687	PLIPA2A160pp	0
684	h2o[p]	1289	hdca[p]	1687	PLIPA2A160pp	0
1459	pa160[p]	437	h[p]	1687	PLIPA2A160pp	0
1459	pa160[p]	760	1hdecg3p[p]	1687	PLIPA2A160pp	1
1459	pa160[p]	1289	hdca[p]	1687	PLIPA2A160pp	1
684	h2o[p]	437	h[p]	1688	PLIPA2A161pp	0
684	h2o[p]	761	1hdec9eg3p[p]	1688	PLIPA2A161pp	0
684	h2o[p]	1290	hdcea[p]	1688	PLIPA2A161pp	0
1461	pa161[p]	437	h[p]	1688	PLIPA2A161pp	0
1461	pa161[p]	761	1hdec9eg3p[p]	1688	PLIPA2A161pp	1
1461	pa161[p]	1290	hdcea[p]	1688	PLIPA2A161pp	1
684	h2o[p]	437	h[p]	1689	PLIPA2A180pp	0
684	h2o[p]	762	1odecg3p[p]	1689	PLIPA2A180pp	0
684	h2o[p]	1331	ocdca[p]	1689	PLIPA2A180pp	0
1463	pa180[p]	437	h[p]	1689	PLIPA2A180pp	0
1463	pa180[p]	762	1odecg3p[p]	1689	PLIPA2A180pp	1
1463	pa180[p]	1331	ocdca[p]	1689	PLIPA2A180pp	1
684	h2o[p]	437	h[p]	1690	PLIPA2A181pp	0
684	h2o[p]	763	1odec11eg3p[p]	1690	PLIPA2A181pp	0
684	h2o[p]	1332	ocdcea[p]	1690	PLIPA2A181pp	0
1465	pa181[p]	437	h[p]	1690	PLIPA2A181pp	0
1465	pa181[p]	763	1odec11eg3p[p]	1690	PLIPA2A181pp	1
1465	pa181[p]	1332	ocdcea[p]	1690	PLIPA2A181pp	1
684	h2o[p]	437	h[p]	1691	PLIPA2E120pp	0
684	h2o[p]	764	1agpe120[p]	1691	PLIPA2E120pp	0
684	h2o[p]	1261	ddca[p]	1691	PLIPA2E120pp	0
1486	pe120[p]	437	h[p]	1691	PLIPA2E120pp	0
1486	pe120[p]	764	1agpe120[p]	1691	PLIPA2E120pp	1
1486	pe120[p]	1261	ddca[p]	1691	PLIPA2E120pp	1
684	h2o[p]	437	h[p]	1692	PLIPA2E140pp	0

684	h2o[p]	765	1agpe140[p]	1692	PLIPA2E140pp	0
684	h2o[p]	1356	ttdca[p]	1692	PLIPA2E140pp	0
1487	pe140[p]	437	h[p]	1692	PLIPA2E140pp	0
1487	pe140[p]	765	1agpe140[p]	1692	PLIPA2E140pp	1
1487	pe140[p]	1356	ttdca[p]	1692	PLIPA2E140pp	1
684	h2o[p]	437	h[p]	1693	PLIPA2E141pp	0
684	h2o[p]	766	1agpe141[p]	1693	PLIPA2E141pp	0
684	h2o[p]	1357	ttdcea[p]	1693	PLIPA2E141pp	0
1488	pe141[p]	437	h[p]	1693	PLIPA2E141pp	0
1488	pe141[p]	766	1agpe141[p]	1693	PLIPA2E141pp	1
1488	pe141[p]	1357	ttdcea[p]	1693	PLIPA2E141pp	1
684	h2o[p]	437	h[p]	1694	PLIPA2E160pp	0
684	h2o[p]	767	1agpe160[p]	1694	PLIPA2E160pp	0
684	h2o[p]	1289	hdca[p]	1694	PLIPA2E160pp	0
1378	pe160[p]	437	h[p]	1694	PLIPA2E160pp	0
1378	pe160[p]	767	1agpe160[p]	1694	PLIPA2E160pp	1
1378	pe160[p]	1289	hdca[p]	1694	PLIPA2E160pp	1
684	h2o[p]	437	h[p]	1695	PLIPA2E161pp	0
684	h2o[p]	768	1agpe161[p]	1695	PLIPA2E161pp	0
684	h2o[p]	1290	hdcea[p]	1695	PLIPA2E161pp	0
1468	pe161[p]	437	h[p]	1695	PLIPA2E161pp	0
1468	pe161[p]	768	1agpe161[p]	1695	PLIPA2E161pp	1
1468	pe161[p]	1290	hdcea[p]	1695	PLIPA2E161pp	1
684	h2o[p]	437	h[p]	1696	PLIPA2E180pp	0
684	h2o[p]	769	1agpe180[p]	1696	PLIPA2E180pp	0
684	h2o[p]	1331	ocdca[p]	1696	PLIPA2E180pp	0
1489	pe180[p]	437	h[p]	1696	PLIPA2E180pp	0
1489	pe180[p]	769	1agpe180[p]	1696	PLIPA2E180pp	1
1489	pe180[p]	1331	ocdca[p]	1696	PLIPA2E180pp	1
684	h2o[p]	437	h[p]	1697	PLIPA2E181pp	0
684	h2o[p]	770	1agpe181[p]	1697	PLIPA2E181pp	0
684	h2o[p]	1332	ocdcea[p]	1697	PLIPA2E181pp	0
1469	pe181[p]	437	h[p]	1697	PLIPA2E181pp	0
1469	pe181[p]	770	1agpe181[p]	1697	PLIPA2E181pp	1
1469	pe181[p]	1332	ocdcea[p]	1697	PLIPA2E181pp	1
321	pg120[p]	437	h[p]	1698	PLIPA2G120pp	0
321	pg120[p]	771	1agpg120[p]	1698	PLIPA2G120pp	1
321	pg120[p]	1261	ddca[p]	1698	PLIPA2G120pp	1
684	h2o[p]	437	h[p]	1698	PLIPA2G120pp	0
684	h2o[p]	771	1agpg120[p]	1698	PLIPA2G120pp	0
684	h2o[p]	1261	ddca[p]	1698	PLIPA2G120pp	0
322	pg140[p]	437	h[p]	1699	PLIPA2G140pp	0
322	pg140[p]	772	1agpg140[p]	1699	PLIPA2G140pp	1
322	pg140[p]	1356	ttdca[p]	1699	PLIPA2G140pp	1
684	h2o[p]	437	h[p]	1699	PLIPA2G140pp	0
684	h2o[p]	772	1agpg140[p]	1699	PLIPA2G140pp	0
684	h2o[p]	1356	ttdca[p]	1699	PLIPA2G140pp	0
323	pg141[p]	437	h[p]	1700	PLIPA2G141pp	0
323	pg141[p]	773	1agpg141[p]	1700	PLIPA2G141pp	1
323	pg141[p]	1357	ttdcea[p]	1700	PLIPA2G141pp	1
684	h2o[p]	437	h[p]	1700	PLIPA2G141pp	0
684	h2o[p]	773	1agpg141[p]	1700	PLIPA2G141pp	0
684	h2o[p]	1357	ttdcea[p]	1700	PLIPA2G141pp	0
324	pg160[p]	437	h[p]	1701	PLIPA2G160pp	0
324	pg160[p]	774	1agpg160[p]	1701	PLIPA2G160pp	1
324	pg160[p]	1289	hdca[p]	1701	PLIPA2G160pp	1

684	h2o[p]	437	h[p]	1701	PLIPA2G160pp	0
684	h2o[p]	774	1agpg160[p]	1701	PLIPA2G160pp	0
684	h2o[p]	1289	hdca[p]	1701	PLIPA2G160pp	0
325	pg161[p]	437	h[p]	1702	PLIPA2G161pp	0
325	pg161[p]	775	1agpg161[p]	1702	PLIPA2G161pp	1
325	pg161[p]	1290	hdcea[p]	1702	PLIPA2G161pp	1
684	h2o[p]	437	h[p]	1702	PLIPA2G161pp	0
684	h2o[p]	775	1agpg161[p]	1702	PLIPA2G161pp	0
684	h2o[p]	1290	hdcea[p]	1702	PLIPA2G161pp	0
326	pg180[p]	437	h[p]	1703	PLIPA2G180pp	0
326	pg180[p]	776	1agpg180[p]	1703	PLIPA2G180pp	1
326	pg180[p]	1331	ocdca[p]	1703	PLIPA2G180pp	1
684	h2o[p]	437	h[p]	1703	PLIPA2G180pp	0
684	h2o[p]	776	1agpg180[p]	1703	PLIPA2G180pp	0
684	h2o[p]	1331	ocdca[p]	1703	PLIPA2G180pp	0
327	pg181[p]	437	h[p]	1704	PLIPA2G181pp	0
327	pg181[p]	777	1agpg181[p]	1704	PLIPA2G181pp	1
327	pg181[p]	1332	ocdcea[p]	1704	PLIPA2G181pp	1
684	h2o[p]	437	h[p]	1704	PLIPA2G181pp	0
684	h2o[p]	777	1agpg181[p]	1704	PLIPA2G181pp	0
684	h2o[p]	1332	ocdcea[p]	1704	PLIPA2G181pp	0
938	man1p	799	man6p	1705	PMANM	1
109	h2o	962	pi	1706	PMDPHT	0
109	h2o	971	4r5au	1706	PMDPHT	0
939	5aprbu	962	pi	1706	PMDPHT	0
939	5aprbu	971	4r5au	1706	PMDPHT	1
135	atp	62	2mahmp	1707	PMPK	0
135	atp	281	adp	1707	PMPK	1
940	4ampm	62	2mahmp	1707	PMPK	1
940	4ampm	281	adp	1707	PMPK	0
135	atp	98	h	1708	PNTK	0
135	atp	281	adp	1708	PNTK	1
135	atp	951	4ppan	1708	PNTK	0
1490	pnto-R	98	h	1708	PNTK	0
1490	pnto-R	281	adp	1708	PNTK	0
1490	pnto-R	951	4ppan	1708	PNTK	1
941	na1[p]	1344	na1	1709	PNTOt4pp	0
941	na1[p]	1490	pnto-R	1709	PNTOt4pp	0
1174	pnto-R[p]	1344	na1	1709	PNTOt4pp	0
1174	pnto-R[p]	1490	pnto-R	1709	PNTOt4pp	1
942	pnto-R[e]	1174	pnto-R[p]	1710	PNTOtex	1
109	h2o	147	ac	1711	POX	0
109	h2o	692	co2	1711	POX	0
109	h2o	1443	q8h2	1711	POX	0
1003	q8	147	ac	1711	POX	0
1003	q8	692	co2	1711	POX	0
1003	q8	1443	q8h2	1711	POX	1
1148	pyr	147	ac	1711	POX	1
1148	pyr	692	co2	1711	POX	0
1148	pyr	1443	q8h2	1711	POX	0
109	h2o	98	h	1712	PPA	0
109	h2o	962	pi	1712	PPA	0
1192	ppi	98	h	1712	PPA	0
1192	ppi	962	pi	1712	PPA	0
109	h2o	98	h	1713	PPA2	0
109	h2o	962	pi	1713	PPA2	0

109	h2o	1192	ppi	1713	PPA2	0
1491	pppi	98	h	1713	PPA2	0
1491	pppi	962	pi	1713	PPA2	0
1491	pppi	1192	ppi	1713	PPA2	0
281	adp	135	atp	1714	PPAKr	1
281	adp	1345	ppa	1714	PPAKr	0
1492	ppap	135	atp	1714	PPAKr	0
1492	ppap	1345	ppa	1714	PPAKr	1
943	ppal[e]	1175	ppal[p]	1715	PPALtex	1
944	ppal	1175	ppal[p]	1716	PPALtpp	1
941	na1[p]	1344	na1	1717	PPAt4pp	0
941	na1[p]	1345	ppa	1717	PPAt4pp	0
1176	ppa[p]	1344	na1	1717	PPAt4pp	0
1176	ppa[p]	1345	ppa	1717	PPAt4pp	0
945	ppa[e]	1176	ppa[p]	1718	PPAtex	0
946	5aop	98	h	1719	PPBNGS	0
946	5aop	109	h2o	1719	PPBNGS	0
946	5aop	1416	ppbng	1719	PPBNGS	1
109	h2o	98	h	1720	PPC	0
109	h2o	962	pi	1720	PPC	0
109	h2o	1318	oaa	1720	PPC	0
692	co2	98	h	1720	PPC	0
692	co2	962	pi	1720	PPC	0
692	co2	1318	oaa	1720	PPC	0
959	pep	98	h	1720	PPC	0
959	pep	962	pi	1720	PPC	0
959	pep	1318	oaa	1720	PPC	1
98	h	692	co2	1721	PPCDC	0
98	h	1609	pan4p	1721	PPCDC	0
947	4ppcys	692	co2	1721	PPCDC	0
947	4ppcys	1609	pan4p	1721	PPCDC	1
135	atp	281	adp	1722	PPCK	1
135	atp	692	co2	1722	PPCK	0
135	atp	959	pep	1722	PPCK	0
1318	oaa	281	adp	1722	PPCK	0
1318	oaa	692	co2	1722	PPCK	0
1318	oaa	959	pep	1722	PPCK	1
948	ppcoa	838	succoa	1723	PPCSCT	1
948	ppcoa	1345	ppa	1723	PPCSCT	1
1008	succ	838	succoa	1723	PPCSCT	1
1008	succ	1345	ppa	1723	PPCSCT	0
109	h2o	798	gdp	1724	PPGPPDP	0
109	h2o	1192	ppi	1724	PPGPPDP	0
1493	ppgpp	798	gdp	1724	PPGPPDP	1
1493	ppgpp	1192	ppi	1724	PPGPPDP	0
135	atp	281	adp	1725	PPK2r	1
135	atp	1491	pppi	1725	PPK2r	0
1192	ppi	281	adp	1725	PPK2r	0
1192	ppi	1491	pppi	1725	PPK2r	0
135	atp	281	adp	1726	PPKr	1
135	atp	1192	ppi	1726	PPKr	0
962	pi	281	adp	1726	PPKr	0
962	pi	1192	ppi	1726	PPKr	0
949	r1p	983	r5p	1727	PPM	1
950	2dr1p	454	2dr5p	1728	PPM2	1
366	cys-L	98	h	1729	PPNCL2	0

366	cys-L	330	cmp	1729	PPNCL2	0
366	cys-L	947	4ppcys	1729	PPNCL2	1
366	cys-L	1192	ppi	1729	PPNCL2	0
392	ctp	98	h	1729	PPNCL2	0
392	ctp	330	cmp	1729	PPNCL2	1
392	ctp	947	4ppcys	1729	PPNCL2	0
392	ctp	1192	ppi	1729	PPNCL2	0
951	4ppan	98	h	1729	PPNCL2	0
951	4ppan	330	cmp	1729	PPNCL2	0
951	4ppan	947	4ppcys	1729	PPNCL2	1
951	4ppan	1192	ppi	1729	PPNCL2	0
856	nad	692	co2	1730	PPND	0
856	nad	870	nadh	1730	PPND	1
856	nad	1218	34hpp	1730	PPND	0
1255	pphn	692	co2	1730	PPND	0
1255	pphn	870	nadh	1730	PPND	0
1255	pphn	1218	34hpp	1730	PPND	1
98	h	109	h2o	1731	PPNDH	0
98	h	692	co2	1731	PPNDH	0
98	h	1562	phpyr	1731	PPNDH	0
1255	pphn	109	h2o	1731	PPNDH	0
1255	pphn	692	co2	1731	PPNDH	0
1255	pphn	1562	phpyr	1731	PPNDH	1
928	o2	109	h2o	1732	PPPGO	0
928	o2	1346	ppp9	1732	PPPGO	0
1494	pppg9	109	h2o	1732	PPPGO	0
1494	pppg9	1346	ppp9	1732	PPPGO	0
540	fum	1008	succ	1733	PPPGO3	1
540	fum	1346	ppp9	1733	PPPGO3	0
1494	pppg9	1008	succ	1733	PPPGO3	0
1494	pppg9	1346	ppp9	1733	PPPGO3	1
98	h	425	cechddd	1734	PPPNDO	0
98	h	856	nad	1734	PPPNDO	0
870	nadh	425	cechddd	1734	PPPNDO	0
870	nadh	856	nad	1734	PPPNDO	1
928	o2	425	cechddd	1734	PPPNDO	0
928	o2	856	nad	1734	PPPNDO	0
1563	pppn	425	cechddd	1734	PPPNDO	1
1563	pppn	856	nad	1734	PPPNDO	0
437	h[p]	98	h	1735	PPPNt2rpp	0
437	h[p]	1563	pppn	1735	PPPNt2rpp	0
1177	pppn[p]	98	h	1735	PPPNt2rpp	0
1177	pppn[p]	1563	pppn	1735	PPPNt2rpp	1
952	pppn[e]	1177	pppn[p]	1736	PPPNtex	1
109	h2o	98	h	1737	PPS	0
109	h2o	177	amp	1737	PPS	0
109	h2o	959	pep	1737	PPS	0
109	h2o	962	pi	1737	PPS	0
135	atp	98	h	1737	PPS	0
135	atp	177	amp	1737	PPS	1
135	atp	959	pep	1737	PPS	0
135	atp	962	pi	1737	PPS	0
1148	pyr	98	h	1737	PPS	0
1148	pyr	177	amp	1737	PPS	0
1148	pyr	959	pep	1737	PPS	1
1148	pyr	962	pi	1737	PPS	0

684	h2o[p]	688	h2[p]	1738	PPTHpp	0
684	h2o[p]	1173	pi[p]	1738	PPTHpp	0
1178	ppt[p]	688	h2[p]	1738	PPTHpp	0
1178	ppt[p]	1173	pi[p]	1738	PPTHpp	0
953	ppt[e]	1178	ppt[p]	1739	PPTtex	1
135	atp	98	h	1740	PRAGSr	0
135	atp	281	adp	1740	PRAGSr	1
135	atp	962	pi	1740	PRAGSr	0
135	atp	1402	gar	1740	PRAGSr	0
642	gly	98	h	1740	PRAGSr	0
642	gly	281	adp	1740	PRAGSr	0
642	gly	962	pi	1740	PRAGSr	0
642	gly	1402	gar	1740	PRAGSr	1
1642	pram	98	h	1740	PRAGSr	0
1642	pram	281	adp	1740	PRAGSr	0
1642	pram	962	pi	1740	PRAGSr	0
1642	pram	1402	gar	1740	PRAGSr	1
954	pran	719	2cpr5p	1741	PRAIi	1
135	atp	98	h	1742	PRAIS	0
135	atp	211	air	1742	PRAIS	0
135	atp	281	adp	1742	PRAIS	1
135	atp	962	pi	1742	PRAIS	0
1495	fpram	98	h	1742	PRAIS	0
1495	fpram	211	air	1742	PRAIS	1
1495	fpram	281	adp	1742	PRAIS	0
1495	fpram	962	pi	1742	PRAIS	0
109	h2o	955	prfp	1743	PRAMPC	0
1496	prbamp	955	prfp	1743	PRAMPC	1
135	atp	98	h	1744	PRASCS	0
135	atp	188	25aics	1744	PRASCS	0
135	atp	281	adp	1744	PRASCS	1
135	atp	962	pi	1744	PRASCS	0
189	asp-L	98	h	1744	PRASCS	0
189	asp-L	188	25aics	1744	PRASCS	1
189	asp-L	281	adp	1744	PRASCS	0
189	asp-L	962	pi	1744	PRASCS	0
212	5aizc	98	h	1744	PRASCS	0
212	5aizc	188	25aics	1744	PRASCS	1
212	5aizc	281	adp	1744	PRASCS	0
212	5aizc	962	pi	1744	PRASCS	0
109	h2o	98	h	1745	PRATPP	0
109	h2o	1192	ppi	1745	PRATPP	0
109	h2o	1496	prbamp	1745	PRATPP	0
1497	prbatp	98	h	1745	PRATPP	0
1497	prbatp	1192	ppi	1745	PRATPP	0
1497	prbatp	1496	prbamp	1745	PRATPP	1
109	h2o	98	h	1746	PRFGS	0
109	h2o	281	adp	1746	PRFGS	0
109	h2o	624	glu-L	1746	PRFGS	0
109	h2o	962	pi	1746	PRFGS	0
109	h2o	1495	fpram	1746	PRFGS	0
135	atp	98	h	1746	PRFGS	0
135	atp	281	adp	1746	PRFGS	1
135	atp	624	glu-L	1746	PRFGS	0
135	atp	962	pi	1746	PRFGS	0
135	atp	1495	fpram	1746	PRFGS	0

625	gln-L	98	h	1746	PRFGS	0
625	gln-L	281	adp	1746	PRFGS	0
625	gln-L	624	glu-L	1746	PRFGS	1
625	gln-L	962	pi	1746	PRFGS	0
625	gln-L	1495	fpram	1746	PRFGS	0
1114	fgam	98	h	1746	PRFGS	0
1114	fgam	281	adp	1746	PRFGS	0
1114	fgam	624	glu-L	1746	PRFGS	0
1114	fgam	962	pi	1746	PRFGS	0
1114	fgam	1495	fpram	1746	PRFGS	1
955	prfp	1179	prlp	1747	PRMICI	1
109	h2o	98	h	1748	PROabcpp	0
109	h2o	281	adp	1748	PROabcpp	0
109	h2o	962	pi	1748	PROabcpp	0
109	h2o	1498	pro-L	1748	PROabcpp	0
135	atp	98	h	1748	PROabcpp	0
135	atp	281	adp	1748	PROabcpp	1
135	atp	962	pi	1748	PROabcpp	0
135	atp	1498	pro-L	1748	PROabcpp	0
1181	pro-L[p]	98	h	1748	PROabcpp	0
1181	pro-L[p]	281	adp	1748	PROabcpp	0
1181	pro-L[p]	962	pi	1748	PROabcpp	0
1181	pro-L[p]	1498	pro-L	1748	PROabcpp	1
158	fad	98	h	1749	PROD2	0
158	fad	520	fadh2	1749	PROD2	1
158	fad	920	1pyr5c	1749	PROD2	0
1498	pro-L	98	h	1749	PROD2	0
1498	pro-L	520	fadh2	1749	PROD2	0
1498	pro-L	920	1pyr5c	1749	PROD2	1
109	h2o	98	h	1750	PROGLYabcpp	0
109	h2o	281	adp	1750	PROGLYabcpp	0
109	h2o	962	pi	1750	PROGLYabcpp	0
109	h2o	1381	progly	1750	PROGLYabcpp	0
135	atp	98	h	1750	PROGLYabcpp	0
135	atp	281	adp	1750	PROGLYabcpp	1
135	atp	962	pi	1750	PROGLYabcpp	0
135	atp	1381	progly	1750	PROGLYabcpp	0
1180	progly[p]	98	h	1750	PROGLYabcpp	0
1180	progly[p]	281	adp	1750	PROGLYabcpp	0
1180	progly[p]	962	pi	1750	PROGLYabcpp	0
1180	progly[p]	1381	progly	1750	PROGLYabcpp	1
956	progly[e]	1180	progly[p]	1751	PROGLYtex	1
437	h[p]	98	h	1752	PROt2rpp	0
437	h[p]	1498	pro-L	1752	PROt2rpp	0
1181	pro-L[p]	98	h	1752	PROt2rpp	0
1181	pro-L[p]	1498	pro-L	1752	PROt2rpp	1
941	na1[p]	1344	na1	1753	PROt4pp	0
941	na1[p]	1498	pro-L	1753	PROt4pp	0
1181	pro-L[p]	1344	na1	1753	PROt4pp	0
1181	pro-L[p]	1498	pro-L	1753	PROt4pp	1
957	pro-L[e]	1181	pro-L[p]	1754	PROtex	1
135	atp	177	amp	1755	PROTRS	1
135	atp	1192	ppi	1755	PROTRS	0
135	atp	1659	protrna	1755	PROTRS	0
1498	pro-L	177	amp	1755	PROTRS	0
1498	pro-L	1192	ppi	1755	PROTRS	0

1498	pro-L	1659	protrna	1755	PROTRS	1
1643	trnapro	177	amp	1755	PROTRS	0
1643	trnapro	1192	ppi	1755	PROTRS	0
1643	trnapro	1659	protrna	1755	PROTRS	1
135	atp	98	h	1756	PRPPS	0
135	atp	177	amp	1756	PRPPS	1
135	atp	1052	prpp	1756	PRPPS	0
983	r5p	98	h	1756	PRPPS	0
983	r5p	177	amp	1756	PRPPS	0
983	r5p	1052	prpp	1756	PRPPS	1
437	h[p]	98	h	1757	PSCLYSt2pp	0
437	h[p]	542	psclys	1757	PSCLYSt2pp	0
1182	psclys[p]	98	h	1757	PSCLYSt2pp	0
1182	psclys[p]	542	psclys	1757	PSCLYSt2pp	1
958	psclys[e]	1182	psclys[p]	1758	PSCLYStex	1
959	pep	309	3psme	1759	PSCVT	1
959	pep	962	pi	1759	PSCVT	0
1499	skm5p	309	3psme	1759	PSCVT	1
1499	skm5p	962	pi	1759	PSCVT	0
98	h	692	co2	1760	PSD120	0
98	h	1565	pe120	1760	PSD120	0
1500	ps120	692	co2	1760	PSD120	0
1500	ps120	1565	pe120	1760	PSD120	1
98	h	692	co2	1761	PSD140	0
98	h	1566	pe140	1761	PSD140	0
1501	ps140	692	co2	1761	PSD140	0
1501	ps140	1566	pe140	1761	PSD140	1
98	h	692	co2	1762	PSD141	0
98	h	1567	pe141	1762	PSD141	0
1502	ps141	692	co2	1762	PSD141	0
1502	ps141	1567	pe141	1762	PSD141	1
98	h	692	co2	1763	PSD160	0
98	h	1568	pe160	1763	PSD160	0
1503	ps160	692	co2	1763	PSD160	0
1503	ps160	1568	pe160	1763	PSD160	1
98	h	692	co2	1764	PSD161	0
98	h	1387	pe161	1764	PSD161	0
1504	ps161	692	co2	1764	PSD161	0
1504	ps161	1387	pe161	1764	PSD161	1
98	h	692	co2	1765	PSD180	0
98	h	1569	pe180	1765	PSD180	0
1505	ps180	692	co2	1765	PSD180	0
1505	ps180	1569	pe180	1765	PSD180	1
98	h	692	co2	1766	PSD181	0
98	h	1388	pe181	1766	PSD181	0
1506	ps181	692	co2	1766	PSD181	0
1506	ps181	1388	pe181	1766	PSD181	1
624	glu-L	213	akg	1767	PSERT	1
624	glu-L	1507	pser-L	1767	PSERT	0
960	3php	213	akg	1767	PSERT	0
960	3php	1507	pser-L	1767	PSERT	1
961	pser-L[e]	1183	pser-L[p]	1768	PSERtex	1
109	h2o	604	ser-L	1769	PSP_L	0
109	h2o	962	pi	1769	PSP_L	0
1507	pser-L	604	ser-L	1769	PSP_L	1
1507	pser-L	962	pi	1769	PSP_L	0

684	h2o[p]	1173	pi[p]	1770	PSP_Lpp	0
684	h2o[p]	1193	ser-L[p]	1770	PSP_Lpp	0
1183	pser-L[p]	1173	pi[p]	1770	PSP_Lpp	0
1183	pser-L[p]	1193	ser-L[p]	1770	PSP_Lpp	1
298	cdpdddecg	98	h	1771	PSSA120	0
298	cdpdddecg	330	cmp	1771	PSSA120	1
298	cdpdddecg	1500	ps120	1771	PSSA120	1
604	ser-L	98	h	1771	PSSA120	0
604	ser-L	330	cmp	1771	PSSA120	1
604	ser-L	1500	ps120	1771	PSSA120	0
299	cdpdtdecg	98	h	1772	PSSA140	0
299	cdpdtdecg	330	cmp	1772	PSSA140	1
299	cdpdtdecg	1501	ps140	1772	PSSA140	1
604	ser-L	98	h	1772	PSSA140	0
604	ser-L	330	cmp	1772	PSSA140	1
604	ser-L	1501	ps140	1772	PSSA140	0
300	cdpdtdec7eg	98	h	1773	PSSA141	0
300	cdpdtdec7eg	330	cmp	1773	PSSA141	1
300	cdpdtdec7eg	1502	ps141	1773	PSSA141	1
604	ser-L	98	h	1773	PSSA141	0
604	ser-L	330	cmp	1773	PSSA141	1
604	ser-L	1502	ps141	1773	PSSA141	0
301	cdpdhdecg	98	h	1774	PSSA160	0
301	cdpdhdecg	330	cmp	1774	PSSA160	1
301	cdpdhdecg	1503	ps160	1774	PSSA160	1
604	ser-L	98	h	1774	PSSA160	0
604	ser-L	330	cmp	1774	PSSA160	1
604	ser-L	1503	ps160	1774	PSSA160	0
302	cdpdhdec9eg	98	h	1775	PSSA161	0
302	cdpdhdec9eg	330	cmp	1775	PSSA161	1
302	cdpdhdec9eg	1504	ps161	1775	PSSA161	1
604	ser-L	98	h	1775	PSSA161	0
604	ser-L	330	cmp	1775	PSSA161	1
604	ser-L	1504	ps161	1775	PSSA161	0
303	cdpdodecg	98	h	1776	PSSA180	0
303	cdpdodecg	330	cmp	1776	PSSA180	1
303	cdpdodecg	1505	ps180	1776	PSSA180	1
604	ser-L	98	h	1776	PSSA180	0
604	ser-L	330	cmp	1776	PSSA180	1
604	ser-L	1505	ps180	1776	PSSA180	0
304	cdpdodec11eg	98	h	1777	PSSA181	0
304	cdpdodec11eg	330	cmp	1777	PSSA181	1
304	cdpdodec11eg	1506	ps181	1777	PSSA181	1
604	ser-L	98	h	1777	PSSA181	0
604	ser-L	330	cmp	1777	PSSA181	1
604	ser-L	1506	ps181	1777	PSSA181	0
948	ppcoa	927	coa	1778	PTA2	1
948	ppcoa	1492	ppap	1778	PTA2	1
962	pi	927	coa	1778	PTA2	0
962	pi	1492	ppap	1778	PTA2	0
128	accoa	927	coa	1779	PTAr	1
128	accoa	1075	actp	1779	PTAr	1
962	pi	927	coa	1779	PTAr	0
962	pi	1075	actp	1779	PTAr	0
684	h2o[p]	1173	pi[p]	1780	PTHRpp	0
684	h2o[p]	1208	thr-L[p]	1780	PTHRpp	0

1207	thrp[p]	1173	pi[p]	1780	PTHRpp	0
1207	thrp[p]	1208	thr-L[p]	1780	PTHRpp	1
98	h	1192	ppi	1781	PTPATi	0
98	h	1347	dpcoa	1781	PTPATi	0
135	atp	1192	ppi	1781	PTPATi	0
135	atp	1347	dpcoa	1781	PTPATi	1
1609	pan4p	1192	ppi	1781	PTPATi	0
1609	pan4p	1347	dpcoa	1781	PTPATi	1
109	h2o	98	h	1782	PTRCabcpp	0
109	h2o	281	adp	1782	PTRCabcpp	0
109	h2o	962	pi	1782	PTRCabcpp	0
109	h2o	1245	ptrc	1782	PTRCabcpp	0
135	atp	98	h	1782	PTRCabcpp	0
135	atp	281	adp	1782	PTRCabcpp	1
135	atp	962	pi	1782	PTRCabcpp	0
135	atp	1245	ptrc	1782	PTRCabcpp	0
1184	ptrc[p]	98	h	1782	PTRCabcpp	0
1184	ptrc[p]	281	adp	1782	PTRCabcpp	0
1184	ptrc[p]	962	pi	1782	PTRCabcpp	0
1184	ptrc[p]	1245	ptrc	1782	PTRCabcpp	1
963	orn	1167	orn[p]	1783	PTRCORNt7pp	1
963	orn	1245	ptrc	1783	PTRCORNt7pp	0
1184	ptrc[p]	1167	orn[p]	1783	PTRCORNt7pp	0
1184	ptrc[p]	1245	ptrc	1783	PTRCORNt7pp	1
437	h[p]	98	h	1784	PTRCt2pp	0
437	h[p]	1245	ptrc	1784	PTRCt2pp	0
1184	ptrc[p]	98	h	1784	PTRCt2pp	0
1184	ptrc[p]	1245	ptrc	1784	PTRCt2pp	1
213	akg	124	4abutn	1785	PTRCTA	0
213	akg	624	glu-L	1785	PTRCTA	1
1245	ptrc	124	4abutn	1785	PTRCTA	1
1245	ptrc	624	glu-L	1785	PTRCTA	0
964	ptrc[e]	1184	ptrc[p]	1786	PTRCtex	1
171	adn	174	ade	1787	PUNP1	1
171	adn	949	r1p	1787	PUNP1	1
962	pi	174	ade	1787	PUNP1	0
962	pi	949	r1p	1787	PUNP1	0
374	dad-2	174	ade	1788	PUNP2	1
374	dad-2	950	2dr1p	1788	PUNP2	1
962	pi	174	ade	1788	PUNP2	0
962	pi	950	2dr1p	1788	PUNP2	0
962	pi	676	gua	1789	PUNP3	0
962	pi	949	r1p	1789	PUNP3	0
965	gsn	676	gua	1789	PUNP3	1
965	gsn	949	r1p	1789	PUNP3	1
962	pi	676	gua	1790	PUNP4	0
962	pi	950	2dr1p	1790	PUNP4	0
966	dgsn	676	gua	1790	PUNP4	1
966	dgsn	950	2dr1p	1790	PUNP4	1
962	pi	713	hxan	1791	PUNP5	0
962	pi	949	r1p	1791	PUNP5	0
967	ins	713	hxan	1791	PUNP5	1
967	ins	949	r1p	1791	PUNP5	1
962	pi	713	hxan	1792	PUNP6	0
962	pi	950	2dr1p	1792	PUNP6	0
968	din	713	hxan	1792	PUNP6	1

968	din	950	2dr1p	1792	PUNP6	1
962	pi	949	r1p	1793	PUNP7	0
962	pi	1231	xan	1793	PUNP7	0
1508	xtsn	949	r1p	1793	PUNP7	1
1508	xtsn	1231	xan	1793	PUNP7	1
109	h2o	292	h2o2	1794	PYAM5PO	0
109	h2o	1160	nh4	1794	PYAM5PO	0
109	h2o	1377	pydx5p	1794	PYAM5PO	0
928	o2	292	h2o2	1794	PYAM5PO	0
928	o2	1160	nh4	1794	PYAM5PO	0
928	o2	1377	pydx5p	1794	PYAM5PO	0
1247	pyam5p	292	h2o2	1794	PYAM5PO	0
1247	pyam5p	1160	nh4	1794	PYAM5PO	0
1247	pyam5p	1377	pydx5p	1794	PYAM5PO	1
135	atp	98	h	1795	PYDAMK	0
135	atp	281	adp	1795	PYDAMK	1
135	atp	1247	pyam5p	1795	PYDAMK	0
1509	pydam	98	h	1795	PYDAMK	0
1509	pydam	281	adp	1795	PYDAMK	0
1509	pydam	1247	pyam5p	1795	PYDAMK	1
135	atp	98	h	1796	PYDXK	0
135	atp	281	adp	1796	PYDXK	1
135	atp	1377	pydx5p	1796	PYDXK	0
1510	pydx	98	h	1796	PYDXK	0
1510	pydx	281	adp	1796	PYDXK	0
1510	pydx	1377	pydx5p	1796	PYDXK	1
135	atp	98	h	1797	PYDXNK	0
135	atp	281	adp	1797	PYDXNK	1
135	atp	1467	pdx5p	1797	PYDXNK	0
1511	pydxn	98	h	1797	PYDXNK	0
1511	pydxn	281	adp	1797	PYDXNK	0
1511	pydxn	1467	pdx5p	1797	PYDXNK	1
109	h2o	962	pi	1798	PYDXPP	0
109	h2o	1510	pydx	1798	PYDXPP	0
1377	pydx5p	962	pi	1798	PYDXPP	0
1377	pydx5p	1510	pydx	1798	PYDXPP	1
98	h	135	atp	1799	PYK	0
98	h	1148	pyr	1799	PYK	0
281	adp	135	atp	1799	PYK	1
281	adp	1148	pyr	1799	PYK	0
959	pep	135	atp	1799	PYK	0
959	pep	1148	pyr	1799	PYK	1
962	pi	949	r1p	1800	PYNP2r	0
962	pi	1552	ura	1800	PYNP2r	0
1512	uri	949	r1p	1800	PYNP2r	1
1512	uri	1552	ura	1800	PYNP2r	1
437	h[p]	98	h	1801	PYRt2rpp	0
437	h[p]	1148	pyr	1801	PYRt2rpp	0
1185	pyr[p]	98	h	1801	PYRt2rpp	0
1185	pyr[p]	1148	pyr	1801	PYRt2rpp	1
969	pyr[e]	1185	pyr[p]	1802	PYRtex	1
928	o2	98	h	1803	QMO2	0
928	o2	1003	q8	1803	QMO2	0
928	o2	1527	o2s	1803	QMO2	0
1443	q8h2	98	h	1803	QMO2	0
1443	q8h2	1003	q8	1803	QMO2	1

1443	q8h2	1527	o2s	1803	QMO2	0
867	mql8	98	h	1804	QMO3	0
867	mql8	1282	mqn8	1804	QMO3	1
867	mql8	1527	o2s	1804	QMO3	0
928	o2	98	h	1804	QMO3	0
928	o2	1282	mqn8	1804	QMO3	0
928	o2	1527	o2s	1804	QMO3	0
826	dhap	109	h2o	1805	QULNS	0
826	dhap	962	pi	1805	QULNS	0
826	dhap	1639	quln	1805	QULNS	1
1513	iasp	109	h2o	1805	QULNS	0
1513	iasp	962	pi	1805	QULNS	0
1513	iasp	1639	quln	1805	QULNS	1
135	atp	281	adp	1806	R15BPK	1
135	atp	1052	prpp	1806	R15BPK	0
1514	r15bp	281	adp	1806	R15BPK	0
1514	r15bp	1052	prpp	1806	R15BPK	1
135	atp	98	h	1807	R1PK	0
135	atp	281	adp	1807	R1PK	1
135	atp	1514	r15bp	1807	R1PK	0
949	r1p	98	h	1807	R1PK	0
949	r1p	281	adp	1807	R1PK	0
949	r1p	1514	r15bp	1807	R1PK	1
109	h2o	962	pi	1808	R5PP	0
109	h2o	1361	rib-D	1808	R5PP	0
983	r5p	962	pi	1808	R5PP	0
983	r5p	1361	rib-D	1808	R5PP	1
684	h2o[p]	1173	pi[p]	1809	R5PPpp	0
684	h2o[p]	1188	rib-D[p]	1809	R5PPpp	0
1186	r5p[p]	1173	pi[p]	1809	R5PPpp	0
1186	r5p[p]	1188	rib-D[p]	1809	R5PPpp	1
970	r5p[e]	1186	r5p[p]	1810	R5Ptex	1
135	atp	98	h	1811	RBFK	0
135	atp	281	adp	1811	RBFK	1
135	atp	538	fmn	1811	RBFK	0
1515	ribflv	98	h	1811	RBFK	0
1515	ribflv	281	adp	1811	RBFK	0
1515	ribflv	538	fmn	1811	RBFK	1
971	4r5au	109	h2o	1812	RBFSa	0
971	4r5au	962	pi	1812	RBFSa	0
971	4r5au	972	dmlz	1812	RBFSa	1
1260	db4p	109	h2o	1812	RBFSa	0
1260	db4p	962	pi	1812	RBFSa	0
1260	db4p	972	dmlz	1812	RBFSa	1
972	dmlz	971	4r5au	1813	RBFSb	1
972	dmlz	1515	ribflv	1813	RBFSb	1
135	atp	98	h	1814	RBK	0
135	atp	281	adp	1814	RBK	1
135	atp	983	r5p	1814	RBK	0
1361	rib-D	98	h	1814	RBK	0
1361	rib-D	281	adp	1814	RBK	0
1361	rib-D	983	r5p	1814	RBK	1
135	atp	98	h	1815	RBK_L1	0
135	atp	281	adp	1815	RBK_L1	1
135	atp	973	ru5p-L	1815	RBK_L1	0
1086	rbl-L	98	h	1815	RBK_L1	0

1086	rbl-L	281	adp	1815	RBK_L1	1
1086	rbl-L	973	ru5p-L	1815	RBK_L1	0
973	ru5p-L	1187	xu5p-D	1816	RBP4E	1
974	dtdprmn	98	h	1817	RHAT1	0
974	dtdprmn	616	icolipa	1817	RHAT1	1
974	dtdprmn	1239	dtdp	1817	RHAT1	1
1516	kphphhlipa	98	h	1817	RHAT1	0
1516	kphphhlipa	616	icolipa	1817	RHAT1	1
1516	kphphhlipa	1239	dtdp	1817	RHAT1	0
975	rhcys	428	dhptd	1818	RHCCE	1
975	rhcys	693	hcys-L	1818	RHCCE	1
109	h2o	98	h	1819	RIBabcpp	0
109	h2o	281	adp	1819	RIBabcpp	0
109	h2o	962	pi	1819	RIBabcpp	0
109	h2o	1361	rib-D	1819	RIBabcpp	0
135	atp	98	h	1819	RIBabcpp	0
135	atp	281	adp	1819	RIBabcpp	1
135	atp	962	pi	1819	RIBabcpp	0
135	atp	1361	rib-D	1819	RIBabcpp	0
1188	rib-D[p]	98	h	1819	RIBabcpp	0
1188	rib-D[p]	281	adp	1819	RIBabcpp	0
1188	rib-D[p]	962	pi	1819	RIBabcpp	0
1188	rib-D[p]	1361	rib-D	1819	RIBabcpp	1
976	rib-D[e]	1188	rib-D[p]	1820	RIBtex	1
977	rmn	1189	rml	1821	RMI	1
135	atp	98	h	1822	RMK	0
135	atp	281	adp	1822	RMK	1
135	atp	979	rml1p	1822	RMK	0
1189	rml	98	h	1822	RMK	0
1189	rml	281	adp	1822	RMK	0
1189	rml	979	rml1p	1822	RMK	1
978	rmn[e]	1190	rmn[p]	1823	RMNtex	1
437	h[p]	98	h	1824	RMNtpp	0
437	h[p]	977	rmn	1824	RMNtpp	0
1190	rmn[p]	98	h	1824	RMNtpp	0
1190	rmn[p]	977	rmn	1824	RMNtpp	1
979	rml1p	826	dhap	1825	RMPA	0
979	rml1p	1422	lald-L	1825	RMPA	1
281	adp	109	h2o	1826	RNDR1	0
281	adp	1348	dadp	1826	RNDR1	1
281	adp	1594	trdox	1826	RNDR1	0
981	trdrd	109	h2o	1826	RNDR1	0
981	trdrd	1348	dadp	1826	RNDR1	0
981	trdrd	1594	trdox	1826	RNDR1	1
281	adp	109	h2o	1827	RNDR1b	0
281	adp	665	grxox	1827	RNDR1b	0
281	adp	1348	dadp	1827	RNDR1b	1
925	grxrd	109	h2o	1827	RNDR1b	0
925	grxrd	665	grxox	1827	RNDR1b	1
925	grxrd	1348	dadp	1827	RNDR1b	0
798	gdp	109	h2o	1828	RNDR2	0
798	gdp	1349	dgdp	1828	RNDR2	1
798	gdp	1594	trdox	1828	RNDR2	0
981	trdrd	109	h2o	1828	RNDR2	0
981	trdrd	1349	dgdp	1828	RNDR2	0
981	trdrd	1594	trdox	1828	RNDR2	1

798	gdp	109	h2o	1829	RNDR2b	0
798	gdp	665	grxox	1829	RNDR2b	0
798	gdp	1349	dgdp	1829	RNDR2b	1
925	grxrd	109	h2o	1829	RNDR2b	0
925	grxrd	665	grxox	1829	RNDR2b	1
925	grxrd	1349	dgdp	1829	RNDR2b	0
980	cdp	109	h2o	1830	RNDR3	0
980	cdp	1350	dcdp	1830	RNDR3	1
980	cdp	1594	trdox	1830	RNDR3	0
981	trdrd	109	h2o	1830	RNDR3	0
981	trdrd	1350	dcdp	1830	RNDR3	0
981	trdrd	1594	trdox	1830	RNDR3	1
925	grxrd	109	h2o	1831	RNDR3b	0
925	grxrd	665	grxox	1831	RNDR3b	1
925	grxrd	1350	dcdp	1831	RNDR3b	0
980	cdp	109	h2o	1831	RNDR3b	0
980	cdp	665	grxox	1831	RNDR3b	0
980	cdp	1350	dcdp	1831	RNDR3b	1
981	trdrd	109	h2o	1832	RNDR4	0
981	trdrd	1351	dudp	1832	RNDR4	0
981	trdrd	1594	trdox	1832	RNDR4	1
1440	udp	109	h2o	1832	RNDR4	0
1440	udp	1351	dudp	1832	RNDR4	1
1440	udp	1594	trdox	1832	RNDR4	0
925	grxrd	109	h2o	1833	RNDR4b	0
925	grxrd	665	grxox	1833	RNDR4b	1
925	grxrd	1351	dudp	1833	RNDR4b	0
1440	udp	109	h2o	1833	RNDR4b	0
1440	udp	665	grxox	1833	RNDR4b	0
1440	udp	1351	dudp	1833	RNDR4b	1
135	atp	109	h2o	1834	RNTR1c	0
135	atp	393	datp	1834	RNTR1c	1
135	atp	537	fldox	1834	RNTR1c	0
982	fldrd	109	h2o	1834	RNTR1c	0
982	fldrd	393	datp	1834	RNTR1c	0
982	fldrd	537	fldox	1834	RNTR1c	1
673	gtp	109	h2o	1835	RNTR2c	0
673	gtp	537	fldox	1835	RNTR2c	0
673	gtp	892	dgtp	1835	RNTR2c	1
982	fldrd	109	h2o	1835	RNTR2c	0
982	fldrd	537	fldox	1835	RNTR2c	1
982	fldrd	892	dgtp	1835	RNTR2c	0
392	ctp	109	h2o	1836	RNTR3c	0
392	ctp	396	dctp	1836	RNTR3c	1
392	ctp	537	fldox	1836	RNTR3c	0
982	fldrd	109	h2o	1836	RNTR3c	0
982	fldrd	396	dctp	1836	RNTR3c	0
982	fldrd	537	fldox	1836	RNTR3c	1
982	fldrd	109	h2o	1837	RNTR4c	0
982	fldrd	466	dutp	1837	RNTR4c	0
982	fldrd	537	fldox	1837	RNTR4c	1
1447	utp	109	h2o	1837	RNTR4c	0
1447	utp	466	dutp	1837	RNTR4c	1
1447	utp	537	fldox	1837	RNTR4c	0
117	ru5p-D	1187	xu5p-D	1838	RPE	1
983	r5p	117	ru5p-D	1839	RPI	1

109	h2o	962	pi	1840	RZ5PP	0
109	h2o	1372	rdmbzi	1840	RZ5PP	0
984	5prdmbz	962	pi	1840	RZ5PP	0
984	5prdmbz	1372	rdmbzi	1840	RZ5PP	1
985	s7p	1352	gmhep7p	1841	S7PI	1
98	h	692	co2	1842	SADH	0
98	h	1160	nh4	1842	SADH	0
98	h	1525	sucorn	1842	SADH	0
109	h2o	692	co2	1842	SADH	0
109	h2o	1160	nh4	1842	SADH	0
109	h2o	1525	sucorn	1842	SADH	0
1644	sucarg	692	co2	1842	SADH	0
1644	sucarg	1160	nh4	1842	SADH	0
1644	sucarg	1525	sucorn	1842	SADH	1
109	h2o	186	aps	1843	SADT2	0
109	h2o	798	gdp	1843	SADT2	0
109	h2o	962	pi	1843	SADT2	0
109	h2o	1192	ppi	1843	SADT2	0
135	atp	186	aps	1843	SADT2	1
135	atp	798	gdp	1843	SADT2	0
135	atp	962	pi	1843	SADT2	0
135	atp	1192	ppi	1843	SADT2	0
673	gtp	186	aps	1843	SADT2	0
673	gtp	798	gdp	1843	SADT2	1
673	gtp	962	pi	1843	SADT2	0
673	gtp	1192	ppi	1843	SADT2	0
1600	so4	186	aps	1843	SADT2	0
1600	so4	798	gdp	1843	SADT2	0
1600	so4	962	pi	1843	SADT2	0
1600	so4	1192	ppi	1843	SADT2	0
109	h2o	292	h2o2	1844	SARCOX	0
109	h2o	505	fald	1844	SARCOX	0
109	h2o	642	gly	1844	SARCOX	0
928	o2	292	h2o2	1844	SARCOX	0
928	o2	505	fald	1844	SARCOX	0
928	o2	642	gly	1844	SARCOX	0
1645	sarcs	292	h2o2	1844	SARCOX	0
1645	sarcs	505	fald	1844	SARCOX	1
1645	sarcs	642	gly	1844	SARCOX	1
856	nad	98	h	1845	SBTPD	0
856	nad	499	f6p	1845	SBTPD	0
856	nad	870	nadh	1845	SBTPD	1
1517	sbt6p	98	h	1845	SBTPD	0
1517	sbt6p	499	f6p	1845	SBTPD	1
1517	sbt6p	870	nadh	1845	SBTPD	0
959	pep	1148	pyr	1846	SBTptspp	1
959	pep	1517	sbt6p	1846	SBTptspp	0
1191	sbt-D[p]	1148	pyr	1846	SBTptspp	0
1191	sbt-D[p]	1517	sbt6p	1846	SBTptspp	1
986	sbt-D[e]	1191	sbt-D[p]	1847	SBTtex	1
109	h2o	390	26dap-LL	1848	SDPDS	0
109	h2o	1008	succ	1848	SDPDS	0
1518	sl26da	390	26dap-LL	1848	SDPDS	1
1518	sl26da	1008	succ	1848	SDPDS	1
213	akg	624	glu-L	1849	SDPTA	1
213	akg	1564	sl2a6o	1849	SDPTA	0

1518	sl26da	624	glu-L	1849	SDPTA	0
1518	sl26da	1564	sl2a6o	1849	SDPTA	1
987	selnp	98	h	1850	SELCYSS	0
987	selnp	962	pi	1850	SELCYSS	0
987	selnp	1660	sectrna	1850	SELCYSS	1
1519	sertrna(sec)	98	h	1850	SELCYSS	0
1519	sertrna(sec)	962	pi	1850	SELCYSS	0
1519	sertrna(sec)	1660	sectrna	1850	SELCYSS	1
109	h2o	177	amp	1851	SELNPS	0
109	h2o	962	pi	1851	SELNPS	0
109	h2o	987	selnp	1851	SELNPS	0
135	atp	177	amp	1851	SELNPS	1
135	atp	962	pi	1851	SELNPS	0
135	atp	987	selnp	1851	SELNPS	0
1646	seln	177	amp	1851	SELNPS	0
1646	seln	962	pi	1851	SELNPS	0
1646	seln	987	selnp	1851	SELNPS	1
98	h	1192	ppi	1852	SERASr	0
98	h	1394	seramp	1852	SERASr	0
135	atp	1192	ppi	1852	SERASr	0
135	atp	1394	seramp	1852	SERASr	1
604	ser-L	1192	ppi	1852	SERASr	0
604	ser-L	1394	seramp	1852	SERASr	1
128	accoa	168	acser	1853	SERAT	1
128	accoa	927	coa	1853	SERAT	1
604	ser-L	168	acser	1853	SERAT	1
604	ser-L	927	coa	1853	SERAT	0
988	ser-D	1148	pyr	1854	SERD_D	1
988	ser-D	1160	nh4	1854	SERD_D	0
604	ser-L	1148	pyr	1855	SERD_L	1
604	ser-L	1160	nh4	1855	SERD_L	0
437	h[p]	98	h	1856	SERt2rpp	0
437	h[p]	604	ser-L	1856	SERt2rpp	0
1193	ser-L[p]	98	h	1856	SERt2rpp	0
1193	ser-L[p]	604	ser-L	1856	SERt2rpp	1
941	na1[p]	604	ser-L	1857	SERt4pp	0
941	na1[p]	1344	na1	1857	SERt4pp	0
1193	ser-L[p]	604	ser-L	1857	SERt4pp	1
1193	ser-L[p]	1344	na1	1857	SERt4pp	0
989	ser-L[e]	1193	ser-L[p]	1858	SERtex	1
135	atp	177	amp	1859	SERTRS	1
135	atp	1192	ppi	1859	SERTRS	0
135	atp	1661	sertrna	1859	SERTRS	0
604	ser-L	177	amp	1859	SERTRS	0
604	ser-L	1192	ppi	1859	SERTRS	0
604	ser-L	1661	sertrna	1859	SERTRS	1
1647	trnaser	177	amp	1859	SERTRS	0
1647	trnaser	1192	ppi	1859	SERTRS	0
1647	trnaser	1661	sertrna	1859	SERTRS	1
135	atp	177	amp	1860	SERTRS2	1
135	atp	1192	ppi	1860	SERTRS2	0
135	atp	1519	sertrna(sec)	1860	SERTRS2	0
604	ser-L	177	amp	1860	SERTRS2	0
604	ser-L	1192	ppi	1860	SERTRS2	0
604	ser-L	1519	sertrna(sec)	1860	SERTRS2	1
1648	trnasecys	177	amp	1860	SERTRS2	0

1648	trnasecys	1192	ppi	1860	SERTRS2	0
1648	trnasecys	1519	sertrna(sec)	1860	SERTRS2	1
109	h2o	98	h	1861	SFGTHi	0
109	h2o	536	for	1861	SFGTHi	0
109	h2o	751	gthrd	1861	SFGTHi	0
990	Sfglutth	98	h	1861	SFGTHi	0
990	Sfglutth	536	for	1861	SFGTHi	1
990	Sfglutth	751	gthrd	1861	SFGTHi	1
109	h2o	624	glu-L	1862	SGDS	0
109	h2o	1008	succ	1862	SGDS	0
1520	sucglu	624	glu-L	1862	SGDS	1
1520	sucglu	1008	succ	1862	SGDS	1
109	h2o	98	h	1863	SGSAD	0
109	h2o	870	nadh	1863	SGSAD	0
109	h2o	1520	sucglu	1863	SGSAD	0
856	nad	98	h	1863	SGSAD	0
856	nad	870	nadh	1863	SGSAD	1
856	nad	1520	sucglu	1863	SGSAD	0
1611	sucgsa	98	h	1863	SGSAD	0
1611	sucgsa	870	nadh	1863	SGSAD	0
1611	sucgsa	1520	sucglu	1863	SGSAD	1
991	ichor	1001	2shchc	1864	SHCHCS2	1
991	ichor	1148	pyr	1864	SHCHCS2	0
991	ichor	1528	thmpp	1864	SHCHCS2	0
1521	ssaltpp	1001	2shchc	1864	SHCHCS2	1
1521	ssaltpp	1148	pyr	1864	SHCHCS2	1
1521	ssaltpp	1528	thmpp	1864	SHCHCS2	1
856	nad	98	h	1865	SHCHD2	0
856	nad	870	nadh	1865	SHCHD2	1
856	nad	1522	scl	1865	SHCHD2	0
992	dscl	98	h	1865	SHCHD2	0
992	dscl	870	nadh	1865	SHCHD2	0
992	dscl	1522	scl	1865	SHCHD2	1
509	fe2	98	h	1866	SHCHF	0
509	fe2	1610	sheme	1866	SHCHF	0
1522	scl	98	h	1866	SHCHF	0
1522	scl	1610	sheme	1866	SHCHF	1
98	h	459	nadp	1867	SHK3Dr	0
98	h	1523	skm	1867	SHK3Dr	0
871	nadph	459	nadp	1867	SHK3Dr	1
871	nadph	1523	skm	1867	SHK3Dr	0
993	3dhsk	459	nadp	1867	SHK3Dr	0
993	3dhsk	1523	skm	1867	SHK3Dr	1
135	atp	98	h	1868	SHKK	0
135	atp	281	adp	1868	SHKK	1
135	atp	1499	skm5p	1868	SHKK	0
1523	skm	98	h	1868	SHKK	0
1523	skm	281	adp	1868	SHKK	0
1523	skm	1499	skm5p	1868	SHKK	1
366	cys-L	98	h	1869	SHSL1	0
366	cys-L	370	cyst-L	1869	SHSL1	1
366	cys-L	1008	succ	1869	SHSL1	0
1524	suchms	98	h	1869	SHSL1	0
1524	suchms	370	cyst-L	1869	SHSL1	1
1524	suchms	1008	succ	1869	SHSL1	1
437	h[p]	98	h	1870	SKMt2pp	0

437	h[p]	1523	skm	1870	SKMt2pp	0
1194	skm[p]	98	h	1870	SKMt2pp	0
1194	skm[p]	1523	skm	1870	SKMt2pp	1
994	skm[e]	1194	skm[p]	1871	SKMtex	1
995	so2[e]	996	so2[p]	1872	SO2tex	0
996	so2[p]	1195	so2	1873	SO2tpp	0
997	so3[e]	1196	so3[p]	1874	SO3tex	0
998	so4[e]	1197	so4[p]	1875	SO4tex	0
213	akg	624	glu-L	1876	SOTA	1
213	akg	1611	sucgsa	1876	SOTA	0
1525	sucorn	624	glu-L	1876	SOTA	0
1525	sucorn	1611	sucgsa	1876	SOTA	1
109	h2o	98	h	1877	SPMDabcpp	0
109	h2o	281	adp	1877	SPMDabcpp	0
109	h2o	962	pi	1877	SPMDabcpp	0
109	h2o	1526	spmd	1877	SPMDabcpp	0
135	atp	98	h	1877	SPMDabcpp	0
135	atp	281	adp	1877	SPMDabcpp	1
135	atp	962	pi	1877	SPMDabcpp	0
135	atp	1526	spmd	1877	SPMDabcpp	0
1198	spmd[p]	98	h	1877	SPMDabcpp	0
1198	spmd[p]	281	adp	1877	SPMDabcpp	0
1198	spmd[p]	962	pi	1877	SPMDabcpp	0
1198	spmd[p]	1526	spmd	1877	SPMDabcpp	1
128	accoa	98	h	1878	SPMDAT1	0
128	accoa	927	coa	1878	SPMDAT1	1
128	accoa	1353	N1aspmd	1878	SPMDAT1	1
1526	spmd	98	h	1878	SPMDAT1	0
1526	spmd	927	coa	1878	SPMDAT1	0
1526	spmd	1353	N1aspmd	1878	SPMDAT1	1
128	accoa	98	h	1879	SPMDAT2	0
128	accoa	927	coa	1879	SPMDAT2	1
128	accoa	1662	n8aspmd	1879	SPMDAT2	1
1526	spmd	98	h	1879	SPMDAT2	0
1526	spmd	927	coa	1879	SPMDAT2	0
1526	spmd	1662	n8aspmd	1879	SPMDAT2	1
999	spmd[e]	1198	spmd[p]	1880	SPMDtex	1
1000	ametam	98	h	1881	SPMS	0
1000	ametam	847	5mta	1881	SPMS	1
1000	ametam	1526	spmd	1881	SPMS	1
1245	ptrc	98	h	1881	SPMS	0
1245	ptrc	847	5mta	1881	SPMS	0
1245	ptrc	1526	spmd	1881	SPMS	1
98	h	292	h2o2	1882	SPODM	0
98	h	928	o2	1882	SPODM	0
1527	o2s	292	h2o2	1882	SPODM	0
1527	o2s	928	o2	1882	SPODM	0
437	h[p]	903	o2[p]	1883	SPODMpp	0
437	h[p]	1122	h2o2[p]	1883	SPODMpp	0
1164	o2s[p]	903	o2[p]	1883	SPODMpp	0
1164	o2s[p]	1122	h2o2[p]	1883	SPODMpp	0
109	h2o	98	h	1884	SSALx	0
109	h2o	870	nadh	1884	SSALx	0
109	h2o	1008	succ	1884	SSALx	0
856	nad	98	h	1884	SSALx	0
856	nad	870	nadh	1884	SSALx	1

856	nad	1008	succ	1884	SSALx	0
1581	sucsal	98	h	1884	SSALx	0
1581	sucsal	870	nadh	1884	SSALx	0
1581	sucsal	1008	succ	1884	SSALx	1
109	h2o	98	h	1885	SSALy	0
109	h2o	871	nadph	1885	SSALy	0
109	h2o	1008	succ	1885	SSALy	0
459	nadp	98	h	1885	SSALy	0
459	nadp	871	nadph	1885	SSALy	1
459	nadp	1008	succ	1885	SSALy	0
1581	sucsal	98	h	1885	SSALy	0
1581	sucsal	871	nadph	1885	SSALy	0
1581	sucsal	1008	succ	1885	SSALy	1
135	atp	177	amp	1886	SUCBZL	1
135	atp	874	sbzcoa	1886	SUCBZL	0
135	atp	1192	ppi	1886	SUCBZL	0
927	coa	177	amp	1886	SUCBZL	0
927	coa	874	sbzcoa	1886	SUCBZL	1
927	coa	1192	ppi	1886	SUCBZL	0
1612	sucbz	177	amp	1886	SUCBZL	0
1612	sucbz	874	sbzcoa	1886	SUCBZL	1
1612	sucbz	1192	ppi	1886	SUCBZL	0
1001	2shchc	109	h2o	1887	SUCBZS	0
1001	2shchc	1612	sucbz	1887	SUCBZS	1
437	h[p]	98	h	1888	SUCCt2_2pp	0
437	h[p]	1008	succ	1888	SUCCt2_2pp	0
1199	succ[p]	98	h	1888	SUCCt2_2pp	0
1199	succ[p]	1008	succ	1888	SUCCt2_2pp	1
437	h[p]	98	h	1889	SUCCt2_3pp	0
437	h[p]	1008	succ	1889	SUCCt2_3pp	0
1199	succ[p]	98	h	1889	SUCCt2_3pp	0
1199	succ[p]	1008	succ	1889	SUCCt2_3pp	1
437	h[p]	98	h	1890	SUCCt3pp	0
437	h[p]	1199	succ[p]	1890	SUCCt3pp	0
1008	succ	98	h	1890	SUCCt3pp	0
1008	succ	1199	succ[p]	1890	SUCCt3pp	1
1002	succ[e]	1199	succ[p]	1891	SUCCtex	1
1003	q8	540	fum	1892	SUCDi	0
1003	q8	1443	q8h2	1892	SUCDi	1
1008	succ	540	fum	1892	SUCDi	1
1008	succ	1443	q8h2	1892	SUCDi	0
551	fum[p]	540	fum	1893	SUCFUMtpp	1
551	fum[p]	1199	succ[p]	1893	SUCFUMtpp	0
1008	succ	540	fum	1893	SUCFUMtpp	0
1008	succ	1199	succ[p]	1893	SUCFUMtpp	1
135	atp	281	adp	1894	SUCOAS	1
135	atp	838	succoa	1894	SUCOAS	0
135	atp	962	pi	1894	SUCOAS	0
927	coa	281	adp	1894	SUCOAS	0
927	coa	838	succoa	1894	SUCOAS	1
927	coa	962	pi	1894	SUCOAS	0
1008	succ	281	adp	1894	SUCOAS	0
1008	succ	838	succoa	1894	SUCOAS	1
1008	succ	962	pi	1894	SUCOAS	0
959	pep	1148	pyr	1895	SUCptspp	1
959	pep	1399	suc6p	1895	SUCptspp	0

1200	sucr[p]	1148	pyr	1895	SUCptspp	0
1200	sucr[p]	1399	suc6p	1895	SUCptspp	1
1004	sucr[e]	1200	sucr[p]	1896	SUCRtex	1
109	h2o	98	h	1897	SULabcpp	0
109	h2o	281	adp	1897	SULabcpp	0
109	h2o	962	pi	1897	SULabcpp	0
109	h2o	1600	so4	1897	SULabcpp	0
135	atp	98	h	1897	SULabcpp	0
135	atp	281	adp	1897	SULabcpp	1
135	atp	962	pi	1897	SULabcpp	0
135	atp	1600	so4	1897	SULabcpp	0
1197	so4[p]	98	h	1897	SULabcpp	0
1197	so4[p]	281	adp	1897	SULabcpp	0
1197	so4[p]	962	pi	1897	SULabcpp	0
1197	so4[p]	1600	so4	1897	SULabcpp	0
109	h2o	98	h	1898	SULFACabcpp	0
109	h2o	281	adp	1898	SULFACabcpp	0
109	h2o	962	pi	1898	SULFACabcpp	0
109	h2o	1630	sulfac	1898	SULFACabcpp	0
135	atp	98	h	1898	SULFACabcpp	0
135	atp	281	adp	1898	SULFACabcpp	1
135	atp	962	pi	1898	SULFACabcpp	0
135	atp	1630	sulfac	1898	SULFACabcpp	0
1201	sulfac[p]	98	h	1898	SULFACabcpp	0
1201	sulfac[p]	281	adp	1898	SULFACabcpp	0
1201	sulfac[p]	962	pi	1898	SULFACabcpp	0
1201	sulfac[p]	1630	sulfac	1898	SULFACabcpp	1
1005	sulfac[e]	1201	sulfac[p]	1899	SULFACtex	1
98	h	109	h2o	1900	SULRi	0
98	h	459	nadp	1900	SULRi	0
98	h	685	h2s	1900	SULRi	0
871	nadph	109	h2o	1900	SULRi	0
871	nadph	459	nadp	1900	SULRi	1
871	nadph	685	h2s	1900	SULRi	0
1590	so3	109	h2o	1900	SULRi	0
1590	so3	459	nadp	1900	SULRi	0
1590	so3	685	h2s	1900	SULRi	0
1006	tdec2eACP	100	cdec3eACP	1901	T2DECAI	1
238	altrn	98	h	1902	TAGURr	0
238	altrn	870	nadh	1902	TAGURr	0
238	altrn	1121	tagur	1902	TAGURr	1
856	nad	98	h	1902	TAGURr	0
856	nad	870	nadh	1902	TAGURr	1
856	nad	1121	tagur	1902	TAGURr	0
468	g3p	405	e4p	1903	TALA	1
468	g3p	499	f6p	1903	TALA	0
985	s7p	405	e4p	1903	TALA	1
985	s7p	499	f6p	1903	TALA	1
1007	tartr-L	109	h2o	1904	TARTD	0
1007	tartr-L	1318	oaa	1904	TARTD	1
1008	succ	1007	tartr-L	1905	TARTRt7pp	0
1008	succ	1199	succ[p]	1905	TARTRt7pp	1
1202	tartr-L[p]	1007	tartr-L	1905	TARTRt7pp	1
1202	tartr-L[p]	1199	succ[p]	1905	TARTRt7pp	0
1009	tartr-L[e]	1202	tartr-L[p]	1906	TARTRtex	1
213	akg	98	h	1907	TAUDO	0

213	akg	441	aacald	1907	TAUDO	0
213	akg	692	co2	1907	TAUDO	0
213	akg	1008	succ	1907	TAUDO	1
213	akg	1590	so3	1907	TAUDO	0
928	o2	98	h	1907	TAUDO	0
928	o2	441	aacald	1907	TAUDO	0
928	o2	692	co2	1907	TAUDO	0
928	o2	1008	succ	1907	TAUDO	0
928	o2	1590	so3	1907	TAUDO	0
1649	taur	98	h	1907	TAUDO	0
1649	taur	441	aacald	1907	TAUDO	1
1649	taur	692	co2	1907	TAUDO	0
1649	taur	1008	succ	1907	TAUDO	0
1649	taur	1590	so3	1907	TAUDO	0
109	h2o	98	h	1908	TAURabcpp	0
109	h2o	281	adp	1908	TAURabcpp	0
109	h2o	962	pi	1908	TAURabcpp	0
109	h2o	1649	taur	1908	TAURabcpp	0
135	atp	98	h	1908	TAURabcpp	0
135	atp	281	adp	1908	TAURabcpp	1
135	atp	962	pi	1908	TAURabcpp	0
135	atp	1649	taur	1908	TAURabcpp	0
1203	taur[p]	98	h	1908	TAURabcpp	0
1203	taur[p]	281	adp	1908	TAURabcpp	0
1203	taur[p]	962	pi	1908	TAURabcpp	0
1203	taur[p]	1649	taur	1908	TAURabcpp	1
1010	taur[e]	1203	taur[p]	1909	TAURtex	1
1011	tcynt[e]	1204	tcynt[p]	1910	TCYNTtex	1
1012	tdecoa	1354	td2coa	1911	TDECOAI	1
109	h2o	98	h	1912	TDP	0
109	h2o	962	pi	1912	TDP	0
109	h2o	1535	thmmp	1912	TDP	0
1528	thmpp	98	h	1912	TDP	0
1528	thmpp	962	pi	1912	TDP	0
1528	thmpp	1535	thmmp	1912	TDP	1
128	accoa	98	h	1913	TDPADGAT	0
128	accoa	120	dtdp4aaddg	1913	TDPADGAT	1
128	accoa	927	coa	1913	TDPADGAT	1
1529	dtdp4addg	98	h	1913	TDPADGAT	0
1529	dtdp4addg	120	dtdp4aaddg	1913	TDPADGAT	1
1529	dtdp4addg	927	coa	1913	TDPADGAT	0
624	glu-L	213	akg	1914	TDPAGTA	1
624	glu-L	1529	dtdp4addg	1914	TDPAGTA	0
1013	dtdp4d6dg	213	akg	1914	TDPAGTA	0
1013	dtdp4d6dg	1529	dtdp4addg	1914	TDPAGTA	1
1013	dtdp4d6dg	1014	dtdp4d6dm	1915	TDPDRE	1
98	h	459	nadp	1916	TDPDRR	0
98	h	974	dtdprmn	1916	TDPDRR	0
871	nadph	459	nadp	1916	TDPDRR	1
871	nadph	974	dtdprmn	1916	TDPDRR	0
1014	dtdp4d6dm	459	nadp	1916	TDPDRR	0
1014	dtdp4d6dm	974	dtdprmn	1916	TDPDRR	1
1015	dtdpglu	109	h2o	1917	TDPGDH	0
1015	dtdpglu	1013	dtdp4d6dg	1917	TDPGDH	1
135	atp	98	h	1918	TDSK	0
135	atp	281	adp	1918	TDSK	1

135	atp	1437	lipidA	1918	TDSK	0
1530	lipidAds	98	h	1918	TDSK	0
1530	lipidAds	281	adp	1918	TDSK	0
1530	lipidAds	1437	lipidA	1918	TDSK	1
456	dsbcox[p]	457	dsbdox	1919	TDSR1	0
456	dsbcox[p]	1265	dsbcrd[p]	1919	TDSR1	1
1016	dsbdrd	457	dsbdox	1919	TDSR1	1
1016	dsbdrd	1265	dsbcrd[p]	1919	TDSR1	0
458	dsbgox[p]	457	dsbdox	1920	TDSR2	0
458	dsbgox[p]	1266	dsbgrd[p]	1920	TDSR2	1
1016	dsbdrd	457	dsbdox	1920	TDSR2	1
1016	dsbdrd	1266	dsbgrd[p]	1920	TDSR2	0
1017	tagdp-D	468	g3p	1921	TGBPA	1
1017	tagdp-D	826	dhap	1921	TGBPA	1
437	h[p]	98	h	1922	THD2pp	0
437	h[p]	856	nad	1922	THD2pp	0
437	h[p]	871	nadph	1922	THD2pp	0
459	nadp	98	h	1922	THD2pp	0
459	nadp	856	nad	1922	THD2pp	0
459	nadp	871	nadph	1922	THD2pp	1
870	nadh	98	h	1922	THD2pp	0
870	nadh	856	nad	1922	THD2pp	1
870	nadh	871	nadph	1922	THD2pp	0
109	h2o	927	coa	1923	THDPS	0
109	h2o	1564	sl2a6o	1923	THDPS	0
838	succoa	927	coa	1923	THDPS	1
838	succoa	1564	sl2a6o	1923	THDPS	1
1592	thdp	927	coa	1923	THDPS	0
1592	thdp	1564	sl2a6o	1923	THDPS	1
292	h2o2	109	h2o	1924	THIORDXi	0
292	h2o2	1594	trdox	1924	THIORDXi	0
981	trdrd	109	h2o	1924	THIORDXi	0
981	trdrd	1594	trdox	1924	THIORDXi	1
109	h2o	98	h	1925	THMabcpp	0
109	h2o	281	adp	1925	THMabcpp	0
109	h2o	962	pi	1925	THMabcpp	0
109	h2o	1534	thm	1925	THMabcpp	0
135	atp	98	h	1925	THMabcpp	0
135	atp	281	adp	1925	THMabcpp	1
135	atp	962	pi	1925	THMabcpp	0
135	atp	1534	thm	1925	THMabcpp	0
1206	thm[p]	98	h	1925	THMabcpp	0
1206	thm[p]	281	adp	1925	THMabcpp	0
1206	thm[p]	962	pi	1925	THMabcpp	0
1206	thm[p]	1534	thm	1925	THMabcpp	1
437	h[p]	98	h	1926	THMDt2pp	0
437	h[p]	1533	thymd	1926	THMDt2pp	0
1205	thymd[p]	98	h	1926	THMDt2pp	0
1205	thymd[p]	1533	thymd	1926	THMDt2pp	1
437	h[p]	98	h	1927	THMDt2rpp	0
437	h[p]	1533	thymd	1927	THMDt2rpp	0
1205	thymd[p]	98	h	1927	THMDt2rpp	0
1205	thymd[p]	1533	thymd	1927	THMDt2rpp	1
1018	thymd[e]	1205	thymd[p]	1928	THMDtex	1
1019	thm[e]	1206	thm[p]	1929	THMtex	1
280	athr-L	131	acald	1930	THRA2i	1

280	athr-L	642	gly	1930	THRA2i	1
109	h2o	98	h	1931	THRabcpp	0
109	h2o	281	adp	1931	THRabcpp	0
109	h2o	962	pi	1931	THRabcpp	0
109	h2o	1020	thr-L	1931	THRabcpp	0
135	atp	98	h	1931	THRabcpp	0
135	atp	281	adp	1931	THRabcpp	1
135	atp	962	pi	1931	THRabcpp	0
135	atp	1020	thr-L	1931	THRabcpp	0
1208	thr-L[p]	98	h	1931	THRabcpp	0
1208	thr-L[p]	281	adp	1931	THRabcpp	0
1208	thr-L[p]	962	pi	1931	THRabcpp	0
1208	thr-L[p]	1020	thr-L	1931	THRabcpp	1
1020	thr-L	131	acald	1932	THRAi	1
1020	thr-L	642	gly	1932	THRAi	1
856	nad	98	h	1933	THRD	0
856	nad	251	2aobut	1933	THRD	0
856	nad	870	nadh	1933	THRD	1
1020	thr-L	98	h	1933	THRD	0
1020	thr-L	251	2aobut	1933	THRD	1
1020	thr-L	870	nadh	1933	THRD	0
1020	thr-L	146	2obut	1934	THRD_L	1
1020	thr-L	1160	nh4	1934	THRD_L	0
1021	thrp[e]	1207	thrp[p]	1935	THRPtex	1
109	h2o	962	pi	1936	THRS	0
109	h2o	1020	thr-L	1936	THRS	0
1531	phom	962	pi	1936	THRS	0
1531	phom	1020	thr-L	1936	THRS	1
437	h[p]	98	h	1937	THRt2pp	0
437	h[p]	1208	thr-L[p]	1937	THRt2pp	0
1020	thr-L	98	h	1937	THRt2pp	0
1020	thr-L	1208	thr-L[p]	1937	THRt2pp	1
437	h[p]	98	h	1938	THRt2rpp	0
437	h[p]	1020	thr-L	1938	THRt2rpp	0
1208	thr-L[p]	98	h	1938	THRt2rpp	0
1208	thr-L[p]	1020	thr-L	1938	THRt2rpp	1
941	na1[p]	1020	thr-L	1939	THRt4pp	0
941	na1[p]	1344	na1	1939	THRt4pp	0
1208	thr-L[p]	1020	thr-L	1939	THRt4pp	1
1208	thr-L[p]	1344	na1	1939	THRt4pp	0
1022	thr-L[e]	1208	thr-L[p]	1940	THRtex	1
135	atp	177	amp	1941	THRTRS	1
135	atp	1192	ppi	1941	THRTRS	0
135	atp	1663	thrtrna	1941	THRTRS	0
1020	thr-L	177	amp	1941	THRTRS	0
1020	thr-L	1192	ppi	1941	THRTRS	0
1020	thr-L	1663	thrtrna	1941	THRTRS	1
1650	trnathr	177	amp	1941	THRTRS	0
1650	trnathr	1192	ppi	1941	THRTRS	0
1650	trnathr	1663	thrtrna	1941	THRTRS	1
437	h[p]	98	h	1942	THYMt3pp	0
437	h[p]	1209	thym[p]	1942	THYMt3pp	0
1532	thym	98	h	1942	THYMt3pp	0
1532	thym	1209	thym[p]	1942	THYMt3pp	1
1023	thym[e]	1209	thym[p]	1943	THYMtex	1
135	atp	177	amp	1944	THZPSN	1

135	atp	222	ala-L	1944	THZPSN	0
135	atp	439	4hba	1944	THZPSN	0
135	atp	692	co2	1944	THZPSN	0
135	atp	1293	4mpetz	1944	THZPSN	0
366	cys-L	177	amp	1944	THZPSN	0
366	cys-L	222	ala-L	1944	THZPSN	1
366	cys-L	439	4hba	1944	THZPSN	0
366	cys-L	692	co2	1944	THZPSN	0
366	cys-L	1293	4mpetz	1944	THZPSN	0
467	dxyl5p	177	amp	1944	THZPSN	0
467	dxyl5p	222	ala-L	1944	THZPSN	0
467	dxyl5p	439	4hba	1944	THZPSN	0
467	dxyl5p	692	co2	1944	THZPSN	0
467	dxyl5p	1293	4mpetz	1944	THZPSN	1
1540	tyr-L	177	amp	1944	THZPSN	0
1540	tyr-L	222	ala-L	1944	THZPSN	0
1540	tyr-L	439	4hba	1944	THZPSN	1
1540	tyr-L	692	co2	1944	THZPSN	0
1540	tyr-L	1293	4mpetz	1944	THZPSN	1
983	r5p	468	g3p	1945	TKT1	1
983	r5p	985	s7p	1945	TKT1	1
1187	xu5p-D	468	g3p	1945	TKT1	1
1187	xu5p-D	985	s7p	1945	TKT1	1
405	e4p	468	g3p	1946	TKT2	1
405	e4p	499	f6p	1946	TKT2	1
1187	xu5p-D	468	g3p	1946	TKT2	1
1187	xu5p-D	499	f6p	1946	TKT2	1
98	h	109	h2o	1947	TMAOR1	0
98	h	1282	mqn8	1947	TMAOR1	0
98	h	1664	tma	1947	TMAOR1	0
867	mql8	109	h2o	1947	TMAOR1	0
867	mql8	1282	mqn8	1947	TMAOR1	1
867	mql8	1664	tma	1947	TMAOR1	0
1651	tmao	109	h2o	1947	TMAOR1	0
1651	tmao	1282	mqn8	1947	TMAOR1	0
1651	tmao	1664	tma	1947	TMAOR1	1
437	h[p]	684	h2o[p]	1948	TMAOR1pp	0
437	h[p]	1211	tma[p]	1948	TMAOR1pp	0
437	h[p]	1282	mqn8	1948	TMAOR1pp	0
867	mql8	684	h2o[p]	1948	TMAOR1pp	0
867	mql8	1211	tma[p]	1948	TMAOR1pp	0
867	mql8	1282	mqn8	1948	TMAOR1pp	1
1210	tmao[p]	684	h2o[p]	1948	TMAOR1pp	0
1210	tmao[p]	1211	tma[p]	1948	TMAOR1pp	1
1210	tmao[p]	1282	mqn8	1948	TMAOR1pp	0
98	h	109	h2o	1949	TMAOR2	0
98	h	560	2dmmq8	1949	TMAOR2	0
98	h	1664	tma	1949	TMAOR2	0
245	2dmmql8	109	h2o	1949	TMAOR2	0
245	2dmmql8	560	2dmmq8	1949	TMAOR2	1
245	2dmmql8	1664	tma	1949	TMAOR2	0
1651	tmao	109	h2o	1949	TMAOR2	0
1651	tmao	560	2dmmq8	1949	TMAOR2	0
1651	tmao	1664	tma	1949	TMAOR2	1
245	2dmmql8	560	2dmmq8	1950	TMAOR2pp	1
245	2dmmql8	684	h2o[p]	1950	TMAOR2pp	0

245	2dmmql8	1211	tma[p]	1950	TMAOR2pp	0
437	h[p]	560	2dmmq8	1950	TMAOR2pp	0
437	h[p]	684	h2o[p]	1950	TMAOR2pp	0
437	h[p]	1211	tma[p]	1950	TMAOR2pp	0
1210	tmao[p]	560	2dmmq8	1950	TMAOR2pp	0
1210	tmao[p]	684	h2o[p]	1950	TMAOR2pp	0
1210	tmao[p]	1211	tma[p]	1950	TMAOR2pp	1
1024	tmao[e]	1210	tmao[p]	1951	TMAOtex	1
1025	tma[e]	1211	tma[p]	1952	TMAtex	1
135	atp	98	h	1953	TMDK1	0
135	atp	281	adp	1953	TMDK1	1
135	atp	882	dtmp	1953	TMDK1	0
1533	thymd	98	h	1953	TMDK1	0
1533	thymd	281	adp	1953	TMDK1	0
1533	thymd	882	dtmp	1953	TMDK1	1
962	pi	950	2dr1p	1954	TMDPP	0
962	pi	1532	thym	1954	TMDPP	0
1533	thymd	950	2dr1p	1954	TMDPP	1
1533	thymd	1532	thym	1954	TMDPP	1
848	mlthf	421	dhf	1955	TMDS	1
848	mlthf	882	dtmp	1955	TMDS	1
875	dump	421	dhf	1955	TMDS	0
875	dump	882	dtmp	1955	TMDS	1
135	atp	98	h	1956	TMK	0
135	atp	281	adp	1956	TMK	1
135	atp	1535	thmmp	1956	TMK	0
1534	thm	98	h	1956	TMK	0
1534	thm	281	adp	1956	TMK	0
1534	thm	1535	thmmp	1956	TMK	1
135	atp	281	adp	1957	TMPK	1
135	atp	1528	thmpp	1957	TMPK	0
1535	thmmp	281	adp	1957	TMPK	0
1535	thmmp	1528	thmpp	1957	TMPK	1
62	2mahmp	1192	ppi	1958	TMPPP	0
62	2mahmp	1535	thmmp	1958	TMPPP	1
98	h	1192	ppi	1958	TMPPP	0
98	h	1535	thmmp	1958	TMPPP	0
1293	4mpetz	1192	ppi	1958	TMPPP	0
1293	4mpetz	1535	thmmp	1958	TMPPP	1
826	dhap	468	g3p	1959	TPI	1
135	atp	174	ade	1960	TPRDCOAS	1
135	atp	1355	2tpr3dpcoa	1960	TPRDCOAS	1
1347	dpcoa	174	ade	1960	TPRDCOAS	0
1347	dpcoa	1355	2tpr3dpcoa	1960	TPRDCOAS	1
98	h	459	nadp	1961	TRDR	0
98	h	981	trdrd	1961	TRDR	0
871	nadph	459	nadp	1961	TRDR	1
871	nadph	981	trdrd	1961	TRDR	0
1594	trdox	459	nadp	1961	TRDR	0
1594	trdox	981	trdrd	1961	TRDR	1
109	h2o	568	g6p	1962	TRE6PH	0
109	h2o	1064	glc-D	1962	TRE6PH	0
1536	tre6p	568	g6p	1962	TRE6PH	1
1536	tre6p	1064	glc-D	1962	TRE6PH	1
109	h2o	962	pi	1963	TRE6PP	0
109	h2o	1537	tre	1963	TRE6PP	0

1536	tre6p	962	pi	1963	TRE6PP	0
1536	tre6p	1537	tre	1963	TRE6PP	1
568	g6p	98	h	1964	TRE6PS	0
568	g6p	1440	udp	1964	TRE6PS	0
568	g6p	1536	tre6p	1964	TRE6PS	1
1042	udpg	98	h	1964	TRE6PS	0
1042	udpg	1440	udp	1964	TRE6PS	1
1042	udpg	1536	tre6p	1964	TRE6PS	1
109	h2o	1064	glc-D	1965	TREH	0
1537	tre	1064	glc-D	1965	TREH	1
684	h2o[p]	606	glc-D[p]	1966	TREHpp	0
1212	tre[p]	606	glc-D[p]	1966	TREHpp	1
959	pep	1148	pyr	1967	TREptspp	1
959	pep	1536	tre6p	1967	TREptspp	0
1212	tre[p]	1148	pyr	1967	TREptspp	0
1212	tre[p]	1536	tre6p	1967	TREptspp	1
1026	tre[e]	1212	tre[p]	1968	TREtex	1
109	h2o	1028	indole	1969	TRPAS2	0
109	h2o	1148	pyr	1969	TRPAS2	0
109	h2o	1160	nh4	1969	TRPAS2	0
1538	trp-L	1028	indole	1969	TRPAS2	1
1538	trp-L	1148	pyr	1969	TRPAS2	1
1538	trp-L	1160	nh4	1969	TRPAS2	0
604	ser-L	109	h2o	1970	TRPS1	0
604	ser-L	468	g3p	1970	TRPS1	1
604	ser-L	1538	trp-L	1970	TRPS1	0
1027	3ig3p	109	h2o	1970	TRPS1	0
1027	3ig3p	468	g3p	1970	TRPS1	0
1027	3ig3p	1538	trp-L	1970	TRPS1	1
604	ser-L	109	h2o	1971	TRPS2	0
604	ser-L	1538	trp-L	1971	TRPS2	1
1028	indole	109	h2o	1971	TRPS2	0
1028	indole	1538	trp-L	1971	TRPS2	1
1027	3ig3p	468	g3p	1972	TRPS3	1
1027	3ig3p	1028	indole	1972	TRPS3	1
437	h[p]	98	h	1973	TRPt2rpp	0
437	h[p]	1538	trp-L	1973	TRPt2rpp	0
1213	trp-L[p]	98	h	1973	TRPt2rpp	0
1213	trp-L[p]	1538	trp-L	1973	TRPt2rpp	1
1029	trp-L[e]	1213	trp-L[p]	1974	TRPtex	1
135	atp	177	amp	1975	TRPTRS	1
135	atp	1192	ppi	1975	TRPTRS	0
135	atp	1665	trptrna	1975	TRPTRS	0
1538	trp-L	177	amp	1975	TRPTRS	0
1538	trp-L	1192	ppi	1975	TRPTRS	0
1538	trp-L	1665	trptrna	1975	TRPTRS	1
1539	trnatrp	177	amp	1975	TRPTRS	0
1539	trnatrp	1192	ppi	1975	TRPTRS	0
1539	trnatrp	1665	trptrna	1975	TRPTRS	1
98	h	856	nad	1976	TRSARr	0
98	h	1118	glyc-R	1976	TRSARr	0
870	nadh	856	nad	1976	TRSARr	1
870	nadh	1118	glyc-R	1976	TRSARr	0
1030	2h3oppan	856	nad	1976	TRSARr	0
1030	2h3oppan	1118	glyc-R	1976	TRSARr	1
109	h2o	98	h	1977	TSULabcpp	0

109	h2o	281	adp	1977	TSULabcpp	0
109	h2o	962	pi	1977	TSULabcpp	0
109	h2o	1391	tsul	1977	TSULabcpp	0
135	atp	98	h	1977	TSULabcpp	0
135	atp	281	adp	1977	TSULabcpp	1
135	atp	962	pi	1977	TSULabcpp	0
135	atp	1391	tsul	1977	TSULabcpp	0
1214	tsul[p]	98	h	1977	TSULabcpp	0
1214	tsul[p]	281	adp	1977	TSULabcpp	0
1214	tsul[p]	962	pi	1977	TSULabcpp	0
1214	tsul[p]	1391	tsul	1977	TSULabcpp	1
1031	tsul[e]	1214	tsul[p]	1978	TSULtex	1
1032	ttdca[e]	1356	ttdca[p]	1979	TTDCAtexi	1
1033	ttdcea[e]	1357	ttdcea[p]	1980	TTDCEAtexi	1
109	h2o	98	h	1981	TUNGSabcpp	0
109	h2o	281	adp	1981	TUNGSabcpp	0
109	h2o	962	pi	1981	TUNGSabcpp	0
109	h2o	1668	tungs	1981	TUNGSabcpp	0
135	atp	98	h	1981	TUNGSabcpp	0
135	atp	281	adp	1981	TUNGSabcpp	1
135	atp	962	pi	1981	TUNGSabcpp	0
135	atp	1668	tungs	1981	TUNGSabcpp	0
1215	tungs[p]	98	h	1981	TUNGSabcpp	0
1215	tungs[p]	281	adp	1981	TUNGSabcpp	0
1215	tungs[p]	962	pi	1981	TUNGSabcpp	0
1215	tungs[p]	1668	tungs	1981	TUNGSabcpp	1
1034	tungs[e]	1215	tungs[p]	1982	TUNGStex	1
1035	tym[e]	1216	tym[p]	1983	TYMtex	1
684	h2o[p]	859	nh4[p]	1984	TYROXDApp	0
684	h2o[p]	1072	4hoxpacd[p]	1984	TYROXDApp	0
684	h2o[p]	1122	h2o2[p]	1984	TYROXDApp	0
903	o2[p]	859	nh4[p]	1984	TYROXDApp	0
903	o2[p]	1072	4hoxpacd[p]	1984	TYROXDApp	0
903	o2[p]	1122	h2o2[p]	1984	TYROXDApp	0
1216	tym[p]	859	nh4[p]	1984	TYROXDApp	0
1216	tym[p]	1072	4hoxpacd[p]	1984	TYROXDApp	1
1216	tym[p]	1122	h2o2[p]	1984	TYROXDApp	0
684	h2o[p]	1173	pi[p]	1985	TYRPpp	0
684	h2o[p]	1219	tyr-L[p]	1985	TYRPpp	0
1217	tyrp[p]	1173	pi[p]	1985	TYRPpp	0
1217	tyrp[p]	1219	tyr-L[p]	1985	TYRPpp	1
1036	tyrp[e]	1217	tyrp[p]	1986	TYRPtex	1
437	h[p]	98	h	1987	TYRt2rpp	0
437	h[p]	1540	tyr-L	1987	TYRt2rpp	0
1219	tyr-L[p]	98	h	1987	TYRt2rpp	0
1219	tyr-L[p]	1540	tyr-L	1987	TYRt2rpp	1
213	akg	624	glu-L	1988	TYRTA	1
213	akg	1218	34hpp	1988	TYRTA	0
1540	tyr-L	624	glu-L	1988	TYRTA	0
1540	tyr-L	1218	34hpp	1988	TYRTA	1
1037	tyr-L[e]	1219	tyr-L[p]	1989	TYRtex	1
135	atp	177	amp	1990	TYRTRS	1
135	atp	1192	ppi	1990	TYRTRS	0
135	atp	1666	tyrtrna	1990	TYRTRS	0
1540	tyr-L	177	amp	1990	TYRTRS	0
1540	tyr-L	1192	ppi	1990	TYRTRS	0

1540	tyr-L	1666	tyrtrna	1990	TYRTRS	1
1541	trnatyr	177	amp	1990	TYRTRS	0
1541	trnatyr	1192	ppi	1990	TYRTRS	0
1541	trnatyr	1666	tyrtrna	1990	TYRTRS	1
70	3hmrsACP	98	h	1991	U23GAAT	0
70	3hmrsACP	118	ACP	1991	U23GAAT	1
70	3hmrsACP	1424	u23ga	1991	U23GAAT	1
1542	u3hga	98	h	1991	U23GAAT	0
1542	u3hga	118	ACP	1991	U23GAAT	0
1542	u3hga	1424	u23ga	1991	U23GAAT	1
135	atp	98	h	1992	UAAGDS	0
135	atp	281	adp	1992	UAAGDS	1
135	atp	962	pi	1992	UAAGDS	0
135	atp	1615	ugmd	1992	UAAGDS	0
389	26dap-M	98	h	1992	UAAGDS	0
389	26dap-M	281	adp	1992	UAAGDS	0
389	26dap-M	962	pi	1992	UAAGDS	0
389	26dap-M	1615	ugmd	1992	UAAGDS	1
1652	uamag	98	h	1992	UAAGDS	0
1652	uamag	281	adp	1992	UAAGDS	0
1652	uamag	962	pi	1992	UAAGDS	0
1652	uamag	1615	ugmd	1992	UAAGDS	1
684	h2o[p]	136	acgal1p[p]	1993	UACGALPpp	0
684	h2o[p]	437	h[p]	1993	UACGALPpp	0
684	h2o[p]	1226	ump[p]	1993	UACGALPpp	0
1222	udpacgal[p]	136	acgal1p[p]	1993	UACGALPpp	1
1222	udpacgal[p]	437	h[p]	1993	UACGALPpp	0
1222	udpacgal[p]	1226	ump[p]	1993	UACGALPpp	1
684	h2o[p]	139	acgam1p[p]	1994	UACGAMPpp	0
684	h2o[p]	437	h[p]	1994	UACGAMPpp	0
684	h2o[p]	1226	ump[p]	1994	UACGAMPpp	0
1220	uacgam[p]	139	acgam1p[p]	1994	UACGAMPpp	1
1220	uacgam[p]	437	h[p]	1994	UACGAMPpp	0
1220	uacgam[p]	1226	ump[p]	1994	UACGAMPpp	1
1038	uacgam[e]	1220	uacgam[p]	1995	UACGAMtex	1
109	h2o	98	h	1996	UACMAMO	0
109	h2o	149	uacmamu	1996	UACMAMO	0
109	h2o	870	nadh	1996	UACMAMO	0
856	nad	98	h	1996	UACMAMO	0
856	nad	149	uacmamu	1996	UACMAMO	0
856	nad	870	nadh	1996	UACMAMO	1
1221	uacmam	98	h	1996	UACMAMO	0
1221	uacmam	149	uacmamu	1996	UACMAMO	1
1221	uacmam	870	nadh	1996	UACMAMO	0
142	uacgam	1221	uacmam	1997	UAG2E	1
70	3hmrsACP	118	ACP	1998	UAGAAT	1
70	3hmrsACP	1547	u3aga	1998	UAGAAT	1
142	uacgam	118	ACP	1998	UAGAAT	0
142	uacgam	1547	u3aga	1998	UAGAAT	1
142	uacgam	962	pi	1999	UAGCVT	0
142	uacgam	1613	uaccg	1999	UAGCVT	1
959	pep	962	pi	1999	UAGCVT	0
959	pep	1613	uaccg	1999	UAGCVT	1
98	h	142	uacgam	2000	UAGDP	0
98	h	1192	ppi	2000	UAGDP	0
1039	acgam1p	142	uacgam	2000	UAGDP	1

1039	acgam1p	1192	ppi	2000	UAGDP	0
1447	utp	142	uacgam	2000	UAGDP	1
1447	utp	1192	ppi	2000	UAGDP	0
142	uacgam	98	h	2001	UAGPT3	0
142	uacgam	845	uaagmda	2001	UAGPT3	1
142	uacgam	1440	udp	2001	UAGPT3	1
1335	uagmda	98	h	2001	UAGPT3	0
1335	uagmda	845	uaagmda	2001	UAGPT3	1
1335	uagmda	1440	udp	2001	UAGPT3	1
135	atp	98	h	2002	UAMAGS	0
135	atp	281	adp	2002	UAMAGS	1
135	atp	962	pi	2002	UAMAGS	0
135	atp	1652	uamag	2002	UAMAGS	0
627	glu-D	98	h	2002	UAMAGS	0
627	glu-D	281	adp	2002	UAMAGS	0
627	glu-D	962	pi	2002	UAMAGS	0
627	glu-D	1652	uamag	2002	UAMAGS	1
1653	uama	98	h	2002	UAMAGS	0
1653	uama	281	adp	2002	UAMAGS	0
1653	uama	962	pi	2002	UAMAGS	0
1653	uama	1652	uamag	2002	UAMAGS	1
135	atp	98	h	2003	UAMAS	0
135	atp	281	adp	2003	UAMAS	1
135	atp	962	pi	2003	UAMAS	0
135	atp	1653	uama	2003	UAMAS	0
222	ala-L	98	h	2003	UAMAS	0
222	ala-L	281	adp	2003	UAMAS	0
222	ala-L	962	pi	2003	UAMAS	0
222	ala-L	1653	uama	2003	UAMAS	1
1614	uamr	98	h	2003	UAMAS	0
1614	uamr	281	adp	2003	UAMAS	0
1614	uamr	962	pi	2003	UAMAS	0
1614	uamr	1653	uama	2003	UAMAS	1
98	h	459	nadp	2004	UAPGR	0
98	h	1614	uamr	2004	UAPGR	0
871	nadph	459	nadp	2004	UAPGR	1
871	nadph	1614	uamr	2004	UAPGR	0
1613	uaccg	459	nadp	2004	UAPGR	0
1613	uaccg	1614	uamr	2004	UAPGR	1
109	h2o	98	h	2005	UDCPDP	0
109	h2o	923	udcpp	2005	UDCPDP	0
109	h2o	962	pi	2005	UDCPDP	0
1543	udcpdp	98	h	2005	UDCPDP	0
1543	udcpdp	923	udcpp	2005	UDCPDP	1
1543	udcpdp	962	pi	2005	UDCPDP	0
684	h2o[p]	437	h[p]	2006	UDCPDPpp	0
684	h2o[p]	1040	udcpp[p]	2006	UDCPDPpp	0
684	h2o[p]	1173	pi[p]	2006	UDCPDPpp	0
1544	udcpdp[p]	437	h[p]	2006	UDCPDPpp	0
1544	udcpdp[p]	1040	udcpp[p]	2006	UDCPDPpp	1
1544	udcpdp[p]	1173	pi[p]	2006	UDCPDPpp	0
697	frdp	1192	ppi	2007	UDCPDPS	0
697	frdp	1543	udcpdp	2007	UDCPDPS	1
726	ipdp	1192	ppi	2007	UDCPDPS	0
726	ipdp	1543	udcpdp	2007	UDCPDPS	1
1040	udcpp[p]	923	udcpp	2008	UDCPPtppi	1

1041	udpacgal[e]	1222	udpacgal[p]	2009	UDPACGALtex	1
1042	udpg	1043	udpgal	2010	UDPG4E	1
1043	udpgal	1358	udpgalfur	2011	UDPGALM	1
684	h2o[p]	437	h[p]	2012	UDPGALPpp	0
684	h2o[p]	571	gal1p[p]	2012	UDPGALPpp	0
684	h2o[p]	1226	ump[p]	2012	UDPGALPpp	0
1223	udpgal[p]	437	h[p]	2012	UDPGALPpp	0
1223	udpgal[p]	571	gal1p[p]	2012	UDPGALPpp	1
1223	udpgal[p]	1226	ump[p]	2012	UDPGALPpp	1
1044	udpgal[e]	1223	udpgal[p]	2013	UDPGALtex	1
109	h2o	98	h	2014	UDPGD	0
109	h2o	870	nadh	2014	UDPGD	0
109	h2o	1545	udpglcur	2014	UDPGD	0
856	nad	98	h	2014	UDPGD	0
856	nad	870	nadh	2014	UDPGD	1
856	nad	1545	udpglcur	2014	UDPGD	0
1042	udpg	98	h	2014	UDPGD	0
1042	udpg	870	nadh	2014	UDPGD	0
1042	udpg	1545	udpglcur	2014	UDPGD	1
856	nad	692	co2	2015	UDPGDC	0
856	nad	870	nadh	2015	UDPGDC	1
856	nad	1546	udpLa4o	2015	UDPGDC	0
1545	udpglcur	692	co2	2015	UDPGDC	0
1545	udpglcur	870	nadh	2015	UDPGDC	0
1545	udpglcur	1546	udpLa4o	2015	UDPGDC	1
1045	udpglcur[e]	1224	udpglcur[p]	2016	UDPGLCURtex	1
684	h2o[p]	437	h[p]	2017	UDPGPpp	0
684	h2o[p]	553	g1p[p]	2017	UDPGPpp	0
684	h2o[p]	1226	ump[p]	2017	UDPGPpp	0
1225	udpg[p]	437	h[p]	2017	UDPGPpp	0
1225	udpg[p]	553	g1p[p]	2017	UDPGPpp	1
1225	udpg[p]	1226	ump[p]	2017	UDPGPpp	1
1046	udpg[e]	1225	udpg[p]	2018	UDPGtex	1
624	glu-L	213	akg	2019	UDPKAAT	1
624	glu-L	1548	udpLa4n	2019	UDPKAAT	0
1546	udpLa4o	213	akg	2019	UDPKAAT	0
1546	udpLa4o	1548	udpLa4n	2019	UDPKAAT	1
684	h2o[p]	437	h[p]	2020	UGLCURPpp	0
684	h2o[p]	681	glcur1p[p]	2020	UGLCURPpp	0
684	h2o[p]	1226	ump[p]	2020	UGLCURPpp	0
1224	udpglcur[p]	437	h[p]	2020	UGLCURPpp	0
1224	udpglcur[p]	681	glcur1p[p]	2020	UGLCURPpp	1
1224	udpglcur[p]	1226	ump[p]	2020	UGLCURPpp	1
1042	udpg	589	g1p	2021	UGLT	0
1042	udpg	1043	udpgal	2021	UGLT	1
1047	gal1p	589	g1p	2021	UGLT	1
1047	gal1p	1043	udpgal	2021	UGLT	0
98	h	631	glx	2022	UGLYCH	0
98	h	692	co2	2022	UGLYCH	0
98	h	1160	nh4	2022	UGLYCH	0
109	h2o	631	glx	2022	UGLYCH	0
109	h2o	692	co2	2022	UGLYCH	0
109	h2o	1160	nh4	2022	UGLYCH	0
1553	urdglyc	631	glx	2022	UGLYCH	1
1553	urdglyc	692	co2	2022	UGLYCH	0
1553	urdglyc	1160	nh4	2022	UGLYCH	0

135	atp	98	h	2023	UGMDDS	0
135	atp	281	adp	2023	UGMDDS	1
135	atp	962	pi	2023	UGMDDS	0
135	atp	1466	ugmda	2023	UGMDDS	0
218	alaala	98	h	2023	UGMDDS	0
218	alaala	281	adp	2023	UGMDDS	0
218	alaala	962	pi	2023	UGMDDS	0
218	alaala	1466	ugmda	2023	UGMDDS	1
1615	ugmd	98	h	2023	UGMDDS	0
1615	ugmd	281	adp	2023	UGMDDS	0
1615	ugmd	962	pi	2023	UGMDDS	0
1615	ugmd	1466	ugmda	2023	UGMDDS	1
109	h2o	147	ac	2024	UHGADA	0
109	h2o	1542	u3hga	2024	UHGADA	0
1547	u3aga	147	ac	2024	UHGADA	0
1547	u3aga	1542	u3hga	2024	UHGADA	1
210	10fthf	98	h	2025	ULA4NFT	0
210	10fthf	1403	thf	2025	ULA4NFT	1
210	10fthf	1550	udpLa4fn	2025	ULA4NFT	1
1548	udpLa4n	98	h	2025	ULA4NFT	0
1548	udpLa4n	1403	thf	2025	ULA4NFT	0
1548	udpLa4n	1550	udpLa4fn	2025	ULA4NFT	1
1048	uLa4n	1359	uLa4n[p]	2026	ULA4Ntppi	1
135	atp	98	h	2027	UM3PL	0
135	atp	281	adp	2027	UM3PL	1
135	atp	962	pi	2027	UM3PL	0
135	atp	1615	ugmd	2027	UM3PL	0
745	LalaDgluMdap	98	h	2027	UM3PL	0
745	LalaDgluMdap	281	adp	2027	UM3PL	0
745	LalaDgluMdap	962	pi	2027	UM3PL	0
745	LalaDgluMdap	1615	ugmd	2027	UM3PL	1
1614	uamr	98	h	2027	UM3PL	0
1614	uamr	281	adp	2027	UM3PL	0
1614	uamr	962	pi	2027	UM3PL	0
1614	uamr	1615	ugmd	2027	UM3PL	1
109	h2o	219	ala-D	2028	UM4PCP	0
109	h2o	1615	ugmd	2028	UM4PCP	0
1549	um4p	219	ala-D	2028	UM4PCP	1
1549	um4p	1615	ugmd	2028	UM4PCP	1
110	ılaDgluMdapDa	98	h	2029	UM4PL	0
110	ılaDgluMdapDa	281	adp	2029	UM4PL	0
110	ılaDgluMdapDa	962	pi	2029	UM4PL	0
110	ılaDgluMdapDa	1549	um4p	2029	UM4PL	1
135	atp	98	h	2029	UM4PL	0
135	atp	281	adp	2029	UM4PL	1
135	atp	962	pi	2029	UM4PL	0
135	atp	1549	um4p	2029	UM4PL	0
1614	uamr	98	h	2029	UM4PL	0
1614	uamr	281	adp	2029	UM4PL	0
1614	uamr	962	pi	2029	UM4PL	0
1614	uamr	1549	um4p	2029	UM4PL	1
135	atp	281	adp	2030	UMPK	1
135	atp	1440	udp	2030	UMPK	0
1242	ump	281	adp	2030	UMPK	0
1242	ump	1440	udp	2030	UMPK	1
1049	ump[e]	1226	ump[p]	2031	UMPtex	1

624	glu-L	213	akg	2032	UNK3	1
624	glu-L	1433	met-L	2032	UNK3	0
1050	2kmb	213	akg	2032	UNK3	0
1050	2kmb	1433	met-L	2032	UNK3	1
109	h2o	536	for	2033	UPLA4FNF	0
109	h2o	1048	uLa4n	2033	UPLA4FNF	0
1360	uLa4fn	536	for	2033	UPLA4FNF	1
1360	uLa4fn	1048	uLa4n	2033	UPLA4FNF	1
923	udcpp	1360	uLa4fn	2034	UPLA4FNT	1
923	udcpp	1440	udp	2034	UPLA4FNT	0
1550	udpLa4fn	1360	uLa4fn	2034	UPLA4FNT	1
1550	udpLa4fn	1440	udp	2034	UPLA4FNT	1
178	amet	98	h	2035	UPP3MT	0
178	amet	209	ahcys	2035	UPP3MT	1
178	amet	992	dscl	2035	UPP3MT	0
1551	uppg3	98	h	2035	UPP3MT	0
1551	uppg3	209	ahcys	2035	UPP3MT	1
1551	uppg3	992	dscl	2035	UPP3MT	1
1051	hmbil	109	h2o	2036	UPP3S	0
1051	hmbil	1551	uppg3	2036	UPP3S	1
98	h	343	cpppg3	2037	UPPDC1	0
98	h	692	co2	2037	UPPDC1	0
1551	uppg3	343	cpppg3	2037	UPPDC1	1
1551	uppg3	692	co2	2037	UPPDC1	0
1052	prpp	1192	ppi	2038	UPPRT	0
1052	prpp	1242	ump	2038	UPPRT	1
1552	ura	1192	ppi	2038	UPPRT	0
1552	ura	1242	ump	2038	UPPRT	1
437	h[p]	98	h	2039	URAt2pp	0
437	h[p]	1552	ura	2039	URAt2pp	0
1227	ura[p]	98	h	2039	URAt2pp	0
1227	ura[p]	1552	ura	2039	URAt2pp	1
437	h[p]	98	h	2040	URAt2rpp	0
437	h[p]	1552	ura	2040	URAt2rpp	0
1227	ura[p]	98	h	2040	URAt2rpp	0
1227	ura[p]	1552	ura	2040	URAt2rpp	1
1053	ura[e]	1227	ura[p]	2041	URAtex	1
856	nad	98	h	2042	URDGLYCD	0
856	nad	870	nadh	2042	URDGLYCD	1
856	nad	919	oxur	2042	URDGLYCD	0
1553	urdglyc	98	h	2042	URDGLYCD	0
1553	urdglyc	870	nadh	2042	URDGLYCD	0
1553	urdglyc	919	oxur	2042	URDGLYCD	1
1054	urea[e]	1055	urea[p]	2043	UREAtex	1
1055	urea[p]	1228	urea	2044	UREAtpp	1
109	h2o	232	alltn	2045	URIC	0
109	h2o	292	h2o2	2045	URIC	0
109	h2o	692	co2	2045	URIC	0
928	o2	232	alltn	2045	URIC	0
928	o2	292	h2o2	2045	URIC	0
928	o2	692	co2	2045	URIC	0
1654	urate	232	alltn	2045	URIC	1
1654	urate	292	h2o2	2045	URIC	0
1654	urate	692	co2	2045	URIC	0
135	atp	281	adp	2046	URIDK2r	1
135	atp	1351	dudp	2046	URIDK2r	0

875	dump	281	adp	2046	URIDK2r	0
875	dump	1351	dudp	2046	URIDK2r	1
109	h2o	1361	rib-D	2047	URIH	0
109	h2o	1552	ura	2047	URIH	0
1512	uri	1361	rib-D	2047	URIH	1
1512	uri	1552	ura	2047	URIH	1
673	gtp	98	h	2048	URIK2	0
673	gtp	798	gdp	2048	URIK2	1
673	gtp	1242	ump	2048	URIK2	0
1512	uri	98	h	2048	URIK2	0
1512	uri	798	gdp	2048	URIK2	0
1512	uri	1242	ump	2048	URIK2	1
437	h[p]	98	h	2049	URIt2pp	0
437	h[p]	1512	uri	2049	URIt2pp	0
1229	uri[p]	98	h	2049	URIt2pp	0
1229	uri[p]	1512	uri	2049	URIt2pp	1
437	h[p]	98	h	2050	URIt2rpp	0
437	h[p]	1512	uri	2050	URIt2rpp	0
1229	uri[p]	98	h	2050	URIt2rpp	0
1229	uri[p]	1512	uri	2050	URIt2rpp	1
1056	uri[e]	1229	uri[p]	2051	URItex	1
109	h2o	98	h	2052	USHD	0
109	h2o	756	lipidX	2052	USHD	0
109	h2o	1242	ump	2052	USHD	0
1424	u23ga	98	h	2052	USHD	0
1424	u23ga	756	lipidX	2052	USHD	1
1424	u23ga	1242	ump	2052	USHD	1
109	h2o	98	h	2053	VALabcpp	0
109	h2o	281	adp	2053	VALabcpp	0
109	h2o	962	pi	2053	VALabcpp	0
109	h2o	1554	val-L	2053	VALabcpp	0
135	atp	98	h	2053	VALabcpp	0
135	atp	281	adp	2053	VALabcpp	1
135	atp	962	pi	2053	VALabcpp	0
135	atp	1554	val-L	2053	VALabcpp	0
1230	val-L[p]	98	h	2053	VALabcpp	0
1230	val-L[p]	281	adp	2053	VALabcpp	0
1230	val-L[p]	962	pi	2053	VALabcpp	0
1230	val-L[p]	1554	val-L	2053	VALabcpp	1
437	h[p]	98	h	2054	VALt2rpp	0
437	h[p]	1554	val-L	2054	VALt2rpp	0
1230	val-L[p]	98	h	2054	VALt2rpp	0
1230	val-L[p]	1554	val-L	2054	VALt2rpp	1
213	akg	624	glu-L	2055	VALTA	1
213	akg	729	3mob	2055	VALTA	0
1554	val-L	624	glu-L	2055	VALTA	0
1554	val-L	729	3mob	2055	VALTA	1
1057	val-L[e]	1230	val-L[p]	2056	VALtex	1
135	atp	177	amp	2057	VALTRS	1
135	atp	1192	ppi	2057	VALTRS	0
135	atp	1667	valtrna	2057	VALTRS	0
1554	val-L	177	amp	2057	VALTRS	0
1554	val-L	1192	ppi	2057	VALTRS	0
1554	val-L	1667	valtrna	2057	VALTRS	1
1555	trnaval	177	amp	2057	VALTRS	0
1555	trnaval	1192	ppi	2057	VALTRS	0

1555	trnaval	1667	valtrna	2057	VALTRS	1
222	ala-L	1148	pyr	2058	VPAMT	1
222	ala-L	1554	val-L	2058	VPAMT	0
729	3mob	1148	pyr	2058	VPAMT	0
729	3mob	1554	val-L	2058	VPAMT	1
1058	xu5p-L	973	ru5p-L	2059	X5PL3E	1
109	h2o	98	h	2060	XAND	0
109	h2o	870	nadh	2060	XAND	0
109	h2o	1654	urate	2060	XAND	0
856	nad	98	h	2060	XAND	0
856	nad	870	nadh	2060	XAND	1
856	nad	1654	urate	2060	XAND	0
1231	xan	98	h	2060	XAND	0
1231	xan	870	nadh	2060	XAND	0
1231	xan	1654	urate	2060	XAND	1
437	h[p]	98	h	2061	XANt2pp	0
437	h[p]	1231	xan	2061	XANt2pp	0
1060	xan[p]	98	h	2061	XANt2pp	0
1060	xan[p]	1231	xan	2061	XANt2pp	1
1059	xan[e]	1060	xan[p]	2062	XANtex	1
1060	xan[p]	1231	xan	2063	XANtpp	1
1061	xmp[e]	1232	xmp[p]	2064	XMPtex	1
1052	prpp	1192	ppi	2065	XPPT	0
1052	prpp	1445	xmp	2065	XPPT	1
1231	xan	1192	ppi	2065	XPPT	0
1231	xan	1445	xmp	2065	XPPT	1
109	h2o	1231	xan	2066	XTSNH	0
109	h2o	1361	rib-D	2066	XTSNH	0
1508	xtsn	1231	xan	2066	XTSNH	1
1508	xtsn	1361	rib-D	2066	XTSNH	1
437	h[p]	98	h	2067	XTSNt2rpp	0
437	h[p]	1508	xtsn	2067	XTSNt2rpp	0
1233	xtsn[p]	98	h	2067	XTSNt2rpp	0
1233	xtsn[p]	1508	xtsn	2067	XTSNt2rpp	1
1062	xtsn[e]	1233	xtsn[p]	2068	XTSNtex	1
109	h2o	98	h	2069	XYLabcpp	0
109	h2o	281	adp	2069	XYLabcpp	0
109	h2o	962	pi	2069	XYLabcpp	0
109	h2o	1063	xyl-D	2069	XYLabcpp	0
135	atp	98	h	2069	XYLabcpp	0
135	atp	281	adp	2069	XYLabcpp	1
135	atp	962	pi	2069	XYLabcpp	0
135	atp	1063	xyl-D	2069	XYLabcpp	0
1236	xyl-D[p]	98	h	2069	XYLabcpp	0
1236	xyl-D[p]	281	adp	2069	XYLabcpp	0
1236	xyl-D[p]	962	pi	2069	XYLabcpp	0
1236	xyl-D[p]	1063	xyl-D	2069	XYLabcpp	1
1063	xyl-D	1234	xylu-D	2070	XYLI1	1
1064	glc-D	1235	fru	2071	XYLI2	1
135	atp	98	h	2072	XYLK	0
135	atp	281	adp	2072	XYLK	1
135	atp	1187	xu5p-D	2072	XYLK	0
1234	xylu-D	98	h	2072	XYLK	0
1234	xylu-D	281	adp	2072	XYLK	0
1234	xylu-D	1187	xu5p-D	2072	XYLK	1
135	atp	98	h	2073	XYLK2	0

135	atp	281	adp	2073	XYLK2	1
135	atp	1058	xu5p-L	2073	XYLK2	0
1311	xylu-L	98	h	2073	XYLK2	0
1311	xylu-L	281	adp	2073	XYLK2	0
1311	xylu-L	1058	xu5p-L	2073	XYLK2	1
437	h[p]	98	h	2074	XYLt2pp	0
437	h[p]	1063	xyl-D	2074	XYLt2pp	0
1236	xyl-D[p]	98	h	2074	XYLt2pp	0
1236	xyl-D[p]	1063	xyl-D	2074	XYLt2pp	1
1065	xyl-D[e]	1236	xyl-D[p]	2075	XYLtex	1
437	h[p]	98	h	2076	XYLUt2pp	0
437	h[p]	1311	xylu-L	2076	XYLUt2pp	0
1237	xylu-L[p]	98	h	2076	XYLUt2pp	0
1237	xylu-L[p]	1311	xylu-L	2076	XYLUt2pp	1
1066	xylu-L[e]	1237	xylu-L[p]	2077	XYLUtex	1
109	h2o	98	h	2078	ZN2abcpp	0
109	h2o	281	adp	2078	ZN2abcpp	0
109	h2o	962	pi	2078	ZN2abcpp	0
109	h2o	1068	zn2[p]	2078	ZN2abcpp	0
135	atp	98	h	2078	ZN2abcpp	0
135	atp	281	adp	2078	ZN2abcpp	1
135	atp	962	pi	2078	ZN2abcpp	0
135	atp	1068	zn2[p]	2078	ZN2abcpp	0
1362	zn2	98	h	2078	ZN2abcpp	0
1362	zn2	281	adp	2078	ZN2abcpp	0
1362	zn2	962	pi	2078	ZN2abcpp	0
1362	zn2	1068	zn2[p]	2078	ZN2abcpp	0
437	h[p]	98	h	2079	ZN2t3pp	0
437	h[p]	1068	zn2[p]	2079	ZN2t3pp	0
1362	zn2	98	h	2079	ZN2t3pp	0
1362	zn2	1068	zn2[p]	2079	ZN2t3pp	0
1067	zn2[e]	1068	zn2[p]	2080	Zn2tex	0
1068	zn2[p]	1362	zn2	2081	ZN2tpp	0
109	h2o	98	h	2082	ZNabcpp	0
109	h2o	281	adp	2082	ZNabcpp	0
109	h2o	962	pi	2082	ZNabcpp	0
109	h2o	1362	zn2	2082	ZNabcpp	0
135	atp	98	h	2082	ZNabcpp	0
135	atp	281	adp	2082	ZNabcpp	1
135	atp	962	pi	2082	ZNabcpp	0
135	atp	1362	zn2	2082	ZNabcpp	0
1068	zn2[p]	98	h	2082	ZNabcpp	0
1068	zn2[p]	281	adp	2082	ZNabcpp	0
1068	zn2[p]	962	pi	2082	ZNabcpp	0
1068	zn2[p]	1362	zn2	2082	ZNabcpp	0
9	12ppd-R[p]	8	12ppd-R[e]	2083	Reversible_12PPDRtex	1
1069	12ppd-R	9	12ppd-R[p]	2084	Reversible_12PPDRtpp	1
11	12ppd-S[p]	10	12ppd-S[e]	2085	Reversible_12PPDStex	1
1070	12ppd-S	11	12ppd-S[p]	2086	Reversible_12PPDStpp	1
22	23camp[p]	14	23camp[e]	2087	Reversible_23CAMPtex	1
21	23ccmp[p]	15	23ccmp[e]	2088	Reversible_23CCMPtex	1
23	23cgmp[p]	16	23cgmp[e]	2089	Reversible_23CGMPtex	1
20	23cump[p]	17	23cump[e]	2090	Reversible_23CUMPtex	1
18	23dappa[p]	19	23dappa[e]	2091	Reversible_23DAPPAtex	1
387	26dap-M[p]	24	26dap-M[e]	2092	Reversible_26DAHtex	1
1071	34dhpac[p]	63	34dhpac[e]	2093	Reversible_34dhpactex	1

84	3amp[p]	64	3amp[e]	2094	Reversible_3AMPtex	1
83	3cmp[p]	65	3cmp[e]	2095	Reversible_3CMPtex	1
85	3gmp[p]	66	3gmp[e]	2096	Reversible_3GMPtex	1
67	3hdecACP	86	3odecACP	2097	Reversible_3OAR100	1
67	3hdecACP	98	h	2097	Reversible_3OAR100	0
67	3hdecACP	871	nadph	2097	Reversible_3OAR100	0
459	nadp	86	3odecACP	2097	Reversible_3OAR100	0
459	nadp	98	h	2097	Reversible_3OAR100	0
459	nadp	871	nadph	2097	Reversible_3OAR100	1
68	3hddecACP	87	3oddecACP	2098	Reversible_3OAR120	1
68	3hddecACP	98	h	2098	Reversible_3OAR120	0
68	3hddecACP	871	nadph	2098	Reversible_3OAR120	0
459	nadp	87	3oddecACP	2098	Reversible_3OAR120	0
459	nadp	98	h	2098	Reversible_3OAR120	0
459	nadp	871	nadph	2098	Reversible_3OAR120	1
70	3hmrsACP	89	3omrsACP	2099	Reversible_3OAR140	1
70	3hmrsACP	98	h	2099	Reversible_3OAR140	0
70	3hmrsACP	871	nadph	2099	Reversible_3OAR140	0
459	nadp	89	3omrsACP	2099	Reversible_3OAR140	0
459	nadp	98	h	2099	Reversible_3OAR140	0
459	nadp	871	nadph	2099	Reversible_3OAR140	1
72	3hpalmACP	91	3opalmACP	2100	Reversible_3OAR160	1
72	3hpalmACP	98	h	2100	Reversible_3OAR160	0
72	3hpalmACP	871	nadph	2100	Reversible_3OAR160	0
459	nadp	91	3opalmACP	2100	Reversible_3OAR160	0
459	nadp	98	h	2100	Reversible_3OAR160	0
459	nadp	871	nadph	2100	Reversible_3OAR160	1
74	3hoctaACP	93	3ooctdACP	2101	Reversible_3OAR180	1
74	3hoctaACP	98	h	2101	Reversible_3OAR180	0
74	3hoctaACP	871	nadph	2101	Reversible_3OAR180	0
459	nadp	93	3ooctdACP	2101	Reversible_3OAR180	0
459	nadp	98	h	2101	Reversible_3OAR180	0
459	nadp	871	nadph	2101	Reversible_3OAR180	1
76	3haACP	95	actACP	2102	Reversible_3OAR40	1
76	3haACP	98	h	2102	Reversible_3OAR40	0
76	3haACP	871	nadph	2102	Reversible_3OAR40	0
459	nadp	95	actACP	2102	Reversible_3OAR40	0
459	nadp	98	h	2102	Reversible_3OAR40	0
459	nadp	871	nadph	2102	Reversible_3OAR40	1
77	3hhexACP	96	3ohexACP	2103	Reversible_3OAR60	1
77	3hhexACP	98	h	2103	Reversible_3OAR60	0
77	3hhexACP	871	nadph	2103	Reversible_3OAR60	0
459	nadp	96	3ohexACP	2103	Reversible_3OAR60	0
459	nadp	98	h	2103	Reversible_3OAR60	0
459	nadp	871	nadph	2103	Reversible_3OAR60	1
78	3hoctACP	97	3ooctACP	2104	Reversible_3OAR80	1
78	3hoctACP	98	h	2104	Reversible_3OAR80	0
78	3hoctACP	871	nadph	2104	Reversible_3OAR80	0
459	nadp	97	3ooctACP	2104	Reversible_3OAR80	0
459	nadp	98	h	2104	Reversible_3OAR80	0
459	nadp	871	nadph	2104	Reversible_3OAR80	1
104	.alaDgluMdap[p	105	LalaDgluMdap[e]	2105	Reversible_3PEPTtex	1
82	3ump[p]	106	3ump[e]	2106	Reversible_3UMPtex	1
1072	4hoxpacd[p]	108	4hoxpacd[e]	2107	Reversible_4HOXPACDtex	1
111	aDgluMdapDala	112	LalaDgluMdapDala[e]	2108	Reversible_4PEPTtex	1
459	nadp	98	h	2109	Reversible_5DGLCNR	0

459	nadp	113	5dglcn	2109	Reversible_5DGLCNR	0
459	nadp	871	nadph	2109	Reversible_5DGLCNR	1
1073	glcn	98	h	2109	Reversible_5DGLCNR	0
1073	glcn	113	5dglcn	2109	Reversible_5DGLCNR	1
1073	glcn	871	nadph	2109	Reversible_5DGLCNR	0
98	h	114	5dglcn[p]	2110	Reversible_5DGLCNt2rpp	0
98	h	437	h[p]	2110	Reversible_5DGLCNt2rpp	0
113	5dglcn	114	5dglcn[p]	2110	Reversible_5DGLCNt2rpp	1
113	5dglcn	437	h[p]	2110	Reversible_5DGLCNt2rpp	0
114	5dglcn[p]	115	5dglcn[e]	2111	Reversible_5DGLCNtex	1
742	ara5p	117	ru5p-D	2112	Reversible_A5PISO	1
125	4abut[p]	126	4abut[e]	2113	Reversible_ABUTtex	1
734	aacoa	128	accoa	2114	Reversible_ACACT1r	1
927	coa	128	accoa	2114	Reversible_ACACT1r	1
98	h	129	acac[p]	2115	Reversible_ACACt2pp	0
98	h	437	h[p]	2115	Reversible_ACACt2pp	0
127	acac	129	acac[p]	2115	Reversible_ACACt2pp	1
127	acac	437	h[p]	2115	Reversible_ACACt2pp	0
129	acac[p]	130	acac[e]	2116	Reversible_ACACtex	1
98	h	131	acald	2117	Reversible_ACALD	0
98	h	856	nad	2117	Reversible_ACALD	0
98	h	927	coa	2117	Reversible_ACALD	0
128	accoa	131	acald	2117	Reversible_ACALD	1
128	accoa	856	nad	2117	Reversible_ACALD	0
128	accoa	927	coa	2117	Reversible_ACALD	1
870	nadh	131	acald	2117	Reversible_ACALD	0
870	nadh	856	nad	2117	Reversible_ACALD	1
870	nadh	927	coa	2117	Reversible_ACALD	0
133	acald[p]	132	acald[e]	2118	Reversible_ACALDtex	1
131	acald	133	acald[p]	2119	Reversible_ACALDtpp	1
136	acgal1p[p]	137	acgal1p[e]	2120	Reversible_ACGAL1Ptex	1
1074	acgal[p]	138	acgal[e]	2121	Reversible_ACGALtex	1
139	acgam1p[p]	140	acgam1p[e]	2122	Reversible_ACGAM1Ptex	1
143	acgam[p]	144	acgam[e]	2123	Reversible_ACGAtex	1
281	adp	135	atp	2124	Reversible_ACKr	1
281	adp	147	ac	2124	Reversible_ACKr	0
1075	actp	135	atp	2124	Reversible_ACKr	0
1075	actp	147	ac	2124	Reversible_ACKr	1
150	acmana[p]	151	acmana[e]	2125	Reversible_ACMANAtex	1
154	acnam[p]	155	acnam[e]	2126	Reversible_ACNAMtex	1
520	fadh2	157	btcoa	2127	Reversible_ACOAD1f	0
520	fadh2	158	fad	2127	Reversible_ACOAD1f	1
1076	b2coa	157	btcoa	2127	Reversible_ACOAD1f	1
1076	b2coa	158	fad	2127	Reversible_ACOAD1f	0
520	fadh2	158	fad	2128	Reversible_ACOAD2f	1
520	fadh2	1367	hxcoa	2128	Reversible_ACOAD2f	0
1556	hx2coa	158	fad	2128	Reversible_ACOAD2f	0
1556	hx2coa	1367	hxcoa	2128	Reversible_ACOAD2f	1
520	fadh2	158	fad	2129	Reversible_ACOAD3f	1
520	fadh2	1368	occoa	2129	Reversible_ACOAD3f	0
1557	oc2coa	158	fad	2129	Reversible_ACOAD3f	0
1557	oc2coa	1368	occoa	2129	Reversible_ACOAD3f	1
520	fadh2	158	fad	2130	Reversible_ACOAD4f	1
520	fadh2	159	dcacoa	2130	Reversible_ACOAD4f	0
1077	dc2coa	158	fad	2130	Reversible_ACOAD4f	0
1077	dc2coa	159	dcacoa	2130	Reversible_ACOAD4f	1

520	fadh2	158	fad	2131	Reversible_ACOAD5f	1
520	fadh2	160	ddcacoa	2131	Reversible_ACOAD5f	0
1078	dd2coa	158	fad	2131	Reversible_ACOAD5f	0
1078	dd2coa	160	ddcacoa	2131	Reversible_ACOAD5f	1
520	fadh2	158	fad	2132	Reversible_ACOAD6f	1
520	fadh2	1369	tdcoa	2132	Reversible_ACOAD6f	0
1354	td2coa	158	fad	2132	Reversible_ACOAD6f	0
1354	td2coa	1369	tdcoa	2132	Reversible_ACOAD6f	1
520	fadh2	158	fad	2133	Reversible_ACOAD7f	1
520	fadh2	1370	pmtcoa	2133	Reversible_ACOAD7f	0
1291	hdd2coa	158	fad	2133	Reversible_ACOAD7f	0
1291	hdd2coa	1370	pmtcoa	2133	Reversible_ACOAD7f	1
520	fadh2	158	fad	2134	Reversible_ACOAD8f	1
520	fadh2	1371	stcoa	2134	Reversible_ACOAD8f	0
1334	od2coa	158	fad	2134	Reversible_ACOAD8f	0
1334	od2coa	1371	stcoa	2134	Reversible_ACOAD8f	1
733	acACP	118	ACP	2135	Reversible_ACOATA	1
733	acACP	128	accoa	2135	Reversible_ACOATA	1
927	coa	118	ACP	2135	Reversible_ACOATA	0
927	coa	128	accoa	2135	Reversible_ACOATA	1
165	acon-C	163	acon-T	2136	Reversible_ACONIs	1
109	h2o	164	cit	2137	Reversible_ACONTa	0
165	acon-C	164	cit	2137	Reversible_ACONTa	1
716	icit	109	h2o	2138	Reversible_ACONTb	0
716	icit	165	acon-C	2138	Reversible_ACONTb	1
207	acg5sa	161	acorn	2139	Reversible_ACOTA	1
207	acg5sa	213	akg	2139	Reversible_ACOTA	0
624	glu-L	161	acorn	2139	Reversible_ACOTA	0
624	glu-L	213	akg	2139	Reversible_ACOTA	1
1079	acser[p]	167	acser[e]	2140	Reversible_ACSERtex	1
98	h	169	ac[p]	2141	Reversible_ACt2rpp	0
98	h	437	h[p]	2141	Reversible_ACt2rpp	0
147	ac	169	ac[p]	2141	Reversible_ACt2rpp	1
147	ac	437	h[p]	2141	Reversible_ACt2rpp	0
169	ac[p]	170	ac[e]	2142	Reversible_ACtex	1
98	h	175	ade[p]	2143	Reversible_ADEt2rpp	0
98	h	437	h[p]	2143	Reversible_ADEt2rpp	0
174	ade	175	ade[p]	2143	Reversible_ADEt2rpp	1
174	ade	437	h[p]	2143	Reversible_ADEt2rpp	0
175	ade[p]	176	ade[e]	2144	Reversible_ADEtex	1
281	adp	135	atp	2145	Reversible_ADK1	1
281	adp	177	amp	2145	Reversible_ADK1	1
281	adp	177	amp	2146	Reversible_ADK3	1
281	adp	673	gtp	2146	Reversible_ADK3	0
798	gdp	177	amp	2146	Reversible_ADK3	0
798	gdp	673	gtp	2146	Reversible_ADK3	1
281	adp	177	amp	2147	Reversible_ADK4	1
281	adp	1249	itp	2147	Reversible_ADK4	0
1558	idp	177	amp	2147	Reversible_ADK4	0
1558	idp	1249	itp	2147	Reversible_ADK4	1
98	h	179	adn[p]	2148	Reversible_ADNt2rpp	0
98	h	437	h[p]	2148	Reversible_ADNt2rpp	0
171	adn	179	adn[p]	2148	Reversible_ADNt2rpp	1
171	adn	437	h[p]	2148	Reversible_ADNt2rpp	0
179	adn[p]	180	adn[e]	2149	Reversible_ADNtex	1
177	amp	187	dcamp	2150	Reversible_ADSL1r	1

540	fum	187	dcamp	2150	Reversible_ADSL1r	1
540	fum	188	25aics	2151	Reversible_ADSL2r	1
1080	aicar	188	25aics	2151	Reversible_ADSL2r	1
1081	agm[p]	199	agm[e]	2152	Reversible_AGMtex	1
98	h	207	acg5sa	2153	Reversible_AGPR	0
98	h	459	nadp	2153	Reversible_AGPR	0
98	h	962	pi	2153	Reversible_AGPR	0
871	nadph	207	acg5sa	2153	Reversible_AGPR	0
871	nadph	459	nadp	2153	Reversible_AGPR	1
871	nadph	962	pi	2153	Reversible_AGPR	0
1082	acg5p	207	acg5sa	2153	Reversible_AGPR	1
1082	acg5p	459	nadp	2153	Reversible_AGPR	0
1082	acg5p	962	pi	2153	Reversible_AGPR	0
1083	fprica	210	10fthf	2154	Reversible_AICART	1
1083	fprica	1080	aicar	2154	Reversible_AICART	1
1403	thf	210	10fthf	2154	Reversible_AICART	1
1403	thf	1080	aicar	2154	Reversible_AICART	0
1084	5caiz	212	5aizc	2155	Reversible_AIRC3	1
98	h	214	akg[p]	2156	Reversible_AKGt2rpp	0
98	h	437	h[p]	2156	Reversible_AKGt2rpp	0
213	akg	214	akg[p]	2156	Reversible_AKGt2rpp	1
213	akg	437	h[p]	2156	Reversible_AKGt2rpp	0
214	akg[p]	215	akg[e]	2157	Reversible_AKGtex	1
98	h	135	atp	2158	Reversible_ALAALAr	0
98	h	219	ala-D	2158	Reversible_ALAALAr	0
218	alaala	135	atp	2158	Reversible_ALAALAr	0
218	alaala	219	ala-D	2158	Reversible_ALAALAr	1
281	adp	135	atp	2158	Reversible_ALAALAr	1
281	adp	219	ala-D	2158	Reversible_ALAALAr	0
962	pi	135	atp	2158	Reversible_ALAALAr	0
962	pi	219	ala-D	2158	Reversible_ALAALAr	0
217	alaala[p]	220	alaala[e]	2159	Reversible_ALAALAtex	1
746	LalaLglu	221	LalaDglu	2160	Reversible_ALAGLUE	1
219	ala-D	222	ala-L	2161	Reversible_ALAR	1
624	glu-L	213	akg	2162	Reversible_ALATA_L	1
624	glu-L	222	ala-L	2162	Reversible_ALATA_L	0
1148	pyr	213	akg	2162	Reversible_ALATA_L	0
1148	pyr	222	ala-L	2162	Reversible_ALATA_L	1
216	ala-L[p]	223	ala-L[e]	2163	Reversible_ALAtex	1
641	glyc	98	h	2164	Reversible_ALCD19	0
641	glyc	224	glyald	2164	Reversible_ALCD19	1
641	glyc	870	nadh	2164	Reversible_ALCD19	0
856	nad	98	h	2164	Reversible_ALCD19	0
856	nad	224	glyald	2164	Reversible_ALCD19	0
856	nad	870	nadh	2164	Reversible_ALCD19	1
98	h	225	etoh	2165	Reversible_ALCD2x	0
98	h	856	nad	2165	Reversible_ALCD2x	0
131	acald	225	etoh	2165	Reversible_ALCD2x	1
131	acald	856	nad	2165	Reversible_ALCD2x	0
870	nadh	225	etoh	2165	Reversible_ALCD2x	0
870	nadh	856	nad	2165	Reversible_ALCD2x	1
235	allul6p	229	all6p	2166	Reversible_ALLPI	1
227	all-D[p]	231	all-D[e]	2167	Reversible_ALLtex	1
98	h	233	alltn[p]	2168	Reversible_ALLTNt2rpp	0
98	h	437	h[p]	2168	Reversible_ALLTNt2rpp	0
232	alltn	233	alltn[p]	2168	Reversible_ALLTNt2rpp	1

232	alltn	437	h[p]	2168	Reversible_ALLTNt2rpp	0
233	alltn[p]	234	alltn[e]	2169	Reversible_ALLTNtex	1
499	f6p	235	allul6p	2170	Reversible_ALLULPE	1
190	acgam6p	242	acmanap	2171	Reversible_AMANAPEr	1
1085	amob	178	amet	2172	Reversible_AMAOTr	1
1085	amob	244	8aonn	2172	Reversible_AMAOTr	0
1559	dann	178	amet	2172	Reversible_AMAOTr	0
1559	dann	244	8aonn	2172	Reversible_AMAOTr	1
886	amp[p]	247	amp[e]	2173	Reversible_AMPtex	1
198	anhgm[p]	248	anhgm[e]	2174	Reversible_ANHGMtex	1
244	8aonn	98	h	2175	Reversible_AOXSr	0
244	8aonn	222	ala-L	2175	Reversible_AOXSr	1
244	8aonn	1624	pmcoa	2175	Reversible_AOXSr	1
692	co2	98	h	2175	Reversible_AOXSr	0
692	co2	222	ala-L	2175	Reversible_AOXSr	0
692	co2	1624	pmcoa	2175	Reversible_AOXSr	0
927	coa	98	h	2175	Reversible_AOXSr	0
927	coa	222	ala-L	2175	Reversible_AOXSr	0
927	coa	1624	pmcoa	2175	Reversible_AOXSr	1
1086	rbl-L	255	arab-L	2176	Reversible_ARAI	1
98	h	256	arab-L[p]	2177	Reversible_ARBt2rpp	0
98	h	437	h[p]	2177	Reversible_ARBt2rpp	0
255	arab-L	256	arab-L[p]	2177	Reversible_ARBt2rpp	1
255	arab-L	437	h[p]	2177	Reversible_ARBt2rpp	0
256	arab-L[p]	257	arab-L[e]	2178	Reversible_ARBtex	1
265	arg-L	197	agm	2179	Reversible_ARGAGMt7pp	0
265	arg-L	264	arg-L[p]	2179	Reversible_ARGAGMt7pp	1
1081	agm[p]	197	agm	2179	Reversible_ARGAGMt7pp	1
1081	agm[p]	264	arg-L[p]	2179	Reversible_ARGAGMt7pp	0
265	arg-L	264	arg-L[p]	2180	Reversible_ARGORNt7pp	1
265	arg-L	963	orn	2180	Reversible_ARGORNt7pp	0
1167	orn[p]	264	arg-L[p]	2180	Reversible_ARGORNt7pp	0
1167	orn[p]	963	orn	2180	Reversible_ARGORNt7pp	1
265	arg-L	266	argsuc	2181	Reversible_ARGSL	1
540	fum	266	argsuc	2181	Reversible_ARGSL	1
264	arg-L[p]	267	arg-L[e]	2182	Reversible_ARGtex	1
98	h	268	aspsa	2183	Reversible_ASAD	0
98	h	459	nadp	2183	Reversible_ASAD	0
98	h	962	pi	2183	Reversible_ASAD	0
871	nadph	268	aspsa	2183	Reversible_ASAD	0
871	nadph	459	nadp	2183	Reversible_ASAD	1
871	nadph	962	pi	2183	Reversible_ASAD	0
1087	4pasp	268	aspsa	2183	Reversible_ASAD	1
1087	4pasp	459	nadp	2183	Reversible_ASAD	0
1087	4pasp	962	pi	2183	Reversible_ASAD	0
270	ascb-L[p]	271	ascb-L[e]	2184	Reversible_ASCBtex	1
98	h	272	asn-L[p]	2185	Reversible_ASNt2rpp	0
98	h	437	h[p]	2185	Reversible_ASNt2rpp	0
273	asn-L	272	asn-L[p]	2185	Reversible_ASNt2rpp	1
273	asn-L	437	h[p]	2185	Reversible_ASNt2rpp	0
272	asn-L[p]	274	asn-L[e]	2186	Reversible_ASNtex	1
1088	aso3[p]	276	aso3[e]	2187	Reversible_ASO3tex	1
281	adp	135	atp	2188	Reversible_ASPK	1
281	adp	189	asp-L	2188	Reversible_ASPK	0
1087	4pasp	135	atp	2188	Reversible_ASPK	0
1087	4pasp	189	asp-L	2188	Reversible_ASPK	1

624	glu-L	189	asp-L	2189	Reversible_ASPTA	0
624	glu-L	213	akg	2189	Reversible_ASPTA	1
1318	oaa	189	asp-L	2189	Reversible_ASPTA	1
1318	oaa	213	akg	2189	Reversible_ASPTA	0
277	asp-L[p]	278	asp-L[e]	2190	Reversible_ASPtex	1
98	h	280	athr-L	2191	Reversible_ATHRDHr	0
98	h	459	nadp	2191	Reversible_ATHRDHr	0
251	2aobut	280	athr-L	2191	Reversible_ATHRDHr	1
251	2aobut	459	nadp	2191	Reversible_ATHRDHr	0
871	nadph	280	athr-L	2191	Reversible_ATHRDHr	0
871	nadph	459	nadp	2191	Reversible_ATHRDHr	1
98	h	281	adp	2192	Reversible_ATPS4rpp	0
98	h	437	h[p]	2192	Reversible_ATPS4rpp	0
98	h	962	pi	2192	Reversible_ATPS4rpp	0
109	h2o	281	adp	2192	Reversible_ATPS4rpp	0
109	h2o	437	h[p]	2192	Reversible_ATPS4rpp	0
109	h2o	962	pi	2192	Reversible_ATPS4rpp	0
135	atp	281	adp	2192	Reversible_ATPS4rpp	1
135	atp	437	h[p]	2192	Reversible_ATPS4rpp	0
135	atp	962	pi	2192	Reversible_ATPS4rpp	0
282	ala-B[p]	283	ala-B[e]	2193	Reversible_BALAtex	1
1089	butso3[p]	286	butso3[e]	2194	Reversible_BUTSO3tex	1
98	h	287	but[p]	2195	Reversible_BUTt2rpp	0
98	h	437	h[p]	2195	Reversible_BUTt2rpp	0
1090	but	287	but[p]	2195	Reversible_BUTt2rpp	1
1090	but	437	h[p]	2195	Reversible_BUTt2rpp	0
287	but[p]	288	but[e]	2196	Reversible_BUTtex	1
1091	ca2[p]	290	ca2[e]	2197	Reversible_CA2tex	0
1091	ca2[p]	289	ca2	2198	Reversible_CAt6pp	0
1091	ca2[p]	941	na1[p]	2198	Reversible_CAt6pp	0
1344	na1	289	ca2	2198	Reversible_CAt6pp	0
1344	na1	941	na1[p]	2198	Reversible_CAt6pp	0
181	adocbi	98	h	2199	Reversible_CBIAT	0
181	adocbi	135	atp	2199	Reversible_CBIAT	1
181	adocbi	1385	cbi	2199	Reversible_CBIAT	1
1491	pppi	98	h	2199	Reversible_CBIAT	0
1491	pppi	135	atp	2199	Reversible_CBIAT	0
1491	pppi	1385	cbi	2199	Reversible_CBIAT	0
1092	adocbl	98	h	2200	Reversible_CBLAT	0
1092	adocbl	135	atp	2200	Reversible_CBLAT	1
1092	adocbl	1386	cbl1	2200	Reversible_CBLAT	1
1491	pppi	98	h	2200	Reversible_CBLAT	0
1491	pppi	135	atp	2200	Reversible_CBLAT	0
1491	pppi	1386	cbl1	2200	Reversible_CBLAT	0
98	h	135	atp	2201	Reversible_CBMKr	0
98	h	692	co2	2201	Reversible_CBMKr	0
98	h	1160	nh4	2201	Reversible_CBMKr	0
281	adp	135	atp	2201	Reversible_CBMKr	1
281	adp	692	co2	2201	Reversible_CBMKr	0
281	adp	1160	nh4	2201	Reversible_CBMKr	0
904	cbp	135	atp	2201	Reversible_CBMKr	0
904	cbp	692	co2	2201	Reversible_CBMKr	1
904	cbp	1160	nh4	2201	Reversible_CBMKr	0
297	cd2[p]	296	cd2[e]	2202	Reversible_CD2tex	1
1093	cgly[p]	306	cgly[e]	2203	Reversible_CGLYtex	1
307	chol[p]	308	chol[e]	2204	Reversible_CHLtex	1

311	cit[p]	312	cit[e]	2205	Reversible_CITtex	1
314	clpn120[p]	321	pg120[p]	2206	Reversible_CLPNS120pp	1
1119	glyc[p]	321	pg120[p]	2206	Reversible_CLPNS120pp	1
315	clpn140[p]	322	pg140[p]	2207	Reversible_CLPNS140pp	1
1119	glyc[p]	322	pg140[p]	2207	Reversible_CLPNS140pp	1
316	clpn141[p]	323	pg141[p]	2208	Reversible_CLPNS141pp	1
1119	glyc[p]	323	pg141[p]	2208	Reversible_CLPNS141pp	1
317	clpn160[p]	324	pg160[p]	2209	Reversible_CLPNS160pp	1
1119	glyc[p]	324	pg160[p]	2209	Reversible_CLPNS160pp	1
318	clpn161[p]	325	pg161[p]	2210	Reversible_CLPNS161pp	1
1119	glyc[p]	325	pg161[p]	2210	Reversible_CLPNS161pp	1
319	clpn180[p]	326	pg180[p]	2211	Reversible_CLPNS180pp	1
1119	glyc[p]	326	pg180[p]	2211	Reversible_CLPNS180pp	1
320	clpn181[p]	327	pg181[p]	2212	Reversible_CLPNS181pp	1
1119	glyc[p]	327	pg181[p]	2212	Reversible_CLPNS181pp	1
328	cl[p]	329	cl[e]	2213	Reversible_CLtex	0
881	cmp[p]	331	cmp[e]	2214	Reversible_CMPtex	1
333	co2[p]	332	co2[e]	2215	Reversible_CO2tex	1
692	co2	333	co2[p]	2216	Reversible_CO2tpp	1
336	cobalt2[p]	335	cobalt2[e]	2217	Reversible_COBALT2tex	0
345	crncoa	344	bbtcoa	2218	Reversible_CRNBTCT	1
345	crncoa	346	crn	2218	Reversible_CRNBTCT	1
1390	gbbtn	344	bbtcoa	2218	Reversible_CRNBTCT	1
1390	gbbtn	346	crn	2218	Reversible_CRNBTCT	0
1094	crnDcoa	345	crncoa	2219	Reversible_CRNCAR	1
345	crncoa	346	crn	2220	Reversible_CRNCBCT	1
345	crncoa	1095	ctbtcoa	2220	Reversible_CRNCBCT	1
1097	ctbt	346	crn	2220	Reversible_CRNCBCT	0
1097	ctbt	1095	ctbtcoa	2220	Reversible_CRNCBCT	1
109	h2o	345	crncoa	2221	Reversible_CRNCDH	0
1095	ctbtcoa	345	crncoa	2221	Reversible_CRNCDH	1
98	h	347	crn-D[p]	2222	Reversible_CRNDt2rpp	0
98	h	437	h[p]	2222	Reversible_CRNDt2rpp	0
1096	crn-D	347	crn-D[p]	2222	Reversible_CRNDt2rpp	1
1096	crn-D	437	h[p]	2222	Reversible_CRNDt2rpp	0
98	h	348	crn[p]	2223	Reversible_CRNt2rpp	0
98	h	437	h[p]	2223	Reversible_CRNt2rpp	0
346	crn	348	crn[p]	2223	Reversible_CRNt2rpp	1
346	crn	437	h[p]	2223	Reversible_CRNt2rpp	0
348	crn[p]	349	crn[e]	2224	Reversible_CRNtex	1
351	csn[p]	352	csn[e]	2225	Reversible_CSNtex	1
98	h	353	ctbt[p]	2226	Reversible_CTBTt2rpp	0
98	h	437	h[p]	2226	Reversible_CTBTt2rpp	0
1097	ctbt	353	ctbt[p]	2226	Reversible_CTBTt2rpp	1
1097	ctbt	437	h[p]	2226	Reversible_CTBTt2rpp	0
356	cu2[p]	355	cu2[e]	2227	Reversible_CU2tex	0
354	cu[p]	358	cu[e]	2228	Reversible_CUtex	0
360	cyan[p]	361	cyan[e]	2229	Reversible_CYANtex	1
363	cynt[p]	364	cynt[e]	2230	Reversible_CYNTtex	1
1098	cys-D[p]	367	cys-D[e]	2231	Reversible_CYSDtex	1
1099	cys-L[p]	369	cys-L[e]	2232	Reversible_CYStex	1
98	h	372	cytd[p]	2233	Reversible_CYTDt2rpp	0
98	h	437	h[p]	2233	Reversible_CYTDt2rpp	0
371	cytd	372	cytd[p]	2233	Reversible_CYTDt2rpp	1
371	cytd	437	h[p]	2233	Reversible_CYTDt2rpp	0
372	cytd[p]	373	cytd[e]	2234	Reversible_CYTDtex	1

281	adp	135	atp	2235	Reversible_CYTK1	1
281	adp	330	cmp	2235	Reversible_CYTK1	0
980	cdp	135	atp	2235	Reversible_CYTK1	0
980	cdp	330	cmp	2235	Reversible_CYTK1	1
281	adp	135	atp	2236	Reversible_CYTK2	1
281	adp	879	dcmp	2236	Reversible_CYTK2	0
1350	dcdp	135	atp	2236	Reversible_CYTK2	0
1350	dcdp	879	dcmp	2236	Reversible_CYTK2	1
281	adp	135	atp	2237	Reversible_DADK	1
281	adp	884	damp	2237	Reversible_DADK	0
1348	dadp	135	atp	2237	Reversible_DADK	0
1348	dadp	884	damp	2237	Reversible_DADK	1
375	dad-2[p]	376	dad-2[e]	2238	Reversible_DADNtex	1
384	ala-D[p]	385	ala-D[e]	2239	Reversible_DALAtex	1
885	damp[p]	386	damp[e]	2240	Reversible_DAMPtex	1
389	26dap-M	390	26dap-LL	2241	Reversible_DAPE	1
1100	15dap[p]	391	15dap[e]	2242	Reversible_DAPtex	1
1101	dca[p]	394	dca[e]	2243	Reversible_DCAtex	1
880	dcmp[p]	395	dcmp[e]	2244	Reversible_DCMPtex	1
398	dcyt[p]	399	dcyt[e]	2245	Reversible_DCYTtex	1
98	h	402	2ddglcn[p]	2246	Reversible_DDGLCNt2rpp	0
98	h	437	h[p]	2246	Reversible_DDGLCNt2rpp	0
404	2ddglcn	402	2ddglcn[p]	2246	Reversible_DDGLCNt2rpp	1
404	2ddglcn	437	h[p]	2246	Reversible_DDGLCNt2rpp	0
402	2ddglcn[p]	403	2ddglcn[e]	2247	Reversible_DDGLCNtex	1
468	g3p	406	2dh3dgal6p	2248	Reversible_DDPGALA	1
1148	pyr	406	2dh3dgal6p	2248	Reversible_DDPGALA	1
281	adp	135	atp	2249	Reversible_DGK1	1
281	adp	887	dgmp	2249	Reversible_DGK1	0
1349	dgdp	135	atp	2249	Reversible_DGK1	0
1349	dgdp	887	dgmp	2249	Reversible_DGK1	1
888	dgmp[p]	407	dgmp[e]	2250	Reversible_DGMPtex	1
408	dgsn[p]	409	dgsn[e]	2251	Reversible_DGSNtex	1
414	dha[p]	413	dha[e]	2252	Reversible_DHAtex	1
412	dha	414	dha[p]	2253	Reversible_DHAtpp	1
98	h	415	23ddhb	2254	Reversible_DHBD	0
98	h	856	nad	2254	Reversible_DHBD	0
416	23dhb	415	23ddhb	2254	Reversible_DHBD	1
416	23dhb	856	nad	2254	Reversible_DHBD	0
870	nadh	415	23ddhb	2254	Reversible_DHBD	0
870	nadh	856	nad	2254	Reversible_DHBD	1
459	nadp	98	h	2255	Reversible_DHFR	0
459	nadp	421	dhf	2255	Reversible_DHFR	0
459	nadp	871	nadph	2255	Reversible_DHFR	1
1403	thf	98	h	2255	Reversible_DHFR	0
1403	thf	421	dhf	2255	Reversible_DHFR	1
1403	thf	871	nadph	2255	Reversible_DHFR	0
98	h	109	h2o	2256	Reversible_DHORTS	0
98	h	424	dhor-S	2256	Reversible_DHORTS	0
1102	cbasp	109	h2o	2256	Reversible_DHORTS	0
1102	cbasp	424	dhor-S	2256	Reversible_DHORTS	1
1103	dhmptp	429	ahdt	2257	Reversible_DHPTPE	1
877	dimp[p]	432	dimp[e]	2258	Reversible_DIMPtex	1
433	din[p]	434	din[e]	2259	Reversible_DINStex	1
98	h	437	h[p]	2260	Reversible_D-LACt2pp	0
98	h	1104	lac-D[p]	2260	Reversible_D-LACt2pp	0

748	lac-D	437	h[p]	2260	Reversible_D-LACt2pp	0
748	lac-D	1104	lac-D[p]	2260	Reversible_D-LACt2pp	1
1104	lac-D[p]	438	lac-D[e]	2261	Reversible_D-LACtex	1
447	dmso[p]	448	dmso[e]	2262	Reversible_DMSOtex	1
1105	dms[p]	449	dms[e]	2263	Reversible_DMStex	1
107	dopa[p]	452	dopa[e]	2264	Reversible_DOPAtex	1
98	h	459	nadp	2265	Reversible_DSERDHr	0
98	h	988	ser-D	2265	Reversible_DSERDHr	0
871	nadph	459	nadp	2265	Reversible_DSERDHr	1
871	nadph	988	ser-D	2265	Reversible_DSERDHr	0
1106	2amsa	459	nadp	2265	Reversible_DSERDHr	0
1106	2amsa	988	ser-D	2265	Reversible_DSERDHr	1
1107	ser-D[p]	460	ser-D[e]	2266	Reversible_DSERtex	1
281	adp	135	atp	2267	Reversible_DTMPK	1
281	adp	882	dtmp	2267	Reversible_DTMPK	0
1239	dtdp	135	atp	2267	Reversible_DTMPK	0
1239	dtdp	882	dtmp	2267	Reversible_DTMPK	1
883	dtmp[p]	461	dtmp[e]	2268	Reversible_DTMPtex	1
878	dump[p]	462	dump[e]	2269	Reversible_DUMPtex	1
950	2dr1p	463	duri	2270	Reversible_DURIPP	1
950	2dr1p	962	pi	2270	Reversible_DURIPP	0
1552	ura	463	duri	2270	Reversible_DURIPP	1
1552	ura	962	pi	2270	Reversible_DURIPP	0
464	duri[p]	465	duri[e]	2271	Reversible_DURItex	1
98	h	109	h2o	2272	Reversible_E4PD	0
98	h	405	e4p	2272	Reversible_E4PD	0
98	h	856	nad	2272	Reversible_E4PD	0
870	nadh	109	h2o	2272	Reversible_E4PD	0
870	nadh	405	e4p	2272	Reversible_E4PD	0
870	nadh	856	nad	2272	Reversible_E4PD	1
930	4per	109	h2o	2272	Reversible_E4PD	0
930	4per	405	e4p	2272	Reversible_E4PD	1
930	4per	856	nad	2272	Reversible_E4PD	0
109	h2o	475	3hbcoa	2273	Reversible_ECOAH1	0
1076	b2coa	475	3hbcoa	2273	Reversible_ECOAH1	1
109	h2o	476	3hhcoa	2274	Reversible_ECOAH2	0
1556	hx2coa	476	3hhcoa	2274	Reversible_ECOAH2	1
109	h2o	477	3hocoa	2275	Reversible_ECOAH3	0
1557	oc2coa	477	3hocoa	2275	Reversible_ECOAH3	1
109	h2o	478	3hdcoa	2276	Reversible_ECOAH4	0
1077	dc2coa	478	3hdcoa	2276	Reversible_ECOAH4	1
109	h2o	479	3hddcoa	2277	Reversible_ECOAH5	0
1078	dd2coa	479	3hddcoa	2277	Reversible_ECOAH5	1
109	h2o	480	3htdcoa	2278	Reversible_ECOAH6	0
1354	td2coa	480	3htdcoa	2278	Reversible_ECOAH6	1
109	h2o	481	3hhdcoa	2279	Reversible_ECOAH7	0
1291	hdd2coa	481	3hhdcoa	2279	Reversible_ECOAH7	1
109	h2o	482	3hodcoa	2280	Reversible_ECOAH8	0
1334	od2coa	482	3hodcoa	2280	Reversible_ECOAH8	1
109	h2o	489	2pg	2281	Reversible_ENO	0
959	pep	489	2pg	2281	Reversible_ENO	1
494	etha[p]	495	etha[e]	2282	Reversible_ETHAtex	1
1108	ethso3[p]	496	ethso3[e]	2283	Reversible_ETHSO3tex	1
98	h	437	h[p]	2284	Reversible_ETOHt2rpp	0
98	h	497	etoh[p]	2284	Reversible_ETOHt2rpp	0
225	etoh	437	h[p]	2284	Reversible_ETOHt2rpp	0

225	etoh	497	etoh[p]	2284	Reversible_ETOHt2rpp	1
497	etoh[p]	498	etoh[e]	2285	Reversible_ETOHtex	1
412	dha	499	f6p	2286	Reversible_F6PA	1
468	g3p	499	f6p	2286	Reversible_F6PA	1
500	f6p[p]	501	f6p[e]	2287	Reversible_F6Ptex	1
98	h	502	hmgth	2288	Reversible_FALDH2	0
98	h	856	nad	2288	Reversible_FALDH2	0
870	nadh	502	hmgth	2288	Reversible_FALDH2	0
870	nadh	856	nad	2288	Reversible_FALDH2	1
990	Sfglutth	502	hmgth	2288	Reversible_FALDH2	1
990	Sfglutth	856	nad	2288	Reversible_FALDH2	0
504	fald[p]	503	fald[e]	2289	Reversible_FALDtex	1
505	fald	504	fald[p]	2290	Reversible_FALDtpp	1
502	hmgth	505	fald	2291	Reversible_FALGTHLs	1
502	hmgth	751	gthrd	2291	Reversible_FALGTHLs	1
468	g3p	506	fdp	2292	Reversible_FBA	1
826	dhap	506	fdp	2292	Reversible_FBA	1
1109	fcl-L	507	fuc-L	2293	Reversible_FCI	1
826	dhap	508	fc1p	2294	Reversible_FCLPA	1
1422	lald-L	508	fc1p	2294	Reversible_FCLPA	1
514	fe2[p]	515	fe2[e]	2295	Reversible_FE2tex	0
1110	fe3[p]	519	fe3[e]	2296	Reversible_FE3tex	0
510	for[p]	539	for[e]	2297	Reversible_FORtex	1
568	g6p	109	h2o	2298	Reversible_FRULYSDG	0
568	g6p	541	frulysp	2298	Reversible_FRULYSDG	1
1425	lys-L	109	h2o	2298	Reversible_FRULYSDG	0
1425	lys-L	541	frulysp	2298	Reversible_FRULYSDG	1
1111	frulys	542	psclys	2299	Reversible_FRULYSE	1
543	frulys[p]	544	frulys[e]	2300	Reversible_FRULYStex	1
545	fru[p]	546	fru[e]	2301	Reversible_FRUtex	1
98	h	437	h[p]	2302	Reversible_FRUURt2rpp	0
98	h	547	fruur[p]	2302	Reversible_FRUURt2rpp	0
1112	fruur	437	h[p]	2302	Reversible_FRUURt2rpp	0
1112	fruur	547	fruur[p]	2302	Reversible_FRUURt2rpp	1
547	fruur[p]	548	fruur[e]	2303	Reversible_FRUURtex	1
550	fuc-L[p]	549	fuc-L[e]	2304	Reversible_FUCtex	1
98	h	437	h[p]	2305	Reversible_FUCtpp	0
98	h	550	fuc-L[p]	2305	Reversible_FUCtpp	0
507	fuc-L	437	h[p]	2305	Reversible_FUCtpp	0
507	fuc-L	550	fuc-L[p]	2305	Reversible_FUCtpp	1
811	mal-L	109	h2o	2306	Reversible_FUM	0
811	mal-L	540	fum	2306	Reversible_FUM	1
551	fum[p]	552	fum[e]	2307	Reversible_FUMtex	1
553	g1p[p]	554	g1p[e]	2308	Reversible_G1Ptex	1
946	5aop	556	glu1sa	2309	Reversible_G1SAT	1
655	g3pc[p]	559	g3pc[e]	2310	Reversible_G3PCtex	1
98	h	459	nadp	2311	Reversible_G3PD2	0
98	h	558	glyc3p	2311	Reversible_G3PD2	0
826	dhap	459	nadp	2311	Reversible_G3PD2	0
826	dhap	558	glyc3p	2311	Reversible_G3PD2	1
871	nadph	459	nadp	2311	Reversible_G3PD2	1
871	nadph	558	glyc3p	2311	Reversible_G3PD2	0
657	g3pe[p]	561	g3pe[e]	2312	Reversible_G3PEtex	1
661	g3pg[p]	562	g3pg[e]	2313	Reversible_G3PGtex	1
663	g3pi[p]	563	g3pi[e]	2314	Reversible_G3PItex	1
659	g3ps[p]	564	g3ps[e]	2315	Reversible_G3PStex	1

98	h	459	nadp	2316	Reversible_G6PDH2r	0
98	h	568	g6p	2316	Reversible_G6PDH2r	0
871	nadph	459	nadp	2316	Reversible_G6PDH2r	1
871	nadph	568	g6p	2316	Reversible_G6PDH2r	0
933	6pgl	459	nadp	2316	Reversible_G6PDH2r	0
933	6pgl	568	g6p	2316	Reversible_G6PDH2r	1
569	g6p[p]	570	g6p[e]	2317	Reversible_G6Ptex	1
571	gal1p[p]	572	gal1p[e]	2318	Reversible_GAL1Ptex	1
583	gal-bD[p]	573	gal-bD[e]	2319	Reversible_GALBDtex	1
577	galctn-L[p]	578	galctn-L[e]	2320	Reversible_GALCTNLtex	1
579	galctn-D[p]	580	galctn-D[e]	2321	Reversible_GALCTNtex	1
98	h	437	h[p]	2322	Reversible_GALCTt2rpp	0
98	h	581	galct-D[p]	2322	Reversible_GALCTt2rpp	0
574	galct-D	437	h[p]	2322	Reversible_GALCTt2rpp	0
574	galct-D	581	galct-D[p]	2322	Reversible_GALCTt2rpp	1
581	galct-D[p]	582	galct-D[e]	2323	Reversible_GALCTtex	1
98	h	135	atp	2324	Reversible_GALKr	0
98	h	1284	gal	2324	Reversible_GALKr	0
281	adp	135	atp	2324	Reversible_GALKr	1
281	adp	1284	gal	2324	Reversible_GALKr	0
1047	gal1p	135	atp	2324	Reversible_GALKr	0
1047	gal1p	1284	gal	2324	Reversible_GALKr	1
585	gal[p]	586	gal[e]	2325	Reversible_GALtex	1
587	galt[p]	588	galt[e]	2326	Reversible_GALTtex	1
98	h	437	h[p]	2327	Reversible_GALURt2rpp	0
98	h	590	galur[p]	2327	Reversible_GALURt2rpp	0
680	galur	437	h[p]	2327	Reversible_GALURt2rpp	0
680	galur	590	galur[p]	2327	Reversible_GALURt2rpp	1
590	galur[p]	591	galur[e]	2328	Reversible_GALURtex	1
592	gam6p[p]	593	gam6p[e]	2329	Reversible_GAMAN6Ptex	1
594	gam[p]	595	gam[e]	2330	Reversible_GAMtex	1
98	h	468	g3p	2331	Reversible_GAPD	0
98	h	856	nad	2331	Reversible_GAPD	0
98	h	962	pi	2331	Reversible_GAPD	0
870	nadh	468	g3p	2331	Reversible_GAPD	0
870	nadh	856	nad	2331	Reversible_GAPD	1
870	nadh	962	pi	2331	Reversible_GAPD	0
1113	13dpg	468	g3p	2331	Reversible_GAPD	1
1113	13dpg	856	nad	2331	Reversible_GAPD	0
1113	13dpg	962	pi	2331	Reversible_GAPD	0
98	h	210	10fthf	2332	Reversible_GARFT	0
98	h	1402	gar	2332	Reversible_GARFT	0
1114	fgam	210	10fthf	2332	Reversible_GARFT	1
1114	fgam	1402	gar	2332	Reversible_GARFT	1
1403	thf	210	10fthf	2332	Reversible_GARFT	1
1403	thf	1402	gar	2332	Reversible_GARFT	0
1115	gbbtn[p]	596	gbbtn[e]	2333	Reversible_GBBTNtex	1
1116	gdp[p]	600	gdp[e]	2334	Reversible_GDPtex	1
98	h	109	h2o	2335	Reversible_GGGABADr	0
98	h	459	nadp	2335	Reversible_GGGABADr	0
98	h	601	ggbutal	2335	Reversible_GGGABADr	0
602	gg4abut	109	h2o	2335	Reversible_GGGABADr	0
602	gg4abut	459	nadp	2335	Reversible_GGGABADr	0
602	gg4abut	601	ggbutal	2335	Reversible_GGGABADr	1
871	nadph	109	h2o	2335	Reversible_GGGABADr	0
871	nadph	459	nadp	2335	Reversible_GGGABADr	1

871	nadph	601	ggbutal	2335	Reversible_GGGABADr	0
109	h2o	604	ser-L	2336	Reversible_GHMT2r	0
109	h2o	1403	thf	2336	Reversible_GHMT2r	0
642	gly	604	ser-L	2336	Reversible_GHMT2r	1
642	gly	1403	thf	2336	Reversible_GHMT2r	0
848	mlthf	604	ser-L	2336	Reversible_GHMT2r	1
848	mlthf	1403	thf	2336	Reversible_GHMT2r	1
281	adp	135	atp	2337	Reversible_GK1	1
281	adp	650	gmp	2337	Reversible_GK1	0
798	gdp	135	atp	2337	Reversible_GK1	0
798	gdp	650	gmp	2337	Reversible_GK1	1
927	coa	128	accoa	2338	Reversible_GLCATr	1
927	coa	1064	glc-D	2338	Reversible_GLCATr	0
1117	acglc-D	128	accoa	2338	Reversible_GLCATr	1
1117	acglc-D	1064	glc-D	2338	Reversible_GLCATr	1
98	h	437	h[p]	2339	Reversible_GLCNt2rpp	0
98	h	607	glcn[p]	2339	Reversible_GLCNt2rpp	0
1073	glcn	437	h[p]	2339	Reversible_GLCNt2rpp	0
1073	glcn	607	glcn[p]	2339	Reversible_GLCNt2rpp	1
607	glcn[p]	608	glcn[e]	2340	Reversible_GLCNtex	1
98	h	437	h[p]	2341	Reversible_GLCRt2rpp	0
98	h	612	glcr[p]	2341	Reversible_GLCRt2rpp	0
611	glcr	437	h[p]	2341	Reversible_GLCRt2rpp	0
611	glcr	612	glcr[p]	2341	Reversible_GLCRt2rpp	1
612	glcr[p]	613	glcr[e]	2342	Reversible_GLCRtex	1
606	glc-D[p]	615	glc-D[e]	2343	Reversible_GLCtex	1
681	glcur1p[p]	619	glcur1p[e]	2344	Reversible_GLCUR1Ptex	1
98	h	437	h[p]	2345	Reversible_GLCURt2rpp	0
98	h	620	glcur[p]	2345	Reversible_GLCURt2rpp	0
679	glcur	437	h[p]	2345	Reversible_GLCURt2rpp	0
679	glcur	620	glcur[p]	2345	Reversible_GLCURt2rpp	1
620	glcur[p]	621	glcur[e]	2346	Reversible_GLCURtex	1
626	gln-L[p]	622	gln-L[e]	2347	Reversible_GLNtex	1
98	h	623	galt1p	2348	Reversible_GLTPD	0
98	h	856	nad	2348	Reversible_GLTPD	0
870	nadh	623	galt1p	2348	Reversible_GLTPD	0
870	nadh	856	nad	2348	Reversible_GLTPD	1
1470	tag6p-D	623	galt1p	2348	Reversible_GLTPD	1
1470	tag6p-D	856	nad	2348	Reversible_GLTPD	0
125	4abut[p]	123	4abut	2349	Reversible_GLUABUTt7pp	1
125	4abut[p]	628	glu-L[p]	2349	Reversible_GLUABUTt7pp	0
624	glu-L	123	4abut	2349	Reversible_GLUABUTt7pp	0
624	glu-L	628	glu-L[p]	2349	Reversible_GLUABUTt7pp	1
98	h	109	h2o	2350	Reversible_GLUDy	0
98	h	459	nadp	2350	Reversible_GLUDy	0
98	h	624	glu-L	2350	Reversible_GLUDy	0
213	akg	109	h2o	2350	Reversible_GLUDy	0
213	akg	459	nadp	2350	Reversible_GLUDy	0
213	akg	624	glu-L	2350	Reversible_GLUDy	1
871	nadph	109	h2o	2350	Reversible_GLUDy	0
871	nadph	459	nadp	2350	Reversible_GLUDy	1
871	nadph	624	glu-L	2350	Reversible_GLUDy	0
1160	nh4	109	h2o	2350	Reversible_GLUDy	0
1160	nh4	459	nadp	2350	Reversible_GLUDy	0
1160	nh4	624	glu-L	2350	Reversible_GLUDy	0
624	glu-L	627	glu-D	2351	Reversible_GLUR	1

98	h	437	h[p]	2352	Reversible_GLUt2rpp	0
98	h	628	glu-L[p]	2352	Reversible_GLUt2rpp	0
624	glu-L	437	h[p]	2352	Reversible_GLUt2rpp	0
624	glu-L	628	glu-L[p]	2352	Reversible_GLUt2rpp	1
628	glu-L[p]	629	glu-L[e]	2353	Reversible_GLUtex	1
633	glyald[p]	632	glyald[e]	2354	Reversible_GLYALDtex	1
224	glyald	633	glyald[p]	2355	Reversible_GLYALDtpp	1
251	2aobut	128	accoa	2356	Reversible_GLYAT	1
251	2aobut	642	gly	2356	Reversible_GLYAT	1
927	coa	128	accoa	2356	Reversible_GLYAT	1
927	coa	642	gly	2356	Reversible_GLYAT	0
634	glyb[p]	635	glyb[e]	2357	Reversible_GLYBtex	1
557	glyc2p[p]	636	glyc2p[e]	2358	Reversible_GLYC2Ptex	1
637	glyc3p[p]	638	glyc3p[e]	2359	Reversible_GLYC3Ptex	1
98	h	437	h[p]	2360	Reversible_GLYCAt2rpp	0
98	h	639	glyc-R[p]	2360	Reversible_GLYCAt2rpp	0
1118	glyc-R	437	h[p]	2360	Reversible_GLYCAt2rpp	0
1118	glyc-R	639	glyc-R[p]	2360	Reversible_GLYCAt2rpp	1
639	glyc-R[p]	640	glyc-R[e]	2361	Reversible_GLYCAtex	1
98	h	437	h[p]	2362	Reversible_GLYCLTt2rpp	0
98	h	643	glyclt[p]	2362	Reversible_GLYCLTt2rpp	0
646	glyclt	437	h[p]	2362	Reversible_GLYCLTt2rpp	0
646	glyclt	643	glyclt[p]	2362	Reversible_GLYCLTt2rpp	1
643	glyclt[p]	644	glyclt[e]	2363	Reversible_GLYCLTtex	1
1119	glyc[p]	645	glyc[e]	2364	Reversible_GLYCtex	1
1119	glyc[p]	641	glyc	2365	Reversible_GLYCtpp	1
647	gly[p]	648	gly[e]	2366	Reversible_GLYtex	1
889	gmp[p]	651	gmp[e]	2367	Reversible_GMPtex	1
666	gsn[p]	667	gsn[e]	2368	Reversible_GSNtex	1
459	nadp	98	h	2369	Reversible_GTHOr	0
459	nadp	669	gthox	2369	Reversible_GTHOr	0
459	nadp	871	nadph	2369	Reversible_GTHOr	1
751	gthrd	98	h	2369	Reversible_GTHOr	0
751	gthrd	669	gthox	2369	Reversible_GTHOr	1
751	gthrd	871	nadph	2369	Reversible_GTHOr	0
1120	gthox[p]	670	gthox[e]	2370	Reversible_GTHOXtex	1
671	gthrd[p]	672	gthrd[e]	2371	Reversible_GTHRDtex	1
891	gtp[p]	675	gtp[e]	2372	Reversible_GTPtex	1
677	gua[p]	678	gua[e]	2373	Reversible_GUAtex	1
676	gua	677	gua[p]	2374	Reversible_GUAtpp	1
1112	fruur	679	glcur	2375	Reversible_GUI1	1
1121	tagur	680	galur	2376	Reversible_GUI2	1
1122	h2o2[p]	682	h2o2[e]	2377	Reversible_H2O2tex	0
684	h2o[p]	683	h2o[e]	2378	Reversible_H2Otex	0
109	h2o	684	h2o[p]	2379	Reversible_H2Otpp	0
1123	h2s[p]	686	h2s[e]	2380	Reversible_H2Stex	0
688	h2[p]	687	h2[e]	2381	Reversible_H2tex	0
1124	h2	688	h2[p]	2382	Reversible_H2tpp	0
79	3hcinnm	437	h[p]	2383	Reversible_HCINNMt2rpp	0
79	3hcinnm	690	3hcinnm[p]	2383	Reversible_HCINNMt2rpp	1
98	h	437	h[p]	2383	Reversible_HCINNMt2rpp	0
98	h	690	3hcinnm[p]	2383	Reversible_HCINNMt2rpp	0
690	3hcinnm[p]	691	3hcinnm[e]	2384	Reversible_HCINNMtex	1
98	h	109	h2o	2385	Reversible_HCO3E	0
98	h	692	co2	2385	Reversible_HCO3E	0
1560	hco3	109	h2o	2385	Reversible_HCO3E	0

1560	hco3	692	co2	2385	Reversible_HCO3E	1
98	h	437	h[p]	2386	Reversible_HEXt2rpp	0
98	h	1128	hxa[p]	2386	Reversible_HEXt2rpp	0
1418	hxa	437	h[p]	2386	Reversible_HEXt2rpp	0
1418	hxa	1128	hxa[p]	2386	Reversible_HEXt2rpp	1
1125	hg2[p]	700	hg2[e]	2387	Reversible_HG2tex	0
98	h	437	h[p]	2388	Reversible_HISt2rpp	0
98	h	1126	his-L[p]	2388	Reversible_HISt2rpp	0
1413	his-L	437	h[p]	2388	Reversible_HISt2rpp	0
1413	his-L	1126	his-L[p]	2388	Reversible_HISt2rpp	1
1126	his-L[p]	701	his-L[e]	2389	Reversible_HIStex	1
1127	hom-L[p]	703	hom-L[e]	2390	Reversible_HOMtex	1
80	3hpppn	437	h[p]	2391	Reversible_HPPPNt2rpp	0
80	3hpppn	707	3hpppn[p]	2391	Reversible_HPPPNt2rpp	1
98	h	437	h[p]	2391	Reversible_HPPPNt2rpp	0
98	h	707	3hpppn[p]	2391	Reversible_HPPPNt2rpp	0
707	3hpppn[p]	708	3hpppn[e]	2392	Reversible_HPPPNtex	1
1030	2h3oppan	709	hpyr	2393	Reversible_HPYRI	1
98	h	459	nadp	2394	Reversible_HSDy	0
98	h	710	hom-L	2394	Reversible_HSDy	0
268	aspsa	459	nadp	2394	Reversible_HSDy	0
268	aspsa	710	hom-L	2394	Reversible_HSDy	1
871	nadph	459	nadp	2394	Reversible_HSDy	1
871	nadph	710	hom-L	2394	Reversible_HSDy	0
437	h[p]	711	h[e]	2395	Reversible_Htex	0
1128	hxa[p]	712	hxa[e]	2396	Reversible_HXAtex	1
715	hxan[p]	714	hxan[e]	2397	Reversible_HYXNtex	1
713	hxan	715	hxan[p]	2398	Reversible_HYXNtpp	1
213	akg	459	nadp	2399	Reversible_ICDHyr	0
213	akg	716	icit	2399	Reversible_ICDHyr	1
692	co2	459	nadp	2399	Reversible_ICDHyr	0
692	co2	716	icit	2399	Reversible_ICDHyr	0
871	nadph	459	nadp	2399	Reversible_ICDHyr	1
871	nadph	716	icit	2399	Reversible_ICDHyr	0
991	ichor	173	chor	2400	Reversible_ICHORS	1
856	nad	98	h	2401	Reversible_IDOND	0
856	nad	113	5dglcn	2401	Reversible_IDOND	0
856	nad	870	nadh	2401	Reversible_IDOND	1
1129	idon-L	98	h	2401	Reversible_IDOND	0
1129	idon-L	113	5dglcn	2401	Reversible_IDOND	1
1129	idon-L	870	nadh	2401	Reversible_IDOND	0
98	h	437	h[p]	2402	Reversible_IDONt2rpp	0
98	h	1130	idon-L[p]	2402	Reversible_IDONt2rpp	0
1129	idon-L	437	h[p]	2402	Reversible_IDONt2rpp	0
1129	idon-L	1130	idon-L[p]	2402	Reversible_IDONt2rpp	1
1130	idon-L[p]	717	idon-L[e]	2403	Reversible_IDONtex	1
98	h	437	h[p]	2404	Reversible_ILEt2rpp	0
98	h	1132	ile-L[p]	2404	Reversible_ILEt2rpp	0
1419	ile-L	437	h[p]	2404	Reversible_ILEt2rpp	0
1419	ile-L	1132	ile-L[p]	2404	Reversible_ILEt2rpp	1
624	glu-L	213	akg	2405	Reversible_ILETA	1
624	glu-L	1419	ile-L	2405	Reversible_ILETA	0
1131	3mop	213	akg	2405	Reversible_ILETA	0
1131	3mop	1419	ile-L	2405	Reversible_ILETA	1
1132	ile-L[p]	720	ile-L[e]	2406	Reversible_ILEtex	1
1083	fprica	109	h2o	2407	Reversible_IMPC	0

1083	fprica	1287	imp	2407	Reversible_IMPC	1
1133	imp[p]	721	imp[e]	2408	Reversible_IMPtex	1
98	h	437	h[p]	2409	Reversible_INDOLEt2rpp	0
98	h	1134	indole[p]	2409	Reversible_INDOLEt2rpp	0
1028	indole	437	h[p]	2409	Reversible_INDOLEt2rpp	0
1028	indole	1134	indole[p]	2409	Reversible_INDOLEt2rpp	1
1134	indole[p]	722	indole[e]	2410	Reversible_INDOLEtex	1
98	h	437	h[p]	2411	Reversible_INSt2rpp	0
98	h	1135	ins[p]	2411	Reversible_INSt2rpp	0
967	ins	437	h[p]	2411	Reversible_INSt2rpp	0
967	ins	1135	ins[p]	2411	Reversible_INSt2rpp	1
1135	ins[p]	724	ins[e]	2412	Reversible_INStex	1
723	inost[p]	725	inost[e]	2413	Reversible_INSTtex	1
444	dmpp	726	ipdp	2414	Reversible_IPDDI	1
109	h2o	727	3c2hmp	2415	Reversible_IPPMIa	0
728	2ippm	727	3c2hmp	2415	Reversible_IPPMIa	1
1136	3c3hmp	109	h2o	2416	Reversible_IPPMIb	0
1136	3c3hmp	728	2ippm	2416	Reversible_IPPMIb	1
1137	isetac[p]	730	isetac[e]	2417	Reversible_ISETACtex	1
98	h	410	23dhmb	2418	Reversible_KARA1	0
98	h	459	nadp	2418	Reversible_KARA1	0
871	nadph	410	23dhmb	2418	Reversible_KARA1	0
871	nadph	459	nadp	2418	Reversible_KARA1	1
1138	alac-S	410	23dhmb	2418	Reversible_KARA1	1
1138	alac-S	459	nadp	2418	Reversible_KARA1	0
411	23dhmp	98	h	2419	Reversible_KARA2	0
411	23dhmp	732	2ahbut	2419	Reversible_KARA2	1
411	23dhmp	871	nadph	2419	Reversible_KARA2	0
459	nadp	98	h	2419	Reversible_KARA2	0
459	nadp	732	2ahbut	2419	Reversible_KARA2	0
459	nadp	871	nadph	2419	Reversible_KARA2	1
1139	k[p]	744	k[e]	2420	Reversible_Ktex	0
856	nad	98	h	2421	Reversible_LCARR	0
856	nad	870	nadh	2421	Reversible_LCARR	1
856	nad	1300	lald-D	2421	Reversible_LCARR	0
1069	12ppd-R	98	h	2421	Reversible_LCARR	0
1069	12ppd-R	870	nadh	2421	Reversible_LCARR	0
1069	12ppd-R	1300	lald-D	2421	Reversible_LCARR	1
856	nad	98	h	2422	Reversible_LCARS	0
856	nad	870	nadh	2422	Reversible_LCARS	1
856	nad	1422	lald-L	2422	Reversible_LCARS	0
1070	12ppd-S	98	h	2422	Reversible_LCARS	0
1070	12ppd-S	870	nadh	2422	Reversible_LCARS	0
1070	12ppd-S	1422	lald-L	2422	Reversible_LCARS	1
1140	lcts[p]	747	lcts[e]	2423	Reversible_LCTStex	1
98	h	437	h[p]	2424	Reversible_LCTStpp	0
98	h	1140	lcts[p]	2424	Reversible_LCTStpp	0
1421	lcts	437	h[p]	2424	Reversible_LCTStpp	0
1421	lcts	1140	lcts[p]	2424	Reversible_LCTStpp	1
98	h	748	lac-D	2425	Reversible_LDH_D	0
98	h	856	nad	2425	Reversible_LDH_D	0
870	nadh	748	lac-D	2425	Reversible_LDH_D	0
870	nadh	856	nad	2425	Reversible_LDH_D	1
1148	pyr	748	lac-D	2425	Reversible_LDH_D	1
1148	pyr	856	nad	2425	Reversible_LDH_D	0
98	h	437	h[p]	2426	Reversible_LEUt2rpp	0

98	h	1141	leu-L[p]	2426	Reversible_LEUt2rpp	0
1423	leu-L	437	h[p]	2426	Reversible_LEUt2rpp	0
1423	leu-L	1141	leu-L[p]	2426	Reversible_LEUt2rpp	1
1141	leu-L[p]	750	leu-L[e]	2427	Reversible_LEUtex	1
98	h	437	h[p]	2428	Reversible_L-LACt2rpp	0
98	h	1142	lac-L[p]	2428	Reversible_L-LACt2rpp	0
754	lac-L	437	h[p]	2428	Reversible_L-LACt2rpp	0
754	lac-L	1142	lac-L[p]	2428	Reversible_L-LACt2rpp	1
1142	lac-L[p]	755	lac-L[e]	2429	Reversible_L-LACtex	1
98	h	459	nadp	2430	Reversible_LSERDHr	0
98	h	604	ser-L	2430	Reversible_LSERDHr	0
871	nadph	459	nadp	2430	Reversible_LSERDHr	1
871	nadph	604	ser-L	2430	Reversible_LSERDHr	0
1106	2amsa	459	nadp	2430	Reversible_LSERDHr	0
1106	2amsa	604	ser-L	2430	Reversible_LSERDHr	1
1143	lys-L[p]	785	lys-L[e]	2431	Reversible_LYStex	1
1144	lyx-L[p]	787	lyx-L[e]	2432	Reversible_LYXtex	1
98	h	788	mnl1p	2433	Reversible_M1PD	0
98	h	856	nad	2433	Reversible_M1PD	0
499	f6p	788	mnl1p	2433	Reversible_M1PD	1
499	f6p	856	nad	2433	Reversible_M1PD	0
870	nadh	788	mnl1p	2433	Reversible_M1PD	0
870	nadh	856	nad	2433	Reversible_M1PD	1
1145	mal-D[p]	790	mal-D[e]	2434	Reversible_MALDtex	1
927	coa	128	accoa	2435	Reversible_MALTATr	1
927	coa	241	malt	2435	Reversible_MALTATr	0
1146	acmalt	128	accoa	2435	Reversible_MALTATr	1
1146	acmalt	241	malt	2435	Reversible_MALTATr	1
1147	mal-L[p]	791	mal-L[e]	2436	Reversible_MALtex	1
499	f6p	799	man6p	2437	Reversible_MAN6PI	1
800	man6p[p]	801	man6p[e]	2438	Reversible_MAN6Ptex	1
98	h	802	mana	2439	Reversible_MANAO	0
98	h	856	nad	2439	Reversible_MANAO	0
870	nadh	802	mana	2439	Reversible_MANAO	0
870	nadh	856	nad	2439	Reversible_MANAO	1
1112	fruur	802	mana	2439	Reversible_MANAO	1
1112	fruur	856	nad	2439	Reversible_MANAO	0
803	manglyc[p]	804	manglyc[e]	2440	Reversible_MANGLYCtex	1
805	man[p]	806	man[e]	2441	Reversible_MANtex	1
1008	succ	808	micit	2442	Reversible_MCITL2	1
1148	pyr	808	micit	2442	Reversible_MCITL2	1
927	coa	118	ACP	2443	Reversible_MCOATA	0
927	coa	1426	malcoa	2443	Reversible_MCOATA	1
1363	malACP	118	ACP	2443	Reversible_MCOATA	1
1363	malACP	1426	malcoa	2443	Reversible_MCOATA	1
98	h	811	mal-L	2444	Reversible_MDH	0
98	h	856	nad	2444	Reversible_MDH	0
870	nadh	811	mal-L	2444	Reversible_MDH	0
870	nadh	856	nad	2444	Reversible_MDH	1
1318	oaa	811	mal-L	2444	Reversible_MDH	1
1318	oaa	856	nad	2444	Reversible_MDH	0
1149	melib[p]	815	melib[e]	2445	Reversible_MELIBtex	1
1150	met-D[p]	817	met-D[e]	2446	Reversible_METDtex	1
1151	metsox-S-L[p]	819	metsox-S-L[e]	2447	Reversible_METSOX1tex	1
1152	metsox-R-L[p]	820	metsox-R-L[e]	2448	Reversible_METSOX2tex	1
1153	met-L[p]	823	met-L[e]	2449	Reversible_METtex	1

437	h[p]	98	h	2450	Reversible_MG2t3_2pp	0
437	h[p]	825	mg2[p]	2450	Reversible_MG2t3_2pp	0
1320	mg2	98	h	2450	Reversible_MG2t3_2pp	0
1320	mg2	825	mg2[p]	2450	Reversible_MG2t3_2pp	0
825	mg2[p]	824	mg2[e]	2451	Reversible_MG2tex	0
589	g1p	833	maltpt	2452	Reversible_MLTP1	1
589	g1p	962	pi	2452	Reversible_MLTP1	0
1380	maltttr	833	maltpt	2452	Reversible_MLTP1	1
1380	maltttr	962	pi	2452	Reversible_MLTP1	0
589	g1p	834	malthx	2453	Reversible_MLTP2	1
589	g1p	962	pi	2453	Reversible_MLTP2	0
833	maltpt	834	malthx	2453	Reversible_MLTP2	1
833	maltpt	962	pi	2453	Reversible_MLTP2	0
589	g1p	835	malthp	2454	Reversible_MLTP3	1
589	g1p	962	pi	2454	Reversible_MLTP3	0
834	malthx	835	malthp	2454	Reversible_MLTP3	1
834	malthx	962	pi	2454	Reversible_MLTP3	0
1154	mmcoa-S	836	mmcoa-R	2455	Reversible_MME	1
1155	mmet[p]	837	mmet[e]	2456	Reversible_MMETtex	1
840	mnl[p]	841	mnl[e]	2457	Reversible_MNLtex	1
839	mn2[p]	842	mn2[e]	2458	Reversible_MNtex	0
1156	mobd[p]	844	mobd[e]	2459	Reversible_MOBDtex	1
1157	mso3[p]	846	mso3[e]	2460	Reversible_MSO3tex	1
98	h	109	h2o	2461	Reversible_MTHFC	0
98	h	1158	methf	2461	Reversible_MTHFC	0
210	10fthf	109	h2o	2461	Reversible_MTHFC	0
210	10fthf	1158	methf	2461	Reversible_MTHFC	1
871	nadph	459	nadp	2462	Reversible_MTHFD	1
871	nadph	848	mlthf	2462	Reversible_MTHFD	0
1158	methf	459	nadp	2462	Reversible_MTHFD	0
1158	methf	848	mlthf	2462	Reversible_MTHFD	1
812	5mdru1p	849	5mdr1p	2463	Reversible_MTRI	1
853	n2o[p]	852	n2o[e]	2464	Reversible_N2Otex	0
1159	n2o	853	n2o[p]	2465	Reversible_N2Otpp	0
855	nac[p]	854	nac[e]	2466	Reversible_NACtex	1
941	na1[p]	857	na1[e]	2467	Reversible_NAtex	0
281	adp	135	atp	2468	Reversible_NDPK1	1
281	adp	798	gdp	2468	Reversible_NDPK1	0
673	gtp	135	atp	2468	Reversible_NDPK1	0
673	gtp	798	gdp	2468	Reversible_NDPK1	1
281	adp	135	atp	2469	Reversible_NDPK2	1
281	adp	1440	udp	2469	Reversible_NDPK2	0
1447	utp	135	atp	2469	Reversible_NDPK2	0
1447	utp	1440	udp	2469	Reversible_NDPK2	1
281	adp	135	atp	2470	Reversible_NDPK3	1
281	adp	980	cdp	2470	Reversible_NDPK3	0
392	ctp	135	atp	2470	Reversible_NDPK3	0
392	ctp	980	cdp	2470	Reversible_NDPK3	1
281	adp	135	atp	2471	Reversible_NDPK4	1
281	adp	1239	dtdp	2471	Reversible_NDPK4	0
555	dttp	135	atp	2471	Reversible_NDPK4	0
555	dttp	1239	dtdp	2471	Reversible_NDPK4	1
281	adp	135	atp	2472	Reversible_NDPK5	1
281	adp	1349	dgdp	2472	Reversible_NDPK5	0
892	dgtp	135	atp	2472	Reversible_NDPK5	0
892	dgtp	1349	dgdp	2472	Reversible_NDPK5	1

281	adp	135	atp	2473	Reversible_NDPK6	1
281	adp	1351	dudp	2473	Reversible_NDPK6	0
466	dutp	135	atp	2473	Reversible_NDPK6	0
466	dutp	1351	dudp	2473	Reversible_NDPK6	1
281	adp	135	atp	2474	Reversible_NDPK7	1
281	adp	1350	dcdp	2474	Reversible_NDPK7	0
396	dctp	135	atp	2474	Reversible_NDPK7	0
396	dctp	1350	dcdp	2474	Reversible_NDPK7	1
281	adp	135	atp	2475	Reversible_NDPK8	1
281	adp	1348	dadp	2475	Reversible_NDPK8	0
393	datp	135	atp	2475	Reversible_NDPK8	0
393	datp	1348	dadp	2475	Reversible_NDPK8	1
859	nh4[p]	858	nh4[e]	2476	Reversible_NH4tex	0
1160	nh4	859	nh4[p]	2477	Reversible_NH4tpp	0
861	ni2[p]	860	ni2[e]	2478	Reversible_NI2tex	0
862	nmn[p]	863	nmn[e]	2479	Reversible_NMNtex	1
1161	dnad	98	h	2480	Reversible_NNATr	0
1161	dnad	135	atp	2480	Reversible_NNATr	1
1161	dnad	1442	nicrnt	2480	Reversible_NNATr	1
1192	ppi	98	h	2480	Reversible_NNATr	0
1192	ppi	135	atp	2480	Reversible_NNATr	0
1192	ppi	1442	nicrnt	2480	Reversible_NNATr	0
98	h	437	h[p]	2481	Reversible_NO2t2rpp	0
98	h	1162	no2[p]	2481	Reversible_NO2t2rpp	0
868	no2	437	h[p]	2481	Reversible_NO2t2rpp	0
868	no2	1162	no2[p]	2481	Reversible_NO2t2rpp	0
1162	no2[p]	865	no2[e]	2482	Reversible_NO2tex	0
866	no3[p]	869	no3[e]	2483	Reversible_NO3tex	0
873	no[p]	872	no[e]	2484	Reversible_NOtex	0
1163	no	873	no[p]	2485	Reversible_NOtpp	0
1164	o2s[p]	901	o2s[e]	2486	Reversible_O2Stex	0
903	o2[p]	902	o2[e]	2487	Reversible_O2tex	0
928	o2	903	o2[p]	2488	Reversible_O2tpp	0
98	h	904	cbp	2489	Reversible_OCBT	0
98	h	963	orn	2489	Reversible_OCBT	0
962	pi	904	cbp	2489	Reversible_OCBT	0
962	pi	963	orn	2489	Reversible_OCBT	0
1165	citr-L	904	cbp	2489	Reversible_OCBT	1
1165	citr-L	963	orn	2489	Reversible_OCBT	1
1166	octa[p]	907	octa[e]	2490	Reversible_OCTAtex	1
213	akg	624	glu-L	2491	Reversible_OHPBAT	1
213	akg	1450	ohpb	2491	Reversible_OHPBAT	0
1364	phthr	624	glu-L	2491	Reversible_OHPBAT	0
1364	phthr	1450	ohpb	2491	Reversible_OHPBAT	1
1167	orn[p]	916	orn[e]	2492	Reversible_ORNtex	1
1168	orot[p]	917	orot[e]	2493	Reversible_OROTtex	1
1052	prpp	918	orot5p	2494	Reversible_ORPT	1
1052	prpp	1192	ppi	2494	Reversible_ORPT	0
1169	orot	918	orot5p	2494	Reversible_ORPT	1
1169	orot	1192	ppi	2494	Reversible_ORPT	0
98	h	437	h[p]	2495	Reversible_PACALDt2rpp	0
98	h	1170	pacald[p]	2495	Reversible_PACALDt2rpp	0
1561	pacald	437	h[p]	2495	Reversible_PACALDt2rpp	0
1561	pacald	1170	pacald[p]	2495	Reversible_PACALDt2rpp	1
1170	pacald[p]	921	pacald[e]	2496	Reversible_PACALDtex	1
1171	peamn[p]	929	peamn[e]	2497	Reversible_PEAMNtex	1

98	h	856	nad	2498	Reversible_PERD	0
98	h	930	4per	2498	Reversible_PERD	0
870	nadh	856	nad	2498	Reversible_PERD	1
870	nadh	930	4per	2498	Reversible_PERD	0
1450	ohpb	856	nad	2498	Reversible_PERD	0
1450	ohpb	930	4per	2498	Reversible_PERD	1
567	gam6p	931	gam1p	2499	Reversible_PGAMT	1
499	f6p	568	g6p	2500	Reversible_PGI	1
281	adp	135	atp	2501	Reversible_PGK	1
281	adp	932	3pg	2501	Reversible_PGK	0
1113	13dpg	135	atp	2501	Reversible_PGK	0
1113	13dpg	932	3pg	2501	Reversible_PGK	1
932	3pg	489	2pg	2502	Reversible_PGM	1
568	g6p	589	g1p	2503	Reversible_PGMT	1
98	h	437	h[p]	2504	Reversible_PHEt2rpp	0
98	h	1172	phe-L[p]	2504	Reversible_PHEt2rpp	0
1485	phe-L	437	h[p]	2504	Reversible_PHEt2rpp	0
1485	phe-L	1172	phe-L[p]	2504	Reversible_PHEt2rpp	1
624	glu-L	213	akg	2505	Reversible_PHETA1	1
624	glu-L	1485	phe-L	2505	Reversible_PHETA1	0
1562	phpyr	213	akg	2505	Reversible_PHETA1	0
1562	phpyr	1485	phe-L	2505	Reversible_PHETA1	1
1172	phe-L[p]	936	phe-L[e]	2506	Reversible_PHEtex	1
98	h	437	h[p]	2507	Reversible_PIt2rpp	0
98	h	1173	pi[p]	2507	Reversible_PIt2rpp	0
962	pi	437	h[p]	2507	Reversible_PIt2rpp	0
962	pi	1173	pi[p]	2507	Reversible_PIt2rpp	0
1173	pi[p]	937	pi[e]	2508	Reversible_PItex	0
799	man6p	938	man1p	2509	Reversible_PMANM	1
1174	pnto-R[p]	942	pnto-R[e]	2510	Reversible_PNTOtex	1
135	atp	281	adp	2511	Reversible_PPAKr	1
135	atp	1492	ppap	2511	Reversible_PPAKr	0
1345	ppa	281	adp	2511	Reversible_PPAKr	0
1345	ppa	1492	ppap	2511	Reversible_PPAKr	1
1175	ppal[p]	943	ppal[e]	2512	Reversible_PPALtex	1
1175	ppal[p]	944	ppal	2513	Reversible_PPALtpp	1
1176	ppa[p]	945	ppa[e]	2514	Reversible_PPAtex	0
281	adp	135	atp	2515	Reversible_PPK2r	1
281	adp	1192	ppi	2515	Reversible_PPK2r	0
1491	pppi	135	atp	2515	Reversible_PPK2r	0
1491	pppi	1192	ppi	2515	Reversible_PPK2r	0
281	adp	135	atp	2516	Reversible_PPKr	1
281	adp	962	pi	2516	Reversible_PPKr	0
1192	ppi	135	atp	2516	Reversible_PPKr	0
1192	ppi	962	pi	2516	Reversible_PPKr	0
983	r5p	949	r1p	2517	Reversible_PPM	1
454	2dr5p	950	2dr1p	2518	Reversible_PPM2	1
98	h	437	h[p]	2519	Reversible_PPPNt2rpp	0
98	h	1177	pppn[p]	2519	Reversible_PPPNt2rpp	0
1563	pppn	437	h[p]	2519	Reversible_PPPNt2rpp	0
1563	pppn	1177	pppn[p]	2519	Reversible_PPPNt2rpp	1
1177	pppn[p]	952	pppn[e]	2520	Reversible_PPPNtex	1
1178	ppt[p]	953	ppt[e]	2521	Reversible_PPTtex	1
98	h	135	atp	2522	Reversible_PRAGSr	0
98	h	642	gly	2522	Reversible_PRAGSr	0
98	h	1642	pram	2522	Reversible_PRAGSr	0

281	adp	135	atp	2522	Reversible_PRAGSr	1
281	adp	642	gly	2522	Reversible_PRAGSr	0
281	adp	1642	pram	2522	Reversible_PRAGSr	0
962	pi	135	atp	2522	Reversible_PRAGSr	0
962	pi	642	gly	2522	Reversible_PRAGSr	0
962	pi	1642	pram	2522	Reversible_PRAGSr	0
1402	gar	135	atp	2522	Reversible_PRAGSr	0
1402	gar	642	gly	2522	Reversible_PRAGSr	1
1402	gar	1642	pram	2522	Reversible_PRAGSr	1
1179	prlp	955	prfp	2523	Reversible_PRMICI	1
1180	progly[p]	956	progly[e]	2524	Reversible_PROGLYtex	1
98	h	437	h[p]	2525	Reversible_PROt2rpp	0
98	h	1181	pro-L[p]	2525	Reversible_PROt2rpp	0
1498	pro-L	437	h[p]	2525	Reversible_PROt2rpp	0
1498	pro-L	1181	pro-L[p]	2525	Reversible_PROt2rpp	1
1181	pro-L[p]	957	pro-L[e]	2526	Reversible_PROtex	1
98	h	135	atp	2527	Reversible_PRPPS	0
98	h	983	r5p	2527	Reversible_PRPPS	0
177	amp	135	atp	2527	Reversible_PRPPS	1
177	amp	983	r5p	2527	Reversible_PRPPS	0
1052	prpp	135	atp	2527	Reversible_PRPPS	0
1052	prpp	983	r5p	2527	Reversible_PRPPS	1
1182	psclys[p]	958	psclys[e]	2528	Reversible_PSCLYStex	1
309	3psme	959	pep	2529	Reversible_PSCVT	1
309	3psme	1499	skm5p	2529	Reversible_PSCVT	1
962	pi	959	pep	2529	Reversible_PSCVT	0
962	pi	1499	skm5p	2529	Reversible_PSCVT	0
1183	pser-L[p]	961	pser-L[e]	2530	Reversible_PSERtex	1
927	coa	128	accoa	2531	Reversible_PTAr	1
927	coa	962	pi	2531	Reversible_PTAr	0
1075	actp	128	accoa	2531	Reversible_PTAr	1
1075	actp	962	pi	2531	Reversible_PTAr	0
1167	orn[p]	963	orn	2532	Reversible_PTRCORNt7pp	1
1167	orn[p]	1184	ptrc[p]	2532	Reversible_PTRCORNt7pp	0
1245	ptrc	963	orn	2532	Reversible_PTRCORNt7pp	0
1245	ptrc	1184	ptrc[p]	2532	Reversible_PTRCORNt7pp	1
1184	ptrc[p]	964	ptrc[e]	2533	Reversible_PTRCtex	1
174	ade	171	adn	2534	Reversible_PUNP1	1
174	ade	962	pi	2534	Reversible_PUNP1	0
949	r1p	171	adn	2534	Reversible_PUNP1	1
949	r1p	962	pi	2534	Reversible_PUNP1	0
174	ade	374	dad-2	2535	Reversible_PUNP2	1
174	ade	962	pi	2535	Reversible_PUNP2	0
950	2dr1p	374	dad-2	2535	Reversible_PUNP2	1
950	2dr1p	962	pi	2535	Reversible_PUNP2	0
676	gua	962	pi	2536	Reversible_PUNP3	0
676	gua	965	gsn	2536	Reversible_PUNP3	1
949	r1p	962	pi	2536	Reversible_PUNP3	0
949	r1p	965	gsn	2536	Reversible_PUNP3	1
676	gua	962	pi	2537	Reversible_PUNP4	0
676	gua	966	dgsn	2537	Reversible_PUNP4	1
950	2dr1p	962	pi	2537	Reversible_PUNP4	0
950	2dr1p	966	dgsn	2537	Reversible_PUNP4	1
713	hxan	962	pi	2538	Reversible_PUNP5	0
713	hxan	967	ins	2538	Reversible_PUNP5	1
949	r1p	962	pi	2538	Reversible_PUNP5	0

949	r1p	967	ins	2538	Reversible_PUNP5	1
713	hxan	962	pi	2539	Reversible_PUNP6	0
713	hxan	968	din	2539	Reversible_PUNP6	1
950	2dr1p	962	pi	2539	Reversible_PUNP6	0
950	2dr1p	968	din	2539	Reversible_PUNP6	1
949	r1p	962	pi	2540	Reversible_PUNP7	0
949	r1p	1508	xtsn	2540	Reversible_PUNP7	1
1231	xan	962	pi	2540	Reversible_PUNP7	0
1231	xan	1508	xtsn	2540	Reversible_PUNP7	1
949	r1p	962	pi	2541	Reversible_PYNP2r	0
949	r1p	1512	uri	2541	Reversible_PYNP2r	1
1552	ura	962	pi	2541	Reversible_PYNP2r	0
1552	ura	1512	uri	2541	Reversible_PYNP2r	1
98	h	437	h[p]	2542	Reversible_PYRt2rpp	0
98	h	1185	pyr[p]	2542	Reversible_PYRt2rpp	0
1148	pyr	437	h[p]	2542	Reversible_PYRt2rpp	0
1148	pyr	1185	pyr[p]	2542	Reversible_PYRt2rpp	1
1185	pyr[p]	969	pyr[e]	2543	Reversible_PYRtex	1
1186	r5p[p]	970	r5p[e]	2544	Reversible_R5Ptex	1
1187	xu5p-D	973	ru5p-L	2545	Reversible_RBP4E	1
1188	rib-D[p]	976	rib-D[e]	2546	Reversible_RIBtex	1
1189	rml	977	rmn	2547	Reversible_RMI	1
1190	rmn[p]	978	rmn[e]	2548	Reversible_RMNtex	1
826	dhap	979	rml1p	2549	Reversible_RMPA	0
1422	lald-L	979	rml1p	2549	Reversible_RMPA	1
1187	xu5p-D	117	ru5p-D	2550	Reversible_RPE	1
117	ru5p-D	983	r5p	2551	Reversible_RPI	1
98	h	856	nad	2552	Reversible_SBTPD	0
98	h	1517	sbt6p	2552	Reversible_SBTPD	0
499	f6p	856	nad	2552	Reversible_SBTPD	0
499	f6p	1517	sbt6p	2552	Reversible_SBTPD	1
870	nadh	856	nad	2552	Reversible_SBTPD	1
870	nadh	1517	sbt6p	2552	Reversible_SBTPD	0
1191	sbt-D[p]	986	sbt-D[e]	2553	Reversible_SBTtex	1
624	glu-L	213	akg	2554	Reversible_SDPTA	1
624	glu-L	1518	sl26da	2554	Reversible_SDPTA	0
1564	sl2a6o	213	akg	2554	Reversible_SDPTA	0
1564	sl2a6o	1518	sl26da	2554	Reversible_SDPTA	1
1192	ppi	98	h	2555	Reversible_SERASr	0
1192	ppi	135	atp	2555	Reversible_SERASr	0
1192	ppi	604	ser-L	2555	Reversible_SERASr	0
1394	seramp	98	h	2555	Reversible_SERASr	0
1394	seramp	135	atp	2555	Reversible_SERASr	1
1394	seramp	604	ser-L	2555	Reversible_SERASr	1
168	acser	128	accoa	2556	Reversible_SERAT	1
168	acser	604	ser-L	2556	Reversible_SERAT	1
927	coa	128	accoa	2556	Reversible_SERAT	1
927	coa	604	ser-L	2556	Reversible_SERAT	0
98	h	437	h[p]	2557	Reversible_SERt2rpp	0
98	h	1193	ser-L[p]	2557	Reversible_SERt2rpp	0
604	ser-L	437	h[p]	2557	Reversible_SERt2rpp	0
604	ser-L	1193	ser-L[p]	2557	Reversible_SERt2rpp	1
1193	ser-L[p]	989	ser-L[e]	2558	Reversible_SERtex	1
459	nadp	98	h	2559	Reversible_SHK3Dr	0
459	nadp	871	nadph	2559	Reversible_SHK3Dr	1
459	nadp	993	3dhsk	2559	Reversible_SHK3Dr	0

1523	skm	98	h	2559	Reversible_SHK3Dr	0
1523	skm	871	nadph	2559	Reversible_SHK3Dr	0
1523	skm	993	3dhsk	2559	Reversible_SHK3Dr	1
1194	skm[p]	994	skm[e]	2560	Reversible_SKMtex	1
996	so2[p]	995	so2[e]	2561	Reversible_SO2tex	0
1195	so2	996	so2[p]	2562	Reversible_SO2tpp	0
1196	so3[p]	997	so3[e]	2563	Reversible_SO3tex	0
1197	so4[p]	998	so4[e]	2564	Reversible_SO4tex	0
1198	spmd[p]	999	spmd[e]	2565	Reversible_SPMDtex	1
1199	succ[p]	1002	succ[e]	2566	Reversible_SUCCtex	1
540	fum	551	fum[p]	2567	Reversible_SUCFUMtpp	1
540	fum	1008	succ	2567	Reversible_SUCFUMtpp	0
1199	succ[p]	551	fum[p]	2567	Reversible_SUCFUMtpp	0
1199	succ[p]	1008	succ	2567	Reversible_SUCFUMtpp	1
281	adp	135	atp	2568	Reversible_SUCOAS	1
281	adp	927	coa	2568	Reversible_SUCOAS	0
281	adp	1008	succ	2568	Reversible_SUCOAS	0
838	succoa	135	atp	2568	Reversible_SUCOAS	0
838	succoa	927	coa	2568	Reversible_SUCOAS	1
838	succoa	1008	succ	2568	Reversible_SUCOAS	1
962	pi	135	atp	2568	Reversible_SUCOAS	0
962	pi	927	coa	2568	Reversible_SUCOAS	0
962	pi	1008	succ	2568	Reversible_SUCOAS	0
1200	sucr[p]	1004	sucr[e]	2569	Reversible_SUCRtex	1
1201	sulfac[p]	1005	sulfac[e]	2570	Reversible_SULFACtex	1
100	cdec3eACP	1006	tdec2eACP	2571	Reversible_T2DECAI	1
98	h	238	altrn	2572	Reversible_TAGURr	0
98	h	856	nad	2572	Reversible_TAGURr	0
870	nadh	238	altrn	2572	Reversible_TAGURr	0
870	nadh	856	nad	2572	Reversible_TAGURr	1
1121	tagur	238	altrn	2572	Reversible_TAGURr	1
1121	tagur	856	nad	2572	Reversible_TAGURr	0
405	e4p	468	g3p	2573	Reversible_TALA	1
405	e4p	985	s7p	2573	Reversible_TALA	1
499	f6p	468	g3p	2573	Reversible_TALA	0
499	f6p	985	s7p	2573	Reversible_TALA	1
1007	tartr-L	1008	succ	2574	Reversible_TARTRt7pp	0
1007	tartr-L	1202	tartr-L[p]	2574	Reversible_TARTRt7pp	1
1199	succ[p]	1008	succ	2574	Reversible_TARTRt7pp	1
1199	succ[p]	1202	tartr-L[p]	2574	Reversible_TARTRt7pp	0
1202	tartr-L[p]	1009	tartr-L[e]	2575	Reversible_TARTRtex	1
1203	taur[p]	1010	taur[e]	2576	Reversible_TAURtex	1
1204	tcynt[p]	1011	tcynt[e]	2577	Reversible_TCYNTtex	1
468	g3p	1017	tagdp-D	2578	Reversible_TGBPA	1
826	dhap	1017	tagdp-D	2578	Reversible_TGBPA	1
98	h	437	h[p]	2579	Reversible_THMDt2rpp	0
98	h	1205	thymd[p]	2579	Reversible_THMDt2rpp	0
1533	thymd	437	h[p]	2579	Reversible_THMDt2rpp	0
1533	thymd	1205	thymd[p]	2579	Reversible_THMDt2rpp	1
1205	thymd[p]	1018	thymd[e]	2580	Reversible_THMDtex	1
1206	thm[p]	1019	thm[e]	2581	Reversible_THMtex	1
1207	thrp[p]	1021	thrp[e]	2582	Reversible_THRPtex	1
98	h	437	h[p]	2583	Reversible_THRt2rpp	0
98	h	1208	thr-L[p]	2583	Reversible_THRt2rpp	0
1020	thr-L	437	h[p]	2583	Reversible_THRt2rpp	0
1020	thr-L	1208	thr-L[p]	2583	Reversible_THRt2rpp	1

1208	thr-L[p]	1022	thr-L[e]	2584	Reversible_THRtex	1
1209	thym[p]	1023	thym[e]	2585	Reversible_THYMtex	1
468	g3p	983	r5p	2586	Reversible_TKT1	1
468	g3p	1187	xu5p-D	2586	Reversible_TKT1	1
985	s7p	983	r5p	2586	Reversible_TKT1	1
985	s7p	1187	xu5p-D	2586	Reversible_TKT1	1
468	g3p	405	e4p	2587	Reversible_TKT2	1
468	g3p	1187	xu5p-D	2587	Reversible_TKT2	1
499	f6p	405	e4p	2587	Reversible_TKT2	1
499	f6p	1187	xu5p-D	2587	Reversible_TKT2	1
1210	tmao[p]	1024	tmao[e]	2588	Reversible_TMAOtex	1
1211	tma[p]	1025	tma[e]	2589	Reversible_TMAtex	1
950	2dr1p	962	pi	2590	Reversible_TMDPP	0
950	2dr1p	1533	thymd	2590	Reversible_TMDPP	1
1532	thym	962	pi	2590	Reversible_TMDPP	0
1532	thym	1533	thymd	2590	Reversible_TMDPP	1
468	g3p	826	dhap	2591	Reversible_TPI	1
1212	tre[p]	1026	tre[e]	2592	Reversible_TREtex	1
1028	indole	109	h2o	2593	Reversible_TRPAS2	0
1028	indole	1538	trp-L	2593	Reversible_TRPAS2	1
1148	pyr	109	h2o	2593	Reversible_TRPAS2	0
1148	pyr	1538	trp-L	2593	Reversible_TRPAS2	1
1160	nh4	109	h2o	2593	Reversible_TRPAS2	0
1160	nh4	1538	trp-L	2593	Reversible_TRPAS2	0
98	h	437	h[p]	2594	Reversible_TRPt2rpp	0
98	h	1213	trp-L[p]	2594	Reversible_TRPt2rpp	0
1538	trp-L	437	h[p]	2594	Reversible_TRPt2rpp	0
1538	trp-L	1213	trp-L[p]	2594	Reversible_TRPt2rpp	1
1213	trp-L[p]	1029	trp-L[e]	2595	Reversible_TRPtex	1
856	nad	98	h	2596	Reversible_TRSARr	0
856	nad	870	nadh	2596	Reversible_TRSARr	1
856	nad	1030	2h3oppan	2596	Reversible_TRSARr	0
1118	glyc-R	98	h	2596	Reversible_TRSARr	0
1118	glyc-R	870	nadh	2596	Reversible_TRSARr	0
1118	glyc-R	1030	2h3oppan	2596	Reversible_TRSARr	1
1214	tsul[p]	1031	tsul[e]	2597	Reversible_TSULtex	1
1215	tungs[p]	1034	tungs[e]	2598	Reversible_TUNGStex	1
1216	tym[p]	1035	tym[e]	2599	Reversible_TYMtex	1
1217	tyrp[p]	1036	tyrp[e]	2600	Reversible_TYRPtex	1
98	h	437	h[p]	2601	Reversible_TYRt2rpp	0
98	h	1219	tyr-L[p]	2601	Reversible_TYRt2rpp	0
1540	tyr-L	437	h[p]	2601	Reversible_TYRt2rpp	0
1540	tyr-L	1219	tyr-L[p]	2601	Reversible_TYRt2rpp	1
624	glu-L	213	akg	2602	Reversible_TYRTA	1
624	glu-L	1540	tyr-L	2602	Reversible_TYRTA	0
1218	34hpp	213	akg	2602	Reversible_TYRTA	0
1218	34hpp	1540	tyr-L	2602	Reversible_TYRTA	1
1219	tyr-L[p]	1037	tyr-L[e]	2603	Reversible_TYRtex	1
1220	uacgam[p]	1038	uacgam[e]	2604	Reversible_UACGAMtex	1
1221	uacmam	142	uacgam	2605	Reversible_UAG2E	1
118	ACP	70	3hmrsACP	2606	Reversible_UAGAAT	1
118	ACP	142	uacgam	2606	Reversible_UAGAAT	0
1547	u3aga	70	3hmrsACP	2606	Reversible_UAGAAT	1
1547	u3aga	142	uacgam	2606	Reversible_UAGAAT	1
1222	udpacgal[p]	1041	udpacgal[e]	2607	Reversible_UDPACGALtex	1
1043	udpgal	1042	udpg	2608	Reversible_UDPG4E	1

1223	udpgal[p]	1044	udpgal[e]	2609	Reversible_UDPGALtex	1
1224	udpglcur[p]	1045	udpglcur[e]	2610	Reversible_UDPGLCURtex	1
1225	udpg[p]	1046	udpg[e]	2611	Reversible_UDPGtex	1
213	akg	624	glu-L	2612	Reversible_UDPKAAT	1
213	akg	1546	udpLa4o	2612	Reversible_UDPKAAT	0
1548	udpLa4n	624	glu-L	2612	Reversible_UDPKAAT	0
1548	udpLa4n	1546	udpLa4o	2612	Reversible_UDPKAAT	1
589	g1p	1042	udpg	2613	Reversible_UGLT	0
589	g1p	1047	gal1p	2613	Reversible_UGLT	1
1043	udpgal	1042	udpg	2613	Reversible_UGLT	1
1043	udpgal	1047	gal1p	2613	Reversible_UGLT	0
281	adp	135	atp	2614	Reversible_UMPK	1
281	adp	1242	ump	2614	Reversible_UMPK	0
1440	udp	135	atp	2614	Reversible_UMPK	0
1440	udp	1242	ump	2614	Reversible_UMPK	1
1226	ump[p]	1049	ump[e]	2615	Reversible_UMPtex	1
98	h	437	h[p]	2616	Reversible_URAt2rpp	0
98	h	1227	ura[p]	2616	Reversible_URAt2rpp	0
1552	ura	437	h[p]	2616	Reversible_URAt2rpp	0
1552	ura	1227	ura[p]	2616	Reversible_URAt2rpp	1
1227	ura[p]	1053	ura[e]	2617	Reversible_URAtex	1
1055	urea[p]	1054	urea[e]	2618	Reversible_UREAtex	1
1228	urea	1055	urea[p]	2619	Reversible_UREAtpp	1
281	adp	135	atp	2620	Reversible_URIDK2r	1
281	adp	875	dump	2620	Reversible_URIDK2r	0
1351	dudp	135	atp	2620	Reversible_URIDK2r	0
1351	dudp	875	dump	2620	Reversible_URIDK2r	1
98	h	437	h[p]	2621	Reversible_URIt2rpp	0
98	h	1229	uri[p]	2621	Reversible_URIt2rpp	0
1512	uri	437	h[p]	2621	Reversible_URIt2rpp	0
1512	uri	1229	uri[p]	2621	Reversible_URIt2rpp	1
1229	uri[p]	1056	uri[e]	2622	Reversible_URItex	1
98	h	437	h[p]	2623	Reversible_VALt2rpp	0
98	h	1230	val-L[p]	2623	Reversible_VALt2rpp	0
1554	val-L	437	h[p]	2623	Reversible_VALt2rpp	0
1554	val-L	1230	val-L[p]	2623	Reversible_VALt2rpp	1
624	glu-L	213	akg	2624	Reversible_VALTA	1
624	glu-L	1554	val-L	2624	Reversible_VALTA	0
729	3mob	213	akg	2624	Reversible_VALTA	0
729	3mob	1554	val-L	2624	Reversible_VALTA	1
1230	val-L[p]	1057	val-L[e]	2625	Reversible_VALtex	1
1060	xan[p]	1059	xan[e]	2626	Reversible_XANtex	1
1231	xan	1060	xan[p]	2627	Reversible_XANtpp	1
1232	xmp[p]	1061	xmp[e]	2628	Reversible_XMPtex	1
98	h	437	h[p]	2629	Reversible_XTSNt2rpp	0
98	h	1233	xtsn[p]	2629	Reversible_XTSNt2rpp	0
1508	xtsn	437	h[p]	2629	Reversible_XTSNt2rpp	0
1508	xtsn	1233	xtsn[p]	2629	Reversible_XTSNt2rpp	1
1233	xtsn[p]	1062	xtsn[e]	2630	Reversible_XTSNtex	1
1234	xylu-D	1063	xyl-D	2631	Reversible_XYLI1	1
1235	fru	1064	glc-D	2632	Reversible_XYLI2	1
1236	xyl-D[p]	1065	xyl-D[e]	2633	Reversible_XYLtex	1
1237	xylu-L[p]	1066	xylu-L[e]	2634	Reversible_XYLUtex	1
1068	zn2[p]	1067	zn2[e]	2635	Reversible_Zn2tex	0
