# Supplementary material for: Path finding methods accounting for stoichiometry in metabolic networks
Source: Genome Biol. 2011 May 27;12(5):R49. doi: 10.1186/gb-2011-12-5-r49 (PMC3219972; doi:10.1186/gb-2011-12-5-r49)
Supplement: Additional file 2 — Supporting data for side-by-side comparison. Word document containing a list of 40 reference pathways used in the side-by-side comparison, a side-by-side comparison using accuracy rate, and a discussion on infeasible pathways in Figure 6 [7,9,12,13,35-37,41,44,50-56]. [file gb-2011-12-5-r49-S2.DOC]

**Supplementary Material III: Accuracy Analysis**

Path finding methods accounting for stoichiometry in metabolic networks

Jon Pey^1^, Joaquin Prada^1^, J.E. Beasley^2,*^ and Francisco J. Planes^1,*^

As mentioned in the main paper, other authors [9, 12, 13] make use of the parameter “accuracy” (instead of recovery rate) to validate their path finding methods. For the 40 reference metabolic pathways discussed in Planes and Beasley [37], we repeated the same analysis as conducted in the main paper, but using the accuracy rate instead of recovery rate.

In essence, the evaluation procedure based on accuracy consists of comparing the intermediate metabolites involved in the computed paths and the reference path. If a metabolite is active both in the computed and the reference path, then it is a true positive. If a metabolite is active in the computed path but it is not in the reference path, then it is a false positive. If a metabolite is active in the reference path but it is not involved in the computed path, it is a false negative. We denote TP as the number of true positives for a given computed path; FP as the number of false positives; and FN the number of false negatives.

We then define sensitivity (Sn) as the fraction of metabolites in the computed path active in the reference path, i.e. Sn = TP/(TP + FP). Similarly, the fraction of metabolites in the reference path active in the computed path is denoted as Positive Predictive Value (PPV), namely PPV= TP/(TP + FN). In some papers [9, 12], accuracy (Acc) is defined as the arithmetic mean of Sn and PPV, i.e. Acc = (Sn+PPV)/2, whilst in others (e.g. Faust *et al*. [13]) the geometric mean is used, i.e. Acc = √(Sn*PPV). In our analysis we have used the geometric mean for calculation of accuracy.

To illustrate the above definitions, we computed the three shortest CFPs for the degradation of propionate (ppa) into succinate (succ), as observed in Table S1. The reference path in this case is: ppa→ppcoa→2micit→2mcacr→micit→succ, which is recovered in the third CFP, since Acc=1.

| **Path** | **TP** | **FP** | **FN** | **Sn** | **PPV** | **Acc** |
| --- | --- | --- | --- | --- | --- | --- |
| ppa→ppcoa→succoa→succ | 1 | 1 | 3 | 0.5 | 0.25 | 0.35 |
| ppa→ppcoa→coa→succoa→succ | 1 | 2 | 3 | 0.33 | 0.25 | 0.29 |
| ppa→ppcoa→2micit→2mcacr→micit→succ | 4 | 0 | 0 | 1 | 1 | 1 |

**Table S1: ·Accuracy for the 3 shortest CFPs from propionate to succinate**

| **A**   |  | **k=1** | **k≤5** | **k≤10** | **k≤100** | | --- | --- | --- | --- | --- | | **CFP approach** | 0.856 | 0.947 | 0.966 | 1 | | **Connectivity** | 0.848 | 0.938 | 0.943 | 0.986 | | **Hubs** | 0.662 | 0.797 | 0.844 | 0.918 | | **Atomic approach** | 0.693 | 0.764 | 0.806 | 0.822 | | **Topology** | 0.575 | 0.709 | 0.743 | 0.831 | | **B**  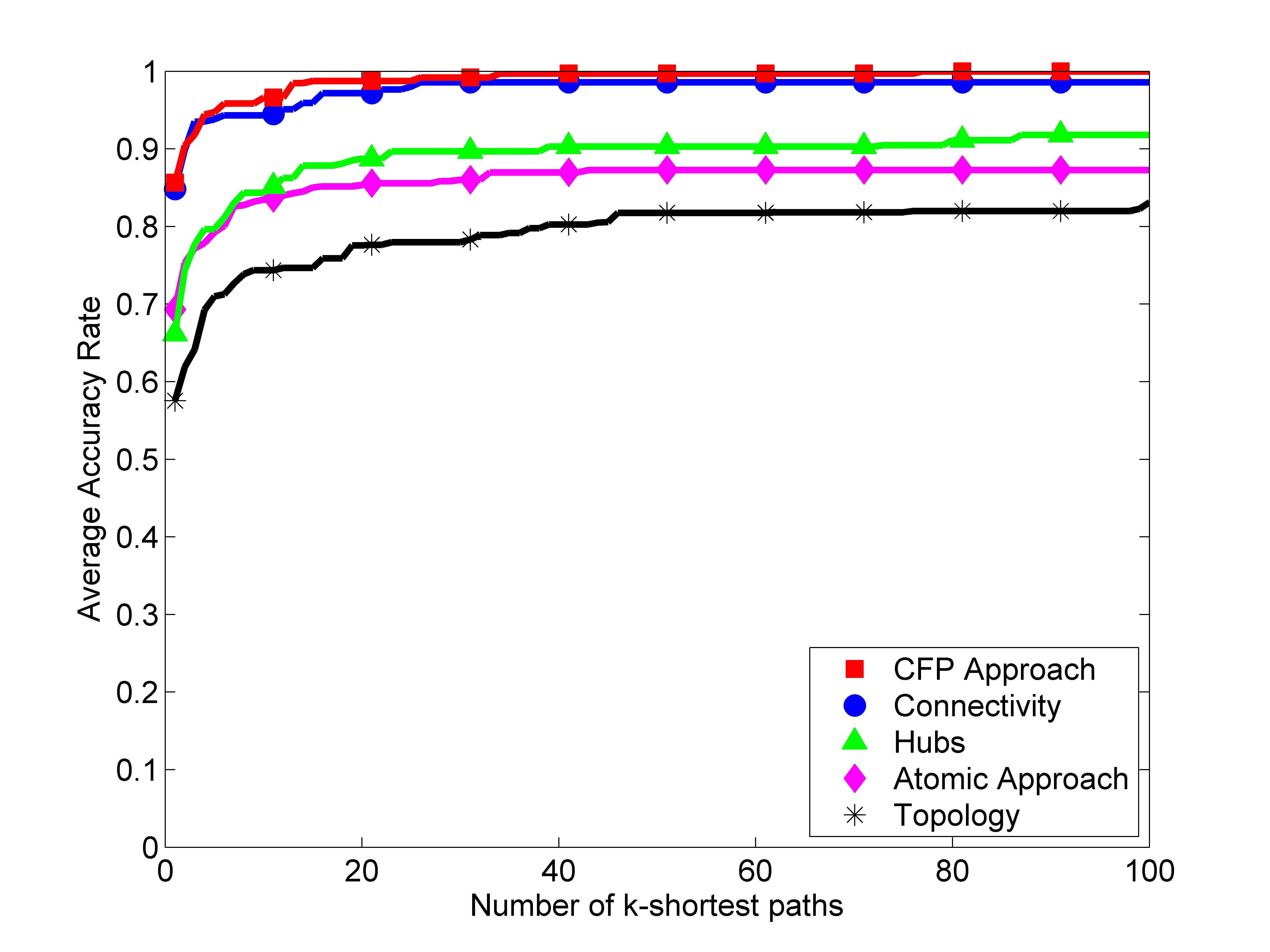 |
| --- | --- | --- | --- | --- | --- | --- | --- | --- | --- | --- | --- | --- | --- | --- | --- | --- | --- | --- | --- | --- | --- | --- | --- | --- | --- | --- | --- | --- | --- | --- | --- |

**Figure S1: (A)** Average accuracy rate among the k-shortest CFPs for k=1,5,10,100; **(B)** Average accuracy rate plot among the k-shortest CFPs for k=1,…,100

Going back to the analysis conducted in the main paper, Figure S1A shows the average accuracy rate among the k-shortest CFPs, for k=1, 5, 10, 100, in the 40 *E. coli* reference pathways, which are summarized in Table S2. Our CFP is compared with existing path finding approaches, which are grouped into different strategies. First strategy consists of removing any arc involving a highly connected metabolite (hubs) from the metabolic graph [7]. We took the list of hubs from Planes and Beasley [37]. Secondly, weights are assigned to metabolite according to their connectivity in the metabolic graph, *i.e.* number of reactions involving a metabolite [9, 35]. The third strategy involves the use of structural atomic information so as to ensure effective carbon exchange between the source and the target metabolite [41]. Finally, the classic definition of topology is added to the analysis. Figure S1B plots the average accuracy rate among the 40 reference pathways for k=1, …, 100 for the different strategies. Though average accuracy rate is generally higher than average recovery rate and the differences between the 4 scenarios are smoothed, the behaviour is very similar.

| **Pathway number** | **Pathway Name** | **Metabolites** |
| --- | --- | --- |
| 1) | Gluconeogenesis | pyr →pep→2pg→3pg→13dpg→g3p→f6p→g6p |
| 2) | Glycogen Biosynthesis | g6p→ g1p→ adpglc→ glycogen |
| 3) | Glycolysis | glc-D→g6p→ f6p→ g3p→13dpg→3pg→2pg→pep→pyr |
| 4) | Proline biosynthesis | akg→glu-L→ glu5p→glu5sa→1pyr5c→pro-L |
| 5) | Ketogluconate metabolism | 25dkglcn→5dglcn→ glcn→6pgc |
| 6) | Pentose Phosphate Pathway | g6p→6pgl→6pgc→ru5p-D→xu5p-D→ f6p |
| 7) | Salvage pathway deoxythymidine phosphate | dcyt→ duri→2dr1p→thymd→ dtmp |
| 8) | TCA Cycle | oaa→ cit→ acon-C→icit→akg→succoa→succ→fum→mal-L→oaa |
| 9) | NAD biosynthesis | asp-L→iasp→quln→nicrnt→dnad→nad |
| 10) | Arginine biosynthesis | glu-L→acglu→acg5p→acg5sa→acorn→orn→citr-L→argsuc→arg-L |
| 11) | Sperdimine biosynthesis  Biosynthesis | orn→ptrc→spmd |
| 12) | Threonine degradation | thr-L→2aobut→gly |
| 13) | Serine biosynthesis | 3pg→3php→pser-L→ser-L |
| 14) | Histidine biosynthesis | prpp→prbatp→prbamp→prfp→prlp→ eig3p→imacp→hisp→histd→his-L |
| 15) | Tirosine biosynthesis | chor→pphn→34hpp→tyr-L |
| 16) | Coenzyme A biosynthesis | pnto-R→4ppan→4ppcys→pan4p→ dpcoa→coa |
| 17) | Pantothenate biosynthesis | val-L→3mob→2dhp→pant-R→ pnto-R |
| 18) | Tetrahydrofolate biosynthesis | gtp→ ahdt→dhpmp→dhnpt→6hmhpt→6hmhptpp→dhpt→dhf→thf |
| 19) | Riboflavin/FMN/FAD biosynthesis | gtp→25drapp→5apru→5aprbu→4r5au→dmlz→ribflv→fmn→fad |
| 20) | Heme biosynthesis | uppg3→cpppg3→pppg9→ppp9→ pheme→hemeO |
| 21) | De novo synthesis pyrimidine ribonucletides | cbp→cbasp→dhor-S→orot→orot5p→ump→udp→utp→ctp |
| 22) | De novo synthesis pyrimidine deoxyribonucleotid eoxyribonucletides | utp→dutp→dump→dtmp→dtdp→dttp |
| 23) | Phenylethylamine degradation | peamn[p]→pacald[p]→pacald→pac |
| 24) | Rhamnose degradation | rmn→rml→rml1p→lald-L→lac-L→pyr |
| 25) | Fucose degradation | fuc-L→fcl-L→fc1p→lald-L→lac-L→ pyr |
| 26) | Entner-Doudoroff | g6p→6pgl→6pgc→2ddg6p→pyr |
| 27) | Anaerobic respiration | pyr→accoa→cit→acon-C→icit→akg |
| 28) | Arginine degradation | arg-L→sucarg→sucorn→sucgsa→sucglu→glu-L |
| 29) | Proline degradation | pro-L→1pyr5c→glu-L |
| 30) | Glycolate degradation | glyclt→glx→2h3oppan→glyc-R→3pg |
| 31) | Phospholipid biosynthesis | cdpdddecg→pgp120→pg120→pg120[p]→clpn120[p] |
| 32) | Biosynthesis of cysteine | ser-L→acser→cys-L |
| 33) | Allantoin degradation | alltn→alltt→urdglyc→glx→2h3oppan→glyc-R→3pg |
| 34) | Deoxycytidine degradation | dcyt→duri→2dr1p→2dr5p→g3p |
| 35) | Phenylalanine biosynthesis | chor→pphn→phpyr→phe-L |
| 36) | Glyoxylate cycle | glx→mal-L→oaa→cit→acon-C→icit→glx |
| 37) | Propionate degradation | ppa→ppcoa→2mcit→2mcacn→micit→succ |
| 38) | Glutamate biosynthesis cycle | glu-L→gln-L→glu-L |
| 39) | Biotin biosynthesis | pmcoa→8aonn→dann→dtbt→btn |
| 40) | Glycerol degradation | glyc→glyc3p→dhap→g3p |

**Table S2:** Set of 40 pathways extracted from Planes and Beasley [37].

The analysis accomplished in section "Side-by-side comparison with stoichiometry" of the main paper so as to evaluate the effect of stoichiometric constraints (Equations (5)-(6)) was repeated, but when accuracy rate was used instead of recovery rate, as can be observed in Figure S2. Results are very similar to Figure 6 in the main paper.

| **A**  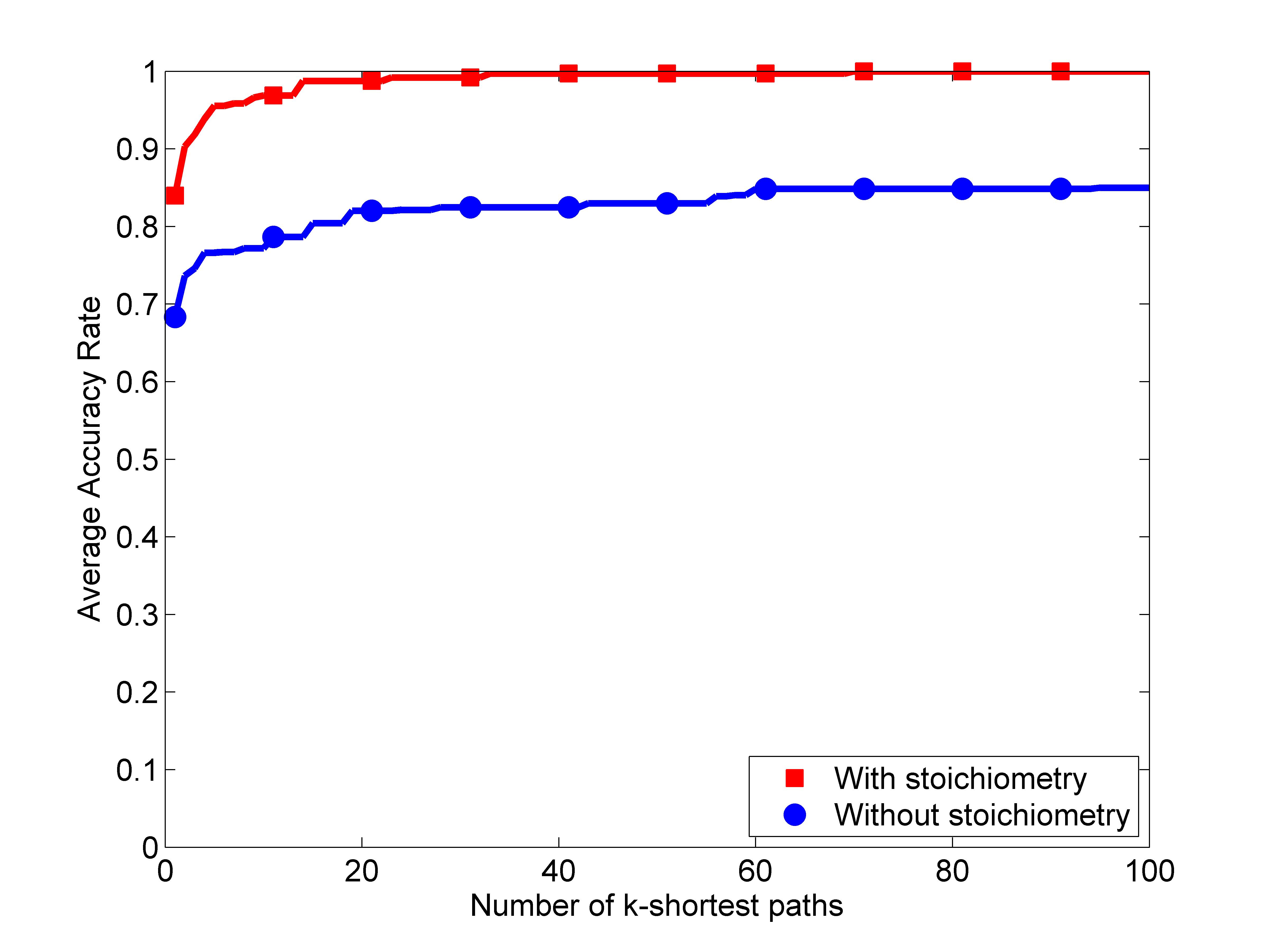 | **B**  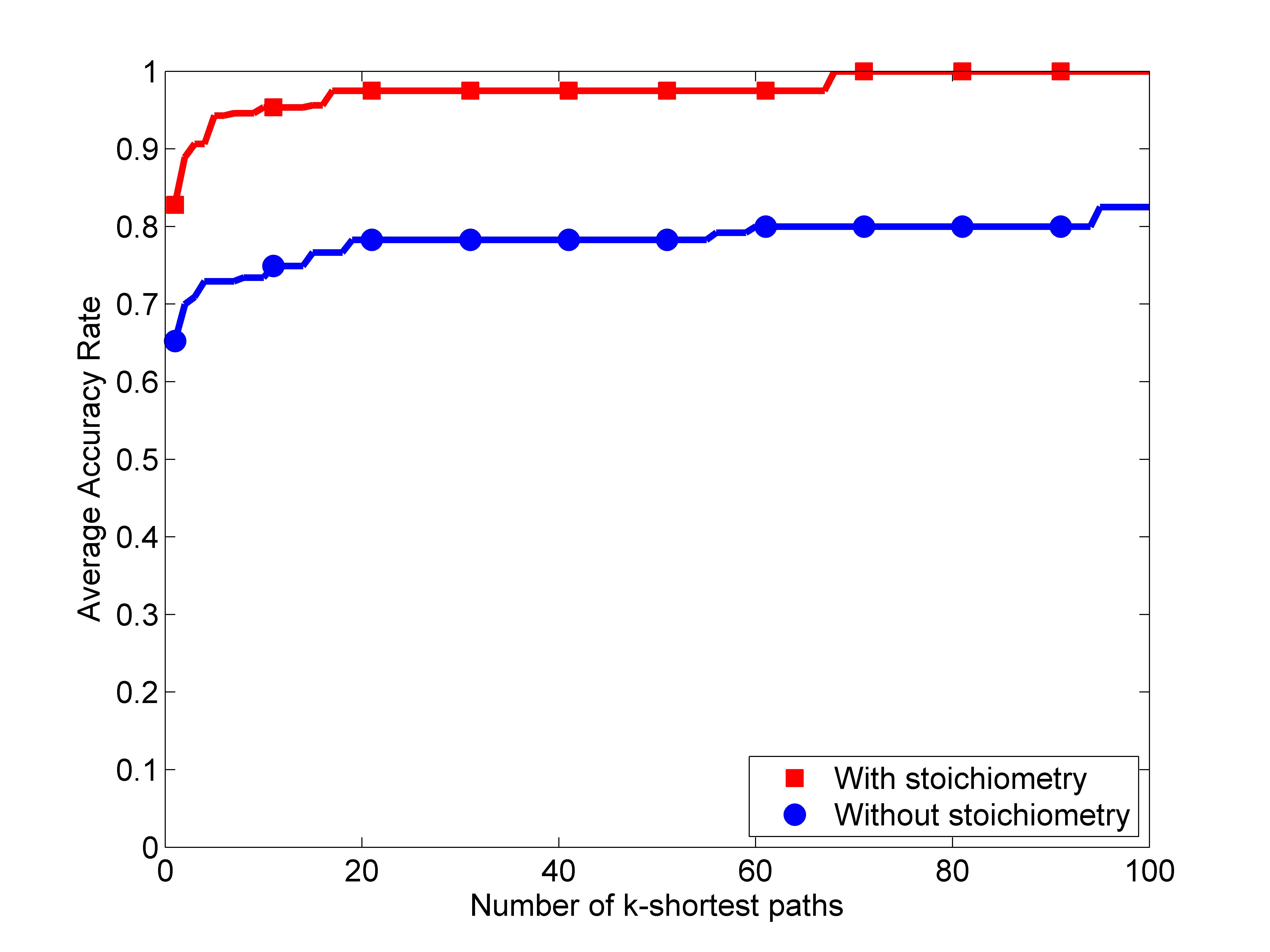 | **C** |
| --- | --- | --- |

**Figure S2:** Average accuracy rate among the k-shortest paths for k=1,…,100 for CFP approach with and without considering stoichometry in oxic **(A)** and anoxic **(B)** conditions; **(C)** Average accuracy rate among the k-shortest paths for k=1,5,10,100 for CFP approach in oxic and anoxic conditions.

As was mentioned in the main paper, the introduction of stoichiometry in our CFP approach allowed us to detect several pathways from our 40 pathways (see Table S2) unable to work in steady-state according to the imposed growth medium (in Figure S2 glucose as sole carbon source in oxic and anoxic conditions, respectively). These infeasible pathways are summarized in Table S3.

| **Infeasible pathways** | |
| --- | --- |
| **Oxic** | **Anoxic** |
| Ketogluconate Metabolism  Salvage pathway deoxythymidine phosphate  Phenylethylamine degradation  Rhamnose degradation  Fucose degradation  Biotin Biosynthesis | Ketogluconate Metabolism  Salvage pathway deoxythymidine phosphate  Phenylethylamine degradation  Rhamnose degradation  Fucose degradation  Biotin Biosynthesis  TCA cycle  Allantoin degradation |

**Table S3:** Infeasible (stoichiometrically unbalanced) metabolic pathways when we fixed a minimal medium based on glucose as sole carbon source in oxic/anoxic conditions.

As noted in the main paper, the metabolism of ketogluconate is not active, since it cannot be produced from glucose [44]. Similarly, the salvage pathway of deoxythymidine phosphate is only feasible if thymine is in the growth medium [55]. In addition, the route of degradation of 2-phenylethylamine does not work in *E. coli* with glucose as a sole carbon source. 2-phenylethylamine is indeed biosynthesized from phenylalanine by enzymatic decarboxylation. However, this enzyme has not yet been found in *E. coli*. With respect to the L-Fucose degradation pathway, most published work studies this pathway with L-Fucose freely available in the growth medium [50]. Though it seems that L-Fucose can be endogenously produced from complex molecules [51], its biosynthesis from glucose is unclear. The same applies to the pathway for Rhamnose degradation. Regarding the pathway for biotin biosynthesis, there is a lack of biochemical information as to the required metabolite S-Adenosyl-4-methylthio-2-oxobutanoate (amob). This metabolite is a dead-end and therefore its metabolism is unknown. This makes the pathway unable to operate at steady-state using the metabolic network of Feist *et al*. [36]. Note that this lack of information is also found in other metabolic databases: EcoCyc [53] and KEGG [52].

In anoxic conditions, two further pathways turned out to be infeasible, namely TCA Cycle and Allantoin degradation. As noted in the main paper, the TCA Cycle requires oxygen to balance certain cofactor production in the respiratory chain [54]. Oxygen is also essential for allantoin metabolism [56].
