# Supplementary material for: Path finding methods accounting for stoichiometry in metabolic networks
Source: Genome Biol. 2011 May 27;12(5):R49. doi: 10.1186/gb-2011-12-5-r49 (PMC3219972; doi:10.1186/gb-2011-12-5-r49)
Supplement: Additional file 3 — Supporting data for Figures 8 and 9. Details of the 100 shortest CFPs in oxic and anoxic conditions from Pyr to OAA. [file gb-2011-12-5-r49-S3.PDF]

Supplementary Material IV: Additional data for pyr-oaa analysis

Medium used in the pyr-oaa simulation

Oxic conditions

Abbrev.	OfficialName	KeggID
pi	Phosphate	C00009
fe2	Fe2+	C00023
h2o	H2O	C00001
h	H+	C00080
co2	CO2	C00011
k	Potassium	C00238
na1	Sodium	C01330
so4	Sulfate	C00059
glc-D	D-Glucose	C00031
nh4	Ammonium	C01342
o2	O2	C00007

Anoxic conditions

Abbrev.	OfficialName	KeggID
pi	Phosphate	C00009
fe2	Fe2+	C00023
h2o	H2O	C00001
h	H+	C00080
co2	CO2	C00011
k	Potassium	C00238
na1	Sodium	C01330
so4	Sulfate	C00059
glc-D	D-Glucose	C00031
nh4	Ammonium	C01342

100-shortest CFPs between pyr and oaa

Oxic conditions

1 pyr->pep->oaa
2 pyr->accoa->mal-L->oaa
3 pyr->accoa->cit->oaa
4 pyr->ac->accoa->mal-L->oaa
5 pyr->ac->accoa->cit->oaa
6 pyr->ac->actp->accoa->mal-L->oaa
7 pyr->ac->actp->accoa->cit->oaa
8 pyr->accoa->coa->succoa->succ->fum->mal-L->oaa
9 pyr->accoa->cit->acon-C->icit->glx->mal-L->oaa
10 pyr->ac->accoa->cit->acon-C->icit->glx->mal-L->oaa
11 pyr->accoa->cit->acon-C->icit->succ->fum->mal-L->oaa
12 pyr->ac->accoa->coa->succoa->succ->fum->mal-L->oaa
13 pyr->accoa->coa->ppcoa->succoa->succ->fum->mal-L->oaa
14 pyr->accoa->hxcoa->coa->succoa->succ->fum->mal-L->oaa
15 pyr->23dhdp->thdp->sl2a6o->sl26da->succ->fum->mal-L->oaa
16 pyr->accoa->malcoa->coa->succoa->succ->fum->mal-L->oaa
17 pyr->ac->accoa->cit->acon-C->icit->succ->fum->mal-L->oaa
18 pyr->ac->actp->accoa->cit->acon-C->icit->glx->mal-L->oaa
19 pyr->accoa->hxcoa->coa->ppcoa->succoa->succ->fum->mal-L->oaa
20 pyr->accoa->coa->succoa->sl2a6o->sl26da->succ->fum->mal-L->oaa
21 pyr->ac->accoa->coa->ppcoa->succoa->succ->fum->mal-L->oaa
22 pyr->ac->actp->accoa->coa->succoa->succ->fum->mal-L->oaa
23 pyr->ac->accoa->malcoa->coa->succoa->succ->fum->mal-L->oaa
24 pyr->for->fgam->10fthf->fprica->imp->dcamp->fum->mal-L->oaa
25 pyr->accoa->malcoa->coa->ppcoa->succoa->succ->fum->mal-L->oaa
26 pyr->ac->accoa->hxcoa->coa->succoa->succ->fum->mal-L->oaa
27 pyr->accoa->cit->acon-C->icit->glx->2h3oppan->glyc-R->2pg->pep->oaa
28 pyr->23dhdp->thdp->sl2a6o->sl26da->succ->succoa->coa->accoa->cit->oaa
29 pyr->accoa->cit->acon-C->icit->akg->succoa->succ->fum->mal-L->oaa
30 pyr->ac->accoa->coa->succoa->sl2a6o->sl26da->succ->fum->mal-L->oaa
31 pyr->ac->actp->accoa->cit->acon-C->icit->succ->fum->mal-L->oaa
32 pyr->ac->actp->accoa->malcoa->coa->succoa->succ->fum->mal-L->oaa
33 pyr->ac->actp->accoa->hxcoa->coa->succoa->succ->fum->mal-L->oaa
34 pyr->ac->accoa->hxcoa->coa->ppcoa->succoa->succ->fum->mal-L->oaa
35 pyr->accoa->hxcoa->coa->succoa->sl2a6o->sl26da->succ->fum->mal-L->oaa
36 pyr->ac->actp->accoa->coa->ppcoa->succoa->succ->fum->mal-L->oaa
37 pyr->23dhdp->thdp->sl2a6o->sl26da->succ->succoa->coa->accoa->mal-L->oaa
38 pyr->for->fgam->fpram->air->5caiz->5aizc->25aics->fum->mal-L->oaa
39 pyr->ac->accoa->malcoa->coa->ppcoa->succoa->succ->fum->mal-L->oaa
40 pyr->accoa->coa->ppcoa->succoa->sl2a6o->sl26da->succ->fum->mal-L->oaa
41 pyr->accoa->coa->ppcoa->2mcit->2mcacn->micit->succ->fum->mal-L->oaa
42 pyr->accoa->malcoa->coa->succoa->sl2a6o->sl26da->succ->fum->mal-L->oaa
43 pyr->pep->2pg->3pg->3php->pser-L->ser-L->acser->ac->accoa->cit->oaa
44 pyr->ac->accoa->cit->acon-C->icit->akg->succoa->succ->fum->mal-L->oaa
45 pyr->ac->actp->accoa->coa->succoa->sl2a6o->sl26da->succ->fum->mal-L->oaa
46 pyr->ac->actp->accoa->hxcoa->coa->ppcoa->succoa->succ->fum->mal-L->oaa
47 pyr->ac->accoa->cit->acon-C->icit->glx->2h3oppan->glyc-R->2pg->pep->oaa
48 pyr->accoa->cit->acon-C->icit->glx->2h3oppan->hpyr->glyc-R->2pg->pep->oaa
49 pyr->ac->accoa->hxcoa->coa->succoa->sl2a6o->sl26da->succ->fum->mal-L->oaa
50 pyr->for->fgam->10fthf->methf->mlthf->ser-L->acser->ac->accoa->cit->oaa
51 pyr->ac->actp->accoa->malcoa->coa->ppcoa->succoa->succ->fum->mal-L->oaa

52 pyr->ac->accoa->coa->ppcoa->2mcit->2mcacn->micit->succ->fum->mal-L->oaa
53 pyr->accoa->cit->acon-C->icit->glx->2h3oppan->glyc-R->3pg->2pg->pep->oaa
54 pyr->pep->2pg->3pg->3php->pser-L->ser-L->acser->ac->accoa->mal-L->oaa
55 pyr->ac->accoa->coa->ppcoa->succoa->sl2a6o->sl26da->succ->fum->mal-L->oaa
56 pyr->accoa->coa->succoa->sucarg->sucorn->sucgsa->sucglu->succ->fum->mal-L->oaa
57 pyr->accoa->acglu->acg5p->acg5sa->acorn->orn->citr-L->argsuc->fum->mal-L->oaa
58 pyr->for->fgam->10fthf->thf->mlthf->ser-L->acser->ac->accoa->mal-L->oaa
59 pyr->accoa->malcoa->coa->ppcoa->succoa->sl2a6o->sl26da->succ->fum->mal-L->oaa
60 pyr->accoa->hxcoa->coa->ppcoa->2mcit->2mcacn->micit->succ->fum->mal-L->oaa
61 pyr->accoa->hxcoa->coa->ppcoa->succoa->sl2a6o->sl26da->succ->fum->mal-L->oaa
62 pyr->accoa->2aobut->athr-L->gly->ser-L->g3p->13dpg->3pg->2pg->pep->oaa
63 pyr->for->fgam->10fthf->methf->mlthf->ser-L->acser->ac->accoa->mal-L->oaa
64 pyr->for->fgam->10fthf->thf->mlthf->ser-L->acser->ac->accoa->cit->oaa
65 pyr->ac->accoa->malcoa->coa->succoa->sl2a6o->sl26da->succ->fum->mal-L->oaa
66 pyr->accoa->malcoa->coa->ppcoa->2mcit->2mcacn->micit->succ->fum->mal-L->oaa
67 pyr->ac->actp->accoa->cit->acon-C->icit->akg->succoa->succ->fum->mal-L->oaa
68 pyr->ac->accoa->acglu->acg5p->acg5sa->acorn->orn->citr-L->argsuc->fum->mal-L->oaa
69 pyr->ac->accoa->2aobut->athr-L->gly->ser-L->g3p->13dpg->3pg->2pg->pep->oaa
70 pyr->for->fgam->10fthf->thf->mlthf->ser-L->g3p->13dpg->3pg->2pg->pep->oaa
71 pyr->ac->accoa->cit->acon-C->icit->glx->2h3oppan->hpyr->glyc-R->2pg->pep->oaa
72 pyr->accoa->2aobut->athr-L->gly->mlthf->ser-L->g3p->13dpg->3pg->2pg->pep->oaa
73 pyr->accoa->cit->acon-C->icit->glx->2h3oppan->hpyr->glyc-R->3pg->2pg->pep->oaa
74 pyr->pep->2pg->3pg->3php->pser-L->ser-L->acser->ac->actp->accoa->mal-L->oaa
75 pyr->ac->actp->accoa->cit->acon-C->icit->glx->2h3oppan->glyc-R->2pg->pep->oaa
76 pyr->ac->accoa->cit->acon-C->icit->glx->2h3oppan->glyc-R->3pg->2pg->pep->oaa
77 pyr->ac->accoa->coa->succoa->sucarg->sucorn->sucgsa->sucglu->succ->fum->mal-L->oaa
78 pyr->ac->actp->accoa->hxcoa->coa->succoa->sl2a6o->sl26da->succ->fum->mal-L->oaa
79 pyr->ac->actp->accoa->malcoa->coa->succoa->sl2a6o->sl26da->succ->fum->mal-L->oaa
80 pyr->pep->kdo8p->kdo->ckdo->cmp->r5p->prpp->imp->dcamp->fum->mal-L->oaa
81 pyr->ac->accoa->malcoa->coa->ppcoa->2mcit->2mcacn->micit->succ->fum->mal-L->oaa
82 pyr->ac->accoa->hxcoa->coa->ppcoa->succoa->sl2a6o->sl26da->succ->fum->mal-L->oaa
83 pyr->accoa->cit->acon-C->icit->akg->glu-L->4abut->sucsal->succ->fum->mal-L->oaa
84 pyr->accoa->cit->acon-C->icit->akg->succoa->sl2a6o->sl26da->succ->fum->mal-L->oaa
85 pyr->ac->accoa->malcoa->coa->ppcoa->succoa->sl2a6o->sl26da->succ->fum->mal-L->oaa
86 pyr->for->fgam->10fthf->methf->mlthf->ser-L->acser->ac->actp->accoa->cit->oaa
87 pyr->for->fgam->10fthf->methf->mlthf->ser-L->g3p->13dpg->3pg->2pg->pep->oaa
88 pyr->pep->2pg->3pg->3php->pser-L->ser-L->acser->ac->actp->accoa->cit->oaa
89 pyr->ac->actp->accoa->coa->ppcoa->succoa->sl2a6o->sl26da->succ->fum->mal-L->oaa
90 pyr->ac->accoa->hxcoa->coa->ppcoa->2mcit->2mcacn->micit->succ->fum->mal-L->oaa
91 pyr->for->fgam->10fthf->thf->mlthf->ser-L->acser->ac->actp->accoa->mal-L->oaa
92 pyr->accoa->hxcoa->coa->succoa->sucarg->sucorn->sucgsa->sucglu->succ->fum->mal-L->oaa
93 pyr->for->fgam->10fthf->methf->mlthf->ser-L->acser->ac->actp->accoa->mal-L->oaa
94 pyr->for->fgam->10fthf->thf->mlthf->ser-L->acser->ac->actp->accoa->cit->oaa
95 pyr->ac->actp->accoa->coa->ppcoa->2mcit->2mcacn->micit->succ->fum->mal-L->oaa
96 pyr->accoa->malcoa->coa->succoa->sucarg->sucorn->sucgsa->sucglu->succ->fum->mal-L->oaa
97 pyr->pep->2pg->3pg->13dpg->g3p->r5p->prpp->imp->dcamp->fum->mal-L->oaa
98 pyr->accoa->coa->ppcoa->succoa->sucarg->sucorn->sucgsa->sucglu->succ->fum->mal-L->oaa
99 pyr->accoa->acgam1p->uacgam->ump->uri->r1p->ins->imp->dcamp->fum->mal-L->oaa
100 pyr->23dhdp->thdp->sl2a6o->sl26da->succ->succoa->mmcoa-R->mmcoa-S->ppcoa->coa->accoa->cit->oaa

Anoxic conditions

1 pyr->pep->oaa
2 pyr->accoa->cit->oaa
3 pyr->accoa->mal-L->oaa
4 pyr->accoa->cit->acon-C->icit->glx->mal-L->oaa
5 pyr->for->fgam->10fthf->fprica->imp->dcamp->fum->mal-L->oaa
6 pyr->for->fgam->fpram->air->5caiz->5aizc->25aics->fum->mal-L->oaa
7 pyr->23dhdp->thdp->sl2a6o->sl26da->succ->succoa->coa->accoa->mal-L->oaa
8 pyr->accoa->cit->acon-C->icit->glx->2h3oppan->glyc-R->2pg->pep->oaa
9 pyr->23dhdp->thdp->sl2a6o->sl26da->succ->succoa->coa->accoa->cit->oaa
10 pyr->accoa->2aobut->athr-L->gly->ser-L->g3p->13dpg->3pg->2pg->pep->oaa
11 pyr->accoa->acglu->acg5p->acg5sa->acorn->orn->citr-L->argsuc->fum->mal-L->oaa
12 pyr->accoa->cit->acon-C->icit->glx->2h3oppan->glyc-R->3pg->2pg->pep->oaa
13 pyr->for->fgam->10fthf->thf->mlthf->ser-L->acser->ac->accoa->mal-L->oaa
14 pyr->for->fgam->10fthf->methf->mlthf->ser-L->acser->ac->accoa->cit->oaa
15 pyr->accoa->cit->acon-C->icit->glx->2h3oppan->hpyr->glyc-R->2pg->pep->oaa
16 pyr->for->fgam->10fthf->methf->mlthf->ser-L->acser->ac->accoa->mal-L->oaa
17 pyr->for->fgam->10fthf->thf->mlthf->ser-L->acser->ac->accoa->cit->oaa
18 pyr->pep->2pg->3pg->3php->pser-L->ser-L->acser->ac->accoa->mal-L->oaa
19 pyr->pep->2pg->3pg->3php->pser-L->ser-L->acser->ac->accoa->cit->oaa
20 pyr->accoa->2aobut->athr-L->gly->mlthf->ser-L->g3p->13dpg->3pg->2pg->pep->oaa
21 pyr->accoa->cit->acon-C->icit->glx->2h3oppan->hpyr->glyc-R->3pg->2pg->pep->oaa
22 pyr->accoa->acgam1p->uacgam->ump->uri->r1p->ins->imp->dcamp->fum->mal-L->oaa
23 pyr->for->fgam->10fthf->thf->mlthf->ser-L->g3p->13dpg->3pg->2pg->pep->oaa
24 pyr->for->fgam->10fthf->methf->mlthf->ser-L->acser->ac->actp->accoa->cit->oaa
25 pyr->for->fgam->10fthf->methf->mlthf->ser-L->acser->ac->actp->accoa->mal-L->oaa
26 pyr->for->fgam->10fthf->thf->mlthf->ser-L->acser->ac->actp->accoa->cit->oaa
27 pyr->pep->kdo8p->kdo->ckdo->cmp->r5p->prpp->imp->dcamp->fum->mal-L->oaa
28 pyr->for->fgam->10fthf->methf->mlthf->ser-L->g3p->13dpg->3pg->2pg->pep->oaa
29 pyr->pep->2pg->3pg->13dpg->g3p->r5p->prpp->imp->dcamp->fum->mal-L->oaa
30 pyr->for->fgam->10fthf->thf->mlthf->ser-L->acser->ac->actp->accoa->mal-L->oaa
31 pyr->pep->2pg->3pg->3php->pser-L->ser-L->acser->ac->actp->accoa->mal-L->oaa
32 pyr->pep->2pg->3pg->3php->pser-L->ser-L->acser->ac->actp->accoa->cit->oaa
33 pyr->23dhdp->thdp->sl2a6o->sl26da->succ->succoa->mmcoa-R->mmcoa-S->ppcoa->coa->accoa->mal-L->oaa
34 pyr->accoa->acgam1p->uacgam->ump->uri->r1p->r5p->g3p->13dpg->3pg->2pg->pep->oaa
35 pyr->for->fgam->10fthf->fprica->imp->dcamp->fum->succ->succoa->coa->accoa->cit->oaa
36 pyr->pep->2pg->3pg->13dpg->g3p->r5p->r1p->ins->imp->dcamp->fum->mal-L->oaa
37 pyr->23dhdp->thdp->sl2a6o->sl26da->succ->succoa->mmcoa-R->mmcoa-S->ppcoa->coa->accoa->cit->oaa
38 pyr->accoa->acgam1p->uacgam->ump->uri->rib-D->r5p->prpp->imp->dcamp->fum->mal-L->oaa
39 pyr->for->fgam->10fthf->thf->mlthf->dtmp->thymd->2dr1p->2dr5p->acald->accoa->cit->oaa
40 pyr->pep->2pg->3pg->13dpg->g3p->r5p->prpp->gmp->imp->dcamp->fum->mal-L->oaa
41 pyr->for->fgam->10fthf->methf->mlthf->dtmp->thymd->2dr1p->2dr5p->acald->accoa->cit->oaa
42 pyr->for->fgam->10fthf->fprica->imp->dcamp->fum->succ->succoa->coa->accoa->mal-L->oaa
43 pyr->accoa->acgam1p->uacgam->ump->uri->rib-D->r5p->g3p->13dpg->3pg->2pg->pep->oaa
44 pyr->accoa->2aobut->athr-L->gly->ser-L->cmp->r5p->g3p->13dpg->3pg->2pg->pep->oaa
45 pyr->accoa->acgam1p->uacgam->ump->uri->r1p->adn->ins->imp->dcamp->fum->mal-L->oaa
46 pyr->for->fgam->10fthf->methf->mlthf->dtmp->thymd->2dr1p->2dr5p->acald->accoa->mal-L->oaa
47 pyr->accoa->2aobut->athr-L->gly->gar->fgam->10fthf->fprica->imp->dcamp->fum->mal-L->oaa
48 pyr->accoa->acgam1p->uacgam->ump->uri->r1p->r15bp->prpp->imp->dcamp->fum->mal-L->oaa
49 pyr->pep->kdo8p->kdo->ckdo->cmp->r5p->r1p->ins->imp->dcamp->fum->mal-L->oaa
50 pyr->ala-L->ala-D->alaala->ugmda->ump->uri->r1p->ins->imp->dcamp->fum->mal-L->oaa
51 pyr->for->fgam->10fthf->thf->mlthf->dtmp->thymd->2dr1p->2dr5p->acald->accoa->mal-L->oaa
52 pyr->pep->kdo8p->kdo->ckdo->cmp->r5p->prpp->gmp->imp->dcamp->fum->mal-L->oaa
53 pyr->accoa->acgam1p->uacgam->ump->uri->r1p->r5p->prpp->imp->dcamp->fum->mal-L->oaa

54 pyr->pep->2pg->3pg->3php->pser-L->ser-L->gly->2aobut->athr-L->acald->accoa->mal-L->oaa

55 pyr->pep->2pg->3pg->3php->pser-L->ser-L->gly->2aobut->athr-L->acald->accoa->cit->oaa

56 pyr->accoa->2aobut->athr-L->gly->ser-L->cmp->r5p->prpp->imp->dcamp->fum->mal-L->oaa

57 pyr->accoa->2aobut->athr-L->gly->mlthf->methf->10fthf->fprica->imp->dcamp->fum->mal-L->oaa

58 pyr->accoa->2aobut->athr-L->gly->mlthf->thf->10fthf->fprica->imp->dcamp->fum->mal-L->oaa

59 pyr->accoa->acgam1p->uacgam->ump->uri->r1p->ins->hxan->imp->dcamp->fum->mal-L->oaa

60 pyr->accoa->acgam1p->uacgam->ump->uri->r1p->gsn->gmp->imp->dcamp->fum->mal-L->oaa

61 pyr->accoa->2aobut->athr-L->gly->ser-L->g3p->r5p->prpp->imp->dcamp->fum->mal-L->oaa

62 pyr->23dhdp->thdp->sl2a6o->sl26da->succ->succoa->coa->accoa->cit->acon-C->icit->glx->mal-L->oaa

63 pyr->for->fgam->10fthf->methf->mlthf->ser-L->cmp->r5p->g3p->13dpg->3pg->2pg->pep->oaa

64 pyr->for->fgam->10fthf->methf->mlthf->dtmp->thymd->2dr1p->2dr5p->acald->ac->accoa->cit->oaa

65 pyr->for->fgam->fpram->air->5caiz->5aizc->25aics->fum->succ->succoa->coa->accoa->mal-L->oaa

66 pyr->accoa->2aobut->athr-L->gly->gar->fgam->fpram->air->5caiz->5aizc->25aics->fum->mal-L->oaa

67 pyr->for->fgam->10fthf->methf->mlthf->ser-L->cmp->r5p->prpp->imp->dcamp->fum->mal-L->oaa

68 pyr->for->fgam->fpram->air->5caiz->5aizc->25aics->aicar->fprica->imp->dcamp->fum->mal-L->oaa

69 pyr->23dhdp->thdp->sl2a6o->sl26da->succ->succoa->coa->tdecoa->td2coa->3htdcoa->3otdcoa->accoa->cit->oaa

70 pyr->for->fgam->10fthf->fprica->imp->ins->r1p->r5p->g3p->13dpg->3pg->2pg->pep->oaa

71 pyr->accoa->dtdp4aaddg->dtdp->dttp->dtmp->thymd->2dr1p->2dr5p->g3p->13dpg->3pg->2pg->pep->oaa

72 pyr->for->fgam->10fthf->thf->mlthf->ser-L->g3p->r5p->prpp->imp->dcamp->fum->mal-L->oaa

73 pyr->ala-L->ala-D->alaala->ugmda->ump->uri->rib-D->r5p->g3p->13dpg->3pg->2pg->pep->oaa

74 pyr->for->fgam->10fthf->thf->mlthf->ser-L->cmp->r5p->prpp->imp->dcamp->fum->mal-L->oaa

75 pyr->accoa->2aobut->athr-L->gly->ser-L->cmp->r5p->r1p->ins->imp->dcamp->fum->mal-L->oaa

76 pyr->23dhdp->thdp->sl2a6o->sl26da->succ->succoa->coa->tdecoa->td2coa->3htdcoa->3otdcoa->accoa->mal-L->oaa

77 pyr->for->fgam->10fthf->methf->mlthf->ser-L->g3p->r5p->prpp->imp->dcamp->fum->mal-L->oaa

78 pyr->pep->2pg->3pg->13dpg->g3p->r5p->r1p->ins->hxan->imp->dcamp->fum->mal-L->oaa

79 pyr->accoa->acACP->ACP->octeACP->pa181->cdpdodec11eg->cmp->r5p->g3p->13dpg->3pg->2pg->pep->oaa

80 pyr->23dhdp->thdp->sl2a6o->sl26da->succ->succoa->coa->ddcacoa->dd2coa->3hddcoa->3oddcoa->accoa->cit->oaa

81 pyr->pep->2pg->3pg->3php->pser-L->ser-L->gly->2aobut->athr-L->acald->ac->accoa->cit->oaa

82 pyr->for->fgam->10fthf->fprica->imp->dcamp->amp->r5p->g3p->13dpg->3pg->2pg->pep->oaa

83 pyr->for->fgam->10fthf->thf->mlthf->dtmp->thymd->2dr1p->2dr5p->acald->ac->accoa->mal-L->oaa

84 pyr->23dhdp->thdp->sl2a6o->sl26da->succ->succoa->coa->dcacoa->dc2coa->3hdcoa->3odcoa->accoa->mal-L->oaa

85 pyr->accoa->2aobut->athr-L->gly->mlthf->ser-L->cmp->r5p->g3p->13dpg->3pg->2pg->pep->oaa

86 pyr->ala-L->ala-D->alaala->ugmda->ump->uri->r1p->r5p->g3p->13dpg->3pg->2pg->pep->oaa

87 pyr->pep->2pg->3pg->13dpg->g3p->r5p->r1p->adn->ins->imp->dcamp->fum->mal-L->oaa

88 pyr->23dhdp->thdp->sl2a6o->sl26da->succ->succoa->coa->occoa->oc2coa->3hocoa->3oocoa->accoa->mal-L->oaa

89 pyr->for->fgam->fpram->air->5caiz->5aizc->25aics->fum->succ->succoa->coa->accoa->cit->oaa

90 pyr->accoa->2aobut->athr-L->gly->ser-L->seramp->amp->r5p->g3p->13dpg->3pg->2pg->pep->oaa

91 pyr->for->fgam->10fthf->fprica->imp->ins->rib-D->r5p->g3p->13dpg->3pg->2pg->pep->oaa

92 pyr->accoa->acACP->ACP->hdeACP->pa161->cdpdhdec9eg->cmp->r5p->g3p->13dpg->3pg->2pg->pep->oaa

93 pyr->for->fgam->10fthf->udpLa4fn->udp->dudp->dump->duri->2dr1p->2dr5p->acald->accoa->mal-L->oaa

94 pyr->accoa->acgam1p->uacgam->ump->uri->rib-D->r5p->prpp->gmp->imp->dcamp->fum->mal-L->oaa

95 pyr->pep->kdo8p->kdo->ckdo->cmp->cytd->uri->r1p->ins->imp->dcamp->fum->mal-L->oaa

96 pyr->accoa->2aobut->athr-L->gly->ser-L->seramp->amp->atp->itp->imp->dcamp->fum->mal-L->oaa

97 pyr->for->fgam->10fthf->methf->mlthf->dtmp->thymd->2dr1p->2dr5p->acald->ac->accoa->mal-L->oaa

98 pyr->pep->2pg->3pg->3php->pser-L->ser-L->gly->2aobut->athr-L->acald->ac->accoa->mal-L->oaa

99 pyr->accoa->acgam1p->uacgam->udp->utp->ump->uri->r1p->ins->imp->dcamp->fum->mal-L->oaa

100 pyr->for->fgam->10fthf->thf->mlthf->dtmp->thymd->2dr1p->2dr5p->acald->ac->accoa->cit->oaa

Frequency of arcs in the 100-shortest CFPs between pyr and oaa

Oxic conditions

**Arcs**	**Frequency**	**Group**
mal-L --> oaa	75	3
fum --> mal-L	62	3
succ --> fum	55	3
pyr --> ac	43	3
pyr --> accoa	34	3
ac --> accoa	32	3
accoa --> cit	30	3
coa --> ppcoa	24	3
ac --> actp	23	3
actp --> accoa	23	3
coa --> succoa	22	3
succoa --> succ	21	3
sl2a6o --> sl26da	21	3
sl26da --> succ	21	3
cit --> acon-C	19	3
acon-C --> icit	19	3
accoa --> coa	18	3
ppcoa --> succoa	17	3
succoa --> sl2a6o	17	3
pep --> oaa	14	3
accoa --> hxcoa	14	3
hxcoa --> coa	14	3
accoa --> malcoa	14	3
malcoa --> coa	14	3
2pg --> pep	13	3
pyr --> for	12	3
for --> fgam	12	3
ser-L --> acser	12	3
acser --> ac	12	3
cit --> oaa	11	3
icit --> glx	11	3
fgam --> 10fthf	11	3
mlthf --> ser-L	11	3
accoa --> mal-L	10	3
glx --> 2h3oppan	8	3
3pg --> 2pg	8	3
pyr --> pep	7	3
ppcoa --> 2mcit	7	3
2mcit --> 2mcacn	7	3
2mcacn --> micit	7	3
micit --> succ	7	3
2h3oppan --> glyc-R	5	2
glyc-R --> 2pg	5	2
icit --> akg	5	2
pep --> 2pg	5	2
2pg --> 3pg	5	2
10fthf --> methf	5	2
methf --> mlthf	5	2
succoa --> sucarg	5	2
sucarg --> sucorn	5	2
sucorn --> sucgsa	5	2

sucgsa --> sucglu	5	2
sucglu --> succ	5	2
10fthf --> thf	5	2
thf --> mlthf	5	2
ser-L --> g3p	5	2
g3p --> 13dpg	5	2
13dpg --> 3pg	5	2
pyr --> 23dhdp	4	2
23dhdp --> thdp	4	2
thdp --> sl2a6o	4	2
imp --> dcamp	4	2
dcamp --> fum	4	2
akg --> succoa	4	2
glx --> mal-L	3	2
icit --> succ	3	2
succ --> succoa	3	2
coa --> accoa	3	2
2h3oppan --> hpyr	3	2
hpyr --> glyc-R	3	2
glyc-R --> 3pg	3	2
accoa --> 2aobut	3	2
2aobut --> athr-L	3	2
athr-L --> gly	3	2
succoa --> coa	2	1
accoa --> acglu	2	1
acglu --> acg5p	2	1
acg5p --> acg5sa	2	1
acg5sa --> acorn	2	1
acorn --> orn	2	1
orn --> citr-L	2	1
citr-L --> argsuc	2	1
argsuc --> fum	2	1
gly --> ser-L	2	1
r5p --> prpp	2	1
prpp --> imp	2	1
10fthf --> fprica	1	1
fprica --> imp	1	1
fgam --> fpram	1	1
fpram --> air	1	1
air --> 5caiz	1	1
5caiz --> 5aizc	1	1
5aizc --> 25aics	1	1
25aics --> fum	1	1
gly --> mlthf	1	1
pep --> kdo8p	1	1
kdo8p --> kdo	1	1
kdo --> ckdo	1	1
ckdo --> cmp	1	1
cmp --> r5p	1	1
akg --> glu-L	1	1
glu-L --> 4abut	1	1
4abut --> sucsal	1	1
sucsal --> succ	1	1
3pg --> 13dpg	1	1
13dpg --> g3p	1	1
g3p --> r5p	1	1

accoa --> acgam1p	1	1
acgam1p --> uacgam	1	1
uacgam --> ump	1	1
ump --> uri	1	1
uri --> r1p	1	1
r1p --> ins	1	1
ins --> imp	1	1
succoa --> mmcoa-R	1	1
mmcoa-R --> mmcoa-S	1	1
mmcoa-S --> ppcoa	1	1
ppcoa --> coa	1	1

Anoxic conditions

Arcs	Frequency	Group
mal-L --> oaa	58	3
pyr --> accoa	35	3
imp --> dcamp	35	3
fum --> mal-L	35	3
pyr --> for	34	3
for --> fgam	34	3
dcamp --> fum	34	3
fgam --> 10fthf	31	3
accoa --> cit	25	3
pep --> oaa	23	3
2pg --> pep	22	3
accoa --> mal-L	21	3
3pg --> 2pg	20	3
cit --> oaa	19	3
pyr --> pep	18	3
g3p --> 13dpg	18	3
13dpg --> 3pg	18	3
2aobut --> athr-L	17	3
mlthf --> ser-L	17	3
succ --> succoa	14	3
ump --> uri	14	3
accoa --> 2aobut	13	3
athr-L --> gly	13	3
pep --> 2pg	13	3
2pg --> 3pg	13	3
r5p --> prpp	13	3
r5p --> g3p	13	3
succoa --> coa	12	3
ser-L --> acser	12	3
acser --> ac	12	3
ac --> accoa	12	3
10fthf --> methf	12	3
methf --> mlthf	12	3
cmp --> r5p	12	3
10fthf --> thf	11	3
thf --> mlthf	11	3
accoa --> acgam1p	11	3
acgam1p --> uacgam	11	3
uri --> r1p	11	3
prpp --> imp	11	3
fprica --> imp	10	3
pyr --> 23dhdp	10	3
23dhdp --> thdp	10	3
thdp --> sl2a6o	10	3
sl2a6o --> sl26da	10	3
sl26da --> succ	10	3
uacgam --> ump	10	3
2dr1p --> 2dr5p	10	3
10fthf --> fprica	9	3
coa --> accoa	9	3
r1p --> ins	9	3
ins --> imp	9	3
dtmp --> thymd	9	3

thymd --> 2dr1p	9	3
2dr5p --> acald	9	3
3pg --> 3php	8	3
3php --> pser-L	8	3
pser-L --> ser-L	8	3
g3p --> r5p	8	3
mlthf --> dtmp	8	3
gly --> ser-L	7	3
ser-L --> g3p	7	3
acald --> accoa	7	3
ser-L --> cmp	7	3
cit --> acon-C	6	3
acon-C --> icit	6	3
icit --> glx	6	3
ac --> actp	6	3
actp --> accoa	6	3
acald --> ac	6	3
fgam --> fpram	5	2
fpram --> air	5	2
air --> 5caiz	5	2
5caiz --> 5aizc	5	2
5aizc --> 25aics	5	2
3pg --> 13dpg	5	2
13dpg --> g3p	5	2
r5p --> r1p	5	2
rib-D --> r5p	5	2
25aics --> fum	4	2
glx --> 2h3oppan	4	2
gly --> mlthf	4	2
pep --> kdo8p	4	2
kdo8p --> kdo	4	2
kdo --> ckdo	4	2
ckdo --> cmp	4	2
r1p --> r5p	4	2
fum --> succ	4	2
uri --> rib-D	4	2
gmp --> imp	4	2
ser-L --> gly	4	2
gly --> 2aobut	4	2
athr-L --> acald	4	2
prpp --> gmp	3	2
pyr --> ala-L	3	2
ala-L --> ala-D	3	2
ala-D --> alaala	3	2
alaala --> ugmda	3	2
ugmda --> ump	3	2
glx --> mal-L	2	1
2h3oppan --> glyc-R	2	1
glyc-R --> 2pg	2	1
glyc-R --> 3pg	2	1
2h3oppan --> hpyr	2	1
hpyr --> glyc-R	2	1
succoa --> mmcoa-R	2	1
mmcoa-R --> mmcoa-S	2	1
mmcoa-S --> ppcoa	2	1
ppcoa --> coa	2	1

r1p --> adn	2	1
adn --> ins	2	1
gly --> gar	2	1
gar --> fgam	2	1
ins --> hxan	2	1
hxan --> imp	2	1
coa --> tdecoa	2	1
tdecoa --> td2coa	2	1
td2coa --> 3htdcoa	2	1
3htdcoa --> 3otdcoa	2	1
3otdcoa --> accoa	2	1
imp --> ins	2	1
accoa --> acACP	2	1
acACP --> ACP	2	1
amp --> r5p	2	1
ser-L --> seramp	2	1
seramp --> amp	2	1
accoa --> acglu	1	1
acglu --> acg5p	1	1
acg5p --> acg5sa	1	1
acg5sa --> acorn	1	1
acorn --> orn	1	1
orn --> citr-L	1	1
citr-L --> argsuc	1	1
argsuc --> fum	1	1
r1p --> r15bp	1	1
r15bp --> prpp	1	1
mlthf --> methf	1	1
methf --> 10fthf	1	1
mlthf --> thf	1	1
thf --> 10fthf	1	1
r1p --> gsn	1	1
gsn --> gmp	1	1
25aics --> aicar	1	1
aicar --> fprica	1	1
ins --> r1p	1	1
accoa --> dtdp4aaddg	1	1
dtdp4aaddg --> dtdp	1	1
dtdp --> dttp	1	1
dttp --> dtmp	1	1
2dr5p --> g3p	1	1
ACP --> octeACP	1	1
octeACP --> pa181	1	1
pa181 --> cdpdodec11eg	1	1
cdpdodec11eg --> cmp	1	1
coa --> ddcacoa	1	1
ddcacoa --> dd2coa	1	1
dd2coa --> 3hddcoa	1	1
3hddcoa --> 3oddcoa	1	1
3oddcoa --> accoa	1	1
dcamp --> amp	1	1
coa --> dcacoa	1	1
dcacoa --> dc2coa	1	1
dc2coa --> 3hdcoa	1	1
3hdcoa --> 3odcoa	1	1
3odcoa --> accoa	1	1

coa --> occoa	1	1
occoa --> oc2coa	1	1
oc2coa --> 3hocoa	1	1
3hocoa --> 3oocoa	1	1
3oocoa --> accoa	1	1
ins --> rib-D	1	1
ACP --> hdeACP	1	1
hdeACP --> pa161	1	1
pa161 --> cdpdhdec9eg	1	1
cdpdhdec9eg --> cmp	1	1
10fthf --> udpLa4fn	1	1
udpLa4fn --> udp	1	1
udp --> dudp	1	1
dudp --> dump	1	1
dump --> duri	1	1
duri --> 2dr1p	1	1
cmp --> cytd	1	1
cytd --> uri	1	1
amp --> atp	1	1
atp --> itp	1	1
itp --> imp	1	1
uacgam --> udp	1	1
udp --> utp	1	1
utp --> ump	1	1

Frequency of metabolites involved in the 100-shortest CFPs between pyr and oaa

Oxic conditions

**Abbrev.**	**OfficialName**	**KeggID**	**Freq**
accoa	Acetyl-CoA	C00024	92
mal-L	L-Malate	C00149	75
fum	Fumarate	C00122	62
succ	Succinate	C00042	58
ac	Acetate	C00033	55
pyr	Pyruvate	C00022	100
oaa	Oxaloacetate	C00036	100
coa	Coenzyme A	C00010	49
succoa	Succinyl-CoA	C00091	46
cit	Citrate	C00158	30
ppcoa	Propanoyl-CoA	C00100	25
actp	Acetyl phosphate	C00227	23
sl2a6o	N-Succinyl-2-L-amino-6-oxoheptanedioate	C04462	21
sl26da	N-Succinyl-LL-2,6-diaminoheptanedioate	C04421	21
pep	Phosphoenolpyruvate	C00074	20
acon-C	cis-Aconitate	C00417	19
icit	Isocitrate	C00311	19
2pg	D-Glycerate 2-phosphate	C00631	18
ser-L	L-Serine	C00065	17
hxcoa	Hexanoyl-CoA (n-C6:0CoA)	C05270	14
malcoa	Malonyl-CoA	C00083	14
3pg	3-Phospho-D-glycerate	C00197	13
for	Formate	C00058	12
fgam	N2-Formyl-N1-(5-phospho-D-ribosyl)glycinamide	C04376	12
acser	O-Acetyl-L-serine	C00979	12
glx	Glyoxylate	C00048	11
10fthf	10-Formyltetrahydrofolate	C00234	11
mlthf	5,10-Methylenetetrahydrofolate	C00143	11
2h3oppan	2-Hydroxy-3-oxopropanoate	C01146	8
glyc-R	(R)-Glycerate	C00258	8
2mcit	2-Methylcitrate	C02225	7
2mcacn	cis-2-Methylaconitate	C04225	7
micit	methylisocitrate	C04593	7
g3p	Glyceraldehyde 3-phosphate	C00661	6
13dpg	3-Phospho-D-glyceroyl phosphate	C00236	6
akg	2-Oxoglutarate	C00026	5
methf	5,10-Methenyltetrahydrofolate	C00445	5
sucarg	N2-Succinyl-L-arginine	C03296	5
sucorn	N2-Succinyl-L-ornithine	C03415	5
sucgsa	N2-Succinyl-L-glutamate 5-semialdehyde	C05932	5
sucglu	N2-Succinyl-L-glutamate	C05931	5
thf	5,6,7,8-Tetrahydrofolate	C00101	5
23dhdp	2,3-Dihydrodipicolinate	C03340	4
thdp	2,3,4,5-Tetrahydrodipicolinate	C03972	4
imp	IMP	C00130	4
dcamp	N6-(1,2-Dicarboxyethyl)-AMP	C03794	4
3php	3-Phosphohydroxypyruvate	C03232	4
pser-L	O-Phospho-L-serine	C01005	4
hpyr	Hydroxypyruvate	C00168	3
2aobut	L-2-Amino-3-oxobutanoate	C03508	3
athr-L	L-Allo-threonine	C05519	3

gly	Glycine	C00037	3
acglu	N-Acetyl-L-glutamate	C00624	2
acg5p	N-Acetyl-L-glutamyl 5-phosphate	C04133	2
acg5sa	N-Acetyl-L-glutamate 5-semialdehyde	C01250	2
acorn	N2-Acetyl-L-ornithine	C00437	2
orn	Ornithine	C01602	2
citr-L	L-Citrulline	C00327	2
argsuc	N(omega)-(L-Arginino)succinate	C03406	2
r5p	alpha-D-Ribose 5-phosphate	C00117	2
prpp	5-Phospho-alpha-D-ribose 1-diphosphate	C00119	2
fprica	5-Formamido-1-(5-phospho-D-ribosyl)imidazole-4-carboxamide	C04734	1
fpram	2-(Formamido)-N1-(5-phospho-D-ribosyl)acetamidine	C04640	1
air	5-amino-1-(5-phospho-D-ribosyl)imidazole	C03373	1
5caiz	5-phosphoribosyl-5-carboxyaminoimidazole	--------	1
5aizc	5-amino-1-(5-phospho-D-ribosyl)imidazole-4-carboxylate	C04751	1
25aics	(S)-2-[5-Amino-1-(5-phospho-D-ribosyl)imidazole-4-carboxamido]succinate	C04823	1
kdo8p	3-Deoxy-D-manno-octulosonate 8-phosphate	C04478	1
kdo	3-Deoxy-D-manno-2-octulosonate	C01187	1
ckdo	CMP-3-deoxy-D-manno-octulosonate	--------	1
cmp	CMP	C00055	1
glu-L	L-Glutamate	C00025	1
4abut	4-Aminobutanoate	C00334	1
sucsal	Succinic semialdehyde	C00232	1
acgam1p	N-Acetyl-D-glucosamine 1-phosphate	C04256	1
uacgam	UDP-N-acetyl-D-glucosamine	C00043	1
ump	UMP	C00105	1
uri	Uridine	C00299	1
r1p	alpha-D-Ribose 1-phosphate	C00442	1
ins	Inosine	C00294	1
mmcoa-R	(R)-Methylmalonyl-CoA	C01213	1
mmcoa-S	(S)-Methylmalonyl-CoA	C00683	1

Anoxic conditions

Abbreviation	OfficialName	KeggID	Freq
accoa	Acetyl-CoA	C00024	74
mal-L	L-Malate	C00149	58
pyr	Pyruvate	C00022	100
oaa	Oxaloacetate	C00036	100
pep	Phosphoenolpyruvate	C00074	40
fum	Fumarate	C00122	39
imp	IMP	C00130	37
fgam	N2-Formyl-N1-(5-phospho-D-ribosyl)glycinamide	C04376	36
dcamp	N6-(1,2-Dicarboxyethyl)-AMP	C03794	35
2pg	D-Glycerate 2-phosphate	C00631	35
for	Formate	C00058	34
10fthf	10-Formyltetrahydrofolate	C00234	33
3pg	3-Phospho-D-glycerate	C00197	33
ser-L	L-Serine	C00065	32
r5p	alpha-D-Ribose 5-phosphate	C00117	31
mlthf	5,10-Methylenetetrahydrofolate	C00143	27
g3p	Glyceraldehyde 3-phosphate	C00661	26
cit	Citrate	C00158	25
13dpg	3-Phospho-D-glyceroyl phosphate	C00236	23
ac	Acetate	C00033	18
2aobut	L-2-Amino-3-oxobutanoate	C03508	17
athr-L	L-Allo-threonine	C05519	17
gly	Glycine	C00037	17
r1p	alpha-D-Ribose 1-phosphate	C00442	17
uri	Uridine	C00299	15
succ	Succinate	C00042	14
succoa	Succinyl-CoA	C00091	14
coa	Coenzyme A	C00010	14
ump	UMP	C00105	14
prpp	5-Phospho-alpha-D-ribose 1-diphosphate	C00119	14
methf	5,10-Methenyltetrahydrofolate	C00445	13
ins	Inosine	C00294	13
cmp	CMP	C00055	13
acald	Acetaldehyde	C00084	13
thf	5,6,7,8-Tetrahydrofolate	C00101	12
acser	O-Acetyl-L-serine	C00979	12
acgam1p	N-Acetyl-D-glucosamine 1-phosphate	C04256	11
uacgam	UDP-N-acetyl-D-glucosamine	C00043	11
fprica	5-Formamido-1-(5-phospho-D-ribosyl)imidazole-4-carboxamide	C04734	10
23dhdp	2,3-Dihydrodipicolinate	C03340	10
thdp	2,3,4,5-Tetrahydrodipicolinate	C03972	10
sl2a6o	N-Succinyl-2-L-amino-6-oxoheptanedioate	C04462	10
sl26da	N-Succinyl-LL-2,6-diaminoheptanedioate	C04421	10
2dr1p	2-Deoxy-D-ribose 1-phosphate	C00672	10
2dr5p	2-Deoxy-D-ribose 5-phosphate	C00673	10
dtmp	dTMP	C00364	9
thymd	Thymidine	C00214	9
3php	3-Phosphohydroxypyruvate	C03232	8
pser-L	O-Phospho-L-serine	C01005	8
acon-C	cis-Aconitate	C00417	6
icit	Isocitrate	C00311	6
glx	Glyoxylate	C00048	6
actp	Acetyl phosphate	C00227	6

fpram	2-(Formamido)-N1-(5-phospho-D-ribosyl)acetamidine	C04640	5
air	5-amino-1-(5-phospho-D-ribosyl)imidazole	C03373	5
5caiz	5-phosphoribosyl-5-carboxyaminoimidazole	--------	5
5aizc	5-amino-1-(5-phospho-D-ribosyl)imidazole-4-carboxylate	C04751	5
25aics	(S)-2-[5-Amino-1-(5-phospho-D-ribosyl)imidazole-4-carboxamido]succinate	C04823	5
rib-D	D-Ribose	C00121	5
2h3oppan	2-Hydroxy-3-oxopropanoate	C01146	4
glyc-R	(R)-Glycerate	C00258	4
kdo8p	3-Deoxy-D-manno-octulosonate 8-phosphate	C04478	4
kdo	3-Deoxy-D-manno-2-octulosonate	C01187	4
ckdo	CMP-3-deoxy-D-manno-octulosonate	--------	4
gmp	GMP	C00144	4
ala-L	L-Alanine	C00041	3
ala-D	D-Alanine	C00133	3
alaala	D-Alanyl-D-alanine	C00993	3
ugmda	UDP-N-acetylmuramoyl-L-alanyl-D-glutamyl-meso-2,6-diaminopimeloyl	C04882	3
amp	AMP	C00020	3
hpyr	Hydroxypyruvate	C00168	2
mmcoa-R	(R)-Methylmalonyl-CoA	C01213	2
mmcoa-S	(S)-Methylmalonyl-CoA	C00683	2
ppcoa	Propanoyl-CoA	C00100	2
adn	Adenosine	C00212	2
gar	N1-(5-Phospho-D-ribosyl)glycinamide	C03838	2
hxan	Hypoxanthine	C00262	2
tdecoa	Tetradecenoyl-CoA (n-C14:1CoA)	--------	2
td2coa	trans-Tetradec-2-enoyl-CoA	C05273	2
3htdcoa	(S)-3-Hydroxytetradecanoyl-CoA	C05260	2
3otdcoa	3-Oxotetradecanoyl-CoA	C05261	2
acACP	Acetyl-ACP	--------	2
ACP	acyl carrier protein	C00229	2
seramp	L-seryl-AMP	--------	2
udp	UDP	C00015	2
acglu	N-Acetyl-L-glutamate	C00624	1
acg5p	N-Acetyl-L-glutamyl 5-phosphate	C04133	1
acg5sa	N-Acetyl-L-glutamate 5-semialdehyde	C01250	1
acorn	N2-Acetyl-L-ornithine	C00437	1
orn	Ornithine	C01602	1
citr-L	L-Citrulline	C00327	1
argsuc	N(omega)-(L-Arginino)succinate	C03406	1
r15bp	D-Ribose 1,5-bisphosphate	C01151	1
gsn	Guanosine	C00387	1
aicar	5-Amino-1-(5-Phospho-D-ribosyl)imidazole-4-carboxamide	C04677	1
dtdp4aaddg	dTDP-4-acetamido-4,6-dideoxy-D-galactose	--------	1
dtdp	dTDP	C00363	1
dttp	dTTP	C00459	1
octeACP	cis-octadec-11-enoyl-[acyl-carrier protein] (n-C18:1)	--------	1
pa181	1,2-dioctadec-11-enoyl-sn-glycerol 3-phosphate	C00416	1
cdpdodec11eg	CDP-1,2-dioctadec-11-enoylglycerol	C00269	1
ddcacoa	Dodecanoyl-CoA (n-C12:0CoA)	C01832	1
dd2coa	trans-Dodec-2-enoyl-CoA	C03221	1
3hddcoa	(S)-3-Hydroxydodecanoyl-CoA	C05262	1
3oddcoa	3-Oxododecanoyl-CoA	C05263	1
dcacoa	Decanoyl-CoA (n-C10:0CoA)	C05274	1
dc2coa	trans-Dec-2-enoyl-CoA	C05275	1
3hdcoa	(S)-3-Hydroxydecanoyl-CoA	C05264	1
3odcoa	3-Oxodecanoyl-CoA	C05265	1

occoa	Octanoyl-CoA (n-C8:0CoA)	C01944	1
oc2coa	trans-Oct-2-enoyl-CoA	C05276	1
3hocoa	(S)-3-Hydroxyoctanoyl-CoA	C05266	1
3oocoa	3-Oxooctanoyl-CoA	C05267	1
hdeACP	cis-hexadec-9-enoyl-[acyl-carrier protein] (n-C16:1)	--------	1
pa161	1,2-dihexadec-9-enoyl-sn-glycerol 3-phosphate	C00416	1
cdpdhdec9eg	CDP-1,2-dihexadec-9-enoylglycerol	C00269	1
udpLa4fn	uridine 5''-diphospho-{beta}-4-deoxy-4-formamido-L-arabinose	--------	1
dudp	dUDP	C01346	1
dump	dUMP	C00365	1
duri	Deoxyuridine	C00526	1
cytd	Cytidine	C00475	1
atp	ATP	C00002	1
itp	ITP	C00081	1
utp	UTP	C00075	1
